# Characterization of TBP and TAFs in Mungbean (*Vigna radiata* L.) and Their Potential Involvement in Abiotic Stress Response

**DOI:** 10.3390/ijms25179558

**Published:** 2024-09-03

**Authors:** Ranran Wu, Qiyuan Jia, Yingjian Guo, Yun Lin, Jinyang Liu, Jingbin Chen, Qiang Yan, Na Yuan, Chenchen Xue, Xin Chen, Xingxing Yuan

**Affiliations:** 1 Institute of Industrial Crops, Jiangsu Academy of Agricultural Sciences, Nanjing 210014, China; rrwu@jaas.ac.cn (R.W.); 2021116004@stu.njau.edu.cn (Q.J.); 15216036216@163.com (Y.G.); 20180006@jaas.ac.cn (Y.L.); 20200024@jaas.ac.cn (J.L.); chenjingbin@jaas.ac.cn (J.C.); yanqiang@jaas.ac.cn (Q.Y.); thefuries@163.com (N.Y.); xuecc@jaas.ac.cn (C.X.); 2 Jiangsu Key Laboratory for Horticultural Crop Genetic Improvement, Nanjing 210014, China; 3 College of Life Sciences, Nanjing Agricultural University, Nanjing 210095, China

**Keywords:** mungbean, TATA-binding protein, TAFs, abiotic stress, salt, water deficit, heat, cold

## Abstract

The TATA-box binding protein (TBP) and TBP-associated factors (TAFs) constitute the transcription factor IID (TFIID), a crucial component of RNA polymerase II, essential for transcription initiation and regulation. Several TFIID subunits are shared with the Spt–Ada–Gcn5–acetyltransferase (SAGA) coactivator complex. Recent research has revealed the roles of TBP and TAFs in organogenesis and stress adaptation. In this study, we identified 1 TBP and 21 putative TAFs in the mungbean genome, among which *VrTAF5*, *VrTAF6*, *VrTAF8*, *VrTAF9*, *VrTAF14*, and *VrTAF15* have paralogous genes. Their potential involvement in abiotic stress responses was also investigated here, including high salinity, water deficit, heat, and cold. The findings indicated that distinct genes exerted predominant influences in the response to different abiotic stresses through potentially unique mechanisms. Specifically, under salt stress, *VrTBP*, *VrTAF2*, and *VrTAF15–1* were strongly induced, while *VrTAF10*, *VrTAF11*, and *VrTAF13* acted as negative regulators. In the case of water-deficit stress, it was likely that *VrTAF1*, *VrTAF2*, *VrTAF5–2*, *VrTAF9*, and *VrTAF15–1* were primarily involved. Additionally, in response to changes in ambient temperature, it was possible that genes such as *VrTAF5–1*, *VrTAF6–1*, *VrTAF9–2*, *VrTAF10*, *VrTAF13*, *VrTAF14b–2*, and *VrTAF15–1* might play a dominant role. This comprehensive exploration of *VrTBP* and *VrTAFs* can offer a new perspective on understanding plant stress responses and provide valuable insights into breeding improvement.

## 1. Introduction

In eukaryotes, three canonical DNA-dependent RNA polymerases (Pol I, II, and III) are responsible for transcribing ribosomal RNA genes, protein-coding (as well as several non-coding) genes, and small RNA genes, respectively [1,2]. Despite this diversification, the TATA box binding protein (TBP) is regarded as the central component recruiting the different polymerases by assembling various sets of subunits [3,4]. RNA Pol II has been extensively researched due to its pivotal role in transcribing protein-coding genes. During RNA Pol II-directed transcription, TBP functions within a multi-subunit complex known as transcription factor IID (TFIID)—a general transcription factor that initiates transcription by nucleating pre-initiation complex (PIC) assembly at the core promoter [5,6,7]. TFIID is a complex of over 1 megadalton (MDa), consisting of TBP and 13–14 evolutionarily conserved TBP-associated factors (TAFs) [8,9,10,11]. The Spt-Ada-Gcn5-acetyltransferase (SAGA) coactivator has been shown to share subunits with TFIID and acts synergistically in chromatin modification and TBP loading [12,13]. In higher eukaryotes, the SAGA complex comprises over 20 polypeptide subunits organized into four functional modules: the TAF module, SPT module, histone acetyltransferase (HAT) module, and histone deubiquitination (DUB) module [10,14,15].

Biological development and adaption to the environment are intricately regulated at the genetic level, and the role of general transcription factors (GTFs) cannot be ignored [2,16]. Research has demonstrated that the release of paused Pol II is a critical developmental checkpoint, either by accelerating the speed of gene activation or suppressing transcriptional noise [17]. TFIID and SAGA make overlapping contributions to TBP binding on the core promoter during transcription regulation. While the presence of a TATA box is not essential for transcription, genes containing it in their promoter region are typically associated with responses to environmental stress and exhibit variable expression levels, which depend more strongly on SAGA-mediated TBP binding [12,18]. In fact, only 5–10% of human core promoters and 20% of yeast genes contain a TATA box element [19,20]. However, TFIID may have a more substantial impact on the regulation of constitutive genes, as its binding to promoters lacking a TATA box is governed by TAFs that recognize other essential promoter elements [1,12,19]. TAFs have been shown to exhibit multifaceted activities in the regulation of transcription, encompassing the following roles: (1) facilitating extended promoter recognition; (2) functioning as coactivators that integrate activator-derived signals into the basal transcription machinery; (3) serving as a conduit to facilitate communication between TFIID and nucleosomes; (4) acting as a factor for activator-independent reinitiation; and (5) interacting with epigenetic modification-related components [5,21,22].

TBP and TAFs exhibit a high degree of conservation from yeast to humans, with their roles in regulating cell differentiation, cell cycle, organ development, and stress adaptation well studied in humans [21,22], fruit flies [23,24], and yeast [12,25,26]. Recently, an increasing number of studies have highlighted the important functions of TBP and TAFs in controlling plant development and stress response, particularly in model plants, such as *Arabidopsis*. Two *TBPs* (*TBP1* and *TBP2*) and 18 putative *TAFs* have been identified through the BLAST search in the *Arabidopsis* genome [7,27,28]. Changes in the abundance of *TBP* in *Arabidopsis* can impact plant development throughout the entire growth stage, including shoot apical meristems, leaves, flower organs, fertility, pollen tube growth, and light response [7]. *AtTAF5 (At5g25150)* has been demonstrated to be an indispensable gene for the complete life cycle of plants, playing a role in inflorescence meristems, male gametogenesis, and pollen tube growth [29]. Additionally, *AtTAF10* (*At4g31720*) is involved in plant osmotic stress adaptation, and the overexpression of *AtTAF10* can enhance seed tolerance to salt stress during germination [30]. It has been demonstrated that *AtTAF10* is one of the selectively expressed *TAFs*, showing a preference for transient expression during plant development. *AtTAF10* is enriched in vascular tissue, and the knockdown of *AtTAF10* results in several abnormal phenotypes related to meristem activity and leaf development [31]. Furthermore, It has been found that *TAF12b* (*At1g17440*) controls the *Arabidopsis enhanced ethylene response 4* (*eer4*), *cytokinin hypersensitive 1* (*ckh1*), and *bz1728* suppressor mutants *nobiro*, indicating its pleiotropic function in ethylene response, cytokinin signal, and environment-responsive root growth control through an unfolded protein response (UPR) [32,33,34]. *AtTAF13 (At1g02680)* collaborates with Polycomb Repressive Complex 2 (PRC2) during seed development, and the *taf13* mutation results in embryo arrest and the over-proliferation of the endosperm [35]. Additionally, both *AtTAF14* (*At2g18000*) and *TAF15b* (*At5g58470*) are implicated in flowering regulation by interacting with *FLOWERING LOCUS C* (*FLC*). *AtTAF14* interacts with the FRIGIDA complex, which activates *FLC* to repress flowering [36]. Conversely, *TAF15b* directly represses *FLC* transcription to influence the flowering time, particularly through the autonomous pathway (AP) in *Arabidopsis* [37]. Moreover, *AtTAF15b* has been demonstrated to regulate toll interleukin 1 receptor-type NLR (TNL)-mediated immunity through post-transcriptional RNA processing. Notably, the homologous gene of *AtTAF15b*, *AtTAF15* (*At1g50300*) exhibits different topologies and a distinct function in *Arabidopsis* [38]. The rice genome also contains two genes encoding TBP proteins: OsTBP1 and OsTBP2 [39]. *OsTBP2.1* can bind to the TATA-box of *OsNRT2.3* and alter the ratio of *OsNRT2.3b* to *OsNRT2.3a*, leading to an increased rice yield by promoting nitrogen uptake [40]. Conversely, knockdown lines of *OsTBP2.2* showed heightened sensitivity to drought stress and growth retardation [41]. Furthermore, *OsTAF2* regulates grain size together with its interacting protein POW1 [42,43]. In finger millet (*Eleusine coracana* L.), transcriptome analysis indicates that *TBP* and *TAFs* exhibit strong responses to drought stress [44]. Additionally, *VrTAF5* is identified as a potential gene for mungbean Cercospora leaf-spot disease resistance [45]. Nonetheless, the specific roles of plant *TBP* and *TAFs* are still poorly understood, and further research is urgently needed.

Mungbean (*Vigna radiata* L.) is a significant grain legume crop with a high nutritional value and economic importance [46]. Mungbean plays a crucial role in agricultural sustainability due to its favorable characteristics, such as wide adaptability, nitrogen fixation capacity, short life span, and high biomass production [47,48]. This study systematically identifies mungbeans *VrTBP* and *VrTAFs*, including their encoded genes, structures, conserved domains, and phylogenetic trees. Furthermore, their potential roles in abiotic stress response to high salinity, water deficit, heat, and cold are preliminarily interpreted. These findings offer a new perspective on plant stress response and can facilitate the application of crop improvement under stressful conditions.

## 2. Results

### 2.1. Characterization of TBP and TAF Genes in Mungbean

**Figure 1:**
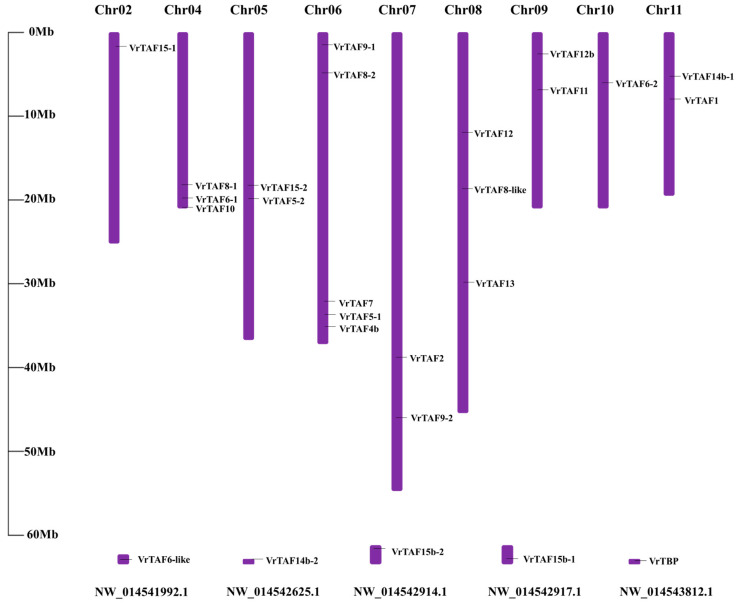
Distribution of *VrTBP* and *VrTAFs* from the mungbean genome.

Based on the initial output of BLASTP and Gcorn plant homology searches, 1 *TBP* and 25 putative *TAFs* are identified from the mungbean genome (Table 1), distributed across 9 chromosomes (except chr01 and chr03) and several unplaced scaffolds (Figure 1). No *TAF3* homologous genes were found in the mungbean genome. It is worth noting that, except for *VrTAF11* and *VrTAF12b*, other *TAFs* tend to have multiple encoding genes (labeled −1, −2, …), various transcripts (labeled X1, X2, …), or a the same transcript with different IDs (labeled (1), (2), …). Specifically, *VrTAF5*, *VrTAF6*, *VrTAF8*, *VrTAF9*, *VrTAF14b*, *VrTAF15*, and *VrTAF15b* have more than two encoding genes. Meanwhile, *VrTAF1*, *VrTAF2*, *VrTAF4b*, *VrTAF5–1*, and *VrTAF9–1* possess two to four transcripts. *VrTAF7*, *VrTAF10*, *VrTAF12*, and *VrTAF13* exhibit multiple transcripts with identical sequences. This phenomenon is not uncommon due to the incomplete assembly of mungbean genomic data and the lack of systematic research on this issue.

### 2.2. Systematic Analysis of Mungbean TBP and Putative TAFs

We further investigate the reliability of the putative *VrTBP* and *VrTAFs* by analyzing the gene structure, protein domains, sequence identity to Arabidopsis homologous proteins, as well as the expression level and phylogenetic tree of TBPs and TAFs proteins from multi-species, respectively. The specific details are as follows.

#### 2.2.1. VrTBP

**Figure 2:**
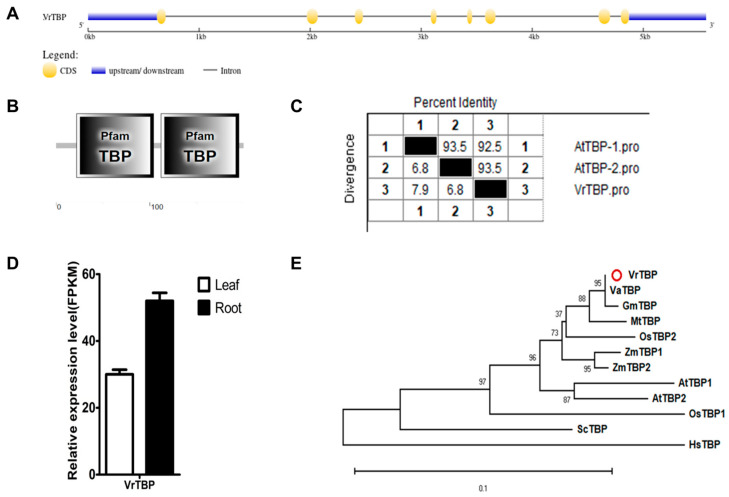
Multi-analysis of *VrTBP.* (**A**) Gene structure of *VrTBP*. (**B**) Conserved domains of VrTBP analyzed by SMART. (**C**) Sequence identity to AtTBP1 and AtTBP2. (**D**) Relative expression level of *VrTBP* in the leaves and roots of mungbean seedlings. (**E**) Phylogenetic tree of TBPs from multi-species, including *Vigna radiata* (Vr), *Vigna angularis* (Va), *Arabidopsis thaliana* (At), *Oryza sativa* (Os), *Glycine max* (Gm), *Medicago truncatula* (Mt), *Zea mays* (Zm), *Homo sapiens* (Hs), and *Saccharomyces cerevisiae* (Sc). The red circle indicates the protein from mungbean.

Only one VrTBP protein, encoded by *LOC106754936* (scaffold NW_014543812.1), has been isolated and characterized in mungbean genome (Table 1 and Figure 1). Despite its small size of 200 amino acids, the gene sequence of *VrTBP* consists of eight exons and seven introns (Figure 2A). VrTBP contains two ‘Pfam TBP’-related domains located at positions ranging from 22–104 aa and 112–195 aa (Figure 2B), and exhibits a very high sequence identity of 92.5% and 93.5% with AtTBP1 and AtTBP2, respectively (Figure 2C and Table S1). Furthermore, *VrTBP* demonstrates significant expression levels in both leavf and root tissues (Figure 2D). TBP exhibits a relatively conservative evolutionary pattern (Figure 2E). Our data indicate that the TBPs from legume crops, such as *Vigna angularis* (Va), *Glycine max* (Gm), and *Medicago truncatula* (Mt), form a distinct cluster, with each of these crops appearing to contain only one TBP, as is the case with mungbean. In contrast, *Arabidopsis thaliana* (At), *Oryza sativa* (Os), and *Zea mays* (Zm) each have two distinct TBPs, but OsTBP1and OsTB2 are not grouped together (Figure 2E).

#### 2.2.2. VrTAF1

**Figure 3:**
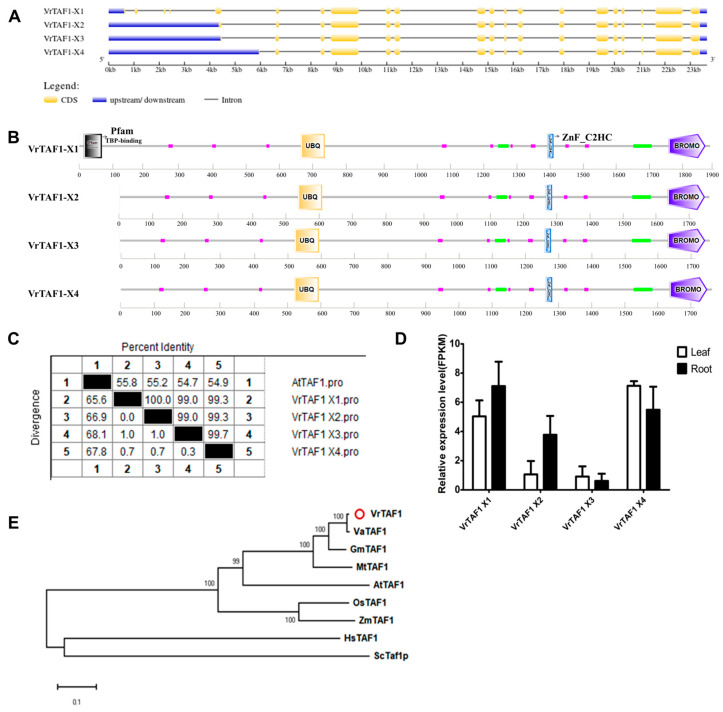
Multi-analysis of *VrTAF1.* (**A**) Gene structure of four transcripts of *VrTAF1*. (**B**) Domains of four VrTAF1 alternative splicing proteins by SMART. (**C**) Sequence identity to AtTAF1. (**D**) Relative expression level of *VrTAF1s* in the leaves and roots of mungbean seedlings. (**E**) Phylogenetic tree of TAF1 from multi-species (see Figure 2), and TAF1–X1 is used here to construct the phylogenetic tree. The red circle indicates the protein from mungbean.

*LOC106777544* encodes the VrTAF1 protein in mungbean, which is located on chr11 (*Vradi11g07970.1*) and has four transcripts, including X1: *XM_014665146.2*, X2: *XM_014665147.2*, X3: *XM_014665148.2*, and X4: *XM_022776030.1* (Table 1 and Figure 1). VrTAF1 is the largest subunit in TFIID with a length of up to 1901 amino acids. *VrTAF1–X1* consists of 21 exons, with alternative splicing mainly occurring on the first five exons, resulting in three additional transcripts (Figure 3A). The absence of a sequence in *VrTAF1–X2/X3/X4* leads to the omission of the ‘Pfam TBP-binding’ domain. Other feature domains, such as UBQ for ubiquitination, ZnF_C2HC, and the BROMO domain for interaction with acetylated lysine are present (Figure 3B). The sequence identity of these four VrTAF1 proteins to AtTAF1 (1919 aa) ranges from 54.9% to 55.8% (Figure 3C and Table S1), with all showing detectable transcriptional levels. Among them, *VrTAF1–X1* and *VrTAF1–X4* exhibit significantly higher expressions (Figure 3D). Phylogenetic analysis reveals that TAF1 proteins from legume crops form a distinct group, with VaTAF1 being the closest relative (Figure 3E). Additionally, TAF1 proteins from monocotyledon and dicotyledon species show clear evolutionary separation. Eventually, *VrTAF1–X1* was chosen for further investigation.

#### 2.2.3. VrTAF2

**Figure 4:**
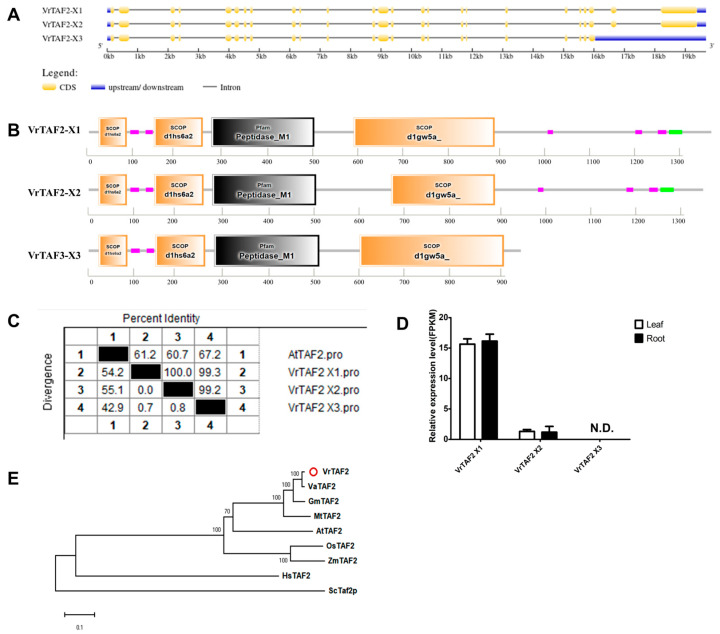
Multi-analysis of *VrTAF2.* (**A**) Gene structure of three transcripts of *VrTAF2*. (**B**) Domain analyzed of three *VrTAF2* alternative splicing proteins by SMART. (**C**) Sequence identity to AtTAF2. (**D**) Relative expression level of *VrTAF2* in the leaves and roots of mungbean seedlings. N.D.: Not detected. (**E**) Phylogenetic tree of TAF2 from multi-species (see Figure 2), and TAF2–X1 is used here to construct the phylogenetic tree. The red circle indicates the protein from mungbean.

VrTAF2 is the second largest subunit of TFIID, encoded by *LOC106768217* on chr07 (*Vradi07g18120.1*), and consists of three alternative splicing transcripts (Table 1 and Figure 1). *VrTAF2–X1* (*XM_014653218.2*) contains 25 exons, while *VrTAF2–X2* (*XM_022783661.1*) lacks the 20th exon, and *VrTAF2–X3* (*XM_022783662.1*) is missing the last two exons (Figure 4A). Protein domain analysis reveals that, compared with *VrTAF2–X1, VrTAF2–X2* has a relatively shorter ‘scop d1gw5a’ domain, and *VrTAF2–X3* lacks the ‘low complexity and coiled coil regions’ on the C-terminal, but retains all four important domains (Figure 4B). Three transcripts all exhibit over 60% identity to AtTAF2 (1390 aa) (Figure 4C and Table S1), however only *VrTAF2–X1* demonstrates a high level of transcript abundance in both the roots and leaves of mungbean (Figure 4D). Therefore, VrTAF2–X1 represents a valuable transcript for further investigation. Similar to TAF1 in evolution, legume TAF2 proteins are closely clustered together, and VrTAF2 is closest to VaTAF2 (Figure 4E).

#### 2.2.4. VrTAF4b

**Figure 5:**
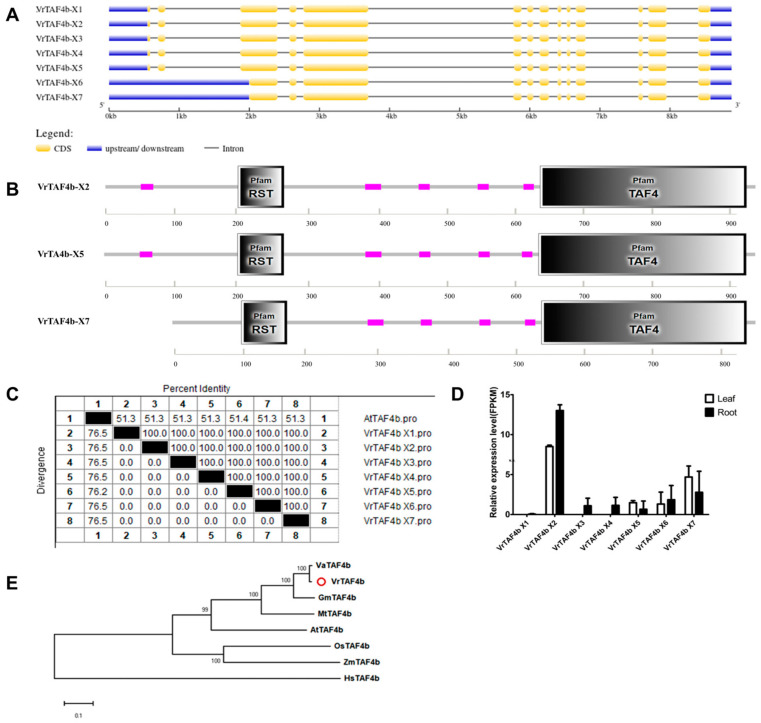
Multi-analysis of *VrTAF4b.* (**A**) Gene structure of seven transcripts of *VrTAF4b*. (**B**) Domains analyzed of VrTAF4b–X2, VrTAF4b–X5, and VrTAF4b–X7 by SMART. (**C**) Sequence identity to AtTAF4b. (**D**) Relative expression level in the leaves and roots of mungbean seedlings. N.D.: Not detected. (**E**) Phylogenetic tree of TAF4b from multi-species (see Figure 2), and VrTAF4b–X2 is used here to construct the phylogenetic tree. The red circle indicates the protein from mungbean.

Up to seven transcripts of *VrTAF4b* are available in the NCBI database (Table 1). Sequence analysis reveals that *VrTAF4b–X1*, *X2*, *X3,* and *X4* share identical sequences, as do *VrTAF4b–X6* and *X7* (Figure 5A,C). Among these, *VrTAF4b–X2*, *X5*, and *X7* are selected for domain analysis due to their relatively higher expression levels (Figure 5D). It is observed that the VrTAF4b–X5 protein lacks a sequence of four amino acids (570–573 LSSQ), which does not appear to affect the main domains, such as RST and TAF4 domains. Pfam RST (for RCD1, SRO, and TAF4) domain is a plant-specific domain-mediated protein–protein interaction. On the other hand, *VrTAF4b–X7* is shorter due to missing the first two exons and part of the third exon, resulting in a loss of ‘low complexity’ on the N-terminal region (Figure 5B). The sequence identity with AtTAF4b (852 aa) is approximately 51.3% (Figure 5C and Table S1). The evolutionary trend of TAF4b shows a similarity to TAF1 and TAF2; however, there is no equivalent TAF4b in yeast (Figure 5E). In conclusion, further study may focus on investigating *VrTAF4b–X2* (*XM_014648978.2*) as a candidate protein due to its highest transcript level.

#### 2.2.5. VrTAF5

**Figure 6:**
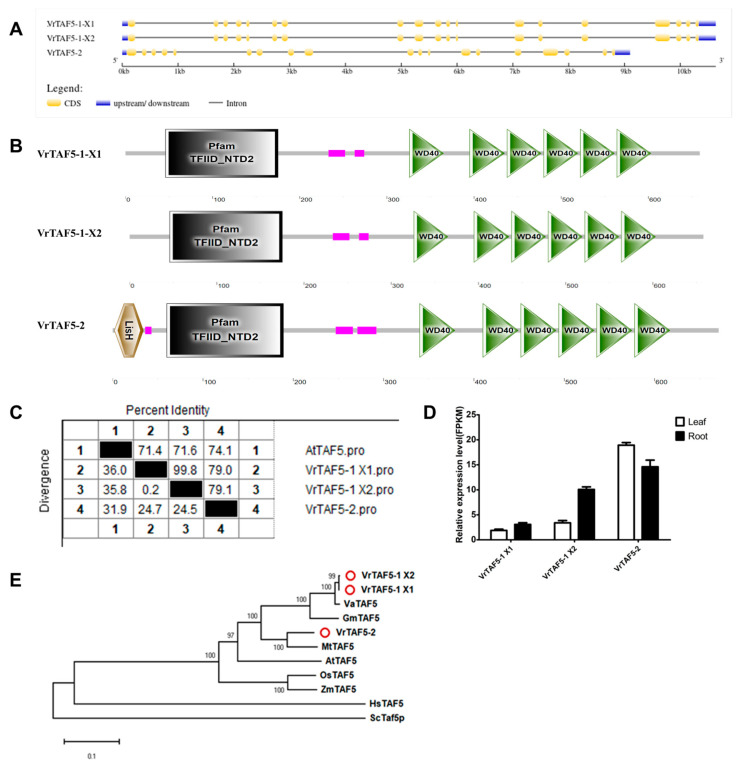
Multi-analysis of *VrTAF5*. (**A**) Gene structures of *VrTAF5–1 X1*, *VrTAF5–1 X2*, and *VrTAF5–2*. (**B**) Domains analyzed of VrTAF5–1 X1, VrTAF5–1 X2, and VrTAF5–2 by SMART. (**C**) Sequence identity to AtTAF5. (**D**) Relative expression levels of *VrTAF5–1 X1*, *VrTAF5–1 X2*, and *VrTAF5–2* in the leaves and roots of mungbean seedlings. (**E**) Phylogenetic tree of TAF5 from multi-species (see Figure 2). The red circles indicate the proteins from mungbean.

Based on the data from the NCBI database, it is observed that *LOC106765332* and *LOC106762344* encode VrTAF5, namely VrTAF5–1 and VrTAF5–2, located on chr06 (*Vradi06g13500.1*) and chr05 (*Vradi05g10920.1*), respectively (Table 1 and Figure 1). *VrTAF5–1* has two transcripts, X1 (*XM_022782472.1*) and X2 (*XM_014649913.2*), with only two amino acid differences at position 616–617 (NR→-K). On the other hand, *VrTAF5–2* contains an equal number of exons as *VrTAF5–1* but has a longer first exon (Figure 6A). All TAF5 proteins are characterized by the presence of highly conserved N-terminal NTD2 and C-terminal WD40 domains, which are also found in VrTAF5 proteins (Figure 6B). Additionally, there is an ‘LisH’ motif in VrTAF5–2 associated with microtubule dynamics, cell migration, nucleokinesis, and chromosome segregation. The sequence identity of all VrTAF5 proteins to AtTAF5 exceeds 70% (Figure 6C and Table S1). Furthermore, the expression of *VrTAF5–1 X1*, *VrTAF5–1 X2*, and *VrTAF5–2* can all be detected in mungbean leaves and roots; however, the relative expression level of *VrTAF5–1 X1* is lower (Figure 6D). In terms of the genetic relationship, VrTAF5-1 is closer to VaTAF5, but VrTAF5–2 is much closer to MtTAF5 (Figure 6E). Based on these findings, *VrTAF5–1 X2* and *VrTAF5–2* are further analyzed.

#### 2.2.6. VrTAF6

**Figure 7:**
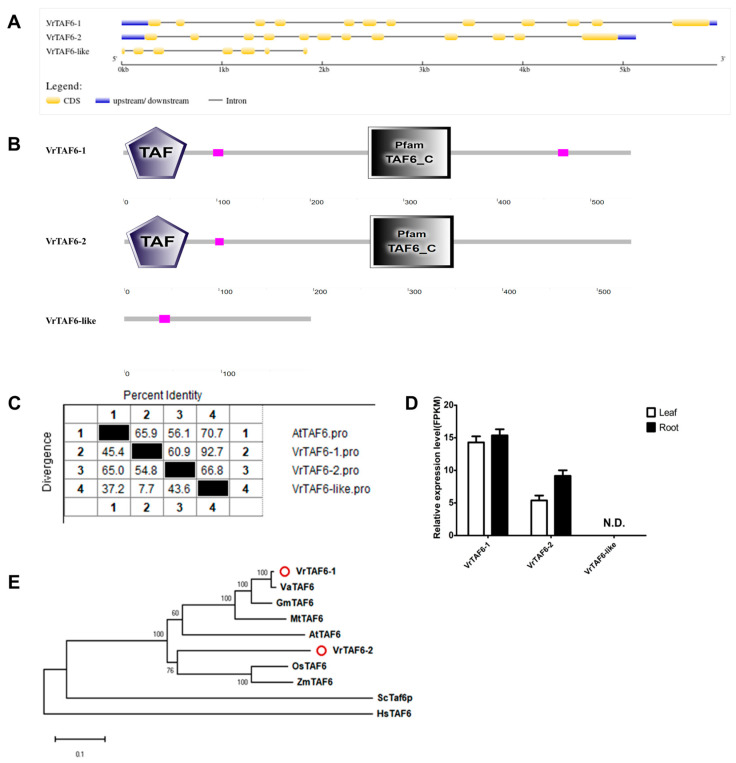
Multi-analysis of *VrTAF6*. (**A**) Gene structures of *VrTAF6–1*, *VrTAF6–2*, and *VrTAF6–like*. (**B**) Domains analyzed of VrTAF6–1, VrTAF6–2, and VrTAF6–like by SMART. (**C**) Sequence identity to AtTAF6. (**D**) Relative expression levels of *VrTAF6–1*, *VrTAF6–2*, and *VrTAF6–like* in the leaves and roots of mungbean seedlings. N.D.: Not detected. (**E**) Phylogenetic tree of TAF6 from multi-species (see Figure 2). The red circles indicate the proteins from mungbean.

Three loci, *LOC106758550*, *LOC106775101*, and *LOC111240733*, are annotated as *VrTAF6*, specifically named *VrTAF6–1*, *VrTAF6–2*, and *VrTAF6*–like, located on chr04 (*Vradi04g10770.1*), chr10 (*Vradi10g02250.1*), and scaffold (NW_014541992.1) (Table 1 and Figure 1). Analysis of the gene structure reveals that both *VrTAF6–1* and *VrTAF6–2* consist of 12 exons and 11 introns, encoding proteins with lengths of 543 aa and 536 aa, respectively. In contrast, *VrTAF6*–like contains only seven exons, encoding a protein of length 192 aa (Figure 7A). Further domain analysis indicates that VrTAF6–like lacks any conserved domains found in TAF6, such as ‘TAF’ and ‘TAF6_C’, which are present in both VrTAF6–1 and VrTAF6–2 (Figure 7B). Additionally, the expression of *VrTAF6*–like cannot be detected at the transcriptional level (Figure 7D), suggesting it may be a pseudogene. Furthermore, VrTAF6–1 and VrTAF6–2 exhibit sequence identities of 65.9% and 56.1%, respectively, to AtTAF6 (549 aa) (Figure 7C and Table S1). In the evolution, VrTAF6–1 is more similar to VaTAF6, while VrTAF6–2 appears to be closer to the TAF6 proteins in monocotyledon (Figure 7E). Based on the results, *VrTAF6–1* and *VrTAF6–2* are being examined further in relation to stress response.

#### 2.2.7. VrTAF7

**Figure 8:**
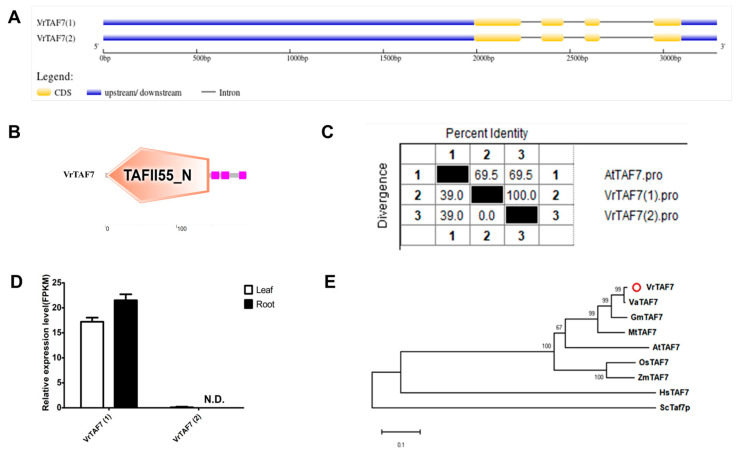
Multi-analysis of *VrTAF7*. (**A**) Gene structures of *VrTAF7(1)* and *VrTAF7(2)*. (**B**) Domains analyzed of VrTAF7 by SMART. (**C**) Sequence identity to AtTAF7. (**D**) Relative expression levels of *VrTAF7(1)* and *VrTAF7(2)* in the leaves and roots of mungbean seedlings. N.D.: Not detected. (**E**) Phylogenetic tree of TAF7 from multi-species (see Figure 2). The red circle indicates the protein from mungbean.

Located on chr06 (*Vradi06g12480.1*), *LOC106763916* is annotated as possessing the TAF7 function, a small protein consisting of only 199 amino acids (Table 1 and Figure 1). Despite the presence of two transcripts, *XM_014648094.2* (1) and *XM_022782426.1* (2), in the NCBI database, they exhibit identical sequences at both the transcriptional and protein levels. However, the transcription of *XM_022782426.1* cannot be detected by RNA-seq (Figure 8D). Therefore, *XM_014648094.2* is deemed to be the authentic transcript for *VrTAF7* and will be utilized for further exploration. *VrTAF7* comprises four exons and three introns, with its encoded protein containing a conserved ‘TAFII55_N’ domain at the N-terminus, characteristic of TAF7 proteins (Figure 8A,B). The protein sequence of VrTAF7 shares a significant identity of 69.5% with AtTAF7 (Figure 8C and Table S1), and its phylogenetic pattern closely resembles that of most TAFs (Figure 8E).

#### 2.2.8. VrTAF8

**Figure 9:**
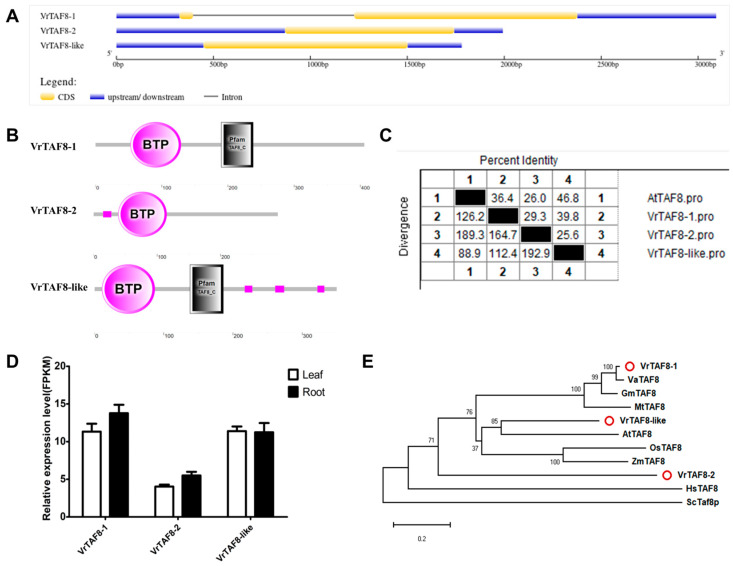
Multi-analysis of *VrTAF8*. (**A**) Gene structures of *VrTAF8–1*, *VrTAF8–2*, and *VrTAF8*–like. (**B**) Domains analyzed of VrTAF8–1, VrTAF8–2, and VrTAF8–like by SMART. (**C**) Sequence identity to AtTAF8. (**D**) Relative expression levels of *VrTAF8–1*, *VrTAF8–2*, and *VrTAF8*–like in the leaves and roots of mungbean seedlings. (**E**) Phylogenetic tree of TAF8 from multi-species (see Figure 2). The red circles indicate the proteins from mungbean.

Three loci distributed on different chromosomes, specifically *LOC106759260* (*Vradi04g09290.1*), *LOC106764373* (*Vradi06g06010.1*), and *LOC106769930* (*Vradi08g07170.1*), are described as TAF8-related proteins, encoding VrTAF8–1, VrTAF8–2, and VrTAF8–like, respectively (Table 1 and Figure 1). *VrTAF8–1* consists of two exons, while both *VrTAF8–2* and *VrTAF8*–like have only one exon. Additionally, there is a significant difference in the length of the encoded proteins (Table 1 and Figure 9A). Notably, VrTAF8–2 is the shortest protein with a length of 290 amino acids and lacks the conserved motif ‘TAF8_C’ at the C-terminal end (Figure 9B). Furthermore, the sequence identity of VrTAF8–2 to AtTAF is only 26.0%, whereas that of VrTAF8–1 and VrTAF8–like is 36.4% and 46.8%, respectively (Figure 9C and Table S1). The transcripts of all three genes can be detected by RNA-seq analysis (Figure 9D). On the phylogenetic tree, it can be observed that VrTAF8-1 clusters with TAF8 proteins from legume crops, while VrTAF8-like is grouped with AtTAF8. In contrast, VrTAF8–2 appears to be distantly related to TAF8 within the plant kingdom (Figure 9E). Hence, *LOC106764373* may be a spurious VrTAF8, and further investigations will be conducted on *VrTAF8–1* and *VrTAF8*–like.

#### 2.2.9. VrTAF9

**Figure 10:**
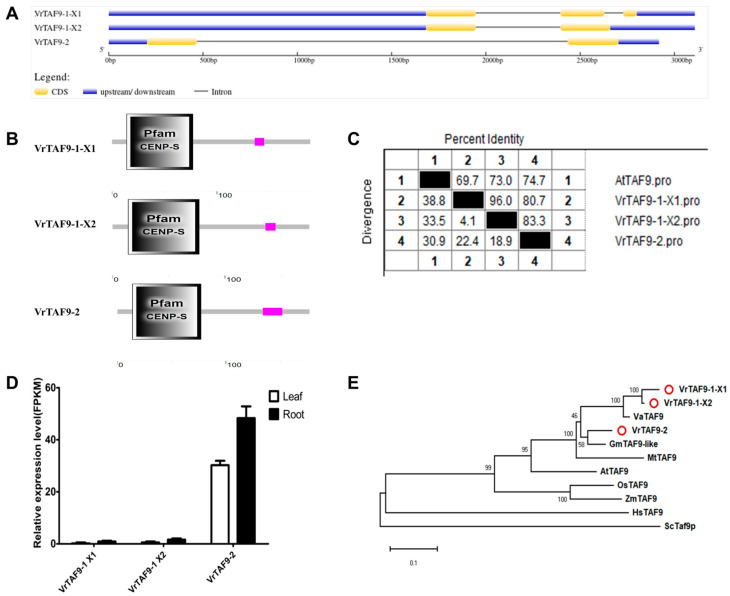
Multi-analysis of *VrTAF9*. (**A**) Gene structures of *VrTAF9–1 X1*, *VrTAF9–1 X2*, and *VrTAF9–2*. (**B**) Domains analyzed of VrTAF9–1 X1, VrTAF9–1 X2, and VrTAF9–2 by SMART. (**C**) Sequence identity to AtTAF9. (**D**) Relative expression levels of *VrTAF9–1 X1*, *VrTAF9–1 X2*, and *VrTAF9–2* in the leaves and roots of mungbean seedlings. (**E**) Phylogenetic tree of TAF9 from multi-species (see Figure 2). The red circles indicate the proteins from mungbean.

*LOC106764413* (*Vradi06g02960.1*) and *LOC106767081* (*Vradi07g24390.1*) are annotated as *VrTAF9*, specifically identified as *VrTAF9–1* and *VrTAF9–2* (Table 1 and Figure 1). *VrTAF9–1* consists of two transcripts, X1 and X2, with three exons and two exons, respectively (Figure 10A). All three proteins contain the ‘Pfam CENP-S’ domain, known for its dual function in DNA repair/recombination and localization to centromeres for promoting chromosome segregation (Figure 10B). The sequence identity of VrTAF9–1 X1, X2, and VrTAF9–2 to AtTAF9 ranges from 69.7% to 74.7% (Figure 10C and Table S1). However, the transcript levels of *VrTAF9–1 X1* and *VrTAF9–1 X2* are almost undetectable (Figure 10D). In terms of evolution, VrTAF9–1 X1 and X2 are closely related to VaTAFT, while VrTAF9–2 is more closely associated with GmTAF9–like (Figure 10E). Consequently, further investigation into the expression response under the abiotic stress of *VrTAF9–2* is warranted.

#### 2.2.10. VrTAF10

**Figure 11:**
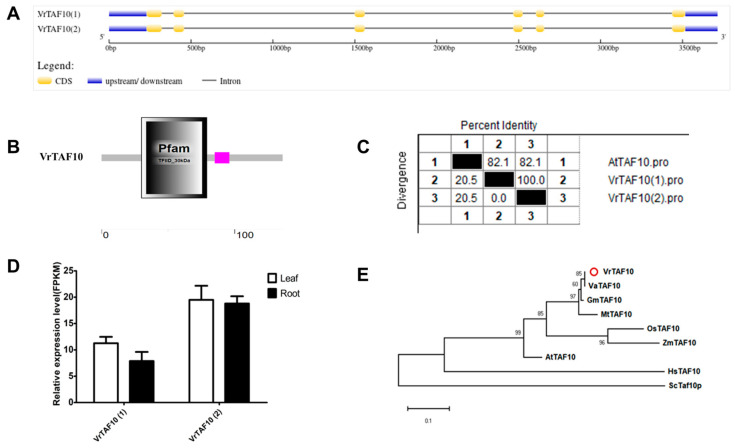
Multi-analysis of *VrTAF10*. (**A**) Gene structure of *VrTAF10*. (**B**) Domain analyzed of VrTAF10 by SMART. (**C**) Sequence identity to AtTAF10. (**D**) Relative expression level of *VrTAF10* in the leaves and roots of mungbean seedlings. (**E**) Phylogenetic tree of TAF10 from multi-species (see Figure 2). The red circle indicates the protein from mungbean.

The locus on chr04 (*Vradi04g11480.1*) *LOC106758746* encodes the VrTAF10 protein in the mungbean genome. Two transcripts, *XM_014641724.2* (1) and *XM_014641723.2* (2)*,* are listed on NCBI with identical sequences at both the mRNA and protein levels (Table 1 and Figure 1). Despite consisting of six exons, *VrTAF10* encodes the smallest subunit of TFIID (136 aa, same as *VrTAF13*) (Figure 11A and Table 1). The ‘Pfam TFIID_30kDa’ domain is present in the VrTAF10 protein, which is also a component of other transcription regulatory multiprotein complexes, such as SAGA, TFTC, STAGA, and PCAF/GCN5 (Figure 11B). VrTAF10 shares a high sequence identity with AtTAF10 (82.1%) (Figure 11C and Table S1). The RNA-seq data show the detection of both *VrTAF10(1)* and *VrTAF10(2)*, with the latter exhibiting approximately double the expression level of the former (Figure 11D). Notably, VrTAF10 can be grouped with TAF10 proteins from legumes, particularly closer to monocotyledon rather than Arabidopsis (Figure 11E). Consequently, further analysis under stresses will focus on the expression of *VrTAF10(2)*.

#### 2.2.11. VrTAF11

**Figure 12:**
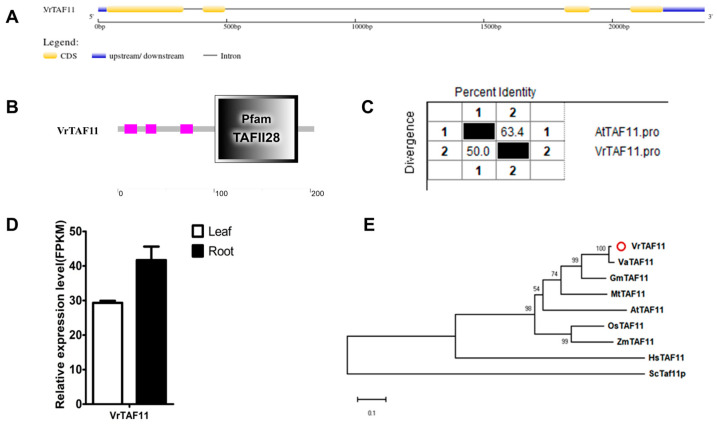
Multi-analysis of *VrTAF11*. (**A**) Gene structure of *VrTAF11*. (**B**) Domain analyzed of VrTAF11 by SMART. (**C**) Sequence identity to AtTAF11. (**D**) Relative expression level of *VrTAF11* in the leaves and roots of mungbean seedlings. (**E**) Phylogenetic tree of TAF11 from multi-species (see Figure 2). The red circle indicates the protein from mungbean.

The *VrTAF11* gene is located on chr09 (*Vradi09g05150.1*) and consists of a single locus (*LOC106773625*) and transcript (*XM_014660347.2*) (Table 1 and Figure 1). It contains four exons, encoding a 204 amino acid protein, which is a relatively small subunit of TFIID (Figure 12A and Table 1). The C terminal of the VrTAF11 protein contains a conserved ‘Pfam TAFII28’ motif with four alpha helices and three loops arranged similarly to histone H3 (Figure 12B). The sequence identity between VrTAF11 and AtTAF11 is approximately 63.4% (Figure 12C and Table S1). A high expression of *VrTAF11* can be detected in both leaves and roots (Figure 12D). In terms of evolution, VrTAF11 is most closely related to VaTAF11 among the TAF11 proteins (Figure 12E). 

#### 2.2.12. VrTAF12

**Figure 13:**
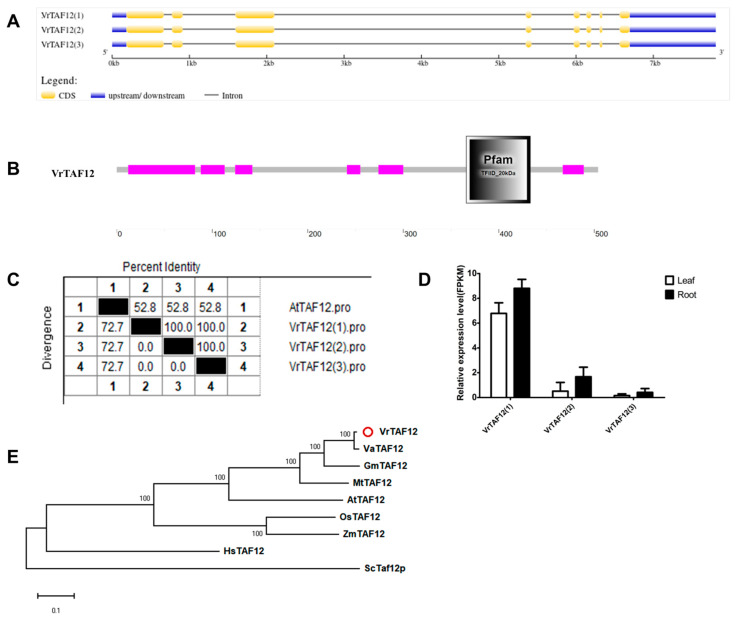
Multi-analysis of *VrTAF12*. (**A**) Gene structure of *VrTAF12*. (**B**) Domain analyzed of VrTAF12 by SMART. (**C**) Sequence identity to AtTAF12. (**D**) Relative expression level of *VrTAF12* in the leaves and roots of mungbean seedlings. (**E**) Phylogenetic tree of TAF12 from multi-species (see Figure 2). The red circle indicates the protein from mungbean.

*LOC106772695*, located on chr08 (*Vradi08g05550.1*) in the NCBI database, encodes the VrTAF12 protein with three transcripts: *XM_014659247.2 (1)*, *XM_014659248.2 (2)*, and *XM_022784568.1 (3)* (Table 1 and Figure 1). Further analysis reveals that all three transcripts encode the same protein sequence with an identical CDS sequence (Figure 13A,C). *VrTAF12* consists of eight exons and encodes a protein with 504 amino acids. A conserved motif, ‘Pfam TFIID_20 kDa’, is present at the C terminal of VrTAF12, showing a 52.8% sequence identity to AtTAF12 (Figure 13B,C and Table S1). However, *VrTAF12 (2)* and *VrTAF12 (3)* exhibit a low expression at the transcriptional level (Figure 13D). The evolutionary pattern of VrTAF12 is similar to most VrTAFs, with VaTAF12 being its closest relative (Figure 13E). Therefore, further investigations will focus on *VrTAF12 (1)*.

#### 2.2.13. VrTAF12b

**Figure 14:**
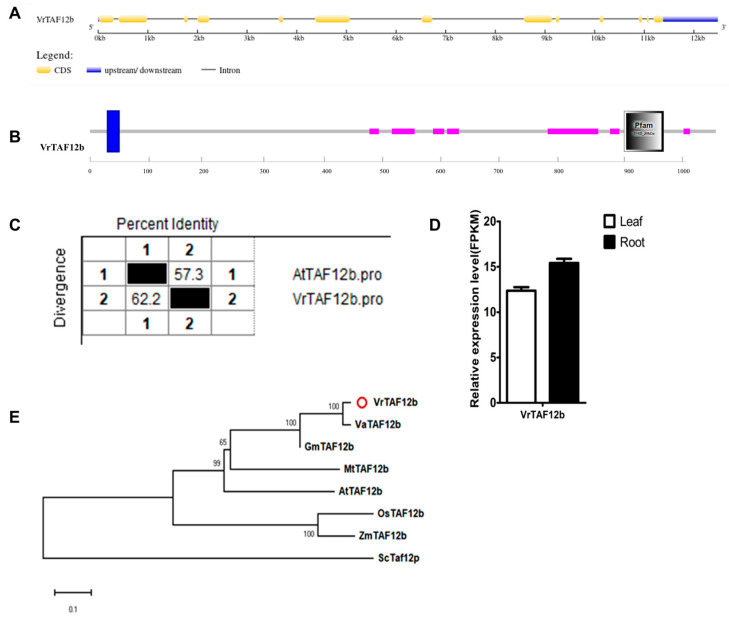
Multi-analysis of *VrTAF12b*. (**A**) Gene structure of *VrTAF12b*. (**B**) Domain analyzed of VrTAF12b by SMART. (**C**) Sequence identity to AtTAF12b. (**D**) Relative expression level of *VrTAF12b* in the leaves and roots of mungbean seedlings. (**E**) Phylogenetic tree of TAF12b from multi-species (see Figure 2). The red circle indicates the protein from mungbean.

A previous study on *Arabidopsis* demonstrated that TAF12 and TAF12b have differential affinities toward TFIID and SAGA components [49]. Therefore, we did not include VrTAF12b in the analysis with VrTAF12, as these two protein sequences share only 32.7% identity. *LOC106774141*, located on chr09 (*Vradi09g02430.1*), encodes VrTAF12b and has a single transcript, *XM_014661010.2* (Table 1 and Figure 1). In the genome, *VrTAF12b* consists of 13 exons and the coding protein is approximately twofold larger than that of *VrTAF12* (Figure 14A). Apart from the C-terminal conserved domain, ‘Pfam TFIID_20 kDa’, there is a transmembrane helix region at the N-terminal (Figure 14B). VrTAF12b shares a 57.3% sequence identity with AtTAF12b, and its expression level is high in both the leaves and roots (Figure 14C, Table S1, and Figure 14D). In terms of evolution, no homologous protein of TAF12b has been identified in humans. The evolutionary model of VrTAF12b is similar to that of VrTAF12 as well as most other VrTAFs (Figure 14E). However, further research should be undertaken to explore the specific roles of VrTAF12b and VrTAF12.

#### 2.2.14. VrTAF13

**Figure 15:**
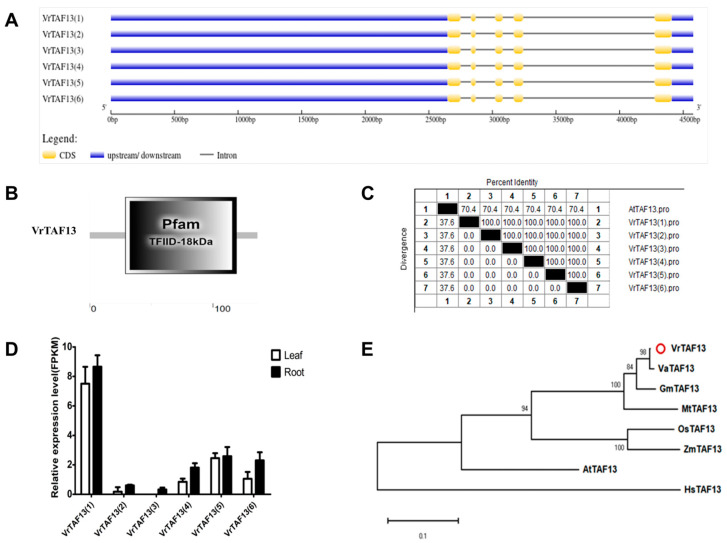
Multi-analysis of *VrTAF13*. (**A**) Gene structure of *VrTAF13* (**B**) Domain analyzed of VrTAF13 by SMART. (**C**) Sequence identity to AtTAF13. (**D**) Relative expression level of *VrTAF13* in the leaves and roots of mungbean seedlings. (**E**) Phylogenetic tree of TAF13 from multi-species (see Figure 2). The red circle indicates the protein from mungbean.

Only one locus, *LOC106770840*, on chr08 (*Vradi08g10630.1*) encodes the VrTAF13 protein, with up to six transcripts (Table 1 and Figure 1). Similar to the case of *VrTAF12*, these six transcripts of *VrTAF13* are identical, containing five exons and producing a protein with 136 aa (Figure 15A,C). VrTAF13 is one of the smallest units in TFIID, featuring a conserved motif, ‘Pfam TFIID_15KDa’ (Figure 15B). Despite showing a high sequence identity of 70.4% to AtTAF13 (Figure 15C and Table S1), the evolutionary distance places it much closer to monocotyledon species (except legume), such as rice and maize, rather than AtTAF13 (Figure 15E). Among the six transcripts, VrTAF13 (1) exhibits the highest expression level in both the leaves and roots (Figure 15D), thus making it suitable for further analysis.

#### 2.2.15. VrTAF14b

**Figure 16:**
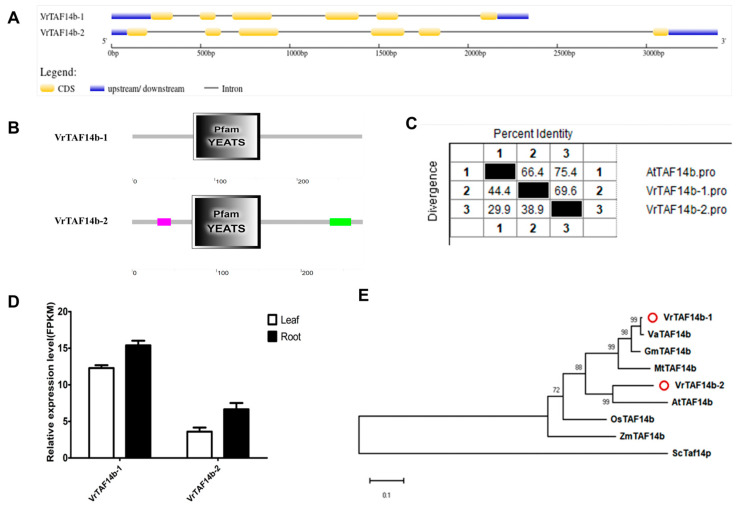
Multi-analysis of *VrTAF14b*. (**A**) Gene structure of *VrTAF14b–1* and *VrTAF14b–2.* (**B**) Domain analyzed of VrTAF14b–1 and VrTAF14b–2 by SMART. (**C**) Sequence identity to AtTAF14b. (**D**) Relative expression level of *VrTAF14b–1* and *VrTAF14b–2* in the leaves and roots of mungbean seedlings. (**E**) Phylogenetic tree of TAF14b from multi-species (see Figure 2). The red circles indicate the proteins from mungbean.

Two genes, *LOC106777003* (*Vradi11g06360.1*) and *LOC106780520* (NW_014542625.1), are annotated as *VrTAF14b* (Table 1 and Figure 1). Both *VrTAF14b–1* and *VrTAF14b–2* consist of six exons, encoding proteins with lengths of 279 aa and 273 aa, respectively (Table 1 and Figure 16A). Both proteins contain the conserved domain ‘Pfam YEATS’ (YNK7, ENL, AF–9, and TFIIF small subunit), which exhibits transcription stimulatory activity. Additionally, VrTAF14b–2 features a ‘low complexity region’ at the N terminal and a ‘coiled coil region’ at the C terminal (Figure 16B). Furthermore, both VrTAF14b–1 and VrTAF14b–2 display a high identity (66.4% and 75.4%) to AtTAF14b (Figure 16C and Table S1), with detectable expression levels for both variants (Figure 16D). In terms of the evolutionary relationships, VrTAF14b–1 is closely associated with VaTAF14b, while VrTAF14b–2 forms a cluster with AtTAF14b (Figure 16E).

#### 2.2.16. VrTAF15

**Figure 17:**
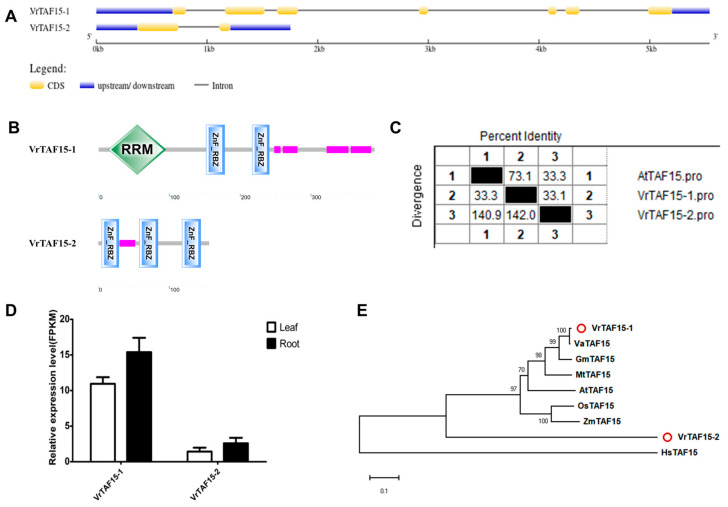
Multi-analysis of *VrTAF15*. (**A**) Gene structures of *VrTAF15–1* and *VrTAF15–2.* (**B**) Domains analyzed of VrTAF15–1 and VrTAF15–2 by SMART. (**C**) Sequence identity to AtTAF15. (**D**) Relative expression levels of *VrTAF15–1* and *VrTAF15–2* in the leaves and roots of mungbean seedlings. (**E**) Phylogenetic tree of TAF15 from multi-species (see Figure 2). The red circles indicate the proteins from mungbean.

*LOC106777580* and *LOC106760812* are identified as VrTAF15 coding genes, located on chr02 (*Vradi02g02560.1*) and chr05 (*Vradi05g09940.1*), respectively, designated as *VrTAF15–1* and *VrTAF15–2* (Table 1 and Figure 1). However, all analyses indicate that *VrTAF15–2* appears to be a pseudogene. It consists of only two exons and encodes a protein with a length of 156 aa, while the protein encoded by VrTAF15–1 is 390 aa long with seven exons (Table 1 and Figure 17A). Protein domain analysis reveals that VrTAF15–2 contains three ‘ZnF_RBZ ‘motifs but lacks the N-terminal’ RRM domains involved in RNA recognition (Figure 17B). The sequence identity of VrTAF15–1 to AtTAF15 is high at 73.1%, whereas that of VrTAF15–2 is only at 33.3% (Figure 17C and Table S1). The relative expression level of *VrTAF15–2* is significantly lower compared to that of *VrTAF15–1* (Figure 17D). Furthermore, VrTAF15–2 does not exhibit clustering with other plant TAF15 proteins on the phylogenetic tree, in contrast to VrTAF15–1, which is grouped with TAF15 from legume crops (Figure 17E). As a result, the further investigation of *VrTAF15–2* is not warranted.

#### 2.2.17. VrTAF15b

**Figure 18:**
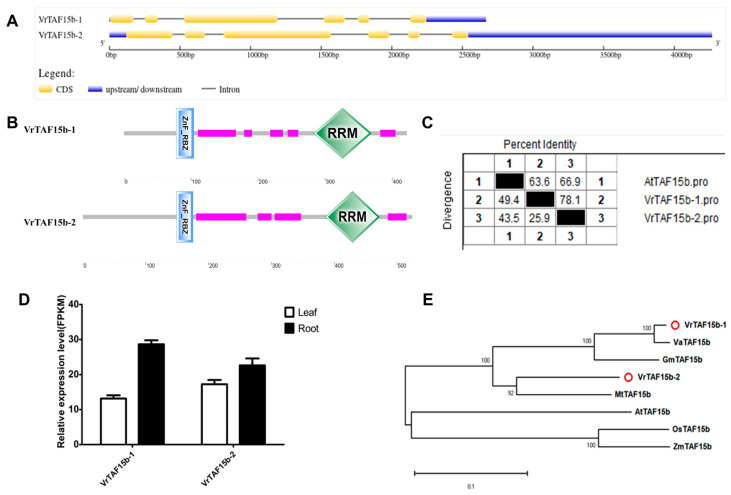
Multi-analysis of *VrTAF15b*. (**A**) Gene structures of *VrTAF15b–1* and *VrTAF15b–2.* (**B**) Domains analyzed of VrTAF15b–1 and VrTAF15b–2 by SMART. (**C**) Sequence identity to AtTAF15b. (**D**) Relative expression levels of *VrTAF15b–1* and *VrTAF15b–2* in the leaves and roots of mungbean seedlings. (**E**) Phylogenetic tree of TAF15b from multi-species (see Figure 2). The red circles indicate the proteins from mungbean.

Two loci, *LOC106752587* and *LOC106752498*, annotated as *VrTAF15b* (Table 1 and Figure 1), namely *VrTAF15b–1* and *VrTAF15b–2*, respectively, both consisted of six exons. However, the length of the VrTAF15b–2 protein is significantly longer than that of VrTAF15b–1, with lengths of 524 aa and 422 aa, respectively (Table 1 and Figure 18A). Despite this difference in length, both variants share similar conserved protein motifs, including the N-terminal ‘ZnF_RBZ’ motif and C-terminal ‘RRM’ domain (Figure 18B). Furthermore, they exhibit a high sequence identity to AtTAF15b at approximately 63.6% and 66.9% for VrTAF15b–1 and VrTAF15b–2, respectively (Figure 18C and Table S1). The expression levels of both *VrTAF15b* variants are approximately equivalent (Figure 18D). Phylogenetic analysis reveals that VrTAf15b–1 is closely related to VaTAF15b, while VrTAF15b–2 is grouped with MtTAF15b–2. Interestingly, predictions for their subcellular locations suggest distinct distributions for the two variants (Table 1), indicating potential diverse roles in transcriptional regulation.

### 2.3. VrTBP and VrTAFs Expression in Response to Abiotic Stress Treatments

Based on the aforementioned analysis, 1 *VrTBP* and 21 *VrTAFs* were further investigated for their potential involvement in responding to abiotic stress. Four genes, including *VrTAF6*–like, *VrTAF8–2*, *VrTAF9–1*, and *VrTAF15–2*, have been identified as pseudogenes due to the absence of certain domains or undetectable expression. For the analysis of stress response, 12-day-old mungbean seedlings were subjected to treatments, including NaCl (200 mM), water deficit (15% PEG6000), heat (35 °C), and cold (4 °C). Different stress conditions resulted in varied patterns of expression for *VrTBP* and *VrTAFs*.

#### 2.3.1. High-Salinity Stress

**Figure 19:**
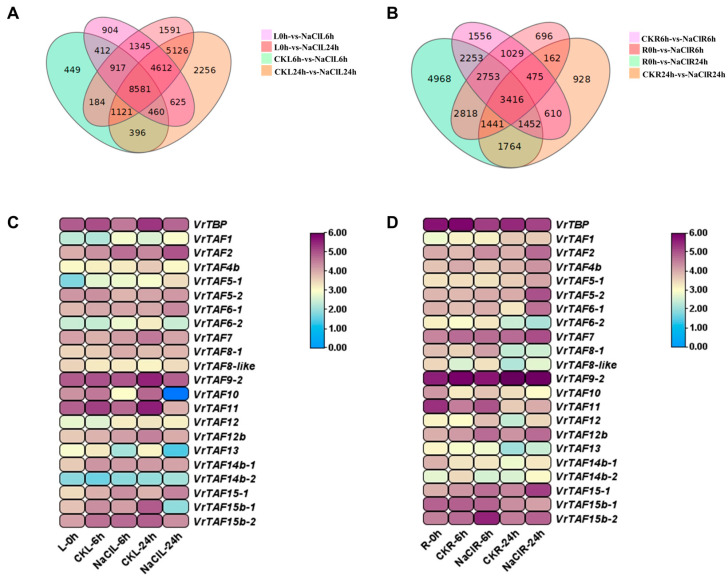
The expressions of *VrTBP* and *VrTAFs* respond to high-salinity stress. (**A**) Venn diagram representing all relationships between the four groups of genes in leaves responding high-salinity stress: L0h-vs-NaClL6h (light purple), L0h-vs-NaClL24h (pink), CKL6h-vs-NaClL6h (green), and CKL24h-vs-NaClL24h (orange). (**B**) Venn diagram representing all relationships between the four groups of genes in roots responding high-salinity stress: R0h-vs-NaClR6h (pink), R0h-vs-NaClR24h (green), CKR6h-vs-NaClR6h (light purple), and CKR24h-vs-NaClR24h (orange). (**C**) The heat map of *VrTBP* and *VrTAFs* in leaves under salt stress. (**D**) The heat map of *VrTBP* and *VrTAFs* in roots under salt stress. Values (RNA-seq data), in fragments per kilobase of transcript per million reads mapped (FPKM), are used as the logarithm (LOG 2) for the heat map. L: leaf; R: root.

We observed the expression responses of *VrTBP* and *VrTAFs* in leaves and roots at 6 h and 24 h following treatment with 200 mM of NaCl. The Venn diagram revealed significant variations in the number of differentially expressed genes among different treatment groups in both the leaves and roots (Figure 19A,B). There were 449 and 2256 unique genes in CKL6h-vs-NaClL6h (leaves treated with liquid MS for 6 h vs. with 200 mM of NaCl for 6 h) and CKL24h-vs-NaClL24h, respectively, while the corresponding numbers in roots were 1556 and 928 (Figure 19A,B). Specifically, the transcript level of *VrTBP* decreased in both the leaves and roots after soaking in an NaCl solution for both time points (Figure 19C,D and Figure S1). *VrTAF2* and *VrTAF15–1* were both induced by NaCl in the leaves and roots at 6 h and 24 h. *VrTAF5–2* (leaf), *VrTAF6–2* (root), *VrTAF8–1*, *VrTAF11* (root), *VrTAF12* (leaf), and *VrTAF14b–2* (root) appeared to function only after 6 h of NaCl exposure. Additionally, several genes showed a relatively slow response, being reduced or inhibited only after 24 h of treatment, such as *VrTAF5–1*, *VrTAF6–1*, *VrTAF7*, *VrTAF9*, *VrTAF14b–1*, and *VrTAF15b–1*. Notably, in leaves, the expression of *VrTAF10*, *VrTAF11*, and *VrTAF13* was sharply downregulated at both 6 h and 24 h time points; similarly, leaf *VTFA15b–1* was also significantly downregulated at the latter time point. This suggests that these genes may play a dominant role in responding to salt stress in leaves. Conversely, in the roots, *VrTAF12*, *VrTAF12b*, and *VrTAF15b–2* were significantly upregulated at both time points, while *VrTAF5–2* and *VrTAF6–1* were strongly reduced at 24 h.

#### 2.3.2. Water-Deficit Stress

**Figure 20:**
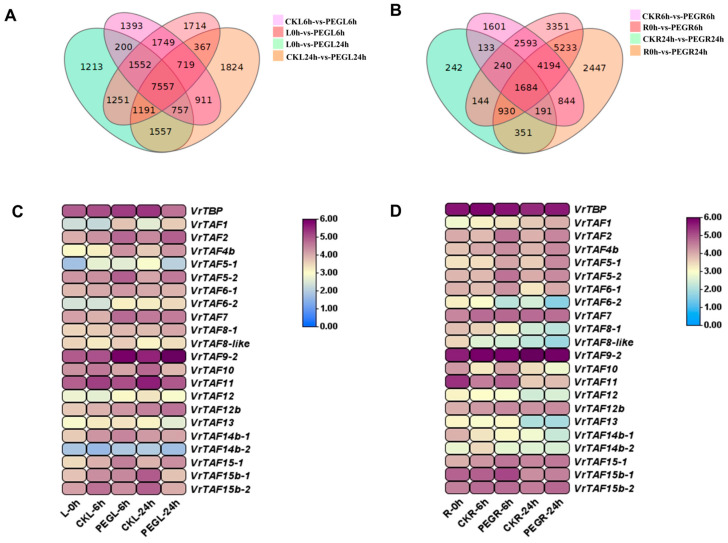
The expression of *VrTBP* and *VrTAFs* responding to water-deficit stress. (**A**) Venn diagram representing all relationships between the four groups of genes in leaves responding to water-deficit stress: L0h-vs-PEGL6h (pink), L0h-vs-PEGL24h (green), CKL6h-vs-PEGL6h (light purple), and CKL24h-vs-PEGL24h (orange). (**B**) Venn diagram representing all relationships between the four groups of genes in roots responding to water-deficit stress: R0h-vs-PEGR6h (pink), R0h-vs-PEGR24h (orange), CKR6h-vs-PEGR6h (light purple), and CKR24h-vs-PEGR24h (green). (**C**) The heat map of *VrTBP* and *VrTAFs* in leaves under water-deficit stress. (**D**) The heat map of *VrTBP* and *VrTAFs* in roots under water-deficit stress. Values (RNA-seq data) FPKM are used as the logarithm (LOG 2) for the heat map. L: leaf; R: root.

For water-deficit stress, 12-day-old mungbean seedlings were subjected to 15% (*w*/*v*) PEG6000 for 6 h and 24 h. The Venn diagram revealed that, in leaf tissue, there were 1393 and 1824 genes unique to CKL6h-vs-PEGL6h and CKL24h-vs-PEGL24h, respectively. In contrast, the corresponding numbers in roots were 1601 and 242 genes (Figure 20A,B). The expression of several genes, including *VrTAF1*, *VrTAF2*, *VrTAF5–2*, *VrTAF8-1*, *VrTAF9*, *VrTAF12b*, and *VrTAF15–1*, was significantly induced after both the 6 h and 24 h treatments in leaves. However, *VrTBP*, *VrTAF10*, *VrTAF11*, *VrTAF15b–1*, and *VrTAF15b–2* showed a downregulation, especially at the later time point. In the roots, *VrTAF2*, *VrTAF5–1*, and *VrTAF6–1* exhibited consistent induction under water-deficit stress conditions, suggesting a potential role in response to this stressor. Conversely, the expression of *VrTBP*, *VrTAF6–2*, *VrTAF8–1*, *VrTAF14b–1*, and *VrTAF14b–2* was inhibited. Notably, only *VrTAF2* was upregulated in both the leaves and roots following treatment at both time points (Figure 20C,D and Figure S2).

#### 2.3.3. Heat and Cold Stress

**Figure 21:**
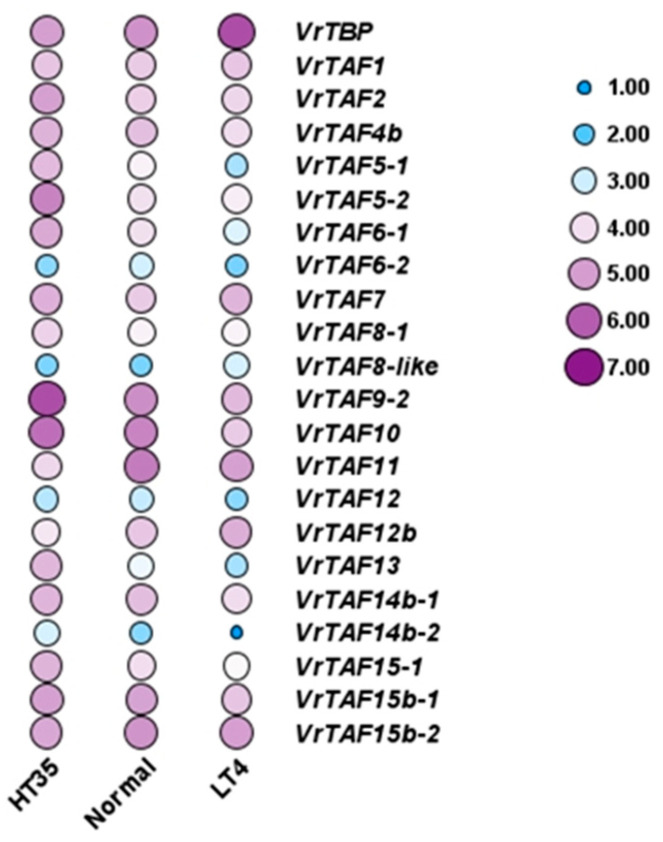
The expression of *VrTBP* and *VrTAFs* responding to heat and cold stress. Values (RNA-seq data). FPKM was used as the logarithm (LOG 2) for the heat map. HT35: mungbean seedlings grown under 35 °C (heat stress); normal: mungbean seedlings grown under 25 °C; LT4: mungbean seedlings grown under 4 °C (cold stress).

Mungbean seedlings were subjected to 35 °C and 4 °C for 24 h to investigate the impact of temperature on the expression of *VrTBP* and *VrTAFs.* Subsequently, gene expression levels in leaves were analyzed. The results depicted in Figure 21 and Figure S3 reveal that *VrTBP* is significantly induced under cold conditions. Furthermore, several genes, including *VrTAF5–1*, *VrTAF6–1*, *VrTAF9–2*, *VrTAF10*, *VrTAF13*, *VrTAF14b–2*, and *VrTAF15–1*, exhibited an upregulation in response to heat stress, but a downregulation under cold stress. Notably, only the high temperature appeared to influence the expression of *VrTAF2* and *VrTAF5–2*, while both heat and cold led to the downregulation of *VrTAF6–2* and *VrTAF11*. These diverse response patterns indicate distinct regulatory mechanisms for these genes in adapting to environmental fluctuations.

## 3. Discussion

TBPs and TAFs are central components of the general transcription factor TFIID, with highly conserved sequences from yeast to humans [50]. Therefore, utilizing bioinformatics tools, such as BLAST and Gcorn, to identify potential homologous proteins is reliable. In our initial screening of the mungbean genome (Table 1), we identified 1 TBP and 25 putative TAFs. Previous research has demonstrated that mammals possess three members in the TBP family, namely TBP, TBP-like protein1 (TBPL1), and TBP-like protein2 (TBPL2 or TBP2) [1,16]. In contrast, *Arabidopsis*, rice, and maize each have two copies of the TBPs [28,41,51], while the mungbean genome appears to only contain one copy of the TBP. This pattern is also observed in other legume crops, such as *Vigna angularis*, *Glycine max*, and *Medicago truncatula.* This phenomenon may be attributed to gene loss during evolution, or potentially supplemented by additional protein molecules to intricately and precisely regulate transcription. As for the VrTAF sequences, they exhibit some complexity: specifically, six types of TAFs (VrTAF5, VrTAF6, VrTAF8, VrTAF9, VrTAF14, and VrTAF15) have two to three encoding genes; five TAFs (VrTAF1, VrTAF2, VrTA4b, VrTAF5–1, and VrTAF9–1) have multiple transcripts; and four TAFs (VrTAF7, VrTAF10, VrTAF12, and VrTAF13) appear to have multiple transcripts, but with identical protein sequences. Then, we perform further biological analyses on all possible sequences, including gene structure, conserved domains, sequence identity to *Arabidopsis* homologous proteins and phylogenetic trees, as well as their expression level in the leaves and roots. The results indicate that *VrTAF6*–like, *VrTAF8–2, VrTAF9–1*, and *VrTAF15–2* are likely to be pseudogenes, while the remaining 21 TAFs were analyzed under abiotic stress. Generally, the typical number of TAF subunits comprising TFIID is 13–14 [13,52]. In *Arabidopsis*, 18 putative AtTAF proteins were identified [27]. It is noteworthy that there is no homologous TAF3 in the mungbean genome, and TAF3 is also absent from the genomes of *Arabidopsis* and rice [27]. Therefore, it is presumed that TAF3 is lacking in plants, but other TAF subunits may compensate for its function. Moreover, TAF4 and TAF14 homologous proteins also cannot be identified in the mungbean genome, but one VrTAF4b with multiple transcripts and two copies of VrTAF14b are isolated. Nevertheless, further biochemical and genetic studies are necessary to verify the real function of the TAF proteins in forming the TFIID complex and regulating transcription. 

The mungbean genome contains up to six TAFs with two or three copies, potentially enhancing its plasticity to adapt to the complexity of transcriptional regulation during development and stress. In *Arabidopsis*, there are seven TAFs with two copies, namely AtTAF1, AtTAF4, AtTAF6, AtTAF11, AtTAF12, AtTAF14, and AtTAF15 [27]. Further studies indicated that these different copies might function during diverse processes. Two *Arabidopsis TAF1*-related genes are known as *TAF1* (*At1g32750*) and *TAF1b* (*At3g19040*), both of which possess histone acetyltransferase activity [27]. A null mutation in *taf1* is lethal, while *taf1b* lines are viable and fertile. Further investigations revealed that *AtTAF1* is essential for resistance to genotoxic stress and pollen tube development through its interaction with MRE11, a core component involved in DNA double-strand break detection and repair [53]. Meanwhile, *AtTAF1b* functions as a coactivator capable of integrating light signals and activating light-regulated genes through histone acetylation [54,55]. Two paralogs of *AtTAF4*, *TAF4* (*At5g43130*) and *TAF4b* (*At1g27720*), exhibit distinct expression patterns: *TAF4* shows a broader constitutive expression, while *TAF4b* is enriched during meiosis and controls meiotic crossover events and germline transcription [56]. Two AtTAF6-related proteins (TAF6 encoded by *At1g04950* and TAF6b encoded by *At1g54360*) also have been identified with non-redundant functions in *Arabidopsis.* Specifically, AtTAF6 regulates pollen tube growth, and loss-of-function mutants result in a lethal phenotype [57]. The occurrence of gene duplication events is frequently attributed to the replication of genes during the process of evolution. Whether the multiple copies of VrTAFs have redundant or distinct functions during developmental process or stress response needs further investigation.

The pivotal regulatory role of TBPs and TAFs in plant development and stress response has garnered the attention of researchers. However, current studies predominantly focus on model plants (such as *Arabidopsis* and rice) [7,39], with limited reports available for other crops. In terms of abiotic stress response, previous studies have demonstrated the involvement of yeast ScTBP in hyperosmotic stress [26], and highlighted the important role of rice OsTBP2.2 during drought stress [41]. In the protozoan parasite *Entamoeba histolytica*, the expression level of *EhTAF1* was upregulated under heat shock stress [58]. Additionally, TAF6 identified from finger millet (*Eleusine coracana* (L.) Gaertn), a drought-adapted crop, played a crucial role in safeguarding the transcription process under drought stress [59]. In *Arabidopsis*, *AtTAF10* has been demonstrated to be involved in the adaptation of plants to osmotic stress [30]. Strikingly, TAF5, TAF6, TAF9, TAF10, and TAF12 subunits are also important components of the SAGA complex [13,60]. Studies have shown that SAGA complexes in yeast, humans, and plants are involved in the regulation of stress-responsive gene transcription [12,15,61]. Interestingly, in yeast, genes regulated by SAGA are predominantly induced by stress and do not rely on TAF subunits [12]. SAGA is capable of facilitating TBP binding to the TATA-box and initiating transcription, as well as modulating gene expression in a manner dependent on other modules, such as the HAT module with the acetylation function and the DUB module with ubiquitination activity [15,18,62]. 

In this study, 1 TBP and 21 putative TAFs are systematically identified from the mungbean genome, including their gene structure, conserved domains, expression level, and phylogenetic tree analysis. Furthermore, the expressions of *VrTBP* and *VrTAFs* responding to stress (salt, water deficit, heat, and cold) were investigated, displaying diverse patterns. Specifically, *VrTBP*, *VrTAF2*, and *VrTAF15–1* were identified as positive regulators of salt stress, while *VrTAF10*, *VrTAF11*, and *VrTAF13* acted as negative regulators, primarily in the leaves. In the case of water-deficit stress, it is suggested that *VrTAF1*, *VrTAF2*, *VrTAF5–2*, *VrTAF9*, and *VrTAF15–1* may play a predominant role. Regarding the changes in ambient temperature response, *VrTAF5–1*, *VrTAF6–1*, *VrTAF9–2*, *VrTAF10*, *VrTAF13*, *VrTAF14b–2*, and *VrTAF15–1* may be key players. Thus, basal regulators like TBPs and TAFs are potential candidates linked to stress adaptation in plants.

## 4. Materials and Methods

### 4.1. Identification, Structure, and Motif Analyses of VrTBP and VrTAFs 

The National Center of Biotechnology Information (NCBI) online tool BLASTP (https://blast.ncbi.nlm.nih.gov/Blast.cgi) (accessed on 28 to 30 December 2023) combined with Gcorn plant [63], a database of plant gene phylogeny (http://www.plant.osakafu-u.ac.jp/~kagiana/gcorn/p/19/) (accessed on 28 to 30 December 2023) and mungbean VC1973A (2n = 2x = 22) genome data released in 2014 [64] (Crop Genomic Lab, http://plantgenomics.snu.ac.kr/mediawiki-1.21.3/index.php/Main_Page) (accessed on 17 January 2024), were used to identify the information of *VrTBP* and *VrTAF* genes and related sequences. Visualization of the distribution of genes on the chromosomes was achieved by TBtools [65], and the figure was redrawn to equal scale using Power Point. The online tool GSDS (http://gsds.gao-lab.org/) (accessed on 25 January 2024) was used to determine gene structure. ExPASY (https://www.expasy.org/) (accessed on 17 January 2024) was applied to predict the theoretical pI (isoelectric point) and Mw (molecular weight). The prediction of subcellular localization was conducted by BaCelLo (https://busca.biocomp.unibo.it/bacello/) (accessed on 17 January 2024). Protein domains were analyzed using SMART (http://smart.embl-heidelberg.de/smart/set_mode.cgi?NORMAL=1) (accessed on 18 January 2024). Sequence identity to *Arabidopsis* homologous proteins was calculated using MegAlign software (DNASTAR, https://www.dnastar.com/software/lasergene/megalign-pro/) (accessed on 17 January 2024).

### 4.2. Phylogenetic Analysis of TBP and TAF Genes from Multi-Species

The amino acid sequences of TBPs and TAFs from *Arabidopsis* were downloaded from The Arabidopsis Information Resource (TAIR, https://www.Arabidopsis.org/) (accessed on 1 February 2024). The amino acid sequences of TBPs and TAFs from *Oryza sativa* were downloaded from the China Rice Data Center (https://www.ricedata.cn/) (accessed on 5 February 2024). Sequences from other species, including *Glycine max*, *Medicago truncatula*, *Zea mays*, *Vigna angularis*, *Homo sapiens*, and *Saccharomyces cerevisiae*, were obtained from NCBI (https://www.ncbi.nlm.nih.gov/genome) (accessed on 4 June 2024). MEGAX software (https://www.megasoftware.net/dload_win_gui) (accessed on 17 January 2024) was used to construct the phylogenetic tree with the neighbor-joining (NJ) method with 1000 bootstrap replicates.

All the sequence data can be found in the ‘Supplementary Data’.

### 4.3. Plant Materials

Mungbean cultivar ‘Sulyu 1’ was selected as the tested material. The seedlings were grown in an illumination incubator at 28 °C/26 °C with a 16 h light/8 h dark photoperiod. The seedlings were cultivated in a 1:1 mixture of peat soil (0–20 mm, PINDSTRUP SUBSTRATE, Pindstrup, Denmark) and vermiculite. The 12-day-old healthy seedlings were selected for further treatment after germination.

### 4.4. High-Salinity Treatment

To simulate high-salinity stress, 12-day-old mungbean seedlings were soaked in liquid MS medium with 200 mM of NaCl for 6 h and 24 h. The seedlings treated with liquid MS medium served as the control. Then, we sampled the treated roots and leaves separately after flushing with ddH_2_O (3 times) and immediately froze them in liquid nitrogen. Treatment was repeated three times.

### 4.5. Water-Deficit Treatment

For water-deficit stress, 12-day-old mungbean seedlings were soaked in liquid MS medium with 15% (*w*/*v*) PEG6000 (polyethylene glycol) for 6 h and 24 h. The seedlings treated with liquid MS medium served as the control. Then, we sampled the treated roots and leaves separately after flushing with ddH_2_O (3 times) and immediately froze them in liquid nitrogen. Treatment was repeated three times.

### 4.6. Heat and Cold Treatment

For heat and cold treatments, 12-day-old mungbean seedlings were placed in the incubator at 4 °C (cold stress) and 35 °C (heat stress) for 24 h, respectively. The seedlings grown under 25 °C served as the control. The treated leaves were sampled and immediately frozen in liquid nitrogen. Treatment was repeated three times.

### 4.7. RNA-Seq, Data Statistical Analysis, and Visualization

Total RNA was extracted using a polysaccharide polyphenol plant total RNA extraction kit (PD Biotech, Shanghai, China), and then we conducted RNA-seq using the DNBSEQ platform (BGI, Shenzhen, China). Each sample produced an average of 6.34 Gb of data. We performed an analysis of differential gene enrichment within and between groups, and Venn maps were drawn. Then, the expression level of the genes of interest were picked out and statistically analyzed using Excel (Office 2017) and GraphPad Prism 5 software (https://www.graphpad.com/features) (accessed on 17 January 2024). TBtools [65] was used to draw heat maps after the FPKM value 698 taken logarithm (LOG 2).

## 5. Conclusions

GTFs were demonstrated to play a crucial role in plant development and adaptation to the environment. In this study, we systematically identified 1 TBP and 21 putative TAFs in the mungbean genome. These factors are essential components of the TFIID complex and are critical for maintaining normal plant growth. Under high-salinity stress, *VrTBP*, *VrTAF2*, and *VrTAF15–1* are the predominant regulators in both the leaves and roots. In contrast, *VrTAF10*, *VrTAF11*, and *VrTAF13* may have greater significance in the leaves, while *VrTAF12*, *VrTAF12b*, and *VrTAF15–2* are the primary contributors in the roots. When exposed to water-deficit stress, only *VrTAF2* is induced in both the leaves and roots at all time points, followed by *VrTAF5–2*, *VrTAF8–1*, *VrTAF12b*, *VrTAF15–1*, and *VrTAF15b–2*. Additionally, *VrTAF1* and *VrTAF9* respond to water-deficit stress exclusively in the leaves. Meanwhile, the induction or inhibition of *VrTAF5–1*, *VrTAF6–1*, *VrTAF14b–1*, and *VrTAF14b–2* was observed solely in the roots. In terms of the ambient temperature response, *VrTAF5–1*, *VrTAF6–1*, *VrTAF9–2*, *VrTAF10*, *VrTAF13*, *VrTAF14b–2*, and *VrTAF15–1* may play a critical role due to their contrasting patterns against heat and cold. However, further validation and studies on the molecular mechanisms are still necessary. In conclusion, our current research provides a novel perspective on understanding plant stress responses that could offer valuable insights into breeding improvement.

## Figures and Tables

**Table 1:** *TBP* and *TAF* genes in the mungbean genome.

Name	Gene	Locus ID	NCBI Protein	NCBI mRNA	Location	Length (aa)	pI	M.W. (kDa)	Predicted Cell Localizations
*VrTBP*	*Vradi0305s00090.1*	*LOC106754936*	XP_014492490.1	XM_014637004.2	NW_014543812.1 (24061..29626, complement)	200	9.68	22.38	Nucleus
*VrTAF1*	*Vradi11g07970.1*	*LOC106777544*	X1:XP_014520632.1 X2:XP_014520633.1 X3:XP_014520634.1 X4:XP_022631751.1	X1:XM_014665146.2 X2:XM_014665147.2 X3:XM_014665148.2 X4:XM_022776030.1	Chr 11, NC_028361.1 (8353773..8377433)	1901 1767 1752 1746	5.62 6.40 6.98 6.90	215.26 200.48 198.72 197.99	Nucleus
*VrTAF2*	*Vradi07g18120.1*	*LOC106768217*	X1:XP_014508704.1 X2:XP_022639382.1 X3:XP_022639383.1	X1:XM_014653218.2 X2:XM_022783661.1 X3:XM_022783662.1	Chr 7, NC_028357.1 (39566756..39586448)	1384 1354 939	6.23 6.41 6.26	156.59 153.22 106.88	Nucleus
*VrTAF4b*	*Vradi06g14750.1*	*LOC106764661*	X1:XP_014504463.1 X2:XP_014504464.1 X3:XP_014504465.1 X4:XP_014504466.1 X5:XP_014504467.1 X6:XP_014504469.1 X7:XP_022638246.1	X1:XM_014648977.2 X2:XM_014648978.2 X3:XM_014648979.2 X4:XM_014648980.2 X5:XM_014648981.2 X6:XM_014648983.2 X7:XM_022782525.1	Chr 6, NC_028356.1 (34643049..34655243)	936 936 936 936 932 845 845	8.38 8.38 8.38 8.38 8.38 9.39 9.39	101.74 101.74 101.74 101.74 101.33 92.01 92.01	Nucleus
*VrTAF5–1*	*Vradi06g13500.1*	*LOC106765332*	X1:XP_022638193.1 X2:XP_014505399.1	X1:XM_022782472.1 X2:XM_014649913.2	Chr 6, NC_028356.1 (32613390..32624024, complement)	659 658	6.37 6.37	72.96 72.82	Nucleus
*VrTAF5–2*	*Vradi05g10920.1*	*LOC106762344*	XP_014501696.1	XM_014646210.2	Chr 5, NC_028355.1 (19815499..19824598, complement)	671	6.30	74.27	Nucleus
*VrTAF6–1*	*Vradi04g10770.1*	*LOC106758550*	XP_014496955.1	XM_014641469.2	Chr 4, NC_028354.1 (19764236..19770172)	543	6.30	60.42	Nucleus
*VrTAF6–2*	*Vradi10g02250.1*	*LOC106775101*	XP_014517632.1	XM_014662146.2	Chr 10, NC_028360.1 (6707871..6712998)	536	7.31	59.80	Nucleus
*VrTAF6–like*	*/*	*LOC111240733*	XP_022632040.1	XM_022776319.1	NW_014541992.1 (22204..24055)	192	5.49	21.37	Nucleus
*VrTAF7*	*Vradi06g12480.1*	*LOC106763916*	(1)XP_014503580.1 (2)XP_022638147.1	(1)XM_014648094.2 (2)XM_022782426.1	Chr 6, NC_028356.1 (30127567..30130853)	199	4.60	22.22	Nucleus
*VrTAF8–1*	*Vradi04g09290.1*	*LOC106759260*	XP_022635899.1	XM_022780178.1	Chr 4, NC_028354.1 (18214406..18217173)	406	5.55	44.92	Nucleus
*VrTAF8–2*	*Vradi06g06010.1*	*LOC106764373*	XP_014504145.1	XM_014648659.2	Chr 6, NC_028356.1 (7592855..7594847, complement)	290	9.22	32.59	Nucleus
*VrTAF8–like*	*Vradi08g07170.1*	*LOC106769930*	XP_014511223.1	XM_014655737.2	Chr 8, NC_028358.1 (18416509..18418289, complement)	350	5.97	38.49	Nucleus
*VrTAF9–1*	*Vradi06g02960.1*	*LOC106764413*	X1:XP_014504185.1 X2:XP_014504186.1	X1:XM_014648699.2 X2:XM_014648700.2	Chr 6, NC_028356.1 (3016606..3019713, complement)	189 175	4.49 4.48	21.32 19.68	Nucleus
*VrTAF9–2*	*Vradi07g24390.1*	*LOC106767081*	XP_014507387.1	XM_014651901.2	Chr 7, NC_028357.1 (47737783..47740700, complement)	177	5.08	20.20	Nucleus
*VrTAF10*	*Vradi04g11480.1*	*LOC106758746*	(1)XP_014497210.1 (2)XP_014497209.1	(1)XM_014641724.2 (2)XM_014641723.2	Chr 4, NC_028354.1 (20649254..20652967, complement)	136	5.16	15.26	Nucleus
*VrTAF11*	*Vradi09g05150.1*	*LOC106773625*	XP_014515833.1	XM_014660347.2	Chr 9, NC_028359.1 (7507755..7510113)	204	7.04	23.07	Nucleus
*VrTAF12*	*Vradi08g05550.1*	*LOC106772695*	(1)XP_014514733.1 (2)XP_014514734.1 (3)XP_022640289.1	(1)XM_014659247.2 (2)XM_014659248.2 (3)XM_022784568.1	Chr 8, NC_028358.1 (12062609..12070416)	504	10.12	53.02	Nucleus
*VrTAF12b*	*Vradi09g02430.1*	*LOC106774141*	XP_014516496.2	XM_014661010.2	Chr 9, NC_028359.1 (2520168..2532650)	1064	8.58	116.82	Nucleus
*VrTAF13*	*Vradi08g10630.1*	*LOC106770840*	(1)XP_014512148.1 (2)XP_022640670.1 (3)XP_022640669.1 (4)XP_014512151.1 (5)XP_014512149.1 (6)XP_014512150.1	(1)XM_014656662.2 (2)XM_022784949.1 (3)XM_022784948.1 (4)XM_014656665.2 (5)XM_014656663.2 (6)XM_014656664.2	Chr8, NC_028358.1 (28680758..28685336, complement)	136	5.31	15.32	Nucleus
*VrTAF14b–1*	*Vradi11g06360.1*	*LOC106777003*	XP_022631586.1	XM_022775865.1	Chr11, NC_028361.1 (6305127..6307465)	279	6.07	31.27	Chloroplast, Nucleus
*VrTAF14b–2*	*Vradi0393s00050.1*	*LOC106780520*	XP_014524308.1	XM_014668822.2	NW_014542625.1 (46546..49945)	273	7.17	30.73	Nucleus
*VrTAF15–1*	*Vradi02g02560.1*	*LOC106777580*	XP_014520706.1	XM_014665220.2	Chr2, NC_028352.1 (2396952..2402492, complement)	390	8.28	43.21	Nucleus
*VrTAF15–2*	*Vradi05g09940.1*	*LOC106760812*	XP_014499728.1	XM_014644242.2	Chr5, NC_028355.1 (18244440..18246193)	156	8.78	17.08	Nucleus
*VrTAF15b–1*	*Vradi0153s00320.1*	*LOC106752587*	XP_014489787.1	XM_014634301.2	NW_014542917.1 (751087..753754, complement)	422	9.00	43.30	Chloroplast
*VrTAF15b–2*	*Vradi0158s00270.1*	*LOC106752498*	XP_022632845.1	XM_022777124.1	NW_014542914.1 (178992..183259)	524	8.92	51.08	Nucleus

## Data Availability

Data is contained within the article and Supplementary Material.

## Supplementary Materials

The following supporting information can be downloaded at: https://www.mdpi.com/article/10.3390/ijms25179558/s1.

## References

[1] Akhtar W., Veenstra G.J. (2011). TBP-related factors: A paradigm of diversity in transcription initiation. Cell Biosci..

[2] Roeder R.G. (2019). 50+years of eukaryotic transcription: An expanding universe of factors and mechanisms. Nat. Struct. Mol. Biol..

[3] Ravarani C.N.J., Flock T., Chavali S., Anandapadamanaban M., Babu M.M., Balaji S. (2020). Molecular determinants underlying functional innovations of TBP and their impact on transcription initiation. Nat. Commun..

[4] Davidson I. (2003). The genetics of TBP and TBP-related factors. Trends Biochem. Sci..

[5] Albright S.R., Tjian R. (2000). TAFs revisited: More data reveal new twists and confirm old ideas. Gene.

[6] Kramm K., Engel C., Grohmann D. (2019). Transcription initiation factor TBP: Old friend new questions. Biochem. Soc. Trans..

[7] Savinkova L.K., Sharypova E.B., Kolchanov N.A. (2023). On the role of TATA boxes and TATA-binding protein in *Arabidopsis thaliana*. Plants.

[8] Hahn S. (1998). The role of TAFs in RNA polymerase II transcription. Cell.

[9] Louder R.K., He Y., Lopez-Blanco J.R., Fang J., Chacon P., Nogales E. (2016). Structure of promoter-bound TFIID and model of human pre-initiation complex assembly. Nature.

[10] Patel A.B., Louder R.K., Greber B.J., Gruberg S., Luo J., Fang J., Liu Y.T., Banish J., Hahn S., Nogales E. (2018). Structure of human TFIID and mechanism of TBP loading onto promoter DNA. Science.

[11] Leurent C., Sanders S.L., Demeny M.A., Garbett K.A., Ruhlmann C., Weil P.A., Tora L., Schultz P. (2004). Mapping key functional sites within yeast TFIID. Embo J..

[12] Huisinga K.L., Pugh B.F. (2004). A genome-wide housekeeping role for TFIID and a highly regulated stress-related role for SAGA in *Saccharomyces cerevisiae*. Mol. Cell.

[13] Timmers H.T.M. (2021). SAGA and TFIID: Friends of TBP drifting apart. Biochim. Biophys. Acta Gene Regul. Mech..

[14] Wang H.B., Dienemann C., Stutzer A., Urlaub H., Cheung A.C.M., Cramer P. (2020). Structure of the transcription coactivator SAGA. Nature.

[15] Moraga F., Aquea F. (2015). Composition of the SAGA complex in plants and its role in controlling gene expression in response to abiotic stresses. Front. Plant Sci..

[16] Mishal R., Luna-Arias J.P. (2022). Role of the TATA-box binding protein (TBP) and associated family members in transcription regulation. Gene.

[17] Levine M. (2011). Paused RNA polymerase II as a developmental checkpoint. Cell.

[18] Zou Y.Y., Huang W., Gu Z.L., Gu X. (2011). Predominant gain of promoter TATA box after gene duplication associated with stress responses. Mol. Biol. Evol..

[19] Bhuiyan T., Timmers H.T.M. (2019). Promoter recognition: Putting TFIID on the spot. Trends Cell Biol..

[20] Basehoar A.D., Zanton S.J., Pugh B.F. (2004). Identification and distinct regulation of yeast TATA box-containing genes. Cell.

[21] Luna-Arias J.P., Castro-Muñozledo F. (2024). Participation of the TBP-associated factors (TAFs) in cell differentiation. J. Cell. Physiol..

[22] Ruppert S., Wang E.H., Tjian R. (1993). Cloning and expression of human TAFII250: A TBP-associated factor implicated in cell-cycle regulation. Nature.

[23] Aoyagi N., Wassarman D.A. (2001). Developmental and transcriptional consequences of mutations in *Drosophila* TAF(II)60. Mol. Cell Biol..

[24] Georgieva S., Kirschner D.B., Jagla T., Nabirochkina E., Hanke S., Schenkel H., de Lorenzo C., Sinha P., Jagla K., Mechler B. (2000). Two novel *Drosophila* TAF(II)s have homology with human TAF(II)30 and are differentially regulated during development. Mol. Cell. Biol..

[25] Uffenbeck S.R., Krebs J.E. (2006). The role of chromatin structure in regulating stress-induced transcription in *Saccharomyces cerevisiae*. Biochem. Cell Biol..

[26] Kim N.R., Yang J., Kwon H., An J., Choi W., Kim W. (2013). Mutations of the TATA-binding protein confer enhanced tolerance to hyperosmotic stress in *Saccharomyces cerevisiae*. Appl. Microbiol. Biotechnol..

[27] Lago C., Clerici E., Mizzi L., Colombo L., Kater M.M. (2004). TBP-associated factors in *Arabidopsis*. Gene.

[28] Gasch A., Hoffmann A., Horikoshi M., Roeder R.G., Chua N.H. (1990). *Arabidopsis thaliana* contains two genes for TFIID. Nature.

[29] Mougiou N., Poulios S., Kaldis A., Vlachonasios K.E. (2012). *Arabidopsis thaliana TBP-associated factor 5* is essential for plant growth and development. Mol. Breed..

[30] Gao X., Ren F., Lu Y.T. (2006). The *Arabidopsis* mutant *stg1* identifies a function for TBP-associated factor 10 in plant osmotic stress adaptation. Plant Cell Physiol..

[31] Tamada Y., Nakamori K., Nakatani H., Matsuda K., Hata S., Furumoto T., Izui K. (2007). Temporary expression of the *TAF10* gene and its requirement for normal development of *Arabidopsis thaliana*. Plant Cell Physiol..

[32] Robles L.M., Wampole J.S., Christians M.J., Larsen P.B. (2007). *Arabidopsis enhanced ethylene response 4* encodes an EIN3-interacting TFIID transcription factor required for proper ethylene response, including *ERF1* induction. J. Exp. Bot..

[33] Kubo M., Furuta K., Demura T., Fukuda H., Liu Y.G., Shibata D., Kakimoto T. (2011). The *CKH1/EER4* gene encoding a TAF12-Like protein negatively regulates cytokinin sensitivity in *Arabidopsis thaliana*. Plant Cell Physiol..

[34] Kim J.S., Sakamoto Y., Takahashi F., Shibata M., Urano K., Matsunaga S., Yamaguchi-Shinozaki K., Shinozaki K. (2022). *Arabidopsis* TBP-ASSOCIATED FACTOR 12 ortholog NOBIRO6 controls root elongation with unfolded protein response cofactor activity. Proc. Natl. Acad. Sci. USA.

[35] Lindner M., Simonini S., Kooiker M., Gagliardini V., Somssich M., Hohenstatt M., Simon R., Grossniklaus U., Kater M.M. (2013). TAF13 interacts with PRC2 members and is essential for *Arabidopsis* seed development. Dev. Biol..

[36] Choi K., Kim J., Hwang H.J., Kim S., Park C., Kim S.Y., Lee I. (2011). The FRIGIDA complex activates transcription of FLC, a strong flowering repressor in *Arabidopsis,* by recruiting chromatin modification factors. Plant Cell.

[37] Eom H., Park S.J., Kim M.K., Kim H., Kang H., Lee I. (2018). TAF15b, involved in the autonomous pathway for flowering, represses transcription of *FLOWERING LOCUS C*. Plant J..

[38] Dong O.X., Meteignier L.V., Plourde M.B., Ahmed B., Wang M., Jensen C., Jin H.L., Moffett P., Li X., Germain H. (2016). *Arabidopsis* TAF15b localizes to RNA processing bodies and contributes to *snc1*-mediated autoimmunity. Mol. Plant-Microbe Interact..

[39] Zhu Q., Ordiz M.I., Dabi T., Beachy R.N., Lamb C. (2002). Rice TATA binding protein interacts functionally with transcription factor IIB and the RF2a bZIP transcriptional activator in an enhanced plant in vitro transcription system. Plant Cell.

[40] Zhang Y., Iqbal M.F., Wang Y.L., Qian K.Y., Xiang J.X., Xu G.H., Fan X.R. (2022). OsTBP2.1, a TATA-binding protein, alters the ratio of *OsNRT2.3b* to *OsNRT2.3a* and improves rice grain yield. Int. J. Mol. Sci..

[41] Zhang Y., Zhao L., Xiao H., Chew J., Xiang J., Qian K., Fan X. (2020). Knockdown of a novel gene *OsTBP2.2* increases sensitivity to drought stress in rice. Genes.

[42] Zhang L., Wang R., Xing Y., Xu Y., Xiong D., Wang Y., Yao S. (2021). Separable regulation of *POW1* in grain size and leaf angle development in rice. Plant Biotechnol. J..

[43] Jiang L., Jiang N., Hu Z., Sun X., Xiang X., Liu Y., Wu M., Liu C., Luo X. (2022). TATA-box binding protein-associated factor 2 regulates grain size in rice. Crop J..

[44] Parvathi M.S., Nataraja K.N., Reddy Y.A.N., Naika M.B.N., Gowda M.V.C. (2019). Transcriptome analysis of finger millet (*Eleusine coracana* (L.) Gaertn.) reveals unique drought responsive genes. J. Genet..

[45] Yundaeng C., Somta P., Chen J.B., Yuan X.X., Chankaew S., Chen X. (2021). Fine mapping of QTL conferring Cercospora leaf spot disease resistance in mungbean revealed *TAF5* as candidate gene for the resistance. Theor. Appl. Genet..

[46] Lambrides C.J., Godwin I.D., Kole C. (2007). Mungbean. Pulses, Sugar and Tuber Crops. Genome Mapping & Molecular Breeding in Plants.

[47] Wu R.R., Zhang Q.X., Lin Y., Chen J.B., Somta P., Yan Q., Xue C.C., Liu J.Y., Chen X., Yuan X.X. (2022). Marker-assisted backcross breeding for improving bruchid (*Callosobruchus* spp.) resistance in mung bean (*Vigna radiata* L.). Agronomy.

[48] Pratap A., Gupta S., Rathore M., Basavaraja T., Singh C.M., Prajapati U., Singh P., Singh Y., Kumari G., Pratap A., Gupta S. (2021). Mungbean. The Beans and the Peas: From Orphan to Mainstream Crops.

[49] Wu C.J., Liu Z.Z., Wei L., Zhou J.X., Cai X.W., Su Y.N., Li L., Chen S., He X.J. (2021). Three functionally redundant plant-specific paralogs are core subunits of the SAGA histone acetyltransferase complex in *Arabidopsis*. Mol. Plant.

[50] Hoffmann A., Sinn E., Yamamoto T., Wang J., Roy A., Horikoshi M., Roeder R.G. (1990). Highly conserved core domain and unique N-terminus with presumptive regulatory motifs in a Human TATA Factor (TFIID). Nature.

[51] Vogel J.M., Roth B., Cigan M., Freeling M. (1993). Expression of the two maize TATA binding protein genes and function of the encoded TBP proteins by complementation in yeast. Plant Cell.

[52] Davidson I., Kobi D., Fadloun A., Mengus G. (2005). New insights into TAFs as regulators of cell cycle and signaling pathways. Cell Cycle.

[53] Waterworth W.M., Drury G.E., Blundell-Hunter G., West C.E. (2015). *Arabidopsis* TAF1 is an MRE11-interacting protein required for resistance to genotoxic stress and viability of the male gametophyte. Plant J..

[54] Benhamed M., Bertrand C., Servet C., Zhou D.X. (2006). *Arabidopsis GCN5*, *HD1*, and *TAF1/HAF2* interact to regulate histone acetylation required for light-responsive gene expression. Plant Cell.

[55] Bertrand C., Benhamed M., Li Y.F., Ayadi M., Lemonnier G., Renou J.P., Delarue M., Zhou D.X. (2005). *Arabidopsis* HAF2 gene encoding TATA-binding protein (TBP)-associated factor TAF1, is required to integrate light signals to regulate gene expression and growth. J. Biol. Chem..

[56] Lawrence E.J., Gao H., Tock A.J., Lambing C., Blackwell A.R., Feng X., Henderson I.R. (2019). Natural variation in *TBP-ASSOCIATED FACTOR 4b* controls meiotic crossover and germline transcription in *Arabidopsis*. Curr. Biol..

[57] Lago C., Clerici E., Dreni L., Horlow C., Caporali E., Colombo L., Kater M.M. (2005). The *Arabidopsis* TFIID factor AtTAF6 controls pollen tube growth. Dev. Biol..

[58] Avendano-Borromeo B., Narayanasamy R.K., Garcia-Rivera G., Labra-Barrios M.L., Lagunes-Guillen A.E., Munguia-Chavez B., Castanon-Sanchez C.A., Orozco E., Luna-Arias J.P. (2019). Identification of the gene encoding the TATA box-binding protein-associated factor 1 (TAF1) and its putative role in the heat shock response in the protozoan parasite *Entamoeba histolytica*. Parasitol. Res..

[59] Parvathi M., Nataraja K.N. (2017). Discovery of stress responsive TATA-box binding protein associated Factor6 (TAF6) from finger millet (*Eleusine coracana* (L.) Gaertn). J. Plant Biol..

[60] Ben-Shem A., Papai G., Schultz P. (2021). Architecture of the multi-functional SAGA complex and the molecular mechanism of holding TBP. FEBS J..

[61] Nagy Z., Riss A., Romier C., le Guezennec X., Dongre A.R., Orpinell M., Han J., Stunnenberg H., Tora L. (2009). The human SPT20-containing SAGA complex plays a direct role in the regulation of endoplasmic reticulum stress-induced genes. Mol. Cell. Biol..

[62] Imran M., Shafiq S., Farooq M.A., Naeem M.K., Widemann E., Bakhsh A., Jensen K.B., Wang R.R.C. (2019). Comparative genome-wide analysis and expression profiling of histone acetyltransferase (HAT) gene family in response to hormonal applications, metal and abiotic stresses in Cotton. Int. J. Mol. Sci..

[63] Ogata Y., Kimura N., Sano R. (2019). Gcorn Plant: A database for retrieving functional and evolutionary traits of plant genes. Plant Physiol..

[64] Kang Y.J., Kim S.K., Kim M.Y., Lestari P., Kim K.H., Ha B.K., Jun T.H., Hwang W.J., Lee T., Lee J. (2014). Genome sequence of mungbean and insights into evolution within *Vigna* species. Nat. Commun..

[65] Chen C., Wu Y., Li J., Wang X., Zeng Z., Xu J., Liu Y., Feng J., Chen H., He Y. (2023). TBtools-II: A “one for all, all for one” bioinformatics platform for biological big-data mining. Mol. Plant.

