# Supplementary material for: Characterization of TBP and TAFs in Mungbean (Vigna radiata L.) and Their Potential Involvement in Abiotic Stress Response
Source: Int J Mol Sci. 2024 Sep 3;25(17):9558. doi: 10.3390/ijms25179558 (PMC11394781; doi:10.3390/ijms25179558)
Supplement: Supplementary file 1 [file ijms-25-09558-s001.zip › Supplementary data - sequences.pdf]

I. Sequences from mungbean (*Vigna radiate*)

1. Genomic DNA sequences of *VrTBP* and *VrTAFs*

>VrTBP LOC106754936

CGTTTCTCTTCTCTCGCTATTGCCTCCAGATCTTTTCACACACATTACAAATACCTTTCACTCTCTCTTTCTCTCTCTTCCTCAC
ATTCTCTGGACATACAAACTCTCTCTCTCTCTCTCTCTCTCTCTCTCTCTCTTCAAATCAAAACCTATCACAACATACAAATCT
CAAATCCTCTGTCTATATAGCTCCTTTTCTCATATAAAAGAACTCTTCTTTCGAATCCTTCCCGCTCCCTCTACACCTTTTCACT
CTTTTCCCTCTCGATTCGCTCCACGCGCGCATCCCAACCCTAATTTCTTTCTTATAGGTCTGATTTCACATGCAAATCCCCAAT
TTTTCTTTTTTCTCAAACTAATTTTAAGACACAAAGAGGGGTTCGTTGAATCGGGTGAAAATCTTTGATTAACTGTTTGGGCT
TGTCTTAATTTCCAGATCTGGGTTCTTTGTCTTTTTGGTTTTTAATCTTTAAAGGGTTTTCCTTTTCTTTTGTTAGAGTTGAAAA
AAAACGTAGTGAACTTTTGTTTGGTACTGATTGGTGTTGAACATGGTGGGTTGTTTGTGGTTTTTTTTTTTTGGTTGCTGGCA
GCTCTGCGTTTGGGTTATTAAATTAGGGTTTGATGGCTGACCAAGGATTGGAAGGGAGCCAGCCGGTGGATCTACAGAAGCA
TCCTTCTGGGATCGTGCCTACTCTTCAGTGAGTATCCGTTGTTTTATTTTCAACAAATTACATCGTTCTCCTTTTCTATTGGTTT
CAATTGCAAGCTTCAGATGGCTGGGTTTTAAGTTTTAATTTGTGGGGTTAGCCCCCTTTTTTTCATAATAAGAAGTGACTTTTG
ATGTGTTGCTAGTAATAGCTATCCAGTAAGTCAACCCCCATGTCTTTGTTTCTTCTTAACACTATTGTGTTGCTAGGGAATTCA
GCTTGTAAATATTGATGTAAATCCTTTTGATTGACGATGTAACTGTAGCTGATATTGTTGGGATCGCTTTGTGGTAAAACATGG
TGCAAAGGAGATGGTGGTTATACCTTTAGTTTACATTCAATTATTGATATCTAACTAGTGATGACTGTGGGAGACTCTATGGTG
TCCTAATCAAGGATTAGTAGGCATATGCTTCTTGGTGGTCGGTTTCGAATTACTGACATTGCGGACAATTGTGAACATAAGAA
GTCGATATGGCCAGACAGTATAACAGATAGACCATAAACATAGACATCAAGGTTGAAATTACAGCTATGGACTTGATAGTTAT
TTGAAACTCTGCTAAATGTTAAAAATACCAATACTTTTACAATTTGCGCTATTTAACAAGTACAACGGGCAAAAAAGATACAA
CTTTTAATTCCCAAATTTTCTACTTAAGTTCATCCTAAGCCTGGGTATTGGTAACCACCATGGAGAAGCACAAACATAGGATT
GCAGGGTTTCAGTTAACACCTTGCGACATTTGCCAAAGCATGTATTTATTCCTTATACTCGTATTGCCGAAACTTGTTTTTTAT
GTTGCTTGAATTATGGTGTTTATGCTAAACCATTTTAATTTGAGGTCGTGCCTAACAAGATGTCAAATCATATTGGCAACTTTC
AATCTCCTATGTCCTTTTTCTGGATGGATTTCTCTGTGGACAAAAGCATGTTATTGTGCAGAGTTGATTCATGTCTTTTTACCA
CCTTGTATTTTTGTGTAATCCTTGACCTATCTGTCATACTCGCACCAAATTTGCCACTTTTAATCATAAAATTTGCCTTTGTTTAT
GTGGGAATATATAATAATTTTGCTTCTGTTCACATGGGAATGTTAGGGATTCTGCCTGAATTTACATAGTATTATTTAATAGCTTT
GCTTCTGTTTACAATTTACTTTGGACTATGCAAATGACTTGTTTCTATTTATATTTTTGTACATGATAATTTTTCCTCTATTTACAC
GGTGTAATTTAAAAATCAAGCTGTTTGTATTGCCAGAAATATTGTGTCCACTGTCAATTTGGACTGTAAGTTGGACCTCAAAA
CAATTGCACTACAAGCTCGTAACGCTGAGTACAATCCCAAGGCAAGTTTCGTTTACTTGTAGTGAGTTTCACTCACAGGTGC
TCTCTGTACAGTTGTGGGTGTATACATGTTTTTTTTTTCTGCTTTTGCATCTGAATTACAACAAAGTTTGCTGGTGGCTGTGCA
GTCATGTGTTATGCTTTGGGAATTCTTTTGTTTTTATTTGGATCAGAGAAGTGTGATTAATCTAAGATTGCTATTTTATAGTAATA
TCCCATGTGATATCTTCCACATGTTAGTATACTTGTGTTATTAAGAATTATTAATTAGCGGATGTACTATATATGCTTGCTCTAGT
TGGTTGGTGATTTACTCTACTGATATTTGTGATGTGTTTGCTTGCAGCGTTTTGCTGCTGTTATTATGAGGATAAGAGAGCCGA
AAACAACTGCACTTATTTTTGCTTCTGGCAAGATGGTATACTTCTTTTCTCTACTGCCATGTCTGTCTACGAGATGAGAGTAGT
TAATTGTGCTATATTCAGCACGGTCCTCAACTTAGTTCAATGTTAATTCGTAGTTGTGTGGTTCTCTTGTCCCATTATAATTGTC
TGTGTTTATCAAATCAATGCACAAACTGGTGTTGGTTAGAACCTGTAAAATTCAAGCTTGTCTTTGTCTTAAGTATATCATTGA
AGGTTCTAGTAATGAGGTTGTAAACACAGTTGCCACTTGTCAGAATTGCATGAATCAATTGGTTCTGTATAGCATTATGAGAG
AAAAGGAGAGATGATGCATGGAGCAACCTCAATGACCCAATTATTAATTTATGCAAAATTGTAAATGAAGAAGACATTCTATT
TCTAAACTTATTTTTACAGTTTTATTTGTTTTTTATTTCAATAATTTAGCGTCAATTTTGCAATTTACTATCAAATCAGATCGTAG
GCCTTCTGGTTTGCAATTTTCTTTTCTGGATTCTGTCATTGCTCTACATTTTTATTACAAAACCAAATTAATTTGGTACCTTTCT
TCTAAAGGTTTCTCTTTCTTATGGACTCTGATTTATTCTTTTATGATCATCAACTAGGTTTGTACTGGAGCTAAGAGTGAACAA
CAGTCTAAATTGGCAGCCAGGAAGGTATGTTACTGTTTTATGTTTTTTTTCTCTATCATTCTGTGGGTATTGATGATTTTTTTTT
CTTCAAATTGAAGCCCTAACCACTTTGTTGGTAGCTTGTTTTTGGAACTGTTCTTGTTTTGAGTAGACTGAGCTCTTAAATAG
TTGAAGGAGGCTGGATGTTTTCTCTGAATGTGTTTAATATTGATCTTTTGGTTCCTCTCTTGAGTAAGAATTGCGAAGTTCATT
CTGAAAAAGTTACATAATTTCTGATGTTTTCTATTTTCCCCCTTTCAGTATGCTCGAATTATCCAAAAGCTTGGTTTCCCAGCC
AAATTTAAGGTATTTCTTTTGCTCTTTATCATAATTTTTTTCTGAAATTGTTTTATCCATTGTTATTTTTCACTTAACTATGTACAG
CTTAGTTACTAGTTTATTCCATCGGTATTGTACTTTTAGGATTTCAAAATTCAGAACATTGTTGGCTCTTGTGATGTCAAGTTCC
CCATTCGGCTGGAGGGTCTTGCGTATTCTCATGGTGCTTTCTCAAGTGTAAGCTTTTTAACTTTTGGTAAAACACTTTACGGG
CTTGGATCGGATCTATGATGTTGACTTTTAAAATAAAACTGTTATGTATTTTCACTGTCTTGATGCTTAGCTGTCTCTATGTAAA
ACAGCTTTAAGGGCGTCTTTTTGAATTGTATACTTGATGTGAAAATAGCCTATGGAAAAAATGTAATTTGTTTGAAGCATTATG
TTGCAAAGCTATTTTAATGTGTTGCCTAACACTTTGGATACTTAATACATTGGTATGCATGGGTTATTAGTTGTGGGGATTGATT
TGACATGATTTGTGAGGGTTGCTTGTATTTGGTTAAGGTGATTTGTGGGGAAAGTTTTGAAAGAGAATGAAGAGTGAGACGA
GGACAAAGTATATATGTGGGGATTGTTTGATTAATTACTAATGTACCCCTACATCTAATATTTGATTTTTTAAATAATTCTATTGG
AATTAATATATATATAATATTTAAGAAATAATTTATTTTCATTTACCCAGTTAATTTCTTTTAACCAAAAAATTTATATTTTAAAAAAT
ACAACTACGGACAATTTTGATATTACACTAAAACAACTTGCCAATCTTCATAAATTCAATCAAATACCAATAAACTTTCTACTC
AAATCCATTATCTTCCATTAAACCAAACCACCTCACTTTCTATTCCCTTTCTCTTAAAATCAATCCTCCACAATCCACCCCTCA
ACCAAACAAAGCATAAAGGAGGGTAAGGGTTTTTGTTTTCGAAAAATTTAAATAGAGTGAAATGGTTAAACCGGAATAATAT
TATTATAAATCACTATGCTCGTGTGATAAAGTAAATTGAGGAAGTATAAATTGTTGAAAGGGGCATCTTCTGTTAAGTTTTGAA
GTAGAGGAAACTGATGGTTATTTGCTGATACTTTGCTTGTTGTGGTTGTAGTATGAACCAGAGCTGTTTCCAGGTCTAATCTA
CCGTATGAAGCAACCTAAAATAGTGTTGCTTATATTTGTCTCTGGAAAAATTGTTCTAACAGGAGCTAAGGTGATTTTTTTTGC
TTTACAATTTCCCATTGTAATTGCCCTCCACCCGCGCCCCCTTTTTTGTGCTGGATTTTAGCTGTTTTCATTTCCATTTTCAGGT
GAGAGATGAGACGTACACTGCCTTTGAAAACATATATCCTGTACTTACCGAATTCAGGAAAAACCAACAATGGTACGAATTT
GTCTTTATATATTTAATTTTCTCTATACATACAAGTGTAATTTCAACAATTTCCATAAGGGTGGCTATCTTTTGTTTAGCCATCAG
ATTTAATGCGTTAGATTTGGATCTGGTAAGGTAGTACTTTTTGTGAGTAGCGATGTAGTTATACTTGATGCATTCGTAAAATGA
ATTGATTTTTTGCTACTTTTGAACTCCGTTTCTGGCTATAAAACTGTTTGACATGGTTATGGCAGATTTGTCAAGAAAGAGCA
GGAGCAGCGGGACTTGCCGAGAAGTTCGTGGAACTGTAAATACTAAGACAAAGGAGATGATATCTAGATTGATATAAATTAA
AGACAGCCGTTTGTGTTGCCTTGTATCTCTTTTTTGATTGATCTTCTTGATGTAGTAAAGTGCCAGACATTGTTCTATTGATTTA
AAATTTTAAAAAAAGATGATTTTGAGTCAGAAATTAGAAAAAAAAAAAGATGGAGAGCAAAATGCAGTAGTAGCACATTCAG
TCCTGACTTTTCAATCTGTTTTGATAACTGTTTCTACTAATTATCCTTAGTGTGTACTGGACATTTTGATCAACAAATCTAATAT
TTTGATGATGGTACTACTTGAATTCAGTATTTTGGACATGGATTTAGTTCCAAAATCTGTAGTTCTCAAGAGAATAAAGTTAAA
CTTGTTCATTAGAA

>VrTAF1 LOC106777544

CAAGAATATCACCCTGGTGGTATCACTTTCAAAATAAATAACACAGCTTGCGGCATCAACAACATGCAACTCAATTCGGAATA
AAGGGTCTTCTCGACGAACACCCTGGTCGGCGTCGAACACGTGGTCGCAGATCTCGAGCGTCTTCTTGAGGGTCTTCTCAG
CCTAACGCCCTCCTCCGCCACTCCGCATGCTCCCGCCTCCCTCCTCTACCGCGACAACCCAATGAAATGAACGATCTCATCTT

CAATTAAATAACAGACCGTGCAATAACAACAAGCTGCAACTCAATTAGGAAATTTCTGATAAACTCTAAAGGCTGATTCGCC
GGAACCCTTCGTCCGTTCACCAACAGGCCAGGGCAGTATTCTGTTCTGCTGAATTTAATCTGGTCCGCGGCGAGGAAACCTA
ATTTGCGGCTTTGTTTTCTTTAGGGGACGAAGGGAAAAATTGCAGAGTGGAATAGGGTTAGTGAAGTTTAAATAGGGTTTTC
GAGGCACAGAAGCACTAGAAGAAGGCAGTAATCGAAATTGATTTTGCTGGAGTTGTGTTTTGGAGCTGCTGTGATGAGTTC
GTGCTGAATTGGGCTATAATTCGTATAGCTCTGGTCCGACAGCATGGGTTACGATTCGGCTAGCCCCTCGCAGGATGGGAGGG
ACGAAGGTAAGTGTCTGCAATCAAAATTGGGGAATTGTAGCATGGGAAGTGGTAGGAACAGGGGAAAGAAAAAAATGAAA
ATAAAGGAAAAAAAATGCTTCAATAGGGAAATGGGTTGCGTAATTGGATGTGCAGTCTAGTTGTTCAGTGCTGTAGGATCGG
AACATTTTTTGTTTGCTTGTTCTGGCTGTATGCTATCATATCTAGGGCTAGAATTGGGAGTAGGATTGAAATGGTTATGCTGAA
TTTTAAGTTAGTGGCTATGTGCTGAAATAGGATATCTGTAAGCTGAAGTTCACGTGATGTTCTAGTATGTTATATGGTTTGTTGT
TTGGTATAGTTTGTTAACTCCATGTCTGATGGATTTGAAATGATAGATGATGAAGAAGAATATGAGGAGTCTGGCAAGGGTAA
TCGGTTTCTTGGGTTCATGTTTGGAAATGTAGATAATTCTGGTGATCTCGATGTTGACTATCTTGATGAGGTAATTTTATGCCGC
CTTTAATGTTGCATTTGCATTTAGTGGGCAAACCATTTAGAAATTCAGGATCGAGCTGATGCATGCATAAATGTCATGCTTAGA
TTTCTTTGTAGTAGTTTTTTTTTTACCAATCATAGTGTTTCTGTTATCTTCCTGGACTTCTATCTACATCTTAGATAGTTATACAG
CCCAAATGAACTTTCATAGCTATCGTATATTTATAATTGGTAAGTAGAGGTTTTTAATTTACTAAACTTCTATTTTAGAAGATTTA
AAGCCACTATATTTGAGATTCGTACTTTGTGGGGAACTTTTACTTGAGTCGTGCATTGTAAGTTCTAGAAAGCAAAAAGGAG
GCATTTTGAAGCTTGTGTTGCTCAGTATGATTGTCCAAAGTCTAAATTATCAAATTACATGCAATTCAATTTTGGAGGATTTTA
GTATTTCGTGTGCAAACTGCAAAGATTTATTTATTATTTGCTATATGTGTATTTGGTTGCCTGTTGGAGAACATCAGAAGTTACT
TTGCCTTTTGGAAACGTCATTTCAGTGAAAATGATGTTTGGTGGTTGTTGGATAGGACCAATTTTGCCTCCAGTCCTTGGTTT
TGACTAGAAGTGATAAAAAGTGGCTTTTTTTTTTTTTTGGGTTAACAACTTTACTTTTTAATTTCATATCTCAAACTTGCATTTTA
GGTAAAATACCTAGACATAAATTATTCCACTTCAAAATCAATTTTAACAAATCCAAAAAATAAGATTTTTCGTAATTAAATTTAT
GTACTAGGCTGCTGTTTTTACTTTATGCCTTTAACTATTTTCTATGACATGGGCATGCCAGATTAGGAATGATCCAGGGAATAAT
AAATTGAAGGTTCTAGGAAAGTTGGTCTTCCTAGAAAACAATTGCACAATTTATGATATGTATACATGTTATGAAAATTAAATT
ATACCGATTGTTGAATGACCAATTGCTTCTTGGTTTATTTATGCATATCTGTTTTGGTGAAAAGCTTTAAAGCTGGTGCTAAAT
AAGCTTATTTCTTCTTTAGGATGCAAAGGAGCATCTTTCTGCATTAGCCGATAAGTTGGGTCCATCACTGACAGATATAGATGT
AAGTAAATCTAAATATCATTACCTGTTTGAGTTTGGAATTCAAAATACTCTCTGTGGAGGCTCTTAAGAAGTGTCTAATTATCA
GATTTTCCTACTGTGTTTCTTTCCTTCAGTTTCTGTTTTGACGATGATAGTAATTTATTTGCAGTTGTCAGGAAAATCGCCACA
AACACCACCTGACGTTGTTGAACAAGGTGAAGTCTGCCAGGAAAAAACTATATCATGCAGTTTTTGATATTCTGTTGGTATCA
TTTTTTTCCCTCATCAACTGAGAGCACTTCACCCGTGTTTTATTTCTACTGAAATTCTTTCCCACATGGCATATGTTTGGTAGA
GAATATCATATAGCAGCTCATTGTCTTTCCTTCTGAAAGAAGAAAATTCCGAACATCTTATGAGAAATTCTGCCACTATACTTT
CTATCACGCTCTATTGTTGATTGAAATTTGTTGGAAAATCACAAAATTTTGTTTCTCACTTCTTATTGATGACTCTTCCCTAATT
TTGTAGTTTATAGTATTGTAAAACTGTTGAGCTGCTGCCATGTGTCATTTCTGTGTACAATTTGGGCCCACTTTTTCCCTCTCAT
GCTCTTGTGCAGAGCACAAACTCATTGCTAGTTTTTAATTAATTTTACCAATAGTCGAGTATGAGCTAAAAAGAATGTTTCACT
AGCATTCCTCATGTTGTATGTATATATATGCATGTATATGGTATTGAAGGCTTAGAGTTAACACTTGGTGTAATTCTCCATTCTGG
AAAATATTTCATGATTTTTTTTTGTAAAACTAAAACACCAGTAGTGATGTCCGAAGCTTGGGGCTAACGGGGCCAGTGGCAC
AAGTCAATAGAAGTAGATCTATAAAACAAACACATGTGACGTTTCGCGGAAGGGTTGCAACTGGTATTAATTAACTTGAGTG
AATAATTGTCATTCATTAGCAGCCATACATTGTTACATATAGATATTGGTTGAACTTAAAATATGGGTCGGAGCTCCTGGCTTCT
GTTTGCTCCAAAAGGATTTTCCTTTGGGGAAGTACATGTTAGTCCAAATGAAGATATATGAAATACTTCATTGGATTTTCTGGG
ATTTGATCTATAGAGGATGTTAAGGTTTTATGCAGTGGAGCTCTTTTTATGGTTTGTTGTGTTTCAAAGCAAAATGCAAGAAA
ATTACAGTGAGTAGCTCCTGCTGTTGAATTATTATTTGGGTTACGGATAGTTTCCATGATTTTTTGTTTTAATTGAACTGTTATC
CTTGCATACGAGGTTCTCGCACAACCGAGTGTCAGTTTGGAGAGACTTCTCCACAAGCTCTCCTGGGTGAGAAAAAAAGAA
GAAAAAGTGAAATCAGTTTTTCTGTAAATTAAATGAACTTGTGAATTGACTAATTTGTATTCACTATTTCCTATTAGCTTTACC
AAAATCTGATTTTTAACTTGTGTAAGTTAATTTCATCTCTTAGAGAAAAACTAATTTCATTTTTCCTTATATTTTTCTTAGAGAA
TTGCTTAGGGAGAAGTTTTCTCTAAACAAGCCCTAGGTCTTCTTGAGTGGCATACCCGAAATTCAGCCATGGGGTCCCACAG
TAATTTTTCTGCATAGTGCATTATACCAATTTTCCTTTGTCATCCTAAGACATAGTCGTGTGACAGTTTTCTCAATTTATTTTGA
GTTACTTTGACAGATTTGCAGAGGGATTTGAGAGTATCTCTGCATGATGTACTGTTAATTTTGTTATTGTTGTTTTCTGTTGGG
AGGATTTCTGACCCTTCTACTCCAACATTTCTTTATCCTTGGAAATTTCATTGGATAAAGCTAATGGACATTGAGGACATTTGT
TGTCTGGAAAATAGTTTGAATATGACACAGTGAAGCCTTGTTTAAAAGAGCAATATAAGCATGTCTTTTCTGCTTTTCTGTAA
AATAGTTTACTAACTTTGGATTCTTTTAATGTTAGACTGTGATGAAAAAGCTGAAGACGCTGTTGATTATGAAGATATTGATGA
AGAGTATGATGGTCCAGAGACAGAGGCTGCCAATGAAGAAGACTATTTATTGCCAAAAAAAGAATTTTTCTCTGCTGAAGCA
TCTGTATGTATGGAATCCAAAGCTTCTGTATTTGATGATGAAAATTATGATGAAGAATCTGAGAAGGAGCAAGACTTTCTGAA
TGAAGATTCTAAACCTGATAATATCTCCTTACCTGGTAATAATTTTTTACACGAATTGCACCACCACTTTGTTGTTGTTGTATTT
TGTCTGGTTTTAACTTAAAACATGAATTTACTTGTTATGTTGGTTTTTCTCCATATAAAAGGGCTAGAAAGATTGATTCTTGTAA
TCTAGCTATGGCATTTTTATGCAGCCTTGTTCAGATGTTGGCTGGAGACAGATTTATGGTGTGTTTGGATTTCAATTTTCAGTT
TTCTTTGAACACTGTGCCAACATATATGAACCTTTTTTCTTGGATCCAGTCAGAAACAAAGTTCACCTCAAACTTCAATTAGC
TTAAAAACTATGATGAGTTGCTTTTTATTTTTTAAAACTAGTTGAGTTCAACTCAACTTAATTACCTAATTGAAATCCAAACAC
ATGTACTGTTATCACGAATGAGAGTGCTTCAACTATTTCACTAAAGTTTTTGTGGCTCTCTTTACGTGCACCTTAAATATAGCT
CTTTTTCACTTTCACTGATGACAGGTTTTGAAGTATATGTTGTTTGATTGATATATTTTCCTTTAGGCATTTTGTTCACTACTAA
GTGATTGTTTAATGACATCCAGTATTCTAGCTAAGCTGTGGATGAGTTTTTGATATTCTGTTTGATGCATGAAATTTAGAAATTG
AATGTGCATGAGTGCGACTAGGAACACTTGTGAACAAATTTTATATATATTAATTCCTTAGCTTGTGAGCTGATTTTCTGAGAA
TTAGTTGTTATATTATGCTACAAATTGTCACTGTAATAAGTTTACTTTGGCTCTATACATTATTGCTAGCCAATATTGGATCTTAA
TTGTTAAACTATAGGAAATGGAGAGACATTGAGTTGAAATTGTGTATATCTGAGACAACAGAGGTGCTTTATTTGTATTGAGA
AAATGAAATCAATACAATAAATAGAAGAGATATGATATGCTCTAATAAAAATATTAATATGACACAGGAATATAAAAATATTCCT
AATAATATTTTATAACCTAGGTATTCCTTTTTCTAAAAGATTATTGAAGATATTCTTGATTATAAATTATCCCTGTCTTGTTTTTAT
AATTATAACATTCCTCCCTCATGCTGGAGCATATAAGTCATGTGCTTATATTGCTGTAAATATGATTAATCCTTGGTGAACATGT
CTACCAACTATTTACTACAATTGACAAATCGAGTGGCGATATTTTCAAACTCCAACTTTTCTCTCATGAAATGATAATTGACCT
CAATATGTTTGAAAGATAAAATTTTAAGCAGTGCGTAATTCTGCTTGATTGTAACATAAAAGGTGTATTTGGGAATTTTCTTCA
AATTTAAGCTCTTGGACGTGTTGCTTTAGACATATGAGCTTATAATTGTCTAATGCTTTTTCTGTTTCTACTCTTGATTTTGCCA
GAACAGTTTGTTTCTTACTTTTTTACAATATTAAGTTACCTCCAACAAGAACACGATACCTGAAGCTTCTATGCTTGATGACAT
TGCCAATCAATGTTAAGTACCAACCAATTTGAGTATTTCCCTCATCTTCATTAATAATGCCTTTAGGATTTAGGGTTTGTACAAC
TGCATCTCAATGGTCTTAATAAAGAGAGTTTATAAATTGACTTACTATACTCATTACAAAGACAATGTTAGGACGTGTCACAAT
GAGACAATTTAGTTTACTAAGTGTTTGTATCTCTTTGGTCAAACGATGTATTGCATATAAGAAAGAAATCAATATTGATATTGTT
TCTTTTATTCCTATTAAAGAAAGAAATGAATAAGACGATTTATGGAATCAAATCTATCCTTTATAAATAAGAAAGGGTTGTGTT
ATGTGTAATAGTAATCTATTAAATGTATTAATTGTTGTTATAGGGTCCAATTTTTTGCAATAAATGATTAATAAGATCCTTACAGG
AGTAAGTTTGACCACTTACTAGAAAGTATTTAAGAAATCAATCCTGGTTTTTTGTGTGTAGACTTTGACTATCAACCAAGTTT
TGTATTTGTCATCTGAACCATCAATATTATATGTTAATTTGAAAGCTTGGAAATTTTAGGAAAGAAAAATAAAATTGCTTGTTT
TCCCTTAATAAATGGTAGATGTACCTCATATCATATTGGAATCTTGCAGTTCACATATGCTAGCTTTGTTCTTTCAGAGGAGCA
GGAAGAGACTTTGGTAGAGGCATCTAAAGAAGAGAGTGCTCTTGAACGTGAATTACATGTTGACTCACTGCAAAGTGAAGA
ATTGGATGCTGATGTACAAAAACCTGAGGAGGTATATTTCAATTTTGCCACCTCTTAAATATTTCTTTTTGGGGTTTGATATTTA
TTCTTCTTGAAATTATAATAGGGTTTTGGGTATGAAGGGATGAAAACAAAATAAGAGAGACTGAATGCACCTTGATTGATATT
TGACTTGATATAAACAACTATCCTTTATTTATTCTTATACTAAATCATAATCTGAAGTTCCAGCTACATGAGGAAATAAATCATA

AAACTAATATATGAAAATGAATCCCAAGAGATAAGATATTCTATGATATTATCTAGATATCATTGGATATTCTTCATATATTCTAA
CAATTCTCATGAATTATATTTAAGTTCTGTACTATTCTGATGATTAAAGGGAATGTATGTACTGTTTTGACTCTGAACCATATTT
GACCTTCAAGTTGTATATTGAATATTTTTTATTAGATTACTTGTAGTCTTGCAGATGAGAATATAAGTGATTCAACTATATAGATA
AAAAGATTTGATCAATGGCTTAGTTGATATAGTGTGGAGGGCTGATCTTCTGATTAATAAGCTGGGTAAATAGAAGAACATTT
TCTGTTGTTAAGAAAACTGATTGATCCTGATATATCTTTTAAATTCGTTTAGACTGGATGCAATCTTAATTAGAAGTAGGAAAT
ACTAAAATTGGTGTACTCATATACGGCCAAACTTGTCCCTAGAGAGAATTAAAGTCAGATCACATAAGTAATTTTTTGACTGA
AGCTGCCTACTCAATGAGTGCATATACTGCATTACTTTATGTAGTACACTATATTGACCCTGAATGATAATCCAGTGTGTTTGTG
TACGTTTATATATATATGTGTGTGTGTGTGTGTGGTTTGAGCCCATGAGCACATGTTAAGGAATTCAGTCCTTTGCCAATGTCA
CCCTAGGATCCTCATTCTTCATAGTTTTGTCCCCTTTTGCTTTTGATTAATTTTAGGGAAAGATAGCTCCCTTATGACCTCAAAC
CCTCCCCTCACACCCTCCCCAAAATGAAATATGAAGATATTAATCTGTAAAGTTGAATAATAAGTTGTTTCGAATACAAAATAA
TATTAATTCAAGTGGACAGCAAATTGAAATCTAAAATAATCTTGGGGACATTGTGGTGCTGTTAGCTCCTTTTATTCAACTCTG
CCTAGTGGGCAAAATTGGTTGTTGTAGTGGAGATGTGGGACAAGCAGTGATATAAGCCTGTGCCAAACAGTTTTAAGTTTTT
GAAGAGTTGCTACTTGAAATATGCATAGTTGCACATAACTCTCGACTTTGTTTTGAAAGGGTGGTTTTACTGGTCTATATACTA
TTTTATTTGATGCATTTAAAAAACAGCAAATAGGATGGCATTTCAGTTTGGTGCTTTGTAAAGGGCTAAAGTGGGGTGGGGA
AGAGTAAAAGGATTTCTCTGTGATGTATGCATCAAATTGATGACAAGTTGGCAACTTGTGTGTGTATTTGCTATTAACTGTGA
AGTATACCAGCTGTAGTACCTTGCTATAAGCCCAGCCAATTAGTAATGCTTGCACTAGCATATTTTTTCTATAACATTGATGATG
ATTATTGTTGATTGGATTAAAAAAGATCATGTTTGACTTTCTATTGACATTAATTTCTTAATTGATGAAACAAACAGGAAGGGA
CTGAAGTCCAGAAAAGGTCTATGGCTATGCCGTTGCCTATTTTATGTGTGGAAGATGGTGTGGCAATTTTACGCTTCTCTGAA
ATCTTTGGCATTCATGAACCTCTCAGAAAGGGAGAAAAGAGAGAACACAGGCAACCTATTACTAGAGGTAATTGGAGATTG
GAAATGTTTACCTGAATTCTCTTTTTTTGTGTCAGTCTATTATATCTTTTAGGAGAGAGATGATTAGAAAATTAAAATTGTTGTT
GCACTATTTGTACAGTATGTGAGGGAAATTAAGGGCTGCCAATGGTGAGATTTGAATGGAAAAATGAGTGCTATCTTTTTGCT
GTGTTTGTATGTGGGGCTTCTGTTCTAATGGGCATTTTACCTGTCATGTTTACAGATAGATACAAGTCTTTGGATTTTACTGATG
ATTTGTAGAAGAGGATGAAGAGGAATTCCTCAAGGGTTCCTCCCAGAGTCTCTCACAGACTAAACAGGTTTCTGTAGTTCA
TAATGATGTATCAGAAAGCAACGATGTTGACTTGGAGTTTCCAAAATTTGGGTTTCTTCATACTGAGCCCTCAGTGGCCAGG
AAAGATGATCATCAATCGAAGGACTCGTGTCATAGTGCTGAACCAATGAAAGGGGATTTTGAAGAAGACCTTTCCTGGAAA
GATCATCCTTTCATATGGACCAACTTTTATCCTCTTGATCAGCAAGACTGGGAAGATGAAATCATTTGGGGCAATTCTCCTGTT
CCAAGTAATAATAATATTGAAAGCTGTGAAGTTTCTGGACCTGAGTTGGGAGTTTCTGGTGGCAGTGAAATAGAAATTGAAA
GTGGGATCCAGAATATTCAGCTGGAGCATCACAAGATACTGGAAGATAAAGATCATAATGTCTTGCTCAGCTCTCCTGTCTCG
TTGGAGGCCTTTGGCTCAAGGGATTCTTCTGAAGCTAAAACCAATCTAATATCTAGAAGTCTTTTTCATCCCCAACTTTTAAG
GTTAGAATCCAGATCTGAAGTGGATAGTTCTAGTCTTCCAGATGGAAAAGAGGGGGAGATATCTAAACATAATCAAAGTGGT
CAAATTACACGTTTTAGCAAGGCTATATCACAAAATAGAGACATGGTGGAGGGTTCCTGGTTAGACGAGATAATATGGGAGG
AGCTTGATCAGCCTATGGTGAAACCAAAGCTTATCTTTGATCTTCAGGATGATCAAATGCACTTCGAAGTTTTGGATAGTAAG
GATGGTGCACATCTTCGTCTTCATGCTGGGGCTATAATTTTAACTCGTTCTTCAAAATCAAGCAGTGGGGACTCATCTGAAGT
ACCAGGACATGGAAGTCAATATGGATGGCGATATGTGTCTAATGACAAACATTATTCAAATCGTAAAACTTCTCAACAATTGA
AATCAAATTCCAAAAAACGCTCGGCACATGGTGTCAAAGTTTTCCACTCTCAACCTGCGTTGAAGCTGCAGACAATGAAATT
GAAGTTGAGCAAGTAAGTATTTATTTGTTTGGAGGAGGTAGCTTTGTTGTCCGTAGCATGTTTTGGCTTAGAATATTATATTCT
TTAAGATGAACTTCAGGTAAAATCTTACATTTAGAGGTAGTTGATGATTAAGCTACTAGATACCCAATATGGTGAGAGCATCAT
ATCTCCACATGTTTTGTGTTTTCTATCGTTCTTTCCTCTATGCAATATTCTTGAATAATATAACTTCAGTCCACAGTTTTTCTCAA
ACCACCATCTTTTTGAAAAAATAATATCTTTGTTCATCTGTCGGAGTGTGGGTGAACTTTTGTTGTACACTTAAATGGATAGAG
TCCATGATTTGTAGACTGGTTACTTGATGCATTATCTTCTTGTAGGTTTGTTGTTTATAATTTTTTCATAGTATACCAATTAAATG
GATCTCATTTCACACGCTGATTCACTTAATATAAATACAAGTTTGCAGTTCATTTCTTTTGTCTCTGAACATTTCATGCAACTAA
ATCCCACTCTGTATACTTAACTGAACCTGTCTCATTCTCCACCATCAAATGGTGCATACATGCACTCTTATAATTTTACTGCATA
ACTTATTTTATGTCCATCAACATAGCTCCTTATCTATTGTCAAACGTCCCTGGTAGCAAAATTGCTGTAGAAATCTCTATCATAG
TGCTTTGGATGATTTCCACCAGTTACAGCTTGTGGTGGATAGAAAGATTGATTCTTTTCATCTTGACCTTTGCATCAGAAATGC
TATTGCAAAACAATAATAGACCTTGACACCCATGCTTTTATTAGAGAACGACTCATTAAATTTGAAGTTTGAACCTAGCTCAC
AGTTTCTGTATTAGCTCATAATCAGTCACCTTTTGACCAGTCCATTTGTTTCTGTTAGTTGTCTATAACAATGCTGATATTTCTTA
TGTTCTTTATCAAGTTCTTGCAAAACCTTTGTTGATTTGTGTATAAGTGAGGCATGCAAGTTGTATAATTTATATGGGGTTTTAA
TATCTATTGTCTACATGTGTATTGTAGATTTTCTGTCATTATAGTCTTACCAACTCTTTTGCAGTAAAGATATTGCAAATTTTCAC
CGGCCAAAAGCATTATGGTATCCCCATGACAATGAGGTTGCTGTCAAAGAACAAGGGAAATTGCCAACACAAGGACCCATG
AAAATTATTATAAAGAGTTTGGGTGGCAAGGGAAGTAAATTGCATGTGGATACTGAAGAAACTCTCTCAACTGTTAAAGCAA
AAGCTTCCAAAAAGCTAGGTTGTGAATTTTTCCTTCTATAATAAAATTTCTTATTTCTGTTATGGATTTAAAATCATTATGAGGC
TGCAAGTTTGTTTTAGATTTTAGTGTACTTTCTCCCTTCTGTATGGCTGATATGAGATTTTTTGTAGATTTTAAGGCATCAGAAA
CAGTGAAAATATTTTATTTAGGGAGGGAGCTTGAAGATCAGAAGTCACTTGCCGAACAAAATGTTCAACCAAACTCCTTGTT
ACATCTTGTTCGTTCAAAGATACATTTGTGGCCAAAAGCACAGAGGGTTCCTGGGGAGAACAAGTCCTTGCGTCCTCCTGG
GGCATTCAAGAAAAAATCTGATCTGTCTGTAAAAGACGGACATGTTTTTCTGATGGAGTAAGTGACTTGAAACTTTTATCCTA
TGTATTTTGAAGCACTGAATTAATATTAACTTATATGTTTTATAATATATTGATATATTCTATGTAGTTGCAAGCTTTTATATTTTAT
GCAATTTGCAAGAGTTTGTCTTCAAATCATTATGGGTCTATTTCATTAATTGAATTTGCATAATATCTGATTCATTCCCCTTTAA
AGCTGTCAAACCTGAGGATCGTAAGATTTGAAGGGGATATAAAATTGTGACTCAAAGGTCATAACTTGAGTCATAAGAGTTA
TGAGTACATGAAAAATCATATTTATAGACTGGTGCATGCATTAAAAATAATATAGACAATAAACTAACTCGTAAGGATCCTCAA
AGTAGTTAGCTTCAATACATAACAGAACTAAAAGCGGGTTAACAATTTATAAAATTACTCTCCTGCAAGAACAGAGAAAAGT
ATTAAGAGGAAAATACAGAGTAGAACACAGTGCGAACCAGTGATGAGGAGGGCAACATCGGCATGATGGTCATGAGTGTTG
TGGTAGCTCACAATGGTGAGGCTAGTGTTCGTAGCAGCAGCATTAGTGCTTCAATGGCTATTGATAACGAGACTGATGGTTGT
TTGACAATAGTAGCAGAACAAAAGGGCCATGTGATTTGGGTTTCTTTTTCAGGTCAACCTTTAGTCTTTTAACCTAGAAATGC
GCTGTTTTAACTCTTGAGCCTGCGTGTTCAAACCAAAGCGGAACAATGTGGTGCACGATCCTGCAATTTCATGAGCAACCAT
GCAATCAGAGTACTGTCTGTGCCTCCTGAATCTGCAATGCGCATGTGTCCCTCAAACTCGCAGGAGTATGTGCGTGTATAACA
ATCTATTTTCTTTTTTATAATTTTTTGTTTTTAAGAATCTTATCTTCGATAAATTAATTTTCAGTGGGAAAAAAGAGAAGCACAA
CTATGGGGAGACTAATATTCTCCTAAATAAACAAAATGAAGAAGAACGAGAGCAAATGTCTTTGTATGCTCTATACTCTTCAA
AGCAATATTGATCAGAGTGTATTCATTAGAGAATCTGTATATTATCAACGAGTCCACTGACATGTAGAAATGGGTGACTTAGTT
TCTTCTTGCATATTCATTCATGGTCGAGTATCTTGTTCTGTTTTTTTCCACTGTCTGTTTTATATTTGTTAGGTTATTCTAAATTTT
TCTTCTTTTCCCTTCGCTCTTGTGTGTTGGTGGTGGTGGGTGGGAGAAGTGTTGAAGGCTAGAAGGAGAATGGTACAGTGCC
ATTTTGGTTAATAATTAATAACATCCTTTGCATCTTGAATTCTCCTAAAACATTTTTGTTTTCACATATTACTGATGCTAAATGCA
ATTTTTTTCTTTTGCCTGGTGATATATCCATTTCCATTTGCAAGTTCTGGGTTGTTGACTTTGGCTGACTAAATTTTGGAAGTAT
CTTTTTTTACCAAACAGCCCTCCAACTTGTTACTTTGTTTGTTGTGTAATTTCATGTTTATATTTCTTCCTTGATTGAAATTTAG
GTAAGACTAGGATCATGGCGTTAGATAGTAAGAGAAATTAGATATGATTATATAAATAGGGAAAGAATGGTAGAAGTATTCATT
AACTTTGGCACAGTTGTAGAGAGGGAATCCCTAGGATAATAAAATTCTACCTACTCAGTTTCTGCCTTAACAATTTTGTGTATT
AGTGAATACTCATCTGAAAATAAAAATCCTTGCAATTTATATTTTTTTTTGCATGTTCTAATGACTTCAACTCTATTATATTAGG
AACAATGTTGCGGTTGACTTTAATGTTGTTTTTTTTTAATTTGTTGCCTGGAATGGGGATACACTTTTGTTTCTTCTTGGGACC
TAGTTAAATGGATGAGGGTTGAGGATACCCCATTAGGATTCTAGGAATTGCATTTATTTTAGTTAGGGGTGGGCAAAGTATACT
AAACTGTACTTAACTATAAATAATTATAGTTAACTATCAAATAGTTCAAATAATCTAAACTGAACTAAAAAACAGTTCAAAAA
AACTAAACTGAACTATTTTGTAGTTCAGGTACACTATAATAGTTCAGTTAACTATATATTTAAGAATTCTGCGTTCAATACATTC

TGCACAAAAGAATATGTTACAGCTTCTGATTTAGGTATATAAAACAAGTTGAGTGTTGATGAAGCCGTGCGACTTCTCCACAG
TGACACAGCTATTTCAAACATGCAATGCCTACAAATTCAGGCCCATTTGCTTAAGCTATATAAAAGCAGACTGTAAATTATATA
AAGAAAAAGTGAAATTTATAGAGGTTTTATGTTAATCAGAAGCTTACATTTGGTTATAATCATATGAAAAAAAAAAAAACCTAG
AAAAGTGATTTTACATATCATTTCTCAATAAATAAGTTTGGATTAAAAAATGTTTTCTTTTTGGTTCACGTTAAAAATTTAAAA
ATATAAACCACAATTACATTCATAAGTATTCATAGAAAATTCAAAATAGTACAATTAAATAATGTTGGGTGGGTGATGAAAAGC
AGTCGACAAACGTCATATGTAATGAAAATCCACCGTATACATTCACGTACAACAGTGTTACACACAGGTCAAACAATTAATTC
AGTTAACTGTACTGAAATATGTAAAGTTCAGTTTTTTTAAACTGAACTAAAGAATAGGTTAATTAGTTTTTAGTATATATTAATT
TAATTTTTTTCAGTATAATAGAGTGCAGATAACTGTAGTATAATACAGTTTAGTAAATTTTGTCCAACCCTAATTTTAGTCTATTT
TACAGTTCGGTTTTCAACTGCACTGTTGGTAGTTAGTTGTTAACATAGCTTTTTGTATCATGCTAGTTGGCTAGTAAGTTACTA
ATGCCAACTGTTAGTAACTGTTTTTTGTAACTAATGTACACATTTTAAATTGCAGTTTGTACTCCTTGACCAACACAATAATATA
TTCTCTCTTTCACACATTTTCTCTTAAGTTTTATATTTCTAACGGCATTGAATATGTTCTTTTTGTAGGTATTGTGAAGAAAGAC
CTTTACTTTTGAGCAATGTTGGAATGGGTGCAAGACTTTGTACATACTATCAAAAATGCTCACCAGATGACCAATCTGGCTCT
TTATTACGTAACACAGATAGTAGCTTGGGGCACGTTATTTCTCTGGATCCTGCGGATAAATCTCCGTTCCTTGGAGATTTGAAA
CCTGGTTGCTCTCAGTCATCACTAGAGACAAATATGTATAGAGCACCCGTATTTCCTCATAAAGTTCCACTAACTGACTACCT
GCTGGTTCGCTCACCAAAGGGAAAGCTATCATTAAGGCGCATTGATAAAATTAATGTTGTTGGACAGCAGGTTTGTAAGTTTT
TGTTTGATACTGCGTCGTCTCTGAGAATTCCAGTGCTTTGTTGAGAATATCACAATGGAAGGAATAAAAAGTCGAATCAATAT
TTGTGATTTGTGCTAACTTGTTTCTCTAAATTACTCTTTGCAGGAGCCACTCATGGAGGTATTTTCACCAGGAAGTAAAAATC
TTCAGACTTACATGATGAACAGGCTGTTGGTACACATGTGCCGTGAATTCCAAGCAGCAGAGAAGCGGCACTTGCCTCCTCA
TATCCGTGTTGATGAATTTCTCTCACAGTTTCCTTACCAATCGGAAGCATCATTTCGTAAGAAAATCAAGGAATATGCAAATTT
ACAGGTACAATAGTGTCACAAAGCTTGCTATTACAATATGAATGCTTAACAGAATTCTTTATGGCTTGTTTGGTTTATTACAAT
GAGTTCTATTTCCATTTTTTTTTTCAAAGATATGTATAGAAACAGTAAAATAACAATTCTTTTCATTGTTTTCTGTACAGATTTT
TGAAAACAAGAAATAAAGTAAAAAAGGTGAAATTTTTTTGTTATTTACAAAAATAGGAAATGAAAAATAAAAGTAATCATTT
TCTCGAATGAAACATTTTCATTGTTTTCAAATAAAAAGAGAAAAATGAAAATTTCTGCAAAATAGTTTTCTATTGGAATTACA
ATCTCAATTTGTTATTTTATTGTTTTCTGTATTCCTGAATGTTATCTATTACTGCAGAGGGGAGCAAATGGACAGTCGATTTTGG
TTAAAAAGAGAAATTTCCGCATGTGGTCTGAGGATGAATTGAGAAAAATGGTTCTGCCGGAGCTTGTGAGTTATATCATATGA
CATTCGATTTGTTTTTATTTATAATTTAAGACATGTCTTTCTGTTGAAATGTACTATTAATAGTAAAAAAAGTCATTTAAAATTTT
TTCTGGAGGTTTGGAGGCTGTTTTCTGAAACTTAATATTTGAATTGCATGATTTGTAGACTTCTTTCTGATTCATACTATGCTTT
TGGATTGTAGAAATAATAAGGCTTCATTATGTAACAATGCATTTTATTGGATGCGCAACATTGTTATGCATTCTCACCTTGTATT
GCTTTTATGATTGGGTGGTCTATAATCTACATGTTGCTTAGCTTGGGATAACTGCATGTCAATCTCCGTTCCAAATTCGGGTGC
ATTTTGCTTTACTGATTATATATCCTGTTATCTTGCTTCATTTAATAAGATTCCTTTTTGTGTTGTATTTATCTTAAATTGTTTTAG
GTTTGTGCCTATGAAAGCATGCAAGCAGGCCTCTACCGGCTAAAACATTTAGGAATAACTGAAACACACCCTACAAATATTT
CATCTGCAATGAGTCGGCTCCCTGATGAAGCAATAGCATTGGCTGCTGCATCACACATTGAGAGAGAACTGCAGATTACTCC
TTGGAACTTGAGTAGCAATTTTGTTGCTTGTACAAGCCAGGTGATCTATGTTAAACAGTTTATTTGGCATTGGCATCTATCCTG
GCTGTGTTAAAAATTTCTACTAAATGCAATGATCTATACATGTGCACTATTTGTTTATTATTTTCTACACGAATTTGAAACTTGG
AAACATGCTATTGTTTTTCACAGAATCTTTTGAGAGGGTCGGAAAATTCTTGAGCAAAGAATATTTTGAATTAGATTTGACTA
CACTAAGTTTAGACTGAGTGAATTTTGGTCCCAAGAATTGTTTTTTCCAAGCTCATTAACTCGAGTGTGTTTAAGATGAAGCA
AAAGGTATTCTGCTATCAATTTTCTGTTACCAAGTACCTCAAATTCCCATTTTTTACTTGGTAAATTTTTTGAATTATTTTAAAC
ATTTTTTAATTCAAAACTTACCAATTAAAACATCAAATTTCACCTAGAATGAATAAAGTCTAATGAAATCCTCAAATCTCATGA
GTTTCCTTAAATTTGATCAAAATTTCACTAAATTTATGACTTATAAACCCTTAAAATGATTTCAAATCTTGAGTATATTACAAGT
TGTCAAATGTTCGATTAAAAAGTAATATAAGAATTGATCTTTAACTGAGCTTAATGTTGCCGTGTTAAGACTTTTGTTATTTGA
ATTGAAAATTTACTATTTTAGGGTTTGTGGTAGAAGAAAATTAAAGGCAGGATCAAATTTTCTTAATTTTTGAAGTGGATGAA
CTTAGTTGACTCAAGAAGATGGATTAAAATTTCTGGAGTGGTAGCTAAAGAAATAATAGAATACAATTATGTTTTGCAAAAAG
ATAAACATTTCACTTATCCCCTATATTTCCCCATCACTTATAAAAGCAAAAACTTGTTTTTCTAATTTCTTAGCTGTAAAAATTA
AAGGCAGGATCAAACTTTTCTTAATTTTTGAAGTGGATGAACTTAGTTGGCTCAAAAAGATGAGTAAAATTTCTTGAGTGGT
AGCTAAAGAAATAAAAGAATACAATTATGTTTTGCAAAAAGATAAACATTTCACTTATCCACTATATTTCTAAAAGCAAAAAC
TTGTTTTTCTAATTTCTTACCTGTAATTTATATTTTTCGACTTAATAATTGAGCCCAAAAATAGAAGTTCGCTGTAGTATGTTATA
AAGGTGACCTCTTAAACTGTGTAAGTGTTATGTTGATAAGATGGTCTTTTTGCTTCTTGATATTTGCACAGGGTGACTTGTCCT
GAAATTTTTTTTCTGCATCCTTAGGTTTCCTATAATTACTGCAATTCTTTCATTTGTTTACTGTCATATGAATTGAATAGAATTTG
AATACAGGTGAAAATATGTTATACTGGAGTTTATAAACATGCAGGGTGTAGGGATAATTTGCTTGTATTCATAAAATTAATTTTC
TATATGCTATTTATTTTACATTGGTGGGATACTTTTAGCAGGGTAAGGAAAATATTGAGCGAATGGAAATTACTGGCGTTGGTG
ATCCTTCTGGTCGAGGCATGGGTTTCAGCTATGCTCGGGCACCTCCAAAGGCACCAGTGTCTAGTGCAATGGTGAAGAAGA
AAGCAGCTGCTAACCGTGGAGGTTCCACTGTTACTGGTACAGATGCTGATCTGCGTAGATTAAGCATGGAGGCTGCGCGAGA
GGTGCGTGGTATTTTGGTGATCTGTTCTGAACTGTATATGCTTATGTAAGCTATAGAGCTTGATGTTAAAAAGGCTTTTCTTTC
TGGTAGGTGATAGTTTTTTCACATATTGTACTCAATTGTGTTATGAAGAAAGCTGATATTAATAGAGAATCTTGTTTTGTAATAA
ACTTTTTCTAGCTGCACTTCAAAAATGAAATTTGTTGCAGCAATGTTTTTTCTCGTGTTGTATTCAATTGTGCTATGAATGAAG
CTGATATTGATGGAGATATTAATGGTGAATCTTGTTTATGTACTGAACTGATAGACAAGCTGCATCTCAAAAATGAAAGTTACT
GCAGAAAAATGTTTTGATATCTGTTGGAGTTCTTACATTTATTAAAAATACGACCACATTAAGGTATTTAAGTGGAGGGTAAC
ACTTAGCTTGTAAATCGGTTTTGTAAGGTTAATGATCCAGATACAAATTTTAAGACAGTAGCAGTGTCTACGGTAGTGAATTT
TTCTATTCTTAGTAAGTATTTGAAGCCCCTCAGATTTTCTATTCTTAATGCATAGATACTGTATTTGAAGCCCCTCAAATTTGAT
CTGGTTTTTGCTATTCTAATATTTTGCTGTTGAGCGACAGCAGATAGCATCACTGCTGCCAGCGTCTTACTCCAATTCCTATTT
GGATGGATATGTGTCTTAATTATTGAAGTGTCCAGCTGCTGATCCATGATGCTTTCAAGCCAAGTCCATAATCTAACTGGAAA
AGGACTAGCTAAAAAAGTGATAATTTACATTTTCAAACCCGAGTATAACAATGAGGACAAAATAAAAATACATTTCCTAAGTA
GCTAATCTTTAGGCAGTTTATTATGTAACAATCTCCATGGGATGAATATTTAGAAGGAAGAATGACTTTTCTTCAAATTCTTTT
CCCCCTAGAAACCTGCGGTTTAGAATAATCAATATGTAAATAAGCATCTTCACTGGTTAATTCACCGCGTAGGTCTTCTTCCAT
CTCATCATATGCTAGGCATTAGCAATGAGAATCACAGTTTGTTTAATTTGTTGTACAAAATCATGTGACACTAATAAAATCCCA
ACATAACCATAGTTGCTTAAGCAGCAAATTAATTTTCCACAACTCAATTTGTCTGGTTTTGCCTTATTAGTGTCTACTTGACTT
TTGAATGCAATTATAATGATGGAATAGCGTTTTATATTTACTATGTACCATTCCTGTTTTTAATCTATGTTCTGCTCTTGTCCAGG
TTCTTCTTAAGTTCAATGTTCCTGAGGAAGTCATTACTAAACAAACCAGGTGGCATCGCATTGCTATGATACGTAAACTTTCA
AGTGAGCAAGCTGCATCTGGGGTTAAGGTTGACCCGACAACTATCAGCAAATATGCTCGTGGCCAGCGAATGTCCTTTCTTC
AGTTACAGCAGCAGACTAGAGAAAAATGCCAGGAGATTTGGGATCGACAAGTTCAGAGTCTGTCAGCTGTAAATGCTGATG
AGAACGAGAGTGATTCGGAAGGTAATAGTGATCTGGATTCTTTTGCTGGAGACCTGGAAAATTTACTTGATGCTGAGGAGTT
TGAAGAGGGAGAAGAAGGTACAAATGACTTAAAACGTGACAAGGGAGACGGTGTTAAGGGTCTTAAAATGAGAAGACGCT
CAACTTTGGCTCAGGCAGAGGAGGAAATAGAAGATGAAGCCGCTGAGGCTGCTGAGTTATGCAGGTTGCTGATGGATGGTA
TGACAATGTGTTGATCATTGAAGTATTATTACCAGATATTTTTAGCATTGAATGTGCCAAAGCTGATGTTTTTGGGGCTGTTGT
AATGCAATGCCTATAGATTGTTAATTGGATCAATATTTTGAAATTGAAATCAGGTGTACTTGTCATATTTGACCATTTTTGTTTT
GGGCGATAATAATGCAGATGATGAAGCTGATAGGAAGAAAAAGAAGAAAACTAAAGTAACTGGGGAAGAAACAAGATTGG
TATCAAAGATGCAATCAAAATTTGCCTTCGACAATGCTGAACAAGTTAAACAAATAACAAATAGTTTACAATTAGATGGAAAT
ATTCCCTTGAAAGAGGACACGATTACAGATCTTAGGGAGGTAGGATAATAATTAGTTTATTTACATTGTCCTGTCGTTTGCATC
TTTGTTTAAACTGTTATTTCACAATTTATATGAATATTATGTGAAGACTTAATTTTCTATAAATGAGTCCTAATTTTATTTCATACT
TTTGGCAGGAGGAAAATTTTGGTGCTAAAAAAAGCAAATCACTGAAGGTCAATAAAGCCAAGAAGAATGATATTGCACCTA

TTTCTCTTCCCAATAAAAAAATCAAATTGAATATGGGAGAAGGAATTAAGGTAAGTGATCAAATTTAATCTGTTTCATCACTC
TCCTTTTTCTTCCTCAAAAAACCATTTCTAGTGGTTGAGTTTATGTAAAAAGCAACAGGTATTTTCATGGCTGTAACAAAGTT
TTGACTGTTTTTTGTAGATTTTTTCTTAGTCTTTTAGTTGTTTATGTTGTAAAAATATTCTTCTCGTATTTTGAATTGTAACCCTA
ATTTGAGTGTGATTGCTGCATGTATATTCATTTAAAATTTAAATTTGTCATAAATATCTTTTCTTCTCTTGATTTTAATAACCTTG
GATCAACCTTAGGCCAGCTGAGAAAGGGAGCTTAACCATTAAGGTCAAATCCAAAGTGTACATCATAGTAATTGCATTTCTTT
CCATTGCATTATTGATAACTTAGGACTGTCCTTGTTTGCAACTATTCGAAACATTTATCAATAATTGCTAGAATGGAAGTAAAT
ACATGGAACTAGTCTTCTTGATACTTGTTCAGATTGCAATACATTATTGTGTTTTAAAGATGTGCTAAAAGCTTGTTGAACTCT
GTATGTGGTTGTCTGAGTTTTTTGTTTGGATTGTTCCAGGGAGATTATATTGCATTCTATCATCTATATTGTAATAATATTTTCAA
ATTTTACTCTGCTGCCTGAAATTATCTCTGATTTATGTATGCAGAACCAGGTGTTTAAGGAGAAAAAACCATCAAGGGAAACT
TTTGTTTGTGGAGCATGTGGTCAGGTGTGTGTATCTTGAGTTGTCTATATATGTTTCTTAGTATTCCATCTGGGAGAGTCATCCT
TATATGAAAACTGTAAACTTGTTTATCGAATATGAAATATATAAGTCTTAATTCTCAATTTAGATTTTTGTCACGTTGAATGTGT
GTGTGTGTGTGTAATGGCAGCCTCCTTTGTATATGAGTGGATGCTTCATGGTTTGGTTTACAGTCTCTTTACTCAAACTTTAAA
ATTAGTTTATTACATGTTGCTTTATGTTCGATGCTTTTAATTAAAGGTATTTTTTAAATTCACTTTTTTTTGTGGCTGTGAGGATA
AACTGTAACTAGGCAAGTGTATTTTGAAAACAGAGTGTGATCAGGAAACCAATGTAGTTCTGTATTTTCTACCTTAATAAAAT
CTAGCAATTAAAAATCCTTGATCATGTTAAAGTTTTACTCTCCACTGTTCAATATTGTGTTATAATTATTTCGTTTATGACCAGC
CTGGGCATATGCGAACAAACAAGAACTGTCCAAAATATGGTGAAGATCTTGAAACTCAACTTGAATCTGCAGATATGGAAAA
ATCATCTGGGAAACCCATATCTGTGGACCCCTCCAGTCACTCCCAGCCTAAAACCGCCTCCAAAAAGTCAAGTTCGAAAAG
CAACTCAAAGATTACTCCTGTTGACAATTCGGCAAAAATTCCACTGAAATTCAAATGTGGCTCCACAGAGAAATCTTCTGAT
AAACCTGTAACAGAGACCCTGCAGAACTCTGACAAGCCAGTCACTTCGGATTCAGAAACTGCAAAGTCTGCTAAGGTTAAT
AAGATAATTATTCCCAAAAAAGTGAAACCAGATGATACACAGGCTGAATCTCGTAAGCATGCTGTTGTTATAAGGCCTCCTAC
TGAATCAAGTAGAGGGCTTCCTGCTGATTCAGGTAGAGGGCCTCCTACTGATGCAGGCAGAGGTCAGGTTGATTATCAGAAG
TTACCAATTAAAATACGACCACCAACAGAGGAGCAAAGTCACAAAAAGATTGTTATAAGACGTACGAAGGAGGTTATTGATT
TAGAACTGGATAGTCCTGGTGGAAACACTGGACTTCAACACAGAAAGACAAAAAGAATTGTTGAATTGTCAAATTTTGAGA
AACATAAACAGGAGACCGTGTATGGAACTGGAGCTTTTCCAAAATGGAACACTAAAGAGGACAGAAGATGGTGGGAAGAG
CAAGAGAAACGAAGAAATGATGCAAGACTTAGAGAAGAAGACAGAGCAAGGAGGCATCACAAAGAAGAAATGAGGATGC
TCAAAGAGCAAGAAAGGTTAGATGAGATAAAAAGATTTGAAGAAGATATCAGAAGAGAGAGGGAGGAAGAAGAACGGCA
AAAGGCTAAGAAGAAAAAGAAGAAGAAAAAGCCTGATTTAAGAGATGAGTATTTAGATGATCCCAGAGCAAGAAGACATG
ATAAGAGAATGCCTGAAAGAGACAGGAGTGGAAAAAGGAGATCTGTTGCTGAGTTAGGAAAGCTTAGTGCAGATTATATGC
CGCCAACAAAACGCCGAAGAGGGGGAGGGGGAGAGGTACATTTCTATGTTTGTTACTTCTTTTATTTAGTGGATATTTTATTT
TCATGTATTGCATCTTCATACGTATTAAGTAGAAAGCAAATTAATAATAGATTGTCATTTGTGAATAAAATGTGCCCAGAAGGA
AGAATGTCATTTGTGTTTAAGATTTAAGTAAAACTGTTATATGATTCTGGTAACAAATAGTTCTGAAGTACTCGGTATGTCTGC
GGTGGCAATCTTTGTTACTATGCTTTTGTAGCGATGTTAGTTTGTTTCACATGGCTTGACTTGTATGTTTAATTCAATTTCTGTC
TAAAGGTTGGTTTGGCAAATATCTTGGAGGGCATTGTGGAAACGATGGTTAAAGATAGGTACGAGCTATCGTATCTTTTCGTG
AAACCAGTGTCGAAGAAAGAGGCTCCTGACTACCTGGACATTATAGATACGCCTATGGATCTTTCCAGAATCAGGGAGAGAG
TACGGAATATGGAGTACAAGAGCCGAGAAGATTTCAGGCATGATGTTTGGCAGATTACTTTTAATGCACACAAATACAACGA
CGGGAGAAATCCTGGAATTCCTCCCCTTGCAGATATGCTTTTGGAATATTGTGATTATTTGTTGAATGAGAATGATGATAGCCT
CACTTCAGCAGAAGCTGGCATTGAAACTAGAGATTCCTAAACCATGTCAAACTCAGATCATTAGTCTGGAGGTTTTGCTAGC
GTTAAATCATTTTCTAAATATAGTGTAAATTCTGATTTATAAGGATATACCAAAATGCCTCCAGTATTTTTTTAACGATATCCAA
AGAGGGTTGAAAGTTGTGTGGAAACAAGGATGATGTATCAACCATGTAGCTTGTTAAATTTTCAGCGTGTAACAATGTAGGT
TGAGGCCACTTAAAGGATGATTTTAGATGTTAAAAGAACAAAATAAAAGCAGAATAGCAGCAGTTATGGAC

>VrTAF2 LOC106768217

ATTTCTCTATAAATAAAGCATCACTTCAGCTATCTCGGGGGGAGACACACTCGAAGAGAGAGAAAGATACCTCGATTCGAAT
TCGATTTTCGTTTCTCACTCTTCCCCGTATGGCGAAACCTCGCAAGACCAAGAACAACGAAGACCCGAAGCCCGAAAACTC
TGGTGCCCTAGTTCACCACCAGAAGCTATGTCTCTCCATTGACATCGACAAACGCCTAGTCCACGGGTTCGCAATTATCTTTT
CCCTTCCATTATTCCTTAATTAGCATCTCTACTACACGCCGTTGTTGTGGATTAGGTTTTGTTACGGCCGAGACCGTGTTCGAG
ATCGTGTATTTAAAATCGATCTAGAATTTTATTTGAGTTGATTTCACTTGTAGGTACACTGAATTGGAAATTGCGGTGCCGGAG
ATTGGGATCGTAGGGTTGCACGCGGAGAATTTAGGGATTGAGAGTGTTTGGGTCGATGGTGAGCCCACGGAGTTCGAGTATT
ACCCGCATCAGCAGCAGCAGGTGGAAGATGATAAGAGGTTTAGTTCTGTGTGTTCTCCCAGCTCTGCTGCCGATGCTGCTGT
CTCAGTGTACATGTCCTCTCTGGAGAAGGAGCTGGTGCCCAATTTACTTATTAACTGCTGTAAACCTTCCAAGACTGAAAGT
GAACAACAGCAGGAGCAAACAGTTCCCGAGAACGGGTTTCACTCCACGGCCGAGCCCAAACAGGTTATAGTTGCCGTCTAT
AAACTCTAGGTTTCAGCGCTAACTCCTCGCTGAGCAATTTAGAGAGTTATTTTAAACGAGGCCTGTAAACCCTAGGCATAGC
AACTGAATTGTACATAATATAGCAAATAAGTAATTTAGTCCCTGAAAATGTTATTAGTTCTCACTTTATTCTTAGATTTAGTGTA
AAAAAAAAGATCCCTGCTACTGTGATATTTTACCACACACTGACATTTTGCCTTTTTATTTATGTTAATATCATCACCTAAGTTT
TTACGTTACAGAAACCTTGTACCATCTGAGGGACATACCGCGGTTTCAAGAGCTTTTTAATTATTTTTAGTTCTAGGAACAGG
AGTGACAGTGATTAGCAAATCACAACGGGGGCTAAATTGTGTATATGCTTATAAACATTGTGTTCATACCTGTAATTATTGTTT
ATTGAAAGTAATTAGGCACCGACTGGATTTTATAATTATTTATATAGATGTATTTACTGAATTGAATTTTATATTGTATTTATATAT
TTATTTACGTATGGGTCTTGAAAAGTGCCTAAGGGCACTGTTTAAGGAATTGAAAAGAAAAACTTTTTATTCAAAATCATATT
GGAGCACATTAAAAAGTAATAGGGAAGCACATTTCTTCACATCGAAATAGTAAACTTACCTCTTTTAAGTCCTTATTCTACGC
TCTTAGGGTGCTGGTTAGCATGTCATTTATATATTATATAATATATTTAATGATAATATACTTGTATTTTTTAATAGACTTCCTGCCA
TGGGTTTGGGTTCAGTATGACCAACCTGAAGTTAAATTGTGGTTATGGTTATCCTTTACATTCCGTGAAATAGTGGACTATTTT
AGCAACAATTGTGGTCATGAAGAGTCCAAATCCAAAAGCCTTGATGTTGAGATCACATCCTTCACATTATGGGAAATTGCAG
ACTGATGCAGTTGCAATGCTGTTATGAGTTAGAAAACAATTCCATTGCTGGTGAAGCTACCGACCATTTTATGAAACTTTGGA
CCCATGTTCATTCTAAGGACTATCAACCCATTTATGAACATCGTCCTCTGCCTGCTTGGACTTTCCTCGTGTTATTTAGATGTTG
CACTAATGAAATTGTGATGTGTCACCTCAAGTTCTATTTTATTGTGTCCTTTCTAAATTTTCACCTTATTTTTGTATTTTTATTTC
TATATATTTTTGGCCCACTCATAGTAGGAAATAGGTTATTTTTTTATGGTTGAGATAGTGCAGCATTCTTCCATGAGAATATGGC
AGTGAAATGTGAGAATGAGTTGTTTTGGTGAGGTTTACTATTGGTGTTTTCTCATGTAGGTTGATGTTGGGTGCAGAATGTGA
GAATTGTTCGTATTGACTATTGGATAGAAAAGGCAGAGACAGGAATCCACTTCAGAAATAATCTTCTTCATACTGATAATCAG
ATAAGAAGAGCTAGATGTTGGTTCCCCTGTATAGATGACAATTCACAACGATGCTGGTATGGACTGTTAACACTTTCCCATTT
GTGTGTCCATATCCATGCTTACATTTCATTATAAATTTATTTACTGTTCATTGTTTGAGACTGAAGTAATTATTGTTGTTTTGCTA
ATGTTCAGCTATGACCTGGAGTTCACTGTGGCACACAATCTTGTGGCTGTTAGTACGGGATTCTTGCTTTATCAGGTAGCAAC
GAGTGATACTATTACTAATATCCTGTTATTTAAGTTTTTAAAGCACAAACAATACTATTTTTACATAAGCTGATAACATTTTGGA
GCTGTTTCTATTGTTGATAAGCATATTTAATGTTCTTTCAAGTCAAGTCAGGTTGGAAATTGGAATTTCCTTTGTTTCGGAAAT
TGGAGTTTTGCCTACTTTGGTTTTTCTTTTCTTATATTCCAGAGGTGTTAGATGATGATTGGCTTCAAAGTCAATCAATATTCTT
TTTGGGAATATTCATTGCTGAAAATGAGTGACTGCTATGATAGAACTGTAGAAAACTAAAAATCACCAGTTTCTACATAAACC
ATGAATGTTAAAGTTATTGGTTATCTAAAAAGTATTGGAAATAGTGGTTTTCTATACATGATTAGTGTTTTGCTGCCAAATAATT
TAGCTTGAGATGTCTGGTAGAACAATGAATTATCTGCTGATTTTGATTTTATGCGCTGTGTATTTTCATTTTCTAGTTCATTTATT
CATAATTTGTTCATTGGACCCTTGATATACCTTAAAGGAAGAATCTTCTACAATGTTGAATTTGATCTGCTTATCCTTAAGTTAT
GTTCTTGCATATTGTGAGGCCCAGTGAAACTTTTGGAGATTGAGCTCGCTATTCTGTTTTTTCCCTTTCCTCTTTGGATCGGAT
GCAACCTTCCCCTTTTATGTTCCCTGTTTCTTGTCTTCTTTTTCTTAAATGATAAAATTCATCTGCAAAATACTCTTGTTGTCAT

TGGGACACAACATCAACATAGTGATATGGTTTGGATTTCATTTTGTTGCTGATGCCTGCCTTTTGGATTTAATCCCTGTTAGCC
ATATATGGTTATCACTGTCAATTTGTTGTGAGGGGGTGTGGGGTTAATGCAACTCTGCAACACTATAGACTGGATATTGGTTTC
TGGTTGGTGGCATCTTTGATTTGCGGTGATAGCTTAGTTGGGAGAGCATCAGATTGAAGATCTGAAGGTCGCATGTTCAATCC
ATGCTCACCGCACCTCCAGGGTCATGGATCTGTTTTGTCAAAGTTCTCATCCAAGGAATTTGTCACGTCTTGTTTAGATCTTT
GGGTTGAAAAGTTCATTTAATTTTGTACAAAATTTAGAAGCAGTGTTGCATTTTGTCTTCTGCTACCATTTCATGGAAGGGAA
GCATAAAGGAAAAATACCCTTTGTCTAAGTTGAAAAACATAATTATTGTTATTATTATTGACACATTCCTTTACTTGATTCCATT
CTCTACATGCAATGGAATGTAGATTATATATGATACCCTGTCTGCTGGCCACCCACCCTCATTTCAGGGAAATTTCTTGACCCT
CTCCTTTGTTGTAATGCTTCTCTTTTGTCTCTATTTAAATTTATAGTTCTATATGAAAATTTATCTTGTTACGCATAGTTAAATATG
TTTTTGTTTGAACCTCCTTTCTTTTTCAGGTCTTAAGCAAGGACAATCCTCCTAGGAAAACATATGTCTATAAATTGGATGTTC
CAGTAGCTGCAAGGTGGATATCTTTGGCTGTTGCCCCATTTGAAATTTTTCCTGATCACCAATTTAGTCTCATATCACACATGT
GCTTGATGCCTAATCTGTCAAAGATGCGGAATACAGTGGAATTTTTCCACAGTGCCTTCAGGTTTGACATGTGACAGTTGCAA
TTTAACTAGCAGGTTGCTTTTTAATCACAAATTGTATAATGATTGGATAGTTGGACTTTTATTTGTGAGCAGCTGCTATAAGGA
TTATCTTGCTGTAGACTTCCCATTTGACTCATACACGCAAGTTTTCATAGAGCCAGAGATGGCTGTGTCTTCACTGAGTTTAG
GAGCCTCTATGAGTATATTTAGTTCACAAGTTTTGTTTGATGAGAAGGTTATTGATCAGGTCAGTTCATTACTTCAATTCTTTTA
TTTGATTTCCTTTTTTCTAATCATACTCTTTCTGAGTGAATAAAGAAATTTTTCCTCTTGTCCTTGAGGGTTTTTATGCTTGTGA
ATTTACTACCATGCCAGCTTATGATCAGCCTATGTCTTTATTGACAGACTATTGACACAAGGGCAAAACTTGCATATGCTCTTG
CAAGACAGTGGTTTGGGGTGTATATCACTCCCGAGACACCAAATGATGGTAACTTATCAGAGGATATTTGGATTGCTTTTTTA
ATTTGAGTTGATTCTTTGGGAATTCAGCAAATTTTGTCTTTCCTCTGGTGTGATTGATGAATACTTATTTGCACATTGCAGAGT
GGCTCTTGGATGGACTTGCTGGCTTCTTGACAGATTTTTATATCAAGAAACATTTGGGAAATAATGAGGCACGATACAGGAGA
TACAAGGTTTGTTTTTCTTTTTGGTTTAATCTGGTCTATGTGAAGGCTAACTCATTGTGCATTATATTGTAAGTTAATGAACGTG
GGCTCTTTATGGGTGTATATAGAAATTTGAGGTTCAACTTGTTATTGTGGTTTGGATGCTTTGTAGTTTGAAGGTGTTCATGTG
TTCTCTTAAGTATTCTTGAAGATGGTGCTGTTTATAACCATTTTTTTTATCCCCTAGTTGTTTTTATTTTTCCTTCTCTACAGATT
TGTTTTAAGAGTTTCTCTATTGTGAATTGTATTTAATATTTAATCACATGTAAATACTTTATTCATAACCATTCAAAGTATACTATA
AGGAAATCGTTGTAATGGCTAATAATGGAGAATATCCCTAACTATTAGCGGTATATATTTCCCTAATTACTAAGATAAAAACATC
TTTTGTACCCACCTCAAGCTCGAACATATATGTCATATGAGCCTAGCTTATTTCAAATGTGGTCAACCAGAGCTCTTATGGGAT
TTTGCAAGGGTGTATGCAAGTGGGTCATGTAAGATGGTGGTGCTTATGCTGGAGGAGCGATTGGAGCACAAGATGATGCTAG
AGGATCATGGAGGTGCCACTTCTGGTGGTGCTTATGTCTGTCGATGGTATATAAGAAGTGAAGGTGGAGTAGGAGCTAGTTC
TAGGACAATAAGTGGAACCTATAGGGGGACGCCTTCAATGGTGGAAGTAAAGAAAGTAGTTGCTCAAACGTAGAGACTAGA
TACTGTTGTGGTTGAGGAGGGAAATATCAATAACCTTTCTGGTGTACGGAGTAGCCCAAAAAAGTATATTTTGGGATCGCAG
AGGCCACACGGACAGTGTGTGTGATCAGTGCAATGGAGGAAACGGAAAAGGAAAATCTAGCTCTTTGATACCATTAAGAGT
TTCTCTGTTGTGAATTGTGTGTTTAATCACACATGTAATCCTTTATTATAATCATGGAAAGTATAACAGAGGGAAACCTAATAA
TGGCTCATCATGGAAAATATCCCTAATTATCAGCTGAACATATTTCCCTAATTACTAAGGTCAAAACAATTTTTAACAATATTAA
TTATTTTTAGCTACTTAGTTCAAAATTTCAAGGTATTCTTCTTTTTTGTTTTGGTTTTCTTTACTAAAATTTTATTTCATATTTAAG
CATGGTGGTTGATCTGTCACTCTAGGTTGAGGCTGTAGAGATATTCTGAGTTGAAAGTGCAAGAGTTAATATGTTAATTACAT
TTTTTTTACATGCTAATCTAAATCACTCTACAGGCAAATTGTGCTGTTTGCAAAGTTGATAATGGTGGAGCGACAGCTTTGAG
CTGTTCAGCTTCCTGCAAGGATTTATATGGAACTCAGTGTATTGGTTTGTATGGAAAAATAAGATCGTGGAAGTCTGTAAGTA
TTTTCCTTTCCTTTTCTTCTCCCCCTAGCTTTCATTTCATAGCATTGAGCCAGTACATTAGTGTGATTCCTTAATCAGTATTGAAT
TATATAAATATTATTTTACCCCTTTTCAGGTGGCTGTTCTTCAGATGTTGGAAAAGCAAATGGGTCCTGAATCTTTCCGTAGAG
TGAGAGTCCTTGATATTTGCACATTTATGCTTCTGATCATTCTAGATATATGCTGAATGTCTTAATCATGGTAGTCAATAGTGTG
CTTTCACACCAATATTGTGCTGGTGTGGAGTGGCTCCCCCTGCACCAAAAGGTTTCTTGATGTCGCACCTCAGGATTCTGGCT
GTTGTGTGCGTTGTGAGGCCAAAACTGCAACATATTTCTCCTGCTGACACAATCTAGGTTTAACATAAGGGATTCAATTCCTG
GCTGGAAACAGGGTCGTTTGGGGGTTTCTGTTTATTTTTGCTGACAATAAAAGGGGAACTTTCTATTTTAACCTATAATACAC
AATTTGTACCCCAGTCTTCCATAGACCATCCTTCTCTCCCACATTCTGGTTCTCTACTTTCCTCCTGTCTTGCAGCATCTTTCCT
CTCCATATCTGGCAGGATTTCCAATGAGGTTTCTGGGGGAGATACCAACCTAATCTGGAACTTCTGATTAAAACTGTACAATT
CTTTATTCTTTCTTTTCTGTTTAGCTGCAATGGCTTCATGCGACCAATCACCCTCTGCCACTCAATCTGTTAGATAGTGACGGA
TGCCAAATAATAGTTGTGGAATCTTGTGTAGCAATGTTCAGGCATGTTTTCTAGAATGTACTTCCCCGGAGATATTTAGTAGTC
TCCTTGCATTGAACACCTTTTCCCATTGTTCATCATCTGCCAATAGCCTTTTACTGTATTTTTTGTTTTTCTTACCATTTTTTATG
AAATTTTCTTTTTCTTTATTTCCCCTCCAAAGTTATATATGCTTGAAGGTTTGCTGATATTTTTTTTCTTGCTATTAATGTATATAG
ATTTTACAAACGATAGTTTCTCGGGCTCAAGATAAAACACGTTCCATGAAGACTCTAAGTACTAAGGAGGTAATGTGCTTTTG
GTTCTGTACAATATGTGGCATATTAATGGAATGTAGCTTCTGAGACATTGGCAGTTAACTATGCATAGTTGGCCGAGTTCAATA
AATGTTATAGTGTGTCATAACCACTTCTTTTAGGTTTATCTATCTATAACATTGCATTATCATGAATATGACTGCTATAGGATAAG
TAGATTGTGCCAAATTGACGCAAATGGGAAAAAGGGAGGATAGGAGCATGGGACATAATTATTGTCAGTGGACGATGGAATG
AAAAATGTCTAACATTGGACTAGTTTATCGGAAGAGTTTCTACCATTTAGAAAACTTCAAAGAGTTAGAATTCTTACGATTTC
AAGTTTTTTTTTTTTTTTTTAAATTGTCAGCCATGAGTGGGAGTGCGGGAATTTTCTTTTGTTTAACACATAAGACACAAAGA
AATGCTTTTAGAGAAGCAAGCACTGAAGAATTTAAAGTTGTCCGCTTCAGTCAGTATTTTGGCCTCCTTTTATTTCAACTATTT
TATTTTTCTTTACTTGCTCCTTTCATCTTCTTATAGTTCCCTCCAACTGCTCTTGACTCTCCCTAAATAATTTTCTTTTGTTTTCT
GTCCTAGCAGTTTGAAATGATAGATTTTTTGTATTTTCACTAATTTGCCACATCTTAAACTCGGATGCTCCTTTCTTTTTTTCCG
CCGAGAGGAGTGTTGAATCTTCGGTTGGGGAAAAATTGGCCAACAGTGAGTCAGTACCATAATTAAGCCTGCAACATTTATT
CATTTCATTGACCAAGCTTCATTGTTATGTGACTTGCCCATCAATATTCCCTCCTATCAGTTTCCAATCATCTAGATTGTTCGGC
TATGGGTATTTTTAAGATTTACAAATTTTTCCAAATTTTCATTTTACTAAATTTTCATTAATATGATTCCAATTTTCTGCTCTTTTC
AATTTCAGTTGTGAACCAATACTTTATTTTCTGAAAAAGACATTGTTTGGATATATAGAATAATTAAGTTCAATAATAAGTTAA
AGAGTAATGGGCAGTTTGACCACAAACTATTTGCTACAGAAACAACTATCATATCTCAACGGGAGTGGCAGACTAGGCATTA
CGCATTTTACGTATGCTTTATAACTCAGCTTGTATGCAAACTCCATTTTTGAGTATATCTGCCCATCACGGGCCTTTCCATGTTG
CTCTATCTTATGCGTTTAATTGATCAAACAATATGCATAAAAGGTGGACAAGCAAATTGATGAAATTTGCTCTGAATTGCTTTG
GTAGACTAAATAATTTTGTCCTTGTAATCTCATGTAGAGCTGAGAAGTATAAATGTAGTTTAATCTTCAATGCTGTGATAGCTG
AAGTATTTTTACAGTTTTTGCTTCTGATTACATAAGGTGGATCTGATAAATTTTTTCAATACACAGTTTCGACATTTTGCCAATA
AGGTTGGGAATCTTGAACGTCCATTTCTTAAGGATTTCTTTCCACGGTGGGTTGGTTCTTGTGGGTGTCCTGTTTTAAGGTAT
AAGAAAGGATTTATTTGTTATTCTTGATTTCATTTTTGGAACCCTATCTGTACATGTATTGTGATGGGTTACCTGTTGCTGTTGC
TAAGGATGGGGTTTTCCTATAACAAAAGGAAGAATATGGTTGAATTGGCTGTGTTGCGAGGATGTACAGCATTGCAGACTTC
AACTACATCCACTCTTGATATTAATCCAGAGACAGAAAATAGAGATGGAGATACTGGATGGCCTGGTATGATGAGCATCAGGG
TATATGAACTTGATGGTATGTATGACCATCCTATTCTGCCAATGGCTGGAGAAGCATGGCAACTGTTGGAAATACAATGCCAC
TCAAAGCTTGCTGCTAGACGCTTTCAGAAGCCCAAGAAAGGGTTAAAACATGATGGATCTGATGATAATGGTGACGTGCCTT
CCATGGATGTGCGCTCAAAGTATTTATCAGTTTCATATCTTTTTTGTTTTAATTTGTGGTTATGATTTAGTTTCTGACGATTTTTC
TGTTATGGCAGTACTGAGTCCCCTTTATTATGGATCAGAGCAGATCCTGATATGGAGTACCTTGCCGAGGTTCACTTTAACCA
ACCTGTTCAGATGTGGGTAAGGATGAATTGTTACAGCCAATTGATTTTATTTGGTTTATTGTGATATAGTATTTTGCTATGCTATT
AGAAGCACCCCAGGTTTCAATCATTTTGGTCTTCTTTCTGGGTTTTATGCGTGATGTGTATAGAACTAGTTATTTATCCTATGGT
AAGAAATCCTAGGAGAACCACTTAAGAAAAGCTAACCGTTGTAATTGACAAGCCTAGGGCCTCAACCCACGCTAGGAGTCT
TTTTTAAATGTGGAACTCATTAGTCATACCTTGCAACTGAACACTAACATCCTACCATGCTCCACTGCCTTGACACACATATAA
GCTGGCTGTGGAGTAGTCCTTATCCCATTCAGTGTCCTTGTTTTGCATCTCATAGTGAAAAGCATTGACCAATTGAGGGTTTAT
AAATCCAAACACCAAAAGTCATACTTCTGGTGTGGAACTCAAATTCCATACCTTGCTATTTGATCTTAACATCATACCCTGATT

GCAGATTTTTAATCTTTGTTGTCTTATATTAAAGTGAATGTTCAAATACTTTCCTTTTCTGGTCTTTTAGCATTTGTGGTGTTTG
AATAGTTGATATGAAGTTCTTTGAAAATTAAAATCGAGTGTCTTCCTGAATCATCTCTCTTTGTTTAAGTTCCTTTCTAAGTTTT
ACATCATGCCCTTTTTTATTCTATTAGGGAAGCTAGAAGACCCAATTACATAAAACGTGCAATGATGCAATGTTATGTAATCAT
ACTCATTTACCTCTTGAGCCTTCCTCATTCTCTCGGCTATTTTAGAAATAAATTATGCTCATTCATTTGTGTGGGGTTCGTATTT
GCATCTTTCATTATCATATTTAGTATTTTGTTGGTTATGTTTATTGATTATAGATATTTAATTTTTTATTTAGATCAATCAATTAGAG
AAGGACAAAGATGTTATTGCTCAGGCACAAGCAATTGCAGCTCTTGAGGCATCACCACAACTATCATTTTCTATTGTAAATGC
CCTAAACAATTTTCTCGGTGACTCCAAGGTGATTTTCTTTCTGTAATATATTTTCAGTAGTAGCTTTTAAATATGAAATTAATAT
CTGTAGTCTTTTGGTTCTCTAGGCCTTTTGGAGAGTACGGATTGAAGCGGCATTTGCATTGGCAAATTCAGCATCTGAGGTAT
TCAAATGCTACGTTACTTGTTGCATACTTTATGATATACCTCCTCCTGTCTTGAATATAAGCAAACAAAACATAAAATCTCTTAT
CTATTGGTCTCAAATATTAGGAGTATTAACTAAGTTTAACTTATAAGATTGTTTCCAAAATACCTTCATTTAGTGGAAGTTGGAT
TTCCAATGACTCATTTTTTTGTATTAATTTCCAAGGAAGGCTGGTTTAAGAAAGAAGTTTATGTTTAAAATGAGTTCGGTACAA
TTAATTAATGCTAACTCTTAATGAATGAGTTAGGTACAATTTAAGCCTATCACAACCCTACAAAATTGGGCTTGTAGGGTGATG
TTTACACTTATTTATATATTGTAAAATGTATTTATCTCTCGTCGATATGTGATCTCTAATATACCCTGGTCACACTGTGGCATCTTT
ATATTTCAAGAGTGAAACTAAATATTTGTGGGTGTTCAGATAGTGACTGAAAATGGGTGACACAGTAGTGCAACATACAACA
AACTTTATTAGGATAGACTCTCTTACTATCTTAAGAATTGACTCAATCTTACAAAACTAGCTCTTTAGGTGAAGTTTGCATCCA
CTTGCATATTGTAAATTCTCCTTATCTTTAGTTGGGATCTCCAACAACTAACAAACTTTCTTAATTAGTATGAGTTAGTTATTTT
TCGTTTATATTCAAGACCAGTGGTATGCATATTAAATGAATGGCTGAGTGGGAATGGAGTTGGCTGTCCTTAATTTAACCAAAT
GATTTCAGATCTGGTTTTGAGATAATAGATGAACTTTAATAACTTGCTAATCACTGCTGTAGTAGATATCTGTACACAGGAAAA
AAATGGAAGTTTGCCTTTAAGTTTTAAGCATGCCTTAATCTGGTGGCTGTTACAACTTTGAAATCCATTTAACAGTTGTTCATG
TTCAGGAATAACTGGGATGCCATATATATGACTACTATTTGTTGCATATGTATCACTGTAGATAATGGGTGTAATAATTTGAAAT
GTGAATAATGTTGTCATTTAATTGTCTGACAGGAAACTGATTTTTCTGGTCTACTCCATTTGGTGAAATTTTATAAGAGTAGAA
GGTTTGATCCTGACATTGGACTCCCAAAGTAAGAATATGGAAACTATTGTTCACCTTTTCTCATTTAACTTTGGCAATATCCAT
TGACAAATTTTGTTGTATTATTATTTCCAGGCCGAATGATTTTCATGATTTTGCTGAGTATTTTGTTCTTGAGGTAATGCAAGGA
GGTAGGATGTGTAAGTTACGCCTGATGTGATGTGATTTTGTAATGTTTGTTATTTATTGTGTTTTGAATATTTGTAGTGTTCCAT
AAAGTGTTGTTTCAGTTTTTATAATTTCTAATACAGTTTATTTTTTATTTATTTTATTTTTTTTTGCGAATATGTGGTTATTATATTT
GCATGTGTCAGTATCTTCCTTTTTGATCTAGTGTAATATATTTGTCTGATCTCGATGATACATGCCAGGTTATTGAACTTTGGTA
GCTGTGCTCTGTAGGCAGTTTTACATTGCTTATTGAAATGTAAGCAGTGGTCAGTGTGGTGTGCTTAGCTGTATTCATTTCCTT
AATTATCGTGATGCATGAAATGACACCAGATATGTACTGTATAAAGGTTTAAGTAGGAGTGAATGTGTCTATGTTAGTTGTACC
TGTAAACCCAAATGATTAAACCTTTTCAGTCTGATTCCATGGTTAGTGAAAAATGGAATACAAAACAGTAGTCTCTGGGTTTC
ATTTGGAGACAATGATCATTCTGAAAGAATAAATGATCGCTTTGGGAATTATAACATTTTCTGAACTTGTATAATTATTTGGGG
AGTGATATTTTGAGTCTCAATGCAAAGGACACAAACTTCTCACAATGACTTTTGTGGTTCTGATATGTCTTTGTTACCTAGGAT
GCTTCTAACCTAGTACAAATTACTTTCAGTGTGCTCTATTTTGATTTCTTCTACTGTCTAGTATTATGGTGTTAAACTTGTGTGC
ATTATGAATTAAATAAGTTCAAACTTACAATTTGTGTTGTCATCATCTGGTGATCCTATGGCCTTTTCTATTGCAATCTTGAAGC
TTGATCTTTGTTAACATGCTCGCTCCACCCTCATGAACTTTGCTGCTCTTGTGTTAATTATAATGCCATCCATGAACTAAGATTC
CCATATCCCTTTTACAAACTATAATATCATCCATGAAATACCCTCTTTTGAAATTATAATGTTATCCATGAACTCTATCCTTATTT
GTGATCATTTACATTCTTTTCTTTTACAATGGTTTATTGTGTTTTACAGTTGAATATTGAATGTCTTGTTTATGGTATGTTTTTGT
CAGTTACCTTTTGTGATAGTAATTCTGAACTAGTTTGCATATTCTTCTCGTGGCTAGATAATGAATAATTGTTTATTAATGTGCTT
TTAAAACTTGGCAAGTCATTTTTGGGTAACTAAATTGCTGTTGTAGGCTATTCCACATGCTGTTGCCATGGTTAGAGCCGCTG
ACAAGAAAAGCCCAAGAGAGGCTATCGAGTTTGTTCTACAATTATTGAAGGTTATTGTTTATGTTAGAATCTGTTTTATTAGCT
GTATTATTTCTGTCAGGATTTGTGTTTTATTGTTTCCTTATTTAGCAGATTATAATTACAGTATATGATGGGGATATCCTTGATTCA
TAGTGAATCATAGGGATCTGGTTGTCTAGGAGCTATTATTCTACTTCTTAAAATAGCTTTCTAGCCTTATATATATATAGATTGTATT
CTCAGCATAATAAGATGAACCGTTCTACCCTAAATTATAACATGGTATCAGAGATCTCAAATTTTGGGTGCTGTCTTTGAATGC
GCACACCAAAACCCTAGACAACAGCCTTCATTTCTACCACCATCCCTTTCAGGACAGCCTTCATTGCTTTCGCACACCAAAC
CCTAGCCGCCAACCTCCATTGCTGTCAATCGTTTTGTTCGGGTCCCCTTCATTCATTGCCTTAAGGTTGTATTGCATGGACATG
ACAGACCAAATTCTTGAAGTGGTGAATACCGTGTCACAACACATTCAGGAATAACTGTAGAACCTCGACACTGCCTACTGAT
TGAATGGAAAGAATTATTTGAAATGGGCCTAGCTTGTTTGCAGAATTTTGAAAGGTAAAGGGAAAACTAATCACTTGATTGA
AGATGCTCCTACAGAAGAAGATCCAAGATTTACAAAATGGGATGAAGAAGACTCTATTATCATGGTATGGCTATGGAACTCCA
TGATCCCAGAAATCACTAATATTTGCATGTTTCTTAATTCTGCAAAGGAGATTTGGAATGTCATAGAGCAGAGTTACTCCAAT
GCTAAAGATGTTGCTCAAATATTTGATGTGAAGGTGAAAGCTGATGCTGCAAAACAGGGAAAAAAGACTATTACTGAATATG
CCAATCAACTAAAAAATTCTTGCTCCAATCTGACCTAGCTGGCGAGTGATCCCATTTCGCGGCATAGAATGACAGCAAGCGA
GTGTTATTGAATATCTATTGTAGTCAAGTTTGTTTGGACTTATCGTGTCATCTCCTATCAAGTTCTTTCCTAGTCTCACACTTTG
ATGTCTAGTTCTTGGCATGAAGGGGTGTATTAGGGTATGACCAAATTATAATATATATATATATATATATATATATATATATATATA
TATATATATATATATATATATATATAAGGGAGTGTAAATCTTATCTTCCCAAGATGACTTTGTTGGGTTGTTAGGTTTAAATTCTAC
TTTCTTAGGAACCGTACTTGAATTAGTTAGAGGACATTGTAGTTTTTGGAGACAATAGATGAATGACAGCTAGTTTTTTGTGA
TTCTGAAAAAAAAAAATGGAATTTAGAACTGTGACCTTTGGAACTGAAAAATCACTAATTTTTCAAGTGCCCCCAAACCTCAG
CATTTTCTTCTGTGAAACCTTACTCAGGCTGAAACTAGAGGTTTCTCCTTTAGTGATCATCGCTGCCTTCCTTTTCTGATTCTT
TCTACCAGTTTTACATTCTCTTCTGCCTTCATTTTTTTTTTCTGTACTGTTTTTTAAATCCATTTATTTATAAATGCTTTTCACATG
GCTGTCACGCTTTCACCATTTGCCGCAATGCCATCCTCTATTATTCTATGGCTATTTTTGCTGGCCAGTTTTATCCTTATTTAGTA
TTTAGGATTCAGCTACCCAAATCAAGTGTAACGACTGGTTATATCTGTTTTTTGCTCCTTTGGGCTCTTGCAATATTGTACTATT
TATTACTCATTGCATACACCACTACTCTATGGATACAAGATATGTTGTTTGGGCAGTGTGATTACTTTTATTGGGATGTTTTCTT
GCTCTAGTAATGTAGAAGTTCCATCATTAGCTTCAGGGCTCATGTATTTATCCTCATATTTTTCCAGTACAATGACAACAATGG
GAATCCCTACTCAGATGTCTTTTGGCTTTCTGCATTAGTCCAATCGGTTGGTGAGCTTGAGTTTGGGCAACAGGTTTATAAAC
TATTTTTCTCTTGCTGATTATCTTGGTTACCTTTACAGATTAATAATTTAATATACTGATCTGAGCTTCCTGAGCTTGTTTTGTTT
TATACTTAACAAAGCTCCTTAAGTACCTCTATTAATTAAAATTTGAGCTGTTGAATACGAAGTACTGATAGTTAATAATTTGTTG
GTGGAAAGTGTGATTTTTGAGTTTTTGCAATTTCCATTTCTATTACAACTATGCATATGTTGCTTGTTATGAAAAGTGTTTGTGT
GTGTATTTAACAGCTGTTAAATAATATTTGCAACTGATATGGTTCTTGCTTTCTTCAAGATAATCTTAACTGTCAGCATCCTGAT
TTGAGTATTAGGCTTCTTTCTGACTCAAACTTTTGACAGAGTATTCTGTTGTTGTCGTCACTTCTCAAACGCATTGACCGGCT
TCTACAATTTGATAGGTGAAATGATGCATTGATTACTGTGGTGGATTTATCAATTTTACTTGCAACTATAATTTACATTTTTGAA
ATGTCTTCTGCTAGTCTCATGCCAAGATATAATGGAATCTTGACTATCAGTTGTATCCGAACATTGACCCAGATTGCTTTAAAG
CTTTCGGGTTTCATTCCTCTTGTAAGCACCTTGTTATTGAGCATCAAACTTCTTTTCCATGTTTGGTACAAAGATTCTAAAAGC
TTCATCTTTACTACAGGATCGAGTTTTTGAACTTGTAAAGCCTTTTCGTGACTTGAAGACACTGTGGCAAGTTCGAATTGAAG
CAAGCAGAGCACTCCTTGACCTTGAATTCCACTGCAAGGGCATGGATTCAGCATTGCTTTTATTTATCAAGTACTTAGAGGAG
GAGCATTCCTTAAGAGGTTGTTCATCTTTTCCCTCGCGGAGGATCTATAGATGTATGTCAAACTAAGAAATTGGTTCAGAATG
CTAAAAGTAAAGTATTTATAAAAATGTTTATTAAGTGGGACAAATTTCATAAGATACACGGTTGGATGAGTTTCTCCTTAAGTT
CGTTTTAGGAAGATAAAAATGTCCATAAATTAAAGTTAAAAATTTGCTGAAACACTATAATTTCTCCAATTTTTTAATTTAACTT
ATGCTTAAACTAATTTTAGCTTTCAAAGAAGTTCAAATTCATTTTTTGTTCTTTGTTTTTCTTCCTTAAAATAGGTTTATTGAGA
AATTCGTCCAAACTAGTTTTGATCCTCCAATCCCAAAACAAGTTTAAATATACAATTTTATATATTTTAACTCATCTTGTGGCAC
ATAATTATCTTGTTTTTCAAATATTGACAACCTTCTGAAACAATCAAACCTGTATCTATTTTTTCCTTTCATTTTCATATTTACTT
TCCCCTTTCTTCCATCTTAATTTACCCCTTTTTATGTAATAAAATTCATTTCTTGATTGCAGGACAGTTAAAGCTAGCCACCCAT
GTTATGAGGCTATGTCAGATGAGGGATGGATTGAATTCAGATGAGGAAATTACCAGCCAAACTCTTGTCTCTATGCTTAATTT

ACTGGAAGGGCGTACTGCTTTTAATAATGTCTTTCTCCGGCATTATTTGTTCTGCATACTACAAATACTTGCAAGAAGGTATAT
GTTTCTTATTGAAATGAAATGCAAGCTTGCTTATTTTTTAGCTAATGTATTGTGTGAATATTGCATGTATGTGATAAAACAAAAG
CTGATTTTTAACTCTCCAATTTTTCATTTAGCTTTTGAAAATAATTTATTCACTGAACTTCTTTTATTGGTCGTAGTCGCTCTGTT
CACCTTTTTTTACCTATATTTTGTCTAATTTTGTTGTTTGTTGAACTCAATGAAATTTGAACATAGTAGTAACCATGGTTATTGA
ATCGAGATTCGAAATTTCATGAATTTTGAATCCCATCGAATCTTATTTTGAATCTTAATTTTGAATCTTAAGATATACATAATTTG
TATAGTTTAAAAGATAAAATAAACAAAACCTTTTAAACAATTACTATTTTTTATAGAAAAATAAAGAATATAACAAAAATTGTA
TATTAATTCAAATTTATTTCCTTTCAAACATATTAAATAAAAAATTCTTAAATATAGCCTAATTTAATACAAAAAAAATATAAGA
ATAAATATATCCTAATTTAAATTGAAAAAAAATTATAAAAAAAATAAAAAGACAATAGTTAGCAATAATTAAAGTATATAAGAA
CACTTATATATTAATTTAAACAATAACTAATTTTTTCAAAGAGATAATATTATTGTTTTACAAAAGTCAGAATAAAAAATAATATA
TAAATTATTACTGTCACTGTAAAATGTAAAAGTATGTTAGTATGAGATAAAAAAATATGAAAAAACATTTTATATTTTAAAGTAT
TACTCTGAAAAACTTATTTAGAACTCTCTCACATCTTCCTTCCACGGTCATCTTCTCACTGTTCATGGATTAATCTTCTCCCAG
ATTGATTTCCTCTCCCTTTTCTGTTCATTCCCATCTTCTGATGATTTCATCTTCTCTCATCTTCTCCCTTCTCACTATTCATCTTCT
CCTAATTTCTTTTTACTGTGTCTTCTGCGGTTCTATTATTTGATGTTCATCTTCTACACTGTTTATTTTCCCACTGATTATCTTTTC
CACTGAACTTTCATCTTTGTTGAGTTTCTTCTCCTGTGTTTCTTTCTTATTTTTTTAGTTTTTGTTCCTTCATCTCTTTCTTTTCC
TATCTGTTTTCTATACAAAAGAAGGTGAAAGGTTTTTTTTTTCTTCTTTCATTTGATGTTCACGTCTTTGGAAACGTGAAGTAA
AATGATAAAAATGTGATATTTTTGTAATTATTGATGAGAAAAAAACATGTTCTCTATTTAATAAAGTCCTAATGTTTTATAGTCT
GTAACCATTTTCACTTTGCTGCTATTTTTGCAAAAAATTAAGATCCTCTCTTAATAGGCTTAGCTGATCCAAACAATGACAATT
TGTTCTGGGCGTGTTTATATTTACACTGCAGTTTTGTCTGTATTTGAAAAACGAAAAATTCAGTGACTTTATTCCGTGCTTTTG
TCTGCAGACCACCAACTCTTCATGGGATTCCCAGGGAAAATAGAACGTTGCATATGAGTCTTACCGAAGCTTGTAACTATCA
GAAGAACATGTTTGTTCTTGATTCGGATAGCAAACCTTTGGATCTGCCAAGCTCTACTCAAAACCCTACACCAAATCTTTGCC
TAGATGATTTGAGGGATGCACTTAACGAAGCTTCTAAGGACCCACCTGATGAAGCTCCGGTACAGGTGCACATTGAAGCTCT
GAATGAAGCACCCCTAGAGAAAGCCGAAGAAGTATATACTGAATTTCCACAGGAAGCGCCAATGGAAGCTCCAAATGAAGT
TTCCAAGGAAGCTGATACTGTATCGAATAGCCATGAAAGAAAGAGGCTCATTAAAATCAAGGTCAAACAATCTTCTGCCACC
AGTAGGGCTGATACTGATAATCAAGTGGTTGAACGTTCTTTAGGTGGTCGTAACGAAATGGACCATGGAGCTAGCAGTTCAG
TTTCTGTAGATGCACCCCAGAGGAATTTTGCCGAGACTCTTAGCATGAGTAATCACAATATAGACGAAGTTAATTCTTGGCAT
GATCGTGGTTCGCGCATGACTGCTAGCATTGGTAGTGCTAAATTTTTGAGTGACGGTGATGAATTAGTCAAAGAACTTCAGT
GCACTGCTGATTCAAGTATAGTTTATTCACAACCTCAGCCAGAAGATCCGTCATCATCCAGTATTATACAGGATAACAATGTAG
ATGCTGATGCACGACGATATGCCAGCCTTCAAACTCTTTCAGTTGCAAGATTTGATCCTGAGGGAGAATCATTAGGTAAAGA
AATTTCTGCTCGTGGCAAGGAAAAACACAAAAGCAAGGAAAAGAAACGAAAACGGGAAAGTAATAAAGGACATCACGAT
GATCCAGAATATTTGGAGCGAAAGCGACTAAAGAAGGAGAAAAAACGACGGGAAAAAGAACTGGCAAAACTTCAGAGTG
ATGAAGCGAAGAGATCCTCTGTAGACTTGTCAAGTAAGAAAGAGGAACCTGTAGTGGACGTTTCCAGACAGATTAAATCTG
TTGAGCCAGGCGGCGGACATAGTTCTAAATTAGAAACCAAAAAGATTGATAGCAAACCGGATCCATCCGAAGGCACGTCTG
GTGCACCAAAAATTCGAATTAAAATTAAAAACCGAATGCTCAACAAGTCATAGAGAATAAAACGTCCATTATTTTGTATATTC
TAGAATGAAAATTACAACGCCTTCATATTTTCTGGGTTGTATTTTTCTTTTCCTTACCCGGCGTACACGAATTAGGTATTTCCAA
ATATTGATTCCATGAGCTTCATTCGTTGTTAGTTCTAAACTATGATTGGAGAGATGTGTATGATTGAAATTTCAATCATACAATA
TTTGTTATCGATGTAAATGATGCGTATCACGATTTTTTTAGGATATTGTTGTAAATTTACTTGTAGTTGTAAATTTATAATTTTGT
TGTGGTCTA

>VrTAF4b LOC106764661

AAAGAAAATCTTAATAGAACGTTTTGATCTCCTCAAAGCTTCATCTCTGAATCAGAAAAAACAATTTCGCTCTCAAGTGCTC
GCGAATCTTCGTCTTCAAAACAATTTTGCTCTTCGATTGTGCATNTCTCAATCACAACATTCCATTGCAGGATGTTTGTTTTTA
GGTGTTTTTTGCTTCCGTATCTGTGGTACTTTTGACACCGGTTGCTTTTGGGGTTCGGCATCTGTGGTTTCTCTGTCGCGGGTT
CAGGGCGGTTGCTTTGGTGGTTAGCGCTTGCTTGCTTCACTTTAAGTGTTCGCAGCTGCTTCGCAGGTTCGTGGCCACTTCG
AAGGTCCGCGGCTGCGTCGAAGGTTCACGGTTGCTTCAATTTCACGGGTAAAAATTGAACGCGTGGTTGAACTGCTTATGGA
TTGGAACATGGTGGTGTGATGCCATTTCAGTTTGTTTGCTTCCTCTTATGAGCTAAGATATGGTGTGAAGCAAATTTTCTGAGT
TTGTTTTGGTTAAATAGAGCAACAGAGATTACAACTGCAGTGAAATCGCCATGGACCCATCTATCATGAAGCTTCTAGAAGAT
GACGAGGTCTACTTTGCTCTTCTTTCTTCATCTTCTTTGAAGGAGAAAAACTGTGGTCTTAGTCTTACTGATTTGTGGCTTTG
CTTACCGGACCACTATGTTTAAATATTCACAGGATGAAACAATGCATTCAGGGGTGGATGTGGAAGCCTTCCAGGCTGCTCTA
AATAGAGATATAGGAGGAGATGTGTCTGCTTCTCAGTTTCCTGGTTCAGATGCAGGTATCTGCCAATTTCATTCATCATTTTCT
GTTCAATACTGACCTCTCTTTCTCTCTCATTTTGGTAATATTTGATTTCTTTGTTATTTTTTATCATTTCATTCAAATTCACTGCTT
CTTCTGTCCATGTTTGAGTCTGTTTATACATCTAAAACATAAAAAATGATATCAACACATTTATGTTTGGTCATAGTGTTTGAAC
ACGGAAGTATTTTTGGTTGTCAAAGAAAGTTAATTAACTAGGCCTCAATTGATATCTCCTTTCAGTCTATGTGTTTCTTCTAAT
GGAATTTGGCCAGATGTAGTTACAAAGTCCAAGATATATTTTGTGGTATCCTAAATAAATCTCTATCCTTCCCTTATTTGAGTTT
CTTGTGTGGTCAGCATTCAAATACCCAACAGTATCCACCAAATAAAAAACACAATAAGGACCAGCTTGCCACCAAGTTAGAG
GCTCCTATGGTTTCTTGTACTAGCTACAAGAATTATCCCATTGCTTGATGTGTGGTGGCAGGGAATCCAATCCTTTGAATCCTC
TCTTTTATTAAAGTTGGCATTGGAGATTTGTGTGATTGGCCTGTAACTGCTGAAAATTTCCATGGATCCCATTGTTAGAAATAG
GCTGGCTGGAACTTCCATCTCTTGGAACCCACTTTGCCATTAGAGTTAGCATTGACATTTTCCGGATCAAATACTAACAAGTC
ATAGACCAACCCAACCCTCATGTAATGATTGTATGACTGTTGCATTACCAGTAGTAGTAATTACTTTGTTTCTTGTAATTGCTTT
CATCATTTCCTTCTGGATAGTAACTATAGGACTATAATTCTGAAACAAAGCTGCATGTCGCATTCTGGCTTTGTCTCCCACATA
ATTGACTGAAAAGTGATCGTTCTTCTCTGAAGCTCATTGAAAGAACAAGCATGGTATGAGTTAAATTTAATAGTAAATGTTTT
AGTTTAAGTTCTAAATTTAATTAAGTGTTTGGGACATAGCTTTTTTATATTGCTTTGTGATTTCTCAAGTGAAATGCTCATTCTT
TATGTTTATGGTGGTATATTTGCTGCAGTGTTGTCTCAAGGAAGCAATAATACATCAAGCCAATCTTCATCACAGTGGCCTACT
TCTAACCATGACAGCCAAAGTGATGGTCAAAATCAAGAACCTAAAACTGCACAAGAGCAACATCCATCTGAGATGGAGCCA
AAGCAACATGGGTCTCTTGGCGAACACCTTCAGCATGTTGCTTCTCAGGATGTAAATAATATTCATTTATCACAGAAACAATC
TCAAGATGACAGTCACCAAACACCTGCTGTACAAGTTCCCCTTCACAATTCTCAAACTATCGGAATTCATAATTCTGGAAAA
GATTCAGTCCTTAATAAAGAAGTAGTAAAGAGTCATAATCCCAGCAGTGAATCTCAGTATGCAAAGTTGCAGCAGATGAGTA
ATCAACAGGCTACAGTCTCTGAGCAGCCAAGTAGCCAAGTAAATCGCAGTAATAAACAAGTACCATTTGGCCTGTTGCTACC
CATCTTGCTTCCTCAACTTGCCAAAGACAGAGCCATGCAACTTCAAACCTTGTTTACTAAATTGAAGGTGAGTTTTCGAAAC
CCATCCAGCTATTAGGTCCTTTTCCCCCTCCCCCTTTCCATGGGACATTTTAGTCTTTTTTATGATAGCCCAGAACCAGCTTATA
AACAAATATTACATGCAGTATGCATTATTCGTCTATTTCATCAATTTCTTTTTATTTTTTCATTCTAGAAAGACGAAATACCGAA
AGACAGTTTTGTGCGGCTTATGAAAGGTATCGTAGGGGACCAGATGCTTAGATTAGCATTAGCAAAGGTGCAAATGCAACCT
CAGGTAGTAAGAAACATGGTTTTTGTTGTGGGTGGTTTTTATAATCCAGTTAGTTATTGATGTCATGGTCATGCTTAGATTCAT
TTTTTCATACCATTTAGGCAAGGCCCAATCAAGCATCTGCTGGGCAGCAGCTTCCTGTAAGGATGCCAACTGTTAGTTCAGGT
GCTAGACAATTGAACGATCCACATGCTTTAGCACAGATGCATCAAAGAAGTATGAATGTTGCTGTTGACCAATCTCGCATGA
GTTCTTCAGCTGGCCAAACCATGGATAGCAATGCTAGAAAATCTCAGGAATTTGATGTTAAAATAGAATCTCAAGGATTGCA
ACCAAACCAGTTGACGTCTTCCACTTCCAACTCAGTAGCTCAAGAAACAGAAAGAGCATCAGTTCACATACAAGGGCTCAA
TAAGCAGCAGCAACATCATCTACATTTTGCATCAGCATATGGAAACAGTGGTGGTAACTATAACCCTTATTCTGGGACAACAG
GCAGTTCCACTTCGTCTATCAAACCGCAATCACATGATTCACACATGAGTCAAATTCCTCATCAAAGTATTGGTTCAAATCAC
TTAGGTGGGTCAACACATGGTTTGAGTGTAATTGGTATGCCAAAACTTGAACAGCAGAATTCTTTCAATGATCCCAAGAGAC
TGCCAGGTGGATCTGTGTCCTCTGCTGTAAACAATGCAGCATCTCAACAAACTTCAAATGCTTGGCAACCATCAACAAACAA

AGAACAAAATCTGGGCTTAATGTCATCTGTTTCTTATGTCAAGAAGGAACCTGGTGACATGTCTACTGAGCAGCAAAATAGG
CATAATTTGTCTAAATTGCATGGGTATTCTTCTGTTAATTCTGCTCAGCTTGAACAGAGTGGTGCCAGTCAAGGAGCTCTAAA
AGACGAGTTTTCAAGAGGTCTTCCAGCATCCACAAGCATGCCACCTACAACATCTACTGGCTTGCTACCACACAGTTCTGCT
TCTGCTTCTGCTGTGACCCACCTAGATTCCAGTGTCTTGGTAAGTTGGAGACTTTTGTACATGATCTTGTCTTAAGATATAATG
GTAGTATTTTGCAGATCAAGTAAAATTCAAATTCATTTGCACAATGCATCTTTTAGTACTATGCTATGCTGAATCTATATATGAA
ATATCAGTTTTCTCAGCTCAGATATGTTTTCCATAATTTTTTTTGTCAAATTGTTTTTAGCTAAGAATGTCTTTCACCAGTAGTT
TTTGAAACCATTTTTTAATAAAATTGCATGGATAGTATTCACTGTCTCTTATTTGGGTTAGCTTACTTGCTGTACTTTTGGGTAT
AATAAAGTATGCAAGAACTGATTTAAACAACTCTGAAATTCACCAATTTATGCACCCCACTTCTACCCAAAATATGTAATATGA
AGAACCAAACTGGTAGTAAAGTGGAATAGTAGCACCCCTAAGTGGGAAAACCAAAATCCCCTATTCATAAACCAAAATTAAT
GATTAGTAAATAAGAACCCTGATTGAATCATCTTCTCAATTCTGTACTTTATCTGTAACTAGGTCAATCACGCATTTATAAAATA
TATAGCACCTCTATACATTATCAAGCTTTAGTTAGCTTCTCCTAAAAGATCAAACGTTTTTTTTTTTTTAATTAAACTCTAAGGGC
ATCCATAAATTGACAGTTTGACACAGGATGTACCAGAGCCAAGCAAGCCAACTATACAGAAAAAAAATTGTCCTGGATTTTTG
TTTAAAGTAACTATAATTATGGGGCTGATTTAGATACTTTTGATTATTGCTAGATCTTAAGATCTTAATTTTGAGCATTGTTAGGA
TTGCTAAGCTGTCACGTGGTTAATTTCGTTAGTTCAATACTGGAGGTGCTTTTATGTTCATTTGATCATAGGTTTATGTTCGTGT
CATTTTCCTTGTAGGAAAGACCAATTAAATTTCTGAAAGTTTCACTGTCTTTGCGCATAGGATTGTCCTAGTTTAGTCATTCTG
TATGACAAGTTTCCAGGCTCAAGTTAAAGTCACGAGCTACTTGACTCCACACAATGAGCTCAATTGGGTTAAGCCTAGTAAA
CTATATCTAGTGCTCCAAATGGGTTTTAGAAGGAGCTTCAGATTGCAGAAAAATCACTCAATATATGTAATATACTTACTGTAA
TGCAACCAACACTCATGAGTAAAAACTATTATGTAATACTTTGAGAATCATTTATGTACTTTCCCCCAATGGTTTGAGATTTTG
TTATGACTTGTTTTTGACATGTCATTTATGTTCTAGAGGTCATGAGTTTGATCCTTTGTGTCCATAATCATTTAAATAAGAAGGC
TAAATTCAATTGCAAGGTATTGTAATGCCGGGGCATTATTAATGTGTTAAGCATGAAAACCTGGGGGAACATTTTGATGTAAA
TTCATTCATGTATTTTTACCTTACTGCTTAAGCTTTGGGAATAAATATATATGTTTTGATGATGTATATGTTATGCCAGTTCATGA
GGAAATGAGGTGATAACTAAGTGGCTTATGCTTAGATTATATTTTCGAGAAGTCATATATTTTAGCTCATGATCCTCTCGTTAGA
TCTTGAGCTCAATGTACTGAACTAAAATTGAGTTTAATCATGAGCTGCATGCAAATGGATAGGTTTCGTTATAGTCCTTGTCTG
CTTTAATATGCACTATTAGATCCATCGGCCCCTATTTCCAATTTTGTCAGCGTTGAGTTTTAACTCTTTAACCAATTGTTTGCAA
AATTGCTAACCTCTTACAGGATGTGTAAGAAACCTTTGATTCACCAGTTTTGTTATGGAAGGTTATGATGTATCAAAGATGTAT
TGCAAATTGAAATGGAGGGTAACAAGTACATATTGTTGTGCTTGTTTTTTCATTGGACGTTCTTGTGCTCCCAATTTTTTCTTT
AGCAGAATCTGTTATTGGAAAATAATAACTTTGTCATGCTTTTTCAATGCATTTTTTTTTCTTTAAATGCCAAGATATTATTTTT
GCACCGAGCATACCCCAGGTTTATAAGTATGTCTTCAAATTTGATGTCTAATTTCTTAAGTCCCACTTCTGAAATTAATCAGCT
AAGCTCTCAGATTCCATCAAATGCTTCTGGAATTGCTGCAAGGCCATCTCTTAAAAAGTCTGCTGCTACCCAAAAGAAACCA
CTTGAAGCACTTGGTTCATCACCTCCTCCTTCTAGGTCAGCCATGCCTTTCTCTACTGGTTAATTTCTTAACCTAATTAATTTAA
ATATACATAATGTTCCTTTACCTTACAGTAAAAAACAAAAAACTTCTGGGGGATATGCTGAACAAAGCATTGAACAACTTAAT
GACGTCACTGCTGTTAGTGGAGTTGACCTCAGGGTATGACACTACGTCAATGTTCAACTAAATCAGATAGTCAAATAAGTAG
GGAATGCTAACTCCTGTACTTCTAATGTTTCTTTTACAGGAAGAGGAAGAGCAGTTATTTTCAGGGCCCAAAGAGGATAGTC
GAGTGTCAGAAGCATCTCGAAAGGCTGTGCAAGAAGAAGAGGAAAGGCTGATCTTGCAGAAAGCTCCATTGCAAAAAAAA
TTAATTGACATCAGTCAGTGTCCAATTTGTGTCATAGCTCTGTCCCTATGTTTTAATTTTTTCATTTTGAGCTGATCTGGATGTC
TGCTAGGCATACAAAACCTTATGGTTACTTTATGTTGTTACTCTGACAGTGGCCAAGTGTGGCTTGAAGGGTATGAGCAATGA
TGTGGAGAAATGCTTGTCACTGGTAAGACTCAAGCAATTTTTCACTTTTCAGTGCAATTGGTTTAGGTGTAATCTTACCACTG
AGTAAATGTTCAGAGTGTGGAGGAAAGGATGCGCGGACTGATAAGTAACCTGATTAGATTATCAAAACAGGTTAGTTGTTTT
CTCTTGCACAAATAGTCTTTTATAATTGTATAAAACTTTGTACTCACAAGAATTTTTCTACTAGAGGGTTGATTTTGAGAAAAC
GAGACATCGGACTGTTGTCACTTCAGATGTTCGGCAGCAAATCATGACAATAAATAGGAAAGTAAGGGATGAGTGGGAGAA
AAAACAGGCGGAAGCAGAGAAGCTTCGGAAACTAAATGATGTAAGTTCCAAATATTGGAATTGTGTTGAGCTACTGCAAGA
TAAAAACCTTTTTTTCATGCACATTTCCTTTCCATGCATGTCTTTTGGGAATAAGAGTTCATACGGCTGTGGATAATACCTACC
TGCAACTTATTTCATCTAAAAAAAAATCTGCCTCATTACGGTCTTACTACCAGTTTGGTATCTGCATAAAAATATGTGCCATATAT
TTTTTCCTTTCAGTTTTTGAATTTTAAATAAAATTATTTGAAAAAATGTTTTAAAATATAAACTTAAATAAAAAAATTAGTTTTA
AAATACAACTAATAAAATTGCTTGATAAAATATTAATGCAAATATATTTGATCTTTCTAAAAAAAAACTTTAAATGAGTTGGACC
ACAAAATATAAGTTTTAAAAAATTAATCCTTTTAAAAACAACTTCAATTTCATAGGTCTAAATAAAAAAAATATAAATATATATG
AAATCAAATTTTAAACAATTCTTCAATGGTTAGTATTTCTCTTACTACTAACTTTATTTTGATCACTTAATTTTTTTTTTTAAAAT
ACTCAAATAAAATTCATTAATAATGGTTAGGATTAGCGGGTACTTGTACTTACTCCTTTAACAAACGGATAATTAAAATACTCC
CAATATTCCCACTATATTCGTGGGTACAGATTATTTTACCATCTCTAATGGTCTTTTGTATATTTTCATTTGTAACTTGTGTTACTA
TCATTTATATTACTATTTCTAAATATAGGTTGACAGCAATACTGGAGGTGATGGTGACAAGGATAAGGACGACGGCCGTGCTA
AATCAACAAAGGTATTTATATCTCATTCTTATACTTAACCTTATCAACATTGGAAATCTGTTCTTTGATTTATAATATGCTGTAGG
TGAACAAGGAAGAGGATGACAAGATGAGGACAAATGCTGCAAATGTTGCTGCTCGTGCTGCTTATGGGGGAGATGACATGC
TGTCGAAGTGGCAACTTATGGCTGAGCAAGCCAAGCAGAAACGTGAAGGGGGAGTAGACGTGTCATCTAGTTCTCAACCAG
CTAAAGATGTGAACCGCAAATCTTCATCAACATCTGAAAGAAGTACAAAGGATAACCAAGAAGGGGAAAAAAGAGGTTCA
ACCCCTTTTCTAGCAAGCTCAGGTGAGTCTGCTTGATGGTGTATTTCTGTTATTTCCATTGTTAATCGTTTTAATTGCCAGCTA
GCAATCAATTTTAGTTATTCAAGATTCAAATGTTTCTTAATATATGGGTTAAAAAAATTAAGATAGAATAGAAGTGGATTCTGT
CGTTGTCAGAAAACTTGATCGTATGTTCTCTCTATACTGTTAGTTGAGAATGTTTTGATCAATACTTAAATGTAGATTGCAGAA
TGTCCCACTCTTTCCAGTTATTTGGGGCTGAAGGATTTCAGACTCCGCTCTTGTATAGTGTATGCAGAATATCTACTGATTAAT
GTTGTCACGAAAGTGGCTTCAAGTAACTTTATGTTGGAGAAATGCTATGTTGGTTTGAATAAAGTAAACAGGCGCCCTTTTTT
TGGTTTGTGCCACTTTTCATGTTGTTTCTCCCTTTTTGATTCTTCTAACAGTGGCCAGAAAACTTGGGAAGAGTCATGCCATG
GCCCCTCAAACTAGGGTAGCTCGAAGCATCTCTGTCAAGGATGTGATTGCAGTCCTGGAGAGGGAGCCCCAAATGTCTAAAT
CCCCACTCATACATCGCTTGTACGAGAAAATTCATTCTGAAGCCCCAGTTGAACAAGGTTAATCCAAGTGCCTAGGCGATCTT
GTTTATTTTAATGTCACCGAGCTTATTATCCTATTATCATCTTATGCTTCCCTACGGAAACTGAAACGGTTCCATCAAGTTAAAA
ATTTGCTGTTTTGACTTACATTTCATATAACCCATATAGTTTACCTTTTGAATTTATATTTTCTCCTGTATTCATGATCTCCCTGTG
AGGGATTTACCTAGTTTTTTGAGTTCCTTTGTGCGTATTTTATCGTATATGATTAATCGGGCTACACCAAGTGGAATTAATAATG
CTATTTCAATCTCAATTTTAAAA

>VrTAF5-1 LOC106765332

TCAATATATTTGGGTGAAGTCTATCGAACTGGACTGATTTCCAGCAGGGTGAGGGAGAGATAGAGGGAAACAGTAGCGCAG
AAGAACACAATCTGATTCGGCATGGACGAAGATCAGATAGAAGGTTGTGTGAGTGGGTACCTCAAGCAGAAAGGGTTTGCG
CAAAAGGATGACCAACTTCAACTTTCCAACGCGGATTCCTCTCTTCAACCCGACACTCTCAATCGCGCTCAGTATTCACTCT
CTCTATCCTTTTGCGCTTCAATTTTCGTTTTCTATTTATATCAAATTATAGTACTTCAACATCTTTGAGTCGCTAGGTATCATGAT
TCATAGTTTAAAGTAGTTTCACTCCTGAAACGTGTGAATAATCAAAGCATTCGCCAAATTACTTAGATATTGGTATATGGTTTAT
TCTATGTCTAAAGTGACATCACTAGGAATTGAAGCTTGAATCACTTAAGATTAAGTATACTCACTGCTTAAATTTTGCTGAGGT
ATGTTATGGGACGGCTGAGAGATGTCTTTGTCCTTCAAGCATGTGATTAGGAGTTTGATTTCTAGGCCAGTCTGAAAAGAAA
AAGAATTGTTAGGACTGTTGGGTTTTTCCATGGGAGATTTGACACTAATGATTGGGAGATTATCTTTTTATCCTACAGACAATA
AGTTTGAGTCATTCGTATGTTATATTGCTCAGTTTGTTTATTTCTACTGAATGTAGGACTTCTAAAACATTTGAATTTTTATATGC
AAGACTTATAAAGCACTTGAATTTTTAACTAGGGGAGATAAACTGAATATAGGATTTCTAATGCACTTGAAATTTTAACAACT
GAGAGATAAACTGCATGTAGGACTTCTAATGCACTTGAATATTTATAGATAGCAGTATCTTGAGTGATTAGTCTTCAACTATTG
GGAAGGAATATTCTGCATTCACTGAAAAAACATAATTTTCAATTGTAAGTTTTAGTTTTAAAATTTTAAATGTCAATGAAACTG

CAACAGGGGGTAGTTGCCCACCTTAGTGCCTAAGATTTGGGTTATATTTCATATTTTGAAGATGCATTGTGTGCTGTGGAAAA
CTTATAGATTTGTGAACCGATGGTGCATTTTTAATATATAGAGTTATTGTTCCACATTGCTACACGTTGCTAATTTCTTTGGCCAT
AAACAGTCTTTCTTGTGCTCTTCAATGTCAATTTAGAGGATGAGCTTCAATGCAAATTATGTTTAAAAAGATAGGTTAGTATTT
TTACTTTTGTGAATGAGACAGAAAAAGTAAATTAGTTAGGATTTTACAATATTTTATTTTTTTCAGGGAGGAAATATAGTATTTT
GAACTAATTAAGAGGATCAAACGACAGTTACTTTACTTGGTCTATTTAAAGTGTGTTTTTAAGCTACAATGTTATCTGTTCTTG
ACTTTTTTAGCATCTCACAAGAAAACTATTGCACAAATATGATTTCAAATTCTATTGCTATCCTTTAAAAACAGTATCAAGTAT
GTAGTCTGAAGAAGAGGTTTATGTGTTTTCTTAGAATATAGTCATCAGTTTGCACGAATTCTATATTAGATTGTACTTTCAACT
CAAATATTTTCAGGATTATTTGTTGAATTAAAACCTTTATTTTGCAGATTGGAGAGAGGTTCTGCTAGATACCACGATGGATAT
GGAAGACTGAGATCATGGGCATATAGGTCACTTGAATCATACAAGGTAGCATGGCATTTGGTTACTGTCTTGTTTAAAGTTCA
TTCATGAAATTGCCATGTTTCAGTTTCTTACACATGGTTTGCCGTTTCTTTGCAGCACGAATTACTGCGTGTGCTTTATCCAGT
ATTCGTCCATTGCTTCATGGATCTTGTGGCAAAAGGGCATCTTCAGGAAGGTATGAAATATTTCTTCACACAGACATGTAACA
ATAATTATTAATATTATTTTTTATGGATTTTTGAAGTTCCTAATGTGGCAATCAATGCATCATTTGTTTTATGCTTCTATCACATGA
TTTGTGATTCTGTCGGATATACTCAGCTTGGAACTTTTTCAATACCTTTCGTGAGGACCATGAAATGTTGCACTCACGAGACC
TTCAGAAATTGGGATTAGTTCTTTCTCCTACTCATTTGGAGGTTAGTTGCTATGTGAAATTGAATATGTTTCTTGAAGTTATTAG
ACTGCTAGCACTATATAATCCTTCAATATTCTTTTTTCTCAGGAAATGGAATTCGCTCATTCCCTTAGGCAGAGTAAGTTCAAC
ATAAAGATATGTCGGGTAATGTCTTTATTTACCTGGATTAGTTTGTTAATATTCGTTTGTTGAACTGTTTCTCAGTAACTCCTTT
CAGTACTCATCAGATTAAATATTTTTTTTAGTTAGTTTCGTAATCCATGAGAAGTTGGGCAGAACTTTTGTGCCCTTTTTGGGC
CTTGTTTTTCTGTGTTTGACTATTCTCCTGTGATTTTATTTTCTATTAATATTGATGATGCTTTTCTCTTCGGAAAAATGCTTGGC
TATTAGCATTTTGATTTGCAGTTCTGAATAAAGAAAAGAACCATATACGTAAAAACCAGTTTTGGCTTTATTTATAGTTTTGCT
ACGAATTTTCATCACTGACGTCATTACGTTTATGTTCTCTATACATTGAAAAGTCTGATAGTTAATGTGTTGGACTTCTGGCAG
TATTCTTATGAGCTTCTATTGCAACATCTACATAGTATGCAATCCACCACAATAATTGGAATTATCAATGAGCATATTAACTTCC
AAGGTACTTTTCCTCGTTTTAAGTTGTGTTAATTCTGTTCTTTTACTTTTCAGTTTTGCATAAATTTCATTCTTGTCACACTGCA
GTTACAGCTGGGCAACCGAGCTCAATTTCTGATGATCCAGAAGCTGTTACTCTCAGTGGAAGCATCCAGGATGCAGTAAACC
AGATAAATCAGAAAGAAATCCTTTGGGGGGTGAGTCCTTCAGTATATTCACAAAGCTCGACAATTTTGTAAAATTATCTTGCT
CTTTTTTGTTTCAATGCGTTCCAGTTTTAGTTTTGCAAGCTTTTGTTATAAAAAGTGTATTATAATAGCATCTATTATACCTAAAC
TTTCAAAGACTTAAAAATTGCTCACTTTTATGCATTTCACAATTGTAATGCCTTGAGAAACAATCAAATGAACTTTGACATCT
GTTACATCCTTTGTCATAATTCCATCATTGTACTTGGGTTCTCGAATACTAAATTTTAATGAACAGACTCTTTGATTGGGCCAGA
TTTAGATTCCTGACTTGTTTTCCAGAGTGGGAGCAATGACCCAAAATTTGTAAAACTACACAATTTTTCTTTCCTTTCCTTTAT
TGTGATGTGCTGAAGGGGATTGAAGTAGCTGTTAGTTTCATTTAATAGTAAACGAAATTACTTGGTAAAAGCAAGGTCCTTTA
GGTCGACTGACTTGTTTTTGAGTGATAGTTGATTATTAGATATGTACCCAATATGCTATTCAACCATGTAGAAGTATCCATGCA
GCGTAGGTATGCTTACTTTAAATTTTCAAATTTATAGCTATTGCCATCGGGGTTATGCGTTTAATTTGGACTGGCAGTTCTGGTA
TTGTTAAAATACAGATTTTAGGCATAGAGGGAGACAGAGAGATGAACGGGAGTAAAACACTAATTTATTTTAATTGAGATTCA
TTACATATGCTCATGAAACCTGGAGAACTAACCCTTTATATTAACTAACTGCTTGATAATGAGGAAACTAATCCTAGAGGATAA
CCTAAGATATAATAACCTATTTTACGTATTCTAAAAGTTATCATTCAGTTCATAGACTATGTATGTATGTATGTATGTATGTATGTA
TGTATGTATGTACGTATGTACATATGTATGTATGGATGTATGTATGTATGCATGTATGTATGTATGTATGCATGTATGTGTGTGTCT
TGTGAAAAAGTCAAACTGAGTACACTGGGAGGAAGAAAATAACAGATTGTGACATTTATGGTCGCAGCCTACAATCAGATA
CAGGCTCAAAAATAGGAATTGTATAGACATTAAGGCAAATAATATTACATCAAACATTAATCTAACACTCTCCCTCGAGTTAG
AGTACATAAGTTATATCTCCCAAGCTTGGACATATAAGTTGAATTTGAGGCCCTTTCTAAGACTTGGTCAAGACATGTGTAAG
TTGATCGCTTGAGATGATAAATTTGGTAGGAAATTCCAAACGCGAATAAAATGATAGTTAATCTCTATATATGCTTACTTCTTGC
ATGGAACATTGGATTGAAGGAAGCATGAAAAAACTGTTCGATAGTCACAATACAATTTCAGATGTTGAATATCACAAATTTTA
ACTCTTGAAGTTGTATAATCTAGGAATGACAATGCGGGATGGGCCATGTAGGCCAGCTCGCAAGAAAAAGTGTGGGCCAGA
CTGAATATTTTGAGCTGCGGACATACTTAGATCAATGTATCATCATTAGAAACTACGTTGAGAATAACTCATTAATGAATGTGA
TAAAATATCTTGGCCCTAATGTGGATGCAGTATGACTAGGACATGGAACATAAAAATGGACTAAAGTAGGAGTCATAAAAGA
ACTACGAAATGTTTTCCTAATTGATACTTGATGCAATGGATAATTTGGAGAGAGTTGACACCATTGTCGCAAAATAAGGATTA
CCTAACTGAAATGTGCATAAGGATAAGAGAGTCCATCATTGAACAAGCATGAGTAGAGGTATTGTGTTGATAAAGTCTGTGT
GACTCGCATCTAGTGCCAATCATCCACCCCAAACTCCAGTTTAAAAATGTTCTTCATTGGATTCTTTAATAGAAATATTTTTCT
GATTTAAATAATTATTGGGTGTTAAATTATGGTCTGATTTCAATCTCATTATTATTTCTTCATAGATGTTTGAAGATTCTGTGGAA
GACCACGTAGATAAGGCTGGGGCCTTGCTTTCAGGCACAGAAAAGGGTGAAGGCGAAGGCAAAGAGGGGGACAATGATGA
GAGTAAGGTACATCTGTTATTAAAATGCGCCCCTGACTCTTTATTTTCATGAATGCTTTTACTGGATGATCTGCTATACATTTCT
CAGAAGTTATGCACTTGTAAAAAAGTTGAGCATGAGTTCCATTTGAATTCACTTTGATCACCTTAATACTGCAGATTCTTTGAT
TGTCAAGAAGTGTTAATTGTTTTTCACTTGTTTCTCTTTTAGAAAAGGTCAATTGATGTAGGAAAGCAAGGTAATTCAGTAAA
AAAGGTAAAAAAGGACAAGGTCAGCAGTGCAACAGGGAAAAATGCTAAGCCAGAAGCTACCACTATATCTGCAGCACCTC
GCATTAAACCAGAGATACCTTTGCCCATAGTGTATTCTCTCAAGTCTTTTTTCTGTATAATTGGCTTTACATATTCTGTGTTGGT
TGTTGTTTAAACTAGAAGATCATCCTATCTATATTTTTTATTACAGTGAGAACTGCAGAGTCAAAGTTTTGCTCTATTAAATAAC
ATTGAAACCACAGAAAAGCATTCATTAAAAATATGTAGTTCTATTATGTTGAATGTTGTAATGTTATTCTTGCAGTTCAACTGA
TGTAGAACTGTCTATCCTAGACGATTTAAGGAATCGTGTACAATTGAGTAGTGTTGCTCTCCCTTCAGTGAACTTCTACACAT
TTGTAAATACACATAATGGGTAATCTTTTACACTTTAGCAGAACAAGTTTATTTATGTTTGGTTTTCTGTTGGGTAACACTTAA
GTTTTTGCTCAACTACCCAGTTTAAGCTGTTCATCAATATCCCATGATGGGTCATTGATTGTTGGAGGATTTTCTGACTCTTCA
CTAAAGGTTCTTTCTTTTCATCCTCCTCTTTCCAACCTTTTTCCCTTAACATCTTTTATCATACAAGATAATGTGAACTTGGATT
TATTATTGTGACTTGTGTCTGCAGGTCTGGGATATGGCAAAACTTGAGCAACAATCCACTGCTCGTAAGTTCGTTGCTTAAAT
CCTTTTGTTTAGACCAAGCTGAGTAAATTTGTTGCATACTTTGCTTTATAAACTTGAATACGACTATAAACAAGTTTCACTCAC
TACTATTAATTGAGTATGATGGTTATCTTTAATGTGCTGCAAGGTTCTAATACGACTTATAAAAGAACACACTTTGTTAAATTCC
TTATATGTATGTATGTGTAAAAGTGTATGCAAAATATACAGTAGCATAGCTTCCTCATGGTGCATTTTCTTTTAGATTTTTTTATA
TGAAGAATTTGGAAGGGGTCAAAACTTGGTATAAAACGTGAAGTAAAACAGTGGGTGCTCTATTTTTTTCCCTGTAAGTCAT
TGGACATTTTCTATTAAAATGTTTGGGGGGATTGATGAATCTTTTGGTCTTTTTTTTTTTTTTTTTTTTTTTTCTGGATTGTGCAG
TGGATCTTAAATATCATTTAAGAGAGTGATGTCACCGTAATACACACACATGCCTGCAACATATACAGTAGTAGCTTCCTTCAT
GGTGCGTTTTCTTTTAGATTTTTCCTTAAAAAGTTTTAGGGGAAAAAACTTGTGGCATGAGAATGTGATGTAAAAGAGTGTG
GGTGCACTATTTGGCCCCGTTATGGCTTTTTATTGGAAATGTTCTGTAGAAGGTTTAGGGGATTTATGTGATCACTCTGGGTCT
TTTTTCTGGATTGTGTAGTGGTCTTAAATATCGCTTGAGTGATGCCACTGTAATTTGTAAGCAACGAAATCCATCACTAATATG
ATGATAATCTTGTCCACCCACACATAAAACTGGCTTCTTGAACTCAAATGTCATGTCTAATGTTAAAACATTGGGCTGTCTAA
AAATGTAACTGTTGTAGTTGAACCAAGTCTACACTGAAAGTTCTGGTTTAGCTAAATTTAAACTGATTGAAGCAAGCTTTTAA
GTCTTTAGGAATTCAATATGCTCAAATATGAGTTTATCTGTTTGCTTGCTATTATAGTTCAGTTTTTCTATCATCAGATTTTTCGC
AGGGAGGGAATGACATGTCACAAAACGAACAAATTATTGGGCAAAATAGTGGGAGAAGACAGTACACATTGTATCAAGGTC
ACTCAGGTCCAGTCTATGCAGCCACTTTTAGTGCTGCCGGGGATTTTCTTCTTTCCTCTTCAGCAGACAAAACAGGTAAGTTA
ATTTGATCCCATTTGAGTGATCCATTGTTCTTTCAGAATTCTGTCTTCACCTTTGACAGTTTACTTTGTTGTTTCACATTCAAGT
ACGTGAAAATAGGGTATACTTGACAACTCATACAAGTAATTTTTTATCAATTGACAATGTTAAATAGTTTACATTGGCAGTTTA
TAGCAATTAAGGTCTCTATATATTTGTATTTTTAATTCTGTGAGTCTAATTTTGAACCATGAATGTACGTTAATGCAGTTCGACT
ATGGAGTACAAAGCTTAATGCCAATCTTGTTTGTTACAAGGGTCACAATTACCCTATCTGGGATGTTCAGGTAAACTTTTATCA
TCTAAATCTTTCTAACTTCAATGGATCTTGTTAAACTCGAGGGTAGAGAAATATTGAAACTGTTTCTCAAACAGTATGAGTAC
TTTATTTATATTGAGAAAAAGTAGTCAATACAATAATAGAGGAGATTTGATATTTTTTAATGATAAAGATATGATATGGGAATAA

ATTAAAACATCCTAATATATAATATTCTTGGTTATCTAGGATCAACGCCTATAATTGTAATGGATGTCATGACATTGAACAACTC
TCCATATACTCTAGGGATAATTGTAAATTTAAAGATTTAAATTAAATTGTAATTGACGTGGGGATGCCATATGATTTTTAGAGAA
GTTGTATTGTTACAGAAAATTATGTAACAACGAGAGATGAGGAAGAGAAGAGAGATACTGTGGGACGAAATGGTTGATGGG
ATGAAGAGAGAAAGTGAAAAAAATAATGTAAAAGATATAATATTCTAAAAGTACTATGCAGTGTATTCTCTCTCAACACAAGT
ATGAGCGTATCTTTGGGAATTCGTTCTCTCAAGTTTTATTTTTGAAGCTTATGAAGATTTTCTTCCCTATCCAATGGATATTTTG
TTTATCATCTTTCCTTTTGTCTCATTCTATTTAATTTTGAGTCATTATTGTCTGCATTTACATTCTAGACTGCTACATTATTTTGTT
TTTCTGGTGCAGTTTAGTCCTGCAGGACATTATTTTGCCAGCTGTTCACATGACAGAACTGCTAGGATTTGGTCTATGGACAG
AACAAAACCTTTAAGAATAATGGCAGGACATTTGTCAGACGTTGATGTAAGTTCTTGCAGTTAAATTTATTCCTATTGTGCTT
GTCCTAATTTTTTTCATATTTATATCATCCGCTAGTTGATTACAATTTTTTATTTACGCAATGATTGCTCCAGAAGTTCATACACG
TACACAAGTTACTTCTGTTGCCTTATGTTAATGTGCGACGAAGTGCATTTTTAGAGTAACACACCTTATACTAAATTAAAGTGT
GAATTGAGTTTATCCATACAAATTGTTGTGTCCGAGTCCTAGGCCCTCAAGCTACAAATTAAAGGGAGATCATCAAAATAAAA
ATTGTGTATGCCAACTCAACAAACATTTTTAGCCTTTTTGGTTCAATTATGTAATTTATTATGCTTCTCAAGGAAATACAGGATA
ACATAATCATCAAAATGTCTGGCGAGGCTGAGTTCACATGGCAACTCCCTAGTTACCAAGATAATAGTCAAAATATTGATGAT
GATGATGATGATGAATAGAGGTGTTAAATCTGCATTCTTCCCCTTGTTGCCGGGGTAAAATTAGTTGAGTAAAATAATTATAGA
GGGGTGTTGGCTTTTGTTTTAGATAAATGAAGAATTTATAAAAAAAAATAAAATTCAAAACAAGTGTATCAGGGATGTTCTTA
TGGTTACAAAGATGATAAAACGAGAGAGGAAGAAATCAACACTTTGAAGAAATCCTGAATGGTTTTGCCTATAGTTTTTCTT
AGCTTCCCTCTACCTTTAGCAATTGGTTTCCACACGTCCAGACCTCCTAAGCTGAATTTCTACCATCTTTTTCTACAATAGGTG
CTACTTTTTCTCGAATGCATTCATTCCTGATCTGATCGTGCCCATATGCTCACTCATTCAGCACAACATCTCACCTATATGTTTA
GTTTGTTCTTTTGTTGGTTTTTTACCACCCAACATTTAGCTCCACGCAACATGCATGCCTTAGTTGTGTGCTAACTTTTTCCTT
AACATTTAGCTCCTAGATATTTGAACCAATTGACAATCCTGCATTTATTTATTATTTTCTTTTATAGGGGAAGGGGTTCATAATT
GGGCTGACTGTTTACTCATTCCCTTTCTCCTCTCTTGTCTGTAGATGGATTACTTTTTTCCTCAAAAAATTATGTTAAGTCACTT
GAAGAATATTTTCAGTTCTTCAATCCTTATTAATCATGCATTGTTTACCTGTTCTTCTGTCTCGTTTAGTGTGTGCAGTGGCATC
CCAACTGCAACTACATTGCTACTGGCTCCAGTGATAAAACAGTTCGACTGTGGGATGTGCAGAGCGGGGAATGTGTTCGGGT
TTTTATTGGTCATAGAAGTATGATTTTGTCTTTGGCAATGTCCCCTGACGGTCGCTACATGGCATCTGGTGATGAAGATGGCAC
AATCATGATGTGGGACCTCTCTAGTGGCTGTTGTGTCACACCTCTTGTCAGTCACACTTCATGTGTCTGGTCACTCGCTTTCA
GGTGATCCCCACTCAACATTTCAGCTTGATCATAGAAAGGAAATCAACAGGTATTTTAGGTTGCTATCAAAGTCTTAACATCG
GTTCTGTTCTTTGTTGTTGGATGTGTCAGTTGTGAAGGTTCTCTTCTAGCATCTGGATCTGCTGATTGCACCGTCAAATTTTGG
GATGTGACTACGGGTATAAAGGTTCCAAGGAATGAAGAAAAGTAAGTAGTTTATCTGACGAACCATCTCTTTAGGAATTTTCT
TTTCTAATGGAAAAACTGAGCTCATATTTTTCACATTAGTAGAAGTGGAAATGCTAACAGACTCAGATCATTAAAAAGCCTGC
CAACCAAATCTGCTTCAGTTTACTCTCTCCAGGTACATTCACTAATTACCCATTCTTTGTTTCACTTATCCATGGTTAGTCTTTA
TTCAACTTTATAAAGATTTAACTGTGTTGTGTTCCGGAGCAGTTTTCTCGCAGGAATCTTCTTTTTGCAGCTGGAGCTGTTGC
AAAAAGTGGTTGTTAGTATGTCAACAAATTTTGAAGTGGCACTTCTGACTTAACACCAAAAAAACTTAATGTTGTGTTGTAC
AATATTTTTTATTTATAAATTTATTAGTAAATTGAACTGAAATTATTCCCTCGGCAAGTTCTGATAATCCATGCTATTTCCATAAA
TGTATGATATATATTGTAAAATTATTCAGAGATAGGATGAATTACTCTTCTCAATTTGCTGCTTTACATCCTCATATAGCTCTGAA
TGCTGCTAGGTTAAAAACTAGATATTATTTTGCCTTGATTATTGAAGTAATGTTACCACTATAGCAA

>VrTAF5-2 LOC106762344

AAAAATTGAGTGCGTTCGCGAGGTAGCAAGAGCGCAGTGGAAGAAGAGGAAGAAGAAGGGGTTGTGGTGTGAGATCAGA
GAATGGAGGATGATAAAATAGTTGGATATGTTACCGCGTACCTGAAGAAGAAAGGTTTCACGCAAACCGAGAAGATTTTCCA
GGAAGAGTTTCAGCAAAACAAAACCAACAATTCCTCCTCCAGTAATTCCCTTCTCGAACCCGACATTGCCAACCACCTCCTC
GCATTCTCTCAGTACTTCTCCATTCTCCAACCTCCTATCGTTACCCTCCGCTCACGCTTCTTTTCCTCACCGCACTCACTTTTT
CCCTCTTTCAAACCCTATCTTTTGCAGATTGGAGAGTGGTCCGGCTAGGTTCCACGACGGCTACAGCAAACTGCGAACATGG
ACTTATTCTTCACTGGATTTGTACAAGGTAACGCGCGCCTTTTCACTTTCCTTTATCCGTTTTTTCTTTTTTCTATTTGGTTCGT
TTTTGTCACGTTTATCGGCGTATTGCTGCAGCACGAGTTGCTTCGCGTGCTTTATCCCGTCTTTATCCACTGCTTTATGGACCT
CGTGGCGAAAGGACATGTTCAGGAAGGTAATTTGCCGTGTTCTAAATGGGGGCGCTTGTGCGATGCCGCAAGTGTTTTGTTT
TCTTTTGGTTTGTTCTTGTGTTTGTGTGTGACTGTAGTTGCCGTTTTCAGCTCGGAATTTCTTCAATACTTTCCGTGAAGACCA
CGAAATGATGCACTTGCGTGATCTTCAGAAGTTAGAAGGGGTTCTTTCTCCTACCCATCTAGAGGTTGGTTGCTGTCTAAAGT
TTTGTATTGGTCTTTTAATTTTGTCGAGGTTGTTGGATAGTTAGCAATTAGTGTAAAATATTCCTACGGTAATGTAATTTCTTTT
GCAGGAAATGGAATTCGCTCACTCGCTTAGACAGAGCAAATTCAACATAAAGATATGTGAGGTAACGAGTTGATTCTTCTAG
ATGTGTTTATCAGCTGCGCTGTAATTACTTTCTATTTTTATTTTGCACACATTCAAATTGAATCATCTCACTCAACTACTTTCTG
CAACCTTTAGAACTCTAGTTCCCGGCCCCCTCTGTGGCTGCTTCCCTTACTGTTTAATGACGATTACGTAAGCTATTGTTTGAA
CCATTATTTGTGACGCCTTTTCTCCTTGGGGGTTTTCAGGTCATAATGTATGTCATGTTGGGTCATGCCTTTAACTTAAATGCA
AATCGGTTTATAGCAGTTTGGTTTTCATTCAGCTTTTAAGTTGTTGTAGGTCTAACTCTCCGTTGCTAGTTATTCCAATGTGCT
GGTTTTGTATGCTGGGCCATGGGGATTTGTGATACTAGTCAATTGCTTAATCTTATACATTGAAATTAATGTGTATAAGATTTTC
ATCCTTTCATTTTATGTTGTGGACAAACTTATTGCAGACAGGAAGTGCTTTTATGTTGCATCCTGTAGTTGGATATAATTATACT
AAACTATGACCGTACCAGCATACAAATTAGAAGTCAATCTTAACCTGAAACCCTCTCAATTGCTAATTATAGTCAATGTCCCCT
ATCTGTATGCACTGCATACTGCAGGGATTGTGATTTAGGTAGCACCACAACCAGCATACAAATTAGAAGTCAATCTTAACCTG
AAACCCTCTCAATTGCTAATTATAGTCAATGTCCCCTATCTGTATGCACTGCATACTGCAGGGATTGTGATTTAGGTAGCACCA
CACGAATTAGTGGTGGCAACTATTCATCATTTTGTATAGTGTCACACATGTTCAAGTGTGGAGCTTTGGAATCAGATAACTTAT
TGATTGGTAGTGAAAATTTGGGATGTATCCTAATTTCTAATCTCTGACTCACTTGGCTACCCAGCATTTACAGTGGGCATTTAT
GGTATGCCATTACTGTTGATTACCTTATCTTGGTGGATTTGGCTCATGCAAGGGCTTAATGGAGGTATCATGCAGGTGTTAGGC
TCAGGATTGGTTTATATTTTATATTATAAATATTTGAAATTTATCACTATGTCTCTGGACCATGTTCAAAAGGATGACTTATTTTT
CTTCTGAAGCCCATGAACCATCATTATCTTTATCTACCTTGGTGCTTGTTCCTTTTAACTTTTTATCACTGGTCTTTATGTTTTTC
CATTAAAATGGAGCTAAATATATGGGCAAAGTTGATAGCAGTTGATGTTTTGCCAGTATTCCTATGAGCTTCTCTTGCAACATC
TACACAGTACTCAATCCACCACTATACTCGGTATTATAAATGAGCATATTAACTTTCAAGGTACTTTTATTCGTTTTAGATTGCT
TCTCTACTCATTTGAACTACTTATTATTGCGTACACTTCATTGTCTGTTGTTGTAATATAGTTTCCCCTGGACAACCTAGCTTAA
TTTCTGATGATCCGGAAGCTGTTACCCTTACTGGAAGCAGCCAGGAAGCAGCAAACCGGATAAATCAAAAAGAAATTCATTG
GGGGGTGAGTCTACTAGTATTTCTACTAATTTTGATAAAGTTACAAGTTTTCATGGTTATATTGTTTCTGTCATTAGAATCCTAT
TTATTTACATTTGCAAGTTGGTCTAATGAAAGTGTCGTCCTAGCGTGAGAAACACTTGAAACTTCTTGAAATGCCTTGCTTTC
ACCAATAACATCCAAGTATTGCTTATTTTCATTTTGTTTTCTGTTTTCAAAGATTTGAACAATTAATGGTGGATTGTGTTGTTTT
TTTAGAATTTTTTTATACAAATACATTATTTTAAAACAAGAAATAAAGTGTAAGCTCAGTGACGTGACAGCAGTTTTACATGTT
CCTGGACACTTGTCCAAAAGGAGCTCTTATTCTATTTTCAAAAGAACGGAGTTACTGATTTAAATTATTATTGACTGATTCAGT
AACTATCTGATTTAATTCTCGATGTGTATCTCCTGTAGTTACTTGAAGATTCTCTTGAAGAACGGCTAGAGAAGGCAGGGACC
TTGCTTTCAGACTCGGAAAAGGGTGAAGGGGAAACAAAAGAGGGGGAGAATGACGAGACTAAGGTATATCTGTTATAAAAT
TCACATGACCCTGTCAGTTTATTAAATTTCACTTTTGCTTATATTATATGAGGAAATACGGCACCGTTTATTAGAATGTAGGCCT
TTCAAAGAGTGTGTGTGCATGAATTTTAATTTGGGATGTCTTATTTGAGTGTTTTATCACTTTAGTTTTCGAATGTTTTTCTTTT
AGAAAAGATCCATTGAAGGAGGAAAGCAAGGCGGTTCAGTAAAAAAAGTAAAAAAGGACAAGGGTGGTAGTACAACTGG
AAAGAGTGCAAAACCTGAAGCTAGTACTGTACCTGCAGCTCCTCGAGTTAAGCCTGAACTCCCCTTACCCTTAATGTATTCT
CTCTATCCCATAAGATATATTCATTTTCTTTTCATTAGAAATTGTATAGCTAATGTGAATGGCATAATTCTAAAATAATTGATGCT

ATTAAAGCTGGAGCAAAGGATTCATTTGATTGGTATATGTTATCTAAAAAGGATTTTTCTTTTATTGATGATTTTTCTTTAGTATT
TGTAACTGTCATTTTTGTTTAACCATATAGCCAAATAAAAAAAAAAATCAACATTTAGTCATTCACATATCATCGAAACTAAATT
TAATACGGCTACCTTCCTGTTTTGGTTGGAATAAAAATTATTCTTATAATTTGAGTCCTTTTGCTTACAGAGTTTATCAAAATTC
TGAGACTGGGCTCTATTGCTTGCAATGTTTTGCATGACATCTGCAATTAATTGAATGGAACCTAAATTAATGTATATCACTGAG
TGAATTTATATTTTTAAAATTATAGTCAGCTTCTAAATTAGACTGAACTCTAAATGGTTTGTTTGTGATAAACATTTGCTGGGTC
CCGTCAGTGCAGTTTTTGATTTGTTGTAGATGTTTTAGCATTGTGTTACTTTATTTAGCTCTAGGCTTAATGCTTGTTCTGGATC
AGACAGGAAGAAAGTTCAACCTGATAAAAATGATATGGAGGTATGGGCATTACTGCTTTTTGTATTTTGGGGGGTTGTAAAAT
GAAGAAAATCTATTAGAAAGGGGGTGGGGGGTGACATCAAATAGTAGGAGGATAAGAAACCCTGCGAAAGAGAAACTCTA
AATACAACCATAAACATGGAGAAGTTAAGAATTCGGATAGGTTTCCGAGTTTCTCTGTGAATGCAAGCCTTCCCACTATTTTG
ATCCTTTATTCTGTGGAAATTTGGTTGAAAGAAATAGAGAATCGAGATCAGGTAGAAATTTCCCATTTAGTTTGCTTGACACG
ATAGAAACGAGCAAGAGAAAATTTTCACACATATTCTCGTATGGAAACCTTTTTCTCATTTCTCTTTTTTAGTGTGGACTCTAA
ACTCTTTATTTCTATCCTTAATTTCTCCTTGTTTCTTAAAAAAGAATAAAAGTGTATACATTTCTTATTTTTTCTATATATTTCTCT
TCATCCAAACTAAATAGAATTTCTCTTTTGTATTCATCCAAATAATTCATTCTTTTCTCTATATTTCTATTCATAAACGACAAGAT
GTAGGAGGGGAATCCTCTACTTTAAACGGGAGTTCCAAGTCTAAGACTTGGTTGATTTGGGACTCCTCTATGCTGCATCAATC
CTGTTTAAATGGAAAGAGTGTTCATGTACTATGTGCAGATGATCCTACACTTCATGTACTATGTGCAGATGATCCTACAAATTC
AAAGTACATAACTATTTACCCGTTATTCTATTTGTTGATAAAGTCTTTAGATCCCGCATTTCTCATAATTTGAAGGGTAAAAGG
GCCGTTTTCATTTGTTTCTATCAAAATCCTAAAATTGTAGTTGAGTTCTTGAATTAGTTATTCAGCATATTTATTGGACGAGAAG
TACATAATTTTCTAAATTCTCCACTTCTGTATATTTTGTTATCATATGATGCTAGCTACGTAATGCGAAGTATTAATATTACTAATT
GATGTAAATATTTGAATCACCAAATTCTGAATTTTTTGTAACTTCTTTTAACTGAATTTTGTAATGTTGAATTTTGCAGCCCAAC
TGAGGTGGAACAGTCCATCCTTGAAGATTTAAGGAACCGTGTACAGCTCAGCAGTGTTGCATTGCCGTCAGTTAGCTTTTAC
ACATTTATAAATACACATAACAGGTATTGTTCGACACATCTACTATTGTTATCTATATATTGTTTGGATTTCTGTTGTCCAATTCA
TTTTTGCATACCATCTCAGTTTAAGCTGTTCATCGATATCCCATGACGGATCATTAATTGCTGGAGGATTTTCTGACTCATCACT
CAAGGTTTTTTTCTTCTCTATATTTCCTCTTCCATTCACCTCTAGAAAACCCTCTTTCTATGAAGTTCTAAATTGATTGAATTTC
TGGTAATTCTGATTTGTATACCCAGGTTTGGGATATGGCAAAGCTTGGACAACCGACTGCCAGTTGTAAGTTTGTATTTGTAA
ATTTTTTTTGCTGTTACTGTTGCATCATTGCTTGAGAAATGTGAACATAGTTGACCAGTTACAATGGTTCAGAGAAAATATAGT
TTAAGAATACACTGATGATATCAACCTGTAATTAATTTAAGCAAGACATTCAATGGTATAGCAGTTATACTTTTCACTGCCTCAT
GTATAAAGAGAAGAGAGTTTCTTAGCAGCAGTACCATCTTTCTAGTCATCTAGTGATGGAATTATTGACTTGGACTTTGTTAG
TTTATGAGCATTTAATTCACACAGAAAAATAAATGGTGGTTTACGAATGTGATCCGCACCAGTGTGTTGGCATTCTATTTCAA
AATGGTTTAAGAATGGATCAATTTTCTCCCCTCTAATTGTTAAACGCTCTACCCTGGTCTCTTACAGTAAAATTGAGATTTGAG
TTGAATGAACAAGGCAAACTTAGGTGGACGAAAGCTCATGCAATTTCCTAGCATTTGGAGAATGACAAAAATGGTTTTTCTA
GCTGTAAAAGTCTAGTTATCAATTTTTCTATCATTAGCTCTTTTGCAGGGTGAGAATGACACATCACCAAATGATCAAATATTT
GGGCAAGGGGTTGGGAAAAGACAGTATACACTATTTCAAGGTCATTCAGGACCTGTTTATGCAGCCTCTTTTAGTCCCGTGG
GTGATTTTATCCTTTCATCCTCAGCAGACTCAACAAGTAGACTACCTGATCTCATTTTTGTGCCGCATTCTATTTTTTTTCTTTT
ATATTATAGTTTTAATTCTGAACCTTATGCATCTTGAATTTTTATGCAGTTCGGTTATGGAGCACCAAACTTAATGCCAATCTTG
TTTGTTATAAGGGTCACAATTACCCTGTCTGGGATGTTCAGGTAAAACAATTTATTGTGAACTTTTATGTTCTGATAGTGAATG
TAGAACAAGATACCAGTTGCATGAATAAAAGTTTAAAATTTAAGCATTAAAATATGATGCCTATATGAAAGTTAGACCACATAT
CTTGTTAATGTGATCCACATTATTTAGTAAAGTAAATACAAGAGAGGAATGAAGAGCCTAAAGAGATGGCTTAAGAAATGAC
ATCAATGTAGCAAAGCCTCCCCTATCTTTTTAGCATAATATTTATGAGGGAAAGCCTTTATAATCCCACCCTGTTTTTCTTGCAT
GTTTAATGTGGTATAGTATGTAAATGATACAAGTTAGTTCTTTTTGGCCTCTGTAAATTACTTCATAGTTTTTACTGGTAAAATG
TCGTGAAACTTTTGTCTGTTACGATTAATAAGTTGTGTATATGACGGGCATGCATCTATGGGATTTGCTGAAGAGTCAATGCTG
TGCTTTGTACCCTCAAATTAATAGTTGCAGCTTGCAGAATTCTGCTTTTTAGTCCAAATATACTTTATTCCTCTTTTTTCTTGCC
TTGGGATGTCATTCTTGGCTGCATGTACATTCTGGATGGCTGGATTATTTATTTCTTTATATGGATGCAGTTTAGTCCTGTAGGG
CATTATTTTGCCAGCTCTTCACACGACAGAACTGCTAGGATTTGGTCAATGGACAGGATACAGCCCTTAAGAATAATGGCAG
GGCACTTATCTGATGTTGATGTAAGTTATTGCATTTAAGATTCTTTGTTGCTTCTGTTTTTTTGTAGAATTTTTTCTCTTTTGTGA
TCCATAGTTGGCTCTAGCTTATTTAATTTTATATTTAAATATCGATTGCTTCGGATTCATATACAGTGATTTAATTAGGTGTGCAT
CTTTAAGTTTATGATCGAGCAGGGCTTTGTGAAGTCTGTTTTGAATGGGGGAAATCATGTTTTGTTTCTCTTGTTTAGTCATAA
TAGTGTCATTGAATTCCTAACTGGTTATATATGTTCAAAATATTTGTCTCAGAAAACTATATTTTGCCACCTAATCTTAGAAGGT
AAAGAATGAGCTATACTCGTCATTATGTATTTAAGCCACAATCCTTACGTATTTTGTGTTCATTTTTAGTGTGTACAATGGCATG
CCAACTGCAACTACATTGCAACTGGTTCCAGTGACAAAACAGTTCGACTATGGGATGTGCAGAGCGGTGAGTGTGTCCGGG
TTTTTGTTGGTCACAGGGGTATGATTTTGTCTTTGGCAATGTCTCCTGATGGTCGCTATATGGCATCTGGGGATGAAGATGGCA
CGATCATGATGTGGGACCTCTCTAGTGGCCGCTGTCTCACCCCTTTGATTGGGCACACGTCTTGTGTCTGGTCCCTTGCGTTC
AGGTGATCCCTTATTGCTGTATTTTATCTCAATTAGGAAGTGGAATAAAGGTTTTTGAGCTTGCTATCAATTTGGTAACAATAC
TTACGGTCTACTCATAATGGTTGGATGTATCAGTTCTGAAGTTTCAATTCTAGCATCTGGATCTGCTGATTGCACTGTAAAATT
GTGGGATGTAAATACGAGCACCAAGGTTTCAAGGGCTGAAGAAAAGTAAGTTCGTCTGTTGTGACAATTGTTTTTGAATCGT
TTGTTGTAGGAAATATTTTATTTCTAACTAATCCAAGTTCTTGGGAAAATTTAAAAATGTGTCCGGTTATTATTCAACTTCTACA
GGAATAAATAATATATTTGCCACGAGGTATCATTTAGAGGTGTATTAACACTCAATTATTTTCACTTACCCGTTGTTATCAGAAC
CTGACCGAACCGGCTAGTTGATTCAGGAATCTGGCCAGTGTTCGGTCCAAAACTTTTTAATATCTGGATTTCATTTTAATCGG
GATAAAACTAGAAAAACCCACTGTTTAAGATATTTTGGCTCTTGAAGCGTTTTTTTTTTTTTTTTTTCTTTTCTGGTTGTAGC
TGGGTATTTTAATAGACTTGTTGCTTGTAATGAATTTTATTTGTTATAGATTTTAAGTCTGATGTGTCTTAGATTTGTTTCTTTTT
ATTTATAGCATATTTGTTGTATGTTATGATTTATGACACTTTCAAGTAAAATTTTTTTTTATAAACTTATGTCTAATTTTTTTTATG
TTGAATTTTTGAAACATGAAATCCATATCTTCCGTTACGTTGGTAGAAGTGGGAATGCTAACAGACTTAGATCGCTGAAAACT
CTGGCAACCAAATCAACCCCAGTTTACTCATTGCGGGTAAGATCACTTGACGCTTAATTACATGATCATGGATAATGTTCATAT
AATTTTGTAGGATTCAATCGTGTATGTATGTTGTTTGAAACAGTTTTCTCGAAGGAATCTTCTCTTTGCGGCTGGGGCTCTAGC
AAAAAGTGGGTAATATATTTGAGTACGTCTACTAATAATAGATAGATCAGATCAAAATTGTAGAAGCTGAATTTATCGCCTAAA
AAGCAACGCACATCATAGTTTTGTACTCCCTCGTGTCCTTTATGTACGGAATCTTCACAAGGGCTGAGAAGTTCCGTTAAATT
TTAATGATACTATATTTGTCAGTAATCTGTATATTTTTTTTATAGTTAATTATAAATTTATTCAGACATTATTCTCTTTTCATTTGAT
TGATGTAATTCCACTTCAGTTATTACTA

>VrTAF6-1 LOC106758550

AGGGTCTACTGTGGACGTTTGTCTTCCTGCAAACACCTTCACGACATAGAGCTTCCCACCGCACCACACGCGCCAGTGTTGC
GAATTGGACTCCAACACCGCCCGGAGGTAACGGAATTCGAACCGGAAGTTAGAGAAGGCAACGAAGGAGTTGGGGAATCG
ATGCGATGATGAAAAGCCATGTGCATTAGGGTTTGCGTTTTGCATTCAAATTCCGAACACTCTTTCAGTTTGAGGAAGCGAC
GAGAGTAGCGTGCGTTTGAAAATGAGCATTGTTCCCAAAGAAACGATCGAAGTTATAGCGCAGAGCATTGGGATAAACTCCT
TGTCTCCCGATGTCGCTCTCGCTGTCGCTCCCGATGTCGAATACCGCATGCGCCAGATTATGCAGGTCTCTTCCGCTCTTTTCT
TTTTGCAGTTATCAACTCTTTTTTTGAGTGTGTTTCGATCTGTAGTATCGTTAATTTTAAGCGATTAGACGAGTGTTGTGTTGA
CCTAACTCCTTACTCCTACCTCTTAACACTGTTTGTGCTAACCTCAGGAGGCAATTAAGTGCATGCGGCATTCTAAGAGAACC
ATTCTCACTGCGGATGATGTTGATGTTGCTCTGAATTTGAAGAATGTTGAAGTAAGTTTGAGTCTTAAGTGATTAAATTTTGG
ACTGCCAATAAAAAAGTGTTCGGGTGGTTCTTTTTGTTTTGAGGAATGCTTTCTTTTTTCACCATTAACTTTATAACCATCCAC
GAGTCGTTGAACTAAAGTAGTTTGTTTCGATCAAGTGAATCAAATGTGTCTTTGTTATCTTTAAATGCAAAGCCCCTAAAGTA
CAAGCACTATATTAGTTTTCCTATTTTTGTTTTATCTTCTGTGGTTCTTCGTATATATTGGTTATATTAGATAATTATTGGTTTATGA

TTCCATATATATATATATATATATATATATATATATATATATATATAATACAAAGGTGTGTGATTATTTAATTGTCCTACACTTATGGCCAAA
AAATGCTTTATTATGGGTATTTTGATATGTGCAAAGATAAGTTCATAGCAAATTGATCTTCATTGTTCAAACTGTTCAGTAGTGT
ACATAAAAGTTGTGGAAAGATAAGATATAACAAGCTTCATTTATATTAGAACTGAGAATTGACTGTGCATTGAGAGTAGTGAG
GATTATGATTTTATGGGTTCTTGCTATTTCCAGATAGAAGATGCATATTAGAACTACCTTGAGGAAATTTTTGGTGAATTTCTTT
TCTTTTTAAGAAAATAAAGCATAGTTGTTGTCTCCAATCTCATTATCGCTGTGTTTCGATCCCTTGTGACAGCCAATATATGGAT
TTGCATCTGGTGGTCCTTTGCGGTTCAAAAGAGCTGTTGGACACAGGGACTTGTTTTATATTGATGACAAGGATGTAGATTTA
AAAGATGTAAGGGTGTACTCAATCATTTCCATTGACTCACTTCAATTGGATTTCAACCTGTGCATCACCTCGTTAAAATTATAT
AGCTTCTGTTNATCATAGGTTATTGAAGCTTCTTTACCAAAAGCACCTCTTGATACTGCAGTTACATGCCACTGGCTTGCCATT
GAAGGTGTGCAACCTGCTATTCCAGAAAATGCTCCTATAGAAGGTATGTCTCTCACCTTGAGTTCTACCTGTATTCTTTGGCT
CAGATGATACTAGGTTGTATGGTTTAGTTGCATGGATTGAGGAATTTTTGGATGTATGATTTNTTTTGATGTTATACTTGTCAGC
TTGTCGTTTTGACTTCCTCTGGTTCATGATATCATGAATTTTTACTCTAGTTAGTGTTTAGATTGGGAGATGTTATTTAAGTGAT
TTTTTTTTTGTCACCATAGCATGGTCAGCTTTGTGGGTCTAAAATATGTGGAAAACATCAGTATCTTTATGCACACTAAATCAC
TAGAAATTAGAAATGGAAAACTAGAAGAAGTATACACATCCAATTAAATATACTGATTTTCAGATATATTTAGTTATATGTGTGT
GTGCGCCCCCCATAGAATCATATAAATATTTTGCTCCTTATAATTGTGGAGATGTACATCATACAAACAAAACAAACAGTAACT
TTTTGGCTGCTTAATGCTTCGAGATCCATTATTTGTAAATTCTTCCAGTTTAACTGTAACTTCTACTCTCCTAATACCCCTTTTA
AGAATTATTATTTACTTATTATGCTATATGCAGTAATTTCAGCTCCTTCTGATACCAAAAAGCATGAGCAGAAAGATGATGACC
TTCCAGTTGACATCAAATTGCCTGTTAAGCATATATTATCCAGAGAACTTCAGGTATTAAAATTCTTTAGGCACCATGAGCTAT
CTTGTTTCACATATTGGGATTCATTTTTGTTAGTTTTATTGTTTCTTACAGATGTATTTTGACAAAGTTGCTGAGCTTACTTTGA
GTGAGTCTGATTCAGCTCTCTTTAAAGAAGCATTAGTAAGTTTGGCTACTGATTCGGGGCTTCATCCACTAGTTCCTTATTTCA
CATGCTTTATAGCTGATGAGGTAATAATATTTGTCCTTTTANGATGTTAATGGATAAAAAAAGAATTTTCACCTTATTGTAGCTC
ACTACAACTGCATGCTTCTCATTTCTTGTCAGGTTTCACGTGGTTTGAATAATTTTCCTCTTCTATTTGCCTTGATGCGAGTTGT
TAGTAGCCTTCTGCTAAACCCTCACATCCATATTGAACCTTATGTAAGTTCACTTGTTGATTTAGTATATTTGTTTATATTCTTGC
AGACGAAGTATGATCTTGTTTGAGCACCGTAGTATTTTTAATATCCACGTAAATATTGGTTGTTTCATCCTTTTCTATTTGGTAG
TCTGTTGTCTACATTGAACTGTGAAAGTATTCTGAGATATTTAAGTTGATTTGTCATTTGTTGCTTGTCCATAAAGATGGCCCT
GAACAAACTTGGTCGGTTGGCCCCATAAAAATTTTAAAACAAATAAAAAAAATGTGCTTTCTGCTGTATTTTCTTGTGGCCTG
CCGCATCAGCCTCAAAGCAAAGGTCAGGCTTGGGTTGTGTTTTAGTATGGTCTTTTTCTGGCCAGATACAATGGTTTAGGTTG
GTAGTCCAGTCTATTATTTGCTAGTTCTTAATGGCTTTTCTTATCTTATAAGGTAGTTACAATGTTTATTTTATAATTTGTAGAAT
GTATTGTTTGAATGATTATGGAGTGTTGTGAAAAGAAAGTAGCATGAATAACTAGAAAAGTTTTTCCATATAGGTGGGTTTAG
TCACACGGATCAAACACCACCGTGTTTCAGAAAGGCCATTTGGTTATTGCTCTGTTCACTTGAACGAGTTTATGTGAAATCCT
CTCAACTCTTTTCTTTCTGTTTGTGCATGTTTCAGCTACACCAGTTGATGCCATCTGTTGTGACCTGCCTCGTTGCTAAAAGGT
TGGGCAGTAGGTTGGCAGACAACCACTGGGAACTTAGAGACTTTACGGCTAACCTGGTTGCCTCAATATGCAAAAGGTGAG
ATATCCCTCTTGCCTTATTTATTTTGTATATATGGAAATAAGCAGTCTTCTATATTTATTGGTATGTGTACCTCCATGCTTGGCTAG
TACAGTACGGTATCCCCATTTATTTGATGGTTGTTGCAATTTGTTCACCTTCTTTTATTTCCTAGTAGCAAAGAGAAAGAATAC
TATATTTATTGTGTATTTCCTTTCAAATTCACCTATGTTTTTGGAGATCCAGTTCAAGGACACACTTCAAATTTATTGTGTATTA
AAGCTTATGCCTTTTTGCTGATTTATCAAACAACTTCATGGCATGGTCATTGGTATTTCAAGATGTAGAATTTGGTACAAACTG
TTGGGTCATTGTATTGTAAGCTCGAAAGTCTAGTTTTGCTTGATGGCATGTGGCTTATTTGCTGGGCTTTTATTGATTTCATTTC
TATTTTTCTTACATTAACTTCATGTTTCATGCAGGTTTGGACATGTCTATAGTAATCTCCAGTATCGGTTGACTAAAACATTGCT
GAATGCATTCTTGGATCCTAAGAAGGCAATGACGCAACACTATGGAGCGATTCAGGGGTTGGGGGCACTTGGACCCAACGT
GGTATGTGTCTCTCCCATTTTCCTGGTTATTGTGGAGTTGGTCAAAAGCCAACAGTTAGAAATATACTTTTGGTGGTTTAAAG
ATGACAACTTTCCAATGCAATTTTTATATACGCTTGTCATCATCTTATCATTAGATTAACATGCTATTACATTACAAATTCAGTAT
TGTTGCGTATTTATAATTTTAAAGTTTAAATGACCAATTTAAAAGGAACCTTGTATGGTATATTATGTTTTATTTGTTTAACTCAA
GTAGGTTTGATACTTACAGATGGGGCTGTGATATGTTTTTAATGTTACTTATTGCTATGGAAATAGGTTCGCCTTCTTTTGCTGC
CAAACCTTGAGACATATATGCGACTTCTTGAACCAGAGATGCTTCTTGAGAAGCAGAAAAATGAAATGAAAAGGCATGAAG
CTTGGCGTGTTTATGGAGCCTTGCTGGTAAGATATGCTGTCACATACAGCCACTTTCTAGTAAAAAATAGGTGTTTTTCCAAC
TGAAAAATGTCAGTCTCAATATAATGTGGTTGTTCTGATGATTGTGTGTAATCTATCATTCAGCGTGCTGCAGGTCAGTGTATA
TATGATCGACTAAAGATGTTCCCAACTTTTTCAACTCCTTCTCCTAGTGCTGTCTGGAAGACCAATGCAAAAGTTCTTACTTC
TTCATCTCGTGAGTATTTTCTTTGTCCCCCTTGAAAATACTAGTCACTTGTTCATGAAATACCTACTATAATCTTTTTAAGCTTT
GGAGGGAAATCTTTTTTGTTTGGGCATTTAATTTAATATAAGGTAAACAAGTATGTATGTGTGTGTGTATATAATACCCACAAA
AGAAAGGGATCTGTTTGTCAATATTAGCATCTAACTGCGGCAGGGTTGAAGCAATTGTTAATTGTGCTCATGATTGTCTCTACT
AAATCATACAGGCACATAGCATAGACATAAATTTGATTTAAGTTCAAACCTAAATAAATTCATGCATGGAGGATTATTATAATT
GAAATTTAGAAATAGAACTGTTAAATTAAAACGAATTGCTTCATCAGGTAAATTGACAATTTGTGTTCGAATATGTTATTATCC
TCGAAACTTTTCCTATTTCCTCATTGTCACTTTGGATTTCTTGTTTACAATATTTCCTTTTCTTTTCCTAACTGTTTCTGTATATT
AGCTGGGATTATCTGTCACAGCAGGCACCAGATCTGTTACTTGGTTGTTATTTTCTTAAGAAATTCTTAATGACTGATGTACTG
GGAGGCGCTGACTTAACGATCAATGTTCTAATTATTATATAAATATTGTTTATACTTTTCCGTTCTTTGTTTGTATACTTACTCCT
TTTCATAATTGATTATTAAGGTAAACGCAAGGCAGACCCTGACCAATTGGAACAGCAGCCACCTTTGAAAAAAACTGCTACC
GACGGAGAGGTTGGTGTGGTCCCAATGAATTCCTCACCCGCTCACAAGCAAGAGGAGGCAGAGACTCGAGCTTCTTCAGTC
GATTCAATTATTGGCTCATCATCTTCTGCACAGATGAAAAATGAGACTTCTTTAGATGGCGAACTTAGAAGTAACAAGGGTGA
TACTCAGGCATCGAAGACATCTGCTGCTCTCACCCAGGTTTGGAAAGACGAGCTTAATTCTGGACGGATTCTGGTATCACTG
TTTGACTTGTTTGGTGAAGGAATTCTTTCCTTCATTCAGGCTCCTGAGATGTATATGTTCTTGTAACTTTACAGTAATTTGATG
AATAGAGCATGGATAATCCATTGACTATTAATTCGAGAACAAACTAAAATTCCTCCA

>VrTAF6-2 LOC106775101

CAGCCATTGATCTTTCACAAAAGAACCTAACACTAACAAGTTCATTTCTTTGACCTTCCAACTGTTTCTTAGGGCAATAAAAT
AGTTTTAATTTTTCTATTTTAATTCAAACTATTCTTCTGTTTCTGCAAATTGTTCTGGTTTTGGCATTACCACCATCATCATTCCC
TTGACCTAAGCCACCTTTGTGAATCAGTGAAAGACCAATTGATGTTCAATTGAAATCAAGATGAGTTTTGTGCCAAAGGAGA
CAATCGAAGTCATCGCACAGAGCATCGGCATCACCAATTTGTCCCCCGATGTTGCCCTCGCTCTCGCCCCCGATCTCGAATAT
CGCATTCGGGAAATCATGCAGGTATCCTTTGCAATGTTCTCGCACCTCTTGCGCAATTTTTAAATTTTCGCGGGTAATTTGAGA
CCCCTTTGTTGGTTTCGTCGCGCCGCGATGACCCGGTTCGTCTCAGGTGCTAACGGTGAAACCCACTTTCTAAATCACGTTA
AAAGTTTTATCTTTGAGGGAAAGTAATGAAGTTCCCTTATTTGGTTCTTGAGTTTTTATAAAAAGGGTGTAGCGGTTGGTTGG
ATTTTGATATTGTTGTTCCTTTTTTTGTTGTTACCATTACTACTACTACTACTTTGAATGTGGCTAATTGAAGCATGGGTTGTTT
GTTTTGAATTGGACAGGAGTCGATAAAATGCATGCGCCATTCGATGAGAACTTTTCTTAGTACAGAGGATGTGGATACTGCAC
TTGCATTGAGAAATTTGGAGGTGGCTTGCTTTTCTTTTTATGTGTTTACTTTGGCTTTTAGAATTTATTATTCTGTTTTGAAACT
TTTATGGATTTGTTGACGGTTTATTGAGGTAGCATGTGTTTTTCCTACCCTGTAAACATTTTTCCTCAGACTAGTGTATATGAAG
GTTTTGTAAGTGTATCTATGAAAAGGTACTTATTTGTCTTTAAGAGGATGGCAAATTGGTAATGGTGGAGCTCTTGTGCAGAC
TCTTATGAGAGTAGTGGTTGTGCACAGTAGAATGCCCCATTCAAATCAAGATATTGCTTTCAAATTTTTAAAATGGCAAACAC
TTCTCCATATAATTCGTGTTTCACTATGAAATGCTTATCACCAAGTTTAAACCATCCCTAGCTTGTTGAAAATTTTATCTTTGAA
CTTGATTTTGGTTTATTGTTTCTTATGCTTGTTGTCATGTATCAGCCGATATATGGGTTCACCTCTAATGATCCTCCGAGGTTCA
AAAGAGCTGCTGGACATAAGGATTTGTTCTACATCGATGACAAAGATGTGGATATCAAAGATGTAAGATTTCTAGGTACACCA
AGATTGCCATTCTGATAATAAAAATTGTTTAATTGCTTTTTGATACGGTGTTCTCATATTTTTCTTGATTGAGCAGCTTATTGAA
GCTCCTTTACCGAAAGCACCCCTTGATACATCAATTACCAGTCACTGGTTGGCTATTGAAGGTGTGCAACCTGCAATTCCTGA

AAACGCTCCAGTTGAAGGTATTTTTTCCAGATTTAAGCACAATAAAACTGAATTAACCTCCTTAATGCTCTTAGTTCCAACCA
TGCTAATTCTATGTATGATAATATGATCAATTTTTTCATATCTAGCCAACCTGCTATAGTATTCAAGATTTTAACCTTAATGGTAC
ACTGAATTTTGTATTTTACCAAACTGTATGAGGTTCATGATGAAGTTTGATCTTCCTAACATTGACATATTTATTTGCCCTGCGT
TACTTGCAGCTCCTTCCTCTGAGATAAGAAAATCTGAATATAAGGAAGATGGGCTTTCTGTTGATGTTAAATTACCTGTTAAA
CATTTAATAACAAGGGAGCTTCAGGTATACACATTATGCGGTGTATCAATTGTTTATGCAATTCCATGTTTTTGTACTTCACTTA
AGGATCTTTACTTCTTACAGCTCTACTACGAGAAAATAACTGACCTTACATTAAATAAGTCTGGATCCATTCCGTTCAGAAGA
GCATTGGTTAGCTTGGCAACGGACTCGGGACTCCATCCCTTAGTTCCATATTTCACATGTTTTGTTGCCGATGAGGTGTTGAC
AAGTTTGTCAGTGATGTTTTTGATTAAGGTTCTTAATTCAGTGTGTGAAATAGAGCATAAGCGCTCACTTTTATCCTTTTTCTC
TTGTCATATCAGGTGGCACGTAATTTACATAATTTAGCTGTTTTATTTGCCTTGATGCGCCTTGTGCGGAGCCTTGTGCAAAAT
TCTCAGATACACATAGAACTTTATGTAAGTTTGACACCAATGTAAATGATTTTGCATATATATATATATATATATATATATATATA
TATATATATGTATGTATGTATGTATGTATGTATGTATGTATGTATGTATATATATATGTATTTATACATCGCTGACTTCAGTTGATTGT
TGTCATATATGACAATTGATGAGTTTGATCTCTGATATTTAAAATCTGCAGTTACATCAATTGATGCCACCTATCATTACTTGCC
TTGTTGCAAAAAGGATAGGAAGCAGACTATCTGACGATCATTGGGAGCTTAGGAACTTCAGTGCTAATCTTGTTGCTTCAATA
TGCCAAAGGTGTGGACTTTCTTTCTATTGGATAAGAATTTAAATACCATGGAAGAGATAATTTAATTATGATGTTAACTTTTGC
AGCATGTGCATTGTTTTCAATTTTTCTATTTTTTATGTACAGATTTGAGTAGGAGATTTATTTAGAATTATTTCTTCATTATTTCTA
ATTAAGAAAGATTTTCTCTGATTTGTGGCCCATAAATTGTTGAGGCTGTTAAACTTGCATGTGTTTGAAATACAATACTGTTGG
AGATTCCATATCTAGTAGAGATATGGTCAAATTGTGGTGTATAAGTAGGTGTAAATCTGATTTTATAAGTCGGTTTTATAAGGTT
GAGATAGATTTAAACTTACATGTTTCATATAGAATACTATAGGTATATAGCTGACATTGATACTTTACAGTTTAACTGCACAATT
TGAGCAATGTGTTCTGTAAAACTATAATGATGCAAAGGGCTTCTTAATATGTATATCTAAATGTCCTCAAGATGGCACATACTT
TGGTTTGATAAGGAAGATTTTGTATTTAGCAACTACAGACTACTGAGCTTGCTTTTATTTATTTTTTCGAGTTTTAAATTCCTTG
TCTCTGGAAATTTCAGATTTGGGCATATTTATCACAATCTGCAGCCACGTGTGACAAAGACATTTCTTCATTCTTTCTTGGACC
CTACAAAAGCTCTGCCTCAGCACTATGGTGCAATTAAAGGGATAGAAGCTCTTGGATCAAGAGTGGTATGTTCTGACCTTGA
TTTAAATCTGTTGTGGTTGTTAAATTGTTTCATCTTATTTGTTTGATGATCTCTTACGGATAATAACTGAATGAAGACACTATAT
TTTTCCTTTTTATATCAGATATGATTATTTTCTTATACAAACATGATAACTTTTACCATCTGTTTAGTTGTTGTGGACAATAATGA
AGAACTATCACTTTTTTTTTAATATGCTGTTACTAAATATATGCATTTTTATTGCAATCTTAATTTGCATGGGGTGCACCGGTGA
CTTTTATGCTTTATGATTTAGAACCTCTGGTTGTATTGGTGCAAACTAATGCTTACATTTCTTCACTTTGAGTAGATTCACTTGC
TTATACTTCCAAATCTCGAGCCATATTTGCATCTTCTTGAGCCAGAAATGCAACCTGAGAAACAAAAAAATGAAATGAAGAG
GCATGAAGCTTGGCAGGTTTATGGAGCTTTGTTGGTGAGTTGTGATGGTTTAATTATGCATAAATTGGTTTGTTTTCACTGAAC
ATCTCTTTAGGTGATCACTGAAGTCAAATCTTTCAGGGTGCAGTAGGCAAATGTGTGCATGAAAAGGTAAAAAATTTCAGCA
ATTTGTTCTCTCTTCCAACTCGGCTTATTTCAAGAGGCAGTGGAAAAGCCATTATTCCAATGTCAGGTATGACTTTATTTTTCT
ACCACCGCAATTATATTGATGTGTTTGAAGAGAAATACATGCTTGATCTTGAATGGAGTAATGAATAAATTTGTTTGTATGCAT
GCCTTATGGTGATAGCTATATGATCTCTCTCTCCTTCTCAAAGTTAACTTTTAGTCCTAACAGCCAACACAGACATAAGAAGG
CCAGTGTATTTTTGAAAACATTTTCTTTTTAATAATACACAAGCTAGTTTTTGAAACGTATTGGAATCCCTACACAGAATAATT
ATTCAATAACCATTTATAGTAAATAATATTTCAATATGTTACTGCTTTGAACAATATGTACCAATGAATTTTTAATATTTTGTGCT
AATAATATTTTAATGTATTATTCCATGTTTGGGATAAAAAGAGAGAGAGAAAGGATCAATCTTCTTGTTTCATAGTTAAAAAAG
GGATTCTATATAAATTCCAAGATGATTGATTCTACCTACTGTTACTGTTACTGTTACTGTAATCAGAACACAATCTTTACTTTTG
TGCTCTTATAATGATCATACTCCATCTTTACACTAATGAAGGCAAACGAAAAGCCAGTGCCGACAATCTGATGCAGCAGCAA
CCTCCAATGAAGAAACTTTCAACAGATGGCCATGGAGGTGTAATACCAATGAACTCCATGTCAGTTGACATGCAAGGCTCAA
CAGGGGGATTTTCCACCATGATGGGGGCTCCTGCAATGTCGATGACTCGTCAAATTTCAACCGACAAGGCGTCAGGGAGGG
AGGTTGTAGATCAACAAAAGAAGGTGTCTGCCACTCTTGCTCAGGCTTGGAAGGATGACATTGATGCAGGAAATTTGGTGT
CATCAGTGGTTGAATTGTTTGGCGAAAGGGTGTTACCATTTGTTCCAAACCCTGAAGCATTTATGTTTTTGTAGCACTAGAGT
TGCAGTGTAGGTATTTGTTTTTGCATGTGAAAGTGCATGCAATAAGGTGGCATGCCTCATATGACTTGCTGCAGAATATAAGG
TACCATAGGATAGAGTAGAAATTAACCTGTATTTAAATTTGATATTAAAGTCATGGGAAATGCATCAGAAAAGCTCATGAAAA
AACCA

>VrTAF6-like LOC111240733

ATGCATATTAGAACTACCTTGAGGAAATTTTTGGTGAATTTCTTTTCTTTTTAAGAAAATAAAGTATAGTTGTTGTCTCCATTCT
CATTATCGTTGTGTTTCGATCCCTTGTGACAGCCAATATATGGATTTGCATCTGGTGGTCCTTTGCGGTTCAAAAGAGCTGTTG
GACACAGGGACTTGTTTTATATTGATGACAAGGATGTGGATTTAAAAGATGTAAGGGTGTACTCAGTCATTTTCATTGACTCA
CTTCAATTGGATTTCAACCTGTGCATCACCTCGTTCAAATTATATAGCTTCTGTTAATCATAGGTTATTGAAGCTTCTTTACCAA
AAGCACCTCTTGATACTGCAGTTACATGCCACTGGCTTGCCATTGAAGGTGTGCAACCTGCTATTCCAGAAAATGCTCCTATA
GAAGGTATGTCTCTCACCTTGAGTTCTACCTATATTCTTTGGCTCAGATGATACTAGGTTGTATGGTTTAGTTGCATGGATTGA
GGAAATTTTTGGATGTATGATTTCTTTTGATGTTATACTTGTCAGCTTGTCGTTTTGACTTCCTCTGGTTCATGATATCATGAAT
TTTTACTTTAGTTAGTGTTTAGATTGGGAGATGTTATTTAAGTGATTTTTTTTTGTCACCATAGCATGGTCAGCTTTGTGGGTCT
AAAATATGTGGAAAACATCAGTATCTTTATGCACACTAAATCACTAGAAATTAGAAATGGAAAACTAGAAGAAGTATACACAT
CCAATTAAATATAATGATTTTCAGATATATTTAGTTATATGTGTGTGTGCGCCCCCCATAGAATCATATAAATATTTTGCTCCTTAT
AATTGTGGAGATGTACATCATACAAACAAAACAAACAGGAACTTTTTGGCTGCTTAATGCTTCGAGATCCATTATTTGTAAAT
TCTTCCAGTTTAACTGTAACTTCTACTCTCCTAATACCCCTTTTAAGAATTATTATTTACTTATTATGCTATATGCAGTAATTTCA
GCTCCTTCTGATACCAAAAAGCATGAGCAGAAAGATGATGACCTTCCAGTTGACATCAAATTGCCTGTTAAGCATATATTATC
CAGAGAACTTCAGGTATTAAAATTCTTTAGGCACCATGAGCTATCTTGTTTCACATATTGGGATTCATTTTTGTTAGTTTTATTG
TTTCTTACAGATGTATTTTGACAAAGTTGCTGAGCTTACTTTGAGTGAGTCTGATTCAGCTCTCTTTAAAGAAGCATTAGTAA
GTTTGCCTACTGATTCGGGGCTTCATCCACTAGTTCCTTATTTCACATGCTTTATAGCTGATGAGGTAATAATATCTGTCCTTTT
ATGATGTTAATGGATAAAAAAAGAATTTTCACCTTATTGTAGCTCATTACAACTGCATGCTTCTCATTTCTTGTCAGGTTTCAC
GTGGTTTGAATAATTTTCCTCTTCTATTTGCCTTGATGCGAGTTGTTAGTGAGAAAGGAACGAAAGAAGAAGAAGAAGAAG
AGAGAGAGAAGTGAAACAGTGAAGAGATGAAAAAAGTGGGAGAGAAAAAGGTGAGAGATATTATAATGAAGAGTGATGA
AAATAGAAGAGAGAGAAAAAGGTGAGAGAGAGAGGTAGTTATAATTATGTTGTAATGGTGAGAGAAAGAAGAGAAATTGG
AAAGAGAGAAAGATATGTCAGGAATAACAGAGGAGAGAGAAGAAAAATTAACGCCGTTACAAGCTTCTTAACGGAAGGGA
CCTATTGCAAGCAAAATCAAAAACCTGGAAACCTAATTAAATCACTTTTAAAACCAGGTATCAAACGGAAATTCAGCTCCAA
ACTTGGAGACTAAATAG

>VrTAF7 LOC106763916

AAGGATCGAGAAGTGTGAAGAAAAGTTGTGTAATTAGCATGAATAATAAATAAGATGTGATATGGTTCAGGCATTGGAGTAG
CAGCTATTTCTAGGTCAAATTTCTCCCAAATCAGAGAAAGGTGTTAGGGTTTTTCTTCCTTCTAACCAGCCCTTTTCTCTCTTT
TCTTCTCCGCGAAGGACTCATCCCTCTCCAATCGGACCGCTCTTCAACCACGCACTCACGGTGGTAACCACCCCAGAATTAA
GGTATGTCCTCTTGCTCACGAATTTATTATTTGAATGAAGAAATTTTATTATTGTGCAGAAGAATGAAAACGAGCTCTTTTTAA
CCCATAGGCTCAACAGCTAACAAAAAAGAGTTACAGCAAACAACAATTCCTAACTCTATGAAAGAGTTATAGCAGGAAGGC
AAAAGTTTGATAATCATATGAACATATGTATTTTTGTGGCTGCTAGACGTAGTTAAGGTGGCTAATTGAGAAAATTTATTATTG
GGTAGCTAAACTAACATTTTGAAAAACGGGATTGAGATAAAACGAGGATTCCCCTTCAAATGCATTACAATCACTATGCACA
GACTTTGATCACAGGAGAGAAGAAAACTACGGTTAATAGTATTGGTCTAAATATGCCCCAGGATGACGGTAAACTAGTGTCA

TTGTTTTGCCCCTAGTTAATTCGTCCCTTAAATTTCAGACAAGCTCCCTTGAGCCCACAAAAAAACATAGTTTGGTTGCATGT
TAAACAGACTTAGGGCTTCAAACTTGTGAACTCATCTAACTGTGAGTTCACAAGTGAGGTTAACAACCATGTTTGAAACCAT
TTTTCTCCATTTTGTTGATGGTTTGCTTTATTATGTAGACTAGCTTGTTGTTAGGTAATTTGCTTAATACTTATGATTGATGAAAA
ACCAAAGCGCTAATGATTTTATAAAAATATTATGTTGTTTTTCATTTAAGTTCAATGCTGTCTTTTGTTTGTAGGGTGATGATAC
TGAATTATTTTCCACTGTATCTTTGACCATGAAATGGTTATCATTGGGAAGCCTACTTCTGAATAAATTCTTTTGTAATTTATATG
AATGTAACAGTGTTAATTCTCGTTGTTGCAACTGCTACTTGTTGTAGGTTTGCTTTTGACTGTTTTTTGTTTTGGCTTTTATGTT
AATTTTTTGTTCTCTTTTTTGATTCTTCTTGTTCATTGTGCAATCATCATTCTAGCTTATATCCTAAATGAGTTCTTAAAACATGG
ATTACTGCATCTCTTCTACAAGGGCTTAATGAAAAAAATGGTGTACACTCTTTTTTTAAGTCCGGTATCAATATCCTTAGTGGT
ATCGGATATGACAAACTGTTCAGGAATATTACTTTTTGATATGTTCAAGGGAAAAGTTTATGTTAGAAGAAATAAGAGGGATA
AACAGTTATAACTGTCTGTGGGTTGGGTTTGTTGTGTCAGACTATTATAAAGTCTGTTTGGGAGGTTGTTTTCTTTAGTTTTTG
TCATTCTTGGTATTTCTGTAAAGAGATGGGTGAGGAGACTCTGGTCCTCTCGAAAGACCAGAGGAGTGTGGTGTGTTTGGTT
CCTCCACATTGGTGGAATCCTTTGGTGTTGTTCTAATAAAGACTTCAAGATTCTGTGGGTTTATTCCTTTTAGGTCTGAGTACC
TATCACTTTTTAGCTTGTCTTACAGTTTTATGTTGATGTTTTTTTACTTGTTTACTTTTATTCTGCGGGTAGAGAGGGAGTTTAT
CGTTTCACTCAACAAGTTAATAACTTTGTATTAAAATGGTGAAAGTGAAACATACAGAACCGAAATCAAGTCTATTTTATTTA
ATAACAAAGATTTAAATTCTAAATCATTTTTTTAAATTTCAAAACATGGGATTTGGTTTCTAAATCAATTCAATTCCATTATGTG
TTTTTTAAATCAAGTCATTTTACATTTTAAGTTTTTTAAAACATGTCCTAATGTTTTTAGTTATGGAGGAGCAATTCATACTTAG
AGTTCCACCGAATGTGGCAGAGAGAATAGAGCGGCTTTTGAATGAAACTGATCCTTCTTCATCTGAAGACAAGTCATTAGAT
TTGTCATTTAGTGAGGATGGAAGAAGTGGTACGTTTGTGATTGGGAATGAACACTTCCCAGCTTCTCTATTGGACCTTCCTTG
TGTTGTTGAATCCTACAAGACATATGATGATAACTCTTTGATTAAGACTGCTGATATTGGTCAGGTCAGTTGTACATCATTAAT
CAAATAGTTCATTCATTCCTAATCACATCTTTGATTCATGTAACTTAATAACTGTGTGTCTGATTTTTTTCTTGAGTCCAATTAA
CAGATGATTATGGTTCGGGAATCTGGTGATGCTGCTCCAGATGTAATTGAGTACAGGCATGGTCTCACCCCGCCGATGAGAGA
TGCTCGCAAGCGTAGATTTCGCAGGGAGCCCGATCTTAATGTATTATATTCACTCACATGACAAACATTGACATGCCTCTCTTC
CTCCATATATGTATATGTATGTGTATATGATCTCATATTAACATTTTTGTTAATATTTCATTTTCCAGCCCGAGCTTGTGTCCCGTG
TTGAGAAAGATCTCCTCAAAATCATGGCTCGAGGAACAGCTGAAAATATTGATATCCTTAAACGTCTTTGTTGTCTCAAACAG
CACATGAGTGATGATTTCATTTTTTAATCTTTAATCTATGATTGTGCTTTAAATTATTTTATTGAAATAATGGATAGTTATTGAGA
TTATTATTGTATTTTTCTTACCACCCTTTTATTAATTGTTTTCCTCACTTATAGACACGGGGTTCGTGTGATAACTAGAGTCTATG
TTTGCATAAAGGATAAAATCTAGTTTGTTGCCTTCTTGATAGTGGAAACAATCTTCACTTTGGTTATTTCCTTGACTGAGATTC
ACATGCAGAAGCCGCTGAACAAGAAGTGGAGGAGAATGCCCGAGGTGCTAACAAAAAGGTTGCACCTAAGCCTGCACCAA
AACATGATGTTCCAGAGAATCTTACAAATGCAGGGGAGCCTGACAGGAGTGATTCTGAAGAATCTGATGATTCAGTCTGATC
CAAATCAACTTCCAAATAATATTTATATTACAATGATGAAATGAAAGCCTGTATAGTATAACTGCTATTAAATTTGAATTACGTC
CACCCTTAAAATCAACTTTAGTGGATTTTCCATTGTAGGTGTTTGTATATACTTTTGTATATTTTATCAAATGGTGCATTTTCGTG
GCACTTTTTTTTTGGTAA

>VrTAF8-1 LOC106759260

GTTATTCCTAAATAGTTCTAATTAAAAATAAATTTAAAAAAATCAAATAAAAAATAAACTATTTTTTAAATATATAATTTCTTTTA
ACTATAATTATATATTTATATTTTATTATAACATTTAAAATAATATATATATATATATATATATATATTTATTATGATAAATATATTAATTT
TTTAGTGTTATAGTTTACCTATAAATATGTATTTTTAAAAGTTGTGTAACATACTTAAAAGTGTGAAAACATTATGTTGTTTACG
TATTTATGGAAGTTATTTGTGACTAAAATTATAGTAAAAAAACTAAAAATTGCAGTTGAGAAATGGCTTCACTAGGACTAATTT
CTGATTTTGAATTGGAAAACAAAAGTCATTTGGGTTATAAAGGAAAGGGTCACTTTATGAAATTATTTGGAGAGTGGGATATT
TATATAAATTTTTAATTAAAGAAGTATGTTTTTCAAAATAGAAGTCCCAACAATATCATATTAGATGTAAAAAGTCATAAGATAG
AATATATTTATCATCAATTTTTCGTAAGCATGGCGAGAGATGAGAAATAGGTTCTTTTTTCCTCAACTGAAACGACGTCGTTGC
ATGGAAAATATTTATGATTATGTTTTGGGTACCCCCAATTAAGGTTGAGTTAATAGGAGGGTTCAAATCTCGAAACCTCATCGT
GTCCGTGTCCTTAATTCTGCTTCCAAACTTCCAACTCCTTCTGCTTCTAATGGTAGCAGTGTAGTGTATTTCGATCCAAGGGTT
TCCCAGGGTTTAACCTCAACCAAACCCTAAACCCTAAATTTCCCAATTTCTTCCATCTATATTTCTCTCTCTCTCTCTGTGTCTC
ATAATTGCTGACCTCTCTCGTTTGTGCTCTTAGATCCAATCCCCACCCTCTTATCACTGGTAAGATTTTCCGCTGTTTTCCTTTT
TCTTTTTAATTCTCATTTATGGTGAATATTTATTTCCGATTCCTCCTCCCTTTTTATTGGAGGAGTTAGGGTTTTTGCTTAGCTAT
TAAATATTGTTAGTGGGAAACGCCGAAATTGTGATCTTGGCATTCAACTCAAGTTCTGTGTAATCTGAGACTCGTTTTTTTCA
ATTGCGATTCTTTTCAGAAGTGATTGGCGTTGTTCGGGGTGTGATTCTTGGGGGGATTTGGGATGCTGAATTATAGTGATTTG
GGGAATGTGTATTGTTGGGTTTTCAAGTGATATGAAGTGCTGAATATGAGCAATGGCGGTGGGAAGACTGGAAGACAGCTTG
AGCAGCCTGGCCCATGGAGGAGGAGGAAAGTGGGTGATGGGGATGACTTTGCCAGGGCAATTGCGAAGATTGCGGTAGCG
CAGGTGTGCGAAAGCGAGGGGTTTCAGGCTTTTCAGCAGTCGGCTCTTGAGGCATTGTCTGACGTTGTGGCTCGGTACATTT
TTAACGTTGGGAAATCGGCACATTGCCATGCTAATCTTGCAGGAAGAACTGAATGCCATGCTTTTGATGTCATTCAAGGGTTG
GAAGATATGGGATCAGTCCAGGGATTTGCAGGTGCTTCTGAGGTGGATCATTGCCTTGAAAGTTCAGGTGTTATTAGGGAAA
TTTTTCATTTTGTTAACGAGGGTGAACCGGTTGTGTTTGCGCATCCCATTCCTCGGTTTCCAGTTGTGAAGGAACGGGTGCTT
AATCCAAGCTTTTTGCAAAAAGGAGAAGAACCTCCCGGCGATCATATTCCTGCTTGGTTGCCTGCCTTCCCTGATCCGCAAA
ATTATTCACAGCCACCAGTGGTGAATGGAAGGGGCACAGAACCTCGTGCAGTAAAATTTGAGCAAGAAAGAGAGAACGGC
AAGGGGGAGTGGCCTGTGTTGAATTTGAAGCAGCAGATGGTCTCAAATATGTTTGAAAAGTCTGCCTTGGACCCTGCAGATA
CTAAGGCAAAACGAATAGCAGCAGAAGGTAACCCATTCCTTGCTGCTCCTTTGAAAATTGAGGACAAGGAAATTGCATCTGT
TCCCCCTGCAGCCAAGCTTTTCAATGATGTAGTTCTGGATTATCCTGTGGTTGAAAATTTCGTTGAAAATGAACCAATTTCAG
CTTTGGAGACATTTGCTCCTGCAATTGAAGCAATGAAAAGCACATGCTGTGATTCTAAGGAAGGCCAGACAAAAAGTTTTGT
GAATGAGAAGCCTATTGTGCGTTTTAAGATTGGGATCAAAAACAAATTATTAGGAAGGTCCATTGGTTTGATCCCACAAACA
GAGGAGCATAACAAGACTTTGCCATGGTTTGCGATGGAAGATGAGAAGGATGACAGGAAAAGGAGGGCAGAGAAAATTCT
AAGGGAATCCTTGGAAAACCCAGATCAGCTTGTTCAGTTGTAAACTAGATTTTTCTTTAACTAGAATCTGTATACTTGATCAG
GAGAATGTCAGGATAGCTAGTTCTGGGATATTCAAATCTTGGTAACTGTCTGGTTAGGATGGATATTACTATTTTGCTCTGACA
TGTTCTCACTTTAACAATCTTTCAGGTATGTGCATGTCAATTGTTTGGTAGATCAATTTATGATTCTTTTTATTGAAGTCCTTTC
GATCTGTATGATCTTATGCTAAATATAGCATACGAAAACATCTCAACTTCTGACTTGTGGCAGTTATTCTAAATTGTGTACTCTA
CCCTTTTACTTCGTAGTCAGTGCTACATCCACAGCTAAATTCCCACAAAAAATACAATCACATTCCTTTATGGTTAACTACAAA
CTGGCGGTGGCACCCTGCTGTTTCAAATCACGTGTTATAGCTAATAGTTCAGTGGACAATGCATAAGATTCGTTTAATAGTTAC
TCATATTTATCCTCTGCACATTGTCATTGTAGCATGTTCATGTATTAACAAGTAGTATCTCAGCTTCATGCCTAAAATGTACAGG
GAGAACTATTTTTCTAGTATATAAGTGTTGTATTTTCCCTTTTTTCTTGTTAGCATTTTCTGTGTATCATCTTGAAAAGCTACAG
TTTTTCTTGTACAAAGAGATTGACTTTTGCACTACCAATTTATCTGCATTTTACATATTTTAATCCATTTTTCCTTGTTTTAAA

>VrTAF8-2 LOC106764373

CATAAACAAATACTTGTTTTTGCGTTTAACCTTCTCAGTTGCCGCGGAGGAGTATGGCGAACGCCAAAGGAGGGCACCGAA
CTGTTTCTACTTCGTTTGAATCCACGGTTTGTTTTTCTTTTCTTTTTGCTAATTTGTTTTTCTATTATATTTTCATCTCTTCCCCCA
TTCATGCACATAGATTGGGTGTTTCTTGCAGTATTACCGCATATACTGAATACCATGATCTTCACAATTTCAAATTAATTTTCGA
TGTTACATTGTTGTCATATCTATCTCTTGCTAATTGATATTTGCTTCTTACAGCGTTTCCTCGTGTTTTGTTGAACTCTGTGTTTT
GTTGGGTTAATTGTTATTTTTCTTTTTTGGTTTATGAATTATCCTAATTGATTGATGGCTGCTGAGTGATAAAAGGGAATGATGG
GGGCAGTGTGAAGGAGAATAGAAAAATTTGTGTTTTTGGTGGGGTTGTCTGTTAATGAACTGGAATGCCCTTTTTGTTAGCG

TTTATTGATCACTGAAAAAAATTCTAGCTTTGATTTCGTTGTTCTCTTTGATCAATGTAGTTTCTATCAGTTCCCAGAACCATG
GGGGTTATTCCATGGCTGGTCAAGTAACCATTGAAGTGCCAATCATTTTTGGAATGAACAAATTACGATTAACGAGCGCACAT
GGATACTGTTGTGTGAAAAAAAGCTGAATGGCTCTCTCTTTTGTATTATCGATTCAATTTTGCTTATTATATTTTTTTCGTCTGA
TTGCAGGAAAGGAGCTAGTGTGCTTGTCTTTGATATTAGGGACAAATTGTAGCTGTTTTTCAAAGGGTTCTTCTGAGTTCTAT
ATACCCTCTTATAGTTAACTCCTTTTCCATGAACCCTATGCTAAAGGACAGCAACACAAAAGCCACATCAAAACTACCCCGA
AGGGGAAAAAGAAAAAAGAAGGGTCTGGGAGTAACGGATCCTCAAGTAGCAGAAAACCCATCAAAATTCTCGTTTGCTATA
GCCAAAATTGCAGTTGCCCAAATCTGTCAATCAGCTGGATTCAAAAAGTCCGAAAACAATGCTCTTGAAACCCTAACTGCTG
TTTCCACTAGATATCTGGAAGCAATTGTGAGATCAGCTGCCTCATTTGCTAATGCCTCCAATCGTACTGACTGCAATCTCTTTG
ACCTCGTCAATGGCATTCATGATCTTTGTTCTGTTCAAGGATTTCCGGGTGGTTCAGCATTGCACAAAGATGATCTGCTGAGG
TCTTCAGCTCTAAGAGAGATTATGAATTTTGTCAACCTCTCTGATAAAGTTCCTTTTGCTAAACCAATTCAATGTAGAAATGAT
TCCGATGTAACCATTGATTCTGGGACATTAATGTGCTTCTCTAACCAAACCAAAACTCACATACCAGGATGGCTCCCACATTT
TCCTGAGCAAAACTGTGATCAGGTTTTAGTTAAGGAAAGGAAATGCGGCGAGAAATATTGGGAGGATTCATTTACTGTGGAC
GAAAACAGTGTCATATCACAGAGCAATCACATGAATGGAAAAGAAGGGAAAGACACAAGGAGGGAATTGCCAGAGCGAAG
AGAAAGAATGAAGTTTAAAATTAGAGGGGAGGAGGAGAAGCAAGATGGATTGGGTGTGAATATGATGAGTGGGGTTTGTAA
AGGAAGGAAACGAGTGTCTTGGAATCATTACAAAATGAATGGTTGTATTATTGAGAACAACAAAGATGAGAAAAGATAGCA
TGATTGTTATGGAAATGAAATGATGTTTGTAACTCTTGTGTAAGCTCAGGATATGAAAAAAAGAGTGATATGAAAACAAATGA
AAATTTCGTGCAGCCTAGTTTTAAAGAAAGTGCTCTGTTCGGAAATTCTTTTTGAATGTATGGTAACTATGCCTGGCCAATTA
CTGCATACTAGATTTTTTTTTCAACCCTTTTGGACAATATATTTTGAAGCTATGAAATTATGATCTGTTAACTGTTAGAGTG

>VrTAF8-like LOC106769930

GTGGGCAAAAGCTCCTAGGAGTTTCTGTGGCTTTGATTGACCAAAAAACTAGCTCACTTCTCTATATATAAACCTTCCCCAGC
TCTCTTTTTTACCATTCATGTTCTTTCATTTCATTTGATCCCTCCATTCTTTGAATTCTCATTCCTTACACAATTCTCACTCCAAC
TTCTCAGTCTTCGACCCAATTATCTTTTCACTCCATTATCAGACTAACCTTCAGTTTCATTTCTCCAACACTCTCAATCACCAC
CACCACCACCCTCACCTTTCTCTTAGCAAGGTTTATAAATCACAATAGGTTTTTGTTCATTTTCTTTAACTCTTTCATCTCTTTA
TACTGCTTTCCCAAACGAGGCATTTTTCAATTGCAGCGTGTCAAAGATTCAAACCCCATGGAACGCGAACAATGCCGCGCGT
GGAAACCCTAATTGCGCCCCCGACCAACCATGACCAACGGCGGAGGAAGAGCCGCGCCGGACGACTACGGCCGAGCCGCA
GTACGTCTCGCGTTGGCGCAGCTCTGCAACGCCGCTGGGTTCCACAGCGCCACCTCCTCTGCGCTGGACGCCTTCGCCGAC
GTCGCAATCCGCTACCTGCTCGACCTAGGCAGAACCGCGGAGTCTCATGCGAACCACGCCGGCCGATCGCAGTGCACCGTC
TTCGACGCGATTCGCGGCTTCGAGGACCTGGGAGCGCCGCGAGCCTTCTCCAGCCCCGGCGGAGTCAGAGATATCGTGAGT
TTCGTGGAATCGGCAGACGAGGTTCCGTTCGCTCAGCCCATTCCGCGGTTTCCGGTCGTTCAGGAACGACGTCGCATTCCGA
GTTTTTATCAGATGGGGGAGACTCCACCGTCGAAGCATATTCCCTCGTGGTTACCGGCTTTGCCAGATCCCCATACGTATATTC
ACACTCCAGTGTGGGATGAGAGGGTTTCTGATCCTCGTGAAGATAAAATCGAACAAGCTAGGCAGCGTAGGAAGGCTGAGA
GGTCGTTGTTGAGTTTGCAGAAACGGTTGTTGTTGCGAAATGGGTCATCGGAAGCTAAAGCAAGAACGTCAGCTTCGCCAG
ATAGTACTGTTTTGGAAACTCAATGCGTTGGTGATGACCATAAGGATGTTGATAAAGATGGCGCTCCGGTTGTTAAGGTTTCA
GTTTTGGATGAGGGTAATGTTGGTGACAGGAACCGTGTTTCGGTGTTGGATGCGTTTGCTCCGGCAATTGAGATGCTTGGGA
GTGGAGGGTTGGGTGGTGATGAAGATGATGGATTGGTGGAGATAGACAGAAGTGAGCTTCCTGTTGTGAGACCTACTGTGC
ATTTTAAGTTTAGGACCGGGAAAAAGTTCATTGGGGAGTCTTTGGATATGAGAATTCGGAATAAGGATGCGTCACGGACGGT
GGGATTGGTTGGGAGAGAAGATGAGAGGGATGATAAGAAAAGGAGGGCTGAGTATATTCTCAAACAGTCTATGGAGAACCC
CCAGGAACTCACTCTGTTGTAGATTGATTAGTGTATTTATGGACTTGGGCGTGGTTGATTTGCGTTAAAGAGGGCTGAGGGTG
GATCCTAAATTAAGGGCTTGCGATGCTTTGGTGAGGACTTTGTGACTTTGTAGAATCATTCTTAACATGTTTTTAGAGCAGTT
GAATTTGTTTCTTTTCTTGATAGTGTAGCTGTAATTTGTTAATGGATTTTTGAACATAAAGCTCTTCTGTCCGCTTCTCATATTG
TACTTTACTCACCAAATTCAATTGAATCAATACACTGAATTTTCTATGCA

>VrTAF9-1 LOC106764413

CAGAATTCAGAAACATATCTAACGTCAGTTTTCACTTTTGAGCAGAGAAGCCTCCGATTCCGGCACGGTGGGCGGCATTGCA
GATTCTCGGTGGTTCATCTCCGCGCCGACGTGAGTTCCTTAATCCTTTCCTTTTTCAGCTGTGAAGATGTTGAGAATTTCATG
GATGTGTATTATTCCATTTTCATCTGTGAATATCTTTATCAATGCAAACCATGAATTTTTAGTACTTGTTCTGTCCTGGTCTTGGT
TATATTATATGCACATATATTATATCTCCCCGTGGATAATATATGAGAAATTTTCATGGTGATTGATTATTCACCTTCAGACCCGG
AATCTGCTTTGGAATTATTACATTTGATGTAATTAGCCAAATTATGCCGTTCCTGTCATGATTAAATCGCATCTTCAAGTTGTCT
AAGTATTTGTTTTGTGTTTGTGAAATAGGTTAACATAATGTAGGAATATAGTTAGATTGGAGGAATTATTAGAGCTTTATTCGAT
CTTAAATTTGAGTCCCGAGTTTTATAAGAAATCTCCATTGAAACATAACGATATAACACGAAAAGGATTTTAAATTCATGACTT
GAGATCTTAAAAGAATTCAGTCTTAACCTAATAATTAAATTAGCCTTTAAGTTGTAACGAATTGAATTGTAAATAAAAAATTAT
AAAAAGTAGATGTTATAGGATCTTTAGATTGGACCAAAACTTGTAAGAAAGTCTTAAAATGATGTAACAAGGTGGTAGAATA
AGAACTTGTAAAATCATCTTCATCAGGAGATGTTTACACAGGTGTTTTAATAAAGTACTAGTTGTCTGAAGCAACTTTTCATT
GGAGGAATATGAGATGATACGCAAGTTGCATAAAAATAGTTTGTAGCTGATATGTCAAGAAATCGTTGCTTGTCAATAAGAAA
ATAATTTTTTGGGCCAAGGGAGAGGGAGTGCAAATAAAGAGAGTATAAAATAAGGAGATGGAGAGAGTAAACATATAAAAT
AATGAGTTAGAGTTTCTTAATGTTTTTTTTTCTATTAGAGCAAATTTGAGTATGAGTTGAAGTTTCTTAAAAAAAAAACTCAA
TTTGAGAAAAAATTGATATGAGTTGCTTAAAAATAATTTGAACCACATTTTACATTTTTCAATATGGGGAGTTGCCCTAAGAA
GGTCTAATGAGATCCTGTCAGTTTTAATCAATATTGTACAAAACATATTCAAGTTTTTCATTTTACTCGAAAGAATTAACCGTA
GTTAAATCTCCTAAGCCACTTAATAATGCATCATTGGATGATGATACACAAATATCTTATTTAACACCCATCCAACACCAATAA
AATATTACTTTTATTTTAAAATATAAGTTGATTTAGGATAAATTAACTAGAAAGTAAACATATTTTTTTATTAGGTGTTAAAGGA
GTGTAGGAAATTGAAAGTTCTACATGTAAAATTGTCCGATAGAGCCTATGACATGATAGGTAAACAGGCGAGATAAAGCTTT
GTAATTAGGAACATCAAGTTTAGCCCGATTCACCACTACGGCAATTCTTTTGTCCTTTTCTCTATCTACAAAACCTGTGTCAGA
TTGGATTTTTCAACACCGTTGTAATCTGATAAGATACAATTATCCATTTTGTTTCAAGTAATCAAAAAATGTTTTTTGGTGCAG
TGGACAGGCATGTCAGATAAAGATGAAGAGTTGGCTATGCCAAGGGATGCAAAGATTGTGAAGTCTTTGTTGAAATCAATGG
GCGTGGGGGAATACGAACCTCCTGTTATACACACGTTCCTTGAGTTATGGTATCGCTATATAGTTGATGTGTTAACGGATGCCC
AAGTGTATTCAGAGCACGCAGGCAAGTCCGAAATCGACTGCGATGATGTTAAGCTTGCTATTCAATCCAAGCTTAACTTCAG
TTTCTCGCAACCACCTCCTCGCGAGGTAATATTAGTTATTTTATATTTTTCAATATTCTTACCCAGTGGTTACATATTATATTTTTT
TGTTGTTGAATTTAGGCTGGTTGTGATGTTTCTAATAGAAACTACAGAACTCCTATTCCCAAATGCTTTTATTTTCTGCCTTATT
TGTAGTTAAAAACTAAACAGGTGTTGCAAGTCTCACGCTAATATGAGAGATTTTGGCATGTTTATGTACTTCAATTTAAGTAA
AACCCACGAGGAGAAAGTTGTTCAGTTCTGTTAGTTTCTAATATTAGGAGATATGTTTCATGGAGGAACCTCTTTCAAACTGA
ACATCCTGAATGGAATGATGACACTAACATCTAAGACCACAATTGCAAAAGCTTTGGTCTAGATCAGTGGTTTTGTTAGGAA
AGAAATCACATGTATTACTTCTTGAACGAGTGTTTAATTTAGTGAGTTGGCAGGTGCTTCTGGAGTTGGCTCAAAACCGCAA
CAAGATACCACTGCCAAAGACTATAGCTGGGCCTGGTATCCCGCTTCCACCTGATCAGGACACGTTAACCAGTCCGAACTAC
ATGTTTGGAATTCCAAGCCAAGGGTCTGGTGAACCTGAAGAAACAGAGGATGAAGAAACTAGTATTCCCAACCCCTCTCAG
GAAGAGAAGACAGACATGCAACAGCAGGATCCCCATCAAAGAGTATCATTTCCAACTTCCTTTCATGATTAACAATCTTACTA
TAGCTTTACACGAACTTGTTTTACTACAGTGGTAGTAACAAAAACTAAAATTTTGTAGCTTGTTCAATTTGTACTTTTGACAA
ATTGGTTTCAAAACGGCTCGTTGCGGTCTGAAGTTCTTGTGAACTGAGACTTCACCTCAGCTCTCTATAGTTGTTGGTTTGGA
GCCATGGTAATTCTGATAGGGTGAGAAGATGGAAAGTGGAAGGAGGAAGAAAAAGAAAGGATTTTTATAAAAATGGTGACG

TCACCAATAAATTATTTGAGGGGAGTAGCTGATAGCTGACAACATTCTATTTTTTTTTTTGTATTATTGTTGGTAGAGAAGCTA
AATAAAATAAAATGATGTCTTCTAAAATGATTTAAAAAATAGCAATTAACAGAATGGACGTCAATAGCTGTTATTTATTATGTAT
TTGCTTGTGTTTATTCCTTTGT

>VrTAF9-2 LOC106767081

CCATGGGCCACGGTGTTTTCACTTGAGCCTATCAAAAAATTCCGAGCCATGAGGCCCATGCAAAAGGAAGAGTTTTCGTGCC
CTAGACTTCTCCCAAAGTCTTTCTAGGCGAATAACCAGCATCCGTCCGACACCGTCCACGGAGATTCCGGCCACCGTGCGTT
TGAATTACATTTTGTCTTCCGGTGGAGCAGTAGAGAGATATGGGTGACAAAGACGAAGAGTCAGGAATGCCAAGGGATGCA
AAGATCGTGAAGTCTTTGTTGAAGTCAATGGGCGTGGAGGAGTACGAGCCTCGCGTCATTCACAAGTTCCTTGAACTATGGT
ATCGATACGTCGTCGATGTATTAACAGACGCACAAGTCTATTCAGAGCACGCCGGAAAATCCTCAATCGATTGTGACGATGTC
AAGCTTGCGATTCAATCCAAGATTAACTTCAGCTTCTCGCAACCACCACCCCGTGAGGTAAGAACAGTGATTAATAATTTGC
CTTTCCAATTGTTCAACTCTGTTTCATGTGAATTTATGGCTATAAAATCTTCTGAGAGAAAAAAAAAAACAGACATTCTTTTTGT
TGTCTGAGGTTAAAAGTCAAGTGTGTTAATTTTGTTGGCTTAATTTGGTGTGCTGATAGTTTGAAGCAATTTGCTGAAGTTTG
CAAGAGTTAAGGTAGTGTGAGAGGTTGCCTTGTTTATGTGAAGTTAAATCTGTGTCGCAACTCAAGCAAAGCCCACAACGG
GACTTGAAATAGTTGGGTTCTCTTCACTTCAAAGATTAATTTTCTTTTTATTACTAGGTTGTGTGTCAAATGATGATCACACAC
ATTTATATTCATATTGAGAATAGTGTGTTCTGTGATATCCCTAACATAGGTTAATTTTTAATCCAATCATATAGATAATACATGTG
CTAAAACTAGCGTCTACAATTGCTGCGTCTTCTATATATAACACTTCCCCTTTAAACTGGAGCTCATGGCCACTTGCATCAAGC
TTTTTACGAATGTGAGAAAGGGGATTTTTGTTGTTCGACATATGGTTGGATGCACCAAGAGAGGAGGATTGGTGCATACTTTT
ATGGAAAACAGAGTAATTGGGCATCCATATGAATGTGTCTTGTTGATCACGTGCAAGAGCCTTATGACTCTGATACACTCCCC
AATTTTGACAGATCTTGATTTGTAGCTGCAGGCAGAAACCCTCTGGAGTTAGAAAGCAAATCTGAAATTTTTACATTTTTTGG
ATCATAGACAACCCTATGATCCGCATGCCACACATGCCTCCCAATTTGGTTTCATTGCCAAGAATCATGTATGGTATATCTTGTA
GCCAACTCTCTAACTTGTAAAAGTTTCCTACAAATCTAGCTACCATTATTTGCTGGTTTAATACACCAAAATAAATGCTTCTCT
TTTTGAAAATCATTGTATGTAAGATACAAACCTGGATAGAAAGACTTTTTAGCCTGAACCCAGACATGCTGCTTCATAGCAGC
CAGCTTTATTCCACTCCATTGATGAAGTCTGAGCTCCTTCTTTAAAAGGCATCGAATTTTCTATCAATATTCCCTTTGTACTTGT
ATTAGGGGGACTAGACCCACCTTAAGAACCATCTGCAGATCTTATCCACTCTCTTCAGATCAAAAACCTGCACTGGCAGTAT
GAACACAGTACTCCAGTAAACTTGCATGACCAGTTGTAGAGCGTATTGGATAGAAAGACCTAACACCTAAAATGCAATTGTT
TTTCTCATGTTATGGAGAATATTATTTGGTTTGTTCTAGACTTTGACTGACCAAGTTTGCATGAAACTGTGAATGAATATAGTAT
CCACTGGAGTCTGTCTAAGTTTTTCTAGCGAGTTTATGTTCTTTGCCTTGAATTGTAAACTAGCAAAATTATGTACATTTCTATC
TGACAACCGTGGAACAAGTTCCTGTGCTATGTAGTTTTGATAAATTTATTTTTAAATTCATGTAAATTATTCTTGTCTTCTTTTC
TTTTGTGTGAAAGGCCCCCACCTTTCTGTGTTGCAGTTTCATTACTAGCTTGCAGATATTAATCTTCTACTAATTTTTAATATTC
ATTTGTTTGAGAATTATGGCTGAGATAGCCTTTTCTAAATTATATAGAAACATTTTTCTAAGATTCATTTTATTAGTTACTAATTA
CCATGTCATGAGCTGCAGTCTAGAATTATTTGTTTGCTTAAAAGTCATCTGTCTCTTTGGTGATCCAATACCATCTGCACTTAA
TTAAAAAATTAATTTTAAGTTTAATTCAACTTATAAAACTGACTTGTAAAATAAAATTCATATTCAGTTACATATTATAATTTAGT
GTACTTCTAATCAATATTGGATCTCTAACACTCTTTTCCCTCAAGTATTATGTCATCTTGAAAAATAAAATGTTTAATATAATCA
CTTGCCAGGTGCTGCTGGAGTTGGCTCAGAACCGCAACAAGATACCATTGCCAAAGACTATAGCAGGACCCGGTATCCCACT
TCCGCCTGACCAAGACACATTAATCAGTCCTAACTACCAGTTTGGAATTCGAAACAAAAGGCCTGCTGAACCTTTAGAAGA
AACAGAGGATGAAGAAACTACCATTCCCAATCCCACTCAGGAAGACAAGGTAGAGCTGCAGCAGAATCCCCATCAGAGAG
TTTCGTTTCCCCTGCCCAAACGCCAAAAGGATTGATTTGAATTCCCTTATGTGACCAATGTTTTAGTCTAACTTTTGTGTAATT
GTTGTACAACAGTATTATTAGCTACAAAATCTCTATAAACGATACAATGTTTGTTTTATTGTGTAACATCCACTAGCTTCTGACA
TTATAATGAAATGAAAAAGTTTCCAAACGGCTCATTCTGCTTGGAATATGTAATATGTAATATGTGATTCGTTTCAAACTC

>VrTAF10 LOC106758746

AGGGGGTGCATATAACAAAAAATTGTGAGTTTTTTCGTGTAAGGCTCTGCTATATAAGAAGAGAGAGAAGAAGAGGAGCCG
AAGAAGATAGTGAGTTGAGGTTTGAATTGGGAAGCGCTCCATTCTGTGTTTGTTTGATGCGTCGCTGTTAATTTATTGAACCA
AATGTGTTTGTTGTTATAAATTATGAAGCTTTTTTTGAATAGGGATTGATCGGTAAGAAAGAAGGATGAATCAGAACCCGCAA
TCGAGTGATGGAAGAGGCGACGATGACACTGCTCTCTCCGATTTCCTTGCGTCTTTGATGGATTACACTCCCACTGTAACTTT
CCTTTATCTCTGTTCTTCTAATTTCCCATCCTGCACAATTTAATCATTGTTCCCTTTGTTTAGATACCTGATGAATTGGTGGAGC
ATTACTTGGCCAAGAGCGGTTTTCAGTGTCCTGACGTTCGATTGTGGGTTCCTCCTCTTATCTCCATTTTTCTATTTTCTTCTTT
GAGGAAACAGATTATTGATTACTATCGTTTCTGCTTGGTTTCTCTTGTCTTTTTGTGACAGTTATTGAGACATCTATTTATTTTT
GGCTTAATTATGGTTGAATTTCCTAATAGATTTAGGTATTCTTAAGTTGAGTCTCTATTTAATTATTGTGTCCCATTGTCTTTGTA
GTTTAACCCTAATCATGATATTGTTTTATATTTATATCTCTTAACCCTAGATACAAGTGATCAAGATAACAAGAAAAGATAACAC
TCAATACAGTTAGTTAAATAGAAAAAACATAATGAAAATATTGAGTATTTAATAAACCAAGAAAACTTAATAGGGTCTCAATT
GAAAATAACCATGCATTTGCATAATGTTGACGGCTACAGTGGTTTCATATTATGATAGCTTGTCGATATTTTCTGTAAAAAAAA
TAAAAGGCAGAACCGATCTCTAGAAGGAAATAAAAAAATACCATATTAGGAACCCAAATATGTCATGGATGTCATGGACCCAT
CTCTGCTGTGTCCCTCTGCCCTTGCAGCTGTTATTTTAGAGTTTTTAAGGTATTTAGATACAAGGGGATTAACAAACAAAGAT
ACAAGATTAATAGTTTATATTTCTTTCATGGTTTTTGTGCAAATCACAACTCTTGTATTTATTTATTTTTAACGTGTCTCACTCTT
TCATTGGGATTGTTTAACATGGTGCAGTCATTATATTGGCAATGAGTACCCACTGTTAATCAATTTAAGTGGGAAAATGATGAA
ATATGCTAGCTATTTTATTTTTCTTTTCACTCTTACTTGCTTGATTTTGTATTTCTTGTTGTGCATTGTTGTTTTAGACCCCCAAC
ACAGTGGATAATATGTGCTTATTGTAAACTAAATTGGTTTTCAAAACTTTGGCATTTTGGTTGACTGTTGCTGACTGATTTATT
GGATTCCTGACCCTTGCATTTCTTGTTCATATGCTGTGACCTTGACTTGAAACACTCATCATTTTTTCAGGACTAGATTGGTAG
CTGTTGCCACTCAAAAGTTTGTTGCTGAAGTTGCAGGAGATGCACTTCAGTATGTTTATTTTACTTGTGTTTTTGGTTGAATCT
TTACCTTCTAGCTTTTATTTGATTTATTTTTAACCTTGAATTACGCCATCACAACTAACAATTCCTGTGTTAGTTACCTTTAGAC
ATATTCTTCTATTGAAGTTGATTTCTGGTTTCCTAAAAGTATTGAAATTTTTGCAAATGTTAGTTCATGCTTGCCAGAGGAGCT
TAGGAGTGTGTCTGAATTCTAGTTTTTCTAATGAGTGTGTATGGGTATTATATAGCAAATGCATATACACATGCATTTCAATGGA
ACTATTTGCACTATTGCACTTAAGTTTTTGTGTGATTTTATCCCTGCTTATGTAAATATCCATAAATGTAGAGTCTACAATGAGA
GGACAAATGTGAACCAAAAGCTTAATATTAAGGCTGTTGACAAACTTTTGGCCCCTTGGGATAGCAACTTTTGGTATTATCCA
TTTAGGAAATACTGCACTTTTATTGTAAACAACGTACATTCTTTTTTAAACTCATCATCTTAAGTAAAGTTGAATAGATGATATA
AAATATTCTATATCAACTTAGATCTTTTTTCGTGTTGGTTATATTTTATTTCATTTGATTTCATGTCACTCTCTACTCACGTATGTC
TGCAGAGTCTTACTGTCCTATAGTTTAGATAATAAGTGTTAATGTCTTAATAGAAATATAAGTGGCCAACTTGTTTTTTAATATT
TGCTTTGGTCCAAGTGTTATTGATTTTCTTAGTTTTACCAACGTAGAATCCCACAAATTTCTTCCCTTTTTAATCATCCATGAGT
TTTGTTCTTTGCTAAGTTATAAGTGCATTGTTATTAGTCATTTAGTTGCCTTGTCTTTCTTTTATGTGTCTATACTGGATGATGAG
TTGACTAGATGTTAACTGTAGGCACTGTAAAGCAAGACAAGCAACAATTCCAAAAGACAAAAGGGACAAGCAGCAAAAGG
TTTTGACAGCATATAACTGATTTTCTCTATACGTTCATTATGTGTATGAGCAGTACTCACATTTCATCATGTATAACAGGATAAA
CGTTTAGTTTTGACCATGGAAGACCTATCGAAAGCATTGCGTGAGGTAAGAGTAGTTTGCTATTTCTCTTCATTGTAGGGTCA
TTGCAATGCATTAGTGTATTATGGATGTTTACTCTCACTATGTAATATCTACTCTCCACTCCTGACTATATATATGAATAATTCAC
CCTCTTCCATGCAAGTCTGTGATAGCCCATCACTCATCTTTTAAACCTTTACTTTGCTAGCTTATCTCGATCTTTTTTTGCTCTT
TTGTAAGTTTATAGAAAATGGTGATTTTGGTTAATATTCAATAGTGCGCTGAAGGTCTTAACCCAATTATGACAATTTACACAT
CTTCTGAGAGAATATAATTGGGAACAGAATAGTAGTATCATCTCCACTGCATTGAACACCGTCATTGCCAATGACATATTTCAC
TACTTTCTTATCTTAATTGGTTTAAATCCGTTCAATGGCTTTTAATTCCATGTTTTCTGCCATGTCTGACCAGCTGGCCTAGTTT

CATCATTTTCTTTATTTTTGGGGGTTCTTCACAAAGAGTTTAGGGCGGGCAGTCAGTCTTAGACACATGCCATGTGTAAGCAT
GGTCAGCTGTCGGATCCCGTTCCCCTCTGGGCCAAGAGAAACAAACTGCAAATAATCGCGTCATCCCATTTCGGTGGTGTAT
TAGTGTATAATAAATGTTTTTAAGCTTCTTCTTAGATCAATATTATGTGTTTTGTTGAGATAGAAGAAGATAATATACCGTTCAT
GTTGGCTAATTTTGCTGCTATGTTAATATTCCTTTACTTTGAGTCCACTAATTATCTTTTATGAACAGTATGGCGTGAATCTAAG
GCATCAAGAATATTTTGCCGACAGCCCTTCTACTGGAATGGATCCTGCTACTCGAGAAGAATGAGTATTATCTTGTTTATTTGT
GTGTTCCGGCTTCTAAAGTTTTATGTGTAGTAAGTATTGATTTATATACCATAGCTGAATTGAGTACATTCTATTTATTGATATGT
TGCTAGTTCTGTGTCTCTAGTTTTATGTTCAGTGTACTGAAGTCATAGCGCGGTATGCCCATTTTACTTTCAGTATACTGTTAAT
GTAGTA

>VrTAF11 LOC106773625

TCAGATTCAAAACAAAACCTTAGTGTGTTCGGGATGAAGCAATCGAAGGATCCGTTCGAAGCAGCGTTCGAGGAATCGCCG
CCAGAATCTCCGACGGAGATAGAGGCGGACGCAATCCATAACGATAGCCATAACCATAATCATAACCAAAACCCTATTCCTTC
TCCTTCTGTTCTACCCCTCAACCCAGGGCCTCCGCAGAAGGCCAGTGCGGCGGTCAAGAACAAAGACAAAGACAAGGACG
ATGAAGAGGAGGAAGAAGAAGACAACATGGACGTTGAGCTTTCCAAGCTCCCTTCCACCGGCGACCCTCACAAAATGGCC
AAGATGCAGTTCGTAAATCACTTCACTTCAAATTCGTTACCTTTTTCCACATTTACCAATTTCTCCCCTTTTTCTTCTTCAGGA
CAATTTTATCTCAGTTCAGCGAAGAGCAGATGAGTAGATACGAGTCCTTCCGGAGGGCCGGGTTTCAGAGAGCTAACATGAA
AAGGGTATCTGCTCTTCTCTTCTTATATTACTCCATTTGTGTTTCATTGCTTTGCTTTAATTAACAGCTGCTGTTGTTTTGGGTT
GCTCACTTTCTCAAGAAGCGTGTTTGTATTCACGTCGAAATACCAGTTAATGTGATTTCAGCTATATATGTATTCACATGTTTGA
TGTAACATCAATTAATATTATCTTAGGTGTATATTCACCTGTTTGAGTTAATCCAGTTAATTTGATATTATAGGAAAATGTACACT
TGTTTGACTTTGCTTTGAGGAACTGTGACATTGATGAATTTCGAGTTGAAGCAACTTCACTGGTTTTTGTGTTGGATAAAAAC
ATCATACTGATTTTTACTGTTAACGTACTTTTTGGATCTGAATTTCGTTTCTTTTGATAGAAGATTGAACCAAGAGCAAATTAC
ATTGACAATCCTGAGGTTTCTTGTTTGAAATTGTACATGAGCCCTCTTTCTTTCTTTTTCTTCAACGTTTACAGTAACCTTTCTT
GCAGTGTTGCATGGTGTAATTGTTCCTTAGTGATCTTTCCTACAGAGCTGCACCTTGTAATGTATTTCCTCCTGAGTATTATGTC
GATGATTTGTTTAACGTCTGTTGATCTGTGTAATGTGCTCTATTGGTAAGTGACCGTAATGTATTTTAAACAATTATACACAAG
AAGAGTTGGTGTAGGGTAGGGATTAGAAAGGGAGAGGGTATATTTGATAAAATCTTAGGGCTGTGTAATTTACTCTGGAAATA
AAGAACAAGTATGGTTTTGGATTGGTGGCTTGTTGATGTTAAGGAAATTTTCCACGGGAAATTAATAGGTGCAATGAAAATAT
GATGGGAGAAAAAGAATAAGGGCATCAGATAGGTTATCAATATCATTGCATCAAACATTCATAAAGAGATCAATATACTGCTG
TAAATACTGAAATTGATCATGTATCTGAATTGAAGCTTTATGGTTTAGCCGAGTAGGCTGGTTTATTTCTTGATTGAGAAAAAA
TTAAGTTTAATGTCAACAGTCATAAAGGATTATATTACACCACCACCTTAGACACAAGACATTGTCAAATGTAAAAAGTTAAA
ATTGTCAAATTATTTACTGCTGGACTTTGTCCTGTTTTTAACTGATTGAGGCAACATTTGCATTTTCTCCTTGATTATTGGCTGG
GTGTGTGCATGCTACTCCTTTTGGATTCAACTCATGATTTTGCGTTTGTTTATGGATTATCATTCTCTATATCCAAATTTGCATCA
GTAATTTATATACTATAACAAGATTTGCTGAAATTTTCTCCTCTCCCTCCCCCTATCAGTTATTGGCTAGTATCACGGGGACCCA
GAAGATTTCAGTGCCAATGACAATTGTAGTATCAGGCATTGCAAAAATGTTTGTTGGTGAAGTTGTTGAAACAGGTAAGGCT
AGTTCTTCTTTGTAAGTTTGAGACACTTCTAAAATCATAGTATCTATTTCTACAGATTTTAACGATGTTGTTGTGTTTTATTTAG
TGCAAGCACACATTTTGCATAATTCCAGTTGTTTATGTTAATGAATTGTACTATTTTTTGCAGCTAGAATAGTTATGAAAGAAA
GGAAGGAATCTGGACCAATTCGGCCTTGTCATCTGAGAGAAGCATATAGACGGCTAAAACTTGAAGGAAAAGTCTTTAAGA
GATCAGCCTCAAGGCTCTTCCGGTAGATGGACCAAATATGTGTTTAGATTTTGAGAGTTTTGGAAATATGTTTTGAAAATATG
AAAGATATGATATATGGAGGAAGTTGTATCGAAAGATATTTTTAAAAGTTGGAAGGGTTTTTAAACACTGATTTAAATGAAAA
GATATTACACAAATATTTTCAA

>VrTAF12 LOC106772695

AAGGAAGGAACCAGCTTGGTAGAGAAATTAACAAAGGCAGACCAAGATCATGCGTGAACATTTTATTTTTTTCTAAAACTTA
ATAAGAGGCGAACACGCATTCTGATATAAATTTCAAGCAATATCACAGCACGCTCAGGCAGTGATCCTCTGTTTCAAATTTTC
ATTTGCATTCTCAATTCTCAATGGATTCTCAAGCTCCGGCAACCGGCACCACCCCCCGGAGTGCGGCTGAACCCTCACAATC
TCAACCACCAAAGTCATCTCCTCCTATACTTCCATCTTCCACATCATCAACTCCTCCAGTCTCCGCCCCAACACAGTCTTCCC
CAAACCCTAACCCTAGCTCAAGCCCAAACCCAAGCCCAATTCAAACCCCCAACCCCAAACCCCCCACTCCTACGCCTGCCC
AACCTCGCCCCACGCAATCCTTCAACCGCACTTTGCCGCCGTCGCAGCCTCAGTTCCCGCACTTCTCTTCCGCTCCTTCGCC
TCCCTCCGCTCCTGGCGGCGCCGCTGCGCCGAGGGGTGGTATGGCAATCGGCGTGCCCGCGCACCACCAGAGCCCCTCTCC
TCCGTTTTCATCGTCCTTCGGTCAGCACTTTGGTGGGCTGGGCCGAACTGGAGTCAGTGTGGCTGAACCTACCTCCAATTCA
AGCACCTCTCAAGTGATATTCTGCATCTCTACGTTATTTTGGATATGAATGTGACTACACATTTTCGTGCTTTTTGACTAGTTCA
AATTTTTGCATGTGAATGTGACTACGTTTAGGTGAGAACGCCCGTGCAAGGAATGGGGATGTTAGGGCCTCAAATGAGGCCT
AGTGGAATCGCTGCTCATCAACAGAGGCCCGTGCAATCGTCTCTTAGACCTCCTTCCTCTGCCCCGAATACTCAACCTGGGG
GTTCACAAGTTAGTGTGAATGTTTTTGACTATGCCCTTTCATTTCTGCATTTTTTTTATGCTTGGCCTAGCTACAGGGTTTGTGC
CGTTCAAGGCTTAAATTGCGTTTGTGTTTTTGTGGCATTTCTTGATATTGCGGGGATAAATGTGGCTGATATGACCAAAATGGG
GATCAAGTTATGGTAATTAAGACTTTGAAACCTTGAAGTTGCAGCTGAAACCGCAGTCATGAGCTGTTTCTTAGAACATTTG
GGCGGATTGACTTAAGCTGGGGTTGGATGTGTTTAGAAATAGTGATCTATTTAGGTGCTTTCTATGAATTGGTTTCAAATTTTA
TTGTTTTGCCTTCCGTATGAAACCGTATTAGCAAAATTCATCTAAATACCGACTGAATTTTGTATCAACTCTTGTGTGGGATCC
TTGAATGTCCTTCAGAATTCCAAGTCATCAGGTTTTGGTTCTGTGAGGAGGAAGGGTGTTCTCCATAGTTTAAGGGTGGTGA
AGGAATATGACTTGTTAGTGTGTCAGATTGTAAATATATTGAGCATTTCTTCATTTCTGTTGTTATGTGTATCTTATGAGGTTTTC
TCACTTTCATATCTCATGCTGTATTGCTCGTGCTTTTCTTGCAATATTCATATTTTGAGGGAAAAAGGGTAGGTAGTTTTGTAAT
TCATTGCATTTTGTTCAGAGCTTCCAAGGTCATGGAATTATGAGACCTTCATCTGTGGCATCACCGGCTACTCCATCTCAAGG
TGCTTCACAAAGTGTACAGTCACTCAATCAGCCATGGTTGTCATCTGGGCCACTAGGGAAGCCTCCTTTGCCATCCACTGCTT
ATAGGCAGCAATTGAACCCATCATCAATGCAGCAGAGGTCACATATTCCTCCACAGCAGCAATCCACACCTACATCTTCACA
GCAGCAACAACAACAACAACAACCACCTTTGTCTAATCAATCACAAGAGCATTTTGGGCAACAGGTTCAGCCATCCAGGGC
TTCTCATCACGTACCTCACCAGCAACAGGTTACAAGACTACAGGGGCCAGGAAATCAGAAACCTTCATCTCTTGTGGTGGCG
CAAACCAGTGTAGTTCAGCCAGTGAGTCAAAGTAGACTGACAAATGCAGATACCGAGGAACCCTGTAATAGTATTCTCAGC
AAAAGAAGTATCCATGAGCTGGTCAATCAGGTGAAGTATGCTACTTTCTTGTGATCCATAGAAGATTTTCTGGGATTATATTCT
AGTTTCTACCACCTGTTGTTCAATAAATTAGGAGTGGAACTCTCTGGATTTGTTAATCCATGTAGGAATGTTGAAGGTGTCTG
TTTGTATGAATTTCCTTAAGAAAGAGAAATTTATTAATATGTAGTGGACTTGCATTAATAATTTTGATCAACTCCCATTCTATGT
TGCCTGATTTAATCCGAAAGAATTTCATGGGTTCTCATAATTTAAGAAATTTTATAATTCCCCAGGTCCCATATTAGATGTTTAA
GGAATTGGATGGTGGCATACAAAAACTTAAAGCTTTTAAGATTGGATGGTAGCTTTTATATTTTGTGGTATGTTATAGAGGTTT
GTATTAGCACTTGGAACTTAGAATCCAGTTAATAATAATGTTTTAATGTCTAAATGGCGCTATGCAAGGTAACAGCCTCTTTAG
CCAGCAATTTGGATTTGCAGGTTGTATATTATTCTTAGTATCTATTATAATGATAGAAATAAATAATTGATCCTATATAATCAAGA
ATATCTAGATTATGTATGTATTAGTAGAAACATTTTTATTTATTCCTGCATCATATGTTTGTTATTAGAGGATATCAAATCTCTTCT
ATTTATTTTATTGATTCCTTTTTCTCAATATAAATAAATCACTCACACTCTCTCAGAAACACATAATTTCAGCTTAATGTTTCTCT
TTTTCTTAGAGTTTAGCATGGTATCTAGAGTCTTTTTCAAAATCATATTGGCCTGGTAGACATGTTGTCTCAGGAGGATATATAT
TTTTGCAAACCTTTCTCCCAGGTCTGTGTTCTCTACGTCACCTCTTGTGAGTTTTTAGGTGGTGTTTCTAGCGACCCCTTTTTT
CTTTGACCAACCTCTGCTCAGGTTGCCTCTTGTGCACTCCGACCACTATGATGTCCAGTCCATCAAGGTTTGTTTGGTGGTTT
GATCTACTTTTCCCTCTCATTAGGTTATTTGTTTTAACATCTTTCTAGAAAATAATAATGTCTTCCAACTCCACATCAATCTCTA

GAACATATTCTCTCCTATTTTCCGACCATATTGATAGTATTGAGAAGATACACGAGTTTAACGATTCATTTGGGCTTTTGAGATC
AGGTTACGAGTAAGTGGGTTTGGCCCAAATGACGAAACATTTTTGGTGGATTGTCACTTCATAAGAGAAAAACTGGAATTTT
GATACATCACAACTAGATTTGTCAACTCTAATGAGCAGTTGACTAATGAGCAGTTGACTGACATATCACCAATTCTAAGAGGA
CCATGAATGATCTATTATGTTTGCAACAAGCCAAGGACATAAAACTTATATACTCCAGCTTTAGAGGGAGTGTTATGATTATAG
AATTAAGGAACAGATCCTATATAATTAAGAATATCTAAATTATAGAGTGTTTTTATCTAATCCTATCCTATTATTGTTATCAGAGG
TATCAAATCTTCTTTATTTATCATATTCATCCCTTTTCTCAACATATAAAACATCCATGCTGTCTCAAAAATATGATGTTTCGGTT
CAATGTTTATCTCTTTCCTAGAACTTAACTGTAAAAAACTGTTTCTTATATCTATTATCTTTATATACTACTTGTTTTGACCCGTT
CAAAATAAAGGTCATGTCAATTACCTTTTTGGGTTTTAAAGTTTAAAAATAACTTGTGTGATGTTGTGCTACTAAAGTTTACAT
GGAAATTTAGTGTAATTGATTGTTTTTATCACTGTCAGTTTGGTCATTATAGTCATAGGATCACAGGATTCTTCTAGTTTCACTA
GCCTCCTGTGCTGCTCTGCACTTAGGATTTCAACTTTAACAATGTAATGAAATCACTGGTCTTATTCGCTATCTCATAGAATCT
GCAACGTAATCGTTCCCAATCTGGACACATTTGTTGTAGCTAGGCTCATGGGTCTGGTTTTGAGATCCATGTCGCAAAAACCC
ACAAATTCTCACCTATATTAGGCTAGTTTGTTTAAACCTTAATTAAATGTTTTTTAAATAAGTGAGTTTTAAAGTAATCACTTAT
TTACAAAGCAGATAGGATTTGCCTATTAAAAGAAGCTGTGGCCCTTTAATCTGAATCTCTTCCTCCTTCTCATTCAGATACACT
CTCTCATTCCCTTGTGGTTTGAACTTTGTAGAGAGTTGAAAGCTTCTGTCTGAATTCTGCTCTATGGATCATCTCCTGCCCTTT
CTCACCGTCTCTGTTTCTGCCATCTAGATTCTGATCTGATATTTCTGGTCTGATTTGAGTTTGGTATGTCAGAGTGTCCCTTTTT
GGTTTGGTTGGTGGCACTTTTCCTATGGCTGACTGAAGAAACTCCACTTTTTTTCTTTTCATATTTTAGAGTCATTTCTTGCTT
CTGAAAGGCTGAGTTTCCCAACAAAATTTTAATTTTCTGATGTACTTTTGAGCACCGAGGACCCAAACTAAAAACCAAACAT
ATCCTGAATTTAGGAACTTATATGATCAATTCTTTTGAAGTCACTGGCCTAACCTAGTGTGTGATTGGAGTCTGCTTATTTTGA
GAAAGTTAGTTCTTGTTGGAATCTGTTTTAGCTGTACTATTTCTGTCAGAATCTTTGTTTTTATTGGTTATAATTTTTCTTTATTG
AACAAATTATAATTATTGTGAATAATGGGGAAGTCCTTGAATCATAGGGATCTGGTTGTCTAGGAGCTATTATTATGCTTCCTAA
AATAGCTTTCTAGCCTTGTATATATGGATTATATTCTCAGCATAATAAAACTATAAGATTCTACCCTAAATTATGAGAGTTCTTTT
TGCAACTTCCTTAGATTAAGAGGGCTTTCAAAGAAAAAAAATCAATTATTTTCCTGATATTCAGCTGAAAAAATGGCAGTAAT
ATAGGTGTGTGTTATTTAGTGATTCCCAATTGGTTTTAGCTTATGGTTATCATAAATGTTTACCCAGAATTACTCAATTGCTTAC
AGTTGCCATTGCCCTCTGTTTCTCAATGGATGATCCCTTAAGCACTTTGTTCTACAAAATCGTTGTTCCACTTTCATTTTTAGA
ACATGAATTTACAATCTGGAATTGGTTATTAGATGGAATATACCAGGAGGCATTTCAAAGTTCTTAACTTTAATGATTCATCAA
GCTTTAAACGGCCCTATTTTTCCCAAAAATATGTAGTTTTCTTTATATTTACATTTATTCTTTTTTGGCAGGTGGATCCATTGGA
GAAGTTGGATCATGAAGTTGCAGATATTCTTGTGGATATCGCAGAAAATTTTCTAGAGTCTGTGAGTATATTTAGATTTTAGTC
TGTGATGGAATTGTTCACTTTTGCAACTTCATAAATTTATGTTGTATCTATATCCTTATTAATGGTAGGGCTCACTTTTTTTCTTT
TCAGCTAAGTACATTATTTTGACCCATATAAATCAACGGTCAGATGTTTTAATTCTTCAGAACTACAAGGATTAACCTAGTGTT
AGAGAATTCCTTTCTTCTGTGTGTTCTGTTTGTATATAAAACTAAGGCTTTTATGGATGTATTTTTTGAAAATAAATAACAATAC
AGAAAAGACATGTTCTTAGAGGATGCTTTCTTGTGAACATTGATTATGATTCTTTTGTTTTACTCTCATTTTTTGTTGGTACTTT
CCCAACGTAATTTGCTAGGAGTTGTTTTATGAATTCCTTTTTGTTGATAATGATTAATTTCTTTAGAAATGAATTATGTTCTCAA
TTGCTCTCGGTCTTCTTTTAGATAATTTTCTTATCCTAGTTTTTAACTCTGTAGGTTGTTTCAATAATGTTCATTCTTTTATGGTT
TCTTTAAATAGATAATCAGGTCTGGTTGTTCATTGGCCAAGCATAGGAAATCAACAACTTTGGAGGCGAAGGACATACTTCTC
CATCTTGGTTAGTTACTAAAACTTCTGTTCTCAGATTTTATTGTCTGCAGATTTCACGTCTAATGCTATAATGCTATTCACATTTA
TACAGAGAAAAATTGGAACATGACACTTCCTGGATTTGGCGGTGATGAGATTAAAAGCTACAGAAAACAGGTAAGAAGGAT
CTGAATCTTTGTCGTATCTTTATGTTTTATTCATTGATTGGTTTGTACTCTTTTGAATTTCATATTATTGGCTTGTAATTTGTGATG
ATTTTGCAGATTACAAGTGATATTCACAAGGAGCGTCTATCAGCTGTAAGTTTTGGTCTCTTTTATTCCTCTTCATATTTTTGTG
GTTGTGTTGTATCTATTGGTTGTTCCTTGTGATTGCACAATCCTGCAAGGATGAAGACGTGTTGGTAGACTTTTTCAACACGT
TATTATTCTGATATGTTCTTTATTTTCTTGACTTATTTTTGTTACTATTGCTAATGTGATGGTGGTGATTATTTTGATTTCATTTGG
CAAAATTTGACAGATAAAGAAATCAGTGGCAGCAACTGAGGCGGCACATGCTAAGGGCGCTGGCCAGGCTTCTGGTAGTGC
AAAGGGTAATCAGGCAAAGACACCTATGAATATCATTGGCTCCCCAAACCTAAAAAGTTCATAGGATGCCCGCATTGTTTATG
CTTTACACAGTAATGGAGGAAGCACTTTGGTGAAGGTAATTGAACTTTGTTATAAATCAAACAGGTATTCCTGTTTGAAATCT
TGCATCGTGAACAAAAATAGCACACACGTCATTTCACAGTTCAGAAATTGAATTCAACTTTAAAGTAGTTTCACACTTAGCA
CCAAATTGTTGCATGCAGTGATTCGTTTAGTTTTTTGACCCTGATTAACTTTTAACCAGGCAAGATGATAGAAATTCACAATTT
CTATTAACTTTTAACTAGGCAATTCTGTTAGCAATTTTACATTCTGAACAAAAAATGGGATCTATATCTGTCATTCACTGTTAAG
AAATTAATGCCTAGAAATGCTTTAAGCTTTGAAGTAGTATCACACTTAGCATCTAATTGTTGCATGGAGTCATGCGTTTTTTTT
TTTGACCCTGATTCCTGTGGAAGACGATAGGAATTTCACAACTTCCATTAACTTTGTCTTTGTAGAAGGAAATCAAAAGCATG
TTTAATCTTGATCCTGATAAACATTTAAAATTGCTTGTCATGGTCTCATGATGTTTTAACACCTGATTGGTGGTATTGTTAGCAT
ATGTTAAACTCAGATGTCAATTCTGCAACTGACCTGCCAAAGCCCACCTTTTTTAAGTCAAACTTGTTCGATTGAACCTAATA
TTAAGAGTCTGTTCGACCGCTTGTATTTGAATTCATCATCCGGAGTTCTAAACACGCACTCAATTCTTTCCTGGTTAAGCTTCT
TGTTATTTTCCTTCTTTGTCTGCTTGACGTTATTCTCTCTTTGCACAAGAATGATTTTCCATCTGTTGTGCCTGTCTATTGTTGTT
AGGAAGATCAGGGTTCTTTTCAGGCTCTTCCTGAGCTTTCCATGTGCACCTAGCTTAACCTGAGCAATGCAGAATAGCAGAT
AAAGGGAGTCAAGATTCATATAAATCATCTTACATCTGAAGCTCGAATCATTGCAGAATACCAGATAAAGGGAGCAGAATTG
GCTTCAGCGACTACAATTCTTTTGTACTGATAACAGTAAGAAAGCCATTGGTTGGGTCCGCTGGAATGTTTTTTTTTTTATTCC
GATGAA

>VrTAF12b LOC106774141

CTAACACGTGACGTAACAATGGAAGTGATCGAAAAGAAACCAAACAGCGCCGCAGAAAGTGAGATACGCGAAGAATGTAA
GGTTGGAGTGAGAAGCGTAACCATGGCGCTTACTTTCAGAACCCTCTTTCTCATGCTACTCGCAATCCTCTCTCTTCTCCTTT
TCGAGGACTGGGTGTCAACGCCTTCTTGCAACAACACCGACAACATCATCGACGACAACCTTAAAGTCATGATGGTCGCTG
ACCTCCTTCTCTCCGATTCCGGTTTCGTCAACCGCTTTTTCCGAGACTACTACATGTCCAAATTCTTTCGGGTACTAACGTTGA
GGACTCAAGGTACCCACTTCTACAACCCCGTTTTCTCAATTTCAATTTTTTTTAATTTATCCACGCGTCACCGATTGCTCAGAA
ATCGTTCGAGGTGCTGCGGCCCGATTTGCTTCTGGTGTTGGGCGACGTTTCGGCGCGAGGCTCGGAGCTAACGAGGAGCAA
GTGGGTGTCGGTGCTGCGGAGGTTTTACAGAGTGTTGGGCCCTTTTGTTGGGCTTCCGTTCCACGCTGTTCTCGGCGACAGA
GACGTCGGAGAGTGTGGGGACGTTGATGTTGATAGGGTTAGTTGGATTGCGAGCAAGTTTCCTGGGTTGGATTCTTCTGGCT
GCGCTGCGTTTGAGATTGGGAATGTGAGTTTTGTCACGCTGAACGCGGTGGCTCTGCTTTGCGGGGGGAGTGGTGGGTTGC
GTTTTGATGTGGAGAAGGTGATAGAAAGGGAGAGTGTGGAGGTTCACATGGGAATGGAGAGGGTGGTGAAGAGAGTCAAT
GGTTTTGGGGAGTTTGCAGATGCGGATGTTTTATCTGGGTCAGGGCCTGTCGTTTTGCTTCACCTGCCGTTGGATCAAACAA
GGAATGAACACTTTGGTTCGGTTGGTGATTTTGAGAAATATTGGACTTCTTCCATGGAGGGGTTAAATGTGGTGCCAGAAAG
CAGGTTAGTGGTTGGTAGCTTTAGCCTTTCACATTCATCCTTGGTTTTGAATACGATCATATTATCATTAATATGTAATTTAGTTG
CTCTACATATGACTAATGTTGAAGATCCCCCATTGATATGACTAAATGATAGTATATAAGTTAAGCAGGAGCAAACCTCAAAGG
ATGAACTAGGCTTAAGTTCACTTTCTTAGATAGTATTATAGTCTATACTGGTGAAGTTTGTTTGACATATTATGCAGTTGCAAAT
AGGCCCTTCATTGTTTCTACGTTTAAGATGTTCAATCCATGTTGCATCTTGATGATAACATAAAGAAACTAAATTTTGTTTTAAT
AACACCACAGACAAACTCATTTGAACCAAGAGATGAGTGTGAAAGATGAGACTAAATTGCATTTCAATGATTCATGCAATGA
ATCATATGAAATAGAAGATGAATGTGAAATATGAAAGCTGCTTTCAGGACTTTATTTTAATTTATTTAAGCTTAGGGACTAACT
TGGAATCACACCACACTTTCATGACCAAATAGACCATTCTGCCGAAGGATGTACATTTCTTATAGTCTTTCTGAAAGTGTGTG
CCTGAGTATAATTGTAGTTCCCTGAATAATTTTTTTTGGTATATTTATCTGTGTTTATAGTTTTATGCTGTTTAGTTTGTGTAGGC
AATTGTCAGTGTTCCTTCTTGCATTATTCATGTTGTTAAACTTTCTGTTCCTAATTATTTCTTTTTGAATTAGGGGTGGCCAGTA
CAAGTTGCTTCATATGCTGCCTCCGAATGCTTCTGAGTACATTTTACAAGCTCTGAAACCAAGGTAATCAAGTGCATATTTGA

TTGTGTGTTCATATGATAAGTACTTGATTGGATTCTCTCTTGCATGTTACGTGTGTTTCTGGAAATATGTTATGGTTTCAGTCAT
AAGTTATACTTCTTCCAGCCTTGAGGTAAAAAAATGTTGGTCCTATGATGGTTCTTTGACCTGCCTCTCTACCCGTTGGCTGC
AGGATTATTTTCAGTGCTCACAGGTATACATTTTCTGATCACGTTCATGGGGACAGAACTCGTGAGATTAGTGTTCCAGCAAT
GTCATGGAACGCAAGAGATGACCCTGGATTCGTGATTGCCTCTTTCCAAAAGGCAGGAAGAGCAGTGAGCATAAGCCATTG
TTCTCTTGCTAGGGAATCTCAAATTGTTCTAGTCTACATTTCTGTAATGTTTTTGTTCTGTTTGGTGTGTTTAAAAGGATAGCA
GGCAAAATTACCTCAGAAGAAAAAAAAAAACGAGTTTGGAAAGCTGAATTCCGCCAATTTTGGAATCCAAGATTAGTTCTGT
TGTGATAAACCTGATTCGACCAAGAACATAGCTCATTGTACTCATGTTGTACTACAAATTAGGGTTCTGATTCCTTAACATGTC
TGTTCTCACATGTTTTTCTTCATTTTGTGACACATGTATTCCAGTAATTTATATCAAATTAAATTAATTTTATTTTGCATTCAGAT
CAATTAACAACATTCTTTAGTTTCTTGGCCGATGTTTGTTTGTGTTTTTCTTTTTAATCAATGCAAATCCTCTGGAAAAGCTAA
ACAACGACATCATTGTACTAAATACAATATATATATATATATATATATATATATATATATATATATATATATATTAAACCAAATATTTATCT
AAAAGTAAATAAATAAATTATTTTTTATTTAAGGATAAAATAATTTAATCTAACGATATATCTTCATAATAGATTTTTAAAATTAC
GATATATCTTCATAATAGATTTTTAAAATTACATATAAAATATAGGGTCGGGAATGTTAATAAGTACAATAAATAGATTATATATTA
CGATAATAAATTAAAATAGATATTGGGTTTAATACCTATTTTGGTCCCTCTTTTTAGGAGGGTTTGTTCAAAGTGGTCCTCTCTT
TTTTAAAAAGTTTACTTAAGTCCTAGCTTTTGCAAAAACTGCACAAATAAGTCCTTTTTGCTAACGACGTTAAAAAATTAACG
ACAGAGTTGCTTGCATGGCCAAAATTAAATGACATGTCCAATTAGTCCAGTTAGGTGGAAAACTCAGCCAATAAGAGACTGC
CACGTGGCAATCCCCTTTCCACTCAAGAAAAACCTAGAGGGTGAAGACCAGAGTTCACAAGATTGTTGTTCTTCAGTTTGTT
TATGGTATTTGAATCAAGTGTCAGTGTCAATTGAGAAGAACAATCAAACTTTTGGTGAGCGTTTTCGGAGTTATGGGGATTGG
TTTTATCTGATTCTTTCTTCTCCGCTATAAAATCCATAATCGTACGTTCTTTCCCAAACCCTGATCCCCTTTTTGATTCATTGCAA
TTGCTTACAGTGAAAAAAGTGGGCTCATAGCAGATGATGATGATGACGCCAAACTTGAACCTGGAACCTGAAGAAGATTTC
AAAATTTGCAGCCAAGTTATTGCTGCAAGAAAAACCACAAGATTTTTTGGACATCATAAGATTTTTGGACAACATCTCACCG
GTTCCGATAGGGAACTTTGTAAATTGTGTAACACTTCTCTATAACATTATAAAAAGGTTTGAATTGGCTTTGCACACATTCCTG
GCAAAAGGTGCTCATTGCAAGGTTGAACATCCTTCGCATGGAATTTCTAGTTCTGAGTCTGCTACTGGTATTCATGCTGATTC
TATTAACAGCAAGGAAAAAAGTATAATTGATGGACCTTGCTACCAGTTGGAGGACAACAGTTGGTTTGTTAGGGATCCATCG
AGACATTGTTTTTCATCAAATTCTTGTAATGAGCTCACGTTCAGTGATTGGGGAAGATATGAAACACACCTCCGCCGTAATGG
AAGAGAGCATAGTTACCTTTGCCCANANAATCACGACTTGATTTTACCCCAAATCCTAATTTTATTTTAACCCTAACTTTTACA
TATTCCACGTCACATTTAATTAATTTTTTAAAACACATATCAATACCACATCAGCTCTACCATTAACAAAGTCAACGCAGTTAG
AAAAAAGGACTTATTTAAACACTTTTTGTAAAAGCTGAAACTTAAGTAAACTTTTTAAAAAAAAGAAGAACCACTTTAAACA
AACCCTCCCAAAAGAGGGATCAAAATAGGTATTAAACTTAGATATTGATAACGATAAGTTTGTTTTAGCCAAATAAAAAAATA
TATAATACTCACTTATTTAATTAAATAGCAATTATGATATATCGTGTTAATATTTTGTTAAAACAATGTTAATAAACTTTTATCCCA
GCTGGCATGGAAGTTTTTCATATAGATTAAGGATTAGCCATTAAGAAAGATATTGAAGAATTGTTTCAGTATTCGGTAATGGAT
AAAAGGGTTTTTCGATTTTGAAGAAAGGAAGAGATTGTTTTGATTGTGTTGTTCGAAAGCAAGGTTCTTGACGCAGATCGAT
TCTCCGGCCTTCCGCCTTCCTTCAATTCAACTCAGCTCTCTGCGCCTTCCCCTGCGCGCCCATGGCCGCATCATAGCACCGAT
TGATCAATCGATCACATCAAAATCAATGGCGGAAAGCGCATCACCGTCGTCCAAACCCTCACCAATGGACCCTCAGAACCCC
GCACCATCCTCCAACCCTACAATCCCTTCCCCTTCCCACAACAACATGCCTTCACCTTCGCTCCCTCCGCTTCCCCAAGACCA
GCAACAGCAACAGCAGCAGCAGCAGCAAAATCTTCATCAACAGTTGAGTCCGCCTCAACAACAACAGCCTCTCGTGTCGTC
GCAAGCCATGAACACCATCAACGGCATCAACCCTATCTCCAATTTCCAACTCCAGCAGACCTTGCAGCGATCGCCTTCCATG
TCGCGCCTCAATCAAATCCAGCCTCAGCAGCAGGCGCAGCAGCAGCAGCAGTTCGGCGTCATGCGCCAACAGGCCGGATTG
TACGGCGGACAGATGAGCTTTGCTGCCGCGGGCGGCGGAGCGGGCCAGCAGCAGCAGTTGGGCGGCTCCAATTTGTCGCG
GTCCGCGCTGATAGGGCAGAGCGGACACTTCCCCATGTTGTCTGGTGCTGGCTCGCAGTTTAACTTGCTCTCCTCGGTATGAT
TCAACTTCGTCTTCCTCTGCTAATTGTTTTTGCATTTCGTCTTTCGTGTTTGATACTGAATGGAGTAAAATTGGTAAATCGTACT
GTAGTTATTTGGGTATAGATTTCTTTTCCTAAAAACTCCTAGTAAGCCTGTTTAATGAAAAAAAAAAATTGAAAAGTTTCCATA
AGCCTGTTTACTGTAGCTTTTTCCCCCCGTGAAATTTTATGGGAATGTTGATCCAAAGAGGGCCATAGTCATGTCACTGATATA
ATTTCGCAAATTGTGCATAAGGATGAGTTGTTACTTTATATAAGTTGTATCTGTTTATTATTTCATGAACTGGAGAGGATGGGAA
TCCATCGGAGCAACTGAGTTGCCTTACGGTTTACCTTTTCAACCATGCTGAAGTCTAGTTCCATGGTTTGTTATTAGGTATTTG
TGATAGGTTTTGTATAAATTCTTTCTCGATAGTGTGGCTGTGTTTTAATAACAACAAGTTTTCCTGTCTGGTAATACCGAGCAG
TGCTATTGCTGGATGACTTCACAAACCATGTTGGTTATAAATGTGATTTGCGGTTGGTGTTTTTGTAATGGATGGTTATTACAA
TCTTTATTTGGATTACCTGATTCTATACAATTAGGTTATCCATGAATTTGTATGTCTTCTGATGAATCACATAATCTTTGAATCTT
GTGATACATGGATGAATGAGACAATCACTGCAATTTTATACACTCATACCATTCACACAATCCGTGGTTCTTTATTTTTACTTTT
CTTTTACCATTTTCCACTGGAAGCACTGAGGAGTAGGGTATATTGGGTGATTTTTTAGGCTTGTGAAAAGGGGTAAAGGGAA
ACCAAGTTTAACATTAGAGGAAATTGTTAAAAGAGATTTCATGTGAATAGTATCCTTGAGAATTTGGTCTGTATGTGAGCCCA
ATTATATTAAACAAGTCATGTAGCCAACTTCATCTACTGTGATACGATTTTTGTCTTACTTTTCTTTTACCATTTTTCATAGAAG
AAAATGAAAATCCAATAAGACAACCTTCAAAATAAAAAGGTTTTTAGGTACATTACGACACTTACCCATGTTATTTGTTCCAA
TGCTCTATTTTCATTCCTCTGAAGTAGTAGCAGTTTCAAACTCTCACATTGTCTCTATTGTATTCAGTTCTTCTATTTAGTTGTT
CCCTTATGCTTTTATGAGACTCTTAATAATTTGTTTGATATTGATACTGGTGATTTTGTTTTGTGACTCATAATTTTTTATAAAATT
ATTACGTTTAGAGCTATTTTGCTTTGAACTATAAATCATGAAGAATTAGCTTAACAATTCATGTTATGAATTTGTTTGACAACCT
TTAATTTGGCTACTGCATTGATATTGAAGTGTCATGCCTTCTGTGTCAAAAAAGCTATTTTTCTTAAATAATTTTCAATTTGGTA
TAGCCAAGGCAAAAAGGTGGGCTAGTTCAGCAGTCTCAGTTCTCAGGTAATTCTGCTGGACAGTCTCTGCAAGGAATGCAA
GCGATGGGTATGATTGGAACGCCAAATCTCCCCTCTCAACTGCGAGCTAATGGAGCCCTTGCTTATGCACAGCAGCTGCGAA
TGGGTCACGGTCAAATCAGGCAGCAGATGTCTCAGCAAAGTTCACTTAACACTGGACAGGTATATTCTTGCTTTATTTTTATA
GATACAATTTTAGTGAACTAGGAAATAATAATGCTTACCAATGTTCGTGTTTCGTCATATGACCAGTTTGCCAGTAATACACTC
TTATATGGTTTATATTTTAAATGTACGTGTTGTCAAGGTTTGCTGTGTTTTTGATATTGTAAGATGTACATGTTTTTAAAGTTCAT
GCTTTCGGACTTCCAAATATCTTCTCACTTTTAACATCAATGATATACCCCAATTTGATTTTTTAGTTATGGACTGTTCTTGCAC
GCATTGTGTTTTGGTTTTATGCAGACCATTGTTTTAAGAGGTGGTATCTGAGTAGAATGTTTATCCTAGTGTCACTACCTCGGT
TGTTCATTTAATCTCATAAGTTGGGGTTGGCTGGAGCTCCAGAGGCTAGAAATTCTTATTAGTTGTCATAGACTGTTAATTTGG
TCTTCTATGGCAGGATTTGCAATTTGTTTTCATTGTCACTGGAACACATGCCATAAAAGTTTCTGATACTTGCTATTTTATTAAA
ATGACCCATAAGACTGAAATTATATTCCACCAATTGTTCTGCCACCTTCCATTTTATCTATCTTGTTCAGTTGTTTGAACTTTCT
TTTACAATAACCACTTTTATTTGAAGAAAAATTACTTATAATTTTTTCTAGTATTTTGTCTAAGCCTTATAAGAAAATATAAAAA
AAAAATATTTTTTTTTATTGTGTTTAGAATCTGTTTTAATTAGCTTCTTGAAAAAACTGTCATTTTACACAACTTTCAGAATAAT
AAATACTTTTTATAATCATAACTGAACAAGAAATATATTATTTTATAAAATTTCTAAAAAAGTTTAAACTAAAATGTTTTTGGCT
CTACTAATCCATACTTTCTTTTATTAATTGAATTTGCTTTTAATTCTCATCATACTCTATTCTAATATCTTCAAAATGTTTTGTGCT
CACTCTCTGATTCATTTCAAATAGTTATCCTGTCTTATTCTTTGAATTGCTTCAAACTAGCCCTAACCCCATTGGCGGTCTCGTT
TGTCCCACATAGTAGCGTCCATTGTTTATACCCACTCAATGGGTGGATTATTACCATACATGATTAGGAATGTTGACGAAGTTG
ATTTGGAATTTTGAAATAGTTCACTGAATCTCATGGAACGAGCTAGGGGTCCGTTGTTAAAATTGATATTTTACTCATAAGTTG
CACAATTCAATTATAGTAGAATCACAATATTATAAAAGTGTATGATTATTGTTTAAAAGCATGTGATTCCCTGGCAATCTTTGGG
GGTGGGGGGAACAAACCCTAAACATTTTCTTTCTGTGCAACCTTATCTCCTCACCCATTTGTGACACCTTTACTGTTTTGAAC
TGCCATCCCCAATCTGTGTCACCCTGGACCACAGTCCCATTTTTTTGAGACTTTGTTTCAAACTTGGTTTTGTGAACTAAATTA
TTGCAAACTGTGATTGTGCCGCCACTGCCACCACCATCCTCACCACCCAGGTTTCTCTGATGTTTTTGCTTATAAGGTTTATCT
GATGTATTTTTGCCTTTTGTGAATGGGACTTTTTTTGTTCATAAATACAAGATTTTGAATGGGCCAAATTTTTGGTTACAAATG
GGCTTACTGTATAATATATAATTATAAATTTTCAGAGAATGGCTTTTGAGATGATTATAATATATAATTATTTTCTGTTGGAATGG
ACTTATGTGTTCATCTTCGTGCTCAAGATGTCAAGAATTACTTCAATTGACTTTTGTAAATATGTATAGAAACTTTTCAAAATG

GAATTGAGATGAATTAAAGCATTGATAATGCTGTTGACTGCAGGTTCAAGGTTTACCAAGGTCATCATCCCTTGCTTTTATGA
ACTCTCAGTTGTCTGGGTTGTCGCAGAATGGGCAACCAGGGATGGTTCATAACTCCTTAACACAGCAACAGTGGCTTAAGCA
AATGCCAGCAATGTCTGGCCCTGCCTCACCATTGCGTCTTCAACAGCAGCAGAGGCAGTCGCTGGCTTCTTCTACTCAATTG
CAACAAAACTCTATGAGCCTGAACCAGCAACAATTGTCCCAGCTGATTCAGCAACAGAAGTCGATGGGGCAGTCTCAGCTG
CATCAGCAGCAACAGCAGCAGCAGCCTCAACAACAGCTGCAACAACAGCTCTTACATCAGCAGTCACAACAACAGTCTCAT
CCACAGGCTTCTGTCCATCAACAGCAACAGTCTCCAAGGATGCCAGGAACTGCAGGCCAAAAGTCATTCAGTCTAACAGGA
TCACAGCCAGACGCTACTGCATCTGGTGCAACTACACCAGGTGGTAGTTCTAGCCAAGGAACAGAAGCAACAAACCAAGTT
CTTGGGAAGAGAAAGATACAGGATTTAGTTGCACAGGTTCTCCTTGAACTTTTGTCCTTTTCATAATTTTTTTTATTGGTTATG
TTTTGAATCATAACTTTATTGTACAGGTGGATCCACAAGGTACACTGGACCCCGAAGTTATAGATCTTCTTTTAGAGCTTGCTG
ATGACTTCATTGACTCTGTAAGGATGCTAAGATGTTTTATCTTTTTTTTTGTTTTTCAACTTCATGATGAATGCGTATATAGTGC
ATGCGAAACAGTTCATGGTTACCCAGCAGCAGGCCCTGACTTTAGATATCCAGGGCGAACACCATGTGAAATAAACTTGCCT
GCCACACAGGAATTTATTATTGGTGAAGTTGTTGAGTGGATTGATTTTATTGTTAGAACAAACTAGATACACTCTCAAGAACA
GAGAAGAATAGGGAAAGAGAACAAGGGATATGGACACTGAATCTGAAATTTTATATTTCAAACACATGTATCACAAACAATA
CATCATTACTATTTATAGAAATATTATAAGCAGTTTGTTGACAGCCACTACCTAACTAACATACAACACTAATTGAATTATCTAC
AAAATATATTATAATTGGACTTATAAAGCATGCATGTTGATTACAATTTGAAAGATTAATAATGTCTTAACTCTGGATTCTTGATA
TTATTAATGTTGTGTGTTAAATGTATCCCCACATCACTTTACAACATTAACTTTTAGGTTGAGTGATTTTTTGACACAATAATCT
ATTGACAATTTTCCAGCTTTATTGTTAATTTGCTAAAATTTTACTGCTGTGCATAGTGTAGTACTCAGCACTCGTGCATGTCATG
GTCATGCAGGATTGTCTCCCGAGGAAATAAATATTTTGAAACTGGATTGTATTCTATTTCTTTGCTTTGATGTGAAAATGTACT
GTTGATAATTTTGTGTTTGGCCAAGATTGTTATGTTTTTCAGAAATGTACTTATCATTTTGTTGCACGCAGACAACTACACACG
GTTGCATTTTGGCAAAACATAGAAAATCATCAACTTTGGAGTCCAAGGATTTATTGCTACACCTAGGTATGTTGTTTGTGTAC
CTTATTTTCAGCTCACCTGTTCAAAAGAAGAAGAAACTGTTGGGTGTTTGAAAGTGATTTAGTTTAAAATTTTGTTAAACAAA
TTTGACCACTGGAGTACTATTGGCTACTTTATTAGCTTCATGCAGCTTTTGTCTAAGTGATTAAATATGCTCTCTTAATATGAAT
ATTGTTTACTCATGCAAAGAGATAGGAGAGTTAGTTACTGTATGTTGAGAACCCACCTTAATTGCATTTACCCCTAACCTGCTT
TTGTTATGGCCTCTTTAGGGGCTACTTTAATAATTGCCACAAAGGATAGTAACTTCTCATATCGACTATGAAATAGAACTTGTA
AAGCATGTGCAAGGGTTGACTTAGGCCTTGAGTCTAAATTCTACTGCTGTCTCTTTCTTGTGCATTTTTTTTTCTGATATATGT
ATTTGAACTTTGAATCCTATTCTTTGGGTTACTGACGGACTAGATAAGTTGTATGTTGTGTTGACTTTTACCAAGCCCTATATTA
GAATTTTACTTTCAATATAGTCCTGTGGACTTATTTGAGGCTTCTATGATCTATAAAAGACCGTGGATATTCCTGATATCTTGAA
TGTGGTTTACATTGAATGCTTGGATTGATAGCACATTATGATCATGGTTTGCAAGAATTGGCATCAAAGCTTTGCTGATTTATG
TATTTGCCGTGTTATGCAGAGAAAAATTGGGATTTAACAATTCCTGGATATTCAAGTGAAGAGAAGAAGTATCAAAGCAAAC
CTGTATGCAATAAAGTGTTTTTCTCTCTTCCTCCTTTTCCCCTTTCCCAAAAAATAAGAAATCAATCTCATCATCTTACATTCTAT
TTTTCAAACTTGCAGCAATTAAATGACCTTCACAAGAGGCGCCTAGATATGGTACGTTTGGAGGATTCTTGCATATGGTTGAT
CTGTATTTCTTCCGTTGGACAATATATAATTGATGTTTTTTTTCTAACTTTTCTTTTTCAATGATCAGATTCGCACAATGATGGAA
TCTTCGGCCTCTGAGTCAAATATTAATAGTTCTAAAGAGTTGAGTAGACAGGGCATTTCAAATCCTACCCCTATGGGAGCTCA
CCATCTAGTAAGACCCATGAGTTCAGAGCAGTTGGTTTCTCAGGCAGCTGGTTCTCAAATGCTACAGCAGATGACAAGGTTT
TAATGAACTTTTGATTTTGGACTTCGTGCTGCTAGGTTTGACTGCCTGTGCCTGGTAAGTTCGTATTCTTTTTTTGTTTGTTTG
TTTAACACGTTCCTTTTGTTTTGAAGTCATGGTCTGTTTATTTTGCTTACCACCCGGACAAGTCAATCTGGTTCCAAAATATAT
AATCATCACGATCAGAAATCTTTTCTTGATTTTCAGTAGCACTCACACCTTTATTGCAAGAGTCATAAGTTTAAGACCCAGGA
TTTTATAAGACTTTCCCAGTGGAGTATGCAAGCACGACCTAAAATTTAAGACCCTGAATTTTAATTGAGACTCTTGGTCACGA
GAATAAGACGATATATATTTATTTTTTAATCTTGGAAATATATCTTTATGCTAACAATCAAATTAAACATTACTTCGAGTGTAGAT
CTTACAAGACCTTTACACCGGAGAAAAAGTTGCAAAAATGTCTTTTAAAGTGATGTGGCATAGAGGAACCCAACAAAAGTA
TATTAAGATATTGTAAAATGACTCATCCAACCAAGAATGACCTTATATTTGGACCTCGCTAAAATACCCTGCCCTTTTTATTAGC
TCTATCAGGGCCATGTCTCATAATTGCCAACCCAATAAAGGGTATACTTTTGAGGTGAAAAGGCAAGCTTAATCGGCCGTGCC
TAATTTTACTAAAAGGAAATCACTACATAGTATGAAAACTTCTCAGTGGAAACTGTATACAAGGATATGGATTTCTGTCAAAC
TCCTATGTTTTCTTAAACTCTGTTTAGCTACCGATATTCGACAATGTAGTAATGCTGTAGATAATCAAAATTGTGAATGTCCTTA
TTTTTCTTTAATTTGTCTCTTTGCTACTTTAAACTACCAATATTTGAGCAGATAGTAAGTTTTCTAATTGTGTTATGCATTATTTT
GGTGTCAGGCAGGTCTGGGAAGAGATTGCTTGCTGAAACTGATTAGTATACAAGGGAACTGTAATTACTTAGTATTTAGACAT
GAAAAATAGTTGTTAAAGGATCCCAGTAACTCTTGATATTGCCCCAACCCTCAATAGTCCTTTTGTTTTACTGTTTGATCTTAA
TTGTGGTTTTCTAATGTCCA

>VrTAF13 LOC106770840

CTGAAACTGGGCAGAAGCCATTTTGGGCAGCAGATTAATGAAACGCTGCCTTCACAGCTGTGTGTTTACTGAAGGAGTATAC
CCGTGAAGAAGGCTTCATTTTCTCCAAATTAGAAGCAATTGGAGCTAGGGCTCGCGAAGAAGATTTCCCCAATTCTCTCTAC
CTTCGAGCTAAAGTTCGTCAGCGAGGAGGAGGCCCAAATGAAGCCAGCCCAAGAAGCTAATTGGACCAGAAAGATGATGA
TCAGAAGCTGTTGACGGTTGGAGAGCGATTCAGAGTCAGTTAGGAGTTGCTCGAGAAGCTTCGAGTTAGTTGTAGTTCTTCC
CCTTTCCCTCTTTTCTTTTTCTTTTCTGTTTTACGATTACCCTATATAAAGGGTTGTAATAGCATTTTGTGAGGGTGACAGACGG
TGTACTTAGATTAGGTTTATCATTTTCAGTTAGGTTTATTAAGCTTTTCTCAGAGATAGAACTCCACTCTTTTCACTGGAACTT
GGAACTCGGAGACAATTACATTGGGTGTCAGACTTTGCGATTTTCTTTGATTTCTGGTATCAATTGTACTTTCCTTTTTCAGAT
TTTAATGAAATTCTGCATTTAGTTTGATTGCATTACCAATTTACAATTACTGCTTTGATTTACGATTCTTGCAATTTAGTTTGGTT
TCATTTGAGGTCTTGTTCCTGTTATGATTTTAGCGTTTATCTTGTTGCCATTTACATTCCGATATTAAGTCCTGCTGGTTCATTCT
TTTATCCATTTAGATTACTTTGATTAAATTCATTTCTGTTCTTGTATTTTGAATTGTGTTGGTTTCCTTTACTGTAGATTAGCAAT
AGTTGATTGGTTTAATTCTGTTTTTTTGTTATGATATGAACATTGAATGTATATTACCGTTTTGTTCGTATTTCAATTTTTTTCTCT
GCCATTGCAATTTCCCAGTACCTTTACTCTGTTAATTCTGCATTTTAGTTTGATTACATTGTCAAGTTACAGTTATTGCGTTTAG
TTAGGTCTTGTTAATATAAATTACATTAGTTACAGTAATATACGACACATATCATGCATTGTTTGCTTCATCTTTTTCTATTAGTG
TTACTTAGTTATTAATTCTTTTAGTTTCATTTTTTTAGATTTCAGTTTTACAATTTTTCTCTCCACCTAGTTTTTAGTAAATAACTA
ATTAGGTTTAATTCTACAATTTCTAAACTTGATAAGTATCAAGTCCACGGATTGATCCTTGGTTGACTCCTATATTACATTAGTG
GGGAAATTTAAGGTTTGTTGAGATTTTAGGCGACAAAAAAAATGCTTTCAACAAATGGTGTCTGTGATGGCCATGCAGTGCT
ATTTGGGAATGACATTGATAAAAATCGGAGGGCTTGCTGAAACGCATGGTTCAAGGGAGGGGTTTTGTGCATGGCAGTGGCT
TAGGATTTCCTGCACAGCATGGGCTGGCTAGCGAGCTTTTTAGAACTGTTTTAAGCTGTTTTTTGTGTGTTTTAAGCTTGCTG
AATGGTTGAAAGTCCTCAACTAAATTCTTGCTGTTATAGTTGTAAATTCTGCATCAAACCTTTAATGTTTTACACTGTTTTGGG
AGGTTAAACCTACATTGATATGGCTCCTTACTGCATCAGGTTGTAATGCCATACTTTAAACTAATTCCTGCTGAATTTGCTGCA
TTGTTGTAAGTCACTGGTCTGATTAACTGATGCTGCTTCTTGTATGAATGAAAGAATGATAACTTCTATTTAACACTGCAATTC
ACGGTTGTTGGCTGATTGATATGGTAAGAGAAGGCATGTAAAGCAAACTATACGAAATGGTAGTTTGTTGGCTGTTTGTAGA
ACCTAGTTGAATGGGTAAAGTGTAATTTTGTTTGGGCGTGAAAATCATCTGTGAATTGCTGTTTGATCATGTTTTGTTGTGAA
GTTATGTTGTGACATGTCAAAGATTGGATTAGGTGTGTTTGAACTGCTTATGGTATCGTTGAGACCAATAATTGTCTTGGTATG
GTTGTTATTAAGGCTGGTGGTTACTGTTTGATCAATATGTTATATTGATGCTTTGATGTTTTTTTTTTTCCTGTTAGCTCATCCAT
ATTTGTTTATGTGTGACAATGATTGTATAGTGTTTGTGTTATACGGGAACAAATGCAGATATTTCAGGAGCCGTGTAGACATTG
AGAGATGGGGAAAACTAATGTTTGGCTTTTGTAAGAGTTGTATTTTATTCTGTAAAACATATTTATGTATTTGCAAACAATTGA
ACTTGAATATTTGGATTGTAAATATTAATTGTATTCAATTTATTTGAGAAATTACTCTTGATTGCGAAAATTTTAATTTTTGGCAC
GGTTTAGTGACACCCAGAAGATTAGGATGTTACACGGTGGGGTAGGTTGGGTGGGTTAGCCCGGGACAAGTTGGAAGTCCA
TTTGAGGGGCGTGCAGTTGCTAAGACCAATGTCTCTCATTTTTTCCTTAATTTGATTCCTGTCATTATTCGTGTTTTTTATTCTC

TGGTATTCTGTTTGCAGGACAGAACCTACAAACTGCATTGAAAGATCCAAAAGTATCTTCAGTCTCTCCTATTCATTAACTCC
AGATACCACCTGAAGGTAACAGTATAAGCTTTGACCTATAGCAATGAGCAGCTCTGCTGCTGGAACCTCATCAAAACCAAGA
GCAGCTTCCTCACAACCATCAGAAACTTCATCCAAGCGCAAAAGAGGAGTTTTCCAAAAAGAATGTGAGAATTTTGATCTA
AGTTTTACATTTTAGTCCAGTTTCCTCTCATTCGTAATAATATTCTTTTTTTTGGATACATTAGTGCAGCACATGATGTATGGCTT
TGGAGATGATCCTAATGTGAGTGTTAAGCATTAACCCTTACCCTTCGGCTCATGTGCCTATCTACACATAATTTGCATTGCATT
GTCTTTGTTATGTAACGTTAACTATATTTTTTTTCTTGTAATTTAAAGAATTATGCCTATAATTTTTTTTGCTGTTTCAATTAGCC
GCTTCCTGAAAGTGTGGCTCTTATGGAGGACATTGTTGTGGAATATGTCACAGAACTGGTAATACTCATAACTTTTAAATTGT
TCAATTCCGTCTGAGTGAGCTAGTAAGCATGGTTTTCCTTATGCTTAATTTGTCTGCCAAGGTTCATAAAGCCCAAGATATCGG
ATCTCAAAGAGGGAAGCTATCAGTTGAGGATTTTCTCTATTTGATTCGCAAGGTACTTTGTTAAAATATGGCAAAAAATTATTA
TGAAGATTTAAAATTACTGGGAACCTTGAATTCTAGAGGCATATATTTATGTTGGTCTCTGTACATCCTTTTATTAGTTGTTAAA
ATCTTATGTATTCAAAGAGAAACACAAAACTCAACAAATGAATTAAACAGGAATCCTGCTCAAACTAACGGAAAATTTCTCT
ATAACACTTGAATGTATTAGAATAACCTACGTAATTGATCCGTCTTTTTATAAGTGTTTATTTCTTAATTTATTGAGAATCAGAAT
TTCACAATACAATAGCTATCTAGCAATCATACTAGCCAGAAATTGCTCTTTCCAATCTACCTTTATCAGCATGTGAACAATTAA
CATTACCACTATTAGCATTTTATTAACCTGTAGAGCTAAGGAGTCTGACATTTTCGTGTCTATATACAACCAACTGCCAACATT
TTTAGACTAGTGATCAACTTATAAATACGAGTTTGCAGAGAGCCACTGATGAATGTAATTATAGACTTGGTTTTGTGGCGATG
GGACCAATTAATAATTGGCATTAGACCTACATGTGTCATTTTAAGCTTAGTTAACAGCGTCTGAAGGATCATTAAGTATTACCA
ACAAATCTAAATGTCAGTTAGTACCGTAGAACATGCACTGGCAGAAACGCTTCCTGCATGAACGTTACCTCACTTTTCATTAA
GTTGAGCAAAAGCAGCGCGATATTATAAAAAACTGGTGAAGACTACCTACCTCTTAATCATTTGGTAACAAGTGAAGGTAAA
AAATGACCTCAATCCTTGGGTGGTTGGTATTGACTACTCATTACCCAGCAACCTTCTGGATGACCTCATTCCGTAGATAGTAG
GCAAGAAATTTGAAATTGTCTTATACGGCCAAGAACGCAGTTCAAACAAAGAAAACCCTAAAAGTTTAGGCTGTCATAAGTT
AATATGATCTACATTATAAACACCAGAGATAACACTACTATCATATTTATTGTTTGTCTAATATCCATAACTGTATACTGCAGGAT
TTGCCGAAACTTAACCGCTGTACGGAACTACTGTCTATGAATGAAGAACTGAAACAAGCAAGAAAGGTTTTTGAGTCAGAT
GAAGAGAAACTGAGGAAGGTTTTTGAGGTGGACGAGGCCGTGGAAGGATGAGTAACGGAGATTACTGAAAAATGCACTAA
TGTTTAGTTTTTCTGATGATGTGGATTTTAAATTAATGCCTTCTAATCCATGTCGAATTGAAATATTATTTTATCTTCTTGTTCAA
ATTTAATGTGACAAGATTGGTTTAATTATTCGAATGGTGTCTATGTTGAGTTGA

>VrTAF14b-1 LOC106777003

GATGTTGTCACGCAGAGAGAGCCACCATTCCAGATTATGTTTTTTAAGAGTAACGCAAAAAACTGAACAACTGCCGAGGATC
AAAGCAAGCAAGGCATAGTCTAGTCGTATGAATCGGGCAGTACGAGTGCCAACAATTTCTGTAAAACCCTAAACTTCATTCT
TCTGGCTTGTATCTTTCTCAATTTTGGAAAACCAAAACAATACACTGAATTTGGCAAATGTCTCACTCTCAACCGTTACCCCT
CAAGAGACAAGGTGAGCATTTGTCCGATGATGGTGCTTCTGCCATTAAACCGTCTCGCCTCAAAATCGCAATACCATCTGAG
GATTCCGACAAGAAGGTAATCAGCTTATATCATCAATTTCTTCAACCCTAATTCTGTCTCGTTGTTCCTCTAGGTCTGCATGAT
TCTTTCCTTCTTGGTTTCTTTTTCCTACGTTTTTGGTTTTTGGGTACCGAGTTTTTTCCCTAATGATTTTATTTTGGTTCAGAATG
CGAACAATAGGATCAAAGATGTTGAAATTTGTGTCCCGATAGTGTATGGGACCATTGCATTCTATCTTGGCAGGAAGGCCAGT
GAGTAAGTTGGGTCTCTGTTTGTGTCTTTTTTCTTTTTCTTTCTTTCTTGGGTCACTGGATTTGGCTACAGTCTCTAATCCTTCG
TTTCTACCAGGTCTCAGTCACACAAGTGGACAGTCTATGTACGCGGAGCTTCGAATGAAGATCTTGGGGTGGTGATTAAGCG
GGTTGTGTTTCAATTACATCCTAGTTTTAATAACCCCACTAGAGTTGTGGAGTCGCCACCGTTTGAGTTGTCAGAAAGTGGTT
GGGGTGAATTTGAAATAGCCATCACTCTCTATTTCCACAGTGATGTCTGTGAAAAACAGTTGGACTTGTGAGTTTCGATTGCT
TCTTTTGGGGTCTTCGTATTTTTCTGATTGAATAAACATCCATTTCGTTCTCGAACTATTTGAGTACATTTTAAGAATTAATTTG
AAGGTCGATTTAATATGTTAGATGCTATTTAGCATGATTTCTGATACTTTTAGGGCTACTTGAGTAGTTTGTTTTACTTAGATATT
TCATAGGAGAAAGAGCAATACGTCACACATGAAATAGGTGGCTTATCCTCTACTTGTTACCTGGACGATCACATTATTTTACTA
ACTTATGTTCATGAATTTCGATATTGTAGGTATCACCATTTGAAATTATATCCAGAAGATGAATCTGGCCCCCAGTCTACAAAA
AAACCTGTAGTTGTTGAATCTTATAATGAGATTGTTTTTCCTGAACCCTCTGAGGGTTTTCTTGCACGTATACAGAATCATCCT
GCTGTTAATGTGCCTAGGCTTCCTGCTGGTTTGAATTTACCAAGTCCTGGTACTATGCTTTAAATGTTATTTCCATTCCTACCAT
TATTTCGAATTTTGCCCCTTAAGACTTATACCCTTAAGCTTTCCCTCATTTTGGTTTATGCAGTACCAATTGATACTATGAGTGA
CAAGGAGAGAGGTGACACCAAAGATCATTCTCTGAACCAGTGGTTCTTGAACTTCTCTGAGGCGGATGAGCTCTTAAAACT
TGCAGCAGCACGCCAGCAGGTTATTGTTCATTATTCAATTATTCTATCATGCCTCTTGAACTTGGTAGTTTTATTCTAGGGATTT
ATTAGTGGCTTTTATTGTGCATTAGTTTCTTGTGGATGCTATGCTATGTTATGCCGTTTCTTCCTGTTCCTTAAAGTAATATTATT
TTATGCGCTATGAGGTGGTTACCATATGAAAGTAAATATATTAAATTCACTGATACTGATGTGCAACTAATCAAAATAATCTTTT
AACAAAGTCTAGGAGGCTTTTATTGGATGGTGAAACTTAATGATTCAGTCCGTGGTTTTCACTAAATGTTTTATTTCTTTGGCA
TTGTTTTTGGATGCAAATGAAGCGAGCACTAACTTAACTACTGCAGTTGCACATGTGGGCTTCTCTGTCTTACAGCTTGAAA
GCATTTTTTTAAATGTTTAGTCTTTTAACGGTTGTTGACTTTGTTCCCATGTCCTAGGTGCAAGCTCACATTGTTAAGCTGCGA
AGACAATTAAGTTTGGTGGAAGGGCTACCTCAGCTGTCAAAACCACCCTCTGGTTATGAATGTACATGATTTTCCATTTTATA
ATTTTTTGGGCTCCCCCTTTGGGGTCAAACTTGGAAGATCAAGGATGAATAATCATGTATATATAGTCTTCGTTGTAGATTGTG
TTGAATATTTTTTATCGGCAGAAAATATTAAAGTTTTAGAATAACGGACATGTTTAATAGCATTTCAGCGTAAAGTA

>VrTAF14b-2 LOC106780520

TTCACAATTTTGGAAGAGGAGCATACGAACCCTAAAACTAGATGCTCAGATTGAGCTTCTACTTTTGAGACGAGGTCGTTGA
TTGATATGACCATCAGCTCATCTTCGAAAAAGCACGGTCAAGATCAGCCAGAATTAAGTGGTCCTACCCCCAAATCCCAGCG
AACCAAAATGGGCAAATCTGATGACAACGATAAGAAGGTAACCATTGTTTCGATTCGAATTCGGATAAAACCAGCAACCTCG
TTTTCCAAATCGCCAAAATATCGATTTCCCACAAATCCTACGCCTTTGGGACTGCGTAAAGATTACACCTTGTTTCCGTCATTG
ATTCAAATTCGAAACTTTGATTTGCAGCTATTATATAGCACACTAAAAGGATCTTAAAGTCAATGTTCAGAACAGCAAATGTG
ATTTTAGAATATCATATCATACAAAGTTAAAACAAATCCTCTGCATGTCTTACTAAATAGGTTCTTTGTATTCATCATTATACAA
GTATAACCATTTCTGGGTTTTGTGTAGAACCTGGGTAAGAAACTCAAAGATGTGGAAATAAGCGTTCCAATAGTGTATGGAA
ACATAGCATTTTGGCTTGGGAAAAAGGCTAGCGAGTAAGTATCCTACATTGCCTCTACCTGATATAATGTTTTGCTTGAAGTC
ACTAAAATGGAATTTAAAGCTGAGAAAGTCGCCATGTTTATGTTATGTAAGGTATCAGTCGCATAAATGGACAGTATATGTTC
GTGGAGCAACCAATGAGGATCTTGGGGCGATAATAAAACGTGTTGTTTTTCAGTTGCATTCCAGTTTCAATAATCCCACCCGT
GTTGTGGAGTCTCCACCTTTTGAACTCTCAGAGGCAGGATGGGGGGAATTTGAAGTTGCCATCACACTTTATTTTCACAGTG
ATGTTTGTGACAAGCCTTTGAACTTGTGAGTTGTTGGTTACTGCTTTGGATTTAGTTGCCTGTAGCCTGCTTCCAGGAAAAAA
AATTATATTATTGAGCCTTTTATTGATGCTCCGATTTCTTAGTTCAGTGTAGAGGTTCAAGTATGCTTGAAATTTTTTATGGTATG
CAAAATGTCTACTGATTTTCTGGCATGTTACAACCGTCTACAACTTGCTTATGCTTCTGAAATTCTGCAAGAGTGCACCTCTAT
TCCCTGCTAAATTTAGTTAGATAGATATAGCAATATGGCTGGAGTGCTTCATTATTACCAATGATAAGCTTGGATGACTATATAA
ATAACATCTATTTTGCTAACTTCAAAGTTTGGTAAGAGTGTGCTGTTATATATGGCCCAACTGCTTGAATATTATCTAACCATAT
ACTAGACCACAATACAAACACGTTTCCTTTGTTATTAGTCATGCAGATTGCAAACCATTTTATGTGAATGTCAAAATAATTTCT
GAACCTTGTATTCTATTGAAGAGTGATATATATACAGATATCATCACTTGAAGTTGTATCCAGAGGATGAAAACAGTTCCATGT
CTGCAAAGAAGCCTGTTGTTGTGGAGTTTTATGATGAGATAGTTTTCCCTGATCCTTCGGAGGCCTTTTTAGCTCGTTTGCAG
AGTCATCCAGCTGTGAACTTACCAAGATTACCTGCTGGGCTTACATTGCCTCCTCCCGGTATGTTGTGTAGCAATTACCCCCT
GTTATCTGCAGCTCTCCTGTTTCCATGCTTGAGTTGAAAGAACTTGTAAATGCAGTACCTGTTGAGGATGCAAGTAAAAGGA
GGAAAGGTGACACTAAAGATCATTCTTTAAGTCAATGGTTCACGAATTTCTCAGAAGCAGATGAGCTCTTACAGCTTGCGGC

AGCTCGACAGCAGGTATGTATGACAATATTTTTATCATATTTAAAGTATGTAATATTTATGGATGTCATATCGATTGTCAATTGAT
GCCTTACTGGTATTTTCATGGATTAAAATGAGCCGTGACAGCAAGCAGACGCTTGCATTTTACAAAGTAGAAAGTTTGCATCA
TACTTGGTGACTTGTATATAGCAAAATACCTTAGCAATCCAAATTATAGATGCACCTGATAGTTCTGTATGGAGAAATGTTATA
GCAGTGCATCATGATTTATGATCTTGATGCAAGGTTCATGTTTGATCAAAAAAGGAATATGTATGAGGATTTTTGTAGATTTCT
TTTCCTTGAAATTTCTGCTTTCCGGAACATGTACTTCTGATTCATCCATCAAGAGACTTAAACTAATGATCTATGCTTGGGAGT
TTATTTTCGTTTGTATGATGTTTACGGCCCTTTTTGTGGATTAGTTGAACTCAAAATAACTCCTTTGAAGTATTAGAATAGAAA
GAAGAAATGTTTTTCCTAATTTACATTGCTGTATGTACTGATATGTTCTTGTATGGGATTCCGGGTTGTAATTAAATAATTTAATT
CAAAGCCAACCGAGGGGAAGGAGGAGGGGAGGGGACAGAAGACATATACGATGGTCTATATGCTGATTAACATTATTATAGT
TTTATTTAATAGCTATCATGTTCCATCACTCCTACTTGTTCATCATTTAACGATTTAATTAACACATCCATTATCATCAACATAGG
TGGGTTCTATATGTTCTCGTCATTTTCTATGTGGGGCATGAATATTTAGAATGAACATCAAAGAGTAATCTTAAAATAATAAACA
TGACAAGACTTATATAATGCTGACATTATTCATATTTTCCTTTTAATTTGAGAGCGCACTGTGTAGATGAACTTTATCTTCATCC
AAATCACTGACCTTTGTCAGTGTGTTGAATTGAAGTCCGTATTACAGTCCCTTTCCTTGTATGCACTTCATAAGCTGTTGATTA
CCTTGTTACTTGTATATCCCCATTTATGGTAGTGAATCACAAGGGGGACCTGAGAAAGGTGTGGTTGAATCTTTTTTTTCTTGT
TGGTTAATTGTGAGCATGTTTTAACTTTTCTTGTAGATTTTGATTGTTCAATCATCTTAAAGTAGTAAGAAAATTGTGACTAAA
CTCATATTGTGCTACCAAATTCAGGTTCAAGCACATATTGCTAAACTCAGACGACAGATAAACTTGATAGATGGGCAGCATCA
GCAGTTTAAATCTTCTTCTGACCAGTAGATGTACAATTAGGATTTTGGTGGCTTTTTTTGTGATGACTGAAGGAGCAGAAGAA
TGGGATGATTGTGTGCTTCTAATCAGCTAGTTGTTTATTCAACTGTATTGTAACTTGTTTCGTTTTTATTTTTTTGTCAACGGTG
TTGTAATTTTAGAGTAGTGTACCTCTGCCAATAAACAGAAGCGGCCAAATGTGTATGATTTAACCATAAAAGATTAGAAAGTG
TAGTATTTCTCATTTATTATAACTTATATTCAATATACCTATAAATTTAGATA

>VrTAF15-1 LOC106777580

AGATATTAAGGATTAGCATTGGAGGTAAAGAAGAGAAACAGTGAGAGTGTTGAAGGATTGAGGAGAAGCGATAAATCGCAG
ATCTAACCAACAACACAACACAGTGCTGTTTCTTTCCTCCGATTCCGGGTACGACCATTTCACTTCTCTAGCCTTGTTCTCTAT
TCGTTCATTTGCCCATAATTTCATTTTCTTCCACCAAAGCCTTTCAATTCTCATTTTTCTTCCACGTTCCCCTTTTTCTATCGATT
GTTTTTGTTTTTCAATTGTTATCATGTGACGAGTTTGCGCACTTTACGAATTTTCAACCTGACGAAACTTCCCTAAGCATGCGT
TTCAACTCAATAATCTCTTATCATGCCTTTTATCCTGTGATTGCCCCCCTTCGATTACCACGCTGTTTTTTTTTTTTTTAAAAAA
AACTTGTTTGAAGTTTTTCATCACATTGCTTAATTTCATCCCTTAAATCCGATGTCTGTTACTAATTTATGTATCTCTTATTTTTT
TTTTTACTTTATTGTTAAATTTCTCGAATAACCTTGGTTAGGTCAGGGTTGTGATAGTCAATTACTGGTCGGAAAACCGCCTTT
ATTCTTATGTGTGCGTAAGGAAAACTAACTGGCTTGCTCCTTTTTCAGAGTTCTGGTGGTTCGGTTCTGGTTGCTTCTGTTTCA
TGAGATTAAGGCCCCATGGCAACCCATCCTGGAAAACAAGCTCTTTCAAATGGGTCTGTCTATGTATGCAACTTGCCCTACG
GGACTGACGAAAATATGTTGGCTGAATATTTTGGCACCATTGGACTAGTGAAGGTATGGCGTCTCTGCATCAAGCACGCCAA
TCATTCAAATTAATGTTATCATGTTTTTAAAACTGCTTCCTTACTTAAAGATAGATGAGAAATATTACAAGGAACATAAATATTT
TTACCTTGGCTAGGATGGAATCTTATATGTTTATGTAGGAAGTGCTCATTTGAATTCGAATTTTGGTTTTTATCCTTGCAAAATG
AGGTTTACACGAGAATAGTTTAAAATTTTCAGTTTCATGATTTATTTTCCCTTTTTTCCACTTCTGTCTCGTTCCTTTTCTTCTC
CTTTTTTTCCGTGTTGCATTATATTTTGAAACTTTTAGTCCTATTTCATTTATAATTCATGCTATGCAGAAAGATAAACGTACCG
GGAGACCAAAAATATGGTTATATCGAGACAAAGAGACTAATGAACCAAAGGGAGATGCTACTGTGACGTATGAAGATCCACA
TGCTGCTATAGCGGCTGTTGAATGGTTTAACAACAAAGATTTTCATGGCAATATAATTGGCGTTTTTATAGCAGAGTCAAAAA
ACAAGGATGACCAAACTTATAATTCAGCAGGAGTAGAACCAGTAGTTGCTGGTACTGTTGTTGGATTAGAGGAAACTACCAA
GGATGTTAATGGTGGTAGTGGAAGAGGTAGAGGACAAAATGATCCTTCAGGCAAAGCATGGCAACAAGATGGCGATTGGTT
GTGTCCGAATACAAGGTCTGTGATATGAAATTGGTGAGCAGCTCTTTCTTGCTCTTGTACTCACTTATTCAGTCATTTTGGATT
ATTTAGTGCAGATAGAATAACATTTCTTTCTTGTCATAGCAGTTGCTCCAATGTGAACTTTGCTTTCCGTGGTGCCTGTAACCG
CTGTGGAACTGCCCGTCCTGCTGGTGCTGCTGGAATTTCAGGGGCTGGTGGCCGTGGCAAAGGACGTGCTGCTGGACAAGA
ACCAGGAGGTGTTGGCCGGCCAGTTGGTGGAGGACTATTTGGTCCCAATGATTGGCCCTGTCCAATGTATGACTGTGTTGCA
TTGACCTTGTGTGCTTTTTCCTTTGTATCTGATTTCCTTTTAAATATTTTGTTTAGATTTGAATTTCATTATTTTAGTGGTAAAAC
ATACACTTTTCTTATAAATTTACAAAATTTGCTGCTTAGCTACATCAATTGGATTACTTTTTTGTTATGTTATTTTTGAAGAACTG
TTAGCAAACAATTTAAAACATCTTCAAATTGATTTTTTATGGTAAGATGTCTGTTTATATGCATGGGGTACGCAAACCTAACCT
ATACGCATCCGTTATGAGGGCCCAAACAGAAGTTTCCAGTTCCTGTTTAATTGACGCAGTTAATAAAAAAATGCTTCTTTTGC
ACTTGGTTGGCTTCAAGAGATTTATTAAACATTTGCCATAAACTAGCTATACCCACATTTATGATCTTATTCCTGAGTGTGTTAG
TTTCTGTCTTAACTTATTCTAGCACATTCCAGCCAAATATATCATCCTGTCTGCTGCTATTCAAGTTGTCTGTCAATTTTATAGA
AGTCCAGCAACCATCATTTGATCGAGTATTACTTTGGTGGCATTTCCAACCTAGAGTCACAAGAGATCAGAGCATTATACTAT
TGCTATGGACAATTTGGTTTTCAAGAGACTGTTGATACTAAAACGATAGATTAGAACTTTGGCATGGGGTTTTATTTGAAAAT
AATCACTGGTAACTTCCTTGACTATTTTGTAACCCTTTCTTGGGTTTAGGTTACAACCCAGCTCTTGAATCTCTCCTTCCTCGG
TCCTTGTGGTGATTGGCTTTGTGTTTTCATAAATTTTAATTCCAGTTCTTACAGTAATGTGTGGCAACATTACATACTTCTGTGA
TTTTTGTGTTCATTACTAGTTGGCTATGGTTTATGGAATTATTTTCTGTTCCGTCACTACTAATTGTTGTGTCACTGAGTTTCTTT
ATTTATTGTGAGTCATATTTTACTTAACACCTGTTTGAGGTATTTATACCCGACTTTCAACTTGCTCGTCACTTGTGTACACTTT
TGTGTGTTTTTTAAAATAAAATTAGTGTAATGAATTGCCTTTGTCATGCTCTTTTTTCTTAGGTGTGGCAATATCAACTGGGCG
AAGCGGACTAAATGTAATATTTGCAATACAAATAAGCCTGGGCATAATGAGGGTGGTGTAAGGTATTTTTTTATGCCTTCTATT
TCTAATAAAGTAAATAGTGGCTTATGGAGAAAATGCTTTTTTTTTTTTTTTGTTTTCCGTTGTGATAGATAGAGATACTTGTTTGC
ATGATTGATTCAGCTTATTCGAGAGAAATCATCATTTTTTTGCCAGTTTTATGCTAGAACTTTTGAAAAATAAACAATTATTTTT
CCAGAAAAAAGGTAAACCAAACATGCATGCGACGTCATAGTGTGGTTGTTATATTGAAGGCAATTTATGATATACATCATTTG
AGCATTGCCCATGTCATTTTGTGGCATTGCACTTTTAATCCAGTCAGATTTAAGAAATTAATTTTTAGAAGTCTAGTATAGCCAT
ATAAAATGTTGGTTGCTAATGCCTGTATACTATTTATTTCTTATCTGTTCAAATCTCATTTAGCAATTTATACGTATGTGGAAAAT
TGAATAATCATTTGATGCTGCCTCTGCATGATTATACACCTATGGTTGTGTCCTAGTTGTATACGAGTGTCCATGGGGCAAGGA
GAAAATTATTGTATCATACTTTTCTCTATATCAAAGTACACAGTCACCAATTTGTTTTTTAAATAGAAATGTAGCACGGCTTTCA
TCATTAAAATATTAGGAGATATTTACTATCTTTTCACAGCATAAAAGCACCTTACAATGAAATGTGAGCATATAGACAGACCTC
AAATACATTTTTGTCTGTGTGTTTTATAGGTCTGTCACTGTAAAATGTTTTGCATGGTGTTGTTTTATGTGAAATATCATGAAGT
ATTTCATTTTGGGAGACTGGGAGAATTTCAATTTATGATGTCTTGGCAACCAAAGTTCGACTTAGTTGTAAAATTAACTGAAG
TATACATGCTTTAAGCATGGATTAACCACTATTTTCCATTTTTGTTTAAATTTTGAAAATGTTGATTTCCTTCATAATTTTGCCCC
TTTGTTTTTTCATATGTTGAATTTAAGTTGATACTTCAATTTTGTGTATCTTTTTACCAATCATAATTTGTATTTTAGTTAAAAAT
GTTACTTCAAATATTCTCCTAAGGTTATATTGTCTGATTAACAGAGGAGGACGAGGTGGAGGTTACAAAGAGCTTGATGAAG
AAGAAATAGAAGAAACCAGACGTCGTAGGAGGGAAGCTGAAGTAAGATTGATTGAGAGTAACATATACTACCTCTGTCTCA
AACGCCACGTTTGTCTTATAATCTGTTTTTTACTGTCAGGATGATGGGGAGTTGTATGATGAATTTGGAAATCTAAAGAAAAA
ATTCCGTGCCAAAACACAGCAAGCTGAAGCTGCGCGTGGGCTTCCTGGTTCAGGACGTGCTGGTTGGGAGGTTGAAGAACT
AGGTATTATTGCATATCTAGAATGCTTGCACTCCATAATTTATATTATTATAGAGCAACCTGAACCTGCACTGATAGTTTGTGAG
CTAGTGAAGCTCCCACCTCCACTCTTTCCCCACATGTTCCATAACACCGATACATTTCTGGATAATCATTATAGTTACAGGTCT
GATGGAATTATCATTTTCAATTAGAGTTGCATGTTCTCTTAGAATTATAGTTGCATATAATTTATCTTTTCTCTCGTTATATTTTTT
TCATTGGTCCTTTTTGACCCATACAACTAGTTAACTGTCTGACTTTTTGCTTTCTGACCCTGCATACTAAATCAAACTGGTTAT
TGGTTGTCTTGGTGCATCTATTTATGTAGTAGTTTCATGGTTTATTACATTGTGCGCATCAAGTTTCATGGTTTATTACATTGAG
CTGGTCATTAGTTTTGTAGTATTTTATACATTTGATATTACCATGTTTTTTAAACTCTCCTGGTTTATTGGGACGTGGATATGCAA
ATTAGTGAGGATGTACTTGTTGATATAATCGTTTGAGACGTTAATATGAAATAGTGAATTGATTGGACCGGCAGTAACCGAGTT

CTTGTTATACAATAATACCAATGACAGGGATAGACAAGGATGGTAGAGAAAGTAGAGACAGAGGAAGAGAACGTAATGATG
GGGAGAGCAGGAGCAGAGAGCGAAGTGACAAAGAGAGGCAAAGCAGTTGGAATAGGGACAGAGACAGGGACAGGGGAA
GAGATCGAGACCGAGACTGGGACTATGTTGACCGAGACAGAGATTATGGACGAGATCGGGACCGTAGTAGACACAGATATT
AAAAATAGTAGCACTCAGGTATTGGCTACCATGCTTTGTCGGACGATTCCAATTGCTTTGAATTAAATCCTTAAATTTGGTGAC
AACTTCCTGTAGTCTGATATTAATGCCCATTTTGCTTGGCATGGAGATTTTGTACAACTTATGACATTTTTCTTTTGACTATTTC
ACAAGGACAAGTTGCTGTTTGAGAATGTTTGCAAAAGCTCGAACCGTTAGTTGTCATCACCTAATTTCTTGTGTAACTTGAAT
GAAGTATTTTCATTTGCTATAAGACCGAATGAAAGTATTATCGGGAATGCTTGATTAGACTCGAACATAAATCTTATGTAATATA
ATTCA

>VrTAF15-2 LOC106760812

AGCTACTTACTTCGATCTGCTCGATTTTCGAGGCCCTAATTGGCAGTATATAAACCACCTCAGCCTCTGCACTCATCTGCACCA
CACCACACTTCAACAACATCTCTCTCCTTATTTAACTTCTCCAGCTACTTCCACTCTCATTCTTCACAGGTGAATATCACTCCT
TCCCTCCTTTTCAGTTTTAACGTTTCTCTTCTTCTTTTCCCATGCACTCTCTCATTCTTTTACGTTAACATCTACACTTCCATGC
ACCGCCAACACAATTTCTATAATTCTGCTTTAATAACGTGCTAAAATAAACACACCACAAATTCTTTCAATACTAATTCTATATA
TGCATGGTGGAATCTGGAAACTATGAAGGGAAAATGAGCAGACCAGGAGATTGGAACTGCAGGACATGCAACCACCTCAA
CTTCCAGAGAAGAGAATCGTGCCAGCGATGCGGGGAGCCAAGGAGTGCCGGCGACTACGGCGGCGCCTTCGGAGGCAGAG
GCTCTTCCTCCTTTGGCTTCACCACTGGCCCTGATGTTCGCCCCGGTGACTGGTACTGCACCGTCGGAAACTGTGGAGCCCA
CAACTTCGCCAGCCGCTCCAGCTGCTTCAAGTGCGGTGTCTCCAAGGAGGACTCCTCCACCGGATCATACGACCTCGACATC
ACCCGAATGAGACCCTACGGCTTCGGCAGCGGCTCCTCCGCCCGACCCGGCTGGAAATCCGGTGACTGGATATGCACCAGG
TACATATATCACTTCCATTCAGTATCATACTATACCAAGCAAATTTAATCATATGTTTTTTAGTCAATTGTTGAATGGTTTAAACA
CATTTGAGATAAAGAAAAAAATTGCAACAACAATGCCAAAATTCTTATTTTCTTGATATATATATCTTAGAATTTCTCCTATGCC
AAGTAGCTTTAATTATCTGTAAGCATGTAAATTATATCTACTACCTAAGTTTGTGATAGATATGTTACAGTTTTATTAATTATTTTA
TTATCTGTGTTTGTTTAATAATGATTAGTATAATTTAAGAAGAAAAAGAAGTATGAGTAAATAAAACAGTAGTAGTGATATTGA
AGAAGTGGTGCGATTAAAATGTGCAGGTCTGGATGCAACGAGCATAACTTCGCCAATAGAATGGAGTGCTACAGATGCAATG
CACCCAGGGACTCTAATAGTGGCAGACCTCCCTATTCATCGTAGAGTTCCTGATCCATGTGGTAATTGCAATCATCTTTATAAT
CAAAATCAAAATAATTTCATCATGCTATATATATATATAGCTCTCTGTTTGTGTATGCAGAATATTATAGATAGTTACATGCACTG
ATGACCCTTTATTTTGTAATTTACAGCTTTGCTGTCCAAGGAGAGAATCAAGAGGCTATCCCTATTTCCCAGTGACAGTTACTT
TTTTTTGGACTCTTTCCTCCACATGTTACTTTCATCATCATCATCATCATCTCATGCGGATATTTATTTTTGCTTTTTTTTTCTTTC
TTAATTTGATTTTCTTCCAGCTAGGAAGCTTTAGGTGAGTTATGATTTTGAGGTTTTGTTAGCGCTGAGTGATTCTGGTATATG
ACTCAGCAGCTAGCCCATGCACTCTTTTAAGTATCGACAATTTAATATCAGTGTTAATGTGTTTTGAGAGGGAGCTAGCTATAG
CTAGCTAGCTTCTAAGCCTCACTATGATTTGGTGCAAAGCGTTCTTTATCTAATTCTCAGATATGTGTTCAGAAAAGA

>VrTAF15b-1 LOC106752587

CCCTGTATGCTACGTTGTACTTGTACAACTTCTCTTCCTCTCTTTACCGTCTCTACAATTCCTTATCGGATTCCAATCATGTCTG
GGAATTACGACCAAGATGGTGGCGGATATGGGCGTAACGACGGCGGTGGTTATGGTGGTCGGGGAGGTGGAGGATTCGGCG
GACGAGGTTTCGAATATTCTCTCTATTTTCGCTTCCTTATCTTTTTTTAATCGTAAAATGTCTTAATTATGTGTTAATCGCAACA
GGTGGGTATCAAGGCGGGGATCGTGGTGGTAGAGGTGGTGGCCGCGGCGGCGGAAGTGGTAGAGACGGTGACTGGCGTTG
CCCTAACTCAAGGTTTGAGATTTTACCCTTCGATTTACTTTTCTTATTTCTGCGCCCCNNNNNNNNNNNNNNNNNNNNNNNN
NNNNNNNNNNNNNNNNNNNNNNNNNNNNNNNNNNNNNNNNNNNNNNNNNNNNTTTTAATACACCTAATCATTCTTATGTTC
TGTATTTGATTGGAAAATTGGTATTATTATATAGTTGTGGGAATTTGAACTTTGCGAGAAGGGTTGAATGTAACAAATGTGGTG
CTCCTTCTCCTGCTGGTGCTAATGATCGTGGTGGAGGTGGTTATAATAGAGGGGGATATGGCAACAGTCGTGGGGGTAGATCT
GGTAACTATGATGGAGGAAGAGGTAATGGTTATAATAGTAGTAGGGGGAATAACAATGTTGGAAGAAGTGGGGGAGGCAAC
AGAGGTAGTCAAGGCAGAGAAGATGGTGGCTATGGTCAAGTTCCTGCACCTGCAGCCCAATCTTATGGTGGGGCTGGTGGA
AACTATCCACCTGCGTACAATTCTTCTGGTGGGAGTTCAAATTATGAAACTGATGCAGTTCCTCCGCCTGCTAGCTATACTGG
TGGACCTGCATCTCACCCTCCACCATATGGGAGTAATACTGGTGGTTATGGTGGCGGAGATGCACATAATGGTGGTAGGTCTG
GGCAACCGGTTGGATATGATAGTGGTTATGGTGCCGGTAGTCAAGGTGGATTCGGTGGAGCTCCTGATGAGCCCCCTGCTAA
GGTGAAGCAATGTGATGAGAATTGTGGGGATTCCTGTGACAACTCTAGAATCTACATATCAAACTTACCTCCGGATGTGACTA
TTGAAGAATTGAGAGAGCTTTTTGGAGGCATTGGACAAGTAATATTCTGATCTGTTGACTTTTGTGCGAGCTCTATAAAGAGA
GTTATTTGTTCTATGAGCAATGCTTTGAATGAAAATACCTTTTTCAAAGTAAATTATCATATCATCCATTTTCTTACCGAACATG
CATGTGTATGTCAGTGAATTCTGCATTATTTACACTTGAAATAGTTGACCCCCTCGTGGCTTCCTGAGCATCTTTGTTAATTTTT
CTCGTGAAGTGACAGAACGAGCTTTAGATTTTGTTTCCTGTATTTATTTGCTTATGTCTGATTTTGCAGATGTGTTGAGCTTCA
CACTCTCATGTCTATTTAATTGTTTAGGTTGGAAGAATAAAACAAAAGAGGGGGTACAAAGATCAGTGGCCTTGGAACATAA
AGCTATACACTGATGAGAAAGGAAACAACAAGGGTGATGGTTGTCTTGTGTATGAAGACCCTTCCGCAGCACATTCTGCTGG
CGGTTTTTACAATAGTATTTGATCTAATTGCTACTACCCCTACTTTTGTGACTACAGTTTATCTTCTATCATAAAGAGACACTTA
ACATTAATCTTTCATCTCAGATTATGATTTGAGGGGTTACAAAATCGCTGTTACAATGGCTGAAAAGTCTGCACCAAAAGCCC
CACCTGCATATAACCATGGGTATTCCATATTTCAATTGTTCATGATTTTTCACACAGTTGCTAATTGTTGCCATTGAAAATGACA
GCTTTTGAAATGCGTGAATGAGTTACTCAGAATATAATAATTAATTGATGAAAAGGCTGCTATTGATTTTTATTCTAGGGGTTG
AGTTGATTAGAGTGGGATGTATTTTGCACTATAATAGTTTTAAATGTATTATACAAGTGACTCCTAATTCTCAGAAATAAATGTT
TGTGTTTTTAAATTAATGTACTAACTTTTGTTATATGTTTTTAAATTCCACAGGGGCAACAGGGGTGGCTATGGTGGAGATAGA
CGCAGAGACAATTATAGAGATGCAGGTGGTTCTGGTCCAGATAGGCGAGACAATTACGGTGGGAATCGTTCACGACCATACT
GAGGGCTTCAACAGAGAACATTTTATGTACTATGTTGTATGGTCTGAGGGATTTGTTGTATTCTATTGGCGGGTAACTTGAGAT
GATAGCAAAGAGGTAGGTTGTTCACTTACTTTTACGTGACCTCTTTGCTCCTTTTTTATTTTGTCACTTGCTGGTGGGATTTTA
ATTTTAGGCGGAAATTATTTTGGTCATGTGAATGCTTTTAAAGGGGCAAGTGCATAATCTCGAAGCTGCTAAGGTAGGTTTTA
ATTTCCTTTCTAGTGACTCCTGCTTTCATGAAAACATGTTTTATTCTTAGCTGTGTTATTTTCTAGATAAATGCTTGCATCCTTA
GCTCATCTGGTTCAAACAGCTGGTGGGATTAAAGGGATATTTTATGTTAAGTCATAGTTCCATGGATCAAGTAATCCCAAGTA
GAGAATA

>VrTAF15b-2 LOC106752498

CTTTGAGTATGTGGCCCATTTTATTTCACAGTGCTTTCTTTACATCAGTTCACAAACCCGTTGCTAAAAATCTCACTGATTTTT
CGTCTTTCGCGGTCCCAACGAACCCTAGATCGGATCATGTCTGGAGCATACGGTCAAGACGGCGGCGGCGGCTCTGCGCCA
CCGTCATATGGAGCCAGCGGTGGATACGGTTCCGGTGGTGGTAGTTATGGAGGCGGAACCTATGGAGGGGGCGGTGCCGGT
GGAGGATACGGTGGTAACGATGGCGGTGGAGGTTACGGAGGGAAAAACAGCGGAAACGGCGGCGGATACGGTGGTAACGA
TGGCGGGGGTTATGGAGGAAGGAGCGGCTACGGTGGAAACGATGGCGGAGGCTATGGAGGAAGAGGCGGTGGTCAAGGTG
GCCGGGGAGGTGGAGGATTCGGTGGTGGATATGGTGGTCGAGGTTTACTTTTTGCTGCTTTTTCTATCGTTTTAACTTTGAAT
TTATTTTTGCCCTATCGTGTTCATATTTGATGTGAAATTCTGCAGGTGGAGGTGGAGGTGGAGGTGGAGGTGGCGGTGGCGG
TGGCGGATATCAAGGTGGTGATCGCGGTGGTCGTGGCGGAGGTCGCGGCGGAGGCGGCCGTGGCGGCAGCGGCAGAGATG
GAGACTGGCGTTGCCCTAACCAAAGGTATTTGTTTTCTTACTGTTTAAATTTTGCTCTTGAAACCGTTTCTCTTTTTAAATTGG
ATATAAAACTGTGAGAATTGACTGCTTTTGATTTCTTTGTTTGTTCATTTGATTCGGAATTGCTATTATGTGTAGTTGTGGGAAT

TTGAACTTTGCGAGAAGGGTTGAATGTAACAAATGCGGTGCTCCTTGTCCTAATCCGAATAGTTCCAATGAACGTGGTGGAA
GTGGTGGGGGTGGGGGTGGCTTTAGTAGAGGCGGAGGTGGCGGTGGGGGGTATGGTAACACGCGAGGGGGAAGATCTGGG
AACTATGATGGAGGGAGAGGTAATGACTATAACAGTGGAGGGGGTCGATCTGGTAACTATGATGGTGGAAGGGGTAATGATT
ATAATAGTGGAAGGGGTGGTAGTAATGATGGTAGAGGTGGTTCATATAGAGGTAATCAAGGTAGAGAAGATGGTAGCTACGG
TCAAGTTCCCGCTCCTAATGCCCAATCTTATGGTAGTGCTGGTGGAAGCTTCCCACCCTCTTACAGTTCTTATGGTGGGAATG
CAAGTTATGGAACTGATGCAGTTCCTCCACCTTCAAGCTATACTGGCGGACCTAATTCCTATCCTCCATCGTATGGGGGTAATG
TCGGAGGTTATGGGGGAGATAATCAAGGGAATGGACGGAGTGGTGGTAGATCTGGGCCTCCTTCTGGGTTTGACAATAGTTA
TGGTGCTGGTAATCGAGGTGGATTTGGTGGGTCTCCTGCCGAGCCCCCAGCTGCAGTGAAGCAGTGTGATGAGAATTGTGAT
GACACTTGCGACAACTCTAGAATATACATCTCAAACCTTCCACCAGATGTGTCAATTGAAGAATTGAGGGAACTTTTTGGAG
GCATTGGACAAGTAATATTATGATCTTTTTTACATTAGAGATTTTCACAAGCTTTAGCTTTAACGAGTCATTGTGAATTTTGTTT
GATGAATCTTATATATAATTAAATCTAATGCCACTTTCAGCATAAGCATGTTGTTGAGTTATATTAGCAATAGTGTTTTATACAAG
AAATATTTCCTATCTTATGGGGAAATAAGATTATTTTTCCGGACTATTCCTTGAAAGAGGCTTATTGTGTCAGTAATTTTGTTGC
ATGTTTTAACTTTATAGGTTGGAAGGATAAAGCAGAAGAGAGGCTACAAAGATCAGTGGCCTTGGAACATTAAGATTTACAC
AGATGAGAATGGAAATAACAAGGGAGATGCTTGTCTTGCTTATGAAGACCCTTCTGCAGCACATTCGGCTGGCGGTTTTTAC
AATAGTACTTAATTGAACAATTTCTTCTTTTTATCATTCCGTCTAGCTTATATATATGGAGAGAGAGAGAGAGACACACACAC
ACACATGGTGCTTAACATGTAAACATACACTAATAATGAATATTTCATTTCAGATTATGATTTGAGGGGTTACAAAATCAGTGT
TGCAATGGCAGAAAAATCTGCTCCAAGAGCTGCACCTGCTTATAACCAAGGGTATGCATTACATACTGAATTATTTTGGACAT
CTATAAGAGTATTTGTCAATTATTTGCTATGTAAATTGTTTGGTTGATAAATTATTTTGGGAACTGTTCTGTAAATTTATCAATTA
TGTGCTATATATATTTTATTACACAACTGATCGATGTGTTTGAATGTTTCTTTTTTCAAAAATTTATTTCTCTTCTGAAACTGAAT
ATTCTTTAATTAATTGTTGTTCTATAGGGGCAATAGGGGTGGCTATGGTGGAGATAGACGCCGAGACAACTATAGAGATGGAG
GTGGCTCTGGGCCTGATAGGCGTGATCATTATGGAAATCGTTCTCGTCCGTACTAGGAGCTTGGACATTGAACGTTTTATGTA
CTATGAATTGTGGGCTGAGGGTTTAATTATTTCAGTTGGCTGGTTTCTTGAACTGGAAGCAAAGAGGTAGGTTGTTCACTTAC
TTTGAGCAATACCTCTTCCGCTCCTTTTTTGTTTCAATTGTCTAGATGGTGGGTTTTGAATTTAGCCAGGGATTAATTTAATCAT
GTGAATGTTTTTATAGAGGCAAGTGGATAGTCTCCCAATTGCTTGCTGCTAAGGTAGGTGTTGAATATTTCAGGTGAGTGCAG
CTTTCACGTAAATGTGCCTAATACTTGCTTGGAAATTCCTTGTGTCATAATGTGGATAAATGCTTGCAGTTCTGGCTCATCCCA
TGTGGCATCTGTGGTACAAGCGGGTGTTGGCATTAATTAATAGAATGTTTTTATGTTAGTCATTGTTCCATAAATCAATAAATCT
TAATTACAATGTTTCAAGTTTCAAGTAAAATTGGTTGTTTGTAAGGAATGGAGTAAATCGGGCTGCCATTTTACTTTAAATTGA
AGCCTTTTGTAATCAAATATATCATGAGTTATTCTATCGTAGTCTGTTTGATCTCGAGGCTGTCTGAAGTCTTGTATTTTTCTTT
GTTTACATGCAACTTTTCAGGAGTTTTTATTTAACTGTTTAAATTGACATCCAAGCAACTCAAGGTAAAGCCAAGCCTTTGAG
CTGATATTTAACATCTGAAAAGAATTCAATTTACTCTAAATTGTTTTCATTTGAAGTGTTGAAAAACCACACTTTTGTTGAAAA
TTTTCTGTGTAGAGTTGCAAAGCTGTTGCATTTTCTATGGATACAGCCAATCAAGTTTTCAATCTAGGTTTTTTAAATATTTTTT
GGAGATTAACTAGATGAAATAGTTCCATATATTCTTATATGATTAAGTAGAACATGAACTTGTATTAATAGCATATAAATGTGAA
ATTTGACTCTTGCTTATACTTTGTCTGATGTAAATAATGCAGAAGTCATTCTTGTTTAGTCTTGAATCTTGTTTAGCTATCTTATG
GTTTGCATTCTTATTTTATATTTTTTGCTTTTGCACTGGATTTGGTCCATTTGTAACTGTAATTTACCGAATAGCCAATAGGGAA
GTCAAGTATCTTTTGGACTTCATAATACGGCATGTGTTGCTTTTGGCAAGCTAACTATTGGAGTCCGCACTTCACAGTTGCATT
CCTCTGCCACTTATTCTCTGCAGTCCAAGTTTGTTTCTACTTTCTGATAAAATTAGTACACAGTTGCACTGCGAGTTTGATTTA
ATGTTCACACATCTATATCCGGTGGCTTTTGCGTATTTGGTACTTGCGAGCAGAACTTTCTTCCATGGGGCTATTGTGGGTGAC
AAGTGTCGTTGCTTCCAAACCATGCTTGTTCATTTAGTTCCTTACCAATGGATGCTATAAAATTCAAATGTACTTTGACCCGTA
GTGTAGATTGTGATAGCCTGTTTGATTTGTTGGTTGGGAATTTTTACTTTTTTAAACCATGTCATTCTTCTAGTTATTAAAATAC
CCCTTCCTTTCTGACTATTATTCCGGATTTTGGGTTCTCTGTTTTTGAAGGGATCATCGGCGTTGTAATAATTTTCATATAATTTG
GTCGATATAACGGTCTATCAAATCCTGGAATCGGACAGCATGTTTTTATTGTAACTAAATATAGTCATTTGCGTACTGCATTTGC
GGGAGTTTCAA

2. CDS sequences of *VrTBP* and *VrTAFs*

>VrTBP

ATGGCTGACCAAGGATTGGAAGGGAGCCAGCCGGTGGATCTACAGAAGCATCCTTCTGGGATCGTGCCTACTCTTCAAAATA
TTGTGTCCACTGTCAATTTGGACTGTAAGTTGGACCTCAAAACAATTGCACTACAAGCTCGTAACGCTGAGTACAATCCCAA
GCGTTTTGCTGCTGTTATTATGAGGATAAGAGAGCCGAAAACAACTGCACTTATTTTTGCTTCTGGCAAGATGGTTTGTACTG
GAGCTAAGAGTGAACAACAGTCTAAATTGGCAGCCAGGAAGTATGCTCGAATTATCCAAAAGCTTGGTTTCCCAGCCAAATT
TAAGGATTTCAAAATTCAGAACATTGTTGGCTCTTGTGATGTCAAGTTCCCCATTCGGCTGGAGGGTCTTGCGTATTCTCATG
GTGCTTTCTCAAGTTATGAACCAGAGCTGTTTCCAGGTCTAATCTACCGTATGAAGCAACCTAAAATAGTGTTGCTTATATTTG
TCTCTGGAAAAATTGTTCTAACAGGAGCTAAGGTGAGAGATGAGACGTACACTGCCTTTGAAAACATATATCCTGTACTTAC
CGAATTCAGGAAAAACCAACAATGA

>VrTAF1-X1

ATGGGTTACGATTCGGCTAGCCCCTCGCAGGATGGGAGGGACGAAGATGATGAAGAAGAATATGAGGAGTCTGGCAAGGGT
AATCGGTTTCTTGGGTTCATGTTTGGAAATGTAGATAATTCTGGTGATCTCGATGTTGACTATCTTGATGAGGATGCAAAGGA
GCATCTTTCTGCATTAGCCGATAAGTTGGGTCCATCACTGACAGATATAGATTTGTCAGGAAAATCGCCACAAACACCACCTG
ACGTTGTTGAACAAGACTGTGATGAAAAAGCTGAAGACGCTGTTGATTATGAAGATATTGATGAAGAGTATGATGGTCCAGA
GACAGAGGCTGCCAATGAAGAAGACTATTTATTGCCAAAAAAAGAATTTTTCTCTGCTGAAGCATCTGTATGTATGGAATCC
AAAGCTTCTGTATTTGATGATGAAAATTATGATGAAGAATCTGAGAAGGAGCAAGACTTTCTGAATGAAGATTCTAAACCTG
ATAATATCTCCTTACCTGAGGAGCAGGAAGAGACTTTGGTAGAGGCATCTAAAGAAGAGAGTGCTCTTGAACGTGAATTACA
TGTTGACTCACTGCAAAGTGAAGAATTGGATGCTGATGTACAAAAACCTGAGGAGGAAGGGACTGAAGTCCAGAAAAGGT
CTATGGCTATGCCGTTGCCTATTTTATGTGTGGAAGATGGTGTGGCAATTTTACGCTTCTCTGAAATCTTTGGCATTCATGAAC
CTCTCAGAAAGGGAGAAAAGAGAGAACACAGGCAACCTATTACTAGAGATAGATACAAGTCTTTGGATTTTACTGATGATTT
TGTAGAAGAGGATGAAGAGGAATTCCTCAAGGGTTCCTCCCAGAGTCTCTCACAGACTAAACAGGTTTCTGTAGTTCATAAT
GATGTATCAGAAAGCAACGATGTTGACTTGGAGTTTCCAAAATTTGGGTTTCTTCATACTGAGCCCTCAGTGGCCAGGAAAG
ATGATCATCAATCGAAGGACTCGTGTCATAGTGCTGAACCAATGAAAGGGGATTTTGAAGAAGACCTTTCCTGGAAAGATCA
TCCTTTCATATGGACCAACTTTTATCCTCTTGATCAGCAAGACTGGGAAGATGAAATCATTTGGGGCAATTCTCCTGTTCCAA
GTAATAATAATATTGAAAGCTGTGAAGTTTCTGGACCTGAGTTGGGAGTTTCTGGTGGCAGTGAAATAGAAATTGAAAGTGG
GATCCAGAATATTCAGCTGGAGCATCACAAGATACTGGAAGATAAAGATCATAATGTCTTGCTCAGCTCTCCTGTCTCGTTGG
AGGCCTTTGGCTCAAGGGATTCTTCTGAAGCTAAAACCAATCTAATATCTAGAAGTCTTTTTCATCCCCAACTTTTAAGGTTA
GAATCCAGATCTGAAGTGGATAGTTCTAGTCTTCCAGATGGAAAAGAGGGGGAGATATCTAAACATAATCAAAGTGGTCAAA
TTACACGTTTTAGCAAGGCTATATCACAAAATAGAGACATGGTGGAGGGTTCCTGGTTAGACGAGATAATATGGGAGGAGCT
TGATCAGCCTATGGTGAAACCAAAGCTTATCTTTGATCTTCAGGATGATCAAATGCACTTCGAAGTTTTGGATAGTAAGGATG
GTGCACATCTTCGTCTTCATGCTGGGGCTATAATTTTAACTCGTTCTTCAAAATCAAGCAGTGGGGACTCATCTGAAGTACCA
GGACATGGAAGTCAATATGGATGGCGATATGTGTCTAATGACAAACATTATTCAAATCGTAAAACTTCTCAACAATTGAAATC
AAATTCCAAAAAACGCTCGGCACATGGTGTCAAAGTTTTCCACTCTCAACCTGCGTTGAAGCTGCAGACAATGAAATTGAA

GTTGAGCAATAAAGATATTGCAAATTTTCACCGGCCAAAAGCATTATGGTATCCCCATGACAATGAGGTTGCTGTCAAAGAA
CAAGGGAAATTGCCAACACAAGGACCCATGAAAATTATTATAAAGAGTTTGGGTGGCAAGGGAAGTAAATTGCATGTGGAT
ACTGAAGAAACTCTCTCAACTGTTAAAGCAAAAGCTTCCAAAAAGCTAGATTTTAAGGCATCAGAAACAGTGAAAATATTTT
ATTTAGGGAGGGAGCTTGAAGATCAGAAGTCACTTGCCGAACAAAATGTTCAACCAAACTCCTTGTTACATCTTGTTCGTTC
AAAGATACATTTGTGGCCAAAAGCACAGAGGGTTCCTGGGGAGAACAAGTCCTTGCGTCCTCCTGGGGCATTCAAGAAAAA
ATCTGATCTGTCTGTAAAAGACGGACATGTTTTTCTGATGGAGTATTGTGAAGAAAGACCTTTACTTTTGAGCAATGTTGGAA
TGGGTGCAAGACTTTGTACATACTATCAAAAATGCTCACCAGATGACCAATCTGGCTCTTTATTACGTAACACAGATAGTAGC
TTGGGGCACGTTATTTCTCTGGATCCTGCGGATAAATCTCCGTTCCTTGGAGATTTGAAACCTGGTTGCTCTCAGTCATCACT
AGAGACAAATATGTATAGAGCACCCGTATTTCCTCATAAAGTTCCACTAACTGACTACCTGCTGGTTCGCTCACCAAAGGGA
AAGCTATCATTAAGGCGCATTGATAAAATTAATGTTGTTGGACAGCAGGAGCCACTCATGGAGGTATTTTCACCAGGAAGTA
AAAATCTTCAGACTTACATGATGAACAGGCTGTTGGTACACATGTGCCGTGAATTCCAAGCAGCAGAGAAGCGGCACTTGC
CTCCTCATATCCGTGTTGATGAATTTCTCTCACAGTTTCCTTACCAATCGGAAGCATCATTTCGTAAGAAAATCAAGGAATATG
CAAATTTACAGAGGGGAGCAAATGGACAGTCGATTTTGGTTAAAAAGAGAAATTTCCGCATGTGGTCTGAGGATGAATTGA
GAAAAATGGTTCTGCCGGAGCTTGTTTGTGCCTATGAAAGCATGCAAGCAGGCCTCTACCGGCTAAAACATTTAGGAATAAC
TGAAACACACCCTACAAATATTTCATCTGCAATGAGTCGGCTCCCTGATGAAGCAATAGCATTGGCTGCTGCATCACACATTG
AGAGAGAACTGCAGATTACTCCTTGGAACTTGAGTAGCAATTTTGTTGCTTGTACAAGCCAGGGTAAGGAAAATATTGAGCG
AATGGAAATTACTGGCGTTGGTGATCCTTCTGGTCGAGGCATGGGTTTCAGCTATGCTCGGGCACCTCCAAAGGCACCAGTG
TCTAGTGCAATGGTGAAGAAGAAAGCAGCTGCTAACCGTGGAGGTTCCACTGTTACTGGTACAGATGCTGATCTGCGTAGAT
TAAGCATGGAGGCTGCGCGAGAGGTTCTTCTTAAGTTCAATGTTCCTGAGGAAGTCATTACTAAACAAACCAGGTGGCATCG
CATTGCTATGATACGTAAACTTTCAAGTGAGCAAGCTGCATCTGGGGTTAAGGTTGACCCGACAACTATCAGCAAATATGCTC
GTGGCCAGCGAATGTCCTTTCTTCAGTTACAGCAGCAGACTAGAGAAAAATGCCAGGAGATTTGGGATCGACAAGTTCAGA
GTCTGTCAGCTGTAAATGCTGATGAGAACGAGAGTGATTCGGAAGGTAATAGTGATCTGGATTCTTTTGCTGGAGACCTGGA
AAATTTACTTGATGCTGAGGAGTTTGAAGAGGGAGAAGAAGGTACAAATGACTTAAAACGTGACAAGGGAGACGGTGTTA
AGGGTCTTAAAATGAGAAGACGCTCAACTTTGGCTCAGGCAGAGGAGGAAATAGAAGATGAAGCCGCTGAGGCTGCTGAG
TTATGCAGGTTGCTGATGGATGATGATGAAGCTGATAGGAAGAAAAAGAAGAAAACTAAAGTAACTGGGGAAGAAACAAG
ATTGGTATCAAAGATGCAATCAAAATTTGCCTTCGACAATGCTGAACAAGTTAAACAAATAACAAATAGTTTACAATTAGATG
GAAATATTCCCTTGAAAGAGGACACGATTACAGATCTTAGGGAGGAGGAAAATTTTGGTGCTAAAAAAAGCAAATCACTGA
AGGTCAATAAAGCCAAGAAGAATGATATTGCACCTATTTCTCTTCCCAATAAAAAAATCAAATTGAATATGGGAGAAGGAATT
AAGAACCAGGTGTTTAAGGAGAAAAAACCATCAAGGGAAACTTTTGTTTGTGGAGCATGTGGTCAGCCTGGGCATATGCGA
ACAAACAAGAACTGTCCAAAATATGGTGAAGATCTTGAAACTCAACTTGAATCTGCAGATATGGAAAAATCATCTGGGAAA
CCCATATCTGTGGACCCCTCCAGTCACTCCCAGCCTAAAACCGCCTCCAAAAAGTCAAGTTCGAAAAGCAACTCAAAGATT
ACTCCTGTTGACAATTCGGCAAAAATTCCACTGAAATTCAAATGTGGCTCCACAGAGAAATCTTCTGATAAACCTGTAACAG
AGACCCTGCAGAACTCTGACAAGCCAGTCACTTCGGATTCAGAAACTGCAAAGTCTGCTAAGGTTAATAAGATAATTATTCC
CAAAAAAGTGAAACCAGATGATACACAGGCTGAATCTCGTAAGCATGCTGTTGTTATAAGGCCTCCTACTGAATCAAGTAGA
GGGCTTCCTGCTGATTCAGGTAGAGGGCCTCCTACTGATGCAGGCAGAGGTCAGGTTGATTATCAGAAGTTACCAATTAAAA
TACGACCACCAACAGAGGAGCAAAGTCACAAAAAGATTGTTATAAGACGTACGAAGGAGGTTATTGATTTAGAACTGGATA
GTCCTGGTGGAAACACTGGACTTCAACACAGAAAGACAAAAAGAATTGTTGAATTGTCAAATTTTGAGAAACATAAACAGG
AGACCGTGTATGGAACTGGAGCTTTTCCAAAATGGAACACTAAAGAGGACAGAAGATGGTGGGAAGAGCAAGAGAAACGA
AGAAATGATGCAAGACTTAGAGAAGAAGACAGAGCAAGGAGGCATCACAAAGAAGAAATGAGGATGCTCAAAGAGCAAG
AAAGGTTAGATGAGATAAAAAGATTTGAAGAAGATATCAGAAGAGAGAGGGAGGAAGAAGAACGGCAAAAGGCTAAGAA
GAAAAAGAAGAAGAAAAAGCCTGATTTAAGAGATGAGTATTTAGATGATCCCAGAGCAAGAAGACATGATAAGAGAATGCC
TGAAAGAGACAGGAGTGGAAAAAGGAGATCTGTTGCTGAGTTAGGAAAGCTTAGTGCAGATTATATGCCGCCAACAAAACG
CCGAAGAGGGGGAGGGGGAGAGGTTGGTTTGGCAAATATCTTGGAGGGCATTGTGGAAACGATGGTTAAAGATAGGTACGA
GCTATCGTATCTTTTCGTGAAACCAGTGTCGAAGAAAGAGGCTCCTGACTACCTGGACATTATAGATACGCCTATGGATCTTT
CCAGAATCAGGGAGAGAGTACGGAATATGGAGTACAAGAGCCGAGAAGATTTCAGGCATGATGTTTGGCAGATTACTTTTAA
TGCACACAAATACAACGACGGGAGAAATCCTGGAATTCCTCCCCTTGCAGATATGCTTTTGGAATATTGTGATTATTTGTTGA
ATGAGAATGATGATAGCCTCACTTCAGCAGAAGCTGGCATTGAAACTAGAGATTCCTAA

>VrTAF1-X2

ATGGAATCCAAAGCTTCTGTATTTGATGATGAAAATTATGATGAAGAATCTGAGAAGGAGCAAGACTTTCTGAATGAAGATT
CTAAACCTGATAATATCTCCTTACCTGAGGAGCAGGAAGAGACTTTGGTAGAGGCATCTAAAGAAGAGAGTGCTCTTGAACG
TGAATTACATGTTGACTCACTGCAAAGTGAAGAATTGGATGCTGATGTACAAAAACCTGAGGAGGAAGGGACTGAAGTCCA
GAAAAGGTCTATGGCTATGCCGTTGCCTATTTTATGTGTGGAAGATGGTGTGGCAATTTTACGCTTCTCTGAAATCTTTGGCAT
TCATGAACCTCTCAGAAAGGGAGAAAAGAGAGAACACAGGCAACCTATTACTAGAGATAGATACAAGTCTTTGGATTTTACT
GATGATTTTGTAGAAGAGGATGAAGAGGAATTCCTCAAGGGTTCCTCCCAGAGTCTCTCACAGACTAAACAGGTTTCTGTAG
TTCATAATGATGTATCAGAAAGCAACGATGTTGACTTGGAGTTTCCAAAATTTGGGTTTCTTCATACTGAGCCCTCAGTGGCC
AGGAAAGATGATCATCAATCGAAGGACTCGTGTCATAGTGCTGAACCAATGAAAGGGGATTTTGAAGAAGACCTTTCCTGG
AAAGATCATCCTTTCATATGGACCAACTTTTATCCTCTTGATCAGCAAGACTGGGAAGATGAAATCATTTGGGGCAATTCTCC
TGTTCCAAGTAATAATAATATTGAAAGCTGTGAAGTTTCTGGACCTGAGTTGGGAGTTTCTGGTGGCAGTGAAATAGAAATT
GAAAGTGGGATCCAGAATATTCAGCTGGAGCATCACAAGATACTGGAAGATAAAGATCATAATGTCTTGCTCAGCTCTCCTG
TCTCGTTGGAGGCCTTTGGCTCAAGGGATTCTTCTGAAGCTAAAACCAATCTAATATCTAGAAGTCTTTTTCATCCCCAACTT
TTAAGGTTAGAATCCAGATCTGAAGTGGATAGTTCTAGTCTTCCAGATGGAAAAGAGGGGGAGATATCTAAACATAATCAAA
GTGGTCAAATTACACGTTTTAGCAAGGCTATATCACAAAATAGAGACATGGTGGAGGGTTCCTGGTTAGACGAGATAATATGG
GAGGAGCTTGATCAGCCTATGGTGAAACCAAAGCTTATCTTTGATCTTCAGGATGATCAAATGCACTTCGAAGTTTTGGATAG
TAAGGATGGTGCACATCTTCGTCTTCATGCTGGGGCTATAATTTTAACTCGTTCTTCAAAATCAAGCAGTGGGGACTCATCTG
AAGTACCAGGACATGGAAGTCAATATGGATGGCGATATGTGTCTAATGACAAACATTATTCAAATCGTAAAACTTCTCAACAA
TTGAAATCAAATTCCAAAAAACGCTCGGCACATGGTGTCAAAGTTTTCCACTCTCAACCTGCGTTGAAGCTGCAGACAATG
AAATTGAAGTTGAGCAATAAAGATATTGCAAATTTTCACCGGCCAAAAGCATTATGGTATCCCCATGACAATGAGGTTGCTGT
CAAAGAACAAGGGAAATTGCCAACACAAGGACCCATGAAAATTATTATAAAGAGTTTGGGTGGCAAGGGAAGTAAATTGCA
TGTGGATACTGAAGAAACTCTCTCAACTGTTAAAGCAAAAGCTTCCAAAAAGCTAGATTTTAAGGCATCAGAAACAGTGAA
AATATTTTATTTAGGGAGGGAGCTTGAAGATCAGAAGTCACTTGCCGAACAAAATGTTCAACCAAACTCCTTGTTACATCTTG
TTCGTTCAAAGATACATTTGTGGCCAAAAGCACAGAGGGTTCCTGGGGAGAACAAGTCCTTGCGTCCTCCTGGGGCATTCA
AGAAAAAATCTGATCTGTCTGTAAAAGACGGACATGTTTTTCTGATGGAGTATTGTGAAGAAAGACCTTTACTTTTGAGCAA
TGTTGGAATGGGTGCAAGACTTTGTACATACTATCAAAAATGCTCACCAGATGACCAATCTGGCTCTTTATTACGTAACACAG
ATAGTAGCTTGGGGCACGTTATTTCTCTGGATCCTGCGGATAAATCTCCGTTCCTTGGAGATTTGAAACCTGGTTGCTCTCAG
TCATCACTAGAGACAAATATGTATAGAGCACCCGTATTTCCTCATAAAGTTCCACTAACTGACTACCTGCTGGTTCGCTCACC
AAAGGGAAAGCTATCATTAAGGCGCATTGATAAAATTAATGTTGTTGGACAGCAGGAGCCACTCATGGAGGTATTTTCACCA
GGAAGTAAAAATCTTCAGACTTACATGATGAACAGGCTGTTGGTACACATGTGCCGTGAATTCCAAGCAGCAGAGAAGCGG
CACTTGCCTCCTCATATCCGTGTTGATGAATTTCTCTCACAGTTTCCTTACCAATCGGAAGCATCATTTCGTAAGAAAATCAAG
GAATATGCAAATTTACAGAGGGGAGCAAATGGACAGTCGATTTTGGTTAAAAAGAGAAATTTCCGCATGTGGTCTGAGGATG

AATTGAGAAAAATGGTTCTGCCGGAGCTTGTTTGTGCCTATGAAAGCATGCAAGCAGGCCTCTACCGGCTAAAACATTTAGG
AATAACTGAAACACACCCTACAAATATTTCATCTGCAATGAGTCGGCTCCCTGATGAAGCAATAGCATTGGCTGCTGCATCAC
ACATTGAGAGAGAACTGCAGATTACTCCTTGGAACTTGAGTAGCAATTTTGTTGCTTGTACAAGCCAGGGTAAGGAAAATAT
TGAGCGAATGGAAATTACTGGCGTTGGTGATCCTTCTGGTCGAGGCATGGGTTTCAGCTATGCTCGGGCACCTCCAAAGGCA
CCAGTGTCTAGTGCAATGGTGAAGAAGAAAGCAGCTGCTAACCGTGGAGGTTCCACTGTTACTGGTACAGATGCTGATCTG
CGTAGATTAAGCATGGAGGCTGCGCGAGAGGTTCTTCTTAAGTTCAATGTTCCTGAGGAAGTCATTACTAAACAAACCAGGT
GGCATCGCATTGCTATGATACGTAAACTTTCAAGTGAGCAAGCTGCATCTGGGGTTAAGGTTGACCCGACAACTATCAGCAA
ATATGCTCGTGGCCAGCGAATGTCCTTTCTTCAGTTACAGCAGCAGACTAGAGAAAAATGCCAGGAGATTTGGGATCGACAA
GTTCAGAGTCTGTCAGCTGTAAATGCTGATGAGAACGAGAGTGATTCGGAAGGTAATAGTGATCTGGATTCTTTTGCTGGAG
ACCTGGAAAATTTACTTGATGCTGAGGAGTTTGAAGAGGGAGAAGAAGGTACAAATGACTTAAAACGTGACAAGGGAGAC
GGTGTTAAGGGTCTTAAAATGAGAAGACGCTCAACTTTGGCTCAGGCAGAGGAGGAAATAGAAGATGAAGCCGCTGAGGC
TGCTGAGTTATGCAGGTTGCTGATGGATGATGATGAAGCTGATAGGAAGAAAAAGAAGAAAACTAAAGTAACTGGGGAAGA
AACAAGATTGGTATCAAAGATGCAATCAAAATTTGCCTTCGACAATGCTGAACAAGTTAAACAAATAACAAATAGTTTACAA
TTAGATGGAAATATTCCCTTGAAAGAGGACACGATTACAGATCTTAGGGAGGAGGAAAATTTTGGTGCTAAAAAAAGCAAAT
CACTGAAGGTCAATAAAGCCAAGAAGAATGATATTGCACCTATTTCTCTTCCCAATAAAAAAATCAAATTGAATATGGGAGA
AGGAATTAAGAACCAGGTGTTTAAGGAGAAAAAACCATCAAGGGAAACTTTTGTTTGTGGAGCATGTGGTCAGCCTGGGCA
TATGCGAACAAACAAGAACTGTCCAAAATATGGTGAAGATCTTGAAACTCAACTTGAATCTGCAGATATGGAAAAATCATCT
GGGAAACCCATATCTGTGGACCCCTCCAGTCACTCCCAGCCTAAAACCGCCTCCAAAAAGTCAAGTTCGAAAAGCAACTCA
AAGATTACTCCTGTTGACAATTCGGCAAAAATTCCACTGAAATTCAAATGTGGCTCCACAGAGAAATCTTCTGATAAACCTGT
AACAGAGACCCTGCAGAACTCTGACAAGCCAGTCACTTCGGATTCAGAAACTGCAAAGTCTGCTAAGGTTAATAAGATAAT
TATTCCCAAAAAAGTGAAACCAGATGATACACAGGCTGAATCTCGTAAGCATGCTGTTGTTATAAGGCCTCCTACTGAATCAA
GTAGAGGGCTTCCTGCTGATTCAGGTAGAGGGCCTCCTACTGATGCAGGCAGAGGTCAGGTTGATTATCAGAAGTTACCAAT
TAAAATACGACCACCAACAGAGGAGCAAAGTCACAAAAAGATTGTTATAAGACGTACGAAGGAGGTTATTGATTTAGAACT
GGATAGTCCTGGTGGAAACACTGGACTTCAACACAGAAAGACAAAAAGAATTGTTGAATTGTCAAATTTTGAGAAACATAA
ACAGGAGACCGTGTATGGAACTGGAGCTTTTCCAAAATGGAACACTAAAGAGGACAGAAGATGGTGGGAAGAGCAAGAGA
AACGAAGAAATGATGCAAGACTTAGAGAAGAAGACAGAGCAAGGAGGCATCACAAAGAAGAAATGAGGATGCTCAAAGA
GCAAGAAAGGTTAGATGAGATAAAAAGATTTGAAGAAGATATCAGAAGAGAGAGGGAGGAAGAAGAACGGCAAAAGGCT
AAGAAGAAAAAGAAGAAGAAAAAGCCTGATTTAAGAGATGAGTATTTAGATGATCCCAGAGCAAGAAGACATGATAAGAG
AATGCCTGAAAGAGACAGGAGTGGAAAAAGGAGATCTGTTGCTGAGTTAGGAAAGCTTAGTGCAGATTATATGCCGCCAAC
AAAACGCCGAAGAGGGGGAGGGGGAGAGGTTGGTTTGGCAAATATCTTGGAGGGCATTGTGGAAACGATGGTTAAAGATA
GGTACGAGCTATCGTATCTTTTCGTGAAACCAGTGTCGAAGAAAGAGGCTCCTGACTACCTGGACATTATAGATACGCCTATG
GATCTTTCCAGAATCAGGGAGAGAGTACGGAATATGGAGTACAAGAGCCGAGAAGATTTCAGGCATGATGTTTGGCAGATTA
CTTTTAATGCACACAAATACAACGACGGGAGAAATCCTGGAATTCCTCCCCTTGCAGATATGCTTTTGGAATATTGTGATTATT
TGTTGAATGAGAATGATGATAGCCTCACTTCAGCAGAAGCTGGCATTGAAACTAGAGATTCCTAA

>VrTAF1-X3

ATGAAGATTCTAAACCTGATAATATCTCCTTACCTGTTCACATATGCTAGCTTTGTTCTTTCAGAGGAGCAGGAAGAGACTTT
GGTAGAGGCATCTAAAGAAGAGAGTGCTCTTGAACGTGAATTACATGTTGACTCACTGCAAAGTGAAGAATTGGATGCTGAT
GTACAAAAACCTGAGGAGGAAGGGACTGAAGTCCAGAAAAGGTCTATGGCTATGCCGTTGCCTATTTTATGTGTGGAAGAT
GGTGTGGCAATTTTACGCTTCTCTGAAATCTTTGGCATTCATGAACCTCTCAGAAAGGGAGAAAAGAGAGAACACAGGCAA
CCTATTACTAGAGATAGATACAAGTCTTTGGATTTTACTGATGATTTTGTAGAAGAGGATGAAGAGGAATTCCTCAAGGGTTC
CTCCCAGAGTCTCTCACAGACTAAACAGGTTTCTGTAGTTCATAATGATGTATCAGAAAGCAACGATGTTGACTTGGAGTTTC
CAAAATTTGGGTTTCTTCATACTGAGCCCTCAGTGGCCAGGAAAGATGATCATCAATCGAAGGACTCGTGTCATAGTGCTGA
ACCAATGAAAGGGGATTTTGAAGAAGACCTTTCCTGGAAAGATCATCCTTTCATATGGACCAACTTTTATCCTCTTGATCAGC
AAGACTGGGAAGATGAAATCATTTGGGGCAATTCTCCTGTTCCAAGTAATAATAATATTGAAAGCTGTGAAGTTTCTGGACCT
GAGTTGGGAGTTTCTGGTGGCAGTGAAATAGAAATTGAAAGTGGGATCCAGAATATTCAGCTGGAGCATCACAAGATACTG
GAAGATAAAGATCATAATGTCTTGCTCAGCTCTCCTGTCTCGTTGGAGGCCTTTGGCTCAAGGGATTCTTCTGAAGCTAAAAC
CAATCTAATATCTAGAAGTCTTTTTCATCCCCAACTTTTAAGGTTAGAATCCAGATCTGAAGTGGATAGTTCTAGTCTTCCAGA
TGGAAAAGAGGGGGAGATATCTAAACATAATCAAAGTGGTCAAATTACACGTTTTAGCAAGGCTATATCACAAAATAGAGAC
ATGGTGGAGGGTTCCTGGTTAGACGAGATAATATGGGAGGAGCTTGATCAGCCTATGGTGAAACCAAAGCTTATCTTTGATCT
TCAGGATGATCAAATGCACTTCGAAGTTTTGGATAGTAAGGATGGTGCACATCTTCGTCTTCATGCTGGGGCTATAATTTTAA
CTCGTTCTTCAAAATCAAGCAGTGGGGACTCATCTGAAGTACCAGGACATGGAAGTCAATATGGATGGCGATATGTGTCTAAT
GACAAACATTATTCAAATCGTAAAACTTCTCAACAATTGAAATCAAATTCCAAAAAACGCTCGGCACATGGTGTCAAAGTTT
TCCACTCTCAACCTGCGTTGAAGCTGCAGACAATGAAATTGAAGTTGAGCAATAAAGATATTGCAAATTTTCACCGGCCAAA
AGCATTATGGTATCCCCATGACAATGAGGTTGCTGTCAAAGAACAAGGGAAATTGCCAACACAAGGACCCATGAAAATTATT
ATAAAGAGTTTGGGTGGCAAGGGAAGTAAATTGCATGTGGATACTGAAGAAACTCTCTCAACTGTTAAAGCAAAAGCTTCC
AAAAAGCTAGATTTTAAGGCATCAGAAACAGTGAAAATATTTTATTTAGGGAGGGAGCTTGAAGATCAGAAGTCACTTGCCG
AACAAAATGTTCAACCAAACTCCTTGTTACATCTTGTTCGTTCAAAGATACATTTGTGGCCAAAAGCACAGAGGGTTCCTGG
GGAGAACAAGTCCTTGCGTCCTCCTGGGGCATTCAAGAAAAAATCTGATCTGTCTGTAAAAGACGGACATGTTTTTCTGATG
GAGTATTGTGAAGAAAGACCTTTACTTTTGAGCAATGTTGGAATGGGTGCAAGACTTTGTACATACTATCAAAAATGCTCAC
CAGATGACCAATCTGGCTCTTTATTACGTAACACAGATAGTAGCTTGGGGCACGTTATTTCTCTGGATCCTGCGGATAAATCTC
CGTTCCTTGGAGATTTGAAACCTGGTTGCTCTCAGTCATCACTAGAGACAAATATGTATAGAGCACCCGTATTTCCTCATAAA
GTTCCACTAACTGACTACCTGCTGGTTCGCTCACCAAAGGGAAAGCTATCATTAAGGCGCATTGATAAAATTAATGTTGTTGG
ACAGCAGGAGCCACTCATGGAGGTATTTTCACCAGGAAGTAAAAATCTTCAGACTTACATGATGAACAGGCTGTTGGTACAC
ATGTGCCGTGAATTCCAAGCAGCAGAGAAGCGGCACTTGCCTCCTCATATCCGTGTTGATGAATTTCTCTCACAGTTTCCTTA
CCAATCGGAAGCATCATTTCGTAAGAAAATCAAGGAATATGCAAATTTACAGAGGGGAGCAAATGGACAGTCGATTTTGGTT
AAAAAGAGAAATTTCCGCATGTGGTCTGAGGATGAATTGAGAAAAATGGTTCTGCCGGAGCTTGTTTGTGCCTATGAAAGCA
TGCAAGCAGGCCTCTACCGGCTAAAACATTTAGGAATAACTGAAACACACCCTACAAATATTTCATCTGCAATGAGTCGGCT
CCCTGATGAAGCAATAGCATTGGCTGCTGCATCACACATTGAGAGAGAACTGCAGATTACTCCTTGGAACTTGAGTAGCAAT
TTTGTTGCTTGTACAAGCCAGGGTAAGGAAAATATTGAGCGAATGGAAATTACTGGCGTTGGTGATCCTTCTGGTCGAGGCA
TGGGTTTCAGCTATGCTCGGGCACCTCCAAAGGCACCAGTGTCTAGTGCAATGGTGAAGAAGAAAGCAGCTGCTAACCGTG
GAGGTTCCACTGTTACTGGTACAGATGCTGATCTGCGTAGATTAAGCATGGAGGCTGCGCGAGAGGTTCTTCTTAAGTTCAA
TGTTCCTGAGGAAGTCATTACTAAACAAACCAGGTGGCATCGCATTGCTATGATACGTAAACTTTCAAGTGAGCAAGCTGCA
TCTGGGGTTAAGGTTGACCCGACAACTATCAGCAAATATGCTCGTGGCCAGCGAATGTCCTTTCTTCAGTTACAGCAGCAGA
CTAGAGAAAAATGCCAGGAGATTTGGGATCGACAAGTTCAGAGTCTGTCAGCTGTAAATGCTGATGAGAACGAGAGTGATT
CGGAAGGTAATAGTGATCTGGATTCTTTTGCTGGAGACCTGGAAAATTTACTTGATGCTGAGGAGTTTGAAGAGGGAGAAG
AAGGTACAAATGACTTAAAACGTGACAAGGGAGACGGTGTTAAGGGTCTTAAAATGAGAAGACGCTCAACTTTGGCTCAG
GCAGAGGAGGAAATAGAAGATGAAGCCGCTGAGGCTGCTGAGTTATGCAGGTTGCTGATGGATGATGATGAAGCTGATAGG
AAGAAAAAGAAGAAAACTAAAGTAACTGGGGAAGAAACAAGATTGGTATCAAAGATGCAATCAAAATTTGCCTTCGACAAT
GCTGAACAAGTTAAACAAATAACAAATAGTTTACAATTAGATGGAAATATTCCCTTGAAAGAGGACACGATTACAGATCTTA

GGGAGGAGGAAAATTTTGGTGCTAAAAAAAGCAAATCACTGAAGGTCAATAAAGCCAAGAAGAATGATATTGCACCTATTT
CTCTTCCCAATAAAAAAATCAAATTGAATATGGGAGAAGGAATTAAGAACCAGGTGTTTAAGGAGAAAAAACCATCAAGGG
AAACTTTTGTTTGTGGAGCATGTGGTCAGCCTGGGCATATGCGAACAAACAAGAACTGTCCAAAATATGGTGAAGATCTTGA
AACTCAACTTGAATCTGCAGATATGGAAAAATCATCTGGGAAACCCATATCTGTGGACCCCTCCAGTCACTCCCAGCCTAAA
ACCGCCTCCAAAAAGTCAAGTTCGAAAAGCAACTCAAAGATTACTCCTGTTGACAATTCGGCAAAAATTCCACTGAAATTC
AAATGTGGCTCCACAGAGAAATCTTCTGATAAACCTGTAACAGAGACCCTGCAGAACTCTGACAAGCCAGTCACTTCGGAT
TCAGAAACTGCAAAGTCTGCTAAGGTTAATAAGATAATTATTCCCAAAAAAGTGAAACCAGATGATACACAGGCTGAATCTC
GTAAGCATGCTGTTGTTATAAGGCCTCCTACTGAATCAAGTAGAGGGCTTCCTGCTGATTCAGGTAGAGGGCCTCCTACTGAT
GCAGGCAGAGGTCAGGTTGATTATCAGAAGTTACCAATTAAAATACGACCACCAACAGAGGAGCAAAGTCACAAAAAGATT
GTTATAAGACGTACGAAGGAGGTTATTGATTTAGAACTGGATAGTCCTGGTGGAAACACTGGACTTCAACACAGAAAGACA
AAAAGAATTGTTGAATTGTCAAATTTTGAGAAACATAAACAGGAGACCGTGTATGGAACTGGAGCTTTTCCAAAATGGAAC
ACTAAAGAGGACAGAAGATGGTGGGAAGAGCAAGAGAAACGAAGAAATGATGCAAGACTTAGAGAAGAAGACAGAGCAA
GGAGGCATCACAAAGAAGAAATGAGGATGCTCAAAGAGCAAGAAAGGTTAGATGAGATAAAAAGATTTGAAGAAGATATC
AGAAGAGAGAGGGAGGAAGAAGAACGGCAAAAGGCTAAGAAGAAAAAGAAGAAGAAAAAGCCTGATTTAAGAGATGAG
TATTTAGATGATCCCAGAGCAAGAAGACATGATAAGAGAATGCCTGAAAGAGACAGGAGTGGAAAAAGGAGATCTGTTGCT
GAGTTAGGAAAGCTTAGTGCAGATTATATGCCGCCAACAAAACGCCGAAGAGGGGGAGGGGGAGAGGTTGGTTTGGCAAA
TATCTTGGAGGGCATTGTGGAAACGATGGTTAAAGATAGGTACGAGCTATCGTATCTTTTCGTGAAACCAGTGTCGAAGAAA
GAGGCTCCTGACTACCTGGACATTATAGATACGCCTATGGATCTTTCCAGAATCAGGGAGAGAGTACGGAATATGGAGTACA
AGAGCCGAGAAGATTTCAGGCATGATGTTTGGCAGATTACTTTTAATGCACACAAATACAACGACGGGAGAAATCCTGGAAT
TCCTCCCCTTGCAGATATGCTTTTGGAATATTGTGATTATTTGTTGAATGAGAATGATGATAGCCTCACTTCAGCAGAAGCTGG
CATTGAAACTAGAGATTCCTAA

>VrTAF1-X4

ATGACATTGCCAATCAATTTCACATATGCTAGCTTTGTTCTTTCAGAGGAGCAGGAAGAGACTTTGGTAGAGGCATCTAAAG
AAGAGAGTGCTCTTGAACGTGAATTACATGTTGACTCACTGCAAAGTGAAGAATTGGATGCTGATGTACAAAAACCTGAGG
AGGAAGGGACTGAAGTCCAGAAAAGGTCTATGGCTATGCCGTTGCCTATTTTATGTGTGGAAGATGGTGTGGCAATTTTACG
CTTCTCTGAAATCTTTGGCATTCATGAACCTCTCAGAAAGGGAGAAAAGAGAGAACACAGGCAACCTATTACTAGAGATAGA
TACAAGTCTTTGGATTTTACTGATGATTTTGTAGAAGAGGATGAAGAGGAATTCCTCAAGGGTTCCTCCCAGAGTCTCTCAC
AGACTAAACAGGTTTCTGTAGTTCATAATGATGTATCAGAAAGCAACGATGTTGACTTGGAGTTTCCAAAATTTGGGTTTCTT
CATACTGAGCCCTCAGTGGCCAGGAAAGATGATCATCAATCGAAGGACTCGTGTCATAGTGCTGAACCAATGAAAGGGGATT
TTGAAGAAGACCTTTCCTGGAAAGATCATCCTTTCATATGGACCAACTTTTATCCTCTTGATCAGCAAGACTGGGAAGATGA
AATCATTTGGGGCAATTCTCCTGTTCCAAGTAATAATAATATTGAAAGCTGTGAAGTTTCTGGACCTGAGTTGGGAGTTTCTG
GTGGCAGTGAAATAGAAATTGAAAGTGGGATCCAGAATATTCAGCTGGAGCATCACAAGATACTGGAAGATAAAGATCATAA
TGTCTTGCTCAGCTCTCCTGTCTCGTTGGAGGCCTTTGGCTCAAGGGATTCTTCTGAAGCTAAAACCAATCTAATATCTAGAA
GTCTTTTTCATCCCCAACTTTTAAGGTTAGAATCCAGATCTGAAGTGGATAGTTCTAGTCTTCCAGATGGAAAAGAGGGGGA
GATATCTAAACATAATCAAAGTGGTCAAATTACACGTTTTAGCAAGGCTATATCACAAAATAGAGACATGGTGGAGGGTTCCT
GGTTAGACGAGATAATATGGGAGGAGCTTGATCAGCCTATGGTGAAACCAAAGCTTATCTTTGATCTTCAGGATGATCAAATG
CACTTCGAAGTTTTGGATAGTAAGGATGGTGCACATCTTCGTCTTCATGCTGGGGCTATAATTTTAACTCGTTCTTCAAAATCA
AGCAGTGGGGACTCATCTGAAGTACCAGGACATGGAAGTCAATATGGATGGCGATATGTGTCTAATGACAAACATTATTCAA
ATCGTAAAACTTCTCAACAATTGAAATCAAATTCCAAAAAACGCTCGGCACATGGTGTCAAAGTTTTCCACTCTCAACCTGC
GTTGAAGCTGCAGACAATGAAATTGAAGTTGAGCAATAAAGATATTGCAAATTTTCACCGGCCAAAAGCATTATGGTATCCC
CATGACAATGAGGTTGCTGTCAAAGAACAAGGGAAATTGCCAACACAAGGACCCATGAAAATTATTATAAAGAGTTTGGGT
GGCAAGGGAAGTAAATTGCATGTGGATACTGAAGAAACTCTCTCAACTGTTAAAGCAAAAGCTTCCAAAAAGCTAGATTTT
AAGGCATCAGAAACAGTGAAAATATTTTATTTAGGGAGGGAGCTTGAAGATCAGAAGTCACTTGCCGAACAAAATGTTCAA
CCAAACTCCTTGTTACATCTTGTTCGTTCAAAGATACATTTGTGGCCAAAAGCACAGAGGGTTCCTGGGGAGAACAAGTCCT
TGCGTCCTCCTGGGGCATTCAAGAAAAAATCTGATCTGTCTGTAAAAGACGGACATGTTTTTCTGATGGAGTATTGTGAAGA
AAGACCTTTACTTTTGAGCAATGTTGGAATGGGTGCAAGACTTTGTACATACTATCAAAAATGCTCACCAGATGACCAATCTG
GCTCTTTATTACGTAACACAGATAGTAGCTTGGGGCACGTTATTTCTCTGGATCCTGCGGATAAATCTCCGTTCCTTGGAGATT
TGAAACCTGGTTGCTCTCAGTCATCACTAGAGACAAATATGTATAGAGCACCCGTATTTCCTCATAAAGTTCCACTAACTGAC
TACCTGCTGGTTCGCTCACCAAAGGGAAAGCTATCATTAAGGCGCATTGATAAAATTAATGTTGTTGGACAGCAGGAGCCAC
TCATGGAGGTATTTTCACCAGGAAGTAAAAATCTTCAGACTTACATGATGAACAGGCTGTTGGTACACATGTGCCGTGAATTC
CAAGCAGCAGAGAAGCGGCACTTGCCTCCTCATATCCGTGTTGATGAATTTCTCTCACAGTTTCCTTACCAATCGGAAGCATC
ATTTCGTAAGAAAATCAAGGAATATGCAAATTTACAGAGGGGAGCAAATGGACAGTCGATTTTGGTTAAAAAGAGAAATTTC
CGCATGTGGTCTGAGGATGAATTGAGAAAAATGGTTCTGCCGGAGCTTGTTTGTGCCTATGAAAGCATGCAAGCAGGCCTCT
ACCGGCTAAAACATTTAGGAATAACTGAAACACACCCTACAAATATTTCATCTGCAATGAGTCGGCTCCCTGATGAAGCAATA
GCATTGGCTGCTGCATCACACATTGAGAGAGAACTGCAGATTACTCCTTGGAACTTGAGTAGCAATTTTGTTGCTTGTACAA
GCCAGGGTAAGGAAAATATTGAGCGAATGGAAATTACTGGCGTTGGTGATCCTTCTGGTCGAGGCATGGGTTTCAGCTATGC
TCGGGCACCTCCAAAGGCACCAGTGTCTAGTGCAATGGTGAAGAAGAAAGCAGCTGCTAACCGTGGAGGTTCCACTGTTAC
TGGTACAGATGCTGATCTGCGTAGATTAAGCATGGAGGCTGCGCGAGAGGTTCTTCTTAAGTTCAATGTTCCTGAGGAAGTC
ATTACTAAACAAACCAGGTGGCATCGCATTGCTATGATACGTAAACTTTCAAGTGAGCAAGCTGCATCTGGGGTTAAGGTTG
ACCCGACAACTATCAGCAAATATGCTCGTGGCCAGCGAATGTCCTTTCTTCAGTTACAGCAGCAGACTAGAGAAAAATGCCA
GGAGATTTGGGATCGACAAGTTCAGAGTCTGTCAGCTGTAAATGCTGATGAGAACGAGAGTGATTCGGAAGGTAATAGTGAT
CTGGATTCTTTTGCTGGAGACCTGGAAAATTTACTTGATGCTGAGGAGTTTGAAGAGGGAGAAGAAGGTACAAATGACTTA
AAACGTGACAAGGGAGACGGTGTTAAGGGTCTTAAAATGAGAAGACGCTCAACTTTGGCTCAGGCAGAGGAGGAAATAGA
AGATGAAGCCGCTGAGGCTGCTGAGTTATGCAGGTTGCTGATGGATGATGATGAAGCTGATAGGAAGAAAAAGAAGAAAAC
TAAAGTAACTGGGGAAGAAACAAGATTGGTATCAAAGATGCAATCAAAATTTGCCTTCGACAATGCTGAACAAGTTAAACA
AATAACAAATAGTTTACAATTAGATGGAAATATTCCCTTGAAAGAGGACACGATTACAGATCTTAGGGAGGAGGAAAATTTT
GGTGCTAAAAAAAGCAAATCACTGAAGGTCAATAAAGCCAAGAAGAATGATATTGCACCTATTTCTCTTCCCAATAAAAAAA
TCAAATTGAATATGGGAGAAGGAATTAAGAACCAGGTGTTTAAGGAGAAAAAACCATCAAGGGAAACTTTTGTTTGTGGAG
CATGTGGTCAGCCTGGGCATATGCGAACAAACAAGAACTGTCCAAAATATGGTGAAGATCTTGAAACTCAACTTGAATCTGC
AGATATGGAAAAATCATCTGGGAAACCCATATCTGTGGACCCCTCCAGTCACTCCCAGCCTAAAACCGCCTCCAAAAAGTCA
AGTTCGAAAAGCAACTCAAAGATTACTCCTGTTGACAATTCGGCAAAAATTCCACTGAAATTCAAATGTGGCTCCACAGAG
AAATCTTCTGATAAACCTGTAACAGAGACCCTGCAGAACTCTGACAAGCCAGTCACTTCGGATTCAGAAACTGCAAAGTCT
GCTAAGGTTAATAAGATAATTATTCCCAAAAAAGTGAAACCAGATGATACACAGGCTGAATCTCGTAAGCATGCTGTTGTTAT
AAGGCCTCCTACTGAATCAAGTAGAGGGCTTCCTGCTGATTCAGGTAGAGGGCCTCCTACTGATGCAGGCAGAGGTCAGGT
TGATTATCAGAAGTTACCAATTAAAATACGACCACCAACAGAGGAGCAAAGTCACAAAAAGATTGTTATAAGACGTACGAA
GGAGGTTATTGATTTAGAACTGGATAGTCCTGGTGGAAACACTGGACTTCAACACAGAAAGACAAAAAGAATTGTTGAATT
GTCAAATTTTGAGAAACATAAACAGGAGACCGTGTATGGAACTGGAGCTTTTCCAAAATGGAACACTAAAGAGGACAGAAG
ATGGTGGGAAGAGCAAGAGAAACGAAGAAATGATGCAAGACTTAGAGAAGAAGACAGAGCAAGGAGGCATCACAAAGAA
GAAATGAGGATGCTCAAAGAGCAAGAAAGGTTAGATGAGATAAAAAGATTTGAAGAAGATATCAGAAGAGAGAGGGAGGA

AGAAGAACGGCAAAAGGCTAAGAAGAAAAAGAAGAAGAAAAAGCCTGATTTAAGAGATGAGTATTTAGATGATCCCAGAG
CAAGAAGACATGATAAGAGAATGCCTGAAAGAGACAGGAGTGGAAAAAGGAGATCTGTTGCTGAGTTAGGAAAGCTTAGT
GCAGATTATATGCCGCCAACAAAACGCCGAAGAGGGGGAGGGGGAGAGGTTGGTTTGGCAAATATCTTGGAGGGCATTGTG
GAAACGATGGTTAAAGATAGGTACGAGCTATCGTATCTTTTCGTGAAACCAGTGTCGAAGAAAGAGGCTCCTGACTACCTGG
ACATTATAGATACGCCTATGGATCTTTCCAGAATCAGGGAGAGAGTACGGAATATGGAGTACAAGAGCCGAGAAGATTTCAG
GCATGATGTTTGGCAGATTACTTTTAATGCACACAAATACAACGACGGGAGAAATCCTGGAATTCCTCCCCTTGCAGATATGC
TTTTGGAATATTGTGATTATTTGTTGAATGAGAATGATGATAGCCTCACTTCAGCAGAAGCTGGCATTGAAACTAGAGATTCCT
AA

>VrTAF2-X1

ATGGCGAAACCTCGCAAGACCAAGAACAACGAAGACCCGAAGCCCGAAAACTCTGGTGCCCTAGTTCACCACCAGAAGCT
ATGTCTCTCCATTGACATCGACAAACGCCTAGTCCACGGGTACACTGAATTGGAAATTGCGGTGCCGGAGATTGGGATCGTA
GGGTTGCACGCGGAGAATTTAGGGATTGAGAGTGTTTGGGTCGATGGTGAGCCCACGGAGTTCGAGTATTACCCGCATCAGC
AGCAGCAGGTGGAAGATGATAAGAGGTTTAGTTCTGTGTGTTCTCCCAGCTCTGCTGCCGATGCTGCTGTCTCAGTGTACAT
GTCCTCTCTGGAGAAGGAGCTGGTGCCCAATTTACTTATTAACTGCTGTAAACCTTCCAAGACTGAAAGTGAACAACAGCA
GGAGCAAACAGTTCCCGAGAACGGGTTTCACTCCACGGCCGAGCCCAAACAGAATGTGAGAATTGTTCGTATTGACTATTG
GATAGAAAAGGCAGAGACAGGAATCCACTTCAGAAATAATCTTCTTCATACTGATAATCAGATAAGAAGAGCTAGATGTTGG
TTCCCCTGTATAGATGACAATTCACAACGATGCTGCTATGACCTGGAGTTCACTGTGGCACACAATCTTGTGGCTGTTAGTAC
GGGATTCTTGCTTTATCAGGTCTTAAGCAAGGACAATCCTCCTAGGAAAACATATGTCTATAAATTGGATGTTCCAGTAGCTG
CAAGGTGGATATCTTTGGCTGTTGCCCCATTTGAAATTTTTCCTGATCACCAATTTAGTCTCATATCACACATGTGCTTGATGC
CTAATCTGTCAAAGATGCGGAATACAGTGGAATTTTTCCACAGTGCCTTCAGCTGCTATAAGGATTATCTTGCTGTAGACTTC
CCATTTGACTCATACACGCAAGTTTTCATAGAGCCAGAGATGGCTGTGTCTTCACTGAGTTTAGGAGCCTCTATGAGTATATTT
AGTTCACAAGTTTTGTTTGATGAGAAGGTTATTGATCAGACTATTGACACAAGGGCAAAACTTGCATATGCTCTTGCAAGAC
AGTGGTTTGGGGTGTATATCACTCCCGAGACACCAAATGATGAGTGGCTCTTGGATGGACTTGCTGGCTTCTTGACAGATTTT
TATATCAAGAAACATTTGGGAAATAATGAGGCACGATACAGGAGATACAAGGCAAATTGTGCTGTTTGCAAAGTTGATAATG
GTGGAGCGACAGCTTTGAGCTGTTCAGCTTCCTGCAAGGATTTATATGGAACTCAGTGTATTGGTTTGTATGGAAAAATAAGA
TCGTGGAAGTCTGTGGCTGTTCTTCAGATGTTGGAAAAGCAAATGGGTCCTGAATCTTTCCGTAGAATTTTACAAACGATAG
TTTCTCGGGCTCAAGATAAAACACGTTCCATGAAGACTCTAAGTACTAAGGAGTTTCGACATTTTGCCAATAAGGTTGGGAA
TCTTGAACGTCCATTTCTTAAGGATTTCTTTCCACGGTGGGTTGGTTCTTGTGGGTGTCCTGTTTTAAGGATGGGGTTTTCCTA
TAACAAAAGGAAGAATATGGTTGAATTGGCTGTGTTGCGAGGATGTACAGCATTGCAGACTTCAACTACATCCACTCTTGATA
TTAATCCAGAGACAGAAAATAGAGATGGAGATACTGGATGGCCTGGTATGATGAGCATCAGGGTATATGAACTTGATGGTATG
TATGACCATCCTATTCTGCCAATGGCTGGAGAAGCATGGCAACTGTTGGAAATACAATGCCACTCAAAGCTTGCTGCTAGAC
GCTTTCAGAAGCCCAAGAAAGGGTTAAAACATGATGGATCTGATGATAATGGTGACGTGCCTTCCATGGATGTGCGCTCAAA
TACTGAGTCCCCTTTATTATGGATCAGAGCAGATCCTGATATGGAGTACCTTGCCGAGGTTCACTTTAACCAACCTGTTCAGA
TGTGGATCAATCAATTAGAGAAGGACAAAGATGTTATTGCTCAGGCACAAGCAATTGCAGCTCTTGAGGCATCACCACAACT
ATCATTTTCTATTGTAAATGCCCTAAACAATTTTCTCGGTGACTCCAAGGCCTTTTGGAGAGTACGGATTGAAGCGGCATTTG
CATTGGCAAATTCAGCATCTGAGGAAACTGATTTTTCTGGTCTACTCCATTTGGTGAAATTTTATAAGAGTAGAAGGTTTGAT
CCTGACATTGGACTCCCAAAGCCGAATGATTTTCATGATTTTGCTGAGTATTTTGTTCTTGAGGCTATTCCACATGCTGTTGCC
ATGGTTAGAGCCGCTGACAAGAAAAGCCCAAGAGAGGCTATCGAGTTTGTTCTACAATTATTGAAGTACAATGACAACAATG
GGAATCCCTACTCAGATGTCTTTTGGCTTTCTGCATTAGTCCAATCGGTTGGTGAGCTTGAGTTTGGGCAACAGAGTATTCTG
TTGTTGTCGTCACTTCTCAAACGCATTGACCGGCTTCTACAATTTGATAGTCTCATGCCAAGATATAATGGAATCTTGACTATC
AGTTGTATCCGAACATTGACCCAGATTGCTTTAAAGCTTTCGGGTTTCATTCCTCTTGATCGAGTTTTTGAACTTGTAAAGCCT
TTTCGTGACTTGAAGACACTGTGGCAAGTTCGAATTGAAGCAAGCAGAGCACTCCTTGACCTTGAATTCCACTGCAAGGGC
ATGGATTCAGCATTGCTTTTATTTATCAAGTACTTAGAGGAGGAGCATTCCTTAAGAGGACAGTTAAAGCTAGCCACCCATGT
TATGAGGCTATGTCAGATGAGGGATGGATTGAATTCAGATGAGGAAATTACCAGCCAAACTCTTGTCTCTATGCTTAATTTACT
GGAAGGGCGTACTGCTTTTAATAATGTCTTTCTCCGGCATTATTTGTTCTGCATACTACAAATACTTGCAAGAAGACCACCAA
CTCTTCATGGGATTCCCAGGGAAAATAGAACGTTGCATATGAGTCTTACCGAAGCTTGTAACTATCAGAAGAACATGTTTGTT
CTTGATTCGGATAGCAAACCTTTGGATCTGCCAAGCTCTACTCAAAACCCTACACCAAATCTTTGCCTAGATGATTTGAGGGA
TGCACTTAACGAAGCTTCTAAGGACCCACCTGATGAAGCTCCGGTACAGGTGCACATTGAAGCTCTGAATGAAGCACCCCT
AGAGAAAGCCGAAGAAGTATATACTGAATTTCCACAGGAAGCGCCAATGGAAGCTCCAAATGAAGTTTCCAAGGAAGCTGA
TACTGTATCGAATAGCCATGAAAGAAAGAGGCTCATTAAAATCAAGGTCAAACAATCTTCTGCCACCAGTAGGGCTGATACT
GATAATCAAGTGGTTGAACGTTCTTTAGGTGGTCGTAACGAAATGGACCATGGAGCTAGCAGTTCAGTTTCTGTAGATGCAC
CCCAGAGGAATTTTGCCGAGACTCTTAGCATGAGTAATCACAATATAGACGAAGTTAATTCTTGGCATGATCGTGGTTCGCGC
ATGACTGCTAGCATTGGTAGTGCTAAATTTTTGAGTGACGGTGATGAATTAGTCAAAGAACTTCAGTGCACTGCTGATTCAA
GTATAGTTTATTCACAACCTCAGCCAGAAGATCCGTCATCATCCAGTATTATACAGGATAACAATGTAGATGCTGATGCACGAC
GATATGCCAGCCTTCAAACTCTTTCAGTTGCAAGATTTGATCCTGAGGGAGAATCATTAGGTAAAGAAATTTCTGCTCGTGGC
AAGGAAAAACACAAAAGCAAGGAAAAGAAACGAAAACGGGAAAGTAATAAAGGACATCACGATGATCCAGAATATTTGGA
GCGAAAGCGACTAAAGAAGGAGAAAAAACGACGGGAAAAAGAACTGGCAAAACTTCAGAGTGATGAAGCGAAGAGATCC
TCTGTAGACTTGTCAAGTAAGAAAGAGGAACCTGTAGTGGACGTTTCCAGACAGATTAAATCTGTTGAGCCAGGCGGCGGA
CATAGTTCTAAATTAGAAACCAAAAAGATTGATAGCAAACCGGATCCATCCGAAGGCACGTCTGGTGCACCAAAAATTCGAA
TTAAAATTAAAAACCGAATGCTCAACAAGTCATAG

>VrTAF2-X2

ATGGCGAAACCTCGCAAGACCAAGAACAACGAAGACCCGAAGCCCGAAAACTCTGGTGCCCTAGTTCACCACCAGAAGCT
ATGTCTCTCCATTGACATCGACAAACGCCTAGTCCACGGGTACACTGAATTGGAAATTGCGGTGCCGGAGATTGGGATCGTA
GGGTTGCACGCGGAGAATTTAGGGATTGAGAGTGTTTGGGTCGATGGTGAGCCCACGGAGTTCGAGTATTACCCGCATCAGC
AGCAGCAGGTGGAAGATGATAAGAGGTTTAGTTCTGTGTGTTCTCCCAGCTCTGCTGCCGATGCTGCTGTCTCAGTGTACAT
GTCCTCTCTGGAGAAGGAGCTGGTGCCCAATTTACTTATTAACTGCTGTAAACCTTCCAAGACTGAAAGTGAACAACAGCA
GGAGCAAACAGTTCCCGAGAACGGGTTTCACTCCACGGCCGAGCCCAAACAGAATGTGAGAATTGTTCGTATTGACTATTG
GATAGAAAAGGCAGAGACAGGAATCCACTTCAGAAATAATCTTCTTCATACTGATAATCAGATAAGAAGAGCTAGATGTTGG
TTCCCCTGTATAGATGACAATTCACAACGATGCTGCTATGACCTGGAGTTCACTGTGGCACACAATCTTGTGGCTGTTAGTAC
GGGATTCTTGCTTTATCAGGTCTTAAGCAAGGACAATCCTCCTAGGAAAACATATGTCTATAAATTGGATGTTCCAGTAGCTG
CAAGGTGGATATCTTTGGCTGTTGCCCCATTTGAAATTTTTCCTGATCACCAATTTAGTCTCATATCACACATGTGCTTGATGC
CTAATCTGTCAAAGATGCGGAATACAGTGGAATTTTTCCACAGTGCCTTCAGCTGCTATAAGGATTATCTTGCTGTAGACTTC
CCATTTGACTCATACACGCAAGTTTTCATAGAGCCAGAGATGGCTGTGTCTTCACTGAGTTTAGGAGCCTCTATGAGTATATTT
AGTTCACAAGTTTTGTTTGATGAGAAGGTTATTGATCAGACTATTGACACAAGGGCAAAACTTGCATATGCTCTTGCAAGAC
AGTGGTTTGGGGTGTATATCACTCCCGAGACACCAAATGATGAGTGGCTCTTGGATGGACTTGCTGGCTTCTTGACAGATTTT
TATATCAAGAAACATTTGGGAAATAATGAGGCACGATACAGGAGATACAAGGCAAATTGTGCTGTTTGCAAAGTTGATAATG
GTGGAGCGACAGCTTTGAGCTGTTCAGCTTCCTGCAAGGATTTATATGGAACTCAGTGTATTGGTTTGTATGGAAAAATAAGA

TCGTGGAAGTCTGTGGCTGTTCTTCAGATGTTGGAAAAGCAAATGGGTCCTGAATCTTTCCGTAGAATTTTACAAACGATAG
TTTCTCGGGCTCAAGATAAAACACGTTCCATGAAGACTCTAAGTACTAAGGAGTTTCGACATTTTGCCAATAAGGTTGGGAA
TCTTGAACGTCCATTTCTTAAGGATTTCTTTCCACGGTGGGTTGGTTCTTGTGGGTGTCCTGTTTTAAGGATGGGGTTTTCCTA
TAACAAAAGGAAGAATATGGTTGAATTGGCTGTGTTGCGAGGATGTACAGCATTGCAGACTTCAACTACATCCACTCTTGATA
TTAATCCAGAGACAGAAAATAGAGATGGAGATACTGGATGGCCTGGTATGATGAGCATCAGGGTATATGAACTTGATGGTATG
TATGACCATCCTATTCTGCCAATGGCTGGAGAAGCATGGCAACTGTTGGAAATACAATGCCACTCAAAGCTTGCTGCTAGAC
GCTTTCAGAAGCCCAAGAAAGGGTTAAAACATGATGGATCTGATGATAATGGTGACGTGCCTTCCATGGATGTGCGCTCAAA
TACTGAGTCCCCTTTATTATGGATCAGAGCAGATCCTGATATGGAGTACCTTGCCGAGGTTCACTTTAACCAACCTGTTCAGA
TGTGGATCAATCAATTAGAGAAGGACAAAGATGTTATTGCTCAGGCACAAGCAATTGCAGCTCTTGAGGCATCACCACAACT
ATCATTTTCTATTGTAAATGCCCTAAACAATTTTCTCGGTGACTCCAAGGCCTTTTGGAGAGTACGGATTGAAGCGGCATTTG
CATTGGCAAATTCAGCATCTGAGGAAACTGATTTTTCTGGTCTACTCCATTTGGTGAAATTTTATAAGAGTAGAAGGTTTGAT
CCTGACATTGGACTCCCAAAGCCGAATGATTTTCATGATTTTGCTGAGTATTTTGTTCTTGAGGCTATTCCACATGCTGTTGCC
ATGGTTAGAGCCGCTGACAAGAAAAGCCCAAGAGAGGCTATCGAGTTTGTTCTACAATTATTGAAGAGTATTCTGTTGTTGT
CGTCACTTCTCAAACGCATTGACCGGCTTCTACAATTTGATAGTCTCATGCCAAGATATAATGGAATCTTGACTATCAGTTGTA
TCCGAACATTGACCCAGATTGCTTTAAAGCTTTCGGGTTTCATTCCTCTTGATCGAGTTTTTGAACTTGTAAAGCCTTTTCGT
GACTTGAAGACACTGTGGCAAGTTCGAATTGAAGCAAGCAGAGCACTCCTTGACCTTGAATTCCACTGCAAGGGCATGGAT
TCAGCATTGCTTTTATTTATCAAGTACTTAGAGGAGGAGCATTCCTTAAGAGGACAGTTAAAGCTAGCCACCCATGTTATGAG
GCTATGTCAGATGAGGGATGGATTGAATTCAGATGAGGAAATTACCAGCCAAACTCTTGTCTCTATGCTTAATTTACTGGAAG
GGCGTACTGCTTTTAATAATGTCTTTCTCCGGCATTATTTGTTCTGCATACTACAAATACTTGCAAGAAGACCACCAACTCTTC
ATGGGATTCCCAGGGAAAATAGAACGTTGCATATGAGTCTTACCGAAGCTTGTAACTATCAGAAGAACATGTTTGTTCTTGAT
TCGGATAGCAAACCTTTGGATCTGCCAAGCTCTACTCAAAACCCTACACCAAATCTTTGCCTAGATGATTTGAGGGATGCACT
TAACGAAGCTTCTAAGGACCCACCTGATGAAGCTCCGGTACAGGTGCACATTGAAGCTCTGAATGAAGCACCCCTAGAGAA
AGCCGAAGAAGTATATACTGAATTTCCACAGGAAGCGCCAATGGAAGCTCCAAATGAAGTTTCCAAGGAAGCTGATACTGTA
TCGAATAGCCATGAAAGAAAGAGGCTCATTAAAATCAAGGTCAAACAATCTTCTGCCACCAGTAGGGCTGATACTGATAATC
AAGTGGTTGAACGTTCTTTAGGTGGTCGTAACGAAATGGACCATGGAGCTAGCAGTTCAGTTTCTGTAGATGCACCCCAGAG
GAATTTTGCCGAGACTCTTAGCATGAGTAATCACAATATAGACGAAGTTAATTCTTGGCATGATCGTGGTTCGCGCATGACTG
CTAGCATTGGTAGTGCTAAATTTTTGAGTGACGGTGATGAATTAGTCAAAGAACTTCAGTGCACTGCTGATTCAAGTATAGTT
TATTCACAACCTCAGCCAGAAGATCCGTCATCATCCAGTATTATACAGGATAACAATGTAGATGCTGATGCACGACGATATGC
CAGCCTTCAAACTCTTTCAGTTGCAAGATTTGATCCTGAGGGAGAATCATTAGGTAAAGAAATTTCTGCTCGTGGCAAGGAA
AAACACAAAAGCAAGGAAAAGAAACGAAAACGGGAAAGTAATAAAGGACATCACGATGATCCAGAATATTTGGAGCGAAA
GCGACTAAAGAAGGAGAAAAAACGACGGGAAAAAGAACTGGCAAAACTTCAGAGTGATGAAGCGAAGAGATCCTCTGTA
GACTTGTCAAGTAAGAAAGAGGAACCTGTAGTGGACGTTTCCAGACAGATTAAATCTGTTGAGCCAGGCGGCGGACATAGT
TCTAAATTAGAAACCAAAAAGATTGATAGCAAACCGGATCCATCCGAAGGCACGTCTGGTGCACCAAAAATTCGAATTAAAA
TTAAAAACCGAATGCTCAACAAGTCATAG

>VrTAF2-X3

ATGGCGAAACCTCGCAAGACCAAGAACAACGAAGACCCGAAGCCCGAAAACTCTGGTGCCCTAGTTCACCACCAGAAGCT
ATGTCTCTCCATTGACATCGACAAACGCCTAGTCCACGGGTACACTGAATTGGAAATTGCGGTGCCGGAGATTGGGATCGTA
GGGTTGCACGCGGAGAATTTAGGGATTGAGAGTGTTTGGGTCGATGGTGAGCCCACGGAGTTCGAGTATTACCCGCATCAGC
AGCAGCAGGTGGAAGATGATAAGAGGTTTAGTTCTGTGTGTTCTCCCAGCTCTGCTGCCGATGCTGCTGTCTCAGTGTACAT
GTCCTCTCTGGAGAAGGAGCTGGTGCCCAATTTACTTATTAACTGCTGTAAACCTTCCAAGACTGAAAGTGAACAACAGCA
GGAGCAAACAGTTCCCGAGAACGGGTTTCACTCCACGGCCGAGCCCAAACAGAATGTGAGAATTGTTCGTATTGACTATTG
GATAGAAAAGGCAGAGACAGGAATCCACTTCAGAAATAATCTTCTTCATACTGATAATCAGATAAGAAGAGCTAGATGTTGG
TTCCCCTGTATAGATGACAATTCACAACGATGCTGCTATGACCTGGAGTTCACTGTGGCACACAATCTTGTGGCTGTTAGTAC
GGGATTCTTGCTTTATCAGGTCTTAAGCAAGGACAATCCTCCTAGGAAAACATATGTCTATAAATTGGATGTTCCAGTAGCTG
CAAGGTGGATATCTTTGGCTGTTGCCCCATTTGAAATTTTTCCTGATCACCAATTTAGTCTCATATCACACATGTGCTTGATGC
CTAATCTGTCAAAGATGCGGAATACAGTGGAATTTTTCCACAGTGCCTTCAGCTGCTATAAGGATTATCTTGCTGTAGACTTC
CCATTTGACTCATACACGCAAGTTTTCATAGAGCCAGAGATGGCTGTGTCTTCACTGAGTTTAGGAGCCTCTATGAGTATATTT
AGTTCACAAGTTTTGTTTGATGAGAAGGTTATTGATCAGACTATTGACACAAGGGCAAAACTTGCATATGCTCTTGCAAGAC
AGTGGTTTGGGGTGTATATCACTCCCGAGACACCAAATGATGAGTGGCTCTTGGATGGACTTGCTGGCTTCTTGACAGATTTT
TATATCAAGAAACATTTGGGAAATAATGAGGCACGATACAGGAGATACAAGGCAAATTGTGCTGTTTGCAAAGTTGATAATG
GTGGAGCGACAGCTTTGAGCTGTTCAGCTTCCTGCAAGGATTTATATGGAACTCAGTGTATTGGTTTGTATGGAAAAATAAGA
TCGTGGAAGTCTGTGGCTGTTCTTCAGATGTTGGAAAAGCAAATGGGTCCTGAATCTTTCCGTAGAATTTTACAAACGATAG
TTTCTCGGGCTCAAGATAAAACACGTTCCATGAAGACTCTAAGTACTAAGGAGTTTCGACATTTTGCCAATAAGGTTGGGAA
TCTTGAACGTCCATTTCTTAAGGATTTCTTTCCACGGTGGGTTGGTTCTTGTGGGTGTCCTGTTTTAAGGATGGGGTTTTCCTA
TAACAAAAGGAAGAATATGGTTGAATTGGCTGTGTTGCGAGGATGTACAGCATTGCAGACTTCAACTACATCCACTCTTGATA
TTAATCCAGAGACAGAAAATAGAGATGGAGATACTGGATGGCCTGGTATGATGAGCATCAGGGTATATGAACTTGATGGTATG
TATGACCATCCTATTCTGCCAATGGCTGGAGAAGCATGGCAACTGTTGGAAATACAATGCCACTCAAAGCTTGCTGCTAGAC
GCTTTCAGAAGCCCAAGAAAGGGTTAAAACATGATGGATCTGATGATAATGGTGACGTGCCTTCCATGGATGTGCGCTCAAA
TACTGAGTCCCCTTTATTATGGATCAGAGCAGATCCTGATATGGAGTACCTTGCCGAGGTTCACTTTAACCAACCTGTTCAGA
TGTGGATCAATCAATTAGAGAAGGACAAAGATGTTATTGCTCAGGCACAAGCAATTGCAGCTCTTGAGGCATCACCACAACT
ATCATTTTCTATTGTAAATGCCCTAAACAATTTTCTCGGTGACTCCAAGGCCTTTTGGAGAGTACGGATTGAAGCGGCATTTG
CATTGGCAAATTCAGCATCTGAGGAAACTGATTTTTCTGGTCTACTCCATTTGGTGAAATTTTATAAGAGTAGAAGGTTTGAT
CCTGACATTGGACTCCCAAAGCCGAATGATTTTCATGATTTTGCTGAGTATTTTGTTCTTGAGGCTATTCCACATGCTGTTGCC
ATGGTTAGAGCCGCTGACAAGAAAAGCCCAAGAGAGGCTATCGAGTTTGTTCTACAATTATTGAAGTACAATGACAACAATG
GGAATCCCTACTCAGATGTCTTTTGGCTTTCTGCATTAGTCCAATCGGTTGGTGAGCTTGAGTTTGGGCAACAGAGTATTCTG
TTGTTGTCGTCACTTCTCAAACGCATTGACCGGCTTCTACAATTTGATAGTCTCATGCCAAGATATAATGGAATCTTGACTATC
AGTTGTATCCGAACATTGACCCAGATTGCTTTAAAGCTTTCGGGTTTCATTCCTCTTGATCGAGTTTTTGAACTTGTAAAGCCT
TTTCGTGACTTGAAGACACTGTGGCAAGTTCGAATTGAAGCAAGCAGAGCACTCCTTGACCTTGAATTCCACTGCAAGGGC
ATGGATTCAGCATTGCTTTTATTTATCAAGTACTTAGAGGAGGAGCATTCCTTAAGAGGTTGTTCATCTTTTCCCTCGCGGAGG
ATCTATAGATGA

>VrTAF4b-X1

ATGGACCCATCTATCATGAAGCTTCTAGAAGATGACGAGGATGAAACAATGCATTCAGGGGTGGATGTGGAAGCCTTCCAGG
CTGCTCTAAATAGAGATATAGGAGGAGATGTGTCTGCTTCTCAGTTTCCTGGTTCAGATGCAGTGTTGTCTCAAGGAAGCAAT
AATACATCAAGCCAATCTTCATCACAGTGGCCTACTTCTAACCATGACAGCCAAAGTGATGGTCAAAATCAAGAACCTAAAA
CTGCACAAGAGCAACATCCATCTGAGATGGAGCCAAAGCAACATGGGTCTCTTGGCGAACACCTTCAGCATGTTGCTTCTC
AGGATGTAAATAATATTCATTTATCACAGAAACAATCTCAAGATGACAGTCACCAAACACCTGCTGTACAAGTTCCCCTTCAC
AATTCTCAAACTATCGGAATTCATAATTCTGGAAAAGATTCAGTCCTTAATAAAGAAGTAGTAAAGAGTCATAATCCCAGCAG

TGAATCTCAGTATGCAAAGTTGCAGCAGATGAGTAATCAACAGGCTACAGTCTCTGAGCAGCCAAGTAGCCAAGTAAATCG
CAGTAATAAACAAGTACCATTTGGCCTGTTGCTACCCATCTTGCTTCCTCAACTTGCCAAAGACAGAGCCATGCAACTTCAA
ACCTTGTTTACTAAATTGAAGAAAGACGAAATACCGAAAGACAGTTTTGTGCGGCTTATGAAAGGTATCGTAGGGGACCAGA
TGCTTAGATTAGCATTAGCAAAGGTGCAAATGCAACCTCAGGCAAGGCCCAATCAAGCATCTGCTGGGCAGCAGCTTCCTGT
AAGGATGCCAACTGTTAGTTCAGGTGCTAGACAATTGAACGATCCACATGCTTTAGCACAGATGCATCAAAGAAGTATGAAT
GTTGCTGTTGACCAATCTCGCATGAGTTCTTCAGCTGGCCAAACCATGGATAGCAATGCTAGAAAATCTCAGGAATTTGATGT
TAAAATAGAATCTCAAGGATTGCAACCAAACCAGTTGACGTCTTCCACTTCCAACTCAGTAGCTCAAGAAACAGAAAGAGC
ATCAGTTCACATACAAGGGCTCAATAAGCAGCAGCAACATCATCTACATTTTGCATCAGCATATGGAAACAGTGGTGGTAACT
ATAACCCTTATTCTGGGACAACAGGCAGTTCCACTTCGTCTATCAAACCGCAATCACATGATTCACACATGAGTCAAATTCCT
CATCAAAGTATTGGTTCAAATCACTTAGGTGGGTCAACACATGGTTTGAGTGTAATTGGTATGCCAAAACTTGAACAGCAGA
ATTCTTTCAATGATCCCAAGAGACTGCCAGGTGGATCTGTGTCCTCTGCTGTAAACAATGCAGCATCTCAACAAACTTCAAAT
GCTTGGCAACCATCAACAAACAAAGAACAAAATCTGGGCTTAATGTCATCTGTTTCTTATGTCAAGAAGGAACCTGGTGACA
TGTCTACTGAGCAGCAAAATAGGCATAATTTGTCTAAATTGCATGGGTATTCTTCTGTTAATTCTGCTCAGCTTGAACAGAGT
GGTGCCAGTCAAGGAGCTCTAAAAGACGAGTTTTCAAGAGGTCTTCCAGCATCCACAAGCATGCCACCTACAACATCTACT
GGCTTGCTACCACACAGTTCTGCTTCTGCTTCTGCTGTGACCCACCTAGATTCCAGTGTCTTGCTAAGCTCTCAGATTCCATC
AAATGCTTCTGGAATTGCTGCAAGGCCATCTCTTAAAAAGTCTGCTGCTACCCAAAAGAAACCACTTGAAGCACTTGGTTCA
TCACCTCCTCCTTCTAGTAAAAAACAAAAAACTTCTGGGGGATATGCTGAACAAAGCATTGAACAACTTAATGACGTCACTG
CTGTTAGTGGAGTTGACCTCAGGGAAGAGGAAGAGCAGTTATTTTCAGGGCCCAAAGAGGATAGTCGAGTGTCAGAAGCAT
CTCGAAAGGCTGTGCAAGAAGAAGAGGAAAGGCTGATCTTGCAGAAAGCTCCATTGCAAAAAAAATTAATTGACATCATGG
CCAAGTGTGGCTTGAAGGGTATGAGCAATGATGTGGAGAAATGCTTGTCACTGAGTGTGGAGGAAAGGATGCGCGGACTGA
TAAGTAACCTGATTAGATTATCAAAACAGAGGGTTGATTTTGAGAAAACGAGACATCGGACTGTTGTCACTTCAGATGTTCG
GCAGCAAATCATGACAATAAATAGGAAAGTAAGGGATGAGTGGGAGAAAAAACAGGCGGAAGCAGAGAAGCTTCGGAAA
CTAAATGATGTTGACAGCAATACTGGAGGTGATGGTGACAAGGATAAGGACGACGGCCGTGCTAAATCAACAAAGGTGAAC
AAGGAAGAGGATGACAAGATGAGGACAAATGCTGCAAATGTTGCTGCTCGTGCTGCTTATGGGGGAGATGACATGCTGTCG
AAGTGGCAACTTATGGCTGAGCAAGCCAAGCAGAAACGTGAAGGGGGAGTAGACGTGTCATCTAGTTCTCAACCAGCTAA
AGATGTGAACCGCAAATCTTCATCAACATCTGAAAGAAGTACAAAGGATAACCAAGAAGGGGAAAAAAGAGGTTCAACCC
CTTTTCTAGCAAGCTCAGTGGCCAGAAAACTTGGGAAGAGTCATGCCATGGCCCCTCAAACTAGGGTAGCTCGAAGCATCT
CTGTCAAGGATGTGATTGCAGTCCTGGAGAGGGAGCCCCAAATGTCTAAATCCCCACTCATACATCGCTTGTACGAGAAAAT
TCATTCTGAAGCCCCAGTTGAACAAGGTTAA

>VrTAF4b-X2

ATGGACCCATCTATCATGAAGCTTCTAGAAGATGACGAGGATGAAACAATGCATTCAGGGGTGGATGTGGAAGCCTTCCAGG
CTGCTCTAAATAGAGATATAGGAGGAGATGTGTCTGCTTCTCAGTTTCCTGGTTCAGATGCAGTGTTGTCTCAAGGAAGCAAT
AATACATCAAGCCAATCTTCATCACAGTGGCCTACTTCTAACCATGACAGCCAAAGTGATGGTCAAAATCAAGAACCTAAAA
CTGCACAAGAGCAACATCCATCTGAGATGGAGCCAAAGCAACATGGGTCTCTTGGCGAACACCTTCAGCATGTTGCTTCTC
AGGATGTAAATAATATTCATTTATCACAGAAACAATCTCAAGATGACAGTCACCAAACACCTGCTGTACAAGTTCCCCTTCAC
AATTCTCAAACTATCGGAATTCATAATTCTGGAAAAGATTCAGTCCTTAATAAAGAAGTAGTAAAGAGTCATAATCCCAGCAG
TGAATCTCAGTATGCAAAGTTGCAGCAGATGAGTAATCAACAGGCTACAGTCTCTGAGCAGCCAAGTAGCCAAGTAAATCG
CAGTAATAAACAAGTACCATTTGGCCTGTTGCTACCCATCTTGCTTCCTCAACTTGCCAAAGACAGAGCCATGCAACTTCAA
ACCTTGTTTACTAAATTGAAGAAAGACGAAATACCGAAAGACAGTTTTGTGCGGCTTATGAAAGGTATCGTAGGGGACCAGA
TGCTTAGATTAGCATTAGCAAAGGTGCAAATGCAACCTCAGGCAAGGCCCAATCAAGCATCTGCTGGGCAGCAGCTTCCTGT
AAGGATGCCAACTGTTAGTTCAGGTGCTAGACAATTGAACGATCCACATGCTTTAGCACAGATGCATCAAAGAAGTATGAAT
GTTGCTGTTGACCAATCTCGCATGAGTTCTTCAGCTGGCCAAACCATGGATAGCAATGCTAGAAAATCTCAGGAATTTGATGT
TAAAATAGAATCTCAAGGATTGCAACCAAACCAGTTGACGTCTTCCACTTCCAACTCAGTAGCTCAAGAAACAGAAAGAGC
ATCAGTTCACATACAAGGGCTCAATAAGCAGCAGCAACATCATCTACATTTTGCATCAGCATATGGAAACAGTGGTGGTAACT
ATAACCCTTATTCTGGGACAACAGGCAGTTCCACTTCGTCTATCAAACCGCAATCACATGATTCACACATGAGTCAAATTCCT
CATCAAAGTATTGGTTCAAATCACTTAGGTGGGTCAACACATGGTTTGAGTGTAATTGGTATGCCAAAACTTGAACAGCAGA
ATTCTTTCAATGATCCCAAGAGACTGCCAGGTGGATCTGTGTCCTCTGCTGTAAACAATGCAGCATCTCAACAAACTTCAAAT
GCTTGGCAACCATCAACAAACAAAGAACAAAATCTGGGCTTAATGTCATCTGTTTCTTATGTCAAGAAGGAACCTGGTGACA
TGTCTACTGAGCAGCAAAATAGGCATAATTTGTCTAAATTGCATGGGTATTCTTCTGTTAATTCTGCTCAGCTTGAACAGAGT
GGTGCCAGTCAAGGAGCTCTAAAAGACGAGTTTTCAAGAGGTCTTCCAGCATCCACAAGCATGCCACCTACAACATCTACT
GGCTTGCTACCACACAGTTCTGCTTCTGCTTCTGCTGTGACCCACCTAGATTCCAGTGTCTTGCTAAGCTCTCAGATTCCATC
AAATGCTTCTGGAATTGCTGCAAGGCCATCTCTTAAAAAGTCTGCTGCTACCCAAAAGAAACCACTTGAAGCACTTGGTTCA
TCACCTCCTCCTTCTAGTAAAAAACAAAAAACTTCTGGGGGATATGCTGAACAAAGCATTGAACAACTTAATGACGTCACTG
CTGTTAGTGGAGTTGACCTCAGGGAAGAGGAAGAGCAGTTATTTTCAGGGCCCAAAGAGGATAGTCGAGTGTCAGAAGCAT
CTCGAAAGGCTGTGCAAGAAGAAGAGGAAAGGCTGATCTTGCAGAAAGCTCCATTGCAAAAAAAATTAATTGACATCATGG
CCAAGTGTGGCTTGAAGGGTATGAGCAATGATGTGGAGAAATGCTTGTCACTGAGTGTGGAGGAAAGGATGCGCGGACTGA
TAAGTAACCTGATTAGATTATCAAAACAGAGGGTTGATTTTGAGAAAACGAGACATCGGACTGTTGTCACTTCAGATGTTCG
GCAGCAAATCATGACAATAAATAGGAAAGTAAGGGATGAGTGGGAGAAAAAACAGGCGGAAGCAGAGAAGCTTCGGAAA
CTAAATGATGTTGACAGCAATACTGGAGGTGATGGTGACAAGGATAAGGACGACGGCCGTGCTAAATCAACAAAGGTGAAC
AAGGAAGAGGATGACAAGATGAGGACAAATGCTGCAAATGTTGCTGCTCGTGCTGCTTATGGGGGAGATGACATGCTGTCG
AAGTGGCAACTTATGGCTGAGCAAGCCAAGCAGAAACGTGAAGGGGGAGTAGACGTGTCATCTAGTTCTCAACCAGCTAA
AGATGTGAACCGCAAATCTTCATCAACATCTGAAAGAAGTACAAAGGATAACCAAGAAGGGGAAAAAAGAGGTTCAACCC
CTTTTCTAGCAAGCTCAGTGGCCAGAAAACTTGGGAAGAGTCATGCCATGGCCCCTCAAACTAGGGTAGCTCGAAGCATCT
CTGTCAAGGATGTGATTGCAGTCCTGGAGAGGGAGCCCCAAATGTCTAAATCCCCACTCATACATCGCTTGTACGAGAAAAT
TCATTCTGAAGCCCCAGTTGAACAAGGTTAA

>VrTAF4b-X3

ATGGACCCATCTATCATGAAGCTTCTAGAAGATGACGAGGATGAAACAATGCATTCAGGGGTGGATGTGGAAGCCTTCCAGG
CTGCTCTAAATAGAGATATAGGAGGAGATGTGTCTGCTTCTCAGTTTCCTGGTTCAGATGCAGTGTTGTCTCAAGGAAGCAAT
AATACATCAAGCCAATCTTCATCACAGTGGCCTACTTCTAACCATGACAGCCAAAGTGATGGTCAAAATCAAGAACCTAAAA
CTGCACAAGAGCAACATCCATCTGAGATGGAGCCAAAGCAACATGGGTCTCTTGGCGAACACCTTCAGCATGTTGCTTCTC
AGGATGTAAATAATATTCATTTATCACAGAAACAATCTCAAGATGACAGTCACCAAACACCTGCTGTACAAGTTCCCCTTCAC
AATTCTCAAACTATCGGAATTCATAATTCTGGAAAAGATTCAGTCCTTAATAAAGAAGTAGTAAAGAGTCATAATCCCAGCAG
TGAATCTCAGTATGCAAAGTTGCAGCAGATGAGTAATCAACAGGCTACAGTCTCTGAGCAGCCAAGTAGCCAAGTAAATCG
CAGTAATAAACAAGTACCATTTGGCCTGTTGCTACCCATCTTGCTTCCTCAACTTGCCAAAGACAGAGCCATGCAACTTCAA
ACCTTGTTTACTAAATTGAAGAAAGACGAAATACCGAAAGACAGTTTTGTGCGGCTTATGAAAGGTATCGTAGGGGACCAGA
TGCTTAGATTAGCATTAGCAAAGGTGCAAATGCAACCTCAGGCAAGGCCCAATCAAGCATCTGCTGGGCAGCAGCTTCCTGT
AAGGATGCCAACTGTTAGTTCAGGTGCTAGACAATTGAACGATCCACATGCTTTAGCACAGATGCATCAAAGAAGTATGAAT

GTTGCTGTTGACCAATCTCGCATGAGTTCTTCAGCTGGCCAAACCATGGATAGCAATGCTAGAAAATCTCAGGAATTTGATGT
TAAAATAGAATCTCAAGGATTGCAACCAAACCAGTTGACGTCTTCCACTTCCAACTCAGTAGCTCAAGAAACAGAAAGAGC
ATCAGTTCACATACAAGGGCTCAATAAGCAGCAGCAACATCATCTACATTTTGCATCAGCATATGGAAACAGTGGTGGTAACT
ATAACCCTTATTCTGGGACAACAGGCAGTTCCACTTCGTCTATCAAACCGCAATCACATGATTCACACATGAGTCAAATTCCT
CATCAAAGTATTGGTTCAAATCACTTAGGTGGGTCAACACATGGTTTGAGTGTAATTGGTATGCCAAAACTTGAACAGCAGA
ATTCTTTCAATGATCCCAAGAGACTGCCAGGTGGATCTGTGTCCTCTGCTGTAAACAATGCAGCATCTCAACAAACTTCAAAT
GCTTGGCAACCATCAACAAACAAAGAACAAAATCTGGGCTTAATGTCATCTGTTTCTTATGTCAAGAAGGAACCTGGTGACA
TGTCTACTGAGCAGCAAAATAGGCATAATTTGTCTAAATTGCATGGGTATTCTTCTGTTAATTCTGCTCAGCTTGAACAGAGT
GGTGCCAGTCAAGGAGCTCTAAAAGACGAGTTTTCAAGAGGTCTTCCAGCATCCACAAGCATGCCACCTACAACATCTACT
GGCTTGCTACCACACAGTTCTGCTTCTGCTTCTGCTGTGACCCACCTAGATTCCAGTGTCTTGCTAAGCTCTCAGATTCCATC
AAATGCTTCTGGAATTGCTGCAAGGCCATCTCTTAAAAAGTCTGCTGCTACCCAAAAGAAACCACTTGAAGCACTTGGTTCA
TCACCTCCTCCTTCTAGTAAAAAACAAAAAACTTCTGGGGGATATGCTGAACAAAGCATTGAACAACTTAATGACGTCACTG
CTGTTAGTGGAGTTGACCTCAGGGAAGAGGAAGAGCAGTTATTTTCAGGGCCCAAAGAGGATAGTCGAGTGTCAGAAGCAT
CTCGAAAGGCTGTGCAAGAAGAAGAGGAAAGGCTGATCTTGCAGAAAGCTCCATTGCAAAAAAAAATTAATTGACATCATGG
CCAAGTGTGGCTTGAAGGGTATGAGCAATGATGTGGAGAAATGCTTGTCACTGAGTGTGGAGGAAAGGATGCGCGGACTGA
TAAGTAACCTGATTAGATTATCAAAACAGAGGGTTGATTTTGAGAAAACGAGACATCGGACTGTTGTCACTTCAGATGTTCG
GCAGCAAATCATGACAATAAATAGGAAAGTAAGGGATGAGTGGGAGAAAAAACAGGCGGAAGCAGAGAAGCTTCGGAAA
CTAAATGATGTTGACAGCAATACTGGAGGTGATGGTGACAAGGATAAGGACGACGGCCGTGCTAAATCAACAAAGGTGAAC
AAGGAAGAGGATGACAAGATGAGGACAAATGCTGCAAATGTTGCTGCTCGTGCTGCTTATGGGGGAGATGACATGCTGTCG
AAGTGGCAACTTATGGCTGAGCAAGCCAAGCAGAAACGTGAAGGGGGGAGTAGACGTGTCATCTAGTTCTCAACCAGCTAA
AGATGTGAACCGCAAATCTTCATCAACATCTGAAAGAAGTACAAAGGATAACCAAGAAGGGGAAAAAAGAGGTTCAACCC
CTTTTCTAGCAAGCTCAGTGGCCAGAAAACTTGGGAAGAGTCATGCCATGGCCCCTCAAACTAGGGTAGCTCGAAGCATCT
CTGTCAAGGATGTGATTGCAGTCCTGGAGAGGGAGCCCCAAATGTCTAAATCCCCACTCATACATCGCTTGTACGAGAAAAT
TCATTCTGAAGCCCCAGTTGAACAAGGTTAA

>VrTAF4b-X4

ATGGACCCATCTATCATGAAGCTTCTAGAAGATGACGAGGATGAAACAATGCATTCAGGGGTGGATGTGGAAGCCTTCCAGG
CTGCTCTAAATAGAGATATAGGAGGAGATGTGTCTGCTTCTCAGTTTCCTGGTTCAGATGCAGTGTTGTCTCAAGGAAGCAAT
AATACATCAAGCCAATCTTCATCACAGTGGCCTACTTCTAACCATGACAGCCAAAGTGATGGTCAAAATCAAGAACCTAAAA
CTGCACAAGAGCAACATCCATCTGAGATGGAGCCAAAGCAACATGGGTCTCTTGGCGAACACCTTCAGCATGTTGCTTCTC
AGGATGTAAATAATATTCATTTATCACAGAAACAATCTCAAGATGACAGTCACCAAACACCTGCTGTACAAGTTCCCCTTCAC
AATTCTCAAACTATCGGAATTCATAATTCTGGAAAAGATTCAGTCCTTAATAAAGAAGTAGTAAAGAGTCATAATCCCAGCAG
TGAATCTCAGTATGCAAAGTTGCAGCAGATGAGTAATCAACAGGCTACAGTCTCTGAGCAGCCAAGTAGCCAAGTAAATCG
CAGTAATAAACAAGTACCATTTGGCCTGTTGCTACCCATCTTGCTTCCTCAACTTGCCAAAGACAGAGCCATGCAACTTCAA
ACCTTGTTTACTAAATTGAAGAAAGACGAAATACCGAAAGACAGTTTTGTGCGGCTTATGAAAGGTATCGTAGGGGACCAGA
TGCTTAGATTAGCATTAGCAAAGGTGCAAATGCAACCTCAGGCAAGGCCCAATCAAGCATCTGCTGGGCAGCAGCTTCCTGT
AAGGATGCCAACTGTTAGTTCAGGTGCTAGACAATTGAACGATCCACATGCTTTAGCACAGATGCATCAAAGAAGTATGAAT
GTTGCTGTTGACCAATCTCGCATGAGTTCTTCAGCTGGCCAAACCATGGATAGCAATGCTAGAAAATCTCAGGAATTTGATGT
TAAAATAGAATCTCAAGGATTGCAACCAAACCAGTTGACGTCTTCCACTTCCAACTCAGTAGCTCAAGAAACAGAAAGAGC
ATCAGTTCACATACAAGGGCTCAATAAGCAGCAGCAACATCATCTACATTTTGCATCAGCATATGGAAACAGTGGTGGTAACT
ATAACCCTTATTCTGGGACAACAGGCAGTTCCACTTCGTCTATCAAACCGCAATCACATGATTCACACATGAGTCAAATTCCT
CATCAAAGTATTGGTTCAAATCACTTAGGTGGGTCAACACATGGTTTGAGTGTAATTGGTATGCCAAAACTTGAACAGCAGA
ATTCTTTCAATGATCCCAAGAGACTGCCAGGTGGATCTGTGTCCTCTGCTGTAAACAATGCAGCATCTCAACAAACTTCAAAT
GCTTGGCAACCATCAACAAACAAAGAACAAAATCTGGGCTTAATGTCATCTGTTTCTTATGTCAAGAAGGAACCTGGTGACA
TGTCTACTGAGCAGCAAAATAGGCATAATTTGTCTAAATTGCATGGGTATTCTTCTGTTAATTCTGCTCAGCTTGAACAGAGT
GGTGCCAGTCAAGGAGCTCTAAAAGACGAGTTTTCAAGAGGTCTTCCAGCATCCACAAGCATGCCACCTACAACATCTACT
GGCTTGCTACCACACAGTTCTGCTTCTGCTTCTGCTGTGACCCACCTAGATTCCAGTGTCTTGCTAAGCTCTCAGATTCCATC
AAATGCTTCTGGAATTGCTGCAAGGCCATCTCTTAAAAAGTCTGCTGCTACCCAAAAGAAACCACTTGAAGCACTTGGTTCA
TCACCTCCTCCTTCTAGTAAAAAACAAAAAACTTCTGGGGGATATGCTGAACAAAGCATTGAACAACTTAATGACGTCACTG
CTGTTAGTGGAGTTGACCTCAGGGAAGAGGAAGAGCAGTTATTTTCAGGGCCCAAAGAGGATAGTCGAGTGTCAGAAGCAT
CTCGAAAGGCTGTGCAAGAAGAAGAGGAAAGGCTGATCTTGCAGAAAGCTCCATTGCAAAAAAAAATTAATTGACATCATGG
CCAAGTGTGGCTTGAAGGGTATGAGCAATGATGTGGAGAAATGCTTGTCACTGAGTGTGGAGGAAAGGATGCGCGGACTGA
TAAGTAACCTGATTAGATTATCAAAACAGAGGGTTGATTTTGAGAAAACGAGACATCGGACTGTTGTCACTTCAGATGTTCG
GCAGCAAATCATGACAATAAATAGGAAAGTAAGGGATGAGTGGGAGAAAAAACAGGCGGAAGCAGAGAAGCTTCGGAAA
CTAAATGATGTTGACAGCAATACTGGAGGTGATGGTGACAAGGATAAGGACGACGGCCGTGCTAAATCAACAAAGGTGAAC
AAGGAAGAGGATGACAAGATGAGGACAAATGCTGCAAATGTTGCTGCTCGTGCTGCTTATGGGGGAGATGACATGCTGTCG
AAGTGGCAACTTATGGCTGAGCAAGCCAAGCAGAAACGTGAAGGGGGGAGTAGACGTGTCATCTAGTTCTCAACCAGCTAA
AGATGTGAACCGCAAATCTTCATCAACATCTGAAAGAAGTACAAAGGATAACCAAGAAGGGGAAAAAAGAGGTTCAACCC
CTTTTCTAGCAAGCTCAGTGGCCAGAAAACTTGGGAAGAGTCATGCCATGGCCCCTCAAACTAGGGTAGCTCGAAGCATCT
CTGTCAAGGATGTGATTGCAGTCCTGGAGAGGGAGCCCCAAATGTCTAAATCCCCACTCATACATCGCTTGTACGAGAAAAT
TCATTCTGAAGCCCCAGTTGAACAAGGTTAA

>VrTAF4b-X5

ATGGACCCATCTATCATGAAGCTTCTAGAAGATGACGAGGATGAAACAATGCATTCAGGGGTGGATGTGGAAGCCTTCCAGG
CTGCTCTAAATAGAGATATAGGAGGAGATGTGTCTGCTTCTCAGTTTCCTGGTTCAGATGCAGTGTTGTCTCAAGGAAGCAAT
AATACATCAAGCCAATCTTCATCACAGTGGCCTACTTCTAACCATGACAGCCAAAGTGATGGTCAAAATCAAGAACCTAAAA
CTGCACAAGAGCAACATCCATCTGAGATGGAGCCAAAGCAACATGGGTCTCTTGGCGAACACCTTCAGCATGTTGCTTCTC
AGGATGTAAATAATATTCATTTATCACAGAAACAATCTCAAGATGACAGTCACCAAACACCTGCTGTACAAGTTCCCCTTCAC
AATTCTCAAACTATCGGAATTCATAATTCTGGAAAAGATTCAGTCCTTAATAAAGAAGTAGTAAAGAGTCATAATCCCAGCAG
TGAATCTCAGTATGCAAAGTTGCAGCAGATGAGTAATCAACAGGCTACAGTCTCTGAGCAGCCAAGTAGCCAAGTAAATCG
CAGTAATAAACAAGTACCATTTGGCCTGTTGCTACCCATCTTGCTTCCTCAACTTGCCAAAGACAGAGCCATGCAACTTCAA
ACCTTGTTTACTAAATTGAAGAAAGACGAAATACCGAAAGACAGTTTTGTGCGGCTTATGAAAGGTATCGTAGGGGACCAGA
TGCTTAGATTAGCATTAGCAAAGGTGCAAATGCAACCTCAGGCAAGGCCCAATCAAGCATCTGCTGGGCAGCAGCTTCCTGT
AAGGATGCCAACTGTTAGTTCAGGTGCTAGACAATTGAACGATCCACATGCTTTAGCACAGATGCATCAAAGAAGTATGAAT
GTTGCTGTTGACCAATCTCGCATGAGTTCTTCAGCTGGCCAAACCATGGATAGCAATGCTAGAAAATCTCAGGAATTTGATGT
TAAAATAGAATCTCAAGGATTGCAACCAAACCAGTTGACGTCTTCCACTTCCAACTCAGTAGCTCAAGAAACAGAAAGAGC
ATCAGTTCACATACAAGGGCTCAATAAGCAGCAGCAACATCATCTACATTTTGCATCAGCATATGGAAACAGTGGTGGTAACT
ATAACCCTTATTCTGGGACAACAGGCAGTTCCACTTCGTCTATCAAACCGCAATCACATGATTCACACATGAGTCAAATTCCT
CATCAAAGTATTGGTTCAAATCACTTAGGTGGGTCAACACATGGTTTGAGTGTAATTGGTATGCCAAAACTTGAACAGCAGA

ATTCTTTCAATGATCCCAAGAGACTGCCAGGTGGATCTGTGTCCTCTGCTGTAAACAATGCAGCATCTCAACAAACTTCAAAT
GCTTGGCAACCATCAACAAACAAAGAACAAAATCTGGGCTTAATGTCATCTGTTTCTTATGTCAAGAAGGAACCTGGTGACA
TGTCTACTGAGCAGCAAAATAGGCATAATTTGTCTAAATTGCATGGGTATTCTTCTGTTAATTCTGCTCAGCTTGAACAGAGT
GGTGCCAGTCAAGGAGCTCTAAAAGACGAGTTTTCAAGAGGTCTTCCAGCATCCACAAGCATGCCACCTACAACATCTACT
GGCTTGCTACCACACAGTTCTGCTTCTGCTTCTGCTGTGACCCACCTAGATTCCAGTGTCTTGATTCCATCAAATGCTTCTGG
AATTGCTGCAAGGCCATCTCTTAAAAAGTCTGCTGCTACCCAAAAGAAACCACTTGAAGCACTTGGTTCATCACCTCCTCCT
TCTAGTAAAAAACAAAAAACTTCTGGGGGATATGCTGAACAAAGCATTGAACAACTTAATGACGTCACTGCTGTTAGTGGA
GTTGACCTCAGGGAAGAGGAAGAGCAGTTATTTTCAGGGCCCAAAGAGGATAGTCGAGTGTCAGAAGCATCTCGAAAGGC
TGTGCAAGAAGAAGAGGAAAGGCTGATCTTGCAGAAAGCTCCATTGCAAAAAAAATTAATTGACATCATGGCCAAGTGTGG
CTTGAAGGGTATGAGCAATGATGTGGAGAAATGCTTGTCACTGAGTGTGGAGGAAAGGATGCGCGGACTGATAAGTAACCT
GATTAGATTATCAAAACAGAGGGTTGATTTTGAGAAAACGAGACATCGGACTGTTGTCACTTCAGATGTTCGGCAGCAAATC
ATGACAATAAATAGGAAAGTAAGGGATGAGTGGGAGAAAAAACAGGCGGAAGCAGAGAAGCTTCGGAAACTAAATGATGT
TGACAGCAATACTGGAGGTGATGGTGACAAGGATAAGGACGACGGCCGTGCTAAATCAACAAAGGTGAACAAGGAAGAGG
ATGACAAGATGAGGACAAATGCTGCAAATGTTGCTGCTCGTGCTGCTTATGGGGGAGATGACATGCTGTCGAAGTGGCAACT
TATGGCTGAGCAAGCCAAGCAGAAACGTGAAGGGGGAGTAGACGTGTCATCTAGTTCTCAACCAGCTAAAGATGTGAACCG
CAAATCTTCATCAACATCTGAAAGAAGTACAAAGGATAACCAAGAAGGGGAAAAAAGAGGTTCAACCCCTTTTCTAGCAAG
CTCAGTGGCCAGAAAACTTGGGAAGAGTCATGCCATGGCCCCTCAAACTAGGGTAGCTCGAAGCATCTCTGTCAAGGATGT
GATTGCAGTCCTGGAGAGGGAGCCCCAAATGTCTAAATCCCCACTCATACATCGCTTGTACGAGAAAATTCATTCTGAAGCC
CCAGTTGAACAAGGTTAA

>VrTAF4b-X6

ATGGAGCCAAAGCAACATGGGTCTCTTGGCGAACACCTTCAGCATGTTGCTTCTCAGGATGTAAATAATATTCATTTATCACA
GAAACAATCTCAAGATGACAGTCACCAAACACCTGCTGTACAAGTTCCCCTTCACAATTCTCAAACTATCGGAATTCATAATT
CTGGAAAAGATTCAGTCCTTAATAAAGAAGTAGTAAAGAGTCATAATCCCAGCAGTGAATCTCAGTATGCAAAGTTGCAGCA
GATGAGTAATCAACAGGCTACAGTCTCTGAGCAGCCAAGTAGCCAAGTAAATCGCAGTAATAAACAAGTACCATTTGGCCTG
TTGCTACCCATCTTGCTTCCTCAACTTGCCAAAGACAGAGCCATGCAACTTCAAACCTTGTTTACTAAATTGAAGAAAGACG
AAATACCGAAAGACAGTTTTGTGCGGCTTATGAAAGGTATCGTAGGGGACCAGATGCTTAGATTAGCATTAGCAAAGGTGCA
AATGCAACCTCAGGCAAGGCCCAATCAAGCATCTGCTGGGCAGCAGCTTCCTGTAAGGATGCCAACTGTTAGTTCAGGTGCT
AGACAATTGAACGATCCACATGCTTTAGCACAGATGCATCAAAGAAGTATGAATGTTGCTGTTGACCAATCTCGCATGAGTT
CTTCAGCTGGCCAAACCATGGATAGCAATGCTAGAAAATCTCAGGAATTTGATGTTAAAATAGAATCTCAAGGATTGCAACC
AAACCAGTTGACGTCTTCCACTTCCAACTCAGTAGCTCAAGAAACAGAAAGAGCATCAGTTCACATACAAGGGCTCAATAA
GCAGCAGCAACATCATCTACATTTTGCATCAGCATATGGAAACAGTGGTGGTAACTATAACCCTTATTCTGGGACAACAGGCA
GTTCCACTTCGTCTATCAAACCGCAATCACATGATTCACACATGAGTCAAATTCCTCATCAAAGTATTGGTTCAAATCACTTA
GGTGGGTCAACACATGGTTTGAGTGTAATTGGTATGCCAAAACTTGAACAGCAGAATTCTTTCAATGATCCCAAGAGACTGC
CAGGTGGATCTGTGTCCTCTGCTGTAAACAATGCAGCATCTCAACAAACTTCAAATGCTTGGCAACCATCAACAAACAAAG
AACAAAATCTGGGCTTAATGTCATCTGTTTCTTATGTCAAGAAGGAACCTGGTGACATGTCTACTGAGCAGCAAAATAGGCA
TAATTTGTCTAAATTGCATGGGTATTCTTCTGTTAATTCTGCTCAGCTTGAACAGAGTGGTGCCAGTCAAGGAGCTCTAAAAG
ACGAGTTTTCAAGAGGTCTTCCAGCATCCACAAGCATGCCACCTACAACATCTACTGGCTTGCTACCACACAGTTCTGCTTC
TGCTTCTGCTGTGACCCACCTAGATTCCAGTGTCTTGCTAAGCTCTCAGATTCCATCAAATGCTTCTGGAATTGCTGCAAGGC
CATCTCTTAAAAAGTCTGCTGCTACCCAAAAGAAACCACTTGAAGCACTTGGTTCATCACCTCCTCCTTCTAGTAAAAAACA
AAAAACTTCTGGGGGATATGCTGAACAAAGCATTGAACAACTTAATGACGTCACTGCTGTTAGTGGAGTTGACCTCAGGGA
AGAGGAAGAGCAGTTATTTTCAGGGCCCAAAGAGGATAGTCGAGTGTCAGAAGCATCTCGAAAGGCTGTGCAAGAAGAAG
AGGAAAGGCTGATCTTGCAGAAAGCTCCATTGCAAAAAAAATTAATTGACATCATGGCCAAGTGTGGCTTGAAGGGTATGA
GCAATGATGTGGAGAAATGCTTGTCACTGAGTGTGGAGGAAAGGATGCGCGGACTGATAAGTAACCTGATTAGATTATCAAA
ACAGAGGGTTGATTTTGAGAAAACGAGACATCGGACTGTTGTCACTTCAGATGTTCGGCAGCAAATCATGACAATAAATAGG
AAAGTAAGGGATGAGTGGGAGAAAAAACAGGCGGAAGCAGAGAAGCTTCGGAAACTAAATGATGTTGACAGCAATACTGG
AGGTGATGGTGACAAGGATAAGGACGACGGCCGTGCTAAATCAACAAAGGTGAACAAGGAAGAGGATGACAAGATGAGG
ACAAATGCTGCAAATGTTGCTGCTCGTGCTGCTTATGGGGGAGATGACATGCTGTCGAAGTGGCAACTTATGGCTGAGCAAG
CCAAGCAGAAACGTGAAGGGGGAGTAGACGTGTCATCTAGTTCTCAACCAGCTAAAGATGTGAACCGCAAATCTTCATCAA
CATCTGAAAGAAGTACAAAGGATAACCAAGAAGGGGAAAAAAGAGGTTCAACCCCTTTTCTAGCAAGCTCAGTGGCCAGA
AAACTTGGGAAGAGTCATGCCATGGCCCCTCAAACTAGGGTAGCTCGAAGCATCTCTGTCAAGGATGTGATTGCAGTCCTGG
AGAGGGAGCCCCAAATGTCTAAATCCCCACTCATACATCGCTTGTACGAGAAAATTCATTCTGAAGCCCCAGTTGAACAAGG
TTAA

>VrTAF4b-X7

ATGGAGCCAAAGCAACATGGGTCTCTTGGCGAACACCTTCAGCATGTTGCTTCTCAGGATGTAAATAATATTCATTTATCACA
GAAACAATCTCAAGATGACAGTCACCAAACACCTGCTGTACAAGTTCCCCTTCACAATTCTCAAACTATCGGAATTCATAATT
CTGGAAAAGATTCAGTCCTTAATAAAGAAGTAGTAAAGAGTCATAATCCCAGCAGTGAATCTCAGTATGCAAAGTTGCAGCA
GATGAGTAATCAACAGGCTACAGTCTCTGAGCAGCCAAGTAGCCAAGTAAATCGCAGTAATAAACAAGTACCATTTGGCCTG
TTGCTACCCATCTTGCTTCCTCAACTTGCCAAAGACAGAGCCATGCAACTTCAAACCTTGTTTACTAAATTGAAGAAAGACG
AAATACCGAAAGACAGTTTTGTGCGGCTTATGAAAGGTATCGTAGGGGACCAGATGCTTAGATTAGCATTAGCAAAGGTGCA
AATGCAACCTCAGGCAAGGCCCAATCAAGCATCTGCTGGGCAGCAGCTTCCTGTAAGGATGCCAACTGTTAGTTCAGGTGCT
AGACAATTGAACGATCCACATGCTTTAGCACAGATGCATCAAAGAAGTATGAATGTTGCTGTTGACCAATCTCGCATGAGTT
CTTCAGCTGGCCAAACCATGGATAGCAATGCTAGAAAATCTCAGGAATTTGATGTTAAAATAGAATCTCAAGGATTGCAACC
AAACCAGTTGACGTCTTCCACTTCCAACTCAGTAGCTCAAGAAACAGAAAGAGCATCAGTTCACATACAAGGGCTCAATAA
GCAGCAGCAACATCATCTACATTTTGCATCAGCATATGGAAACAGTGGTGGTAACTATAACCCTTATTCTGGGACAACAGGCA
GTTCCACTTCGTCTATCAAACCGCAATCACATGATTCACACATGAGTCAAATTCCTCATCAAAGTATTGGTTCAAATCACTTA
GGTGGGTCAACACATGGTTTGAGTGTAATTGGTATGCCAAAACTTGAACAGCAGAATTCTTTCAATGATCCCAAGAGACTGC
CAGGTGGATCTGTGTCCTCTGCTGTAAACAATGCAGCATCTCAACAAACTTCAAATGCTTGGCAACCATCAACAAACAAAG
AACAAAATCTGGGCTTAATGTCATCTGTTTCTTATGTCAAGAAGGAACCTGGTGACATGTCTACTGAGCAGCAAAATAGGCA
TAATTTGTCTAAATTGCATGGGTATTCTTCTGTTAATTCTGCTCAGCTTGAACAGAGTGGTGCCAGTCAAGGAGCTCTAAAAG
ACGAGTTTTCAAGAGGTCTTCCAGCATCCACAAGCATGCCACCTACAACATCTACTGGCTTGCTACCACACAGTTCTGCTTC
TGCTTCTGCTGTGACCCACCTAGATTCCAGTGTCTTGCTAAGCTCTCAGATTCCATCAAATGCTTCTGGAATTGCTGCAAGGC
CATCTCTTAAAAAGTCTGCTGCTACCCAAAAGAAACCACTTGAAGCACTTGGTTCATCACCTCCTCCTTCTAGTAAAAAACA
AAAAACTTCTGGGGGATATGCTGAACAAAGCATTGAACAACTTAATGACGTCACTGCTGTTAGTGGAGTTGACCTCAGGGA
AGAGGAAGAGCAGTTATTTTCAGGGCCCAAAGAGGATAGTCGAGTGTCAGAAGCATCTCGAAAGGCTGTGCAAGAAGAAG
AGGAAAGGCTGATCTTGCAGAAAGCTCCATTGCAAAAAAAATTAATTGACATCATGGCCAAGTGTGGCTTGAAGGGTATGA
GCAATGATGTGGAGAAATGCTTGTCACTGAGTGTGGAGGAAAGGATGCGCGGACTGATAAGTAACCTGATTAGATTATCAAA
ACAGAGGGTTGATTTTGAGAAAACGAGACATCGGACTGTTGTCACTTCAGATGTTCGGCAGCAAATCATGACAATAAATAGG

AAAGTAAGGGATGAGTGGGAGAAAAAACAGGCGGAAGCAGAGAAGCTTCGGAAACTAAATGATGTTGACAGCAATACTGG
AGGTGATGGTGACAAGGATAAGGACGACGGCCGTGCTAAATCAACAAAGGTGAACAAGGAAGAGGATGACAAGATGAGG
ACAAATGCTGCAAATGTTGCTGCTCGTGCTGCTTATGGGGGAGATGACATGCTGTCGAAGTGGCAACTTATGGCTGAGCAAG
CCAAGCAGAAACGTGAAGGGGGAGTAGACGTGTCATCTAGTTCTCAACCAGCTAAAGATGTGAACCGCAAATCTTCATCAA
CATCTGAAAGAAGTACAAAGGATAACCAAGAAGGGGAAAAAAGAGGTTCAACCCCTTTTCTAGCAAGCTCAGTGGCCAGA
AAACTTGGGAAGAGTCATGCCATGGCCCCTCAAACTAGGGTAGCTCGAAGCATCTCTGTCAAGGATGTGATTGCAGTCCTGG
AGAGGGAGCCCCAAATGTCTAAATCCCCACTCATACATCGCTTGTACGAGAAAATTCATTCTGAAGCCCCAGTTGAACAAGG
TTAA

>VrTAF5-1-X1

ATGGACGAAGATCAGATAGAAGGTTGTGTGAGTGGGTACCTCAAGCAGAAAGGGTTTGCGCAAAAGGATGACCAACTTCA
ACTTTCCAACGCGGATTCCTCTCTTCAACCCGACACTCTCAATCGCGCTCAATTGGAGAGAGGTTCTGCTAGATACCACGAT
GGATATGGAAGACTGAGATCATGGGCATATAGGTCACTTGAATCATACAAGCACGAATTACTGCGTGTGCTTTATCCAGTATT
CGTCCATTGCTTCATGGATCTTGTGGCAAAAGGGCATCTTCAGGAAGCTTGGAACTTTTTCAATACCTTTCGTGAGGACCATG
AAATGTTGCACTCACGAGACCTTCAGAAATTGGGATTAGTTCTTTCTCCTACTCATTTGGAGGAAATGGAATTCGCTCATTCC
CTTAGGCAGAGTAAGTTCAACATAAAGATATGTCGGTATTCTTATGAGCTTCTATTGCAACATCTACATAGTATGCAATCCACC
ACAATAATTGGAATTATCAATGAGCATATTAACTTCCAAGTTACAGCTGGGCAACCGAGCTCAATTTCTGATGATCCAGAAGC
TGTTACTCTCAGTGGAAGCATCCAGGATGCAGTAAACCAGATAAATCAGAAAGAAATCCTTTGGGGGATGTTTGAAGATTCT
GTGGAAGACCACGTAGATAAGGCTGGGGCCTTGCTTTCAGGCACAGAAAAGGGTGAAGGCGAAGGCAAAGAGGGGGACA
ATGATGAGAGTAAGAAAAGGTCAATTGATGTAGGAAAGCAAGGTAATTCAGTAAAAAAGGTAAAAAAGGACAAGGTCAGC
AGTGCAACAGGGAAAAATGCTAAGCCAGAAGCTACCACTATATCTGCAGCACCTCGCATTAAACCAGAGATACCTTTGCCCA
TAGTTTCAACTGATGTAGAACTGTCTATCCTAGACGATTTAAGGAATCGTGTACAATTGAGTAGTGTTGCTCTCCCTTCAGTG
AACTTCTACACATTTGTAAATACACATAATGGTTTAAGCTGTTCATCAATATCCCATGATGGGTCATTGATTGTTGGAGGATTTT
CTGACTCTTCACTAAAGGTCTGGGATATGGCAAAACTTGAGCAACAATCCACTGCTCATTTTTCGCAGGGAGGGAATGACAT
GTCACAAAACGAACAAATTATTGGGCAAAATAGTGGGAGAAGACAGTACACATTGTATCAAGGTCACTCAGGTCCAGTCTAT
GCAGCCACTTTTAGTGCTGCCGGGGATTTTCTTCTTTCCTCTTCAGCAGACAAAACAGTTCGACTATGGAGTACAAAGCTTA
ATGCCAATCTTGTTTGTTACAAGGGTCACAATTACCCTATCTGGGATGTTCAGTTTAGTCCTGCAGGACATTATTTTGCCAGCT
GTTCACATGACAGAACTGCTAGGATTTGGTCTATGGACAGAACAAAACCTTTAAGAATAATGGCAGGACATTTGTCAGACGT
TGATTGTGTGCAGTGGCATCCCAACTGCAACTACATTGCTACTGGCTCCAGTGATAAAACAGTTCGACTGTGGGATGTGCAG
AGCGGGGAATGTGTTCGGGTTTTTATTGGTCATAGAAGTATGATTTTGTCTTTGGCAATGTCCCCTGACGGTCGCTACATGGC
ATCTGGTGATGAAGATGGCACAATCATGATGTGGGACCTCTCTAGTGGCTGTTGTGTCACACCTCTTGTCAGTCACACTTCAT
GTGTCTGGTCACTCGCTTTCAGTTGTGAAGGTTCTCTTCTAGCATCTGGATCTGCTGATTGCACCGTCAAATTTTGGGATGTG
ACTACGGGTATAAAGGTTCCAAGGAATGAAGAAAATAGAAGTGGAAATGCTAACAGACTCAGATCATTAAAAAGCCTGCCA
ACCAAATCTGCTTCAGTTTACTCTCTCCAGTTTTCTCGCAGGAATCTTCTTTTTGCAGCTGGAGCTGTTGCAAAAAGTGGTTG
TTAG

>VrTAF5-1-X2

ATGGACGAAGATCAGATAGAAGGTTGTGTGAGTGGGTACCTCAAGCAGAAAGGGTTTGCGCAAAAGGATGACCAACTTCA
ACTTTCCAACGCGGATTCCTCTCTTCAACCCGACACTCTCAATCGCGCTCAATTGGAGAGAGGTTCTGCTAGATACCACGAT
GGATATGGAAGACTGAGATCATGGGCATATAGGTCACTTGAATCATACAAGCACGAATTACTGCGTGTGCTTTATCCAGTATT
CGTCCATTGCTTCATGGATCTTGTGGCAAAAGGGCATCTTCAGGAAGCTTGGAACTTTTTCAATACCTTTCGTGAGGACCATG
AAATGTTGCACTCACGAGACCTTCAGAAATTGGGATTAGTTCTTTCTCCTACTCATTTGGAGGAAATGGAATTCGCTCATTCC
CTTAGGCAGAGTAAGTTCAACATAAAGATATGTCGGTATTCTTATGAGCTTCTATTGCAACATCTACATAGTATGCAATCCACC
ACAATAATTGGAATTATCAATGAGCATATTAACTTCCAAGTTACAGCTGGGCAACCGAGCTCAATTTCTGATGATCCAGAAGC
TGTTACTCTCAGTGGAAGCATCCAGGATGCAGTAAACCAGATAAATCAGAAAGAAATCCTTTGGGGGATGTTTGAAGATTCT
GTGGAAGACCACGTAGATAAGGCTGGGGCCTTGCTTTCAGGCACAGAAAAGGGTGAAGGCGAAGGCAAAGAGGGGGACA
ATGATGAGAGTAAGAAAAGGTCAATTGATGTAGGAAAGCAAGGTAATTCAGTAAAAAAGGTAAAAAAGGACAAGGTCAGC
AGTGCAACAGGGAAAAATGCTAAGCCAGAAGCTACCACTATATCTGCAGCACCTCGCATTAAACCAGAGATACCTTTGCCCA
TAGTTTCAACTGATGTAGAACTGTCTATCCTAGACGATTTAAGGAATCGTGTACAATTGAGTAGTGTTGCTCTCCCTTCAGTG
AACTTCTACACATTTGTAAATACACATAATGGTTTAAGCTGTTCATCAATATCCCATGATGGGTCATTGATTGTTGGAGGATTTT
CTGACTCTTCACTAAAGGTCTGGGATATGGCAAAACTTGAGCAACAATCCACTGCTCATTTTTCGCAGGGAGGGAATGACAT
GTCACAAAACGAACAAATTATTGGGCAAAATAGTGGGAGAAGACAGTACACATTGTATCAAGGTCACTCAGGTCCAGTCTAT
GCAGCCACTTTTAGTGCTGCCGGGGATTTTCTTCTTTCCTCTTCAGCAGACAAAACAGTTCGACTATGGAGTACAAAGCTTA
ATGCCAATCTTGTTTGTTACAAGGGTCACAATTACCCTATCTGGGATGTTCAGTTTAGTCCTGCAGGACATTATTTTGCCAGCT
GTTCACATGACAGAACTGCTAGGATTTGGTCTATGGACAGAACAAAACCTTTAAGAATAATGGCAGGACATTTGTCAGACGT
TGATTGTGTGCAGTGGCATCCCAACTGCAACTACATTGCTACTGGCTCCAGTGATAAAACAGTTCGACTGTGGGATGTGCAG
AGCGGGGAATGTGTTCGGGTTTTTATTGGTCATAGAAGTATGATTTTGTCTTTGGCAATGTCCCCTGACGGTCGCTACATGGC
ATCTGGTGATGAAGATGGCACAATCATGATGTGGGACCTCTCTAGTGGCTGTTGTGTCACACCTCTTGTCAGTCACACTTCAT
GTGTCTGGTCACTCGCTTTCAGTTGTGAAGGTTCTCTTCTAGCATCTGGATCTGCTGATTGCACCGTCAAATTTTGGGATGTG
ACTACGGGTATAAAGGTTCCAAGGAATGAAGAAAAAAGTGGAAATGCTAACAGACTCAGATCATTAAAAAGCCTGCCAACC
AAATCTGCTTCAGTTTACTCTCTCCAGTTTTCTCGCAGGAATCTTCTTTTTGCAGCTGGAGCTGTTGCAAAAAGTGGTTGTTA
G

>VrTAF5-2

ATGGAGGATGATAAAATAGTTGGATATGTTACCGCGTACCTGAAGAAGAAAGGTTTCACGCAAACCGAGAAGATTTTCCAGG
AAGAGTTTCAGCAAAACAAAACCAACAATTCCTCCTCCAGTAATTCCCTTCTCGAACCCGACATTGCCAACCACCTCCTCGC
ATTCTCTCAATTGGAGAGTGGTCCGGCTAGGTTCCACGACGGCTACAGCAAACTGCGAACATGGACTTATTCTTCACTGGAT
TTGTACAAGCACGAGTTGCTTCGCGTGCTTTATCCCGTCTTTATCCACTGCTTTATGGACCTCGTGGCGAAAGGACATGTTCA
GGAAGCTCGGAATTTCTTCAATACTTTCCGTGAAGACCACGAAATGATGCACTTGCGTGATCTTCAGAAGTTAGAAGGGGTT
CTTTCTCCTACCCATCTAGAGGAAATGGAATTCGCTCACTCGCTTAGACAGAGCAAATTCAACATAAAGATATGTGAGTATTC
CTATGAGCTTCTCTTGCAACATCTACACAGTACTCAATCCACCACTATACTCGGTATTATAAATGAGCATATTAACTTTCAAGTT
TCCCCTGGACAACCTAGCTTAATTTCTGATGATCCGGAAGCTGTTACCCTTACTGGAAGCAGCCAGGAAGCAGCAAACCGGA
TAAATCAAAAAGAAATTCATTGGGGGTTACTTGAAGATTCTCTTGAAGAACGGCTAGAGAAGGCAGGGACCTTGCTTTCAG
ACTCGGAAAAGGGTGAAGGGGAAACAAAAGAGGGGGAGAATGACGAGACTAAGAAAAGATCCATTGAAGGAGGAAAGC
AAGGCGGTTCAGTAAAAAAAGTAAAAAAGGACAAGGGTGGTAGTACAACTGGAAAGAGTGCAAAACCTGAAGCTAGTACT
GTACCTGCAGCTCCTCGAGTTAAGCCTGAACTCCCCTTACCCTTAATCCCAACTGAGGTGGAACAGTCCATCCTTGAAGATTT
AAGGAACCGTGTACAGCTCAGCAGTGTTGCATTGCCGTCAGTTAGCTTTTACACATTTATAAATACACATAACAGTTTAAGCT
GTTCATCGATATCCCATGACGGATCATTAATTGCTGGAGGATTTTCTGACTCATCACTCAAGGTTTGGGATATGGCAAAGCTTG

GACAACCGACTGCCAGTTCTCTTTTGCAGGGTGAGAATGACACATCACCAAATGATCAAATATTTGGGCAAGGGGTTGGGA
AAAGACAGTATACACTATTTCAAGGTCATTCAGGACCTGTTTATGCAGCCTCTTTTAGTCCCGTGGGTGATTTTATCCTTTCAT
CCTCAGCAGACTCAACAATTCGGTTATGGAGCACCAAACTTAATGCCAATCTTGTTTGTTATAAGGGTCACAATTACCCTGTC
TGGGATGTTCAGTTTAGTCCTGTAGGGCATTATTTTGCCAGCTCTTCACACGACAGAACTGCTAGGATTTGGTCAATGGACAG
GATACAGCCCTTAAGAATAATGGCAGGGCACTTATCTGATGTTGATTGTGTACAATGGCATGCCAACTGCAACTACATTGCAA
CTGGTTCCAGTGACAAAACAGTTCGACTATGGGATGTGCAGAGCGGTGAGTGTGTCCGGGTTTTTGTTGGTCACAGGGGTAT
GATTTTGTCTTTGGCAATGTCTCCTGATGGTCGCTATATGGCATCTGGGGATGAAGATGGCACGATCATGATGTGGGACCTCT
CTAGTGGCCGCTGTCTCACCCCTTTGATTGGGCACACGTCTTGTGTCTGGTCCCTTGCGTTCAGTTCTGAAGTTTCAATTCTA
GCATCTGGATCTGCTGATTGCACTGTAAAATTGTGGGATGTAAATACGAGCACCAAGGTTTCAAGGGCTGAAGAAAAAAGT
GGGAATGCTAACAGACTTAGATCGCTGAAAACTCTGGCAACCAAATCAACCCCAGTTTACTCATTGCGGTTTTCTCGAAGGA
ATCTTCTCTTTGCGGCTGGGGCTCTAGCAAAAAGTGGGTAA

>VrTAF6-1

ATGAGCATTGTTCCCAAAGAAACGATCGAAGTTATAGCGCAGAGCATTGGGATAAACTCCTTGTCTCCCGATGTCGCTCTCG
CTGTCGCTCCCGATGTCGAATACCGCATGCGCCAGATTATGCAGGAGGCAATTAAGTGCATGCGGCATTCTAAGAGAACCATT
CTCACTGCGGATGATGTTGATGTTGCTCTGAATTTGAAGAATGTTGAACCAATATATGGATTTGCATCTGGTGGTCCTTTGCGG
TTCAAAAGAGCTGTTGGACACAGGGACTTGTTTTATATTGATGACAAGGATGTAGATTTAAAAGATGTTATTGAAGCTTCTTT
ACCAAAAGCACCTCTTGATACTGCAGTTACATGCCACTGGCTTGCCATTGAAGGTGTGCAACCTGCTATTCCAGAAAATGCT
CCTATAGAAGTAATTTCAGCTCCTTCTGATACCAAAAAGCATGAGCAGAAAGATGATGACCTTCCAGTTGACATCAAATTGCC
TGTTAAGCATATATTATCCAGAGAACTTCAGATGTATTTTGACAAAGTTGCTGAGCTTACTTTGAGTGAGTCTGATTCAGCTCT
CTTTAAAGAAGCATTAGTAAGTTTGGCTACTGATTCGGGGCTTCATCCACTAGTTCCTTATTTCACATGCTTTATAGCTGATGA
GGTTTCACGTGGTTTGAATAATTTTCCTCTTCTATTTGCCTTGATGCGAGTTGTTAGTAGCCTTCTGCTAAACCCTCACATCCAT
ATTGAACCTTATCTACACCAGTTGATGCCATCTGTTGTGACCTGCCTCGTTGCTAAAAGGTTGGGCAGTAGGTTGGCAGACA
ACCACTGGGAACTTAGAGACTTTACGGCTAACCTGGTTGCCTCAATATGCAAAAGGTTTGGACATGTCTATAGTAATCTCCAG
TATCGGTTGACTAAAACATTGCTGAATGCATTCTTGGATCCTAAGAAGGCAATGACGCAACACTATGGAGCGATTCAGGGGT
TGGGGGCACTTGGACCCAACGTGGTTCGCCTTCTTTTGCTGCCAAACCTTGAGACATATATGCGACTTCTTGAACCAGAGAT
GCTTCTTGAGAAGCAGAAAAATGAAATGAAAAGGCATGAAGCTTGGCGTGTTTATGGAGCCTTGCTGCGTGCTGCAGGTCA
GTGTATATATGATCGACTAAAGATGTTCCCAACTTTTTCAACTCCTTCTCCTAGTGCTGTCTGGAAGACCAATGCAAAAGTTC
TTACTTCTTCATCTCGTAAACGCAAGGCAGACCCTGACCAATTGGAACAGCAGCCACCTTTGAAAAAAACTGCTACCGACG
GAGAGGTTGGTGTGGTCCCAATGAATTCCTCACCCGCTCACAAGCAAGAGGAGGCAGAGACTCGAGCTTCTTCAGTCGATT
CAATTATTGGCTCATCATCTTCTGCACAGATGAAAAATGAGACTTCTTTAGATGGCGAACTTAGAAGTAACAAGGGTGATACT
CAGGCATCGAAGACATCTGCTGCTCTCACCCAGGTTTGGAAAGACGAGCTTAATTCTGGACGGATTCTGGTATCACTGTTTG
ACTTGTTTGGTGAAGGAATTCTTTCCTTCATTCAGGCTCCTGAGATGTATATGTTCTTGTAA

>VrTAF6-2

ATGAGTTTTGTGCCAAAGGAGACAATCGAAGTCATCGCACAGAGCATCGGCATCACCAATTTGTCCCCCGATGTTGCCCTCG
CTCTCGCCCCCGATCTCGAATATCGCATTCGGGAAATCATGCAGGAGTCGATAAAATGCATGCGCCATTCGATGAGAACTTTT
CTTAGTACAGAGGATGTGGATACTGCACTTGCATTGAGAAATTTGGAGCCGATATATGGGTTCACCTCTAATGATCCTCCGAG
GTTCAAAAGAGCTGCTGGACATAAGGATTTGTTCTACATCGATGACAAAGATGTGGATATCAAAGATCTTATTGAAGCTCCTT
TACCGAAAGCACCCCTTGATACATCAATTACCAGTCACTGGTTGGCTATTGAAGGTGTGCAACCTGCAATTCCTGAAAACGC
TCCAGTTGAAGCTCCTTCCTCTGAGATAAGAAAATCTGAATATAAGGAAGATGGGCTTTCTGTTGATGTTAAATTACCTGTTA
AACATTTAATAACAAGGGAGCTTCAGCTCTACTACGAGAAAATAACTGACCTTACATTAAATAAGTCTGGATCCATTCCGTTC
AGAAGAGCATTGGTTAGCTTGGCAACGGACTCGGGACTCCATCCCTTAGTTCCATATTTCACATGTTTTGTTGCCGATGAGGT
GGCACGTAATTTACATAATTTAGCTGTTTTATTTGCCTTGATGCGCCTTGTGCGGAGCCTTGTGCAAAATTCTCAGATACACAT
AGAACTTTATTTACATCAATTGATGCCACCTATCATTACTTGCCTTGTTGCAAAAAGGATAGGAAGCAGACTATCTGACGATCA
TTGGGAGCTTAGGAACTTCAGTGCTAATCTTGTTGCTTCAATATGCCAAAGATTTGGGCATATTTATCACAATCTGCAGCCAC
GTGTGACAAAGACATTTCTTCATTCTTTCTTGGACCCTACAAAAGCTCTGCCTCAGCACTATGGTGCAATTAAAGGGATAGA
AGCTCTTGGATCAAGAGTGATTCACTTGCTTATACTTCCAAATCTCGAGCCATATTTGCATCTTCTTGAGCCAGAAATGCAAC
CTGAGAAACAAAAAAATGAAATGAAGAGGCATGAAGCTTGGCAGGTTTATGGAGCTTTGTTGGGTGCAGTAGGCAAATGTG
TGCATGAAAAGGTAAAAAATTTCAGCAATTTGTTCTCTCTTCCAACTCGGCTTATTTCAAGAGGCAGTGGAAAAGCCATTATT
CCAATGTCAGGCAAACGAAAAGCCAGTGCCGACAATCTGATGCAGCAGCAACCTCCAATGAAGAAACTTTCAACAGATGG
CCATGGAGGTGTAATACCAATGAACTCCATGTCAGTTGACATGCAAGGCTCAACAGGGGGATTTTCCACCATGATGGGGGCT
CCTGCAATGTCGATGACTCGTCAAATTTCAACCGACAAGGCGTCAGGGAGGGAGGTTGTAGATCAACAAAAGAAGGTGTCT
GCCACTCTTGCTCAGGCTTGGAAGGATGACATTGATGCAGGAAATTTGGTGTCATCAGTGGTTGAATTGTTTGGCGAAAGGG
TGTTACCATTTGTTCCAAACCCTGAAGCATTTATGTTTTTGTAG

>VrTAF6-like

ATGCATATTAGAACTACCTTGAGGAAATTTTTGCCAATATATGGATTTGCATCTGGTGGTCCTTTGCGGTTCAAAAGAGCTGTT
GGACACAGGGACTTGTTTTATATTGATGACAAGGATGTGGATTTAAAAGATGTTATTGAAGCTTCTTTACCAAAAGCACCTCT
TGATACTGCAGTTACATGCCACTGGCTTGCCATTGAAGGTGTGCAACCTGCTATTCCAGAAAATGCTCCTATAGAAGTAATTT
CAGCTCCTTCTGATACCAAAAAGCATGAGCAGAAAGATGATGACCTTCCAGTTGACATCAAATTGCCTGTTAAGCATATATTA
TCCAGAGAACTTCAGATGTATTTTGACAAAGTTGCTGAGCTTACTTTGAGTGAGTCTGATTCAGCTCTCTTTAAAGAAGCATT
AGTAAGTTTGCCTACTGATTCGGGGCTTCATCCACTAGTTCCTTATTTCACATGCTTTATAGCTGATGAGGTTTCACGTGGTTT
GAATAATTTTCCTCTTCTATTTGCCTTGATGCGAGTTGTATCAAACGGAAATTCAGCTCCAAACTTGGAGACTAAATAG

>VrTAF7

ATGGAGGAGCAATTCATACTTAGAGTTCCACCGAATGTGGCAGAGAGAATAGAGCGGCTTTTGAATGAAACTGATCCTTCTT
CATCTGAAGACAAGTCATTAGATTTGTCATTTAGTGAGGATGGAAGAAGTGGTACGTTTGTGATTGGGAATGAACACTTCCC
AGCTTCTCTATTGGACCTTCCTTGTGTTGTTGAATCCTACAAGACATATGATGATAACTCTTTGATTAAGACTGCTGATATTGG
TCAGATGATTATGGTTCGGGAATCTGGTGATGCTGCTCCAGATGTAATTGAGTACAGGCATGGTCTCACCCCGCCGATGAGAG
ATGCTCGCAAGCGTAGATTTCGCAGGGAGCCCGATCTTAATCCCGAGCTTGTGTCCCGTGTTGAGAAAGATCTCCTCAAAAT
CATGGCTCGAGGAACAGCTGAAAATATTGATGCAGAAGCCGCTGAACAAGAAGTGGAGGAGAATGCCCGAGGTGCTAACA
AAAAGGTTGCACCTAAGCCTGCACCAAAACATGATGTTCCAGAGAATCTTACAAATGCAGGGGAGCCTGACAGGAGTGATT
CTGAAGAATCTGATGATTCAGTCTGA

>VrTAF8-1

ATGGCTTCACTAGGACTAATTTCTGATTTTGAATTGGAAAACAAAAGTCATTTGGGTTATAAAGGAAAGGTGCTGAATATGAG
CAATGGCGGTGGGAAGACTGGAAGACAGCTTGAGCAGCCTGGCCCATGGAGGAGGAGGAAAGTGGGTGATGGGGATGACT
TTGCCAGGGCAATTGCGAAGATTGCGGTAGCGCAGGTGTGCGAAAGCGAGGGGTTTCAGGCTTTTCAGCAGTCGGCTCTTG
AGGCATTGTCTGACGTTGTGGCTCGGTACATTTTTAACGTTGGGAAATCGGCACATTGCCATGCTAATCTTGCAGGAAGAACT
GAATGCCATGCTTTTGATGTCATTCAAGGGTTGGAAGATATGGGATCAGTCCAGGGATTTGCAGGTGCTTCTGAGGTGGATCA
TTGCCTTGAAAGTTCAGGTGTTATTAGGGAAATTTTTCATTTTGTTAACGAGGGTGAACCGGTTGTGTTTGCGCATCCCATTC
CTCGGTTTCCAGTTGTGAAGGAACGGGTGCTTAATCCAAGCTTTTTGCAAAAAGGAGAAGAACCTCCCGGCGATCATATTCC
TGCTTGGTTGCCTGCCTTCCCTGATCCGCAAAATTATTCACAGCCACCAGTGGTGAATGGAAGGGGCACAGAACCTCGTGCA
GTAAAATTTGAGCAAGAAAGAGAGAACGGCAAGGGGGAGTGGCCTGTGTTGAATTTGAAGCAGCAGATGGTCTCAAATAT
GTTTGAAAAGTCTGCCTTGGACCCTGCAGATACTAAGGCAAAACGAATAGCAGCAGAAGGTAACCCATTCCTTGCTGCTCCT
TTGAAAATTGAGGACAAGGAAATTGCATCTGTTCCCCCTGCAGCCAAGCTTTTCAATGATGTAGTTCTGGATTATCCTGTGGT
TGAAAATTTCGTTGAAAATGAACCAATTTCAGCTTTGGAGACATTTGCTCCTGCAATTGAAGCAATGAAAAGCACATGCTGT
GATTCTAAGGAAGGCCAGACAAAAAGTTTTGTGAATGAGAAGCCTATTGTGCGTTTTAAGATTGGGATCAAAAACAAATTAT
TAGGAAGGTCCATTGGTTTGATCCCACAAACAGAGGAGCATAACAAGACTTTGCCATGGTTTGCGATGGAAGATGAGAAGG
ATGACAGGAAAAGGAGGGCAGAGAAAATTCTAAGGGAATCCTTGGAAAACCCAGATCAGCTTGTTCAGTTGTAA

>VrTAF8-2

ATGAACCCTATGCTAAAGGACAGCAACACAAAAGCCACATCAAAACTACCCCGAAGGGGAAAAAGAAAAAAGAAGGGTCT
GGGAGTAACGGATCCTCAAGTAGCAGAAAACCCATCAAAATTCTCGTTTGCTATAGCCAAAATTGCAGTTGCCCAAATCTGT
CAATCAGCTGGATTCAAAAAGTCCGAAAACAATGCTCTTGAAACCCTAACTGCTGTTTCCACTAGATATCTGGAAGCAATTG
TGAGATCAGCTGCCTCATTTGCTAATGCCTCCAATCGTACTGACTGCAATCTCTTTGACCTCGTCAATGGCATTCATGATCTTT
GTTCTGTTCAAGGATTTCCGGGTGGTTCAGCATTGCACAAAGATGATCTGCTGAGGTCTTCAGCTCTAAGAGAGATTATGAAT
TTTGTCAACCTCTCTGATAAAGTTCCTTTTGCTAAACCAATTCAATGTAGAAATGATTCCGATGTAACCATTGATTCTGGGACA
TTAATGTGCTTCTCTAACCAAACCAAAACTCACATACCAGGATGGCTCCCACATTTTCCTGAGCAAAACTGTGATCAGGTTTT
AGTTAAGGAAAGGAAATGCGGCGAGAAATATTGGGAGGATTCATTTACTGTGGACGAAAACAGTGTCATATCACAGAGCAA
TCACATGAATGGAAAAGAAGGGAAAGACACAAGGAGGGAATTGCCAGAGCGAAGAGAAAGAATGAAGTTTAAAATTAGA
GGGGAGGAGGAGAAGCAAGATGGATTGGGTGTGAATATGATGAGTGGGGTTTGTAAAGGAAGGAAACGAGTGTCTTGGAA
TCATTACAAAATGAATGGTTGTATTATTGAGAACAACAAAGATGAGAAAAGATAG

>VrTAF8-like

ATGACCAACGGCGGAGGAAGAGCCGCGCCGGACGACTACGGCCGAGCCGCAGTACGTCTCGCGTTGGCGCAGCTCTGCAA
CGCCGCTGGGTTCCACAGCGCCACCTCCTCTGCGCTGGACGCCTTCGCCGACGTCGCAATCCGCTACCTGCTCGACCTAGGC
AGAACCGCGGAGTCTCATGCGAACCACGCCGGCCGATCGCAGTGCACCGTCTTCGACGCGATTCGCGGCTTCGAGGACCTG
GGAGCGCCGCGAGCCTTCTCCAGCCCCGGCGGAGTCAGAGATATCGTGAGTTTCGTGGAATCGGCAGACGAGGTTCCGTTC
GCTCAGCCCATTCCGCGGTTTCCGGTCGTTCAGGAACGACGTCGCATTCCGAGTTTTTATCAGATGGGGGAGACTCCACCGT
CGAAGCATATTCCCTCGTGGTTACCGGCTTTGCCAGATCCCCATACGTATATTCACACTCCAGTGTGGGATGAGAGGGTTTCT
GATCCTCGTGAAGATAAAATCGAACAAGCTAGGCAGCGTAGGAAGGCTGAGAGGTCGTTGTTGAGTTTGCAGAAACGGTTG
TTGTTGCGAAATGGGTCATCGGAAGCTAAAGCAAGAACGTCAGCTTCGCCAGATAGTACTGTTTTGGAAACTCAATGCGTTG
GTGATGACCATAAGGATGTTGATAAAGATGGCGCTCCGGTTGTTAAGGTTTCAGTTTTGGATGAGGGTAATGTTGGTGACAG
GAACCGTGTTTCGGTGTTGGATGCGTTTGCTCCGGCAATTGAGATGCTTGGGAGTGGAGGGTTGGGTGGTGATGAAGATGAT
GGATTGGTGGAGATAGACAGAAGTGAGCTTCCTGTTGTGAGACCTACTGTGCATTTTAAGTTTAGGACCGGGAAAAAGTTCA
TTGGGGAGTCTTTGGATATGAGAATTCGGAATAAGGATGCGTCACGGACGGTGGGATTGGTTGGGAGAGAAGATGAGAGGG
ATGATAAGAAAAGGAGGGCTGAGTATATTCTCAAACAGTCTATGGAGAACCCCCAGGAACTCACTCTGTTGTAG

>VrTAF9-1-X1

ATGTCAGATAAAGATGAAGAGTTGGCTATGCCAAGGGATGCAAAGATTGTGAAGTCTTTGTTGAAATCAATGGGCGTGGGG
GAATACGAACCTCCTGTTATACACACGTTCCTTGAGTTATGGTATCGCTATATAGTTGATGTGTTAACGGATGCCCAAGTGTAT
TCAGAGCACGCAGGCAAGTCCGAAATCGACTGCGATGATGTTAAGCTTGCTATTCAATCCAAGCTTAACTTCAGTTTCTCGC
AACCACCTCCTCGCGAGGTGCTTCTGGAGTTGGCTCAAAACCGCAACAAGATACCACTGCCAAAGACTATAGCTGGGCCTG
GTATCCCGCTTCCACCTGATCAGGACACGTTAACCAGTCCGAACTACATGTTTGGAATTCCAAGCCAAGGGTCTGGTGAACC
TGAAGAAACAGAGGATGAAGAAACTAGTATTCCCAACCCCTCTCAGGAAGAGAAGACAGACATGCAACAGCAGGATCCCC
ATCAAAGACTTGTTCAATTTGTACTTTTGACAAATTGGTTTCAAAACGGCTCGTTGCGGTCTGAAGTTCTTGTGAACTGA

>VrTAF9-1-X2

ATGTCAGATAAAGATGAAGAGTTGGCTATGCCAAGGGATGCAAAGATTGTGAAGTCTTTGTTGAAATCAATGGGCGTGGGG
GAATACGAACCTCCTGTTATACACACGTTCCTTGAGTTATGGTATCGCTATATAGTTGATGTGTTAACGGATGCCCAAGTGTAT
TCAGAGCACGCAGGCAAGTCCGAAATCGACTGCGATGATGTTAAGCTTGCTATTCAATCCAAGCTTAACTTCAGTTTCTCGC
AACCACCTCCTCGCGAGGTGCTTCTGGAGTTGGCTCAAAACCGCAACAAGATACCACTGCCAAAGACTATAGCTGGGCCTG
GTATCCCGCTTCCACCTGATCAGGACACGTTAACCAGTCCGAACTACATGTTTGGAATTCCAAGCCAAGGGTCTGGTGAACC
TGAAGAAACAGAGGATGAAGAAACTAGTATTCCCAACCCCTCTCAGGAAGAGAAGACAGACATGCAACAGCAGGATCCCC
ATCAAAGAGTATCATTTCCAACTTCCTTTCATGATTAA

>VrTAF9-2

ATGGGTGACAAAGACGAAGAGTCAGGAATGCCAAGGGATGCAAAGATCGTGAAGTCTTTGTTGAAGTCAATGGGCGTGGA
GGAGTACGAGCCTCGCGTCATTCACAAGTTCCTTGAACTATGGTATCGATACGTCGTCGATGTATTAACAGACGCACAAGTCT
ATTCAGAGCACGCCGGAAAATCCTCAATCGATTGTGACGATGTCAAGCTTGCGATTCAATCCAAGATTAACTTCAGCTTCTCG
CAACCACCACCCCGTGAGGTGCTGCTGGAGTTGGCTCAGAACCGCAACAAGATACCATTGCCAAAGACTATAGCAGGACCC
GGTATCCCACTTCCGCCTGACCAAGACACATTAATCAGTCCTAACTACCAGTTTGGAATTCGAAACAAAAGGCCTGCTGAAC
CTTTAGAAGAAACAGAGGATGAAGAAACTACCATTCCCAATCCCACTCAGGAAGACAAGGTAGAGCTGCAGCAGAATCCCC
ATCAGAGAGTTTCGTTTCCCCTGCCCAAACGCCAAAAGGATTGA

>VrTAF10

ATGAATCAGAACCCGCAATCGAGTGATGGAAGAGGCGACGATGACACTGCTCTCTCCGATTTCCTTGCGTCTTTGATGGATT
ACACTCCCACTATACCTGATGAATTGGTGGAGCATTACTTGGCCAAGAGCGGTTTTCAGTGTCCTGACGTTCGATTGACTAGA

TTGGTAGCTGTTGCCACTCAAAAGTTTGTTGCTGAAGTTGCAGGAGATGCACTTCAGCACTGTAAAGCAAGACAAGCAACA
ATTCCAAAAGACAAAAGGGACAAGCAGCAAAAGGATAAACGTTTAGTTTTGACCATGGAAGACCTATCGAAAGCATTGCGT
GAGTATGGCGTGAATCTAAGGCATCAAGAATATTTTGCCGACAGCCCTTCTACTGGAATGGATCCTGCTACTCGAGAAGAAT
GA

>VrTAF11

ATGAAGCAATCGAAGGATCCGTTCGAAGCAGCGTTCGAGGAATCGCCGCCAGAATCTCCGACGGAGATAGAGGCGGACGC
AATCCATAACGATAGCCATAACCATAATCATAACCAAAACCCTATTCCTTCTCCTTCTGTTCTACCCCTCAACCCAGGGCCTCC
GCAGAAGGCCAGTGCGGCGGTCAAGAACAAAGACAAAGACAAGGACGATGAAGAGGAGGAAGAAGAAGACAACATGGA
CGTTGAGCTTTCCAAGCTCCCTTCCACCGGCGACCCTCACAAAATGGCCAAGATGCAGACAATTTTATCTCAGTTCAGCGAA
GAGCAGATGAGTAGATACGAGTCCTTCCGGAGGGCCGGGTTTCAGAGAGCTAACATGAAAAGGTTATTGGCTAGTATCACG
GGGACCCAGAAGATTTCAGTGCCAATGACAATTGTAGTATCAGGCATTGCAAAAATGTTTGTTGGTGAAGTTGTTGAAACAG
CTAGAATAGTTATGAAAGAAAGGAAGGAATCTGGACCAATTCGGCCTTGTCATCTGAGAGAAGCATATAGACGGCTAAAACT
TGAAGGAAAAGTCTTTAAGAGATCAGCCTCAAGGCTCTTCCGGTAG

>VrTAF12

ATGGATTCTCAAGCTCCGGCAACCGGCACCACCCCCCGGAGTGCGGCTGAACCCTCACAATCTCAACCACCAAAGTCATCT
CCTCCTATACTTCCATCTTCCACATCATCAACTCCTCCAGTCTCCGCCCCAACACAGTCTTCCCCAAACCCTAACCCTAGCTC
AAGCCCAAACCCAAGCCCAATTCAAACCCCCAACCCCAAACCCCCCACTCCTACGCCTGCCCAACCTCGCCCCACGCAATC
CTTCAACCGCACTTTGCCGCCGTCGCAGCCTCAGTTCCCGCACTTCTCTTCCGCTCCTTCGCCTCCCTCCGCTCCTGGCGGC
GCCGCTGCGCCGAGGGGTGGTATGGCAATCGGCGTGCCCGCGCACCACCAGAGCCCCTCTCCTCCGTTTTCATCGTCCTTCG
GTCAGCACTTTGGTGGGCTGGGCCGAACTGGAGTCAGTGTGGCTGAACCTACCTCCAATTCAAGCACCTCTCAAGTGAGAA
CGCCCGTGCAAGGAATGGGGATGTTAGGGCCTCAAATGAGGCCTAGTGGAATCGCTGCTCATCAACAGAGGCCCGTGCAAT
CGTCTCTTAGACCTCCTTCCTCTGCCCCGAATACTCAACCTGGGGGTTCACAAAGCTTCCAAGGTCATGGAATTATGAGACCT
TCATCTGTGGCATCACCGGCTACTCCATCTCAAGGTGCTTCACAAAGTGTACAGTCACTCAATCAGCCATGGTTGTCATCTGG
GCCACTAGGGAAGCCTCCTTTGCCATCCACTGCTTATAGGCAGCAATTGAACCCATCATCAATGCAGCAGAGGTCACATATTC
CTCCACAGCAGCAATCCACACCTACATCTTCACAGCAGCAACAACAACAACAACAACCACCTTTGTCTAATCAATCACAAG
AGCATTTTGGGCAACAGGTTCAGCCATCCAGGGCTTCTCATCACGTACCTCACCAGCAACAGGTTACAAGACTACAGGGGC
CAGGAAATCAGAAACCTTCATCTCTTGTGGTGGCGCAAACCAGTGTAGTTCAGCCAGTGAGTCAAAGTAGACTGACAAATG
CAGATACCGAGGAACCCTGTAATAGTATTCTCAGCAAAAGAAGTATCCATGAGCTGGTCAATCAGGTGGATCCATTGGAGAA
GTTGGATCATGAAGTTGCAGATATTCTTGTGGATATCGCAGAAAATTTTCTAGAGTCTATAATCAGGTCTGGTTGTTCATTGGC
CAAGCATAGGAAATCAACAACTTTGGAGGCGAAGGACATACTTCTCCATCTTGAGAAAAATTGGAACATGACACTTCCTGGA
TTTGGCGGTGATGAGATTAAAAGCTACAGAAAACAGATTACAAGTGATATTCACAAGGAGCGTCTATCAGCTATAAAGAAAT
CAGTGGCAGCAACTGAGGCGGCACATGCTAAGGGCGCTGGCCAGGCTTCTGGTAGTGCAAAGGGTAATCAGGCAAAGACA
CCTATGAATATCATTGGCTCCCCAAACCTAAAAAGTTCATAG

>VrTAF12b

ATGGAAGTGATCGAAAAGAAACCAAACAGCGCCGCAGAAAGTGAGATACGCGAAGAATGTAAGGTTGGAGTGAGAAGCGT
AACCATGGCGCTTACTTTCAGAACCCTCTTTCTCATGCTACTCGCAATCCTCTCTCTTCTCCTTTTCGAGGACTGGGTGTCAA
CGCCTTCTTGCAACAACACCGACAACATCATCGACGACAACCTTAAAGTCATGATGGTCGCTGACCTCCTTCTCTCCGATTC
CGGTTTCGTCAACCGCTTTTTCCGAGACTACTACATGTCCAAATTCTTTCGGAAATCGTTCGAGGTGCTGCGGCCCGATTTGC
TTCTGGTGTTGGGCGACGTTTCGGCGCGAGGCTCGGAGCTAACGAGGAGCAAGTGGGTGTCGGTGCTGCGGAGGTTTTACA
GAGTGTTGGGCCCTTTTGTTGGGCTTCCGTTCCACGCTGTTCTCGGCGACAGAGACGTCGGAGAGTGTGGGGACGTTGATG
TTGATAGGGTTAGTTGGATTGCGAGCAAGTTTCCTGGGTTGGATTCTTCTGGCTGCGCTGCGTTTGAGATTGGGAATGTGAGT
TTTGTCACGCTGAACGCGGTGGCTCTGCTTTGCGGGGGGAGTGGTGGGTTGCGTTTTGATGTGGAGAAGGTGATAGAAAGG
GAGAGTGTGGAGGTTCACATGGGAATGGAGAGGGTGGTGAAGAGAGTCAATGGTTTTGGGGAGTTTGCAGATGCGGATGTT
TTATCTGGGTCAGGGCCTGTCGTTTTGCTTCACCTGCCGTTGGATCAAACAAGGAATGAACACTTTGGTTCGGTTGGTGATTT
TGAGAAATATTGGACTTCTTCCATGGAGGGGTTAAATGTGGTGCCAGAAAGCAGGGGTGGCCAGTACAAGTTGCTTCATATG
CTGCCTCCGAATGCTTCTGAGTACATTTTACAAGCTCTGAAACCAAGGATTATTTTCAGTGCTCACAGGTATACATTTTCTGAT
CACGTTCATGGGGACAGAACTCGTGAGATTAGTGTTCCAGCAATGTCATGGAACGCAAGAGATGACCCTGGATTCGTGATTG
CCTCTTTCCAAAAGGCAGGAAGAGCAGTGAGCATAAGCCATTGTTCTCTTGCTAGGGAATCTCAAATTGTTCTAGTCTACATT
TCTGTAATGTTTTTGTTCTGTTTGGTGTGTTTAAAAGGATATTCTGAGTCTGCTACTGGTATTCATGCTGATTCTATTAACAGCA
AGGAAAAAAGTATAATTGATGGACCTTGCTACCAGTTGGAGGACAACAGATTGTGTTGTTCGAAAGCAAGGTTCTTGACGC
AGATCGATTCTCCGGCCTTCCGCCTTCCTTCAATTCAACTCAGCTCTCTGCGCCTTCCCCTGCGCGCCCATGGCCGCATCATA
GCACCGATTGATCAATCGATCACATCAAAATCAATGGCGGAAAGCGCATCACCGTCGTCCAAACCCTCACCAATGGACCCTC
AGAACCCCGCACCATCCTCCAACCCTACAATCCCTTCCCCTTCCCACAACAACATGCCTTCACCTTCGCTCCCTCCGCTTCCC
CAAGACCAGCAACAGCAACAGCAGCAGCAGCAGCAAAATCTTCATCAACAGTTGAGTCCGCCTCAACAACAACAGCCTCT
CGTGTCGTCGCAAGCCATGAACACCATCAACGGCATCAACCCTATCTCCAATTTCCAACTCCAGCAGACCTTGCAGCGATCG
CCTTCCATGTCGCGCCTCAATCAAATCCAGCCTCAGCAGCAGGCGCAGCAGCAGCAGCAGTTCGGCGTCATGCGCCAACAG
GCCGGATTGTACGGCGGACAGATGAGCTTTGCTGCCGCGGGCGGCGGAGCGGGCCAGCAGCAGCAGTTGGGCGGCTCCAA
TTTGTCGCGGTCCGCGCTGATAGGGCAGAGCGGACACTTCCCCATGTTGTCTGGTGCTGGCTCGCAGTTTAACTTGCTCTCC
TCGCCAAGGCAAAAAGGTGGGCTAGTTCAGCAGTCTCAGTTCTCAGGTAATTCTGCTGGACAGTCTCTGCAAGGAATGCAA
GCGATGGGTATGATTGGAACGCCAAATCTCCCCTCTCAACTGCGAGCTAATGGAGCCCTTGCTTATGCACAGCAGCTGCGAA
TGGGTCACGGTCAAATCAGGCAGCAGATGTCTCAGCAAAGTTCACTTAACACTGGACAGGTTCAAGGTTTACCAAGGTCAT
CATCCCTTGCTTTTATGAACTCTCAGTTGTCTGGGTTGTCGCAGAATGGGCAACCAGGGATGGTTCATAACTCCTTAACACAG
CAACAGTGGCTTAAGCAAATGCCAGCAATGTCTGGCCCTGCCTCACCATTGCGTCTTCAACAGCAGCAGAGGCAGTCGCTG
GCTTCTTCTACTCAATTGCAACAAAACTCTATGAGCCTGAACCAGCAACAATTGTCCCAGCTGATTCAGCAACAGAAGTCGA
TGGGGCAGTCTCAGCTGCATCAGCAGCAACAGCAGCAGCAGCCTCAACAACAGCTGCAACAACAGCTCTTACATCAGCAG
TCACAACAACAGTCTCATCCACAGGCTTCTGTCCATCAACAGCAACAGTCTCCAAGGATGCCAGGAACTGCAGGCCAAAAG
TCATTCAGTCTAACAGGATCACAGCCAGACGCTACTGCATCTGGTGCAACTACACCAGGTGGTAGTTCTAGCCAAGGAACA
GAAGCAACAAACCAAGTTCTTGGGAAGAGAAAGATACAGGATTTAGTTGCACAGGTGGATCCACAAGGTACACTGGACCC
CGAAGTTATAGATCTTCTTTTAGAGCTTGCTGATGACTTCATTGACTCTACAACTACACACGGTTGCATTTTGGCAAAACATA
GAAAATCATCAACTTTGGAGTCCAAGGATTTATTGCTACACCTAGAGAAAAATTGGGATTTAACAATTCCTGGATATTCAAGT
GAAGAGAAGAAGTATCAAAGCAAACCTCAATTAAATGACCTTCACAAGAGGCGCCTAGATATGATTCGCACAATGATGGAAT
CTTCGGCCTCTGAGTCAAATATTAATAGTTCTAAAGAGTTGAGTAGACAGGGCATTTCAAATCCTACCCCTATGGGAGCTCAC
CATCTAGTAAGACCCATGAGTTCAGAGCAGTTGGTTTCTCAGGCAGCTGGTTCTCAAATGCTACAGCAGATGACAAGGTTTT
AA

>VrTAF13

ATGAGCAGCTCTGCTGCTGGAACCTCATCAAAACCAAGAGCAGCTTCCTCACAACCATCAGAAACTTCATCCAAGCGCAAA
AGAGGAGTTTTCCAAAAAGAATTGCAGCACATGATGTATGGCTTTGGAGATGATCCTAATCCGCTTCCTGAAAGTGTGGCTC
TTATGGAGGACATTGTTGTGGAATATGTCACAGAACTGGTTCATAAAGCCCAAGATATCGGATCTCAAAGAGGGAAGCTATC
AGTTGAGGATTTTCTCTATTTGATTCGCAAGGATTTGCCGAAACTTAACCGCTGTACGGAACTACTGTCTATGAATGAAGAAC
TGAAACAAGCAAGAAAGGTTTTTGAGTCAGATGAAGAGAAACTGAGGAAGGTTTTTGAGGTGGACGAGGCCGTGGAAGG
ATGA

>VrTAF14b-1

ATGTCTCACTCTCAACCGTTACCCCTCAAGAGACAAGGTGAGCATTTGTCCGATGATGGTGCTTCTGCCATTAAACCGTCTCG
CCTCAAAATCGCAATACCATCTGAGGATTCCGACAAGAAGAATGCGAACAATAGGATCAAAGATGTTGAAATTTGTGTCCCG
ATAGTGTATGGGACCATTGCATTCTATCTTGGCAGGAAGGCCAGTGAGTCTCAGTCACACAAGTGGACAGTCTATGTACGCG
GAGCTTCGAATGAAGATCTTGGGGTGGTGATTAAGCGGGTTGTGTTTCAATTACATCCTAGTTTTAATAACCCCACTAGAGTT
GTGGAGTCGCCACCGTTTGAGTTGTCAGAAAGTGGTTGGGGTGAATTTGAAATAGCCATCACTCTCTATTTCCACAGTGATG
TCTGTGAAAAACAGTTGGACTTGTATCACCATTTGAAATTATATCCAGAAGATGAATCTGGCCCCCAGTCTACAAAAAAACC
TGTAGTTGTTGAATCTTATAATGAGATTGTTTTTCCTGAACCCTCTGAGGGTTTTCTTGCACGTATACAGAATCATCCTGCTGT
TAATGTGCCTAGGCTTCCTGCTGGTTTGAATTTACCAAGTCCTGTACCAATTGATACTATGAGTGACAAGGAGAGAGGTGAC
ACCAAAGATCATTCTCTGAACCAGTGGTTCTTGAACTTCTCTGAGGCGGATGAGCTCTTAAAACTTGCAGCAGCACGCCAGC
AGGTGCAAGCTCACATTGTTAAGCTGCGAAGACAATTAAGTTTGGTGGAAGGGCTACCTCAGCTGTCAAAACCACCCTCTG
GTTATGAATGTACATGA

>VrTAF14b-2

ATGACCATCAGCTCATCTTCGAAAAAGCACGGTCAAGATCAGCCAGAATTAAGTGGTCCTACCCCCAAATCCCAGCGAACC
AAAATGGGCAAATCTGATGACAACGATAAGAAGAACCTGGGTAAGAAACTCAAAGATGTGGAAATAAGCGTTCCAATAGTG
TATGGAAACATAGCATTTTGGCTTGGGAAAAAGGCTAGCGAGTATCAGTCGCATAAATGGACAGTATATGTTCGTGGAGCAA
CCAATGAGGATCTTGGGGCGATAATAAAACGTGTTGTTTTTCAGTTGCATTCCAGTTTCAATAATCCCACCCGTGTTGTGGAG
TCTCCACCTTTTGAACTCTCAGAGGCAGGATGGGGGGAATTTGAAGTTGCCATCACACTTTATTTTCACAGTGATGTTTGTGA
CAAGCCTTTGAACTTATATCATCACTTGAAGTTGTATCCAGAGGATGAAAACAGTTCCATGTCTGCAAAGAAGCCTGTTGTTG
TGGAGTTTTATGATGAGATAGTTTTCCCTGATCCTTCGGAGGCCTTTTTAGCTCGTTTGCAGAGTCATCCAGCTGTGAACTTA
CCAAGATTACCTGCTGGGCTTACATTGCCTCCTCCCGTACCTGTTGAGGATGCAAGTAAAAGGAGGAAAGGTGACACTAAA
GATCATTCTTTAAGTCAATGGTTCACGAATTTCTCAGAAGCAGATGAGCTCTTACAGCTTGCGGCAGCTCGACAGCAGGTTC
AAGCACATATTGCTAAACTCAGACGACAGATAAACTTGATAGATGGGCAGCATCAGCAGTTTAAATCTTCTTCTGACCAGTA
G

>VrTAF15-1

ATGGCAACCCATCCTGGAAAACAAGCTCTTTCAAATGGGTCTGTCTATGTATGCAACTTGCCCTACGGGACTGACGAAAATA
TGTTGGCTGAATATTTTGGCACCATTGGACTAGTGAAGAAAGATAAACGTACCGGGAGACCAAAAATATGGTTATATCGAGA
CAAAGAGACTAATGAACCAAAGGGAGATGCTACTGTGACGTATGAAGATCCACATGCTGCTATAGCGGCTGTTGAATGGTTT
AACAACAAAGATTTTCATGGCAATATAATTGGCGTTTTTATAGCAGAGTCAAAAAACAAGGATGACCAAACTTATAATTCAGC
AGGAGTAGAACCAGTAGTTGCTGGTACTGTTGTTGGATTAGAGGAAACTACCAAGGATGTTAATGGTGGTAGTGGAAGAGG
TAGAGGACAAAATGATCCTTCAGGCAAAGCATGGCAACAAGATGGCGATTGGTTGTGTCCGAATACAAGTTGCTCCAATGTG
AACTTTGCTTTCCGTGGTGCCTGTAACCGCTGTGGAACTGCCCGTCCTGCTGGTGCTGCTGGAATTTCAGGGGCTGGTGGCC
GTGGCAAAGGACGTGCTGCTGGACAAGAACCAGGAGGTGTTGGCCGGCCAGTTGGTGGAGGACTATTTGGTCCCAATGATT
GGCCCTGTCCAATGTGTGGCAATATCAACTGGGCGAAGCGGACTAAATGTAATATTTGCAATACAAATAAGCCTGGGCATAAT
GAGGGTGGTGTAAGAGGAGGACGAGGTGGAGGTTACAAAGAGCTTGATGAAGAAGAAATAGAAGAAACCAGACGTCGTA
GGAGGGAAGCTGAAGATGATGGGGAGTTGTATGATGAATTTGGAAATCTAAAGAAAAAATTCCGTGCCAAAACACAGCAAG
CTGAAGCTGCGCGTGGGCTTCCTGGTTCAGGACGTGCTGGTTGGGAGGTTGAAGAACTAGGGATAGACAAGGATGGTAGAG
AAAGTAGAGACAGAGGAAGAGAACGTAATGATGGGGAGAGCAGGAGCAGAGAGCGAAGTGACAAAGAGAGGCAAAGCA
GTTGGAATAGGGACAGAGACAGGGACAGGGGAAGAGATCGAGACCGAGACTGGGACTATGTTGACCGAGACAGAGATTAT
GGACGAGATCGGGACCGTAGTAGACACAGATATTAA

>VrTAF15-2

ATGAGCAGACCAGGAGATTGGAACTGCAGGACATGCAACCACCTCAACTTCCAGAGAAGAGAATCGTGCCAGCGATGCGG
GGAGCCAAGGAGTGCCGGCGACTACGGCGGCGCCTTCGGAGGCAGAGGCTCTTCCTCCTTTGGCTTCACCACTGGCCCTGA
TGTTCGCCCCGGTGACTGGTACTGCACCGTCGGAAACTGTGGAGCCCACAACTTCGCCAGCCGCTCCAGCTGCTTCAAGTG
CGGTGTCTCCAAGGAGGACTCCTCCACCGGATCATACGACCTCGACATCACCCGAATGAGACCCTACGGCTTCGGCAGCGG
CTCCTCCGCCCGACCCGGCTGGAAATCCGGTGACTGGATATGCACCAGGTCTGGATGCAACGAGCATAACTTCGCCAATAGA
ATGGAGTGCTACAGATGCAATGCACCCAGGGACTCTAATAGTGGCAGACCTCCCTATTCATCGTAG

>VrTAF15b-1

ATGCTACGTTGTACTTGTACAACTTCTCTTCCTCTCTTTACCGTCTCTACAATTCCTTATCGGATTCCAATCATGTCTGGGAATT
ACGACCAAGATGGTGGCGGATATGGGCGTAACGACGGCGGTGGTTATGGTGGTCGGGGAGGTGGAGGATTCGGCGGACGA
GGTGGGTATCAAGGCGGGGATCGTGGTGGTAGAGGTGGTGGCCGCGGCGGCGGAAGTGGTAGAGACGGTGACTGGCGTTG
CCCTAACTCAAGTTGTGGGAATTTGAACTTTGCGAGAAGGGTTGAATGTAACAAATGTGGTGCTCCTTCTCCTGCTGGTGCT
AATGATCGTGGTGGAGGTGGTTATAATAGAGGGGGATATGGCAACAGTCGTGGGGGTAGATCTGGTAACTATGATGGAGGAA
GAGGTAATGGTTATAATAGTAGTAGGGGGAATAACAATGTTGGAAGAAGTGGGGGAGGCAACAGAGGTAGTCAAGGCAGAG
AAGATGGTGGCTATGGTCAAGTTCCTGCACCTGCAGCCCAATCTTATGGTGGGGCTGGTGGAAACTATCCACCTGCGTACAA
TTCTTCTGGTGGGAGTTCAAATTATGAAACTGATGCAGTTCCTCCGCCTGCTAGCTATACTGGTGGACCTGCATCTCACCCTC
CACCATATGGGAGTAATACTGGTGGTTATGGTGGCGGAGATGCACATAATGGTGGTAGGTCTGGGCAACCGGTTGGATATGAT
AGTGGTTATGGTGCCGGTAGTCAAGGTGGATTCGGTGGAGCTCCTGATGAGCCCCCTGCTAAGGTGAAGCAATGTGATGAG
AATTGTGGGGATTCCTGTGACAACTCTAGAATCTACATATCAAACTTACCTCCGGATGTGACTATTGAAGAATTGAGAGAGCT
TTTTGGAGGCATTGGACAAGTTGGAAGAATAAAACAAAAGAGGGGGTACAAAGATCAGTGGCCTTGGAACATAAAGCTATA
CACTGATGAGAAAGGAAACAACAAGGGTGATGGTTGTCTTGTGTATGAAGACCCTTCCGCAGCACATTCTGCTGGCGGTTTT
TACAATAATTATGATTTGAGGGGTTACAAAATCGCTGTTACAATGGCTGAAAAGTCTGCACCAAAAGCCCCACCTGCATATAA
CCATGGGGGCAACAGGGGTGGCTATGGTGGAGATAGACGCAGAGACAATTATAGAGATGCAGGTGGTTCTGGTCCAGATAG

GCGAGACAATTACGGTGGGAATCGTTCACGACCATACTGA

>VrTAF15b-2

ATGTCTGGAGCATACGGTCAAGACGGCGGCGGCGGCTCTGCGCCACCGTCATATGGAGCCAGCGGTGGATACGGTTCCGGT
GGTGGTAGTTATGGAGGCGGAACCTATGGAGGGGGCGGTGCCGGTGGAGGATACGGTGGTAACGATGGCGGTGGAGGTTAC
GGAGGGAAAAACAGCGGAAACGGCGGCGGATACGGTGGTAACGATGGCGGGGGTTATGGAGGAAGGAGCGGCTACGGTG
GAAACGATGGCGGAGGCTATGGAGGAAGAGGCGGTGGTCAAGGTGGCCGGGGAGGTGGAGGATTCGGTGGTGGATATGGT
GGTCGAGGTGGAGGTGGAGGTGGAGGTGGAGGTGGCGGTGGCGGTGGCGGATATCAAGGTGGTGATCGCGGTGGTCGTGG
CGGAGGTCGCGGCGGAGGCGGCCGTGGCGGCAGCGGCAGAGATGGAGACTGGCGTTGCCCTAACCAAAGTTGTGGGAATT
TGAACTTTGCGAGAAGGGTTGAATGTAACAAATGCGGTGCTCCTTGTCCTAATCCGAATAGTTCCAATGAACGTGGTGGAAG
TGGTGGGGGTGGGGGTGGCTTTAGTAGAGGCGGAGGTGGCGGTGGGGGGTATGGTAACACGCGAGGGGGAAGATCTGGGA
ACTATGATGGAGGGAGAGGTAATGACTATAACAGTGGAGGGGGTCGATCTGGTAACTATGATGGTGGAAGGGGTAATGATTA
TAATAGTGGAAGGGGTGGTAGTAATGATGGTAGAGGTGGTTCATATAGAGGTAATCAAGGTAGAGAAGATGGTAGCTACGGT
CAAGTTCCCGCTCCTAATGCCCAATCTTATGGTAGTGCTGGTGGAAGCTTCCCACCCTCTTACAGTTCTTATGGTGGGAATGC
AAGTTATGGAACTGATGCAGTTCCTCCACCTTCAAGCTATACTGGCGGACCTAATTCCTATCCTCCATCGTATGGGGGTAATGT
CGGAGGTTATGGGGGAGATAATCAAGGGAATGGACGGAGTGGTGGTAGATCTGGGCCTCCTTCTGGGTTTGACAATAGTTAT
GGTGCTGGTAATCGAGGTGGATTTGGTGGGTCTCCTGCCGAGCCCCCAGCTGCAGTGAAGCAGTGTGATGAGAATTGTGAT
GACACTTGCGACAACTCTAGAATATACATCTCAAACCTTCCACCAGATGTGTCAATTGAAGAATTGAGGGAACTTTTTGGAG
GCATTGGACAAGTTGGAAGGATAAAGCAGAAGAGAGGCTACAAAGATCAGTGGCCTTGGAACATTAAGATTTACACAGATG
AGAATGGAAATAACAAGGGAGATGCTTGTCTTGCTTATGAAGACCCTTCTGCAGCACATTCGGCTGGCGGTTTTTACAATAAT
TATGATTTGAGGGGTTACAAAATCAGTGTTGCAATGGCAGAAAAATCTGCTCCAAGAGCTGCACCTGCTTATAACCAAGGGG
GCAATAGGGGTGGCTATGGTGGAGATAGACGCCGAGACAACTATAGAGATGGAGGTGGCTCTGGGCCTGATAGGCGTGATCA
TTATGGAAATCGTTCTCGTCCGTACTAG

3. Protein sequences of VrTBP and VrTAFs

>VrTBP

MADQGLEGSQPVDLQKHPSGIVPTLQNIVSTVNLDCKLDLKTIALQARNAEYNPKRFAAVIMRIREPKTTALIFASGKMVCTGAK
SEQQSKLAARKYARIIQKLGFPAKFKDFKIQNIVGSCDVKFPIRLEGLAYSHGAFSSYEPELFPGLIYRMKQPKIVLLIFVSGKIVLT
GAKVRDETYTAFENIYPVLTEFRKNQQ

>VrTAF1-X1

MGYDSASPSQDGRDEDDEEEYEESGKGNRFLGFMFGNVDNSGDLDVDYLDEDAKEHLSALADKLGPSLTDIDLSGKSPQTPPD
VVEQDCDEKAEDAVDYEDIDEEYDGPETEAANEEDYLLPKKEFFSAEASVCMESKASVFDDENYDEESEKEQDFLNEDSKPDNI
SLPEEQEETLVEASKEESALERELHVDSLQSEELDADVQKPEEEGTEVQKRSMAMPLPILCVEDGVAILRFSEIFGIHEPLRKGEKR
EHRQPITRDRYKSLDFTDDFVEEDEEEFLKGSSQSLSQTKQVSVVHNDVSESNDVDLEFPKFGFLHTEPSVARKDDHQSKDSCHS
AEPMKGDFEEDLSWKDHPFIWTNFYPLDQQDWEDEIIWGNSPVPSNNNIESCEVSGPELGVSGGSEIEIESGIQNIQLEHHKILED
KDHNVLLSSPVSLEAFGSRDSSEAKTNLISRSLFHPQLLRLESRSEVDSSSLPDGKEGEISKHNQSGQITRFSKAISQNRDMVEGSW
LDEIIWEELDQPMVKPKLIFDLQDDQMHFEVLDSKDGAHLRLHAGAIILTRSSKSSSGDSSEVPGHGSQYGWRYVSNDKHYSNR
KTSQQLKSNSKKRSAHGVKVFHSQPALKLQTMKLKLSNKDIANFHRPKALWYPHDNEVAVKEQGKLPTQGPMKIIIKSLGGKGS
KLHVDTEETLSTVKAKASKKLDFKASETVKIFYLGRELEDQKSLAEQNVQPNSLLHLVRSKIHLWPKAQRVPGENKSLRPPGAF
KKKSDLSVKDGHVFLMEYCEERPLLLSNVGMGARLCTYYQKCSPDDQSGSLLRNTDSSLGHVISLDPADKSPFLGDLKPGCSQS
SLETNMYRAPVFPHKVPLTDYLLVRSPKGKLSLRRIDKINVVGQQEPLMEVFSPGSKNLQTYMMNRLLVHMCREFQAAEKRHL
PPHIRVDEFLSQFPYQSEASFRKKIKEYANLQRGANGQSILVKKRNFRMWSEDELRKMVLPELVCAYESMQAGLYRLKHLGITET
HPTNISSAMSRLPDEAIALAAASHIERELQITPWNLSSNFVACTSQGKENIERMEITGVGDPSGRGMGFSYARAPPKAPVSSAMVK
KKAAANRGGSTVTGTDADLRRLSMEAAREVLLKFNVPEEVITKQTRWHRIAMIRKLSSEQAASGVKVDPTTISKYARGQRMSFL
QLQQQTREKCQEIWDRQVQSLSAVNADENESDSEGNSDLDSFAGDLENLLDAEEFEEGEEGTNDLKRDKGDGVKGLKMRRRST
LAQAEEEIEDEAAEAAELCRLLMDDDEADRKKKKKTKVTGEETRLVSKMQSKFAFDNAEQVKQITNSLQLDGNIPLKEDTITDL
REEENFGAKKSKSLKVNKAKKNDIAPISLPNKKIKLNMGEGIKNQVFKEKKPSRETFVCGACGQPGHMRTNKNCPKYGEDLET
QLESADMEKSSGKPISVDPSSHSQPKTASKKSSSKSNSKITPVDNSAKIPLKFKCGSTEKSSDKPVTETLQNSDKPVTSDSETAKSA
KVNKIIIPKKVKPDDTQAESRKHAVVIRPPTESSRGLPADSGRGPPTDAGRGQVDYQKLPIKIRPPTEEQSHKKIVIRRTKEVIDLEL
DSPGGNTGLQHRKTKRIVELSNFEKHKQETVYGTGAFPKWNTKEDRRWWEEQEKRRNDARLREEDRARRHHKEEMRMLKEQ
ERLDEIKRFEEDIRREREEEERQKAKKKKKKKKPDLRDEYLDDPRARRHDKRMPERDRSGKRRSVAELGKLSADYMPPTKRRR
GGGGEVGLANILEGIVETMVKDRYELSYLFVKPVSKKEAPDYLDIIDTPMDLSRIRERVRNMEYKSREDFRHDVWQITFNAHKY
NDGRNPGIPPLADMLLEYCDYLLNENDDSLTSAEAGIETRDS

>VrTAF1-X2

MESKASVFDDENYDEESEKEQDFLNEDSKPDNISLPEEQEETLVEASKEESALERELHVDSLQSEELDADVQKPEEEGTEVQKRS
MAMPLPILCVEDGVAILRFSEIFGIHEPLRKGEKREHRQPITRDRYKSLDFTDDFVEEDEEEFLKGSSQSLSQTKQVSVVHNDVSES
NDVDLEFPKFGFLHTEPSVARKDDHQSKDSCHSAEPMKGDFEEDLSWKDHPFIWTNFYPLDQQDWEDEIIWGNSPVPSNNNIES
CEVSGPELGVSGGSEIEIESGIQNIQLEHHKILEDKDHNVLLSSPVSLEAFGSRDSSEAKTNLISRSLFHPQLLRLESRSEVDSSSLPD
GKEGEISKHNQSGQITRFSKAISQNRDMVEGSWLDEIIWEELDQPMVKPKLIFDLQDDQMHFEVLDSKDGAHLRLHAGAIILTRS
SKSSSGDSSEVPGHGSQYGWRYVSNDKHYSNRKTSQQLKSNSKKRSAHGVKVFHSQPALKLQTMKLKLSNKDIANFHRPKAL
WYPHDNEVAVKEQGKLPTQGPMKIIIKSLGGKGSKLHVDTEETLSTVKAKASKKLDFKASETVKIFYLGRELEDQKSLAEQNVQ
PNSLLHLVRSKIHLWPKAQRVPGENKSLRPPGAFKKKSDLSVKDGHVFLMEYCEERPLLLSNVGMGARLCTYYQKCSPDDQSGS
LLRNTDSSLGHVISLDPADKSPFLGDLKPGCSQSSLETNMYRAPVFPHKVPLTDYLLVRSPKGKLSLRRIDKINVVGQQEPLMEVF
SPGSKNLQTYMMNRLLVHMCREFQAAEKRHLPPHIRVDEFLSQFPYQSEASFRKKIKEYANLQRGANGQSILVKKRNFRMWSED
ELRKMVLPELVCAYESMQAGLYRLKHLGITETHPTNISSAMSRLPDEAIALAAASHIERELQITPWNLSSNFVACTSQGKENIERM
EITGVGDPSGRGMGFSYARAPPKAPVSSAMVKKKAAANRGGSTVTGTDADLRRLSMEAAREVLLKFNVPEEVITKQTRWHRIA
MIRKLSSEQAASGVKVDPTTISKYARGQRMSFLQLQQQTREKCQEIWDRQVQSLSAVNADENESDSEGNSDLDSFAGDLENLLD
AEEFEEGEEGTNDLKRDKGDGVKGLKMRRRSTLAQAEEEIEDEAAEAAELCRLLMDDDEADRKKKKKTKVTGEETRLVSKMQ
SKFAFDNAEQVKQITNSLQLDGNIPLKEDTITDLREEENFGAKKSKSLKVNKAKKNDIAPISLPNKKIKLNMGEGIKNQVFKEKKP
SRETFVCGACGQPGHMRTNKNCPKYGEDLETQLESADMEKSSGKPISVDPSSHSQPKTASKKSSSKSNSKITPVDNSAKIPLKFKC
GSTEKSSDKPVTETLQNSDKPVTSDSETAKSAKVNKIIIPKKVKPDDTQAESRKHAVVIRPPTESSRGLPADSGRGPPTDAGRGQV
DYQKLPIKIRPPTEEQSHKKIVIRRTKEVIDLELDSPGGNTGLQHRKTKRIVELSNFEKHKQETVYGTGAFPKWNTKEDRRWWEE
QEKRRNDARLREEDRARRHHKEEMRMLKEQERLDEIKRFEEDIRREREEEERQKAKKKKKKKKPDLRDEYLDDPRARRHDKR
MPERDRSGKRRSVAELGKLSADYMPPTKRRRGGGGEVGLANILEGIVETMVKDRYELSYLFVKPVSKKEAPDYLDIIDTPMDLS

RIRERVRNMEYKSREDFRHDVWQITFNAHKYNDGRNPGIPPLADMLLEYCDYLLNENDDSLTSAEAGIETRDS

>VrTAF1-X3

MKILNLIISPYLFTYASFVLSEEQEETLVEASKEESALERELHVDSLQSEELDADVQKPEEEGTEVQKRSMAMPLPILCVEDGVAIL
RFSEIFGIHEPLRKGEKREHRQPITRDRYKSLDFTDDFVEEDEEEFLKGSSQSLSQTKQVSVVHNDVSESNDVDLEFPKFGFLHTEP
SVARKDDHQSKDSCHSAEPMKGDFEEDLSWKDHPFIWTNFYPLDQQDWEDEIIWGNSPVPSNNNIESCEVSGPELGVSGGSEIEI
ESGIQNIQLEHHKILEDKDHNVLLSSPVSLEAFGSRDSSEAKTNLISRSLFHPQLLRLESRSEVDSSSLPDGKEGEISKHNQSGQITR
FSKAISQNRDMVEGSWLDEIIWEELDQPMVKPKLIFDLQDDQMHFEVLDSKDGAHLRLHAGAIILTRSSKSSSGDSSEVPGHGSQ
YGWRYVSNDKHYSNRKTSQQLKSNSKKRSAHGVKVFHSQPALKLQTMKLKLSNKDIANFHRPKALWYPHDNEVAVKEQGKLP
TQGPMKIIIKSLGGKGSKLHVDTEETLSTVKAKASKKLDFKASETVKIFYLGRELEDQKSLAEQNVQPNSLLHLVRSKIHLWPKA
QRVPGENKSLRPPGAFKKKSDLSVKDGHVFLMEYCEERPLLLSNVGMGARLCTYYQKCSPDDQSGSLLRNTDSSLGHVISLDPA
DKSPFLGDLKPGCSQSSLETNMYRAPVFPHKVPLTDYLLVRSPKGKLSLRRIDKINVVGQQEPLMEVFSPGSKNLQTYMMNRLL
VHMCREFQAAEKRHLPPHIRVDEFLSQFPYQSEASFRKKIKEYANLQRGANGQSILVKKRNFRMWSEDELRKMVLPELVCAYES
MQAGLYRLKHLGITETHPTNISSAMSRLPDEAIALAAASHIERELQITPWNLSSNFVACTSQGKENIERMEITGVGDPSGRGMGFS
YARAPPKAPVSSAMVKKKAAANRGGSTVTGTDADLRRLSMEAAREVLLKFNVPEEVITKQTRWHRIAMIRKLSSEQAASGVKV
DPTTISKYARGQRMSFLQLQQQTREKCQEIWDRQVQSLSAVNADENESDSEGNSDLDSFAGDLENLLDAEEFEEGEEGTNDLKR
DKGDGVKGLKMRRRSTLAQAEEEIEDEAAEAAELCRLLMDDDEADRKKKKKTKVTGEETRLVSKMQSKFAFDNAEQVKQITN
SLQLDGNIPLKEDTITDLREEENFGAKKSKSLKVNKAKKNDIAPISLPNKKIKLNMGEGIKNQVFKEKKPSRETFVCGACGQPGH
MRTNKNCPKYGEDLETQLESADMEKSSGKPISVDPSSHSQPKTASKKSSSKSNSKITPVDNSAKIPLKFKCGSTEKSSDKPVTETL
QNSDKPVTSDSETAKSAKVNKIIIPKKVKPDDTQAESRKHAVVIRPPTESSRGLPADSGRGPPTDAGRGQVDYQKLPIKIRPPTEEQ
SHKKIVIRRTKEVIDLELDSPGGNTGLQHRKTKRIVELSNFEKHKQETVYGTGAFPKWNTKEDRRWWEEQEKRRNDARLREED
RARRHHKEEMRMLKEQERLDEIKRFEEDIRREREEEERQKAKKKKKKKKPDLRDEYLDDPRARRHDKRMPERDRSGKRRSVA
ELGKLSADYMPPTKRRRGGGGEVGLANILEGIVETMVKDRYELSYLFVKPVSKKEAPDYLDIIDTPMDLSRIRERVRNMEYKSR
EDFRHDVWQITFNAHKYNDGRNPGIPPLADMLLEYCDYLLNENDDSLTSAEAGIETRDS

>VrTAF1-X4

MTLPINFTYASFVLSEEQEETLVEASKEESALERELHVDSLQSEELDADVQKPEEEGTEVQKRSMAMPLPILCVEDGVAILRFSEIF
GIHEPLRKGEKREHRQPITRDRYKSLDFTDDFVEEDEEEFLKGSSQSLSQTKQVSVVHNDVSESNDVDLEFPKFGFLHTEPSVARK
DDHQSKDSCHSAEPMKGDFEEDLSWKDHPFIWTNFYPLDQQDWEDEIIWGNSPVPSNNNIESCEVSGPELGVSGGSEIEIESGIQN
IQLEHHKILEDKDHNVLLSSPVSLEAFGSRDSSEAKTNLISRSLFHPQLLRLESRSEVDSSSLPDGKEGEISKHNQSGQITRFSKAIS
QNRDMVEGSWLDEIIWEELDQPMVKPKLIFDLQDDQMHFEVLDSKDGAHLRLHAGAIILTRSSKSSSGDSSEVPGHGSQYGWRY
VSNDKHYSNRKTSQQLKSNSKKRSAHGVKVFHSQPALKLQTMKLKLSNKDIANFHRPKALWYPHDNEVAVKEQGKLPTQGPM
KIIIKSLGGKGSKLHVDTEETLSTVKAKASKKLDFKASETVKIFYLGRELEDQKSLAEQNVQPNSLLHLVRSKIHLWPKAQRVPGE
NKSLRPPGAFKKKSDLSVKDGHVFLMEYCEERPLLLSNVGMGARLCTYYQKCSPDDQSGSLLRNTDSSLGHVISLDPADKSPFL
GDLKPGCSQSSLETNMYRAPVFPHKVPLTDYLLVRSPKGKLSLRRIDKINVVGQQEPLMEVFSPGSKNLQTYMMNRLLVHMCRE
FQAAEKRHLPPHIRVDEFLSQFPYQSEASFRKKIKEYANLQRGANGQSILVKKRNFRMWSEDELRKMVLPELVCAYESMQAGLY
RLKHLGITETHPTNISSAMSRLPDEAIALAAASHIERELQITPWNLSSNFVACTSQGKENIERMEITGVGDPSGRGMGFSYARAPPK
APVSSAMVKKKAAANRGGSTVTGTDADLRRLSMEAAREVLLKFNVPEEVITKQTRWHRIAMIRKLSSEQAASGVKVDPTTISK
YARGQRMSFLQLQQQTREKCQEIWDRQVQSLSAVNADENESDSEGNSDLDSFAGDLENLLDAEEFEEGEEGTNDLKRDKGDGV
KGLKMRRRSTLAQAEEEIEDEAAEAAELCRLLMDDDEADRKKKKKTKVTGEETRLVSKMQSKFAFDNAEQVKQITNSLQLDG
NIPLKEDTITDLREEENFGAKKSKSLKVNKAKKNDIAPISLPNKKIKLNMGEGIKNQVFKEKKPSRETFVCGACGQPGHMRTNKN
CPKYGEDLETQLESADMEKSSGKPISVDPSSHSQPKTASKKSSSKSNSKITPVDNSAKIPLKFKCGSTEKSSDKPVTETLQNSDKP
VTSDSETAKSAKVNKIIIPKKVKPDDTQAESRKHAVVIRPPTESSRGLPADSGRGPPTDAGRGQVDYQKLPIKIRPPTEEQSHKKIVI
RRTKEVIDLELDSPGGNTGLQHRKTKRIVELSNFEKHKQETVYGTGAFPKWNTKEDRRWWEEQEKRRNDARLREEDRARRHH
KEEMRMLKEQERLDEIKRFEEDIRREREEEERQKAKKKKKKKKPDLRDEYLDDPRARRHDKRMPERDRSGKRRSVAELGKLSA
DYMPPTKRRRGGGGEVGLANILEGIVETMVKDRYELSYLFVKPVSKKEAPDYLDIIDTPMDLSRIRERVRNMEYKSREDFRHDV
WQITFNAHKYNDGRNPGIPPLADMLLEYCDYLLNENDDSLTSAEAGIETRDS

>VrTAF2-X1

MAKPRKTKNNEDPKPENSGALVHHQKLCLSIDIDKRLVHGYTELEIAVPEIGIVGLHAENLGIESVWVDGEPTEFEYYPHQQQQV
EDDKRFSSVCSPSSAADAAVSVYMSSLEKELVPNLLINCCKPSKTESEQQQEQTVPENGFHSTAEPKQNVRIVRIDYWIEKAETGI
HFRNNLLHTDNQIRRARCWFPCIDDNSQRCCYDLEFTVAHNLVAVSTGFLLYQVLSKDNPPRKTYVYKLDVPVAARWISLAVAPF
EIFPDHQFSLISHMCLMPNLSKMRNTVEFFHSAFSCYKDYLAVDFPFDSYTQVFIEPEMAVSSLSLGASMSIFSSQVLFDEKVIDQT
IDTRAKLAYALARQWFGVYITPETPNDEWLLDGLAGFLTDFYIKKHLGNNEARYRRYKANCAVCKVDNGGATALSCSASCKDL
YGTQCIGLYGKIRSWKSVAVLQMLEKQMGPESFRRILQTIVSRAQDKTRSMKTLSTKEFRHFANKVGNLERPFLKDFFPRWVGSC
GCPVLRMGFSYNKRKNMVELAVLRGCTALQTSTTSTLDINPETENRDGDTGWPGMMSIRVYELDGMYDHPILPMAGEAWQLLE
IQCHSKLAARRFQKPKKGLKHDGSDDNGDVPSMDVRSNTESPLLWIRADPDMEYLAEVHFNQPVQMWINQLEKDKDVIAQAQ
AIAALEASPQLSFSIVNALNNFLGDSKAFWRVRIEAAFALANSASEETDFSGLLHLVKFYKSRRFDPDIGLPKPNDFHDFAEYFVL
EAIPHAVAMVRAADKKSPREAIEFVLQLLKYNDNNGNPYSDVFWLSALVQSVGELEFGQQSILLLSSLLKRIDRLLQFDSLMPRY
NGILTISCIRTLTQIALKLSGFIPLDRVFELVKPFRDLKTLWQVRIEASRALLDLEFHCKGMDSALLLFIKYLEEEHSLRGQLKLATH
VMRLCQMRDGLNSDEEITSQTLVSMLNLLEGRTAFNNVFLRHYLFCILQILARRPPTLHGIPRENRTLHMSLTEACNYQKNMFVL
DSDSKPLDLPSSTQNPTPNLCLDDLRDALNEASKDPPDEAPVQVHIEALNEAPLEKAEEVYTEFPQEAPMEAPNEVSKEADTVSN
SHERKRLIKIKVKQSSATSRADTDNQVVERSLGGRNEMDHGASSSVSVDAPQRNFAETLSMSNHNIDEVNSWHDRGSRMTASIG
SAKFLSDGDELVKELQCTADSSIVYSQPQPEDPSSSSIIQDNNVDADARRYASLQTLSVARFDPEGESLGKEISARGKEKHKSKEK
KRKRESNKGHHDDPEYLERKRLKKEKKRREKELAKLQSDEAKRSSVDLSSKKEEPVVDVSRQIKSVEPGGGHSSKLETKKIDSK
PDPSEGTSGAPKIRIKIKNRMLNKS

>VrTAF2-X2

MAKPRKTKNNEDPKPENSGALVHHQKLCLSIDIDKRLVHGYTELEIAVPEIGIVGLHAENLGIESVWVDGEPTEFEYYPHQQQQV
EDDKRFSSVCSPSSAADAAVSVYMSSLEKELVPNLLINCCKPSKTESEQQQEQTVPENGFHSTAEPKQNVRIVRIDYWIEKAETGI
HFRNNLLHTDNQIRRARCWFPCIDDNSQRCCYDLEFTVAHNLVAVSTGFLLYQVLSKDNPPRKTYVYKLDVPVAARWISLAVAPF
EIFPDHQFSLISHMCLMPNLSKMRNTVEFFHSAFSCYKDYLAVDFPFDSYTQVFIEPEMAVSSLSLGASMSIFSSQVLFDEKVIDQT
IDTRAKLAYALARQWFGVYITPETPNDEWLLDGLAGFLTDFYIKKHLGNNEARYRRYKANCAVCKVDNGGATALSCSASCKDL
YGTQCIGLYGKIRSWKSVAVLQMLEKQMGPESFRRILQTIVSRAQDKTRSMKTLSTKEFRHFANKVGNLERPFLKDFFPRWVGSC
GCPVLRMGFSYNKRKNMVELAVLRGCTALQTSTTSTLDINPETENRDGDTGWPGMMSIRVYELDGMYDHPILPMAGEAWQLLE
IQCHSKLAARRFQKPKKGLKHDGSDDNGDVPSMDVRSNTESPLLWIRADPDMEYLAEVHFNQPVQMWINQLEKDKDVIAQAQ
AIAALEASPQLSFSIVNALNNFLGDSKAFWRVRIEAAFALANSASEETDFSGLLHLVKFYKSRRFDPDIGLPKPNDFHDFAEYFVL

EAIPHAVAMVRAADKKSPREAIEFVLQLLKSILLLSSLLKRIDRLLQFDSLMPRYNGILTISCIRTLTQIALKLSGFIPLDRVFELVKPF
RDLKTLWQVRIEASRALLDLEFHCKGMDSALLLFIKYLEEEHSLRGQLKLATHVMRLCQMRDGLNSDEEITSQTLVSMLNLLEG
RTAFNNVFLRHYLFCILQILARRPPTLHGIPRENRTLHMSLTEACNYQKNMFVLDSDSKPLDLPSSTQNPTPNLCLDDLRDALNEA
SKDPPDEAPVQVHIEALNEAPLEKAEEVYTEFPQEAPMEAPNEVSKEADTVSNSHERKRLIKIKVKQSSATSRADTDNQVVERSL
GGRNEMDHGASSSVSVDAPQRNFAETLSMSNHNIDEVNSWHDRGSRMTASIGSAKFLSDGDELVKELQCTADSSIVYSQPQPED
PSSSSIIQDNNVDADARRYASLQTLSVARFDPEGESLGKEISARGKEKHKSKEKKRKRESNKGHHDDPEYLERKRLKKEKKRREK
ELAKLQSDEAKRSSVDLSSKKEEPVVDVSRQIKSVEPGGGHSSKLETKKIDSKPDPSEGTSGAPKIRIKIKNRMLNKS

>VrTAF2-X3

MAKPRKTKNNEDPKPENSGALVHHQKLCLSIDIDKRLVHGYTELEIAVPEIGIVGLHAENLGIESVWVDGEPTEFEYYPHQQQQV
EDDKRFSSVCSPSSAADAAVSVYMSSLEKELVPNLLINCCKPSKTESEQQQEQTVPENGFHSTAEPKQNVRIVRIDYWIEKAETGI
HFRNNLLHTDNQIRRARCWFPCIDDNSQRCCYDLEFTVAHNLVAVSTGFLLYQVLSKDNPPRKTYVYKLDVPVAARWISLAVAPF
EIFPDHQFSLISHMCLMPNLSKMRNTVEFFHSAFSCYKDYLAVDFPFDSYTQVFIEPEMAVSSLSLGASMSIFSSQVLFDEKVIDQT
IDTRAKLAYALARQWFGVYITPETPNDEWLLDGLAGFLTDFYIKKHLGNNEARYRRYKANCAVCKVDNGGATALSCSASCKDL
YGTQCIGLYGKIRSWKSVAVLQMLEKQMGPESFRRILQTIVSRAQDKTRSMKTLSTKEFRHFANKVGNLERPFLKDFFPRWVGSC
GCPVLRMGFSYNKRKNMVELAVLRGCTALQTSTTSTLDINPETENRDGDTGWPGMMSIRVYELDGMYDHPILPMAGEAWQLLE
IQCHSKLAARRFQKPKKGLKHDGSDDNGDVPSMDVRSNTESPLLWIRADPDMEYLAEVHFNQPVQMWINQLEKDKDVIAQAQ
AIAALEASPQLSFSIVNALNNFLGDSKAFWRVRIEAAFALANSASEETDFSGLLHLVKFYKSRRFDPDIGLPKPNDFHDFAEYFVL
EAIPHAVAMVRAADKKSPREAIEFVLQLLKYNDNNGNPYSDVFWLSALVQSVGELEFGQQSILLLSSLLKRIDRLLQFDSLMPRY
NGILTISCIRTLTQIALKLSGFIPLDRVFELVKPFRDLKTLWQVRIEASRALLDLEFHCKGMDSALLLFIKYLEEEHSLRGCSSFPSRR
IYR

>VrTAF4b-X1

MDPSIMKLLEDDEDETMHSGVDVEAFQAALNRDIGGDVSASQFPGSDAVLSQGSNNTSSQSSSQWPTSNHDSQSDGQNQEPKT
AQEQHPSEMEPKQHGSLGEHLQHVASQDVNNIHLSQKQSQDDSHQTPAVQVPLHNSQTIGIHNSGKDSVLNKEVVKSHNPSSES
QYAKLQQMSNQQATVSEQPSSQVNRSNKQVPFGLLLPILLPQLAKDRAMQLQTLFTKLKKDEIPKDSFVRLMKGIVGDQMLRL
ALAKVQMQPQARPNQASAGQQLPVRMPTVSSGARQLNDPHALAQMHQRSMNVAVDQSRMSSSAGQTMDSNARKSQEFDVKI
ESQGLQPNQLTSSTSNSVAQETERASVHIQGLNKQQQHHLHFASAYGNSGGNYNPYSGTTGSSTSSIKPQSHDSHMSQIPHQSIGS
NHLGGSTHGLSVIGMPKLEQQNSFNDPKRLPGGSVSSAVNNAASQQTSNAWQPSTNKEQNLGLMSSVSYVKKEPGDMSTEQQN
RHNLSKLHGYSSVNSAQLEQSGASQGALKDEFSRGLPASTSMPPTTSTGLLPHSSASASAVTHLDSSVLLSSQIPSNASGIAARPSL
KKSAATQKKPLEALGSSPPPSSKKQKTSGGYAEQSIEQLNDVTAVSGVDLREEEEQLFSGPKEDSRVSEASRKAVQEEEERLILQK
APLQKKLIDIMAKCGLKGMSNDVEKCLSLSVEERMRGLISNLIRLSKQRVDFEKTRHRTVVTSDVRQQIMTINRKVRDEWEKKQ
AEAEKLRKLNDVDSNTGGDGDKDKDDGRAKSTKVNKEEDDKMRTNAANVAARAAYGGDDMLSKWQLMAEQAKQKREGGV
DVSSSSQPAKDVNRKSSSTSERSTKDNQEGEKRGSTPFLASSVARKLGKSHAMAPQTRVARSISVKDVIAVLEREPQMSKSPLIHR
LYEKIHSEAPVEQG

>VrTAF4b-X2

MDPSIMKLLEDDEDETMHSGVDVEAFQAALNRDIGGDVSASQFPGSDAVLSQGSNNTSSQSSSQWPTSNHDSQSDGQNQEPKT
AQEQHPSEMEPKQHGSLGEHLQHVASQDVNNIHLSQKQSQDDSHQTPAVQVPLHNSQTIGIHNSGKDSVLNKEVVKSHNPSSES
QYAKLQQMSNQQATVSEQPSSQVNRSNKQVPFGLLLPILLPQLAKDRAMQLQTLFTKLKKDEIPKDSFVRLMKGIVGDQMLRL
ALAKVQMQPQARPNQASAGQQLPVRMPTVSSGARQLNDPHALAQMHQRSMNVAVDQSRMSSSAGQTMDSNARKSQEFDVKI
ESQGLQPNQLTSSTSNSVAQETERASVHIQGLNKQQQHHLHFASAYGNSGGNYNPYSGTTGSSTSSIKPQSHDSHMSQIPHQSIGS
NHLGGSTHGLSVIGMPKLEQQNSFNDPKRLPGGSVSSAVNNAASQQTSNAWQPSTNKEQNLGLMSSVSYVKKEPGDMSTEQQN
RHNLSKLHGYSSVNSAQLEQSGASQGALKDEFSRGLPASTSMPPTTSTGLLPHSSASASAVTHLDSSVLLSSQIPSNASGIAARPSL
KKSAATQKKPLEALGSSPPPSSKKQKTSGGYAEQSIEQLNDVTAVSGVDLREEEEQLFSGPKEDSRVSEASRKAVQEEEERLILQK
APLQKKLIDIMAKCGLKGMSNDVEKCLSLSVEERMRGLISNLIRLSKQRVDFEKTRHRTVVTSDVRQQIMTINRKVRDEWEKKQ
AEAEKLRKLNDVDSNTGGDGDKDKDDGRAKSTKVNKEEDDKMRTNAANVAARAAYGGDDMLSKWQLMAEQAKQKREGGV
DVSSSSQPAKDVNRKSSSTSERSTKDNQEGEKRGSTPFLASSVARKLGKSHAMAPQTRVARSISVKDVIAVLEREPQMSKSPLIHR
LYEKIHSEAPVEQG

>VrTAF4b-X3

MDPSIMKLLEDDEDETMHSGVDVEAFQAALNRDIGGDVSASQFPGSDAVLSQGSNNTSSQSSSQWPTSNHDSQSDGQNQEPKT
AQEQHPSEMEPKQHGSLGEHLQHVASQDVNNIHLSQKQSQDDSHQTPAVQVPLHNSQTIGIHNSGKDSVLNKEVVKSHNPSSES
QYAKLQQMSNQQATVSEQPSSQVNRSNKQVPFGLLLPILLPQLAKDRAMQLQTLFTKLKKDEIPKDSFVRLMKGIVGDQMLRL
ALAKVQMQPQARPNQASAGQQLPVRMPTVSSGARQLNDPHALAQMHQRSMNVAVDQSRMSSSAGQTMDSNARKSQEFDVKI
ESQGLQPNQLTSSTSNSVAQETERASVHIQGLNKQQQHHLHFASAYGNSGGNYNPYSGTTGSSTSSIKPQSHDSHMSQIPHQSIGS
NHLGGSTHGLSVIGMPKLEQQNSFNDPKRLPGGSVSSAVNNAASQQTSNAWQPSTNKEQNLGLMSSVSYVKKEPGDMSTEQQN
RHNLSKLHGYSSVNSAQLEQSGASQGALKDEFSRGLPASTSMPPTTSTGLLPHSSASASAVTHLDSSVLLSSQIPSNASGIAARPSL
KKSAATQKKPLEALGSSPPPSSKKQKTSGGYAEQSIEQLNDVTAVSGVDLREEEEQLFSGPKEDSRVSEASRKAVQEEEERLILQK
APLQKKLIDIMAKCGLKGMSNDVEKCLSLSVEERMRGLISNLIRLSKQRVDFEKTRHRTVVTSDVRQQIMTINRKVRDEWEKKQ
AEAEKLRKLNDVDSNTGGDGDKDKDDGRAKSTKVNKEEDDKMRTNAANVAARAAYGGDDMLSKWQLMAEQAKQKREGGV
DVSSSSQPAKDVNRKSSSTSERSTKDNQEGEKRGSTPFLASSVARKLGKSHAMAPQTRVARSISVKDVIAVLEREPQMSKSPLIHR
LYEKIHSEAPVEQG

>VrTAF4b-X4

MDPSIMKLLEDDEDETMHSGVDVEAFQAALNRDIGGDVSASQFPGSDAVLSQGSNNTSSQSSSQWPTSNHDSQSDGQNQEPKT
AQEQHPSEMEPKQHGSLGEHLQHVASQDVNNIHLSQKQSQDDSHQTPAVQVPLHNSQTIGIHNSGKDSVLNKEVVKSHNPSSES
QYAKLQQMSNQQATVSEQPSSQVNRSNKQVPFGLLLPILLPQLAKDRAMQLQTLFTKLKKDEIPKDSFVRLMKGIVGDQMLRL
ALAKVQMQPQARPNQASAGQQLPVRMPTVSSGARQLNDPHALAQMHQRSMNVAVDQSRMSSSAGQTMDSNARKSQEFDVKI
ESQGLQPNQLTSSTSNSVAQETERASVHIQGLNKQQQHHLHFASAYGNSGGNYNPYSGTTGSSTSSIKPQSHDSHMSQIPHQSIGS
NHLGGSTHGLSVIGMPKLEQQNSFNDPKRLPGGSVSSAVNNAASQQTSNAWQPSTNKEQNLGLMSSVSYVKKEPGDMSTEQQN
RHNLSKLHGYSSVNSAQLEQSGASQGALKDEFSRGLPASTSMPPTTSTGLLPHSSASASAVTHLDSSVLLSSQIPSNASGIAARPSL
KKSAATQKKPLEALGSSPPPSSKKQKTSGGYAEQSIEQLNDVTAVSGVDLREEEEQLFSGPKEDSRVSEASRKAVQEEEERLILQK
APLQKKLIDIMAKCGLKGMSNDVEKCLSLSVEERMRGLISNLIRLSKQRVDFEKTRHRTVVTSDVRQQIMTINRKVRDEWEKKQ
AEAEKLRKLNDVDSNTGGDGDKDKDDGRAKSTKVNKEEDDKMRTNAANVAARAAYGGDDMLSKWQLMAEQAKQKREGGV
DVSSSSQPAKDVNRKSSSTSERSTKDNQEGEKRGSTPFLASSVARKLGKSHAMAPQTRVARSISVKDVIAVLEREPQMSKSPLIHR

LYEKIHSEAPVEQG

>VrTAF4b-X5

MDPSIMKLLEDDEDETMHSGVDVEAFQAALNRDIGGDVSASQFPGSDAVLSQGSNNTSSQSSSQWPTSNHDSQSDGQNQEPKT
AQEQHPSEMEPKQHGSLGEHLQHVASQDVNNIHLSQKQSQDDSHQTPAVQVPLHNSQTIGIHNSGKDSVLNKEVVKSHNPSSES
QYAKLQQMSNQQATVSEQPSSQVNRSNKQVPFGLLLPILLPQLAKDRAMQLQTLFTKLKKDEIPKDSFVRLMKGIVGDQMLRL
ALAKVQMQPQARPNQASAGQQLPVRMPTVSSGARQLNDPHALAQMHQRSMNVAVDQSRMSSSAGQTMDSNARKSQEFDVKI
ESQGLQPNQLTSSTSNSVAQETERASVHIQGLNKQQQHHLHFASAYGNSGGNYNPYSGTTGSSTSSIKPQSHDSHMSQIPHQSIGS
NHLGGSTHGLSVIGMPKLEQQNSFNDPKRLPGGSVSSAVNNAASQQTSNAWQPSTNKEQNLGLMSSVSYVKKEPGDMSTEQQN
RHNLSKLHGYSSVNSAQLEQSGASQGALKDEFSRGLPASTSMPPTTSTGLLPHSSASASAVTHLDSSVLIPSNASGIAARPSLKKS
AATQKKPLEALGSSPPPSSKKQKTSGGYAEQSIEQLNDVTAVSGVDLREEEEQLFSGPKEDSRVSEASRKAVQEEEERLILQKAPL
QKKLIDIMAKCGLKGMSNDVEKCLSLSVEERMRGLISNLIRLSKQRVDFEKTRHRTVVTSDVRQQIMTINRKVRDEWEKKQAEA
EKLRKLNDVDSNTGGDGDKDKDDGRAKSTKVNKEEDDKMRTNAANVAARAAYGGDDMLSKWQLMAEQAKQKREGGVDVS
SSSQPAKDVNRKSSSTSERSTKDNQEGEKRGSTPFLASSVARKLGKSHAMAPQTRVARSISVKDVIAVLEREPQMSKSPLIHRLYE
KIHSEAPVEQG

>VrTAF4b-X6

MEPKQHGSLGEHLQHVASQDVNNIHLSQKQSQDDSHQTPAVQVPLHNSQTIGIHNSGKDSVLNKEVVKSHNPSSESQYAKLQQ
MSNQQATVSEQPSSQVNRSNKQVPFGLLLPILLPQLAKDRAMQLQTLFTKLKKDEIPKDSFVRLMKGIVGDQMLRLALAKVQM
QPQARPNQASAGQQLPVRMPTVSSGARQLNDPHALAQMHQRSMNVAVDQSRMSSSAGQTMDSNARKSQEFDVKIESQGLQPN
QLTSSTSNSVAQETERASVHIQGLNKQQQHHLHFASAYGNSGGNYNPYSGTTGSSTSSIKPQSHDSHMSQIPHQSIGSNHLGGSTH
GLSVIGMPKLEQQNSFNDPKRLPGGSVSSAVNNAASQQTSNAWQPSTNKEQNLGLMSSVSYVKKEPGDMSTEQQNRHNLSKLH
GYSSVNSAQLEQSGASQGALKDEFSRGLPASTSMPPTTSTGLLPHSSASASAVTHLDSSVLLSSQIPSNASGIAARPSLKKSAATQK
KPLEALGSSPPPSSKKQKTSGGYAEQSIEQLNDVTAVSGVDLREEEEQLFSGPKEDSRVSEASRKAVQEEEERLILQKAPLQKKLID
IMAKCGLKGMSNDVEKCLSLSVEERMRGLISNLIRLSKQRVDFEKTRHRTVVTSDVRQQIMTINRKVRDEWEKKQAEAEKLRK
LNDVDSNTGGDGDKDKDDGRAKSTKVNKEEDDKMRTNAANVAARAAYGGDDMLSKWQLMAEQAKQKREGGVDVSSSSQP
AKDVNRKSSSTSERSTKDNQEGEKRGSTPFLASSVARKLGKSHAMAPQTRVARSISVKDVIAVLEREPQMSKSPLIHRLYEKIHSE
APVEQG

>VrTAF4b-X7

MEPKQHGSLGEHLQHVASQDVNNIHLSQKQSQDDSHQTPAVQVPLHNSQTIGIHNSGKDSVLNKEVVKSHNPSSESQYAKLQQ
MSNQQATVSEQPSSQVNRSNKQVPFGLLLPILLPQLAKDRAMQLQTLFTKLKKDEIPKDSFVRLMKGIVGDQMLRLALAKVQM
QPQARPNQASAGQQLPVRMPTVSSGARQLNDPHALAQMHQRSMNVAVDQSRMSSSAGQTMDSNARKSQEFDVKIESQGLQPN
QLTSSTSNSVAQETERASVHIQGLNKQQQHHLHFASAYGNSGGNYNPYSGTTGSSTSSIKPQSHDSHMSQIPHQSIGSNHLGGSTH
GLSVIGMPKLEQQNSFNDPKRLPGGSVSSAVNNAASQQTSNAWQPSTNKEQNLGLMSSVSYVKKEPGDMSTEQQNRHNLSKLH
GYSSVNSAQLEQSGASQGALKDEFSRGLPASTSMPPTTSTGLLPHSSASASAVTHLDSSVLLSSQIPSNASGIAARPSLKKSAATQK
KPLEALGSSPPPSSKKQKTSGGYAEQSIEQLNDVTAVSGVDLREEEEQLFSGPKEDSRVSEASRKAVQEEEERLILQKAPLQKKLID
IMAKCGLKGMSNDVEKCLSLSVEERMRGLISNLIRLSKQRVDFEKTRHRTVVTSDVRQQIMTINRKVRDEWEKKQAEAEKLRK
LNDVDSNTGGDGDKDKDDGRAKSTKVNKEEDDKMRTNAANVAARAAYGGDDMLSKWQLMAEQAKQKREGGVDVSSSSQP
AKDVNRKSSSTSERSTKDNQEGEKRGSTPFLASSVARKLGKSHAMAPQTRVARSISVKDVIAVLEREPQMSKSPLIHRLYEKIHSE
APVEQG

>VrTAF5-1-X1

MDEDQIEGCVSGYLKQKGFAQKDDQLQLSNADSSLQPDTLNRAQLERGSARYHDGYGRLRSWAYRSLESYKHELLRVLYPVFV
HCFMDLVAKGHLQEAWNFFNTFREDHEMLHSRDLQKLGLVLSPTHLEEMEFAHSLRQSKFNIKICRYSYELLLQHLHSMQSTTII
GIINEHINFQVTAGQPSSISDDPEAVTLSGSIQDAVNQINQKEILWGMFEDSVEDHVDKAGALLSGTEKGEGEGKEGDNDESKKRS
IDVGKQGNSVKKVKKDKVSSATGKNAKPEATTISAAPRIKPEIPLPIVSTDVELSILDDLRNRVQLSSVALPSVNFYTFVNTHNGLS
CSSISHDGSLIVGGFSDSSLKVWDMAKLEQQSTAHFSQGGNDMSQNEQIIGQNSGRRQYTLYQGHSGPVYAATFSAAGDFLLSSS
ADKTVRLWSTKLNANLVCYKGHNYPIWDVQFSPAGHYFASCSHDRTARIWSMDRTKPLRIMAGHLSDVDCVQWHPNCNYIATG
SSDKTVRLWDVQSGECVRVFIGHRSMILSLAMSPDGRYMASGDEDGTIMMWDLSSGCCVTPLVSHTSCVWSLAFSCEGSLLASG
SADCTVKFWDVTTGIKVPRNEENRSGNANRLRSLKSLPTKSASVYSLQFSRRNLLFAAGAVAKSGC

>VrTAF5-1-X2

MDEDQIEGCVSGYLKQKGFAQKDDQLQLSNADSSLQPDTLNRAQLERGSARYHDGYGRLRSWAYRSLESYKHELLRVLYPVFV
HCFMDLVAKGHLQEAWNFFNTFREDHEMLHSRDLQKLGLVLSPTHLEEMEFAHSLRQSKFNIKICRYSYELLLQHLHSMQSTTII
GIINEHINFQVTAGQPSSISDDPEAVTLSGSIQDAVNQINQKEILWGMFEDSVEDHVDKAGALLSGTEKGEGEGKEGDNDESKKRS
IDVGKQGNSVKKVKKDKVSSATGKNAKPEATTISAAPRIKPEIPLPIVSTDVELSILDDLRNRVQLSSVALPSVNFYTFVNTHNGLS
CSSISHDGSLIVGGFSDSSLKVWDMAKLEQQSTAHFSQGGNDMSQNEQIIGQNSGRRQYTLYQGHSGPVYAATFSAAGDFLLSSS
ADKTVRLWSTKLNANLVCYKGHNYPIWDVQFSPAGHYFASCSHDRTARIWSMDRTKPLRIMAGHLSDVDCVQWHPNCNYIATG
SSDKTVRLWDVQSGECVRVFIGHRSMILSLAMSPDGRYMASGDEDGTIMMWDLSSGCCVTPLVSHTSCVWSLAFSCEGSLLASG
SADCTVKFWDVTTGIKVPRNEEKSGNANRLRSLKSLPTKSASVYSLQFSRRNLLFAAGAVAKSGC

>VrTAF5-2

MEDDKIVGYVTAYLKKKGFTQTEKIFQEEFQQNKTNNSSSSNSLLEPDIANHLLAFSQLESGPARFHDGYSKLRTWTYSSLDLYK
HELLRVLYPVFIHCFMDLVAKGHVQEARNFFNTFREDHEMMHLRDLQKLEGVLSPTHLEEMEFAHSLRQSKFNIKICEYSYELLL
QHLHSTQSTTILGIINEHINFQVSPGQPSLISDDPEAVTLTGSSQEAANRINQKEIHWGLLEDSLEERLEKAGTLLSDSEKGEGETKE
GENDETKKRSIEGGKQGGSVKKVKKDKGGSTTGKSAKPEASTVPAAPRVKPELPLPLIPTEVEQSILEDLRNRVQLSSVALPSVSF
YTFINTHNSLSCSSISHDGSLIAGGFSDSSLKVWDMAKLGQPTASSLLQGENDTSPNDQIFGQGVGKRQYTLFQGHSGPVYAASFS
PVGDFILSSSADSTIRLWSTKLNANLVCYKGHNYPVWDVQFSPVGHYFASSSHDRTARIWSMDRIQPLRIMAGHLSDVDCVQWH
ANCNYIATGSSDKTVRLWDVQSGECVRVFVGHRGMILSLAMSPDGRYMASGDEDGTIMMWDLSSGRCLTPLIGHTSCVWSLAF
SSEVSILASGSADCTVKLWDVNTSTKVSRAEEKSGNANRLRSLKTLATKSTPVYSLRFSRRNLLFAAGALAKSG

>VrTAF6-1

MSIVPKETIEVIAQSIGINSLSPDVALAVAPDVEYRMRQIMQEAIKCMRHSKRTILTADDVDVALNLKNVEPIYGFASGGPLRFKRA
VGHRDLFYIDDKDVDLKDVIEASLPKAPLDTAVTCHWLAIEGVQPAIPENAPIEVISAPSDTKKHEQKDDDLPVDIKLPVKHILSR

ELQMYFDKVAELTLSESDSALFKEALVSLATDSGLHPLVPYFTCFIADEVSRGLNNFPLLFALMRVVSSLLLNPHIHIEPYLHQLMPSVVTCLVAKRLGSRLADNHWELRDFTANLVASICKRFGHVYSNLQYRLTKTLLNAFLDPKKAMTQHYGAIQGLGALGPNVVRLLLLPNLETYMRLLEPEMLLEKQKNEMKRHEAWRVYGALLRAAGQCIYDRLKMFPTFSTPSPSAVWKTNAKVLTSSSRKRKADPDQLEQQPPLKKTATDGEVGVVPMNSSPAHKQEEAETRASSVDSIIGSSSSAQMKNETSLDGELRSNKGDTQASKTSAALTQVWKDELNSGRILVSLFDLFGEGILSFIQAPEMYMFL

>VrTAF6-2

MSFVPKETIEVIAQSIGITNLSPDVALALAPDLEYRIREIMQESIKCMRHSMRTFLSTEDVDTALALRNLEPIYGFTSNDPPRFKRAAGHKDLFYIDDKDVDIKDLIEAPLPKAPLDTSITSHWLAIEGVQPAIPENAPVEAPSSEIRKSEYKEDGLSVDVKLPVKHLITRELQLYYEKITDLTLNKSGSIPFRRALVSLATDSGLHPLVPYFTCFVADEVARNLHNLAVLFALMRLVRSLVQNSQIHIELYLHQLMPPIITCLVAKRIGSRLSDDHWELRNFSANLVASICQRFGHIYHNLQPRVTKTFLHSFLDPTKALPQHYGAIKGIEALGSRVIHLLILPNLEPYLHLLEPEMQPEKQKNEMKRHEAWQVYGALLGAVGKCVHEKVKNFSNLFSLPTRLISRGSGKAIIPMSGKRKASADNLMQQQPPMKKLSTDGHGGVIPMNSMSVDMQGSTGGFSTMMGAPAMSMTRQISTDKASGREVVDQQKKVSATLAQAWKDDIDAGNLVSSVVELFGERVLPFVPNPEAFMFL

>VrTAF6-like

MHIRTTLRKFLPIYGFASGGPLRFKRAVGHRDLFYIDDKDVDLKDVIEASLPKAPLDTAVTCHWLAIEGVQPAIPENAPIEVISAPSDTKKHEQKDDDLPVDIKLPVKHILSRELQMYFDKVAELTLSESDSALFKEALVSLPTDSGLHPLVPYFTCFIADEVSRGLNNFPLLFALMRVVSNGNSAPNLETK

>VrTAF7

MEEQFILRVPPNVAERIERLLNETDPSSSEDKSLDLSFSEDGRSGTFVIGNEHFPASLLDLPCVVESYKTYDDNSLIKTADIGQMIMVRESGDAAPDVIEYRHGLTPPMRDARKRRFRREPDLNPELVSRVEKDLLKIMARGTAENIDAEAAEQEVEENARGANKKVAPKPAPKHDVPENLTNAGEPDRSDSEESDDSV

>VrTAF8-1

MASLGLISDFELENKSHLGYKGKVLNMSNGGGKTGRQLEQPGPWRRRKVGDGDDFARAIAKIAVAQVCESEGFQAFQQSALEALSDVVARYIFNVGKSAHCHANLAGRTECHAFDVIQGLEDMGSVQGFAGASEVDHCLESSGVIREIFHFVNEGEPVVFAHPIPRFPVVKERVLNPSFLQKGEEPPGDHIPAWLPAFPDPQNYSQPPVVNGRGTEPRAVKFEQERENGKGEWPVLNLKQQMVSNMFEKSALDPADTKAKRIAAEGNPFLAAPLKIEDKEIASVPPAAKLFNDVVLDYPVVENFVENEPISALETFAPAIEAMKSTCCDSKEGQTKSFVNEKPIVRFKIGIKNKLLGRSIGLIPQTEEHNKTLPWFAMEDEKDDRKRRAEKILRESLENPDQLVQL

>VrTAF8-2

MNPMLKDSNTKATSKLPRRGKRKKKGLGVTDPQVAENPSKFSFAIAKIAVAQICQSAGFKKSENNALETLTAVSTRYLEAIVRSAASFANASNRTDCNLFDLVNGIHDLCSVQGFPGGSALHKDDLLRSSALREIMNFVNLSDKVPFAKPIQCRNDSDVTIDSGTLMCFSNQTKTHIPGWLPHFPEQNCDQVLVKERKCGEKYWEDSFTVDENSVISQSNHMNGKEGKDTRRELPERRERMKFKIRGEEEKQDGLGVNMMSGVCKGRKRVSWNHYKMNGCIIENNKDEKR

>VrTAF8-like

MTNGGGRAAPDDYGRAAVRLALAQLCNAAGFHSATSSALDAFADVAIRYLLDLGRTAESHANHAGRSQCTVFDAIRGFEDLGAPRAFSSPGGVRDIVSFVESADEVPFAQPIPRFPVVQERRRIPSFYQMGETPPSKHIPSWLPALPDPHTYIHTPVWDERVSDPREDKIEQARQRRKAERSLLSLQKRLLLRNGSSEAKARTSASPDSTVLETQCVGDDHKDVDKDGAPVVKVSVLDEGNVGDRNRVSVLDAFAPAIEMLGSGGLGGDEDDGLVEIDRSELPVVRPTVHFKFRTGKKFIGESLDMRIRNKDASRTVGLVGREDERDDKKRRAEYILKQSMENPQELTLL

>VrTAF9-1-X1

MSDKDEELAMPRDAKIVKSLLKSMGVGEYEPPVIHTFLELWYRYIVDVLTDAQVYSEHAGKSEIDCDDVKLAIQSKLNFSFSQPPPREVLLELAQNRNKIPLPKTIAGPGIPLPPDQDTLTSPNYMFGIPSQGSGEPEETEDEETSIPNPSQEEKTDMQQQDPHQRLVQFVLLTNWFQNGSLRSEVLVN

>VrTAF9-1-X2

MSDKDEELAMPRDAKIVKSLLKSMGVGEYEPPVIHTFLELWYRYIVDVLTDAQVYSEHAGKSEIDCDDVKLAIQSKLNFSFSQPPPREVLLELAQNRNKIPLPKTIAGPGIPLPPDQDTLTSPNYMFGIPSQGSGEPEETEDEETSIPNPSQEEKTDMQQQDPHQRVSFPTSFHD

>VrTAF9-2

MGDKDEESGMPRDAKIVKSLLKSMGVEEYEPRVIHKFLELWYRYVVDVLTDAQVYSEHAGKSSIDCDDVKLAIQSKINFSFSQPPPREVLLELAQNRNKIPLPKTIAGPGIPLPPDQDTLISPNYQFGIRNKRPAEPLEETEDEETTIPNPTQEDKVELQQNPHQRVSFPLPKRQKD

>VrTAF10

MNQNPQSSDGRGDDDTALSDFLASLMDYTPTIPDELVEHYLAKSGFQCPDVRLTRLVAVATQKFVAEVAGDALQHCKARQATIPKDKRDKQQKDKRLVLTMEDLSKALREYGVNLRHQEYFADSPSTGMDPATREE

>VrTAF11

MKQSKDPFEAAFEESPPESPTEIEADAIHNDSHNHNHNQNPIPSPSVLPLNPGPPQKASAAVKNKDKDKDDEEEEEEDNMDVELSKLPSTGDPHKMAKMQTILSQFSEEQMSRYESFRRAGFQRANMKRLLASITGTQKISVPMTIVVSGIAKMFVGEVVETARIVMKERKESGPIRPCHLREAYRRLKLEGKVFKRSASRLFR

>VrTAF12

MDSQAPATGTTPRSAAEPSQSQPPKSSPPILPSSTSSTPPVSAPTQSSPNPNPSSSPNPSPIQTPNPKPPTPTPAQPRPTQSFNRTLPPSQ

PQFPHFSSAPSPPSAPGGAAAPRGGMAIGVPAHHQSPSPPFSSSFGQHFGGLGRTGVSVAEPTSNSSTSQVRTPVQGMGMLGPQM
RPSGIAAHQQRPVQSSLRPPSSAPNTQPGGSQSFQGHGIMRPSSVASPATPSQGASQSVQSLNQPWLSSGPLGKPPLPSTAYRQQLN
PSSMQQRSHIPPQQQSTPTSSQQQQQQQQPPLSNQSQEHFGQQVQPSRASHHVPHQQQVTRLQGPGNQKPSSLVVAQTSVVQPV
SQSRLTNADTEEPCNSILSKRSIHELVNQVDPLEKLDHEVADILVDIAENFLESIIRSGCSLAKHRKSTTLEAKDILLHLEKNWNMT
LPGFGGDEIKSYRKQITSDIHKERLSAIKKSVAATEAAHAKGAGQASGSAKGNQAKTPMNIIGSPNLKSS

>VrTAF12b

MEVIEKKPNSAAESEIREECKVGVRSVTMALTFRTLFLMLLAILSLLLFEDWVSTPSCNNTDNIIDDNLKVMMVADLLLSDSGFV
NRFFRDYYMSKFFRKSFEVLRPDLLLVLGDVSARGSELTRSKWVSVLRRFYRVLGPFVGLPFHAVLGDRDVGECGDVDVDRVS
WIASKFPGLDSSGCAAFEIGNVSFVTLNAVALLCGGSGGLRFDVEKVIERESVEVHMGMERVVKRVNGFGEFADADVLSGSGPV
VLLHLPLDQTRNEHFGSVGDFEKYWTSSMEGLNVVPESRGGQYKLLHMLPPNASEYILQALKPRIIFSAHRYTFSDHVHGDRTR
EISVPAMSWNARDDPGFVIASFQKAGRAVSISHCSLARESQIVLVYISVMFLFCLVCLKGYSESATGIHADSINSKEKSIIDGPCYQL
EDNRLCCSKARFLTQIDSPAFRLPSIQLSSLRLPLRAHGRIIAPIDQSITSKSMAESASPSSKPSPMDPQNPAPSSNPTIPSPSHNNMPS
PSLPPLPQDQQQQQQQQQQNLHQQLSPPQQQQPLVSSQAMNTINGINPISNFQLQQTLQRSPSMSRLNQIQPQQQAQQQQQFGV
MRQQAGLYGGQMSFAAAGGGAGQQQQLGGSNLSRSALIGQSGHFPMLSGAGSQFNLLSSPRQKGGLVQQSQFSGNSAGQSLQ
GMQAMGMIGTPNLPSQLRANGALAYAQQLRMGHGQIRQQMSQQSSLNTGQVQGLPRSSSLAFMNSQLSGLSQNGQPGMVHNS
LTQQQWLKQMPAMSGPASPLRLQQQQRQSLASSTQLQQNSMSLNQQQLSQLIQQQKSMGQSQLHQQQQQQQPQQQLQQQLLH
QQSQQQSHPQASVHQQQQSPRMPGTAGQKSFSLTGSQPDATASGATTPGGSSSQGTEATNQVLGKRKIQDLVAQVDPQGTLDPE
VIDLLLELADDFIDSTTTHGCILAKHRKSSTLESKDLLLHLEKNWDLTIPGYSSEEKKYQSKPQLNDLHKRRLDMIRTMMESSASE
SNINSSKELSRQGISNPTPMGAHHLVRPMSSEQLVSQAAGSQMLQQMTRF

>VrTAF13

MSSSAAGTSSKPRAASSQPSETSSKRKRGVFQKELQHMMYGFGDDPNPLPESVALMEDIVVEYVTELVHKAQDIGSQRGKLSVE
DFLYLIRKDLPKLNRCTELLSMNEELKQARKVFESDEEKLRKVFEVDEAVEG

>VrTAF14b-1

MSHSQPLPLKRQGEHLSDDGASAIKPSRLKIAIPSEDSDKKNANNRIKDVEICVPIVYGTIAFYLGRKASESQSHKWTVYVRGAS
NEDLGVVIKRVVFQLHPSFNNPTRVVESPPFELSESGWGEFEIAITLYFHSDVCEKQLDLYHHLKLYPEDESGPQSTKKPVVVESY
NEIVFPEPSEGFLARIQNHPAVNVPRLPAGLNLPSPVPIDTMSDKERGDTKDHSLNQWFLNFSEADELLKLAAARQQVQAHIVKL
RRQLSLVEGLPQLSKPPSGYECT

>VrTAF14b-2

MTISSSSKKHGQDQPELSGPTPKSQRTKMGKSDDNDKKNLGKKLKDVEISVPIVYGNIAFWLGKKASEYQSHKWTVYVRGATN
EDLGAIIKRVVFQLHSSFNNPTRVVESPPFELSEAGWGEFEVAITLYFHSDVCDKPLNLYHHLKLYPEDENSSMSAKKPVVVEFYD
EIVFPDPSEAFLARLQSHPAVNLPRLPAGLTLPPPVPVEDASKRRKGDTKDHSLSQWFTNFSEADELLQLAAARQQVQAHIAKLR
RQINLIDGQHQQFKSSSDQ

>VrTAF15-1

MATHPGKQALSNGSVYYVCNLPYGTDENMLAEYFGTIGLVKKDKRTGRPKIWLYRDKETNEPKGDATVTYEDPHAAIAAVEWFN
NKDFHGNIIGVFIAESKNKDDQTYNSAGVEPVVAGTVVGLEETTKDVNGGSGRGRGQNDPSGKAWQQDGDWLCPNTSCSNVN
FAFRGACNRCGTARPAGAAGISGAGGRGKGRAAGQEPGGVGRPVGGGLFGPNDWPCPMCGNINWAKRTKCNICNTNKPGHNE
GGVRGGRGGGYKELDEEEIEETRRRRREAEDDGELYDEFGNLKKKFRAKTQQAEAARGLPGSGRAGWEVEELGIDKDGRESRD
RGRERNDGESRSRERSDKERQSSWNRDRDRDRGRDRDRDWDYVDRDRDYGRDRDRSRHRY

>VrTAF15-2

MSRPGDWNCRTCNHLNFQRRESCQRCGEPRSAGDYGGAFGGRGSSSFGFTTGPDVRPGDWYCTVGNCGAHNFASRSSCFKCG
VSKEDSSTGSYDLDITRMRPYGFGSGSSARPGWKSGDWICTRSGCNEHNFANRMECYRCNAPRDSNSGRPPYSS

>VrTAF15b-1

MLRCTCTTSLPLFTVSTIPYRIPIMSGNYDQDGGGYGRNDGGGYGGRGGGGFGGRGGYQGGDRGGRGGGRGGGSGRDGDWR
CPNSSCGNLNFARRVECNKCGAPSPAGANDRGGGGYNRGGYGNSRGGRSGNYDGGRGNGYNSSRGNNNVGRSGGGNRGSQG
REDGGYGQVPAPAAQSYGGAGGNYPPAYNSSGGSSNYETDAVPPPASYTGGPASHPPPYGSNTGGYGGGDAHNGGRSGQPVGY
DSGYGAGSQGGFGGAPDEPPAKVKQCDENCGDSCDNSRIYISNLPPDVTIEELRELFGGIGQVGRIKQKRGYKDQWPWNIKLYT
DEKGNNKGDGCLVYEDPSAAHSAGGFYNNYDLRGYKIAVTMAEKSAPKAPPAYNHGGNRGGYGGDRRRDNYRDAGGSGPDR
RDNYGGNRSRPY

>VrTAF15b-2

MSGAYGQDGGGGSAPPSYGASGGYGSGGGSYGGGTYGGGGAGGGYGGNDGGGGYGGKNSGNGGGYGGNDGGGYGGRSGY
GGNDGGGYGGRGGGQGGRGGGGFGGGYGGRGGGGGGGGGGGGGGYQGGDRGGRGGGRGGGGRGGSGRDGDWRCPNQS
CGNLNFARRVECNKCGAPCPNPNSSNERGGSGGGGGGFSRGGGGGGGYGNTRGGRSGNYDGGRGNDYNSGGGRSGNYDGGR
GNDYNSGRGGSNDGRGGSYRGNQGREDGSYGQVPAPNAQSYGSAGGSFPPSYSSYGGNASYGTDAVPPPSSYTGGPNSYPPSY
GGNVGGYGGDNQGNGRSGGRSGPPSGFDNSYGAGNRGGFGGSPAEPPAAVKQCDENCDDTCDNSRIYISNLPPDVSIEELRELF
GGIGQVGRIKQKRGYKDQWPWNIKIYTDENGNNKGDACLAYEDPSAAHSAGGFYNNYDLRGYKISVAMAEKSAPRAAPAYNQ
GGNRGGYGGDRRRDNYRDGGGSGPDRRDHYGNRSRPY

II. Protein sequences from *Arabidopsis thaliana*

>NP_187953.1 TATA binding protein 1 [*Arabidopsis thaliana*] AT3G13445.1 AtTBP1

MTDQGLEGSNPVDLSKHPSGIVPTLQNIVSTVNLDCKLDLKAIALQARNAEYNPKRFAAVIMRIREPKTTALIFASGKMVCTGAK
SEDFSKMAARKYARIVQKLGFPAKFKDFKIQNIVGSCDVKFPIRLEGLAYSHAAFSSYEPELFPGLIYRMKVPKIVLLIFVSGKIVIT
GAKMRDETYKAFENIYPVLSEFRKIQQ

>NP_175948 TATA binding protein 2 [*Arabidopsis thaliana*] AT1G55520 AtTBP1

MADQGTEGSQPVDLTKHPSGIVPTLQNIVSTVNLDCKLDLKAIALQARNAEYNPKRFAAVIMRIREPKTTALIFASGKMVCTGAK
SEHLSKLAARKYARIVQKLGFPAKFKDFKIQNIVGSCDVKFPIRLEGLAYSHSAFSSYEPELFPGLIYRMKLPKIVLLIFVSGKIVIT
GAKMREETYTAFENIYPVLREFRKVQQ

>NP_174552.1 HAC13 protein (HAC13) TBP-associated factor 1 [*Arabidopsis thaliana*] AT1G32750 AtTAF1

MAESNGKGSHNETSSDDDDEYEDNSRGFNLGFIFGNVDNSGDLDADYLDEDAKEHLSALADKLGSSLPDINLLAKSERTASDPA
EQDYDRKAEDAVDYEDIDEEYDGPEVQVVSEEDHLLPKKEYFSTAVALGSLKSRASVFDDEDYDEEEEQEEEQAPVEKSLETEK
REPVVLKEDKALEYEEEASILDKEDHMDTEDVQEEEVDELLEGTLDDKGATPLPTLYVEDGMVILQFSEIFAIHEPPQKRDRREN
RYVTCRDKYKSMDISELVEDDEEVLLKSHGRIDTHVEQADLIQLDVPFPIREGLQLVKASTIGGITPESREFTKLGRDSCIMGELLK
QDFIDDNSSLCQSQLSMQVFPLDQHEWERRIIWEHSPEISGNSGEIFEPGLEPEGMLVKGTNSETEQESLNVVNSRVQVQADNNM
FVPFSANLLESFGSRGSQSTNESTNKSRHHPQLLRLESQWDENHLSGNDEAGVKKIKRLEKDALGRFSRLVLRERDLGDEAWLD
SIIWDSEKELSRSKLIFDLQDEQMVFEIFDNEESKNLQLHAGAMIVSRSSKSKDETFQEGCESNSGWQFNLSNDKFYMNGKSSQQ
LQANTNKSSVHSLRVFHSVPAIKLQTMKSKLSNKDIANFHRPKALWYPHDNELAIKQQGKLPTRGSMKIIVKSLGGKGSKLHVGI
EESVSSLRAKASRKLDFKETEAVKMFYKGKELDDEKSLAAQNVQPNSLVHLIRTKVHLWPWAQKLPGENKSLRPPGAFKKKSD
LSTKDGHVFLMEYCEERPLMLSNAGMGANLCTYYQKSSPEDQRGNLLRNQSDTLGNVMILEPGDKSPFLGEIHAGCSQSSVET
NMYKAPIFPQRLQSTDYLLVRSPKGKLSLRRIDKIVVVGQQEPRMEVMSPGSKNLQTYLVNRMLVYVYREFFKRGGGEHPIAAD
ELSFLFSNLTDAIIKKNMKIIACWKRDKNGQSYWTKKDSLLEPPESELKKLVAPEHVCSYESMLAGLYRLKHLGITRFTLPASISN
ALAQLPDEAIALAAASHIERELQITPWNLSSNFVACTNQDRANIERLEITGVGDPSGRGLGFSYVRAAPKAPAAAGHMKKKAAA
GRGAPTVTGTDADLRRLSMEAAREVLIKFNVPDEIIAKQTRWHRIAMIRKLSSEQAASGVKVDPTTIGKYARGQRMSFLQMQQQ
AREKCQEIWDRQLLSLSAFDGDENESENEANSDLDSFAGDLENLLDAEEGGEGEESNISKNDKLDGVKGLKMRRRPSQVETDEE
IEDEATEYAELCRLLMQDEDQKKKKKKMKGVGEGMGSYPPPRPNIALQSGEPVRKANAMDKKPIAIQPDASFLVNESTIKDNRN
VDSIIKTPKGKQVKENSNSLGQLKKVKILNENLKVFKEKKSARENFVCGACGQHGHMRTNKHCPRYRENTESQPEGIDMDKSA
GKPSSSEPSGLPKLKPIKNSKAAPKSAMKTSVDEALKGDKLSSKTGGLPLKFRYGIPAGDLSDKPVSEAPGSSEQAVVSDIDTGIK
STSKISKLKISSKAKPKESKGESERRSHSLMPTFSRERGESESHKPSVSGQPLSSTERNQAASSRHTISIPRPSLSMDTDQAESRRPH
LVIRPPTEREQPQKKLVIKRSKEMNDHDMSSLEESPRFESRKTKRMAELAGFQRQQSFRLSENSLERRPKEDRVWWEEEEISTGR
HREVRARRDYDDMSVSEEPNEIAEIRRYEEVIRSEREEEERQKAKKKKKKKKLQPEIVEGYLEDYPPRKNDRRLSERGRNVRSR
YVSDFERDGAEYAPQPKRRKKGEVGLANILERIVDTLRLKEEVSRLFLKPVSKKEAPDYLDIVENPMDLSTIRDKVRKIEYRNRE
QFRHDVWQIKYNAHLYNDGRNPGIPPLADQLLEICDYLLDDYEDQLKEAEKGIDPND

>NP_177536.2 TBP-associated factor 2 [*Arabidopsis thaliana*] AT1G73960 AtTAF2

MAKARKPKNEEAPGAKTSENTGAKVLHQKLFLSIDFKKRQIYGYTELEVSVPDIGIVGLHAENLGIESVLVDGEPTVFEYYPHHQ
NSETESNWNSVSDPASAADAAAMEYVGVLKREDTANLLINCCKPSKDLSEQLDSVTLENGSQSSGEAKQNVKLIRINYWVEKIE
SGIHFDGNIVHTDNQMRRARCWFPCIDDEYHRCSFDLEFTVPHNFVAVSVGKLLYQVMCKEDTTQKTYVYELAIPIAPRWVSLVA
GPLEILPDQTNFLISNLCLPHDLSRLRNTMEFFHEAYSYYEDYLSANFPFGFYKQVFLPPEMVVTSSTSGASLSIFSSHILYDERVID
QTIDTRIKLASALAKQWFGVYITPESPNDDWLLDGLAGFLTDMFIKQFLGNNEARYRRYKANCAVCKADDSGAMCLSSSPSCRD
LFGTHSIGMHGKIRSWKSGAVLQMLEKQMGSDSFRKILQKIISRAKDPSNSIRSLSTKEFRQFANKIGNLERPFLKEFFQRWVASY
GCPVLRIGLSYNKRKNNVEMAALRECTAALDARLSVIGATSDSESRDVDAGWPGIMSIRVYELDGMSDHPKLPMAGDRWQLLE
LPCHSKLAAKRYQKPKKGGKPDGAEDNVDAIAPLENKTSIESPLAWIKADPEMEYIAEIHLHQPLQMWVNQLEKDGDVVAQAQ
AIASLEALKQHSFSIVNALKNVLTDSKVFWRIRIAAAFALAKTASEESDWAGLQHLIKFYKSRRFDAEIGLPKPNDFRDFPEYFVL
EAIPHAIAIVRGAEGKSPREAVEFILQLLKYNDNSGNSYSDVFWLAVLVQSVGDLEFCQQSLTFLAPLLKRIDRLLQFDRLMPSYN
GILTISCIRTLAQTALKLSDSISFDHICKLIEPFRNSDTILQIRIEGSRALLDIEYQSKGISSALLLFMKYLVEESSLRGQVKLCVHTMR
LCQIAVGCDSDDCVDTVTLLDLLHLFKSHVVFNNELLRYYLFCIFQILAGRPPTLFGVPKEKPLQLVDVEACIEPKNVFLVPGAEA
GEPSLSALGDAKGQSLDVAPYGVPIIPQEMFMPIVPELMLPEPVAAYDETQHLEPRMESQNQPSHENPIVHEIPSDVEGPTEELAH
REANPPTKEPQKEPDVVSVSVSHEVKKSVIRIKVRPSGATSRAEGSARTIERSQGIVVRHDIDRGQTSSASVDAPQRISTDAVSISN
QNHVEEVNSCHDVGSRMTASIGSVKFASEGDIFGKELQCTAESGKPSTSQKADNNNRTVPPSFLPLDHSMENEAQQKYASLQTLS
IGKEKEKKKDKEKKEKKRKREDPVYLEKKRLKKEKKRKEKEMAKLVSSTTDPAKKKIESVAEVKEEEPSDGAMLIKVEPKTEPS
TAEARPLPKFRIKLKSKAFNNS

>NP_001190458.1 TBP-associated factor 4b [*Arabidopsis thaliana*] AT5G43130 AtTAF4b

MDPSIFKLLEEDEDESMHSGADVDAFQAALNRDIEGSMTTSIPHVTNPGNNHSSRQQFSTWKNGIGDSNINVQTQHSLESTQMK
EQEGSTLENQHQHDLKRANEPHLQHNQPQDLHRAGQLWENPSQVPQSTGLPISEKNPTGNESDRSHNQESESQYMKLQKMSSQ
QARGVEPPVNPMNVNPINRNPKQVPFAALLPTLMNQLDKDRALQLRTLYARLKKNEIPKEGFTRHMKDIVGDQMLRMAVSKLQ
QVNYNQGKIGIQAPSTEINNQKSQSDPRAVHLNQLPSSASGTLGSSVPVQGLTKHPQHQMQHPPSSFPMYTTSGSFHSFPGPNTN
ASGSTLRPHLHDSHMRHVAHNQPMGSTGLGGPPQSTTNMMTMPKFERPSSVNDPSRVQGGATSHFQNSSSLPLNSAPGQGSSVS
HVKQESVDQSFEKNNAASMTSNEDLEKESSRMVLSTPNNMAPASSVSPSMTTQLDASTTMNSRGPLGTSQGGANARMPPKKPS
VGQKKPLETLGSSPPPPSKKQKVAGNSMDQSIEQLNDVTAVSGVNLREEEEQLFSGAKEDGRVSEASRRVVHEEEERLILQKNPL
QRKLAEIMAKAGLKQISNDVERCLSLCVEERMRGLLSHIIRLSKQRVDAEKSRHRTFITSDIRLQINEMNQKVKEEWEKKQAEAE
KLKKPSESEEGDGGVDSEKDKEDNRSKGVKGNKEDDDKMRTTAANVAARAAVGGDDAFLKWQLMAEARQKSVSEAGKDGN
QKTTSGGGKNSKDRQDGGRRFSGTESSCGVGIVYRVSSSRFWFAMMSFGFLFAGGRRVGKNQGSSLQPKVVRTISVKDVVAVLE
REPQMSKSTLMYRLIQ

>NP_197897.3 TBP-associated factor 5 [*Arabidopsis thaliana*] AT5G25150 AtTAF5

MDPEQINEFVVGYLKKKGFSSAAKDLESYHHQNNNGSSFTSVDYHNDPELTKLIRSFSQQEDDPTRYREGYSKLRSWAYNSLDL
YKHELLRVMYPVFIHCYMDLVGKGHTQEARAFFNSFRKDHEMVHLRDLQKLEGVLSPSHLEEMEFARSLRKSKVNIKFCQYSY
ELLLQYLHSTVSTLMLGIINEHINFQVYSGQPTSSSDDIEAVTIVGSFQDTANHINQKEIQWGLLEDSLEDRLEKTGGLLSDSEKGQ
GESKDGDADDSKKRSTEIGKQGSSLKKLKKDKAGNATAKVARLETITVSPAPRVKPELALPVMSTDVEQSILEDLRNRVQLSSVA
MPSVSFYTFVNTHNGLNCSSISHDGSLVAGGFSDSSIKVWDMAKIGQAGSGALQAENDSSDQSIGPNGRRSYTLLLGHSGPVYSA

TFSPPGDFVLSSSADTTIRLWSTKLNANLVCYKGHNYPVWDAQFSPFGHYFASCSHDRTARIWSMDRIQPLRIMAGHLSDVDCV
QWHPNCNYIATGSSDKTVRLWDVQTGECVRIFIGHRSMVLSLAMSPDGRYMASGDEDGTIMMWDLSTARCITPLMGHNSCVW
SLSYSGEGSLLASGSADCTVKLWDVTSSTKLTKAEEKNGNSNRLRSLRTFPTKSTPVHALRFSRRNLLFAAGAISKPAN

>NP_001030969.1 TATA BOX ASSOCIATED FACTOR 6 [*Arabidopsis thaliana*] AT1G04950 AtTAF6

MSIVPKETVEVIAQSIGITNLLPEAALMLAPDVEYRVREIMQEAIKCMRHSKRTTLTASDVDGALNLRNVEPIYGFASGGPFRFRK
AIGHRDLFYTDDREVDFKDVIEAPLPKAPLDTEIVCHWLAIEGVQPAIPENAPLEVIRAPAETKIHEQKDGPLIDVRLPVKHVLSRE
LQLYFQKIAELAMSKSNPPLYKEALVSLASDSGLHPLVPYFTNFIADEVSNGLNDFRLLFNLMHIVRSLLQNPHIHIEPYLHQLMPS
VVTCLVSRKLGNRFADNHWELRDFAANLVSLICKRYGTVYITLQSRLTRTLVNALLDPKKALTQHYGAIQGLAALGHTVVRLLIL
SNLEPYLSLLEPELNAEKQKNQMKIYEAWRVYGALLRAAGLCIHGRLKIFPPLPSPSPSFLHKGKGKGKIISTDPHKRKLSVDSSE
NQSPQKRLITMDGPDGVHSQDQSGSAPMQVDNPVENDNPPQNSVQPSSSEQASDANESESRNGKVKESGRSRAITMKAILDQIW
KDDLDSGRLLVKLHELYGDRILPFIPSTEMSVFL

>NP_175926.1 TBP-associated factor 7 [*Arabidopsis thaliana*] AT1G55300 AtTAF7

MEEQFILRVPPSVSERIDRLLSEDASTSDEIPLDLFFSEDGRNGTFMIGNDEFPASLLDLPAVVESFKTYDDCALVKTADIGQMIMV
REPGDPAPNTVEYRHGLTPPMKDARKRRFRREPDLNPELVQRVERDLLNILSGGTVENVHEQEEPATNENASNASKKVSSSSPTP
VEKPEAPETGTSNPTGVEPERSESEDSDDSM

>NP_567964.1 TBP-associated factor 8 [*Arabidopsis thaliana*] AT4G34340 AtTAF8

MNTERAQEGDRNDAASSSGCSESYEFSHAAAKAAVAQVCESVGYENFKDPALESLSGFALQYILQLGKTATSFANLTGRSQCNVF
DIILALDDLTDNNGEQGISSESCSLGRSIKLREIIDFVNSSEEVPFSQPLPSFPVAISDRSRKMIPSFVEIGETPPGKHIPLWLPAFPDPH
TYKETPMWIERVSDPRGDKIEQARQRRKAERALLSLQRKLVCKISSRNPVWGDMDGVKEEMRDDESELRSVSSGEKVESLNRD
GLSVIEAFAPAMEAARDGFSSEAHTEWKKNKPVALSKLRTEKKFLGQPLDLSLQMKGEDRPISFVREEDRDDKRRRAEFILRQC
MENPVDLNQL

>NP_175816.1 TATA binding protein associated factor 9 [*Arabidopsis thaliana*] AT1G54140 AtTAF9

MAGEGEEDVPRDAKIVKSLLKSMGVEDYEPRVIHQFLELWYRYVVEVLTDAQVYSEHASKPNIDCDDVKLAIQSKVNFSFSQPP
PREVLLELAASRNKIPLPKSIAGPGVPLPPEQDTLLSPNYQLVIPKKSVSTEPEETEDDEEMTDPGQSSQEQQQQQQQTSDLPSQTP
QRVSFPLSRRPK

>NP_194900.1 TBP-associated factor10 [*Arabidopsis thaliana*] AT4G31720 AtTAF10

MNHGQQSGEAKHEDDAALTEFLASLMDYTPTIPDDLVEHYLAKSGFQCPDVRLIRLVAVATQKFVADVASDALQHCKARPAPVV
KDKKQQKDKRLVLTMEDLSKALREYGVNVKHPEYFADSPSTGMDPATRDE

>NP_193761.1 TBP-associated factor 11 [*Arabidopsis thaliana*] AT4G20280 AtTAF11

MKHSKDPFEAAIEEEQEESPPESPVGGGGGGDGSEDGRIEIDQTQDEDERPVDVRRPMKKAKTSVVVTEAKNKDKDEDDEEEEE
NMDVELTKYPTSSDPAKMAKMQTILSQFTEDQMSRYESFRRSALQRPQMKKLLIGVTGSQKIGMPMIIVACGIAKMFVGELVETA
RVVMAERKESGPIRPCHIRESYRRLKLEGKVPKRSVPRLFR

>NP_566367.1 TBP-associated factor 12 [*Arabidopsis thaliana*] AT3G10070 AtTAF12

MDQPRQSSTASQPPETPPQPSDSKPSTLTQIQPTPSTNPSPSSVVSSIPSSPAPQSPSLNPNPNPPQYTRPVTSPATQQQQHLSQPLVRP
PPQAYSRPWQQHSSYTHFSSASSPLLSSSSAPASSSSSLPISGQQRGGMAIGVPASPIPSPSPTPSQHSPSAFPGSFGQQYGGLGRGT
VGMSEATSNTSSPQVRMMQGTQGIGMMGTLGSGSQIRPSGMTQHQQRPTQSSLRPASSTSTQSPVAQNFQGHSLMRPSPISSPNV
QSTGASQQSLQAINQPWLSSTPQGKPPLPPPSYRPQVNSPSMQQRPHIPQQHISTSAATPQPQQQQSQQQHQPQEQLQQLRSPQQP
LAHPHQPTRVQGLVNQKVTSPVMPSQPPVAQPGNHAKTVSAETEPSDDRILGKRSIHELLQQIDPSEKLDPEVEDILSDIAEDFVES
ITTFGCSLAKHRKSDILEAKDILLHVERNWNIRPPGFSSDEFKTFRKPLTTDIHKERLAAIKKSVTATEAANARNQFGHGTANARG
GQAKTPSNPMGSTTFNH

>NP_564023.1 Transcription initiation factor TFIID 12b [*Arabidopsis thaliana*] At1g17440 AtTAF12b

MAEPIPSSSLSPKSLQSPNPMEPSPASSTPLPSSSSQQQQLMTAPISNSVNSAASPAMTVTTTEGIVIQNNSQPNISSPNPTSSNPPIGA
QIPSPSPLSHPSSSLDQQTQTQQLVQQTQQLPQQQQQIMQQISSSPIPQLSPQQQQILQQQHMTSQQIPMSSYQIAQSLQRSPSLSRL
SQIQQQQQQQHQGQYGNVLRQQAGLYGTMNFGGSGSVQQSQQNQQMVNPNMSRAGLVGQSGHLPMLNGAAGAAQMNIQPQ
LLAASPRQKSGMVQGSQFHPGSSGQQLQGMQAMGMMGSLNLTSQMRGNPALYAQQRINPGQMRQQLSQQNALTSPQVQNLQ
RTSSLAFMNPQLSGLAQNGQAGMMQNSLSQQQWLKQMSGITSPNSFRLQPSQRQALLLQQQQQQQQQLSSPQLHQSSMSLNQ
QQISQIIQQQQQQSQLGQSQMNQSHSQQQLQQMQQQLQQQPQQQMQQQQQQQQQMQINQQQPSPRMLSHAGQKSVSLTGSQP
EATQSGTTTPGGSSSQGTEATNQLLGKRKIQDLVSQVDVHAKLDPDVEDLLLEVADDFIDSVTSFACSLAKHRKSSVLEPKDILLH
LEKNLHLTIPGFSSEDKRQTKTVPTDLHKKRLAMVRALLESSKPETNASNSKETMRQAMVNPNGPNHLLRPSQSSEQLVSQTSGP
HILQHMTRY

>NP_171768.2 TBP-associated factor 13 [*Arabidopsis thaliana*] At1g02680 AtTAF13

MSNTPAAAASSSSKSKAAGTSQPQEKRKTLFQKELQHMMYGFGDEQNPLPESVALVEDIVVEYVTDLTHKAQEIGSKRGRLLVD
DFLYLIRKDLPKLNRCRELLAMQEELKQARKAFDVDEKELVD

>NP_199373.1 YEATS family protein 14b [*Arabidopsis thaliana*] AT5G45600 AtTAF14b

MTNSSSSKKQAQDQPETSEPTLKSLKTKMTKSDEKQKKLKDIEISVPIVYGNVAFWLGKKASEYQSHKWAVYVRGATNEDISVV
VKKVVFQLHSSFNSPTRVIEEPPFEVSESGWGEFEIAMTLHFHSDVCDKPLSLYHHLKLYPEDESGPLTMKKPVVVESYDEIVFPD
PSESFLARVQNHPALTFPRLPSGYNLPAPMQVEDTGKKKRGDTKDHSLGQWFMSFSEADELLQLAAARQQVQAHIAKLRRQISL
LEGQNQTVKTGSDL

>NP_564565.1 TBP-associated factor 15 [*Arabidopsis thaliana*] AT1G50300 AtTAF15

MAGYPTNGSVYVSNLPLGTDENMLADYFGTIGLLKRDKRTGTPKVWLYRDKETDEPKGDATVTYEDPHAALAAVEWFNNKDF
HGNTIGVFMAESKNKNAGDAVEFVEFDGGAEETNGGAGRGRGQADSSAKPWQQDGDWMCPNTSCTNVNFAFRGVCNRCGTA

RPAGASGGSMGAGRGRGRGGGADGGAPGKQPSGAPTGLFGPNDWACPMCGNVNWAKRLKCNICNTNKPGQNEGGVRGGRG
GGYKELDEQELEETKRRRREAEEDDGEMYDEFGNLKKKYRVKTNQADTRPAVAAGRAGWEVEELGIDKDGRERSRDRQRDRG
RDHHYDKDRRRSRSRERERGKERDYDYDHDRDRDRDYGRERGSRYRN

>NP_568879.3 TBP-associated factor 15B [*Arabidopsis thaliana*] AT5G58470 AtTAF15b

MAGMYNQDGGGGAPIPSYGGDGYGGGGGYGGGDAGYGGRGASGGGSYGGRGGYGGGGGRGNRGGGGGGYQGGDRGGRG
SGGGGRDGDWRCPNPSCGNVNFARRVECNKCGALAPSGTSSGANDRGGGGYSRGGGDSDRGGGRGGRNDSGRSYESSRYDG
GSRSGGSYGSGSQRENGSYGQAPPPAAAIPSYDGSGSYPPPTGYGMEAVPPPTSYSGGPPSYGGPRGGYGSDAPSTGGRGGRSGG
YDGGSAPRRQEASYEDAATEKVKQCDADCDDNCDNARIYISNLPPDVTTDELKDLFGGIGQVGRIKQKRGYKDQWPYNIKIYT
DEKGNYKGDACLAYEDPSAAHSAGGFFNNYEMRGNKISVTMAEKSAPRAPTFDQRGGGRGGGGGGYGGGGGDRRRDNYSSG
PDRNHHGGNRSRPY

III.   Protein sequences from *Vigna angularis*

>VaTBP

MADQGLEGSQPVDLQKHPSGIVPTLQNIVSTVNLDCKLDLKTIALQARNAEYNPKRFAAVIMRIREPKTTALIFASGKMVCTGAK
SEQQSKLAARKYARIIQKLGFPAKFKDFKIQNIVGSCDVKFPIRLEGLAYSHGAFSSYEPELFPGLIYRMKQPKIVLLIFVSGKIVLT
GAKVRDETYTAFENIYPVLTEFRKNQQ

>VaTAF1

MGYDSASPSQDGRDEDDEEEYEESGKGNRFLGFMFGNVDNSGDLDVDYLDEDAKEHLSALADKLGPSLTDIDLSGKSPQTPPD
VVEQDCDEKAEDAVDYEDIDEEYDGPETEAANEEDYLLPKKEFFSAEASVCMESKASVFDDENYDEESEKEQDFLNEDSKPDNI
SLLEEQEETLVEASKEESALERELHVDSLQSEELDADIQKPEEEGTEVQKRSMAMPLPVLCVEDGVAILRFSEIFGIHEPLRKGEKR
EHRQPITRDRYKSLDFTDDFVEEDEEEFLKGSSQSLSQTKQVSVVHNDVSESNDVDLEFPKFGFLHTEPSVARKDDHQSKDSCHS
AEPMKGDFEEDLSWKDHPFIWTNFYPLDQQDWEDEIIWGNSPVQSNNNIESCEVSGPELGVSGGSEIEIESGIQNIQLEPHKILED
KDHNVLLSSPVSLEAFGSRDSSEAKTNLISRSLFHPQLLRLESRSEVDSSILADGKEGEISKHNQSGQITRFSKAISKNRDMMEGS
WLDEIIWEELDQPVVKPKLIFDLQDDQMHFEVLDSKDGAHLRLHAGAIILTRSSKSSSGDSSEVPGHGSQYGWRYVSNDKHYSN
RKTSQQLKSNSKKRSAHGVKVFHSQPALKLQTMKLKLSNKDIANFHRPKALWYPHDNEVAVKEQGKLPTQGPMKIIIKSLGGKG
SKLHVDTDETLSTVKAKASKKLDFKASETVKIFYLGRELEDQKSLAEQNVQPNSLLHLVRSKIHLWPKAQRVPGENKSLRPPGAF
KKKSDLSVKDGHVFLMEYCEERPLLLSNVGMGARLCTYYQKCSPDDQSGSLLRNTDSSLGHVISLDPADKSPFLGDLKPGCCQS
SLETNMYRAPVFPHKVPLTDYLLVRSPKGKLSLRRIDKINVVGQQEPLMEVFSPGSKNLQTYMMNRLLVHMCREFQAAEKRHL
PPHIRVDEFLSQFPYQSEASFRKKIKEYANLQRGANGQSILVKKRNFRIWSEDELRKMVLPELVCAYESMQAGLYRLKHLGITETH
PTNISSAMSRLPDEAIALAAASHIERELQITPWNLSSNFVACTSQGKENIERMEITGVGDPSGRGMGFSYARAPPKAPVSSAMVKK
KAAANRGGSTVTGTDADLRRLSMEAAREVLLKFNVPEEVIAKQTRWHRIAMIRKLSSEQAASGVKVDPTTISKYARGQRMSFL
QLQQQTREKCQEIWDRQVQSLSAVNADENESDSEGNSDLDSFAGDLENLLDAEEFEEGEEGTNDLKRDKGDGVKGLKMRRRST
LAQAEEEIEDEAAEAAELCRLLMDDDEADRKKKKKTKVTGEETRLVSKMQSKFAFDNAEQVKQITNSLQLDGNIPLKEDTITDL
REEENFGAKKSKSLKVNKAKKNDIAPISLPNKKIKLNMGEGIKNQVFKEKKPSRETFVCGACGQPGHMRTNKNCPKYGEDLET
QLESADMEKSSGKPISVDPSSHSQSKTASKKSSSKSNSKITPVDNSAKIPLKFKCGSTEKSSDKPVTETLQNSDKPVTSDSETAKSA
KVNKIIIPKKVKPDDTQAESRKHAVVIRPPTESSRGLPADSGRGPPTDTGRGQVDYHKLPIKIRPPTEEQSHKRIVIRRTKEVIDLEL
DSPGGNTGLQHRKTKRIVELSNFEKQKKQETVYGTGAFPKWNTKEDRRWWEEQEKRRNDARLREEDRARRHHKEEMRMLKE
QERLDEIKRFEEDIRREREEEERQKAKKKKKKKKPDLRDEYLDDPRARRHDKRMPERDRSGKRRSVAELGKLSADYMPPTKRR
RGGGGEVGLANILEGIVETMVKDRYELSYLFVKPVSKKEAPDYLDIIDTPMDLSRIRERVRNMEYKSREDFRHDVWQITFNAHK
YNDGRNPGIPPLADMLLEYCDYLLNENDDSLTSAEAGIETRDS

>VaTAF2

MAKPRKTKNNEDPKPENSGALVHHQKLCLSIDIDKRLVHGYTELEIAVPEIGIVGLHAENLGIESVWVDGEPTEFEYYPHQQQQV
EDDKRFSSVCSPSSAADAAVSVYMSSLEKELVPNLLINCCKPSKTESEQQQEQPVPENGFHSTAEPKQNVRIVRIDYWIEKAETGI
HYRNNLLHTDNQIRRARCWFPCIDDNSQRCCYDLEFTVAHNLVAVSTGFLLYQVLSKDNPPRKTYVYKLDVPVAARWISLAVAPF
EILPDHQFSLISHMCLMPNLSKMRNTVEFFHSAFSCYKDYLAVDFPFDSYTQVFIEPETAVSSLSLGASMSIFSSQVLFDEKVIDQTI
DTRAKLAYALARQWFGVYITPETPNDEWLLDGLAGFLTDFYIKKHLGNNEARYRRYKANCAVCKVDNGGATALSCSASCKDLY
GTQCIGLYGKIRSWKSVAVLQMLEKQMGPESFRRILQTIVSRAQDKTRSMKTLSTKEFRHFANKVGNLERPFLKDFFPRWVGSCG
CPVLRMGFSYNKRKNMVELAVLRGCTALQTSTTSTLDINPETENRDGDTGWPGMMSIRVYELDGMYDHPILPMAGEAWQLLEI
QCHSKLAARRFQKPKKGLKHDGSDDNGDVPSMDVRSNTESPLLWIRADPDMEYLAEVHFNQPVQMWINQLEKDKDVIAQAQ
AIAALEASPQLSFSIVNALNNFLGDSKAFWRVRIEAAFALANSASEETDFSGLLHLVKFYKSRRFDPDIGLPKPNDFHDFAEYFVL
EAIPHAVAMVRAADKKSPREAIEFVLQLLKYNDNNGNPYSDVFWLSALVQSVGQLEFGQQSILLLSSLLKRIDRLLQFDSLMPRY
NGILTISCIRTLTQIALKLSGFIPLDRVYELVKPFRDLKTLWQVRIEASRALLDLEFHCKGIDSALLLFIKYLEEEHSLRGQLKLATHV
MRLCQMRDGLNSEEEITSQTLVSMLNLLEGRTAFNNVFLRHYLFCILQILAKRPPTLHGVPRENRTLHMSLTEACNYQKNMFVL
DSDSKPLDLPSSTQNPTPNLGLDGLRDALNEASKDPPDEAPTQVPPTQVHIEALNEAPLEKAEEVYTEFPQEAPMEAPNEVSKEA
DTVSNSHERKRLIKIKVKQSSATSRADTDNQVVERSLGGRNEMDHGASSSVSVDAPQRNFPETLSMSNQNIDEVNSWHDRGSR
MTASIGSAKFLSDGDELVKELQCTADSSIVYSQPQPEDPSSSSIIQDNNVDADARRYASLQTLSVARFDPDGESLGKEISARGKEKH
KSKEKKRKRESNKGHHDDPEYLERKRLKKEKKRREKEMAKLQSDEAKRSSVDLSSKKEEPVVDVTRQIKSVEPSDGHNSKLET
KKIDSKPDPSEGTSGAPKIRIKIKNRMLNKS

>VaTAF4b

MDPSIMKLLEDDEDETMHSGVDVEAFQAALNRDIGGDVSASQFPGSDAVLSQGSNNTSSQSSSQWPTSNHDSQSDGQNQEPKT
AQEQHPSEMEPKQHGSLGEHLQHVASQDVNNIHLSQKQSQDDSHQAPAVQVPLHNSQTIGIHNSGKDSVLNKEVVKSHNPSSES
QYAKLQQMSNQQATVSEQPSSQVNRSNKQVPFGLLLPILLPQLAKDRAMQLQTLFTKLKKDEIQKDSFVRLMKGIVGDQMLRL
ALAKVQMQPQARPNQASAGQQLPVRMPTVSSGARQLNDPHALAQMHQRSMNVAVDQSRMSSSAGQTIDSNARKSQEFDVKIE
SQGLQPNQLTSSTSNSPAQETERASVHIQGLNKQQQHHLHFASAYGNSGGNYNPYSGTTSTSTSSIKPQSHDSHMSQIPHQGIGSN
HLGGSTHGLGVIGMPKLEQQNSFNDPKRLPGGSVSSAVNNAASQQTSNAWQPSTNKEQNLGLMSSVSYVKKEPGDLSTEQQNR
HNLSKLHGYSSVNSAQLEQSGASQGGLKDEFSRGLPASTSMPPTTSTGLLPHSSASASAVTHLDSSVLLSSQIPSNASGIAARPSLK
KSAATQKKPLEALGSSPPPSSKKQKTSGGYAEQSIEQLNDVTAVSGVDLREEEEQLFSGPKEDSRVSEASRKAVQEEEERLILQKA
PLQKKLIDIMAKCGLKGMSNDVEKCLSLSVEERMRGLISNLIRLSKQRVDFEKTRHRTVVTSDVRQQIMTINRKVREEWDKKQA
EAEKLRKLNDVDSNTGGDGDKDKDDGRAKSTKVNKEEDDKMRTNAANVAARAAYGGDDMLSKWQLMAEQAKQKREGGVD
VSSGSQPAKDVNRKSSSTSERNTKDNQEGEKRGSTPFLASSVARKLGKSHAMAPQTRVARSISVKDVIAVLEREPQMSKSPLIHRL
YEKIHSETPVEQG

>VaTAF5

MDEDQIEGCVSGYLKQKGFAQNDDQLQLSKADSSLQPDTLNRSQLERGSARYHNGYGRLRSWAYRSLESYKHELLRVLYPVFV
HCFMDLVAKGHLQEAWNFFNTFREDHEMLHSRDLQKLELVLSPTHLEEMEFAHSLRQSKFNIKICRYSYELLLQHLHSMQSTTII
GIINEHISFQVTAGQPSSISDDPEAVTLSGSIHDAVNQINQKEILWGMFEDSVEDHIDKAGALLSGTEKGEGEGKEGDNDESKKRSI
DVGKQGNSVKKVKKDKVSSATGKNAKPEANTISAAPRIKPEIPLPTVSTDVELSILDDLRNRVQLSSVALPSVNFYTFVNTHNGLS
CSSISHDGSLIVGGFSDSSLKVWDMAKLEQQSTAHFSQGGNDMSQNEQIIGQNSGRRQYTLYQGHSGPVYAATFSAAGDFLLSSS
ADKTVRLWSTKLNANLVCYKGHNYPIWDVQFSPAGHYFASCSHDRTARIWSMDRTKPLRIMAGHLSDVDCVQWHPNCNYIATG
SSDKTVRLWDVQSGECVRVFIGHRSMILSLAMSPDGRYMASGDEDGTIMMWDLSSGCCVTPLVSHTSCVWSLAFSCEGSLLASG
SADCTVKFWDVTTGIKVPRNEENRSGNANRLRSLKSLPTKSASVYSLQFSRRNLLFAAGAVAKSGC

>VaTAF6

MSIVPKETIEVIAQSIGISSLSPDVALAVAPDVEYRMRQIMQEAIKCMRHSKRTILTADDVDVALNLKNVEPIYGFASGGPLRFKRA
VGHRDLFYIEDKDVDLKEVIEASLPKAPLDTAVTCHWLAIEGVQPAIPENAPIEVISAPSDTKKHEQKDDDLPVDIKLPVKHVLSR
ELQMYFDKVAELTLSESDSALFKEALVSLATDSGLHPLVPYFTCFIADEVSRGLNNFPLLFALMRVVSSLLLNPHIHIEPYLHQLMP
SVVTCLVAKRLGSRLADNHWELRDFTANLVASICKRFGHVYSNLQYRLTKTLLNAFLDPKKAMTQHYGAIQGLGALGPNVVRL
LLLPNLETYMRLLEPEMLLEKQKNEMKRHEAWRVYGALLRAAGQCIYDRLKMFPTFSTPSPSAVWKTNAKVLTSSSRKREADP
DQLEQQPPLKKTATDGEVGVVPMNSSPAHKQEEAETRASPVDSIIGSSSSAQMKNETSLDGELRSNRGDTQASKTSAALTQVWK
DELNSGRILVSLFDLFGEGILSFIQAPEMYMFL

>VaTAF7

MEEQFILRVPPNVAERIERLLNETDPSSSEDKSLDLSFSEDGRSGTFMIGNEHFPASLLDLPCVVESYKTYDDNSLIKTADIGQMIM
VRESGDAAPDVIEYRHGLTPPMRDARKRRFRREPDLNPELVSRVEKDLLKIMARGTAENLDAEAAEQEVEENARGANKKVAPKP
APKHDVPENLTNAGEPDRSDSEESDDSV

>VaTAF8

MSNGGGKTGRQLEQPGPWRRRKVGDGDDFARGIAKIAVAQVCESEGFQAFQQSALEALSDVVARYIFNVGKSAHCHANLAGRT
ECHAFDVIQGLEDMASVQGFAGASEVDHCLESSGVIREIFHFVNEGEPVLFAHPIPRFPVVKKRVLNPSFLQKGEEPPGDHIPAWLP
AFPDPQNYSQSPVVNGRGTEPRAVKFEQERENGKGEWPVLNLKQQMVSNMFDKSALDPADTKAKRIAAEGNPFLAAPLKIEGK
EIASVPPAAKVFNDVVLDYPAVENFVENEPILALETFAPAIEAMKSTCYDSKEDQTKKFVNEKPIVRFKIGIKNKLLGRSIGLIPQTE
EHNNTLPWFAMEDEKDDRKRRAEKILRESLENPDQLVQL

>VaTAF9

MSDKDEELAMPRDAKIVKSLLKSMGVGDYEPPVIHTFLELWYRYIVDVLTDAQVYSEHAGKSEIDCDDVKLAIQSKLNFSFSQPP
PREVLLELAQNRNKIPLPKTIAGPGIPLPPDQDTLISPNYMFGIPSQGYGEPEETEDEETSIPNPSQEEKTDMQQQDPHQRVSFPLPK
CQKDYF

>VaTAF10

MNQNPQSSDGRGDDDTALSDFLASLMDYTPTIPDELVEHYLAKSGFQCPDVRLTRLVAVATQKFVAEVAGDALQYVYFTHCKAR
QATIPKDKRDKQQKDKRLVLTMEDLSKALREYGVNLRHQEYFADSPSTGMDPATREE

>VaTAF11

MKQSKDPFEAAFEESPPESPTEIEADTIHNHSHNHNHNQNPIPSPSVLPLNPGPPQKASTVVKNKDKDKDDEEEEEEDNMDVELS
KLPSTGDPHKMAKMQTILFQFSEEQMSRYESFRRAGFQRANMKRLLASITGTQKISVPMTIVVSGIAKMFVGEVVETARIVMKE
RKESGPIRPCHLREAYRRLKLEGKVFKRSASRLFR

>VaTAF12

MDSQAPATGTTPRSAAEPSQSQPPKSSPPILPSSTSSTPPVSAPTQSSPNPNPSSSPNPSPIQAPNPKPPTPTPAQPRPTQSFNRTLPPSQ
PQFPHFSSAPSPPSAPGGAPAPRGGMAIGVPAHHQSPSPPFSSSFGQHFGGLARTGVSVAEPTSNSSTSQVRTPVQGMGMLGPQMR
PSGIAAHQQRPVQSSLRPPSSAPNTQPGGSQSFQGHGIMRPSSVGSPATPSQGASQSVQSLNQPWLSSGPLGKPPLPSTAYRQQLNP
PSMQQRSHIPPQQQSTPTSSQQQQQQPPLSNQSQEHHGQQVQPSRASHHVPHQQQVSRLQGPGNQKPSSLVVAQTSVVQPVSQS
RLTNADTEEPCNSILSKRSIHELVNQVDPLEKLDHEVADILVDIAENFLESIIRSGCSLAKHRKSTTLEAKDILLHLEKNWNMTLPG
FGGDEIKSYRRQITSDIHKERLSAIKKSVTATEAAHAKGAGQASGSAKGNQAKTPMNIIGSPNLKSS

>VaTAF12b

MALTFRTLFLMLLAILSLLLFEDWVSTPSCNNTDNIIDNNLKVMMVADLLLSDSGFVNRFFRDYYMSKFFRKSFEVLRPDLLLVL
GDVSARGSELTRSKWVSVLRRFYRVLGPFVGLPFHAVLGDRDVGECGAVDVDRVSWIASKFPGLDSSGCAAFEIGNVSFVTLNS
MALLCGGSGGLRFEVEKVIERESVEVHMGTERVVKRVNGFGEFADADVLSGSGPVVLLHLPLDQTRNEQFGSVGDFEKYWTSS
MEGLNVVPESRIRGGLYKLLHTLPPNASEYILQALKPRIIFSAHRYTFSDHVHGDRTREISVPAMSWNARDDPGFVIASFQKAGRA
VSISHCSLARESQIVLVYISVMFLFCLMCLKGYLIFKWNRLVQEGVFGKKQYSETLFGGLSLIFSKFSTFANSVSVLCHSFDSPGFF
DFEERKRLFCLCCSKARFLTQIDSPAFRLPSIQLSSLRLPLHAHGRIIAPIDQSITSKSMAESASPSSKPSPMDPQNPAPSSNPTIPSPSH
NNMPSPSLPPLPQDQQQQQQQQQQQQQQQQNLHQQLSPPQQQQPLVSSQAMNTINGINPISNFQLQQTLQRSPSISRLNQIQPQQQ
AQQQQQFGVMRQQAGLYGGQMSFAAAGGGAGQQQQLGGSNLSRSALIGQSGHFPMLSGAGSQFNLLSSPRQKGGLVQQSQFS
GNSAGQSLQGMQAMGMIGTPNLTSQLRANGALAYAQQLRMGPGQIRQQMSQQSSLTGQVQGLPRSSSLAFMNSQLSGLSQNG
QPGMVHNSLTQQQWLKQMPAMSGPASPLRLQQQQRQPLASSTQLQQNSMSLNQQQLSQLIQQQKSMGQSQLHQQQQQHQPQ
QQLQQQLLHQQPQQQSQPQASVHQQQQSPRMPGTAGQKSFSLTGSQPDATASGATTPGGSSSQGTEATNQVLGKRKIQDLVAQV
DPQGTLDPEVIDLLLELADDFIDSTTTHGCILAKHRKSSTLESKDLLLHLEKNWDLTIPGYSSEEKKYQSKPQLNDLHKRRLDMIR
TMMESSASESNINSSKELSRQGISNPTPMGAHHLVRPMSSEQLVSQAAGSQMLQQMTRF

>VaTAF13

MSSSAAGTLSKPRAASSQPSETSSKRKRGVFQKELQHMMYGFGDDPNPLPESVALMEDIVVEYVTELVHKAQDIGSQRGKLSVE
DFLYLIRKDLPKLNRCTELLSMNEELKQARKVFESDEEKLRKVFEVDEAVEG

>VaTAF14b

MSHSQPLPLKRQGEHLSDDGASAIKPSRLKIAIPSEDSDKKNANNRIKDVEICVPIVYGTIAFYLGRKASESQSHKWTVYVRGAS
NEDLGVVIKRVVFQLHPSFNNPTRVVESPPFELSESGWGEFEIAITLYFHSDVCEKQLDLYHHLKLYPEDESGPQSTKKPVVVESY
NEIVFPEPSEGFLARIQNHPAVNVPRLPSGLNLPSPVQIDTMSDKERGDTKDHSLSQWFLNFSEADELLKLAAARQQVQAHIVKL
RRQLSLVEGLPQLSKPPSGYECT

>VaTAF15

MATHPGKQAPSNGSVYVCNLPYGTDENMLAEYFGTIGLVKKDKRTGRPKIWLYRDKETNEPKGDATVTYEDPHAAIAAVEWFN
NKDFHGNIIGVFIAESKNKDDQTYNSAGVEPVVAGTVVGLEETTKDVNGGSGRGRGQNDPSGKAWQQDGDWLCPNTSCSNVN

FAFRGACNRCGTARPAGASGISGAGGRGKGRAAGQEPGGVGRPVGGGLFGPNDWPCPMCGNINWAKRTKCNICNTNKPGHNE
GGVRGGRGGGYKELDEEEIEETRRRRREAEDDGELYDEFGNLKKKFRAKTQQAEAARGLPGSGRAGWEVEELGIDKDGRESRD
RGRERNDGESRSRERSDKERQSSWNRDRDRGRDRDRDWDYVDRDRDYGRDRDRSRHRH

>VaTAF15b

MLHCTGTTSRPLFTVSTIPYRIPIMSGNYDQDGGGYGRNDGGGYGGRGGGGFGGRGGYQGGDRGGRGSGRGGGSGRDGDWR
CPNSSCGNLNFARRVECNKCGAPSPAGANDRGGGGGGYNRGGFGNSRGGRSGNYDGGRGNGYNSSRGNNNVGRSGGGNRGS
QGREDGGYGQVPAPAAQSYGGAGGNYPPAYNSSGGSSNYETDAVPPPASYAGGPASYPPPHGSNAGGYGGGDSHNGGRSGQPV
GYDSGYGAGSQGGFGGAPDEPPAKVKQCDENCGDSCDNSRIYISNLPPDVTIEELRELFGGIGQVGRIKQKRGYKDQWPWNIKL
YTDEKGNNKGDGCLVYEDPSAAHSAGGFYNNYDLRGYKIAVTMAEKSAPKAPPAYNHGGNRGGYGGDRRRDNYRDAGGSGP
DRRDNYGGNRSRPY

IV. Protein sequences from *Oryza sativa*

>OsTBP1

MAAAAVDPMALGLGTSGGGGGGESAVGGDGAEPVDLVEHPSGIVPTLQNIVSTVNLDCRLDLKKIALQARNAEYNPKRFAAVI
MRIRDPKTTALIFASGKMVCTGAKSEDHSKLAARKYARIVQKLGFPAKFKDFKIQNIVGSCDVKFPIRLEGLAYSHGAFSSYEPEL
FPGLIYRMKQPKIVLLIFVSGKIVLTGAKYRKEIYAAFENMFPVLTEYRKTQQR

>OsTBP2

MAAEAAAALEGSEPVDLAKHPSGIIPTLQNIVSTVNLDCKLDLKAIALQARNAEYNPKRFAAVIMRIREPKTTALIFASGKMVCTG
AKSEQQSKLAARKYARIIQKLGFPAKFKDFKIXNIVGSCDVKFPIRLEGLAYSHGAFSSYEPELFPGLIYRMKQPKIVLLIFVSGKIV
LTGAKVRDETYTAFENIYPVLTEFRKVQQ

>OsTAF1

MGDGERREDENPTTSAADDDDDEDYDEPGGGNHFLGFMFGNVDDSGDLDADYLDEDAKEHLFALADKLGPSLKDIDLIKPSA
APTDPSEQDYDAKAEDAVDYEDIDEEYDGPEVEAATEEDHLLSKKDYFSSNAVYASVNSKVSVFDEENYDEDEEPPNDNDLPSD
NIVQNCTSASAEQLDMAPSNDNLAVEKMSSSLSEPEESFESEAFQKEMVAEEQLESKTATSLPVLCIEDGSVILKFSEIFGAQEPVR
KAKMDRHKRPVNKELQITNFTDIVEEDEEVFLRSTIQNLSALKHIKTNDNFVESDSDESTSDVALRLKDSCLSEQPMKDKDIPTAV
QSPVFPDFYPLEHENWENDIVWGNSPTTAIQPCLTSCAISKESLDDHNEDQAEGYVSGCWDVQNKFHSSSVMADPFGHTEIPDST
SYRSPENSYSPLRKETAQENNSLDEPNNITQPVKIDTTRHLNKLSLLNKELLEGSWLDNIVWDPSEDVPKPKLIFDLKDDHMLFEI
LDEKNGDHLRSHARAMIVTRPMKTSAVENVDHNNQAIALSGRFNISNDKFYSNRKMSQQARSHAKKRATMGLKLVHSVPAQK
LQTMKPKLSIKEIANFHRPKAKWYPHENKLTARFQGDECSHGPMTAIVMTLGGKGVKFLVNAEETPLSVKSKASKKLEFKPSEKI
KLFCSGKELQDDISLAMQNVRPNSILHVVRTEIHLWPKAQRLPGENKPLRPPGAFRKKSDLSVKDGHVFLMEYCEERPLLLANA
GMAARLCTYYQKTSPSDQTATSLRSNSDGLGTMLAIDPADKSPFLGNIRSGSHQSCLETNMYRAPVFPHKVATTDYLLVRSPKG
MLSLRRIDKLYAVGQQEPHMEVFSPGTKNMQNYILNRILVYVYREFRAREKPGIIPQIRADELPIQPPITEAIVRKRLKHCADLRKG
PKGHLFYIQRPDFRIPSEEELRRLLTPENVCCYESMQAGQYRLKHLGIEKLTQPVGLASAMNQLPDEAIELAAAAHIERELQITSW
NLTSNFVACTNQDKENIERLEITGVGDPSGRGLGFSYVRVTPKAPVSNSTHKKKSAAAKGTTVTGTDADLRRLSMDAARELLLK
FGVPEEQIDKLTRWHRIAMVRKLSSEQAASGVTMDEIPVSKFARGQRMSFLQLQQQTKEKCQEIWDRQIQSLSAMDGNENGSDT
EANSDLDSFAGDLENLLDAEEFDDEDVGNTDIRSDKMDGMRGLKMRRCHTQSQINEEIQDDVAEAALVEKLLEESDSDMKRKK
QPVETTNYSTPMYNQGNKMKQGKAGQMIKSSVYAGALTPKESIPREAKEVENFAEGSLPSKLRTKTGFDANDDIILVKRKNIPGK
DGFKEKRQGARGDTLVCGACGQLGHMRTNKLCPKYGEDPETSEMDVNSIRSHPPDIVSNAQIKTSNKRLVAKVSSEAFETEGPES
IEKAKPVPVKFKCGAPEKSLDRNMSISASLVSDKRMMDATDSKSTGKVNKIKISNKIKYDDYPPDTPKPSVVIRPPAEVEKDLPRK
KIIIKQPKVLGDQQRPTELRSGQEPRKTRKIVELSSFEKRDREDDNGFSGQPIQINSSHDRGWGLVGKRSKGIMESSESWRAFEEQ
RERQEQRLIEARIYDARREDELQKAKKKNKKKKHEFRDDDLLDPRPYKNDRRVPERGRAAKRRTPADMTEYTPPAKRHRGGE
VELSNILEKIVDHLRTMSCSFLFRKPVTKKEAPDYFDIIERPMDLGTIRDKVRKMEYKNREDFRHDVAQIALNAHTYNLNRHPHIP
PLADELLELCDYLLEESADVLDDAEYAIED

>OsTAF2

MAKARKQKGEEQKPDGGGAGGGGGGATVLHQKLCLSIDMENRLIYGYTEIKVQAENDTFALHADNMTIRNILVDGQAAEFEYS
PQWKNAGDQQSWSSVSCSKTAADAACSVYISSLNSEAAPNLIISSERSSKAITEPQYEENGENHEENGEKHEENGEKQNENGEKC
EENGGKPAQISDDQAVNGCNGSADKKDKEEETEKDNEKEKEDKEEETEKDNEKEKEDKEEETKKDNEKEKEQLMGTDEKEKE
KEKEDENEEEKLEEEEKKDKEEKLEEKEKENEEENGNEKDKENDNEIEKVKNTKLVHIDYILEKAETGLYFTGNILHSNNQIRRA
HCWFPCIDSATQRCPFDLEFTVSTNLVAVSNGDLLYQVLSKEDPPRKTYVYKLSTPVSAQWISLVVGPFEVLPDRNDISVSHMCLS
QSLSKLENTISFFHSVYSCYEDYLAASFPFGLYKQVFLPPEMIVSPTSLGASTCIFNSDILHDEKVIDQIIDTRIKVAYALARQWFGIY
TNAEEATDEWLLDGLAGFLTEHFVKRYLGNNEARYRRFKANCIVCEFDVSGATALSSPSASSDLFGTQTIGSYGKIRSLKAVSVLQ
MLEKQMGPDSFRKILQMIVAPTRASRTLSTKEFRHLANKVGNLERPFLKEFFPRWVESSGCPVMRLGISYSKRRNLVELAVSRGC
TTKVDPGPDIRTNGDSREGDTGWPGMMSVRVHETDGVYDHPIVPMAGEALQVVEIQCHSKVAAKRFQKTKKGSKPDGSDENID
ASNQDNRASMDAPLLWIRVDPEMEYLAEIHFHQPVQMWINQLEKDKDVISQSQAISVLEKSPQLTFAVTNALNNFLNDTKAFWR
VRVEAAYALAVTASEGTELTGLLHLVKFYKSRRFDADIGLPRPNDFHDIPEYFVLEAIPHAVALVRSADKSSPKEAIEFILQLLKYN
DNNGNVYSDVYWLSAMVQAIGELEFGQQGVGLLSSLLKRIDRLLQFDNFMPGYNGVLTVSCIRTLARIAQRVSSSICLDRVCELI
VPYRNMDKPWKVRMEAGRVLIDLEFHHKGLDAALLLFLKYANEERSLRGGTKLAVHVLRLCQANIESHDNNQIQLPTLVGLLC
LLAGKKAYNNVYLRHNVFCILQIAAGRSPTLHGVPKVVTPPQVVLEISSDQHTKADSSVPQQSRPQEPSTSTPSVREVLPTSGPLK
DADNISNCSERRNVIFIPTKDADNISNCSERRNVISIPTKDADNISNCSERRNVVKIRVKRASSSSKADDADHRDHSHGRNENEAG
PCSSMSVDAPMTEAPEPVNVSNHNIEEQNSCHDREQNSCHDRESRMSASIGNVKLMDKHEVSKELQCTADSRLDALPKDHFSPV
VNGQEVLDKPRSQLEVVSTSYDGNQAPDSMNGLETKEKKKDKKDKKRHRDKKDDPEYLEKKRLKKEKKRMEKEKGKKQKE
GEGVSSSEQKNTAKPSDSQGTSSARPPAPMRIPEPKISNVGTPVDTTRTLTTTKIRIKVKPLQR

>OsTAF4b

MSTQLTEHDHQPEQEPPHSENHLKQAEPNSFQFAEKETGYAGLQNFTGPKVDVGQTSGEQQHVKQMVGQQAPPGAQDARKRG
YQPSIPFNMLIPILQAHLDRDKDMQLQTVWAKLRRNEVHKDDFLRVIRNIVGDQMLKQAAHKVFAQMQAQAQRSGQANANQQ
ANANQYSLQSQVSSSGSAQLHDQQVHVSTTPNQGQKNQALSSSQTFVQSGTQVQSSMTAHDNSIQRPDAKGMHVTPNRPPVM
NSAISAQTMNKQQQPTQVQQASQQIYGTTNRPDQPYTRPIGGSTPLSSLSSESEIRPSSHPAKMEILPSHPMTQQNAAAQQMQQN
KDVKTNASNPRSNAKQDSGTGKGRAVGTGGSSTKSQGKQGPPNFSTPPAAKSNKKTAGQKKSLETSGSTPPPPSKKQKTSGTFQ
EQSFDQLNDVTAVSGVNLREEEEQLLSAPKEESWASEEARKIAQEEDGKLFLQKGPLLKKLAAIVPKCNLKSIGGDVEHCLSMCV
EERLRRFISTLIRVSKQRIDTEKSGHQLVITSDVGRQILRMNQKAKEEWDKKQAEETDKNKKQNEVDGGGTVELDKEKEETRSK
NAKPNKEEDDKMRTTAANVAARQAVGGSDMLSKWQLMAEQARQKREGLDLAASSQRGTASRSHMAGKGPTDHHEASKRTHS
AAFGTGGMNRQGRGPFAASHPKGPQRTISMKDVICVLEREPQMTKSRLIYRLYERLPGDSTRD

>OsTAF5

MEDEEMEKKVQQYLQRKGFRLTELALQEERNRISTSSVSDVALARSENDPARYYDGYSKLRTWAYSSLDQYKHELLRVLYPVFI
HSFMDLVAEGHTQEARSFFHTFREDHELMHSRDLQKLEGILSPSHLEEMELARSLRQNQFRIKLCEYSYELLLQYLQKTQALVVL
GIINERTTFDVSPGQPSLISDDTDVVALVGTKKDLAKQINLKEVHWGLLEDSVEERMEKTLLESDKTEAESKDADAEDNNKRKSS
EGGKQGGSVKKVKKDKIAGATGKTNKSETSIVSVAPRVKPELTLPVIPVEVEQSILEDLRNRAQLNSLALPSVSFYTFLNTHNGLN
CSSISHDGSLVVGGFSDSSVKVWDMSKIGQPPKTSSPQGENGLSQGERTSASDYGKRPYTLFQGHSGPVYSAAFSPFGDFLLSSSS

DSTIRLWSTKLNANLVCYKGHNYPVWDVQFSPVGHYFASASHDRTARIWSMDKIQPLRIMAGHLSDVDCVQWHVNCNYIATGS
SDKTVRLWDVQTGECIRMFIGHRSMVLSLAMSPDGRYMASGDEDGTIMMWDLSSGRCVSPLGGHSSCVWSLAYSCEGALLAS
GSADCTVKLWDVASSTKVLKTDDTSTNRLRMLKTLRTKSTPVYTLRFSRRNLLFAAGALSLGS

>OsTAF6

MSIVPKETIEVIGQSVGIANLPADVSAALAPDVEYRLREIMQEAIKCMRHAKRTVLTADDVDSALSLRNVEPVYGFASGDPLRFK
RAVGHKDLFYIDDREVDFKEIIEAPLPKAPLDTAVVAHWLAIEGVQPAIPENPPVDAIVAPTENKRTEHGKDDGLPVDIKLPVKHV
LSRELQMYFDKIAELTMSRSETSVFREALVSLSRDSGLHPLVPYFSYFIADEVTRSLGDLPVLFALMRVVQSLLHNPHIHIEPYLHQ
LMPSIITCMVAKRLGHRLSDNHWELRDFSANLVGSVCRRFGHAYHNIQTRVTRTLVQGFLDPQKSLTQHYGAIQGISALGPSAIRL
LLLPNLETYMQLLEPELQLDKQKNEMKRKEAWRVYGALLCAAGKCLYDRLKLFPNLLSPSTRPLLRSNKRVVTNNPNKRKSST
DLSTSQPPLKKMTTDGAMNSMTSAPMPGTMDGFSTQLPNPSMTQTSSSGQLVESTASGVIRRDQGSNHTQRVSTVLRLAWKED
QNAGHLLSSLYEVFGEAIFSFVQPPEISFFL

>OsTAF7

MEEQFILRVPPSVAERIERLMNESAAASSSSNPEDASLDLSFSEDGRNGTFMIGNESFPASLLDLPTVVESYKTYDDSVLIKTADIG
QMIMVREEEDPAPEGVEYKHGLTPPMRDARRRRFRREPDLNAELVHRVEKDLISIMHGVSINQNASAILRAGEGGDRKKAGPAPA
TKPNVKQPAANGEEAEAERSDSDESVDP

>OsTAF8

MIGARPSAKASMSSRGGGGGGRAGRVGGGGGGAGGGGGGGGGGAAAAAAGGGGDFGRAVARAAVARMLEAAGFVCAHRS
AVDALVDVLLRYICQLGRAATFHANLAGRAAANECDVIQFLEECGAAYYGFAGAASVSARCLANSAVVKDMAVFVGASKESPF
AGRPLPRFPVQRVPLHSTTSFAVLGRESGMSHVPEWLPAFPEPHTYVRSELWSEEVAKAGADEVERARQRRKAEKSLLSLQRRLA
LAGADGFRPGMLVDDAVKANGLDVVESKANPFHERALPYGEKAVSEVTMPGVGKTFSVVEAFAPAFEESKGGEFDEGMDQGQ
NDSQTQKRVVPKERPPVYFRIGIDKKSMVMALNSRALVELKDPWFFKEDKEQRAELILREAMDNPHELTQL

>OsTAF9

MDPGGLRPAPQSAAAAAAAAAAGAGAGASAADEPRDARVVRELLRSMGLSEGEYEPRVVHQFLDLAYRYVGDVLGDAQVYA
DHAGKPQLDADDVRLAIQSKVNFSFSQPPPREVLLEVARNRNKIPLPKSIAPPGSIPLPPEQDTLLSQNYQLLAPLKPPPQFEETED
DNAGANPTPTSNPSNPSPNNLQEQQQLPQHGQRVSFQLNAVAAAKRRGTMDQLNMG

>OsTAF10

MMGSNSAGGGGGAMVPGGMGGGPMGAAAAGGGGGDGRHDDEAVLTEFLSSLMDYTPTIPDELVEHYLGRSGFYCPDLR
LTRLVAVATQKFISDIASDSLQHCKARVAAPIKDNKSKQPKDRRLVLTMDDLSKALQEHGVNLKHPEYFADSPSAGMAPAAREE

>OsTAF11

MKDPFEAAVEEQESPPESPAANEEDAAGAPEGYDGASGSRGPPLRLPPSRAAPSGSGGAAAAAARGKVVRVQKEQQEEEDDEE
DHMEVDLDKLPSGTSDPDKLAKMNAILSQFTEDQMNRYESFRRSGFQKSNMKKLLASITGSQKISLPTTIVVSGIAKMFVGELVE
TARIVMTERKDSGPVRPCHIREAYRRLKLEGKIPRRTVPRLFR

>OsTAF12

MDAPPPAQPDAAAAAPAAPPTSTSASAPSSAPQPNPTPSASTAAPPTPDTTLAPAPNPTPAPVQTLETPAPSPASARPPVPRMRPPY
THLASPITMSSSPATGAASSSSASAPAATSASSSAMPRGGVALGLPAHPRAPQTPVGYTGFVPPPTLAAQFGSMHRGPDQPPPSSTQ
PRQPSPGIQNIGTVGSINTSQVRPGAISSLPQQTRPNFPSSTAPSPSDSQIASSQKTPIQALARPPSMASSPSMPLQQTPPNVSAPLRP
PQHRPHPRPYHAPAISHPQNALLTQQQQKLPQHQHLQQQQQQQQQQKLQQQQQQQQKLQQQQQQQQKLQQQQQQQQN
QPQHSSQQSQQTTTLRNQQQISQQQTARTPVSMAQKLDSPAVLKATNVQSGDMASVDVDAGGSGNRLLSKRSIHELVAQIDPSE
KLDPEVEDVLIDIAEDFVESVATFACSLAKHRKSSILEAKDVLLHAERSWNITLPGFSGDEIKLYKKPHVNDIHRERLTLIKKSMAS
ESNAKGSAAQAAANQKNQTPKPPATGSP

>OsTAF12b

MIPWGQPGSDPKDSNHPPPDSTTKRLRAHVKLPHRTRTCPRTPRRNRPPRVVSGRRPPPPEPRRPQPAMADPPSAAATASPQPDQL
AAAAVSTPQNPNPNPLLSPQIPPSPTVSDLSAISSPQLDPSAAGGGAMDYPPRPPQMQAPSPGQAAAGAGGFGQIHRSGSGSRLAA
VGQLPQYAAAAARMYGSQVNFSGGGGQVGQQQQQQQQLAARAAMLSQGQIGMLQGQGNAASAAHYGLQSQMMAQPRQKG
MVQGAQFNTANAAQALQGMQSMGVMGGMRGNGTIPYNQQRFAHAQAQLRPQQTSQQGTLSPQKVVGQGLTRTASIAALNPQ
LPGSSTNGPMAQMSLPQKQQQAAWLKQMQSSLGSPVSPQQFQHQQRMLLIHQLQQQSGLNQHQIAQTQQQHPHLNTQLLQQQ
HILQQLQQQQQSPRISASGSQKSMNLTGSQPGTPLSGGTMTGGSASQGAEVTNQLLGKRKIQDLVSQVDPLGKVDPEVEDLLLEI
ADDFIDSVTAFACTLAKHRKSSVLEAKDVLLHLEKNWHLSVPGFLREDKNPQRHPVKVSVDPQQPECDAAGIRSTGNKLVINNS
VANHQTRPPVTEPSPMPTMGPLSKVPRF

>OsTAF13

MQNPGGHHASPASAAKSKSSTAAAASASGQGSSHHHHHHHSGGGGGGGGGGADASATTLKRKRGVFQKDLQHMMYGFGDDP
NPLPETVALVEDIVVEYVTDLVHKAQNVASKRGKLLTEDFLYLIRKDVRKLHRATELLSMNEELKQARKAFDVNEETLATNNE

>OsTAF14b

MPQASSSSSPATAAAPPPPQPAADPSPSAVPASEEALDPQTPAPPPQAQPEAVLTAAQKALRSKPTRPPEDSDKKNKKLKDVEISFPI
VYGTISFWLGKKASEYNSHKWTVYVRSATNEDLSVIVKRVVFQLHPSFTNPTRVVEQPPFELSESGWGEFEIAITLYFHSDVCEKR
LDLFHQLKLYPEEDTGPQSTKKPVVVETYDEIVFPEPTEAFFQRVQNHPAATVPRLPPGITLPPPGPMELVPHEKKRGDTKDHPLS
QWFSNFSEADELLKLAAARQQVQAHIAKLRRQLSMIDGMPQQSKAVSVQGQQFGHG

>OsTAF15

MAGYMSRGPPNGSVYVCNLPPGTDETMLADYFGTIGLLKKDKRTGRPKIWIYRDKVTNEPKGDATVTYEDPHAASAAVEWFN
NKDFHGSTIQVHIAESKNKDTYDNSASLNNSAGLGGQDELDNGAGRGRGHGDGPGKAWQQDGDWLCPNTSCGNVNFAFRGV
CNRCGAARPAGVSGSGAGGGGRGRGRGSDDAKGGSRAAAVGGPPGLFGPNDWSCPMCGNINWAKRMKCNICNTTKPGHNEG

GVRGGRGGGYKELDEEELEEVKRRRKEAEEDDGEMYDEFGNLKKKFRAKTQQTENAPTLPGSGRAGWEVEQRGSTRRESRER
SRDRGRDHDYNERDSRNRDRGSHGRERRRSRSRSRDREKERGRDRGRDHSYERSWERGAERDRDRYR

>OsTAF15b

MSGSYGSDDYRGGYGGRGRVGILRVVCVLASGSAPDWLVVFFVGGGGGGRGRGGGGGGGGGYGGGGVGGGYGGGGGGYG
GGGGGYGGGGRGGGGGGGYGGGGGGGRGGGGGGGGRGGGGRGGGRDGDWVCPDPSCGNVNFARRTECNKCGAPSPAGGG
GGGGGGGYNKSGGGGGGYNRGGGDFSSGGGGGYNRGGGDYNSGGRGGGTGGGGRGGGYNRGGGDDRGFDDHRGGRGGY
GGRDQGNNQRGDESGYDAGSYGQVPPQGPPSYGGPGGDYAAPPSSYGGNNAYNSDSAVPPPSSYGGGPGSYPPSYGAPPPNPPY
SGGAPGGQGSLPPSYDGGYGGRPMPGGGGPGAPPPYHGGGGGGGGGGGGGGGYTGSAAPEPAAKVKQCDANCDETCDNARIYI
SNLPPDVTVEELQELFGGIGQVGRIKQKRGYKDQWPWNIKIYTDDSGKNKGDACLAYEDPSAAHSAGGFYNNYEMRGYKISVA
MAEKSAPRAPAYGHGGGRGGYGGGRRDNFRDGGGHGPNRHQGGGSRSRPY

V. Protein sequences from *Glycine max*

>GmTBP

MADQGLEGSQPVDLQKHPSGIVPTLQNIVSTVNLDCKLDLKTIALQARNAEYNPKRFAAVIMRIRDPKTTALIFASGKMVCTGAK
SEQQSKLAARKYARIIQKLGFPAKFKDFKIQNIVGSCDVKFPIRLEGLAYSHGAFSSYEPELFPGLIYRMKQPKIVLLIFVSGKIVLT
GAKVRDETYTAFENIYPVLTEFRKNQQ

>GmTAF1

MGYDSDSPSQDGRDEDDEEEYEDSGKGNRFLGFMFGNVDNSGDLDVDYLDEDAKEHLSALADKLGPSLTDIDLSGKSPQTPPD
VVEQDCDVKAEDAVDYEDIDEEYDGPETEAANEEDYLLPKKEFFSSEASVCLESKASVFDDENYDEESEKEQDFVNDDSKVYNI
PLAGEQEESFVDASKEESSLEHELHVDSPQTEELDADVQKLEEDGPEVQKRSMAMPLPVLCVEDGVAILRFSEIFGIHEPLRKGEK
REHRHSIPREEDEEEFLKGFSQSLSLSKQVCVVHNDVSESNDVDLEFPKFGFLHADASVDRKDDQQSKDSCHSAEPMKGDFVED
HFWKDHPFMLANFYPLDQQDWEDKILWGNSPVPSYNNVESCEISGPELGASGGSEIEIESGIHNIQMEPQKVLEDKNHNVLMRSS
PVKLEPFGSRDSSGAKTNLISRSLFHPQLLRLESRSEVDSSSLADGRDAEISEHNQSGQVKRFTKVISQNRDMMEGSWLDKIIWEE
LDQPTVKPKLIFDLQDDQMHFEVLDTKDGTHLCLHAGAMILTHSLKLSSGDSSELPGHGSQYGWRYVANDKHYSNRKTSQQLK
SNSKKRSAHGVKVFHSQPALKLQTMKLKLSNKDIANFHRPKALWYPHDNEVAVKEQGKLPTQGPMKIIIKSLGGKGSKLHVDVE
ETLSSVKAKASKKLDFKVSETVKTFYLGRELEDHKSLAAQNVQPNSLLHLVRTKIHLWPKAQRVPGENKSLRPPGAFKKKSDLS
VKDGHVFLMEYCEERPLLLSNVGMGARLCTYYQKCSPDDQSGSLLRNTDSRLGHIISLDPADKFPFLGDLKPGCSQSSLETNMY
RAPIFPHKVPLTDYLLVRSSKGKLSLRRIDKINVVGQQEPLMEVLSPGSKNLQTYMMNRLLVHMCREFQAAEKRHLPPYIGVDEF
LSQFPYQSEASFRKKIKEYANLQRGTNGQSILVKKRNFRIWSEDELRKMVTPELVCAYESMQASLYRLKHLGITETHPTNISSAMS
RLPDEAIALAAASHIERELQITPWNLSCNFVACTSQGKENIERMEITGVGDPSGRGMGFSYARAPPKAPVSSAMVKKKAAANRG
GSTVTGTDADLRRLSMDAAREVLLKFNVPEEVIAKQTRWHRIAMIRKLSSEQATSGVKVDPTTISKYARGQRMSFLQLQQQTRE
KCQEIWDRQVQSLSAVNGDENESDSEGNSDLDSFAGDLENLLDAEECEEGEEGTNDLKRDKGDGVKGLKMRRRPTLAQAEEEI
EDEAAEAAELCRLLMDDYEADRKKKKKAKVMVGEARLVPKMQSKFSFDNAEQVKQITNTLQLDGTNHLKEDAITDLREEENV
PAKKSKSLKVNKAKKNDIMPISIPNKKIKLNMGEGIKNQVFKEKKPSRETFVCGACGKAGHMRTNKNCPKYGEDLETQLESAD
MEKSSGKSSFVDPSSLSQHKAPSKKSMSKSATKVAPVDNSTKIPLKFKCSSTEKSSDKPAVETLQSSDKPVTSDSETAKSAKVNKII
IPKKVKPDDTLAESRKHAIVIRPPTDSGRGQVDSHKFPIKIRPPTEIDREQSHKKIVIKRTKEVIDLELDSPGGNTGLQHRKTKRIVE
LSNFEKQKKQETVYGTEGFKKWNSKEDRRWREEQEKWRNDARLREEDRARRHHKEEIRMLKEQERLDEIKRFEEDIRREREEE
ERQKAKKKKKKKKPELRDEYLDDPRARRHDKRMPERDRSGKRRSVTELGKIGADYMPPTKRRRGGGGEVGLANILESVVDTI
VKDRYDLSYLFLKPVSKKEAPDYLDVIERPMDLSRIRERVRNMEYKSREDFRHDMWQITFNAHKYNDGRNPGIPPLADMLLEY
CDYLLNENDDSLTEAEAGIEIRDF

>GmTAF2

MAKPRKPKNNEDPKPENSGAVVHHQKLCLSIDIDKRQVHGYTELEIAVPEIGIVGLHAENLGIESVWVDGEPTEFEYYPHRQQQA
EDDKRFSSVCSPSSAADAAVSVYMSALEKELVPNLLINCCKPSKAESEQQQERQPASENGFHSSAEPKQNVRTVRIDYWIEKAET
GIHFRNNLLHTDNQIRRARCWFPCIDDNSQRCCYDLEFTVAHNLVAVSTGSLLYQVLSKDNPPRKTYFYKLDVPVAARWISLAVA
PFEVFPDHQFSLISHMCSPPNLSKMRNTVDFFHSAFSCYKDFLSVDFPFDSYTQVFIEPEMAVSSLSLGASMSIFSSQVLFDEKVID
QTIDTRVKLAYALARQWFGVYITPEAPNDEWLLDGLAGFLTDFFIKKHLGNNEARYRRYKENCAVCKVDNDGATALSCSASCKD
LYGTQCIGLYGKIRSWKSVAVLQMLEKQMGPESFRRILQTIVSRAQDKTRSIKTLSTKEFRHFANKVGNLERPFLKDFFPRWVSSC
GCPVLRMGFSYNKRKNMVELAVLRGCTTLQTSSTSILDINPDTETRDGDIGWPGMMSIRVYELDGMYDHPILPMAGEAWQLLEI
QCHSKLAARRFQKPKKGLKLDGSDDNGDVPSMDMRLNTESPLLWIRADPDMEYLAEVHFNQPVQMWINQLEKDKDVIAQAQ
AIAALEASPQLSFSIVNALNNFLSDSKAFWRVRIEAAFALANSASEETDFSGLLHLVKFYKSRRFDPDIGLPKPNDFQDFAEYFVLE
AIPHAVAMVRAADKKSPREAIEFVLQLLKYNDNNGNPYSDVFWLAALVQSVGELEFGQQSILLLSSLLKRIDRLLQFDSLMPSYN
GILTISCIRTLTQIALKLSGFIPLDRVYELVKPFRDLKALWQVQIEASKALLDLEFHCKGMDSALLLFIKYIEEEHSLRGQLKLATHV
MRLCQMRDGLNSNDEITSQTLVSMLNLLEGRIAFNNVSLRHYLFCILQILARRPPTLHGIPRGNRMLHMSLAEACNYQKNIFALD
SESKPLDLPSSTKNLTQNLGPTMEGLRDAVDEAPKDQPCEASTQVHLEALKEASLEKPKEVFTEFPQEAPIEAPNPNEVSKEVDT
VSNSHERKRPIKIKVKQSSATSRADTDNQVVECSLGGRNEMDHGASSSVSVDAPQRNFAETVSISNHNIDEVNSWHDRGSRMTA
SIGSAKFLSDGDELVKELQCTADSSIVYSQPQPEDPSSSSIIQDNNIDADARRYASLQTLSVARFDPDGESLGKEISARGKEKHKSKE
KKRKQESNKGHHDDVEYLERKRLKKEKKHREKELAKLQSDEAKRSSIDLSSKKVEPVVDVARQVKSVEPSGYNSKVEIKKIDT
KPEPSEGTSGAPKIRIKIKNRMLSKS

>GmTAF4b

MILARNFGMGSQYHKFITMKLNSIVVLSRSNRDNECGEVAMDPSIMKLLEDDEDEAMHSGVDVEAFQAALNRDIGGDVSTSQF
SGSDAVLSQGSNNTSSQSLSQWPTSNHDSQTDCQKQESKTAQQQDQPSSGVELKQRGSLAEQLHHVASQDINNPHLSQKQSQDE
CHQAPALQVSLHNSQAIGIQNSGKDPVLNNEVVKNHNPSSESQYAKLQQMSNQQATVSEQPSSQGNRSTSKQVPFGMLLPILLPQ
LAKDRAMQLQTLFAKLKKEEIPKDSFVRLMKGIVGDQMLRLALAKVQVQPQTRPNQASAGQQHPMRMPTVGSGASQLNDPH
ALAEMHQRSMNAAVDQSRMGSSAGQTMESNARKSQELDVKIESQGLQPSQLTSSSSNKIAQETERTSVHIQGLNKQQQQHLHFP
SAYGNSGGNYNPFSGTTSSSTSSIKSQSHDSHMSQISYQSIGSNHHLGGSTHGLNVIGMSKLEQQNSFNDPKRLPGGSVSPAVNNT
VSQQTKNAWQPSTNKEQNLGLLSSVSYVKKEPSDLSTEQQNRHNLSKLHGYSSVNSAQLEQGGASQGTVKDEFSRGLPAPPSKP
PTSTGLLPQSSSSPSVMTQLGPGVSLSTQIPSNASGIGARTSLKKPAAAQKKPHEALGSSPPPANKKQKTSGGSVEQSIEQLNDVTA
VSGVDLREEEEQLFSGPKEDSRVSEASRKAVQEEEERLILQKAPLQKKLIDIMAKCGLKGMSNDVEKCLSLCVEERMRGLISNLI
RISKQRVDFEKTRHRTVVTSDVRQQIMTINRKVRKEWDIKQAEAEKIRKLHNVDSNTGVDGDKEKDDGRGKSTKVNKEEDEK
MRTNAANVAARAAYGGDDMMSKWQLMAEQAKQKREGGVDVSSGSQPAKDVNRKSLSTSGRSTKDNQEGEKKGSSTFIASSV
ARKLGRSHAMASQTRVARSISVKDVIAVLEREPHMSKSPLIHRLYERIHSDAPV

>GmTAF5

MDEDQIEGCVSGYLKQKGFTQNDDQLQLTNTDSSLQPDTLNRAQLERGSARYHDGYGRLRSWAYRSLDSYKHELLRVLFPLFIH
CFMDLVAKGNLQEAWNFFNTFREDHEMMHSRDLQKLELVLSPTHFKEMEFAHSLRQSKFNIKICGYSYELLMQHLHSMQSTTII
GIINEHISFQVTAGQPSSTSDDPEAVSLIGNIKDEANQINQKEILWGMFKDSAEDCVDKTGSLLSDTEKGEGEGKEGENDEIKKRSI
DGGKQGSSIKKAKKDKAGSATGKNAKPEANTVSAAPRIKPELPLPTFSTDVELSILEDLRNRVQLSSVALPSVNFYTIVNTHNGLS
CSSISHDGSLIAGGFSDSSLKVWDMAKLEKQPTTSFSQGGNDTSQNEQNIGQNSGKRLCTLFQGHSGPVYAATFSPAGDFILSSSA
DKTIRLWSTKLNANLVCYKGHNYPIWDVQFSPAGHYFASCSHDRTARIWSMDRIQPLRIMAGHLSDVDCVQWHVNCNYIATGSS
DKTVRLWDVQSGECVRVFIGHRSMILSLAMSPDGRYMASGDEDGTIMMWDLSSGCCVTPLVGHTSCVWSLAFSCEGSLLASGS

ADCTVKFWDVTTGIKVPRNEENRSGNTNRLRSLKSLPTKSASVYSLQFCRRNLLFAAGAIAKTG

>GmTAF6

MSIVPKETIEVIAQSIGINNLSHDVALAVAPDVEYRMRQIMQEAIKCMRHSKRTTLTADDVDAALNLKNVEPIYGFASGGPLRFKR
AVGHRDLFYIDDKDVDLKDVIEASLPKAPLDTAVTCHWLAIEGVQPAIPENAPVEVISAPSDVKKHEQKDDNLPVDIKLPVKHVL
SRELQLYFDKVAELTLSESDSVLFKEALVSLATDSGLHPLVPYFTCFIADEVSRGLNNFPLLFALMRVVSSLLQNPHIQIEPYLHQL
MPSVVTCLVAKRLGTRLADNHWELRDFTAHLVASICKRFGHVYSNLQSRLTKTLLNAFLDPKKALTQHYGAIQGLGALGPNVVR
LLLLPNLETYMQLLEPEMLLEKQKNELKRHEAWRVYGALLRAAGQCIYDRLKIFPTFPSPPLHAVWKTNSKVLTSSTYKRKASP
DQLEQQPPLKKAATDGEVGVDLMNFSPVHKQEEAGTQASSADSIIGTSSSSAQMKNETTLDGELRGKRGDTQALKTSAALTQV
WKDELNSGRTLVSLFELFGEGILSFIKAPEMYMFL

>GmTAF7

MEEQFILRVPPNVAERIERLLNENNASSSEDKSLDLSFREDGRSGTFMIGNEQFPASLLDLPCVVESYKTYDDNSLIKTADIGQMIM
VRESGDAAPDVIEYRHGLTPPMRDARKRRFRREPDLNPELVSRVEKDLLKIMAGGTADNLDVETAEQEEGDENARGANKKSAP
KPAPKHDIPENLTNAGEADRSDSEESDDSV

>GmTAF8

MSNGGGKTGRQLEQPGTWRRRKVGGGDDYARAIAKIAVAQVCEGEGFQAFQQSALEALSDVVVRYILNVGKSAHCHANLSGR
TECNAFDVIQGLEDMGSVQGFAGAADVDHCLESSGVIREIVHFVNDAEPVMFAHPIPRFPVVKERVPNPSFLQKGEEPPGEHIPA
WLPAFPDPQTYSQSPAVNGRGTEPRAVKFDQERESGKGEWPALNLQQQMVSNMFEKSASIDPADAKAKRVAAEGNPFLAAPLKI
EDKEVASVPPPAKLFNDEALDNPVVENLVENEPISALETFAPAIEAMKSTICDSKEDQTKFCANEKPTVRFKIGIKNKLLGKSIGLIP
QKEEHEKTLPWFAMEDEKDDRKRRAEKILRESLENPDQLVQLICILDQENVRIASSGIFKFSSKFDLI

>GmTAF9-like

MGDKEEESAMPRDAKIVKSLLKSMGVEDYEPRVIHKFLELWYRYVVDVLTDAQVYSEHAGKPAIDCDDVKLAIQSKVNFSFSQP
PPREVLLELAQNRNKIPLPKTIAGPGIPLPPDQDTLISPNYQFAIPNKRPAQPLEETEDEEATIPNPSQEQKVDTQPNPHQRVSFPLPK
RQKD

>GmTAF10

MNQNPQSSDGRNDDDSALSDFLASLMDYTPTIPDELVEHYLAKSGFQCPDVRLTRLVAVATQKFVAEVAGDALQHCKARQATIPK
DKRDKQQKDKRLVLTMEDLSKALREYGVNLRHQEYFADSPSTGMDPATREE

>GmTAF11

MKQSKDPFEAAFEESPPESPTATETEAETQNPTPPPPPPPPPPSSFSGVAVVALNPQQPQNQKVKNKDNEEEEEEEDNMDVELAKLP
STGDPHKMAKMQAILSQFTEEQMSRYESFRRAGFQKANMKRLLASITGTQKISVPMTIVVSGIAKMFVGEVVETARIVMKERKE
SGPIRPCHLREAYRRLKLEGKVFKRSSSRLFR

>GmTAF12

MDPQAPATGTTVRSAAEPSQPQPPKPSPPLPLPPPSTSSTPPHPPPIPNPNPSPGPGPGPGPIQAPSPKPPTPTPTPPQPRPPQPFNRAL
PPPSQPQFPHFSSPSPPSSAPSAAPPPRGGMAIGVPAHHQSPSPPFSSSFGQHFGGLGRTAVNVAESTSNSSTSQARTPVQGMGMLG
SQMRPSGIGSHQQRSVQSSLRPPTSAPNNQPAGSQSFQGHGLMRPSSVGSTATPSPSSSQSMQSLNQPWLSSGSQGKPPLPSAAYR
QQLNPQSMQQRSHIPPMQQSTPTSSQQQQQQPLLSNQSQEHFGQQVPPSRAPLHMPHQPQVTRLQGPGNQKPSSLVAAQSSAAQ
PGTQSRLTNSDTDESSNSILSKRSIHELVNQVDPLEKLEPEVADILVDIAENFLESITRSGCSLAKHRKSTTLESKDILLHLEKNWNM
TLPGFGGDEIKSYRRPITSDIHKERLAVIKKSMASTEAAHGKGSAGQASGSAKGNQGKTPLNIIGSPNLKNS

>GmTAF12b

MAETASPSSKPSPMDPQIQNPAPSNSNSNPPIPSPSPSHNMASPSLPPLPQDQQQQLQQQLSPPQQQQQQQQQQQQPLVSSNSMNN
MNPISNFQLQQSMQRSPSLSRLNQIQPQQTTQQQQQFGVMRQQAGLYGGQMSFAAAGGAGAAQQQQLGGSNLSRSALMGQSG
HFPMLSGAGTQFNLLSSPRQKGGLVQSSQFSSGNSAGQSLQGMQAMGMMGSPNLTSQLRANGAMAYAQQLRMSQGQIRQQLS
QQGSLNTAQVQGLPRSSSLAFMSSQLSGLSQNGQPAMINSLTQQQWLKQMPAMSGPAAPLRLQHQRQQQLGSSTQLQQNSMTL
NQQQLSQLMQQQKSMGQPQLLQQQQQQQQQPQPQQLQQQLIQQQPQQQSHLQVSVHQQQQQQQQSPRMPGPAGQKSLSLTG
SQPDVTASGATTPGGSSSQGTEATNQVLGKRKIQDLVAQVDPQGRLDPEVIDLLLELADDFIDSATTHGCILAKHRKSSTLESKDL
LLHLEKNWDLTIPGYSSEEKKNQSKPQLNDLHKRRLDMVRTLMESSSSESSINSSKEMSRQGISNPPPVGTHHLVRPLSPEQLVSH
AAGSQMLQQMTRF

>GmTAF13

MSNSSAGTSSKPRTASSQPSETSSKRKRGVFQKELQHMMYGFGDDPNPLPESVALMEDIVVEYVTELVHKAQDIGSQRGKLSVE
DFLYLIRKDSPKLNRCTELLSMNEELKQARKVFESDEEKLRKVFEVDDSVEG

>GmTAF14b

MSNSNPLPLKRQGEQSSDDGASAIKPSRLKIAIPSEDSDKKNANRRLKDVEICVPIVYGTIAFYLGRKASESQSHKWTVYVRGAS
NEDLGVVVKRVVFQLHPSFNNPTRVVESPPFELSECGWGEFEIAITLYFHSDVCEKQLDLYHHLKLYPEDESGPQSTKKPVVVES
YNEIVFPEPSEVFLARVQNHPAVNVPRLPAGLNLPSPVPSDTVNDKERGDTKDHLLTQWFLNFSEADELLKLAAARQQVQAHIVK
LRRQLSLVEGLPQLSKPPSGYECT

>GmTAF15

MASNPGNHAPSNGSVYVCNLPYGTDDIMLAEYFGTIGLIKKDKRTGRPKIWLYRDKETNEPKGDATVTYEDPHAAVAAVEWFN
NKDFHGNTIGVFIAESKNKDEQAYNAAVEPVVADDVGGLEETTKDVNGGSGRGRGQNDSSGKAWQQDGDWLCLNTSCSNVNF
AFRGACNRCGTARPAGASGISGAGGRGKGRAAQESGGIGRPAGGGLFGPNDWPCPMCGNINWAKRTKCNICNTNKPGHNEGGV
RGGRGGGYKELDEEEIEETRRRRREAEDDGELYDEFGNLKKKFRAKTQQAEAARGLPGSGRAGWEVEELGIDRDARESRDRAR
ERNDGESRNRERSEKERPSSWNRERERGRDRDWDYVDRDRDYGRDRDRDRSRHRY

>GmTAF15b

MSGTYDQDGGYGGAASAGYGHGGRGGGGFGGRGGDRGGRSGGRGGGSGRDGDWRCPNPSCGNLNFARRAECNKCGAPSPS
GSNDRGGGGGYNRGGHGNSRGGRSGNFDGGRGNGYNGGSRGNNNSSRSGGGNRGGSFSGSQGFDDGGYGQVPPPAAQSYGG
AGGNYPPAYGSYGGNSNYETDVVPPPASYTGGPASGPPSYGSNAVTSGNNVADARYGGRSGPPVGNDSGYGAGSQGGFGGAPA
EPPAKVKQCDENCGDSCDNSRIYISNLPPDVTIEELRELFGGIGQVGRIKQKRGYKDQWPWNIKLYTDEKGNNKGDGCLVYEDP
SAAHSAGSFYNNYDLRGYKIGVAMAEKSAPKAPPAYNHGGNRSGYGGDRRRDNYRDGGSSGPDRRDNYGGNRSRPY

VI. Protein sequences from *Medicago truncatula*

>MtTBP

MADQGLEGSQPVDLSKHPSGIVPTLQNIVSTVNLDCKLELKSIALQARNAEYNPKRFAAVIMRIREPKTTALIFASGKMVCTGAKS
EVQSKLAARKYARIIQKLGFPAKFKDFKIQNIVGSCDVKFPIRLEGLAYSHGAFSSYEPELFPGLIYRMKQPKIVLLIFVSGKIVLTG
AKVRDETYTAFENIYPVLTEFRKNQQ

>MtTAF1

MKQETATPESQIASRKRNDEEEYEESGKGNHFLGFMFGNVDNSGDLDVDYLDEDAKEHLSALADKLGPSLTDIDLSGKSPRTPP
GIVEQDCGEKAEDAVDYEDIDEEYDGPETETANEEDYLLPKKDFFAAEASLEALERKTSVFDDENYDEESDKEQDFVNNEAKVD
NISLSVEQEESFVDASKEGSALEHDLQVSLQTEELDADVQTPEEVPEFLKRSMATPLPVLYVDDGKAVLRFSEIFGIQEPPRKGEK
KERRHSTPRDRYKSFDLSDDIVEEDEEEFLKGFSESLTLNKQVCVVRTDVSENNVDLEFPKFGFLHGDASLTVKDDRQPKDSCLS
GEPMKGDFADDLAWKDHTLMLANFYPLDQRDWEDEILWGNSPAASDNDNNVESCEISGSELRTSDGGEIEIETRNNLQSVPLKIL
EEKDHNVFTCCSPVSLDPLDSRDSNGVKTNSISESLFHPQLLRLEVDSSHIEDGRGVDVSEKHNQIGQAKRLTKVMSQNRDLMD
DSWVDKIMWEELDRPKMKPKLIFDLQDNQMHFEVLDSNDGTHLHLHAGAMILTRSLKSISVESSELPGHGGQYGWRYVANDK
HYSNRKTSQQLKSNSKKRSAHGVKIFHSQSALKLQTMKLKLSNKDIANFHRPRALWYPHDNEVAVKEQGKLPTHGPMKIIMKSL
GGKGCKLHVGAEETLSSVKAKASKKLDFKATETVKIFYLGRELDDQISLIAQNVQPNSLLHLVRTKIHLWPRAQKVPGENKSLRP
PGAFKKKSDLSVKDGHVFLMEYCEERPLLLSNVGMGARLCTYYQKSSPDDHSGAALLRNTDSSLGHIISLDPADKSPFLGDLKP
GSSQSSLETNMYRAPVFAHKVPPTDYLLVRSPKGKLSLRRIDKVNVVGQQEPLMEVFSPGSKNLQTFMMNRLLVHMCREFQAA
EKQHLSPYIRIDDFLSQFPFLSEASFRKRIKEYANLQRGANGQSIFVKKRNFRMWSEDELRKMVTPELVCAFESMQAGLYRLKHL
GITETHPNNISSAMSRLPDEAIALAAASHMERELQITPWNLSSNFVACTSQGKENIERMEITGVGDPSGRGLGFSYARAPPKAPVS
NAMVKKKAAANRGGSTVTGTDADLRRLSMEAAREVLLKFNVPEEDIANQTRWHRIATIRKLSSEQAASGVKVDPTTIGKYARG
GQRMSFLQLQQQTREKCQEIWDRQVQSLSTLNGDDNESDSEGNSDLDSFAGDLENLLDAEEFEDGEEATNDLKRDKGDGVKAL
KMRRRTTLAQTEEEIQDEAAEAAELCRLLMDDDEAYRKKKKKGKVMVNPRRLVPKLQPKFVFDNTEQVKQITNTLQLNGSNHF
KEDALTDHREEENLSAKKSKSVKVNKVKKNDISPISVPNKKIKLNMGEGIKNQVFKEKKPSRETFVCGACGQLGHMRTNKNCP
KYGEDPEAQLESTDMEKPTGKSSFGDPSSQSQHQLPSKKSISKIVTKLAPVENSTKIPLKFKCSSTEKSSDRPAVETLQSSDKPVTS
DKPVISDSETAKSAKISKIIIPNKVKSDDTQAESLKHAIVIRPPTDPGRGQVDSHKFPIKIRPPAEIDRERSHKKIVIKRTKDVVDLEL
DSPGGNTGFEHRKTKRIVELANFEKHRKQETMYSTESLVKRNSKEDRRWWEEQEKRRNEARLREDKARRYRKEEMRMQEQER
LNDLKMQEQERLDDLRRYEEDIRKEREEEERQKAKKKKKKRKPELRDEYLDDSRERRHGKRMLERERSGKRRSVVELGKFGE
DFMPPTKRRRGGGGEVGLANILESIVDAIVKDRYDLSNLFLKPVPKKLAPDYLDIIERPMDLSKIRERVRNMEYKSREDFRHDVW
QITFNAHKYNDGRNPGIPPVADMLLEYCDYLLNENDDSLTAAEAGIETKDF

>MtTAF2

MAKPRKPKPEDPKPENSGAVVQHQKLCLSIDMDKRLVYGYTELKIAVPEIGIVGLHAENLGIESVWVDGEPTEFEYYPHQYSQN
DDEKRWSSVTSPSSAADAAVSVYLSSLEKELVPNLLINCFKPSKTETEQQQKEHEKEQPVTENGFHSAAAAEPPKQNVRLVRINY
SIEKAETGVHFRDSVLHTDNQIRRARCWFPCIDDNIQQCCYDLEFTVAHNLVAVSTGSLLYQVLSKDNPPRKTYVYKLDVPVSAR
WISLAVAPFEVLPDHQFGLISHMCLPPNLAKMRHTVEFFHSAFSCYKDYLSVDFPFDSYTQVFVEPEMVVSSLSLGASMSIFSSQV
LYDEKVIDQTIDTRVKLAYALARQWFGVYITPESTNDEWLLEGLAGCLADFFIKKHLGNNEARYQRYKANCAVCKADDSGATIL
SCPASCKDLYGTQCIGLYGRIRAWKSVAVVQMLEKQMGPESFRRILQAIVTRAQDKTRCVKTLSTKEFRHFANKVGNLERPFLKD
FFPRWVSSCGCPDLRMGFSYNKRKNIVELAVLRGCTALQTSSTAVLDINPDSENRDGDSGWPGMMSIRVYELDGMYDHPILPMA
GEAWQLLEIQCHSRLAARRLPKSKKGVKHDGSDDNGDVPPVDTRSSTESPLLWIRADPDMEYLAEVHFNQPVQMWINQLEKDK
DVIAQAQAIAALEASPQLSFSVVNALNGFLTDSKAFWRVRIEAAFALANLASKETDFSGLLHLVKFYKSQRFDPDIGLPKPNDFH
DFAEYFVLEAIPHAVATIRAADKKSPREAIEFVLQLLKYNDNTGNPYSDVFWLAALVQSIGEFEFGQQSILLLSSLLKRIDRLLQFD
SLMPSYNGVLTVSCIRTLAQIALKLAGFIPLDRVYELVKPFRDQKAIWQVRIEASRALLDLEFHCKGIDSALLLFTKYVEEEPSLRG
KLKLATHVMKLCQMRDGLNSNDEITSQTLVSLLSLLEGRMAFNNVFLRHYLFCILQILAKRPPTLHGIPRESRTLHMSLTGASNY
QRNLFVIDSDSKPLELPSSTQNLTQDLIITEGLRDALNEAPKDQTVEAPKEVHVEVLKDVPLETSKEDLTGLPPEAPIEAPNEISKET
DTVSNSHERKRLFKIKVKQSSATSRADTDNQLVERSLGGRNETDHGASSSVSVDAPQRNFAETVSISNHNIEEVNSCYDPGSRMT
ASIGSAKILSDGDELVKELQCTADSSVVYSQVQPEDPSPSSIIQDNNIDVDARRFASLQTLSVTRFDQAGESCGKEVPARGKHKHK
DKDKKRKRESHKGQQNDPEYLERKRLKKEKKRKEKELAKLLSNEAKRSSIDLSCKKEEPEVNDAKQLKSVEPSCYNSVSEIGRV
DPKPVPPEGTSGAPKIRIKIKNRMLSKS

>MtTAF4b

MDPSIVKLLEDDEDETMHSGADVEAFQAALNRDIGGDASNSHLSDSDAGSNNSFSQSLPTWPTSSHDNQTDSQNQEPKIEQQQE
QPSSETEQGPIVEPIQNVASQDASNLNLSHSQSQDESLQRQTLPVSHQQSQTNEVIKSEKEPVFNNEAIKTNNPNEQSQYAKLQQM
SNQQASVNEQPISQVNRSKQVPFGLLLPILIPQLPKDRAMQLQTLFNKLKRDEIPKEHFVRLMKGIVGDQMLRIALTKVQQQTKT
NTGSSGQQPPVRMPTVTSSGTKFNDPHALAQLHQRSMNAAADHSHNASSAIQVKSEPTYSTMDISAKKPQEHDVRVVQSNQLP
TSSSIAVSQETERSAVHMQGLNKQQQQHIHFPSTYGGSGGNYSHFSGTTTSSSSSFRPQPHPHDSHIRQIPHPSIGLNHLGAERQSSF
NDPKRMPGGSVSTVVNNTSSHQTSNSWQTSAEQNSGLFSSTSYVKKEPNDLSIEQQHRHHLSKLHGLPSVNSGQNEQGSGINQG
TVKDEFSRGSLASTSMAHTTSASLLAPNTASHFASQPDPTVSVSSQIPASTSGVMSKTPLKKPPLGQKKPLEALGSSPPPPSKKQK
VSSLEPSIDQLNDVTAVSGVDLREEEEQLFSGPKDDSRVSEASRRVVQEEEESLILLKAPLQRKLIEIMTECGLKGMGNDVERCLS
LCVEERMRGVISNIIRMSKQRVDIEKTRHRTVVTSDVRQQIMTMNRKAREEWEKKQAETEKLRKLNDVEGSSGVDGDKEKDEG
RNKATKVNREVDDKMRTNAANVAARAAVGGDDMLSKWQLMAEQARQKREGGTDTASGSQQTKDISRKSSPSSGRSTKDNQE
RERKGPTSLGNSAARKFGKNHSHGSQTRIARSISVKDVIAVLEREPQMSKSSLLYRMHERIHSDTSTE

>MtTAF5

MDEDEILGYVTAYLKKKGFKQTEKVFQEEFQQNKTSSSSNSILEPDIANHLHAFSQLENGPARYHNGYSRLRTWTYSSLDLYKHE
LLRVLYPVFIHCFMDLVAKGHIQEARNFFTTFREDHELMHLRDIQKLEGVLSPTHLKEMEFAHSLRQSKFNIKICEYSYELLLQHL
HSTQSTTILGIINEHINFQVTSGQPSLISDDPEAVTLTGSSQEAANQTNQKEIHWGLLEDSLEERLEKPGALLSDSEKGDGEAKEGE
NDENKKRSIEVGKQGASSKKMKKDRGGTATGKSAKPEVTTVSAAPRVKAELPLPIIPTEVEHSILEDLRNRVQLSSVALPSVSFYT
FINTHNGLSCSSISHDGSLVAGGFSDSSLKVWDMAKLGQQPSSSLSQGENDTSQNEQMLGKSGGKRQYTLFQGHSGPVYAASFC
PVGDFILSSSADSTIRLWSTKLNANLVCYKGHNYPVWDVQFSPMGHYFASSSHDRTARVWSMDRIQPLRIMAGHLSDVDCVQW
HANCNYIATGSSDKTVRLWDVQSGECVRVFVGHRGMILSLSMSPDGRYMASGDEDGTIMMWDLSSGRCVTPLVGHTSCVWSL
AFSSEGSILASGSADCTVKLWDVNTSTKVSRTEEKNGNANRLRSLKTLPTKSTPVNTLRFSRRNLLFAAGALAKNA

>MtTAF6

MSIVPKETIEVIAQSIGINNLSPDVALSVAPDVEYRMRQIMQEAIKCMRHSKRTTLTADDVDAALNLKNVEPIYGFASGGPLRFKR
AVGHRDLFYIDDKDLDLKDVIEAALPKAPLDTALTCHWLAIEGVQPAIPENAPVDVISAPSDIKKHEQKDDNLPVDIKLPVKHVLS
RELQLYFDKVTELALNEPDSVLFKEALVSLATDSGLHPLVPYFTCFVADEVSRGLSNFPLLFALMRVVNSLLQNPHIHIEPYLHQL
MPSIVTCLVAKRLGSRLTDNHWELRDFTANLVASICKRFGHVYSNLQSRLSKTLLNAFSDPKKAMTQHYGAIQGLGALGPNVVR
LLLLPNLEAYIRLLEPEMLLETQKNEMKRHEAWRVYGALLRAAGQCVYVSLKLFPAFPSPLPHTVWKTSASVLTSPPNKRKASP
MELEQQPPLKKVDTDGEVSVVQENSSASHKEEETVTQASSAELKIGAASSSGETKNKITTDGVVRSSGGDTQDLKISSVLAHIWK
DELNSGRVLTSLVELFGENILSFIQNREMCMFL

>MtTAF7

MEEQFILRVPPNVAERIERLLNENNASSSEDKSLDLQFSDDGRSGTFVIGDEHFPASLLDLPAVVESYKTYDDNSLVKTADIGQMI
MVRESGDAAPDVIEYRHGLTPPMRDARKRRFRREPDLNPELVSRVEKDLLKIIAGGTAENIDVEVAEQEGGENARGANKKPAATS
ASKNDVPETHTNAGDADRSDSDDSDDSV

>MtTAF8

MSNGNGKTGKQIEQQPNTTCRRKRVGGGDEFAQSIAKVAVAQVCESKGFQGFQQSALEAMSDVTARYIMNIGKSANCYANLAG
RNECNVFDVIQGLEDMGSMQGFTGASDIDHWLEDSGVVREIVQFVNEVEPVMFAHPIPPFPVVKERVLPPSFLQRGKEPPDEHIP
AWLPAFPDPETYLQSTTVNGRGTEPRTTTFEHERENGKGDRPLLNSQQQMVSTMFENSTMVDPAVAKAKIVGAESNPFLAAPLKI
EDKEVSSVAPPAKFFNNVSSDTPVGENLIQNEPGSVLETFAPAIEAINSASCDSKEDQTKFPVKEKPTVRFKVGTKNKFLGRSIGLIP
QNEEHKKTLPWFAMEDEKDDRKRRAEKILRESLENPDQLVQL

>MtTAF9

MADNEEDSNMPRDAKIMQSLLKSMGVEEYEPRVINKFLELWYRYVVDVLTDAQVYSEHAGKPAIDVDDVKLAIQSQVNFSFSQ
PPPREVLLELAQNRNKIPLPKSIAGPGFPLPPDQDTLIAPNYQFAIPNKRSVEPMEETEDEEVPNADPNPSQEEKTDAEQNPHQRVS
FPAKKETMSLTLLLVGDGCDKRRIFGYIDHPCW

>MtTAF10

MNQNQPQSSSSEGRADDDSALSDFLASLVDYTPTIPDELVEHYLAKSGFQCPDVRLTRLVAVATQKFVAEVAGDALQHCKARQAT
IPKDKRDRQQKDRRLVLTMEDLSKALREYGVNIRHQEYFADSPSTGMDPATRDE

>MtTAF11

MKQSKDPFEAAFEESPPESPIETEPDPDASTENPNSTNSSLPQSTLTHEEEHNHIKTPNSNNTITKHKDEEDDEEEDNMDVELAKFP
TAGDPHKMAKMQAILSQFTEEQMSRYESFRRAGFQKANMKRLLTSITGTQKISIPITIAVSGIAKVFVGEVVETARTIMKERKETG
PIRPCHLREAHRRLKLEGKIFKRTTSRLFR

>MtTAF12

MESQPPATGTTARTTTEPSQSQPQHQPPPSLPPPTSSTPPISAPSHPSPIPNPNPNLIQTPNPKPPTPLPPQSRPPPSSFSRNPPPTQSHYS
HFSSIPPSANPSQASSFPSNPASSISSASAPRGGMAIGVPAHHQSPSPPFSSSFSQHFGGMGRSDSTTNSNTSQVRAPMQGMGTLGS
FGSNSQMRPGGMPPHQQRPVQSSLRPPPPSAQNNQPAGSQSFQGHGLMRPSSAGPPSAPSSSASQGMQSINQPWLSSGPPGKPPL
PSPAYRQQINPQSLQQRTHISQQQQSMPTASQQQQPLPSNQTQEHFGQQVPSSRAPHVPHQAQVTRLQGPGNQKPSSLVAGQSGA
VQPGSQSRLPNTLPNADIEESGKSVLSKRSIHELVHQVDPLEKLDPEVADILGDIAENFLESIIRSGCSLAKHRKSTTLEAKDVLLH
LEKNWNITLPGFGGDEIKNYRKPLSTDIHKERLAAIKKSMIATEAAHPKGSAGQASGSAKGSQAKIPFNVLGSPNLKNP

>MtTAF12b

MATPPSTMAQNMISSQQSTIDTQTQNPISSNPTIPSPSPIPQQLPAIINLNPSPNPNVPSFQIQPLQQQVAVQQAGLYGGLMNFGGSA
AVPDQQQQLSGSVGVGVGGNASNLSGAGATQFNLLTSPGQKVGLVQTSQLSSANSAGQSLQGMQQAIGTLGSSNLASQLMTTN
RSLYGQKQQLAQQASLNNQQAQQQQLAGGVGVCIGSGAFNFGGSAVVTAQQQQLAGGVGLGIGGNTSSLSQSALAGPSGPFPK
LSGASATQSNQLTSPGLQVGLVQSSQFSSGNSAGQSLQGTQQAIGTMGSSNLASQLMTTNGALYAQQLQLSQQAPFNNQQLVGL
PQSGQPAMIQNQLLKQIPAVSGSASLLPLQQPQSQQQLASSAQLQQSSLTLNQQQLPQLMQQPKPMGQPQLQQQLQHQQLLQQQ
LQLQQQYQRLQQQLASSAQLQQNSLTLNQQQLPLLMQQPKSMGQPLLQQQQQQLLQQQLQLQQQYQRLQQQLASSAQLQQN
SSTLNQLPQLMQQQKSMGQPLLQQQLQQQQLLLQQQQLVLQQQQQQQQQQRTQLLQQQMQALRIPGPAGHKSLSLTGSQPEAT
AFGMTTPGGSLSQGTRTNAMFGQTEIMSVVNELKETRKEVKAIIKILLRFFKVISIVMALFIFVTLFVVLIKPSSSSNPMAENVIGSP
DSQIQNPSSSNPSIPSPSPISQQQQSPSIHMSNSSPSLSQDQQQLHTINTINPNSNFQLQQTLQRSPSMSRLNQIQPQQQQQVVARQQ
AALYGGQMNFGGSAAVSAQQQQLSGGVAAMGGSASNLSRSALIGQSGHFPMLSGAGAAQFNLLTSPRQKGGMVQSSQFSSANS
AGQSLQGMQQAIGMMGSPNLASQMRTNGGLYTQQQQIRLTPAQMRQQLSQQALNSQQVQGIPRSSSLAFMNSQLSGLSQNGQP
GMMHNSLTQSQWLKQMPAMSGPASPLRLQQHQRQQQQLASSGQLQQNSMTLNQQQLSQFMQQQKSMGQPQLHQQQPSPQQ
QQQQQLLQPQQQSQLQASVHQQQHLHSPRVAGPTGQKSISLTGSQPDATASGATTPGGSSSQGTEAATNQVLGKRKIQDLVAQV
DPQGKLDPEVIDLLLEFADDFIDSVTTHGCILAKHRKSSTLESKDLLLHLEKNWDLTIPGYSSEEKKYQSRPLSNELHKRRLDAVR
MLMESSSVPESIVNNSKDISRQGHPNPAGSHHLMRPLSSDQLVSHSTSSQMLQQMTRF

>MtTAF13

MSNSAAGTSSKQRAASSQPPDTSSKRRRGVFQRELQHMMYGFGDDPNPLPESVALMDDIVVEYITELVHKAQDIGSQRGKLSVE
DFLYLIRKDMPKLNRCTELLSMNEELKQARKLFEHDEENLRKVFEVDEPAEG

>MtTAF14b

MSQSQTLPLKRHQDENPRDDDSSDIKPSRLKISLPSEDSDKKILIKRVKDVEICVPIVYGTIAFFLGRKASESQSHKWTVYVRGAS
NEDLSAVVKRVVFQLHPSFNNPTRVVESPPFEISECGWGEFEIAITLFFHSDACEKQLDLYHHLKLYPEDESGPQNTKRPVVIESYN
EVVFPEPSEAFLARVQNHPAVVVPRLPDGLNLPSPVPVEPMNDKERGDTKDHPLNHWFLNFSEADELLKLAAARQQVQAHIVKL
RRQLTLMEGLPQSKQPSG

>MtTAF15

MASIPGKFAPSNGSIYVCNLPYGTDDNMLAEYFGTIGVIKKDKRTGRPKIWLYHDKETNEPKGDATVTYEDPHAAVAAVEWFNN
KDFHGNTIGVYIAESKNKDDQTYTAIAEPVVAGTDVGPEETESDVDGVNGRGRGQIDASSKPWQQEGDWMCPNTSCSNVNFAF

RGACNRCGSARPAGASGVAGGGGRGRGRSGPDAGGIGRPAGATAGLFGPNDWPCPMCGNINWAKRLKCNICNTNKPGHNEGG
VRGGRAGGYKELDEEEIEETKRRRRQAEDDGELYDEFGNLKKKFRAKTQQSETARVLPGSGRAGWEVEDLGIDRDRDATEKSR
DRGRESRDRYDGEQRNREHNEERQSSRNRERDRGRDRDRDYGRERDRSRHRY

>MtTAF15b

MSGGYGHDAGGGSAPPSYGASGGYGGGGGGGYAGGYAGNESGGGYAAKGSGNGGGRGYGGNDGGGYGGRSGYGGNDGGG
YGGRGGGGRGGGGGGGGYGGRGGGGGFQGGDRGGRGGGGGRGGSGRDGDWRCPNESCGNNNFARRSECNKCGTPCPTSGN
DRGGGGGGGGYNRGGSGGGYDSNRGGRSENYGGRTSDYNGGRGNNNDGRSGGSNRGGSYGGNQGREDGGYGQAPPVAAAQ
SYGGAGGNYPPTYGGNANYGTDAVPPPTSYTGGPNSYPPSYGGNTGGYGGGDARSGGRAVPQAGYDSGNRGGFGGAPAEPAP
AAPVKQCDENCGDTCDNSRIYISNLPPDVTVDELQQLFGGIGQVGRIKQKRGYKDQWPYNIKIYTDENGKNKGDACLAYEDPSA
AHSAGGFYNDYDLRGYKIGVAMAEKSAPRAPPANNHGGNRGGYGGDRRRDNSGPDRRDHYGGNRSRPY

VII. Protein sequences from *Zea mays*

>ZmTBP1

MAEPGLEDSQPVDLSKHPSGIVPTLQNIVSTVNLDCKLDLKAIALQARNAEYNPKRFAAVIMRIREPKTTALIFASGKMVCTGAKS
EQQSKLAARKYARIIQKLGFPAKFKDFKIQNIVGSCDVKFPIRLEGLAYSHGAFSSYEPELFPGLIYRMKQPKIVLLIFVSGKIVLTG
AKVREETYTAFENIYPVLAEFRKVQQ

>ZmTBP2

MAEPGLEGSQPVDLSKHPSGIVPTLQNIVSTVNLDCKLDLKAIALQARNAEYNPKRFAAVIMRIREPKTTALIFASGKMVCTGAKS
EQQSKLAARKYARIIQKLGFPAKFKDFKIQNIVGSCDVKFPIRLEGLAYSHGAFSSYEPELFPGLIYRMKQPKIVLLIFVSGKIVLTG
AKVREETYTAFENIYPVLSEFRKIQQ

>ZmTAF1

MDDSELHEEENPTNSAVDDDDDEDYEEPGGGNHLLGFMFGNVDDSGDLDADYLDEDAKEHLFALADKLGPSLKDIDLMKSSPA
PTDPSEQDYDEKAEDAVDYEDIDEEYDGPEVEATTEEDNVLSKKDYFSSSTVYASVNSTVSVFDDENYDEEEEPPNDNEPPGDSA
AQNLSSVSIEQADTTTSSDNLAMEKIGLLSHPEENMDFEYEDLENEKGTGEGQLEPESATSLPVLCIEDGNAILRFSEIFGIQEPVR
KVKTDHHKRPVDKELQIANVADNVEEDEELILRSTMQNFSTLKHIHVNEDFVESDSDESISDVTLRLKDSCLSEQPMKDVHMDIR
IVQRSPTCPDFYPLEHYDWESDIIWNNSPATDQQAYAKICESEESEDTHGEDQGKDYGQASRCWDVQSKTNGSPVIKETFCCTEM
PAPANYCPPGKSYPPLTNEDNLDHIMPNNLDDAVKIDTMVRLNNLSVLNRELLEGSWLDNIIWDPNEVTPKPKLIFDLKDDHMLF
EILDEKNVDHLQSHARAMIVSQSTKTSTPTVDNFGDQPKPLSGRFNISNDKFYSNRKTPQQAKSHTKKRALMGIKVVHSAPAHK
LQTMKPVLSNKEIANFHRPRAKWYPHENKIASQLQGPACSHGRMAVLLMSLGGKGVKILVNAEDTPVSVKLKASKKFELKPSE
KIKLFCSGKELQDDISLAMQNVRPNSIVHAVRTEVYLWPKAQKLPGEDKPLRPPGAFRKKTDLSIKDGHVFLMEYCEERPLLLSN
AGMGARLCTYYQKTTPTDQTAASLRNNSDGLGTVLAIDPADKSPFLGEIHSGSHQSCLETNMYRSPVFPHKVAPTDYLLVRSAK
GALSLRRIDKLYVVGQQEPHMEVFSPGTKNMQNYLLNRVLAYVYREFRARERPDAIPQIRADELPIQSPLTEAIVKKRLKHCADL
KKGPKGHFFWTQRPDFRVPSEEELRRLLTPESVCCYESMQAGLYRLKRLGILKLTQPVGLASAMNQLPDEAIELAAASHIERELQI
TSWNLTSNFVACTNQDRENIERLEITGVGDPSGRGLGFSYVRVAPKAPASNSVLKKKSAAAKGTTVTGTDADLRRLSMDAAREL
LLKFGVPEEQIDKLTRWHRIAMVRKLSSEQAASGITIDEIPVSKFARGQRMSFLQLQQQTREKCQEIWDRQVQSLSALDGDDNAS
DTEAHSDLDSFAGDLENLLDAEEFDDEDTSTADLRIDKADGMRGLKMRRCSTQAQINKEIEDDETEASLAKKLLEDDGNDVKR
KKQPVELTNCGTSLVANKMKQSKTGQTIKSSGYAGALTPKENTPREGKEVENSFVEGGLSSKLKPKMALDVNETVLVKKKSVPG
KDGPKEKKQGARGDSLVCGACSQVGHMRTNKLCPKYREDPEMSEMDANSVKPNSMDINHLQAKTPKRLITKVSSEVTETEGPE
GIEKTKSVPVKFKVGAPDKSLERNMSVSVSLVSDKRVMDVTDSRSTGKVNKIVIPNKMKSDDFPPDTPKPSVVFRPPAEEKDVPR
KKITIKQPKGIDQQRHVESRSVQEPTRKTRKIVELSSFEDKSREDDHWVGGEPSQVNSSHGRRLGLEGKISKATMENERSWRDFE
ERREMPQARLFDATIYASREEDHLKAKKKNKKKKKHEFKDDDLLDHRPYRNDRRVPERHRALKRSSPAPVVGYASSAKRRRGG
EVELSNILEKVVDHLRGLSGSLLFLKPVTKKEASDYLDIIRYPMDLGTIRDKVRKMVYRNRDEFRHDVAQIQLNAHIYNDTRYPH
IPPLADELMEVCDHLLYENADLLTEAEDAIE

>ZmTAF2

MAKARKQKGDGSGTGGGATVLHQKLCLSIDMENQLIYGYTEIKALLAENDTFALHADNMTIRSILVDGETVEFDYSPHWKNET
DQPNWSSISCLNTVADAACSTYTSSLNREATPNLIVSSERSIKSMIEQQLDENSEKYEENSGRLEKHGGKTIQTSYDQIVNGCNGS
AVEEGIENSAVEEGKKKEKEKEKEEKNGNEKSKENGNETENEKVKNIKLVHIDYILEKAETGIHFVGNVMHSSNQIRRAHCWFPC
VDTATQRCPFDLEFTVSTDFIAVSNGDLLYQVLSKEDPSKKTYVYKLNTPVSAQWISLVVGPLEILLDRNDINVSHICLSPALSKLQ
NTIAFFHDAYSCYEDYLAAPFPLGLYKQIFLPSEMTVLPASLGASMCIFSSDILHDEKVIDLIIGTRIKLAYALAKQWFGIYTSAEEP
NDEWLLDGLAGFLTDLFIKRYLGNNEARYRRFKANHTVCESDVSGATALSSTAASSDLYGTQTIGSYGKVRSLKAVAVLQMLEK
QMGPDSFRKILQVIVAPNRASRTLSTKEFRHLANKVGNLERPFLKEFFPRWIESCGCPVMRLGISYSKKRNMVELAVSRGCTGKA
TPDPDSHTNGDTREGDAPGWPGMMSVRVHETDGAYDHPVLPMAGEALQVVEIQCHSKLAAKRVWKSKKNTKLDGSDDNIDTS
NQENRTSMDSPLLWIRVDPEMEYLAEIHFHQPVQMWINQLEKDKDVISQSQAISALEKLPQLSFAVINALNNFLNDTKAFWRVRI
EAAYALAVTSSEATDLAGLLHLVKFYKSRRFDSDIGMPRPNDFHDVPEYFVLEAIPHAVALVRSSDKNSPKGAIEFILQLLKYNDN
NGNVYSDVYWLSAMVLAIGELEFGQQGTGFLPSLLKRIDRLLQFDNFMPGYNGVLTVSCIRTLARIARRLSSSVSLDPIRELIAPF
RDMDKPWKVRIEASMVLIDLELHHKGLDAALLLFLKYVDEEKSLRGATKLAVHILRICQANIVPSANDQINLTTLIGLLHLLAGT
KAYNNVFLRHHVFCILQVAAGRSPTLFGVPKVVTPPLVVKDICSDQHTKADSSIPQPSKPQEPSTSTPSVREVLPTSGPTKDADNIS
NCSERRNVVKIRVKLTASSSKASDADHRGHFHGGRNENEPGPCSSMSVDAPMVGTANEPLNVSNHNIEEQNSCHDRESRMSASV
STVKLLDRHDISRELQCTADSRLDALPKNQFSPAINLPEALDKPGSQLEGVSTSYEGNQAPESVNGVETKERKKKDKKDKKRKR
DEKRLKKDDPEYLEKKRLKKEKKMEKELARKQLEGEGRATPEQPKTVKPSGSLEVLPARPPAPVLSAEPAAVRSSEPQVSSKETT
VDTARTAAKPRIKIKVKPLVRKPEGN

>ZmTAF4b

MDPIMKLLEDDEDESLHSGADVEAFTAALNREVEASASASASTSTSVPAGSSSQPTDHGAGLLPQEHKSLLNHDPGQWQDPVKN
EIVNQESQQQEQTHAFRNDHPSRPEMVSQGSNNCPLPTNTPKECDLLKAKQEPGSTSQQGIVAQHQPMQQMKSEQTPIVSQQQS
MQQMKNQQTPVTNQTNSATTTAKAPVVTFHMLIPILSRYLDKARDIEVQSIFAKLRKNEVSKEYFLKTVRNIVGDKLLKQAASQ
YQMQAQRSPQTNPSNYSLSGQVSGQQTAPSGSVTGDEQKGYPGAHTIPMRQAIASTRPPQFRPSSSGQMRSNTGYPPSQTNLHK
ANEMGNMSDGKGVHMLQTRPPNNSIPVQTMQHHVQRPQTSSPVFGANSIHARPFPRPLGGPAAPFRPQMADSNPKAQLIQGAV
TTVSGSVPTRSIVSGNAPGNQSTRQQSANKEQKTISFTRTAHMNNETVSQNSEFSQNSFAVMHAKQVNQALGSSKVSAGMESQS
QQLSAPKPLAATSLSQTQSHGIQEEPKIQIQSSVQAPPAAASKTPQRKASSGQKKPLEVLGSSPPPSSKKQKVSGGFHEQSIDQLND
VTAVSGVNLRVDDFTCYVYWLCACHLPCSTYYLMDFEGITVCDFCQEEEEHLFSAPKEEGRVSEVARKVVQLEEEKLILQKGPLT
QKLAGIMSKCNLKVIGTDVERCLSMCVEERLRGFIRSIIRFSKQRVEVEKSRHHFYPLSSDVRSHIMRVNREAREQWEKKQAEDA
ERIRKQNDGDGNANVDLEKDRIETRGLSKHAKTYKEDDDKMRTTAANVAARVAAGGDDMLSKWQLLAERNKQRSEGGDGSS
GSVPGNMLQHRPSLKSGKDLREEHEVEKRGYSTMLGSGGVRRSPLTKVARSISMKDVVAALEREPQMSKSSLLFRLYGRPLTEPS
AK

>ZmTAF5

MEDEEMEKKVQQYLQRKGFRLTELALQEERNRLSTSAISDVALARSDNDPARYYDGYSRLRTWAYSSLDQYKHELLRVLYPVFI
HSFMDLVAEGHMQEARSFFHTFREDHEVMHSRDLQKLEGVLSPSHLEEMELARSFRKNKFKIKLCEYSYELLLQYLQKTQALVV
LGVINEHITFEVSPGQPSLISDDADVVALIGTSKDLAKQINQKEVHWGLLEDSVEERMEKALAESDKIEAESKDADAEDNKKRNA
EGGKQGASLKKAKKDKLVGATGKSVRTETSMVSVAPRVKPELTLPATPVEVEQSILEDLRNRAQLNSVALPSVSFYTFLNTHNGL
NCSSISHDGSLVVGGFSDSSVKVWDMSKMGQPAKISRSQGENGPSQGERISTLDEGKRTYTLFQGHSGPVYSAAFSPFGDFLLSSS

SDSTIRLWSTKLNANLVCYKGHNYPVWDVQFSPVGHYFASASHDRTARIWSMDKIQPLRIMAGHLSDVDCVQWHVNCNYIATG
SSDKTVRLWDVQTGECIRMFIGHRSMVLSLAMSPDGRYMASGDEDGTIMIWDLSTGRCVSPLLGHSSCVWTLAFSCEGALLAS
GSADCTVKLWDVASSTKTLKTEDTKGSSANRLRLLKALPTKSTPVYSLRFSRRNLLFASGALSLSSS

>ZmTAF6

MSIVPKETIEVIAQSVGIPSLGADVAVALAPDVEYRLREIMQESIKCMRHAKRTVLTADDVDSALGLRNVEPVYGFASGDPLRFKR
AVGHKDLFYLDDREVDFKEIIDCPLPKAPLDTSVVAHWLAIEGVQPAIPENPAIDAIVPPTENKRSEHGKDDGLPADVKLPVKHVL
SRELQMYFDKIAELTMSRSDTSLFKEALVSLAKDSGLHPLVPYFSYFIADEVTRSLGDLPVLLALMRVVQSLLRNPHIHIEPYLHQ
LMPSMITCIVAKRLGHRLSDNHWELRDFSANLVALVCQRFGHVYHNLQNRLTKTLIHAFLDPAKSLTQHYGAVQGISALGPSAIR
LLLLPNLVTYMQLLEPELQLEKQKNEMKRKEAWRVYGALLCAAGKCLYDRLKLFPGLLSPSMRPLLQSNKRVLTNNPNKRKSS
TDLSATQPPLKKMATDATANSMASASMGGNMQGAMDGFPNQLGNPGMMQASSSGQTVESIPSAAVRRDQGSDLAQRVSAVLR
QAWKEDQDTGHLLGSLYEVFGEAIFSFVQPPEISLFV

>ZmTAF7

MEEQFILRVPPSVAERIERLMNESAASSSNPDEASLDLSFSEDGRNGTFMIGNESFPASLLDLPAVVESYKTYDDSVLIKTADVGQ
MIMVREENDPAPEGVEYKHGLTPPMRDARRRRFRREPDLNAELVNQVEKHLISIMHGVSVNQNASATGGEEGGDRKKPPVARA
AKQPGVQEPAANGEEADPERSDSDDSDN

>ZmTAF8

MGGWKAGERRSSGDMNGGGGSSGDEFGRAVARAAVAQALQAAGFDCAHRSAVDAVVDTVLRYITHLGRSAAFHANLAGRAH
ANELDVIQALEEVGADTYGFAGAATTGHCLVGSGVVKDLMAFVHSKDEVPFARPLPRFPIQRVEPQPSASFAVTGRETGMKHVP
EWLPAFPDPHTYVTTEVWVEPLATKDRVDKVEQVRQRRKAEKSLLSLQRRLAMAGADGFRPAVAHDNALKGKEIQAAGSKRNP
FLEPALLPGQKDVAEVDMPPEKKKFSVLEAFAPAIQARTIREIDASAGLDQNQRNIVPKERAPVHLKIGFSNKPLAAAQNSRALDL
RDDLSFLKEETKDDKKRRAGMILRASMENPQELPQL

>ZmTAF9

MDAGAARPSAPSTAAVAGASVADEPRDARVVRELLRSMGLREGEYEPRVVHQFLDLAYRYVGDVLGDAQVYADHAGKAQIDA
DDVRLAIQAKVNFSFSQPPPREVLLELARSRNRMPLPKSIAPPGSIPLPPEQDTLLAQNYQLLPPLKPPPQYEETEDETGEPNPSNP
ANSNPSYSQDQSNKEQQQQHTPQHGQRVSFQLNAVAAAAAAAAKRPRMTIDQLNMG

>ZmTAF10

MKRNICFGVQKSNFFWEKCEHIVCLEKSNFLLTDAAPVPRHRLRYARRPNTSARLRSCLRCPSPRRLFGLLPLVTTCAVGAVRQQ
DVRSRMMNNNSGGGAGGPGGGMGTGVGGGGDGRHDDEAALTEFLSSLMDYTPTIPDELVEHYLGRSGFHCPDLRLTRLVAVAT
QKFLSDIASDSLQHCKARVAAPIKDNKSKQPKDRRLVLTMDDLSKALREHGVNLKHAEYFADSPSAGMAPSTREE

>ZmTAF11

MKDPFEAAVEEQDSPPDSPAPPEEEPGAAGLADDAEDYDGGPPPRQPSAPASHAASAAAKAKGRVQREQQEDDDDDEDQMEV
DLDKLPSSTGDPDKLAKMNAILSQFTQQQMNRYESFRRSGFQKSNMKRLLASITGSQKISIPTSIVVSGIAKIFVGELIETARVVMS
ERKDSGPIRPCHIREAYRRLKLEGKIPRRSVPRLFR

>ZmTAF12

MDAPPPSQPDDAAPAPSSTLASAPSPAPTSNPPTSAAAAAPAPDSAIANPNPNLGTVANPAQTLEAPGPSSAAARPPPPRMRTPYTH
LAAPITMSSSSAATATASSASVPAASSSAPPIPRGGVVLGVPAPRSAQTPAGYTGFVPPPPLAHQFGSMHRGPDQPPPSSSQFRQPSP
GTQNIGIVGSLNTSQISPGTISGPQKPRPGLPSSTPIPSGSQMPGSQRPPSQSLMRPMTVSSPSLASQQTPQSSSSTFRPQQRPQVSQP
RPQQSQLVTPSQQNTILTQQQQQQKQQQSASHQNQQIAAPKNQPQLSQHPTARTPISMTPKPDLPAIQNVAVLQSVDTAATDANA
SETGTRLLTKRSIHELVAQIDPNEKLDPEVEDVLIDIAEDFVESVTTFACSLAKHRKSSTLEAKDVLLHAERSWNITLPGFSGDEIKL
YKKQHINDIHRERLALIKKSMATDTRNSAAQAAANQKNQTPKPPAPASP

>ZmTAF12b

MADPPPVAASASPAQPDQLAAAASVSTPQNPNPLLSPQIPPSPTVSDLSAHISSPQQLDQAAAAADASGGGGSMDYLPRPPQLQA
PSPTQAGVGAAGFAQIPRSGSTSRLSTATQLQQYAARMYGGQMSFSGGGGLVGQQQLAGRAPMLGQGQLGMLQGQGNAASAA
HFGIQSQMLAQARQKVMAQNTQLNNANTAQVLQGMQPMGVMGAMGMNQTRPNVTIPYGAQQRFAQAQMRPQASQQPALSP
QKVPGQGLSRTASITALNSQLSGSSQNGQMVAMSMPQQQQQQQQWLKQMQPSMGSPLSPQQQYQNQQRLLLMQHLQQKTGL
NQQQLLQIQQQHPHLNVQQLIKQQQFLKQFQQQQPLQSPRVLASGSQKSANLTGSQPGTPLSGGTMTGGSGSQGAEGTSQLLGK
RKIQDLVAQVDPLGKVDPEVEDLLLEIADDFIGSVTAFACTLAKHRKSSVVEAKDVLLHLEKNWNLSIPGFSRGDKNPQRNSAKQ
LVDPQHSESDVTGIRGTSNKLGANNSVGNHQIRPPMAEPSAMPTMGPLSKAPRF

>ZmTAF13

MQNPSGHHTATAAPSSAPSKGKSSAPNPSGNHAATPAPSGTPSKGKSAAAQAAALGHGSSSHHHSAGGGGGGADASATTLKRK
RGVFQKDLQHMMYGFGDDPNPLPETVALVEDIVVEYVTDLVHKAQNVASKRGKLLTEDFLYLIRKDLRKLHRATELLSMNEELK
QARKAFDVDEETLATTNI

>ZmTAF14b

MAQSGRPCSFELSLSCFSRLTPVPPACLRALSASWRRRRPPPGGQAIVGSYSSAPSPVMSTQNKRLKDVEVSFPIVYGTISFWLGK
KASEYNSHKWTVYVRSANNEDLSVIVKRAVFQLHPSFQNPTRVVEQPPFELSESGWGEFEIAITLYFHSDVCDKRLDLFHQLKLY
PEEEAGPQSTKKPVVVETYDEIVFPEPTEAFFLRVQNHPAANVPRLPPGITLPPPGPMEIVPYEKKRGDTKDHPLSQWFSNFSEAD
ELLKLAAARQQSLDSSLGTSNVVHLVCKEDVGMEPFSNCLTDWVLCIRIEVAALGLQMTCIFYEV

>ZmTAF15

MVLLFHNSVVLLQMAGYMSRGPQNGSVYVCNLPPGTDENMLAEYFGTIGLLKKDKRTGRPKIWIYRDKVTNEPKGDATVTYE
DPHAASAAVEWFDNKDFHGSTIQVHIAESKSKDAFDNPTSLNIIGGVGEQDELDNGAGRGRGRGDGPGKAWQQDGDWMCPNT
SCGNVNFAFRGVCNRCGAARPAGAGGTAAGGGGRGRGRGSSDARGSSHAGAAVGGPPGLFGPNDWPCPMCGNINWAKRTKC

NICNTSKPGTNEGGVRGGRGGGYKELDEEELEEVKKRRKEAEEDDGEIYDEFGNLKKKFRSKALHTEGAQALPGSGRAGWEVE
HRGPSEREGRERSRDRVRDDYYEKETRGRDRGDLGRDQRRSRSRSRDRERERRERRREHDYERRERDRDRDRRHR

>ZmTAF15b

MSASYGSEDYRGGGGGGGYGGRGGGGGRGRGGGGGYGGGGGGGGYGGGGSGGYGGGGGGGGRGSGGGGFGGGGRGGGG
GGRGGGGRGGGREGDWVCPDASCGNVNFARRAECNKCGAPCPSGGGGGGGGGYNRSGGGGGGYNRGSGDYGSGGGGFDRD
GGDYNSGGRGGGSGGGGGYNRSGGSDRGFDDHRGGRGGSYGGRDQENQRGSEGGYSAGGYGQAPPQAPPSYGGPAGDYAAP
PSSYGGNNAYGSDSAVPPPNSYSGGPGSYPPSYGAPPPHQYGGAPGGQGGLPPTYDGGYGGRSMPGGGGSGGAPPPYHGGGSG
YTGSADPEPAGKVKQCDENCDETCDNARIYISNLPPDVTVEELQELFGGIGQVGRIKQKRGYKDQWPWNIKIYADDSGKAKGD
ACLAYEDPSAAHSAGGFYNNYDMRGYKISVVMAEKSAPRAAPSYGHGGGRGGGRGGGGRRDNYRDGGGHGPNRNQGGGSR
SRPY

VIII. Protein sequences from *Homo sapiens*

>HsTBP

MAHHHHHHSSGLEVLFQGPSGIVPQLQNIVSTVNLGCKLDLKTIALRARNAEYNPKRFAAVIMRIREPRTTALIFSSGKMVCTGA
KSEEQSRLAARKYARVVQKLGFPAKFLDFKIQNMVGSCDVKFPIRLEGLVLTHQQFSSYEPELFPGLIYRMIKPRIVLLIFVSGKVV
LTGAKVRAEIYEAFENIYPILKGFRKTT

>HsTAF1

MSDTDSDEDSAGGGPFSLAGFLFGNINGAGQLEGESVLDDECKKHLAGLGALGLGSLITELTANEELTGTDGALVNDEGWVRST
EDAVDYSDINEVAEDESRRYQQTMGSLQPLCHSDYDEDDYDADCEDIDCKLMPPPPPPPGPMKKDKDQDSITGVSENGEGIILPSI
IAPSSLASEKVDFSSSSDSESEMGPQEATQAESEDGKLTLPLAGIMQHDATKLLPSVTELFPEFRPGKVLRFLRLFGPGKNVPSVW
RSARRKRKKKHRELIQEEQIQEVECSVESEVSQKSLWNYDYAPPPPPEQCLSDDEITMMAPVESKFSQSTGDIDKVTDTKPRVAE
WRYGPARLWYDMLGVPEDGSGFDYGFKLRKTEHEPVIKSRMIEEFRKLEENNGTDLLADENFLMVTQLHWEDDIIWDGEDVK
HKGTKPQRASLAGWLPSSMTRNAMAYNVQQGFAATLDDDKPWYSIFPIDNEDLVYGRWEDNIIWDAQAMPRLLEPPVLTLDPN
DENLILEIPDEKEEATSNSPSKESKKESSLKKSRILLGKTGVIKEEPQQNMSQPEVKDPWNLSNDEYYYPKQQGLRGTFGGNIIQH
SIPAVELRQPFFPTHMGPIKLRQFHRPPLKKYSFGALSQPGPHSVQPLLKHIKKKAKMREQERQASGGGEMFFMRTPQDLTGKDG
DLILAEYSEENGPLMMQVGMATKIKNYYKRKPGKDPGAPDCKYGETVYCHTSPFLGSLHPGQLLQAFENNLFRAPIYLHKMPE
TDFLIIRTRQGYYIRELVDIFVVGQQCPLFEVPGPNSKRANTHIRDFLQVFIYRLFWKSKDRPRRIRMEDIKKAFPSHSESSIRKRLK
LCADFKRTGMDSNWWVLKSDFRLPTEEEIRAMVSPEQCCAYYSMIAAEQRLKDAGYGEKSFFAPEEENEEDFQMKIDDEVRTAP
WNTTRAFIAAMKGKCLLEVTGVADPTGCGEGFSYVKIPNKPTQQKDDKEPQPVKKTVTGTDADLRRLSLKNAKQLLRKFGVPE
EEIKKLSRWEVIDVVRTMSTEQARSGEGPMSKFARGSRFSVAEHQERYKEECQRIFDLQNKVLSSTEVLSTDTDSSSAEDSDFEE
MGKNIENMLQNKKTSSQLSREREEQERKELQRMLLAAGSAASGNNHRDDDTASVTSLNSSATGRCLKIYRTFRDEEGKEYVRC
ETVRKPAVIDAYVRIRTTKDEEFIRKFALFDEQHREEMRKERRRIQEQLRRLKRNQEKEKLKGPPEKKPKKMKERPDLKLKCGAC
GAIGHMRTNKFCPLYYQTNAPPSNPVAMTEEQEEELEKTVIHNDNEELIKVEGTKIVLGKQLIESADEVRRKSLVLKFPKQQLPPK
KKRRVGTTVHCDYLNRPHKSIHRRRTDPMVTLSSILESIINDMRDLPNTYPFHTPVNAKVVKDYYKIITRPMDLQTLRENVRKRL
YPSREEFREHLELIVKNSATYNGPKHSLTQISQSMLDLCDEKLKEKEDKLARLEKAINPLLDDDDQVAFSFILDNIVTQKMMAVPD
SWPFHHPVNKKFVPDYYKVIVNPMDLETIRKNISKHKYQSRESFLDDVNLILANSVKYNGPESQYTKTAQEIVNVCYQTLTEYD
EHLTQLEKDICTAKEAALEEAELESLDPMTPGPYTPQPPDLYDTNTSLSMSRDASVFQDESNMSVLDIPSATPEKQVTQMRQGRG
RLGEEDSDVDIEGYDDEEEDGKPKTPAPEGEDGDGDLADEEEGTVQQPQASVLYEDLLMSEGEDDEEDAGSDEEGDNPFSAIQL
SESGSDSDVGSGGIRPKQPRMLQENTRMDMENEESMMSYEGDGGEASHGLEDSNISYGSYEEPDPKSNTQDTSFSSIGGYEVSE
EEEDEEEEEQRSGPSVLSQVHLSEDEEDSEDFHSIAGDSDLDSDE

>HsTAF2

MPLTGVEPARMNRKKGDKGFESPRPYKLTHQVVCINNINFQRKSVVGFVELTIFPTVANLNRIKLNSKQCRIYRVRINDLEAAFIY
NDPTLEVCHSESKQRNLNYFSNAYAAAVSAVDPDAGNGELCIKVPSELWKHVDELKVLKIHINFSLDQPKGGLHFVVPSVEGSM
AERGAHVFSCGYQNSTRFWFPCVDSYSELCTWKLEFTVDAAMVAVSNGDLVETVYTHDMRKKTFHYMLTIPTAASNISLAIGPF
EILVDPYMHEVTHFCLPQLLPLLKHTTSYLHEVFEFYEEILTCRYPYSCFKTVFIDEAYVEVAAYASMSIFSTNLLHSAMIIDETPLT
RRCLAQSLAQQFFGCFISRMSWSDEWVLKGISGYIYGLWMKKTFGVNEYRHWIKEELDKIVAYELKTGGVLLHPIFGGGKEKDN
PASHLHFSIKHPHTLSWEYYSMFQCKAHLVMRLIENRISMEFMLQVFNKLLSLASTASSQKFQSHMWSQMLVSTSGFLKSISNVS
GKDIQPLIKQWVDQSGVVKFYGSFAFNRKRNVLELEIKQDYTSPGTQKYVGPLKVTVQELDGSFNHTLQIEENSLKHDIPCHSKS
RRNKKKKIPLMNGEEVDMDLSAMDADSPLLWIRIDPDMSVLRKVEFEQADFMWQYQLRYERDVVAQQESILALEKFPTPASRL
ALTDILEQEQCFYRVRMSACFCLAKIANSMVSTWTGPPAMKSLFTRMFCCKSCPNIVKTNNFMSFQSYFLQKTMPVAMALLRDV
HNLCPKEVLTFILDLIKYNDNRKNKFSDNYYRAEMIDALANSVTPAVSVNNEVRTLDNLNPDVRLILEEITRFLNMEKLLPSYRHT
ITVSCLRAIRVLQKNGHVPSDPALFKSYAEYGHFVDIRIAALEAVVDYTKVDRSYEELQWLLNMIQNDPVPYVRHKILNMLTKNP
PFTKNMESPLCNEALVDQLWKLMNSGTSHDWRLRCGAVDLYFTLFGLSRPSCLPLPELGLVLNLKEKKAVLNPTIIPESVAGNQE
AANNPSSHPQLVGFQNPEDDHLAKEASCNISAHQQGVKRKSDTPLGSPLEPGQILEKNEDSSKVKLKIRFSSSQDEEEIDMDTVH
DSQAFISHHLNMLERPSTPGLSKYRPASSRSALIPQHSAGCDSTPTTKPQWSLELARKGTGKEQAPLEMSMHPAASAPLSVFTKES
TASKHSDHHHHHHHEHKKKKKKHKHKHKHKHKHDSKEKDKEPFTFSSPASGRSIRSPSLSD

>HsTAF4b

MPAGLTEPAGAAPPAAVSASGTVTMAPAGALPVRVESTPVALGAVTKAPVSVCVEPTASQPLRSPVGTLVTKVAPVSAPPKVSSGP
RLPAPQIVAVKAPNTTTIQFPANLQLPPGTVLIKSNSGPLMLVSPQQTVTRAETTSNITSRPAVPANPQTVKICTVPNSSSQLIKKVAV
TPVKKLAQIGTTVVTTVPKPSSVQSVAVPTSVVTVTPGKPLNTVTTLKPSSLGASSTPSNEPNLKAENSAAVQINLSPTMLENVKK
CKNFLAMLIKLACSGSQSPEMGQNVKKLVEQLLDAKIEAEEFTRKLYVELKSSPQPHLVPFLKKSVVALRQLLPNSQSFIQQCVQ
QTSSDMVIATCTTTVTTSPVVTTTVSSSQSEKSIIVSGATAPRTVSVQTLNPLAGPVGAKAGVVTLHSVGPTAATGGTTAGTGLLQ
TSKPLVTSVANTVTTVSLQPEKPVVSGTAVTLSLPAVTFGETSGAAICLPSVKPVVSFCWDHICKPVIGTPVQIKLAQPGPVLSQPA
GIPTGSSSKQLFSLFHVVQQPSGGNEKQVTTISHSSTLTIQKCGQKTMPVNTIIPTSQFPPASILKQITLPGNKILSLQASPTQKNRIK
ENVTSCFRDEDDINDVTSMAGVNLNEENACILATNSELVGTLIQSCKDEPFLFIGALQKRILDIGKKHDITELNSDAVNLISQATQE
RLRGLLEKLTAIAQHRMTTYKASENYILCSDTRSQLKFLEKLDQLEKQRKDLEEREMLLKAAKSRSNKEDPEQLRLKQKAKELQ
QLELAQIQHRDANLTALAAIGPRKKRPLESGIEGLKDNLLASGTSSLTATKQLHRPRITRICLRDLIFCMEQEREMKYSRALYLALL
K

>HsTAF5

MAALAEEQTEVAVKLEPEGPPTLLPPQAGDGAGEGSGGTTNNGPNGGGGNVAASSSTGGDGGTPKPTVAVSAAAPAGAAPVPA
AAPDAGAPHDRQTLLAVLQFLRQSKLREAEEALRREAGLLEEAVAGSGAPGEVDSAGAEVTSALLSRVTASAPGPAAPDPPGTG
ASGATVVSGSASGPAAPGKVGSVAVEDQPDVSAVLSAYNQQGDPTMYEEYYSGLKHFIECSLDCHRAELSQLFYPLFVHMYLEL
VYNQHENEAKSFFEKFHGDQECYYQDDLRVLSSLTKKEHMKGNETMLDFRTSKFVLRISRDSYQLLKRHLQEKQNNQIWNIVQ
EHLYIDIFDGMPRSKQQIDAMVGSLAGEAKREANKSKVFFGLLKEPEIEVPLDDEDEEGENEEGKPKKKKPKKDSIGSKSKKQDP
NAPPQNRIPLPELKDSDKLDKIMNMKETTKRVRLGPDCLPSICFYTFLNAYQGLTAVDVTDDSSLIAGGFADSTVRVWSVTPKKL
RSVKQASDLSLIDKESDDVLERIMDEKTASELKILYGHSGPVYGASFSPDRNYLLSSSEDGTVRLWSLQTFTCLVGYKGHNYPVW
DTQFSPYGYYFVSGGHDRVARLWATDHYQPLRIFAGHLADVNCTRFHPNSNYVATGSADRTVRLWDVLNGNCVRIFTGHKGPIH
SLTFSPNGRFLATGATDGRVLLWDIGHGLMVGELKGHTDTVCSLRFSRDGEILASGSMDNTVRLWDAIKAFEDLETDDFTTATGH
INLPENSQELLLGTYMTKSTPVVHLHFTRRNLVLAAGAYSPQ

>HsTAF6

MAEEKKLKLSNTVLPSESMKVVAESMGIAQIQEETCQLLTDEVSYRIKEIAQDALKFMHMGKRQKLTTSDIDYALKLKNVEPLY
GFHAQEFIPFRFASGGGRELYFYEEKEVDLSDIINTPLPRVPLDVCLKAHWLSIEGCQPAIPENPPPAPKEQQKAEATEPLKSAKPG
QEEDGPLKGKGQGATTADGKGKEKKAPPLLEGAPLRLKPRSIHELSVEQQLYYKEITEACVGSCEAKRAEALQSIATDPGLYQML
PRFSTFISEGVRVNVVQNNLALLIYLMRMVKALMDNPTLYLEKYVHELIPAVMTCIVSRQLCLRPDVDNHWALRDFAARLVAQIC
KHFSTTTNNIQSRITKTFTKSWVDEKTPWTTRYGSIAGLAELGHDVIKTLILPRLQQEGERIRSVLDGPVLSNIDRIGADHVQSLLL
KHCAPVLAKLRPPPDNQDAYRAEFGSLGPLLCSQVVKARAQAALQAQQPRPTLTLSQAPQPGPRTPGLLKVPGSIALPVQTLVSA
RAAAPPQPSPPPTKFIVMSSSSSAPSTQQVLSLSTSAPGSGSTTTSPVTTTVPSVQPIVKLVSTATTAPPSTAPSGPGSVQKYIVVSLP
PTGEGKGGPTSHPSPVPPPASSPSPLSGSALCGGKQEAGDSPPPAPGTPKANGSQPNSGSPQPAP

>HsTAF7

MSKSKDDAPHELESQFILRLPPEYASTVRRAVQSGHVNLKDRLTIELHPDGRHGIVRVDRVPLASKLVDLPCVMESLKTIDKKTFY
KTADICQMLVSTVDGDLYPPVEEPVASTDPKASKKKDKDKEKKFIWNHGITLPLKNVRKRRFRKTAKKKYIESPDVEKEVKRLLS
TDAEAVSTRWEIIAEDETKEAENQGLDISSPGMSGHRQGHDSLEHDELREIFNDLSSSSEDEDETQHQDEEDINIIDTEEDLERQLQ
DKLNESDEQHQENEGTNQLVMGIQKQIDNMKGKLQETQDRAKRQEDLIMKVENLALKNRFQAVLDELKQKEDREKEQLSSLQ
EELESLLEK

>HsTAF8

MADAAATAGAGGSGTRSGSKQSTNPADNYHLARRRTLQVVVSSLLTEAGFESAEKASVETLTEMLQSYISEIGRSAKSYCEHTAR
TQPTLSDIVVTLVEMGFNVDTLPAYAKRSQRMVITAPPVTNQPVTPKALTAGQNRPHPPHIPSHFPEFPDPHTYIKTPTYREPVSDY
QVLREKAASQRRDVERALTRFMAKTGETQSLFKDDVSTFPLIAARPFTIPYLTALLPSELEMQQMEETDSSEQDEQTDTENLALHI
SMEDSGAEKENTSVLQQNPSLSGSRNGEENIIDNPYLRPVKKPKIRRKKSLS

>HsTAF9

MESGKTASPKSMPKDAQMMAQILKDMGITEYEPRVINQMLEFAFRYVTTILDDAKIYSSHAKKATVDADDVRLAIQCRADQSFT
SPPPRDFLLDIARQRNQTPLPLIKPYSGPRLPPDRYCLTAPNYRLKSLQKKASTSAGRITVPRLSVGSVTSRPSTPTLGTPTPQTMSV
STKVGTPMSLTGQRFTVQMPTSQSPAVKASIPATSAVQNVLINPSLIGSKNILITTNMMSSQNTANESSNALKRKREDDDDDDDDD
DDYDNL

>HsTAF10

MSCSGSGADPEAAPASAASAPGPAPPVSAPAALPSSTAAENKASPAGTAGGPGAGAAAGGTGPLAARAGEPAERRGAAPVSAGG
AAPPEGAISNGVYVLPSAANGDVKPVVSSTPLVDFLMQLEDYTPTIPDAVTGYYLNRAGFEASDPRIIRLISLAAQKFISDIANDAL
QHCKMKGTASGSSRSKSKDRKYTLTMEDLTPALSEYGINVKKPHYFT

>HsTAF11

MDDAHESPSDKGGETGESDETAAVPGDPGATDTDGIPEETDGDADVDLKEAAAEEGELESQDVSDLTTVEREDSSLLNPAAKKL
KIDTKEKKEKKQKVDEDEIQKMQILVSSFSEEQLNRYEMYRRSAFPKAAIKRLIQSITGTSVSQNVVIAMSGISKVFVGEVVEEAL
DVCEKWGEMPPLQPKHMREAVRRLKSKGQIPNSKHKKIIFF

>HsTAF12

MCWRAPREWRGRAAARAREEPLLPVAVSAGSFLSTAHDRRRLMALEYRLSAALIGLGPHSSKKKQDLDKLYELKSKARQIMNQ
FGPSALINLSNFSSIKPEPASTPPQGSMANSTAVVKIPGTPGAGGRLSPENNQVLTKKKLQDLVREVDPNEQLDEDVEEMLLQIAD
DFIESVVTAACQLARHRKSSTLEVKDVQLHLERQWNMWIPGFGSEEIRPYKKACTTEAHKQRMALIRKTTKK

>HsTAF13

MADEEEDPTFEEENEEIGGGAEGGQGKRKRLFSKELRCMMYGFGDDQNPYTESVDILEDLVIEFITEMTHKAMSIGRQGRVQVE
DIVFLIRKDPRKFARVKDLLTMNEELKRARKAFDEANYGS

>HsTAF15

MSDSGSYGQSGGEQQSYSTYGNPGSQGYGQASQSYSGYGQTTDSSYGQNYSGYSSYGQSYSQSYGGYENQKQSSYSQQPYNN
QGQQQNMESSGSQGGRAPSYDQPDYGQQDSYDQQSGYDQHQGSYDEQSNYDQQHDSYSQNQQSYHSQRENYSHHTQDDRR
DVSRYGEDNRGYGGSQGGGRGRGGYDKDGRGPMTGSSGGDRGGFKNFGGHRDYGPRTDADSESDNSDNNTIFVQGLGEGVS
TDQVGEFFKQIGIIKTNKKTGKPMINLYTDKDTGKPKGEATVSFDDPPSAKAAIDWFDGKEFHGNIIKVSFATRRPEFMRGGGSG
GGRRGRGGYRGRGGFQGRGGDPKSGDWVCPNPSCGNMNFARRNSCNQCNEPRPEDSRPSGGDFRGRGYGGERGYRGRGGRG
YGGDRGGYGGDRSRGGYGGDRGGGSGYGGDRSGGYGGDRSGGGYGGDRGGGYGGDRGGYGGKMGGRNDYRNDQRNRPY
CLLLSSWPLLG

IX. Protein sequences from *Saccharomyces cerevisiae*

>ScTBP

MSGIVPTLQNIVATVTLGCRLDLKTVALHARNAEYNPKRFAAVIMRIREPKTTALIFASGKMVVTGAKSEDDSKLASRKYARIIQK
IGFAAKFTDFKIQNIVGSCDVKFPIRLEGLAFSHGTFSSYEPELFPGLIYRMVKPKIVLLIFVSGKIVLTGAKQREEIYQAFEAIYPVL
SEFRKM

>ScTaf1p

MVKQQGSGKTNLANEDEAYEAIFGGEFGSLEIGSYIGGDEGANSKDYTEHLPDAVDFEDEDELADDDDDLPEESDANLHPAMM
TMGAYDDVNENGAVLGIDSNSLNMQLPEINGDLSQQFILEDDGGTPATSNALFMGMDANEIHLATETGVLDGSGANEIGHSQLSI
GGVNGNDMSINGGFIMEPDMSDGKHKKATKLDLINHEKYLLKKYFPDFEKGKILKWNKLIYRRSVPYHWHSEISRVKKPFMPL
NLKFKVQQDDKRLFNSRTISYVAPIYQGKNNLLQSNSSASRRGLIHVSIDELFPIKEQQKKRKIIHDEKTISEDLLIATDDWDQEKII
NQGTSSTATLADSSMTPNLKFSGGYKLKSLIEDVAEDWQWDEDMIIDAKLKESKHAELNMNDEKLLLMVEKTNNLAQQKQQL
DSSNLILPLNETILQQKFNLSNDDKYQILKKTHQTKVRSTISNLNIQHSQPAINLQSPFYKVAVPRYQLRHFHRENFGSHIRPGTKIV
FSKLKARKRKRDKGKDVKESFSTSQDLTIGDTAPVYLMEYSEQTPVALSKFGMANKLINYYRKANEQDTLRPKLPVGETHVLGV
QDKSPFWNFGFVEPGHIVPTLYNNMIRAPVFKHDISGTDFLLTKSSGFGISNRFYLRNINHLFTVGQTFPVEEIPGPNSRKVTSMKA
TRLKMIIYRILNHNHSKAISIDPIAKHFPDQDYGQNRQKVKEFMKYQRDGPEKGLWRLKDDEKLLDNDAVKSLITPEQISQVESM
SQGLQFQEDNEAYNFDSKLKSLEENLLPWNITKNFINSTQMRAMIQIHGVGDPTGCGEGFSFLKTSMKGGFVKSGSPSSNNNSSN
KKGTNTHSYNVAQQQKAYDEEIAKTWYTHTKSLSISNPFEEMTNPDEINQTNKHVKTDRDDKKILRIVRKKRDENGIIQRQTIFIR
DPRVIQGYIKIKEQDKEDVNKLLEEDTSKINNLEELEKQKKLLQLELANLEKSQQRRAARQNSKRNGGATRTENSVDNGSDLAG
VTDGKAAKNKGKNTTRRCATCGQIGHIRTNKSCPMYSSKDNPASPK

>ScTaf2p

MMSFSKNATPRAIVSESSTLHEMKFRNFRVAHEKISLDIDLATHCITGSATIIIIPLIQNLEYVTFDCKEMTIKDVLVENRRCDQFIHD
DPLQTNLNGLTSQNVLYSDNSIEQSHFLRSKFASLNEYPETDSKSQLTIKIPSSIKISLEDANALSNYTPITPSIKTTPGFQESVFTPITL
QIEYEIRNPKSGIKFDTVYADKPWLWNVYTSNGEICSSASYWVPCVDLLDEKSTWELEFSVPRLVKNIGTSKLIGQNGEESEKEKE
DTPEHDEEEEGKPARVIKDEDKDSNLKNDEEGKNSKSKDAQDNDEEEEEGESDEEEEEGEEERRNIEESNNPSLRDVIVCCSEYS
NIKELPHPIDLTKKKCIFQIINPVAPHHIGWAIGAFNSWSLPLISPPSVDAEDEVEEDKLRENVVDNVNDTMDDDIGSDIIPIQIFTLP
TQETDELTVINSTVVCQKIIDFYSKEFGSYPFTCYSMVFLPTAPSKHMDFAALGICNTRLLYPLEVIDKAFSTTNELAWALANQWS
CVNITPLDMNDYWCCLGIAGYMVFQVTKKLMGNNTYKYQLKRNSEAIVEQDFEKPPIGSTFTGSSRPISWSSKDLSFIQLKAPMI
LHILDRRMTKTERSFGMSRVLPKIFLQAMSGDLPNNSLTSSHFQHVCERVNKSKLENFFNEWVYGSGVPILRVTQRFNRKRMVIE
LGIRQVQDEELGHEKVVGEEGFFKSALDHLEHPDLNRTECFTGSMTIRIHEHDGTPYEHIVEIKDTFTKIDIQYNTKYRRLRKRGG
GANDENGVENNNEEKPIVVDVNCLGNVYMSPEECSRFSLTEFNRTSESNELLKQNEAFEWIRIDSDLEWICQMHINQPDYMFSSQ
LRQDGDIEAQLEAIRYYEDVVVNGGVKSLVYSSILFRTAIDERYFFGIRLAACEALSKYVYDPDFTGGVKHLIQIFQILFCLEDSNIP
KSNNFENPKLYFLQCNIPKYLAKVKNENGKCPKLVKQFLLDILVYNENGENKYSDDAYVRSLIENVVKVALNEYKDKAYMEKV
KTQLLRYENLVNWLSSYESLIKTTIMYAKYKLHKVGAYDFTELTGMIMHTLTLGINNGDISRESFQNEFLMVLKIMLLEGGLKNK
DALVLFTEILCFHEDSYIRDKSVDVLSECVNLVVMDGSLDTISDDIKSSVQSVHNEVKNIKSEDDIELFLSGHYVDDMKIKIEKIGR
QNISGLIQICRDMFKGYSPLKILLWDVLNLPVLSLYQRKQIHDLVRVMYTLINSFVVRLETPRERRLVAKMNSNEEGKLDIVIKRES
ILKVHIKKEVTSTVEAPKKANKIKISLKGDKPVRKVEKQIVKPKVTSKQRKVKSHVNRMGSLPLRFVKIQQQPRVMVHLSSVPYS
QFVQITKVTSRSFMVKIRTKNDAKN

>ScTaf5p

MSQKQSTNQNQNGTHQPQPVKNQRTNNAAGANSGQQPQQQSQGQSQQQGRSNGPFSASDLNRIVLEYLNKKGYHRTEAMLR
AESGRTLTPQNKQSPANTKTGKFPEQSSIPPNPGKTAKPISNPTNLSSKRDAEGGIVSSGRLEGLNAPENYIRAYSMLKNWVDSSLE
IYKPELSYIMYPIFIYLFLNLVAKNPVYARRFFDRFSPDFKDFHGSEINRLFSVNSIDHIKENEVASAFQSHKYRITMSKTTLNLLLYF
LNENESIGGSLIISVINQHLDPNIVESVTAREKLADGIKVLSDSENGNGKQNLEMNSVPVKLGPFPKDEEFVKEIETELKIKDDQEK
QLNQQTAGDNYSGANNRTLLQEYKAMNNEKFKDNTGDDDKDKIKDKIAKDEEKKESELKVDGEKKDSNLSSPARDILPLPPKT
ALDLKLEIQKVKESRDAIKLDNLQLALPSVCMYTFQNTNKDMSCLDFSDDCRIAAAGFQDSYIKIWSLDGSSLNNPNIALNNND
KDEDPTCKTLVGHSGTVYSTSFSPDNKYLLSGSEDKTVRLWSMDTHTALVSYKGHNHPVWDVSFSPLGHYFATASHDQTARLW
SCDHIYPLRIFAGHLNDVDCVSFHPNGCYVFTGSSDKTCRMWDVSTGDSVRLFLGHTAPVISIAVCPDGRWLSTGSEDGIINVWDI
GTGKRLKQMRGHGKNAIYSLSYSKEGNVLISGGADHTVRVWDLKKATTEPSAEPDEPFIGYSGDVTASINQDIKEYGRRRTVIPT
SDLVASFYTKKTPVFKVKFSRSNLALAGGAFRP

>ScTaf6p

MSTQQQSYTIWSPQDTVKDVAESLGLENINDDVLKALAMDVEYRILEIIEQAVKFKRHSKRDVLTTDDVSKALRVLNVEPLYGY
YDGSEVNKAVSFSKVNTSGGQSVYYLDEEEVDFDRLINEPLPQVPRLPTFTTHWLAVEGVQPAIIQNPNLNDIRVSQPPFIRGAIVT
ALNDNSLQTPVTSTTASASVTDTGASQHLSNVKPGQNTEVKPLVKHVLSKELQIYFNKVISTLTAKSQADEAAQHMKQAALTSL
RTDSGLHQLVPYFIQFIAEQITQNLSDLQLLTTILEMIYSLLSNTSIFLDPYIHSLMPSILTLLLAKKLGGSPKDDSPQEIHEFLERTNA
LRDFAASLLDYVLKKFPQAYKSLKPRVTRTLLKTFLDINRVFGTYYGCLKGVSVLEGESIRFFLGNLNNWARLVFNESGITLDNIE
EHLNDDSNPTRTKFTKEETQILVDTVISALLVLKKDLPDLYEGKGEKVTDEDKEKLLERCGVTIGFHILKRDDAKELISAIFFGE

>ScTaf7p

MAVIRIKKPRGPGEKDQPLEGEPKLKRIRIKTKVTDEDIKPKPKLKINLKKKKESADGKEKKNSLKLKLNLKKNEEPVKKIHKAP
KLRLKPIRIPGEAYDSEASDIEDDPLIESGVILRILPDIQLEFVKNSLESGDYSGISIKWKNERHAVVTINDVMYGAILVDLPTVIEVN
KSVDRKNLLKTFDVSQMLLCIRPIQEEEEVYALEAPDTEDLVVKHFEDIEDEIWENKETFLKGYNGAPLSDMEAKHLKEIALKGY
DYKHGISPPLYNVRNRRFRRKMDPNEIDYVEKVVDMLLKQDKQAEEVSYDLVDKSELHAKQERVSSWENFKEEPGEPLSRPAL
KKEEIHTIASAVGKQGAEEEGEEGMEEEEEEDLDLGAAFESEEEGSGAEGDKEQQQEEVGDEVDQDTGGEDDDDDDDGDIEAA
GGESESDDEKDENRQHTELLADELNELETTLAHTKHKLSKATNPLLKSRFIDSIKKLEKEAELKRKQLQQTEDSVQKQHQHRSD
AETANNVEEEEEEEEEEEEEEEEEEDEVDEDEEDDEENDEDEDNVHEREHVQENKVVRELDEAPAEETLDQNDLDMMMLFGA
EGDE

>ScTaf8p

MTSKTSESGTGTQSTIVQLRNLPDLTEISHLEIDAPVVEILKKTVLFQLNSLNICISNFALDELVNLVTVQMDGMFRNLHNLTLLQR
RSQASQADLKLLLREFNLDASSLYQQFQASEFIKSKHSTEYEKLMSWSSLAALPHNEEDEEDELNNIEEQQNEINVLLPPSNPLEK

QIPSWLPNFPPDHTYKFTPEFNHPITDLKTIKKEIVKESQESEKALLNLNKSLSHISSASNTPQPPGLDDEDAIEQQLEIWGNALEES
KPTITEKSFNENNIEQYAKYRVELARERVTKFEVNQLKRTKNPFLKISETLYLPEGPHQSHKTIQKTIELQFRKSMTLFMHNLPKV
QKLKKEKIRMAKEERAKSLKRRQEELISQRTKREQDEGHDLELLLNNEHARDAADDTTTPNALNNSTIVINTNAEDEDDDINLF
GILGSSEDENEMSSMPAENLVAESEPPTMTAQDTTNTTPVAHNTTNIDATTSHSPHSTPNENAPTSPPADIATDHDITM

>ScTaf9p

MNGGGKNVLNKNSVGSVSEVGPDSTQEETPRDVRLLHLLLASQSIHQYEDQVPLQLMDFAHRYTQGVLKDALVYNDYAGSGN
SAGSGLGVEDIRLAIAARTQYQFNPTAPKELMLQLAAERNKKALPQVMGTWGVRLPPEKYCLTAKEWDLEDPKSM

>ScTaf10p

MDFEEDYDAEFDDNQEGQLETPFPSVAGADDGDNDNDDSVAENMKKKQKREAVVDDGSENAFGIPEFTRKDKTLEEILEMMDS
TPPIIPDAVIDYYLTKNGFNVADVRVKRLLALATQKFVSDIAKDAYEYSRIRSSVAVSNANNSQARARQLLQGQQQPGVQQISQQQ
HQQNEKTTASKVVLTVNDLSSAVAEYGLNIGHPDFYR

>ScTaf11p

MTEPQGPLDTIPKVNYPPILTIANYFSTKQMIDQVISEDQDYVTWKLQNLRTGGTSINNQLNKYPKYKYQKTRINQQDLDSINKV
PENLIFPQDILQQQTQNSNYEDTNTNEDENEKLAQDEQFKLLVTNLDKDQTNRFEVFHRTSLNKTQVKKLASTVANQTISENIRV
FLQAVGKIYAGEIIELAMIVKNKWLTSQMCIEFDKRTKIGYKLKKISKKIDFLHHRKPAIQARLSIR

>ScTaf12p

MSSNPENSGVNANNNTGTGNADAITGAQQNMVLQPRQLQEMAAKFRTLLTEARNVGETTPRGKELMFQAAKIKQVYDALTLN
RRRQQAAQAYNNTSNPNSSNPASIPTENVPNSSQQQQQQQQQQQQTRNNSNKFSNMIKQVLTPEENQEYEKLWQNFQVRHTSI
KERETYLKQNIDRLEQEINKQTDEGPKQQLQEKKNELLNDWKVLKIEYTKLFNNYQNSKKTFYVECARHNPALHKFLQESTQQ
QRVQQQRVQQQQQQQQQQQQQQQQQQQQQQQQQQQQQQRQGQNQRKISSSNSTEIPSVTGPDALKSQQQQQNTIPATNNPR
GNVNTSQTEQSKAKVTNVNATASMLNNISSSKSAIFKQTEPAIPISENISTKTPAPVAYRSNRPTITGGSAMNASALNTPATTKLPPY
EMDTQRVMSKRKLRELVKTVGIDEGDGETVIDGDVEELLLDLADDFVTNVTAFSCRLAKHRKSDNLEARDIQLHLERNWNIRIP
GYSADEIRSTRKWNPSQNYNQKLQSITSDKVAAAKNNGNNVASLNTKK

>ScTaf12bp

MSSNPENSGVNANNNTGTGNADAITGAQQNMVLQPRQLQEMAAKFRTLLTEARNVGETTPRGKELMFQAAKIKQVYDALTLN
RRRQQAAQAYNNTSNPNSSNPASIPTENVPNSSQQQQQQQQQQTRNNSNKFSNMIKQVLTPEENQEYEKLWQNFQVRHTSIKEK
ETYLKQNIDRLEQEINKQTDEGPKQQLQEKKIELLNDWKVLKIEYTKLFNNYQNSKKTFYVECARHNPALHKFLQESTQQQRVQ
QQRVQQQQQQQQQQQQQQRQGQNQRKISSSNSTEIPSVTGPDALKSQQQQQNTIPATNNPRGNVNTSQTEQSKAKVTNVNAT
ASMLNNISSSKSAIFKQTEPAIPISENISTKTPAPVAYRSNRPTITGGSAMNASALNTPATTKLPPYEMDTQRVMSKRKLRELVKTV
GIDEGDGETVIDGDVEELLLDLADDFVTNVTAFSCRLAKHRKSDNLEARDIQLHLERNWNIRIPGYSADEIRSTRKWNPSQNYNQ
KLQSITSDKVAAAKNNGNNVASLNNKKIIMLPTFSTLPQKKFLKL

>ScTaf14p

MVATVKRTIRIKTQQHILPEVPPVENFPVRQWNIEIVLLDDEGKEIPATIFDKVIYHLHPTFANPNRTFTDPPFRIEEQGWGGFPLDIS
VFLLEKAGERKIPHDLNFLQESYEVEHVIQIPLNKPLLTEELAKSGSTEETTANTGTIGKRRTTTNTTAEPKAKRAKTGSASTVKGS
VDLEKLAFGLTKLNEDDLVGVVQMVTDNKTPEMNVTNNVEEGEFIIDLYSLPEGLLKSLWDYVKKNTE
