# Supplementary material for: Characterization of TBP and TAFs in Mungbean (Vigna radiata L.) and Their Potential Involvement in Abiotic Stress Response
Source: Int J Mol Sci. 2024 Sep 3;25(17):9558. doi: 10.3390/ijms25179558 (PMC11394781; doi:10.3390/ijms25179558)
Supplement: Supplementary file 1 [file ijms-25-09558-s001.zip › supplementary tables and figures.pdf]

Table S1. Sequence identity of TBP and TAFs between mungbean and Arabidopsis

Proteins in *Vigna radiate*	Protein Length (aa)	Homologous Protains in *A. thaliana*	Protein Length (aa)	Sequence identity
VrTBP	200	AtTBP1(AT3G13445)	200	92.5%
		AtTBP2(AT1G55520)	200	93.5%
VrTAF1 X1	1901	AtTAF1(AT1G32750)	1919	55.8%
VrTAF1 X2	1767			55.2%
VrTAF1 X3	1752			54.7%
VrTAF1 X4	1746			54.9%
VrTAF2-X1	1384	AtTAF2(AT1G73960)	1390	61.2%
VrTAF2-X2	1354			60.7%
VrTAF2-X3	939			67.2%
VrTAF4b-X1	936	AtTAF4b(AT5G43130)	852	51.3%
VrTAF4b-X2	936			51.3%
VrTAF4b-X3	936			51.3%
VrTAF4b-X4	936			51.3%
VrTAF4b-X5	932			51.4%
VrTAF4b-X6	845			51.3%
VrTAF4b-X7	845			51.3%
VrTAF5-1-X1	659	AtTAF5(AT5G25150)	669	71.4%
VrTAF5-1-X2	658			71.6%
VrTAF5-2	671			74.1%
VrTAF6-1	543	AtTAF6(AT1G04950)	549	65.9%
VrTAF6-2	536			56.1%
VrTAF6-like	192			70.6%
VrTAF7	199	AtTAF7(AT1G55300)	203	69.5%
VrTAF8-1	406	AtTAF8(AT4G34340)	353	36.4%
VrTAF8-2	290			26.0%
VrTAF8-like	350			46.8%
VrTAF9-1-X1	189	AtTAF9(AT1G54140)	183	69.7%
VrTAF9-1-X2	175			73.0%
VrTAF9-2	177			74.7%
VrTAF10	136	AtTAF10(AT4G31720)	134	82.1%
VrTAF11	204	AtTAF11(AT4G20280)	210	63.4%
VrTAF12	504	AtTAF12(AT3G10070)	539	52.8%
VrTAF12b	1064	AtTAF12b(AT1g17440)	683	57.3%
VrTAF13	136	AtTAF13(AT1g02680)	126	70.4%
VrTAF14b-1	279	AtTAF14b(AT5G45600)	268	66.0%
VrTAF14b-2	273			75.4%
VrTAF15-1	390	AtTAF15(AT1G50300)	372	73.1%
VrTAF15-2	156			34.4%
VrTAF15b-1	422	AtTAF15b(AT5G58470)	422	63.8%
VrTAF15b-2	524			69.8%

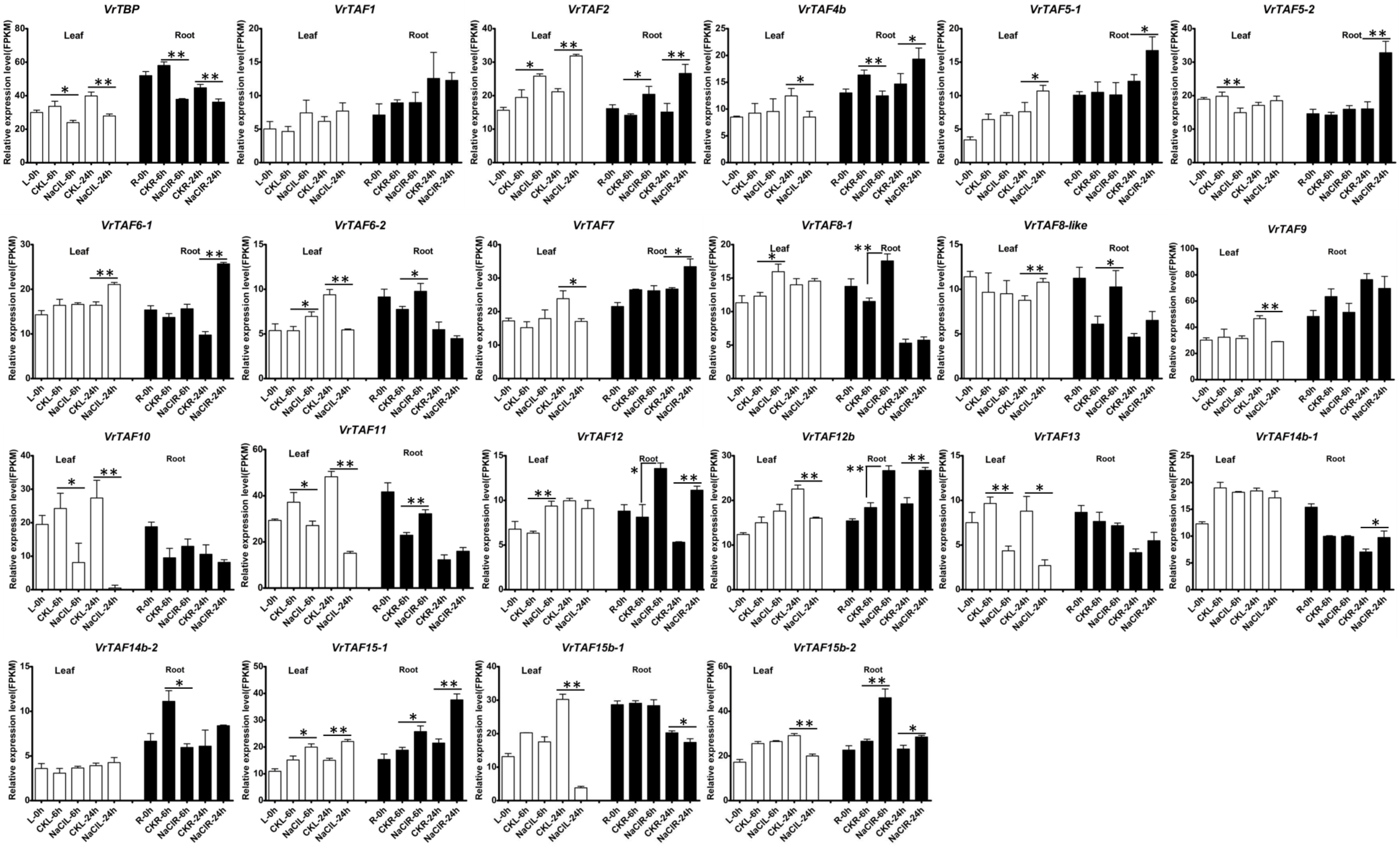

Figure S1. The expression of *VrTBP* and *VrTAFs* under high salinity stress.

Date were from RNA-seq. The difference significance analysis was performed between each treatment and its contemporaneous control group (CK). *, $p<0.05$; **, $p<0.01$.

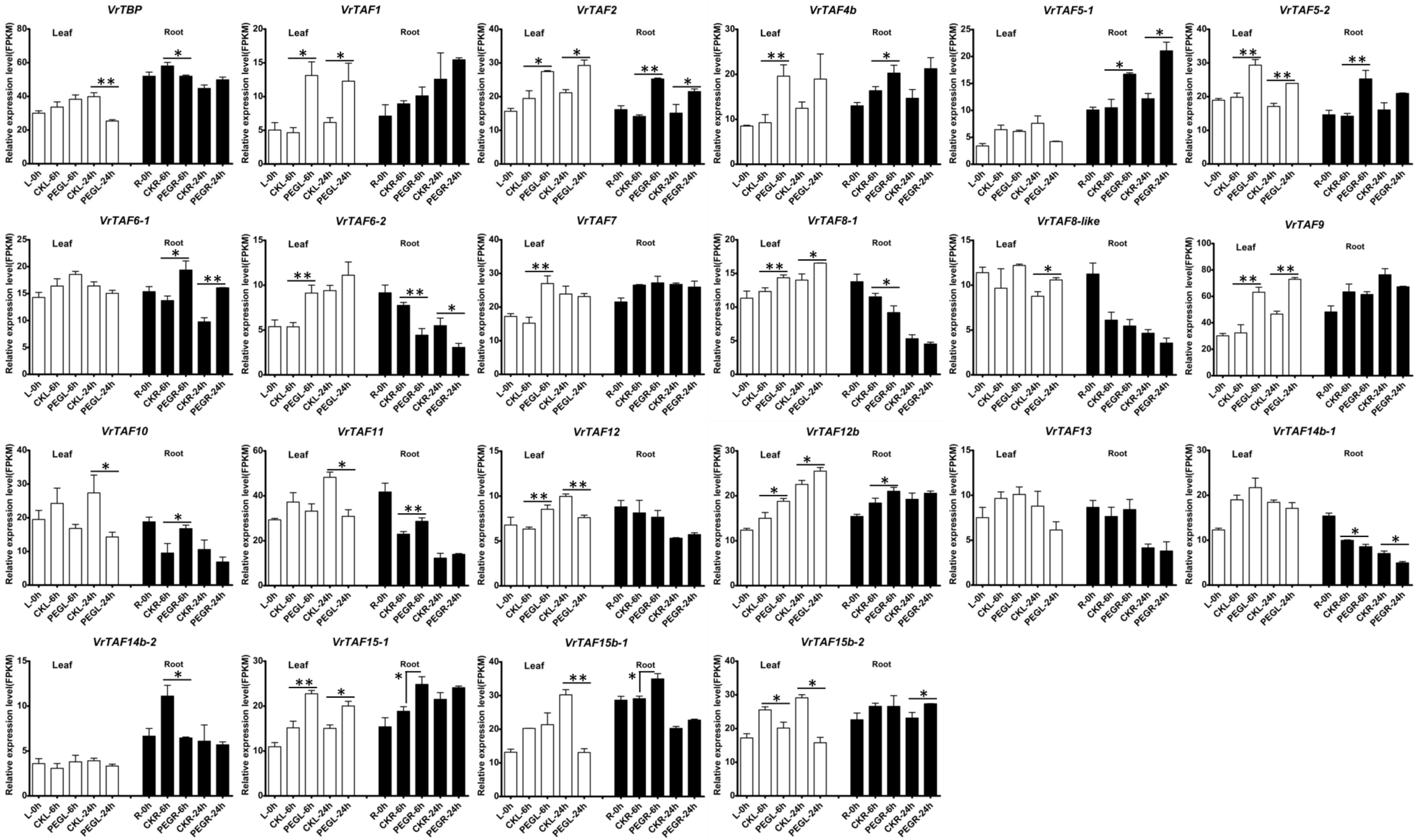

Figure S2. The expression of *VrTBP* and *VrTAFs* under drought stress.

Date were from RNA-seq. The difference significance analysis was performed between each treatment and its contemporaneous control group (CK). *, $p<0.05$; **, $p<0.01$.

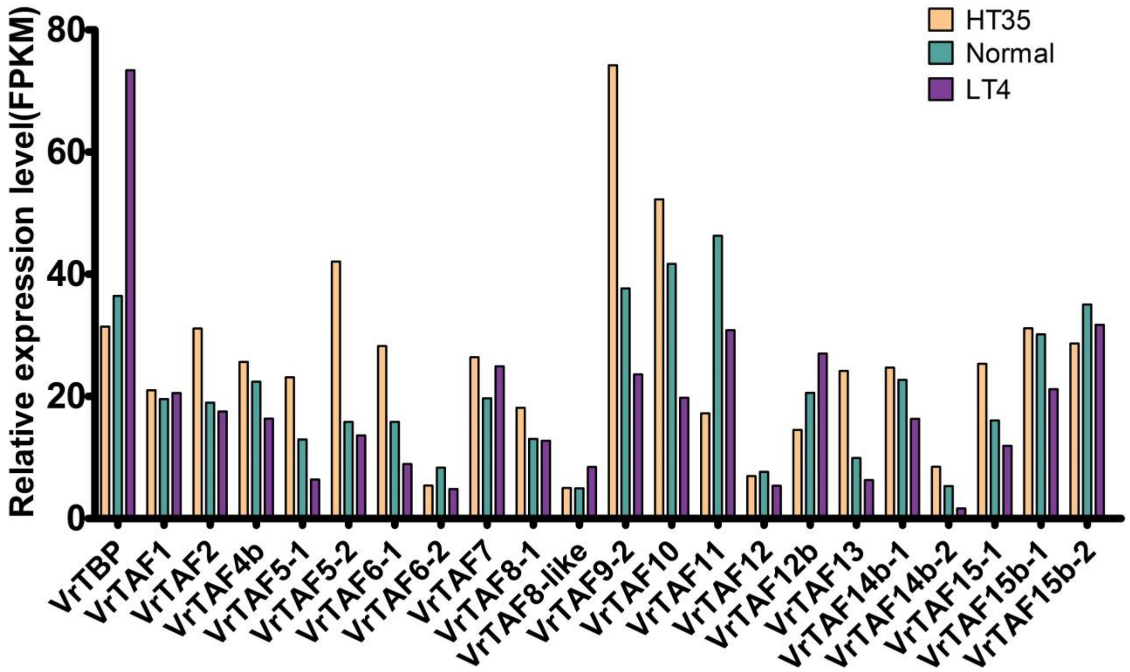

Figure S3. The expression of *VrTBP* and *VrTAFs* under heat and cold stress.
Date were from RNA-seq. HT35, high temperature 35 ℃; LT4, low temperature 4 ℃.
